# Integration of ubiquitination-related genes in predictive signatures for prognosis and immunotherapy response in sarcoma

**DOI:** 10.3389/fonc.2024.1446522

**Published:** 2024-10-14

**Authors:** Haotian Qin, Tiantian Qi, Juan Xu, Tianbing Wang, Hui Zeng, Jun Yang, Fei Yu

**Affiliations:** ^1^ Department of Bone & Joint Surgery, Peking University Shenzhen Hospital, Shenzhen Peking University-The Hong Kong University of Science and Technology Medical Center, Shenzhen, Guangdong, China; ^2^ National & Local Joint Engineering Research Center of Orthopaedic Biomaterials, Peking University Shenzhen Hospital, Shenzhen, Guangdong, China; ^3^ Shenzhen Key Laboratory of Orthopaedic Diseases and Biomaterials Research, Peking University Shenzhen Hospital, Shenzhen, Guangdong, China; ^4^ Department of Oncology, Chaohu Hospital of Anhui Medical University, Hefei, China; ^5^ Department of Orthopedics and Trauma, Peking University People’s Hospital, Beijing, China; ^6^ Department of Radiology, Peking University Shenzhen Hospital, Shenzhen, China

**Keywords:** ubiquitination, sarcoma, prognostic signatures, bioinformatics analysis, immunotherapy response

## Abstract

**Background:**

Ubiquitination is one of the most prevalent and complex post-translational modifications of proteins in eukaryotes, playing a critical role in regulating various physiological and pathological processes. Targeting ubiquitination pathways, either through inhibition or activation, holds promise as a novel therapeutic approach for cancer treatment. However, the expression patterns, prognostic significance, and underlying mechanisms of ubiquitination-related genes (URGs) in sarcoma (SARC) remain unclear.

**Methods:**

We analyzed URG expression patterns and prognostic implications in TCGA-SARC using public databases, identifying DEGs related to ubiquitination among SARC molecular subtypes. Functional enrichment analysis elucidated their biological significance. Prognostic signatures were developed using LASSO-Cox regression, and a predictive nomogram was constructed. External validation was performed using GEO datasets and clinical tissue samples. The association between URG risk scores and various clinical parameters, immune response, drug sensitivity, and RNA modification regulators was investigated. Integration of data from multiple sources and RT-qPCR confirmed upregulated expression of prognostic URGs in SARC. Single-cell RNA sequencing data analyzed URG distribution across immune cell types. Prediction analysis identified potential target genes of microRNAs and long non-coding RNAs.

**Results:**

We identified five valuable genes (CALR, CASP3, BCL10, PSMD7, PSMD10) and constructed a prognostic model, simultaneously identifying two URG-related subtypes in SARC. The UEGs between subtypes in SARC are mainly enriched in pathways such as Cell cycle, focal adhesion, and ECM-receptor interaction. Analysis of URG risk scores reveals that patients with a low-risk score have better prognoses compared to those with high-risk scores. There is a significant correlation between DRG riskscore and clinical features, immune therapy response, drug sensitivity, and genes related to pan-RNA epigenetic modifications. High-risk SARC patients were identified as potential beneficiaries of immune checkpoint inhibitor therapy. We established regulatory axes in SARC, including CALR/hsa-miR-29c-3p/LINC00943, CASP3/hsa-miR-143-3p/LINC00944, and MIR503HG. RT-qPCR data further confirmed the upregulation of prognostic URGs in SARC. Finally, we validated the prognostic model’s excellent predictive performance in predicting outcomes for SARC patients.

**Conclusion:**

We discovered a significant correlation between aberrant expression of URGs and prognosis in SARC patients, identifying a prognostic model related to ubiquitination. This model provides a basis for individualized treatment and immunotherapy decisions for SARC patients.

## 1 Introduction

Sarcomas are rare, heterogeneous malignant tumors originating from mesenchymal tissue, comprising less than 1% of all solid malignancies (1). Current treatments primarily include surgery, (neo)adjuvant chemotherapy, and/or radiotherapy (2). Despite standard therapies, clinical outcomes for metastatic or locally advanced sarcomas remain limited, with conventional treatments leading to non-durable responses and a survival rate of approximately 12 to 18 months (3). Immunotherapy has shown significant success in various solid tumors, providing promising treatment options (4–6). However, most sarcomas are “cold” tumors with minimal immune cell infiltration, potentially leading to poor immunotherapy responses (7). Combined immunotherapy has demonstrated efficacy in certain sarcoma subtypes, including alveolar soft part sarcoma, angiosarcoma, and undifferentiated pleomorphic sarcoma (8–10). Therefore, exploring the mechanisms of immune infiltration in sarcomas and combining immune checkpoint inhibitors (ICIs) with targeted therapies or chemoradiotherapy may offer new strategies for treating sarcomas.

Ubiquitin (Ub) is a small protein composed of 76 amino acids, widely present in most eukaryotic cells. It plays a crucial role in various cellular processes, including cell cycle progression, signal transduction, transcription regulation, receptor down-regulation, and endocytosis (11). Ubiquitination, an essential post-translational modification, involves an ATP-dependent cascade reaction, attaching Ub to substrate proteins (12, 13). While the ubiquitin-proteasome system primarily degrades intracellular target proteins, some ubiquitinated proteins perform non-degradative functions, such as gene transcription, expression, and DNA damage repair. Abnormal ubiquitination can contribute to tumor development and progression (14, 15). Tumor metastasis remains a leading cause of death in sarcomas and other malignancies. The extensive role of ubiquitination in tumor invasion and metastasis warrants further investigation. This study aims to explore the role of abnormal ubiquitination in SARC development, providing new insights for diagnosis and treatment.

In this study, we conducted a comprehensive bioinformatics analysis of URGs in SARC, examining their expression and prognostic implications, as well as their relationship with the tumor microenvironment. We developed a novel prognostic signature based on five URGs and constructed a ceRNA regulatory network. Our findings aim to enhance treatment strategies and prognostic evaluations for SARC.

## 2 Materials and methods

### 2.1 Data sources and preprocessing

We obtained transcriptomic data of SARC samples from the GSE17674 dataset in the GEO database (https://www.ncbi.nlm.nih.gov/geo/) (16). Differential expression analysis between 44 SARC samples and 18 normal samples was performed using the “limma” package in R (v3.40.2) (17) (**Supplementary Table 1**). Adjusted P-values were used to correct for false positives, with DEGs selected based on |fold change (FC)| = 1.5 and adj.P-value < 0.05. Volcano plots were generated using the “ggplot2” R package (v4.2.1). Principal Component Analysis (PCA) plots were created with the “ggord” package to assess group differences. Using the GeneCards database (https://www.genecards.org/) (18), we searched for ubiquitination-related genes (URGs) with the keyword “Ubiquitination Related Genes.” In the GeneCards database, each gene is assigned a relevance score to evaluate its correlation with various elements, including chemical substances and diseases. A higher score indicates a stronger statistical correlation between the gene and the relevant components. Genes with a relevance score greater than 5 were included in the analysis (19), resulting in a total of 1,055 ubiquitination-related genes identified. (**Supplementary Table 2**). Additionally, 1794 immune-related genes (IRGs) were obtained from the Immunology Database and Analysis Portal (ImmPort) (https://www.immport.org/home) (20) (**Supplementary Table 3**). We used the “VennDiagram” package in R(v4.2.1) (21) to visualize the overlap of DEGs, IRGs, and URGs, identifying differentially expressed URGs (DEURGs). RNAseq data and clinical information for SARC, including 260 SARC samples and 2 normal samples, were integrated from the Cancer Genome Atlas (TCGA, https://portal.gdc.cancer.gov/) (22). Protein-protein interaction (PPI) network analysis of URGs was conducted using STRING (https://string-db.org/, version 11.5) (23). Data, normalized as Transcripts Per Million (TPM), were analyzed using the “ggplot2” R package (v4.2.1), and gene expression data were extracted to construct a data matrix, analyzed via Wilcoxon test.

### 2.2 Subtype establishment

We extracted immune-related DEURGs from the TCGA expression matrix and performed consensus clustering analysis using the R package ConsensusClusterPlus (v1.54.0) (24). The maximum number of clusters was set to 6, with 100 repetitions and 80% resampling of the total samples (clusterAlg = “hc”, innerLinkage = ‘ward.D2’). Clustering heatmaps were generated using the R package pheatmap (v1.0.12), retaining genes with variance above 0.1. Based on URG expression profiles, TCGA cases were classified into two clusters (K=2), labeled as C1 and C2.

### 2.3 Identification and enrichment analysis of differentially expressed genes

Differential expression analysis between C1 and C2 subtypes was conducted using the Limma package (v3.40.2) in R. DEGs were identified with the criteria of “Adjusted P < 0.05 and log2(fold change) > 1.5 or log2(fold change) < -1.5.” Heatmaps were drawn using the “heatmap” package in R (v4.2.1). Functional enrichment of DEGs was performed using the “clusterProfiler” R package (v3.18.0) (25), analyzing Gene Ontology (GO) and Kyoto Encyclopedia of Genes and Genomes (KEGG) pathways. GO enrichment analysis encompassed Biological Process (BP), Cellular Component (CC), and Molecular Function (MF). Gene set enrichment analysis (GSEA) (http://software.broadinstitute.org/gsea/index.jsp) (26) identified potential biological pathways, with significant pathways defined by p.adjust < 0.05 and FDR < 0.25.

### 2.4 Genetic alteration analysis

Gene Set Cancer Analysis (GSCA) (http://bioinfo.life.hust.edu.cn/GSCA/#/) integrated expression, mutation, drug sensitivity, and clinical data from four public sources for 33 cancer types. Somatic mutations in SARC patients were downloaded and visualized using the maftools package in R, covering seven mutation types: Missense_Mutation, Multi_Hit, Frame_Shift_Del. We analyzed the correlation between URG mRNA expression and CNV/methylation, and their impact on survival outcomes, including Disease Free Interval (DFI), Disease Specific Survival (DSS), Overall Survival (OS), and Progression Free Survival (PFS). The cBioPortal (http://www.cbioportal.org/index) (27) was used to visualize genetic alterations and their effects on survival outcomes.

### 2.5 Construction of the URG prognostic model

Kaplan-Meier curves, P-values, and hazard ratios (HR) with 95% Confidence Intervals (CI) were obtained via logrank test and univariate Cox regression. Significant URGs affecting SARC prognosis (CALR, CASP3, BCL10, PSMD7, PSMD10) were identified. Expression and diagnostic efficiency of URGs were validated in SARC using GSE21122, GSE17674, and GSE36001 datasets. A prognostic model was developed using LASSO-Cox regression analysis, with risk scores calculated as: Riskscore=∑iCoefficient(mRNAi)×Expression (mRNAi). Patients were categorized into low- and high-risk subtypes based on average risk scores. Kaplan-Meier analysis and timeROC analysis assessed survival differences and model accuracy. Subsequently, we validated the accuracy of the URG risk model using the aforementioned formula by randomly dividing SARC patients from the TCGA-SARC dataset into two validation sets: Validation Set 1 (n = 130) and Validation Set 2 (n = 130). The external validation cohort included GSE21257, GSE17674, GSE16091, and clinical SARC tissue samples (28, 29), further confirming the previous results. Univariate and multivariate Cox regression analyses, visualized with forest plots, showed each variable’s impact (including P-value, HR, and 95% CI). A nomogram predicting 1, 3, and 5-year survival rates was established using the “rms” R package (v4.2.1).

### 2.6 Immune cell infiltration and immunotherapy response analysis

The “ggstatsplot” R package (v4.2.1) analyzed the abundance of immune cells infiltrating tumors for five prognostic URGs, including B cells, CD4+ T cells, CD8+ T cells, Neutrophils, Macrophages, and Myeloid dendritic cells. Reliable immune scoring was performed using the”immunedeconv” R package (v4.0.3) (30) and six algorithms: TIMER (31), xCell (32), MCP-counter (33), CIBERSORT (34), EPIC (35), and quantTIseq (36). The ssGSEA method in the “GSVA” R package (v4.2.1) (37) quantified the infiltration levels of 24 common immune cell types. Differences in immune cell infiltration levels between high- and low-expression groups were analyzed using the Wilcoxon rank sum test, and correlations between URG expression and immune cell infiltration were assessed with Spearman analysis. The ESTIMATE algorithm estimated immune and stromal cell abundance and tumor purity. Expression of immune checkpoint genes and HLA members was analyzed and visualized using the “ggplot2” and “pheatmap” R packages (v4.2.1). Spearman correlation analysis, visualized with the “circlize” package (v0.4.1), examined relationships between URG expression, immune checkpoints, and HLA members. TIDE algorithm predicted potential responses to immune checkpoint blockade (38), validated using GSE91061, IMvigor210 datasets, and clinical samples.

### 2.7 TMB, MSI, mRNAsi, and drug sensitivity analysis

Clinical application of our signature was explored by comparing TMB and MSI scores between high- and low-risk groups using the Wilcoxon rank sum test, visualized with “ggplot2” R package (v4.2.1). Optimal TMB cutoff values were calculated using the “surv_cutpoint” function in the “survminer” R package (v4.2.1), dividing patients into low- and high-TMB/MSI groups. Kaplan-Meier survival curves compared OS between these groups. Drug response prediction utilized GDSC (https://www.cancerrxgene.org/) and CTRP (https://portals.broadinstitute.org/ctrp/) databases. Chemotherapy drug IC50 values were estimated with the “pRRophetic” R package (v4.0.3) (39), integrating drug sensitivity and gene expression profiles from GDSC and CTRP databases.

### 2.8 Single cell analysis

The Tumor Immune Single Cell Center (TISCH, http://tisch.comp-genomics.org/) (40) was utilized to investigate the expression of prognostic URGs in different single-cell subpopulations within the tumor microenvironment (TME) of SARC patients. TISCH focuses on single-cell RNA sequencing (scRNA-seq) data specific to the TME, offering detailed cell type annotations across various cancer types. In this study, the dataset SARC_GSE119352_aPD1aCTLA4 from TISCH was analyzed, providing t-SNE plots and heatmaps to illustrate the impact of URGs on the SARC tumor microenvironment. The dataset encompasses three primary cell types: immune cells, stromal cells, and malignant cells.

### 2.9 Pan-RNA epitranscriptomic gene selection

The expression differences of m6A, m5C, m1A, and m7G related genes between high and low-risk groups in 260 SARC samples were analyzed using the Wilcoxon test and visualized with the “ggplot2” package in R (v4.2.1). The correlation between prognostic URGs and these RNA modifications was also examined. The expression matrices included:

- m6A genes: RBM15B, VIRMA, HNRNPA2B1, YTHDF3, IGF2BP3, HNRNPC, RBM15, RBMX, METTL14, YTHDC2, METTL3, ZC3H13, WTAP, YTHDF1, YTHDC1, FTO, YTHDF2, ALKBH5.
- m5C genes: DNMT1, DNMT3A, DNMT3B, MBD1, MBD2, MBD4, MECP2, TDG, UHRF1, UHRF2, UNG, ZBTB33, ZBTB38, ZBTB4, TET1, TET2, TET3.
- m1A genes: TRMT10C, TRMT61B, TRMT6, TRMT61A, ALKBH3, ALKBH1, YTHDC1, YTHDF1, YTHDF2, YTHDF3.
- m7G genes: AGO2, CYFIP1, DCP2, DCPS, EIF4A1, EIF4E, EIF4E3, EIF4G3, GEMIN5, IFIT5, LARP1, LSM1, NCBP1, NCBP2, NCBP3, NSUN2, NUDT11, SNUPN, WDR4.

### 2.10 Prediction of potential microRNA and long non-coding RNA target genes

Candidate miRNAs were identified and their potential targets were predicted using ENCORI (http://starbase.sysu.edu.cn/) (41), RNAInter (http://www.rnainter.org/) (42), miRDB (http://mirdb.org) (43), and RNA22 (https://cm.jefferson.edu/rna22/interactive) (44). These selected miRNAs were termed potential miRNA target genes. Additionally, potential lncRNAs were predicted using ENCORI and miRNet (http://www.mirnet.ca/) (45). A regulatory network comprising mRNA-miRNA and miRNA-lncRNA interactions was constructed using Cytoscape (version 3.7.1; http://www.cytoscape.org/) (46). The correlations and prognostic values of these candidate miRNAs and lncRNAs in SARC were further validated through ENCORI, TCGA-SARC, and the Kaplan-Meier plotter database.

### 2.11 Human specimens

The study included tissue samples from 32 pairs of sarcoma and corresponding normal tissues, specifically comprising osteosarcoma (n=6), liposarcoma (n=4), leiomyosarcoma (n=6), and undifferentiated pleomorphic sarcoma (n=16). Additionally, patient follow-up information was provided by the Chaohu Hospital of Anhui Medical University. The tissue specimens were formalin-fixed and pathologically examined for definitive diagnosis. The study was approved by the Ethics Committee of Chaohu Hospital of Anhui Medical University (Approval No: KYXM-202403-014), and informed consent was obtained from all patients. All experiments complied with relevant guidelines and regulations.

### 2.12 RT-qPCR

Total RNA was extracted from cultured cells using high-purity RNA separation kits (Roche Diagnostics, Mannheim, Germany) and DNase I (Roche Diagnostics, Sigma-Aldrich), following the manufacturer’s instructions. RNA was reverse transcribed using the HiScript^®^ II 1st Strand cDNA Synthesis Kit (MR101-01, Vazyme, Nanjing, China). Quantitative RT-PCR was performed using AceTaq^®^ qPCR SYBR Green Master Mix (Q121-03, Vazyme, China). The amplification conditions were: pre-denaturation at 95°C for 30 seconds, denaturation at 95°C for 5 seconds, annealing at 60°C for 30 seconds, for a total of 40 cycles. Primer sequences (Gene Pharma, China) are listed in **Supplementary Table 8**. The mean cycle threshold (Ct) value of each target gene was normalized to the housekeeping gene GAPDH, and results were shown as fold change using the ΔΔCt method.

### 2.13 Cell lines and culture conditions

The cell lines used in this study were 143B, SW982, SW872, osteoblast cell line (hFOB1.19, Punosai, Wuhan, China), synovial fibroblast (HFLS, Jennio Biotech, Guangzhou, China), and human preadipocyte line (HPA-v, Sciencell). All cell lines were cultured in Dulbecco’s Modified Eagle Medium (DMEM; Gibco, Grand Island, NY, United States) supplemented with 10% fetal bovine serum (Gibco, Grand Island, NY, United States), 100 U/ml penicillin, and 100 U/ml streptomycin (Invitrogen, Carlsbad, CA, United States). The hFOB1.19 cell line was cultured at 34°C in an incubator with 5% CO2, while the other cell lines were cultured at 37°C with 5% CO_2_.

## 3 Results

### 3.1 Identification of immune-related differentially expressed URGs in SARC

**Figure 1:**
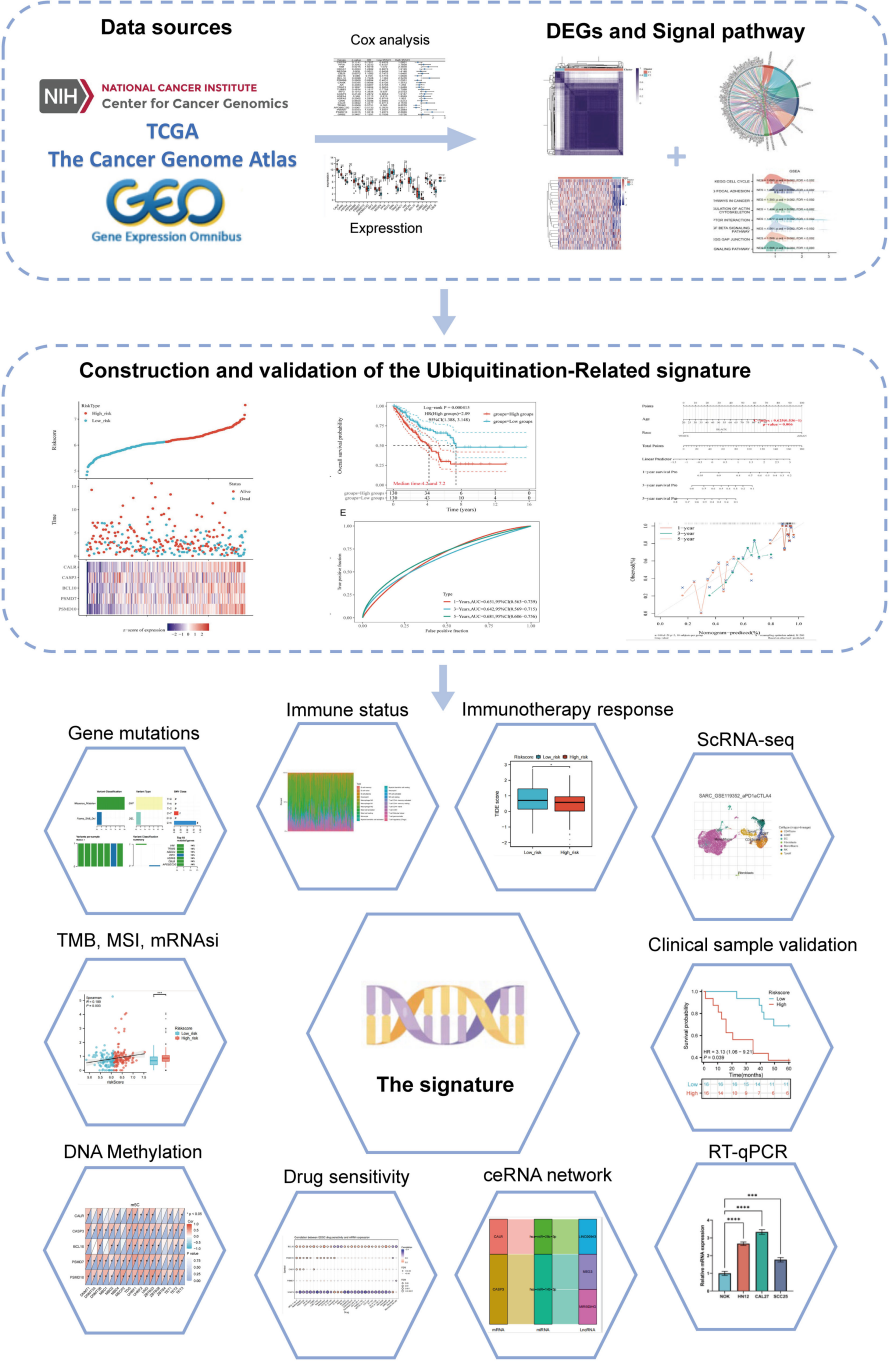
Flowchart of the present study.

**Figure 2:**
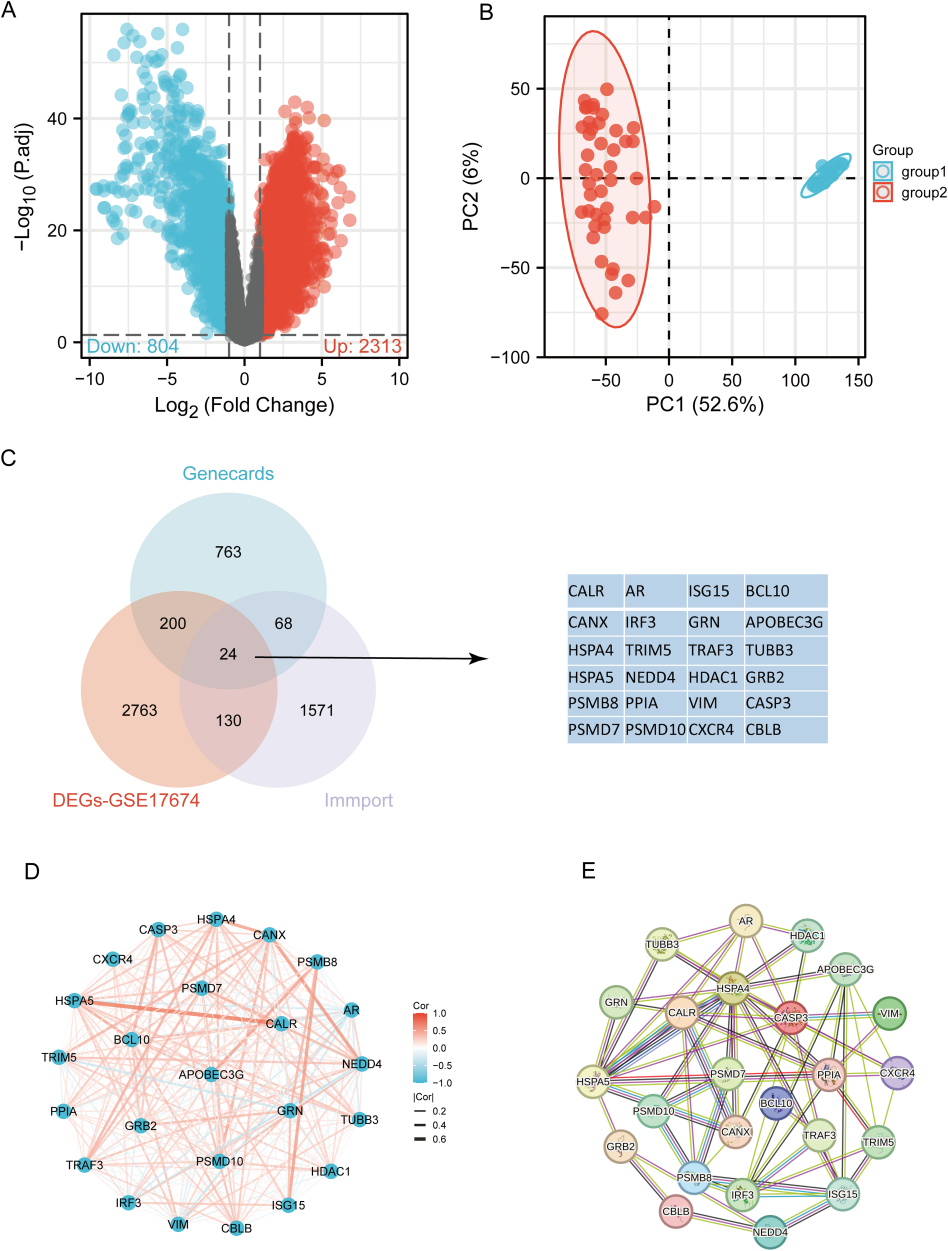
Identification of differentially expressed URGs related to immunity. **(A)** Volcano plot of DEGs between SARC and normal samples in the GSE17674 dataset; **(B)** Principal component analysis of transcriptome profiles of differentially expressed genes between two groups; **(C)** Spearson correlation analysis of 24 URGs expression in SARC; **(D)** Venn diagram showing the overlap between DEGs, URGs, and IRGs; **(E)** Protein-protein interaction (PPI) network of URGs.

The flowchart of the study is illustrated in **Figure 1**. Initially, 3117 differentially expressed genes (DEGs) were identified from the GSE17674 dataset, comprising 2313 upregulated and 804 downregulated genes (**Figure 2A**). Principal component analysis (PCA) indicated distinct transcriptomic profiles between the two groups (**Figure 2B**). From the GeneCards database, 1055 URGs were identified, while the ImmPort database provided 1793 IRGs. A total of 24 overlapping immune-related differentially expressed URGs were selected for further analysis (**Figure 2C**). Additionally, the correlation of expression profiles of these 24 URGs was explored using the TCGA database, revealing a significant positive correlation among most URGs in SARC samples (**Figure 2D**). Protein-protein interaction (PPI) network analysis using STRING demonstrated close interactions among m7G-related proteins, essential for the molecular mechanisms and metabolism of malignancies (**Figure 2E**).

### 3.2 Identification and analysis of clusters of URGs in SARC

**Figure 3:**
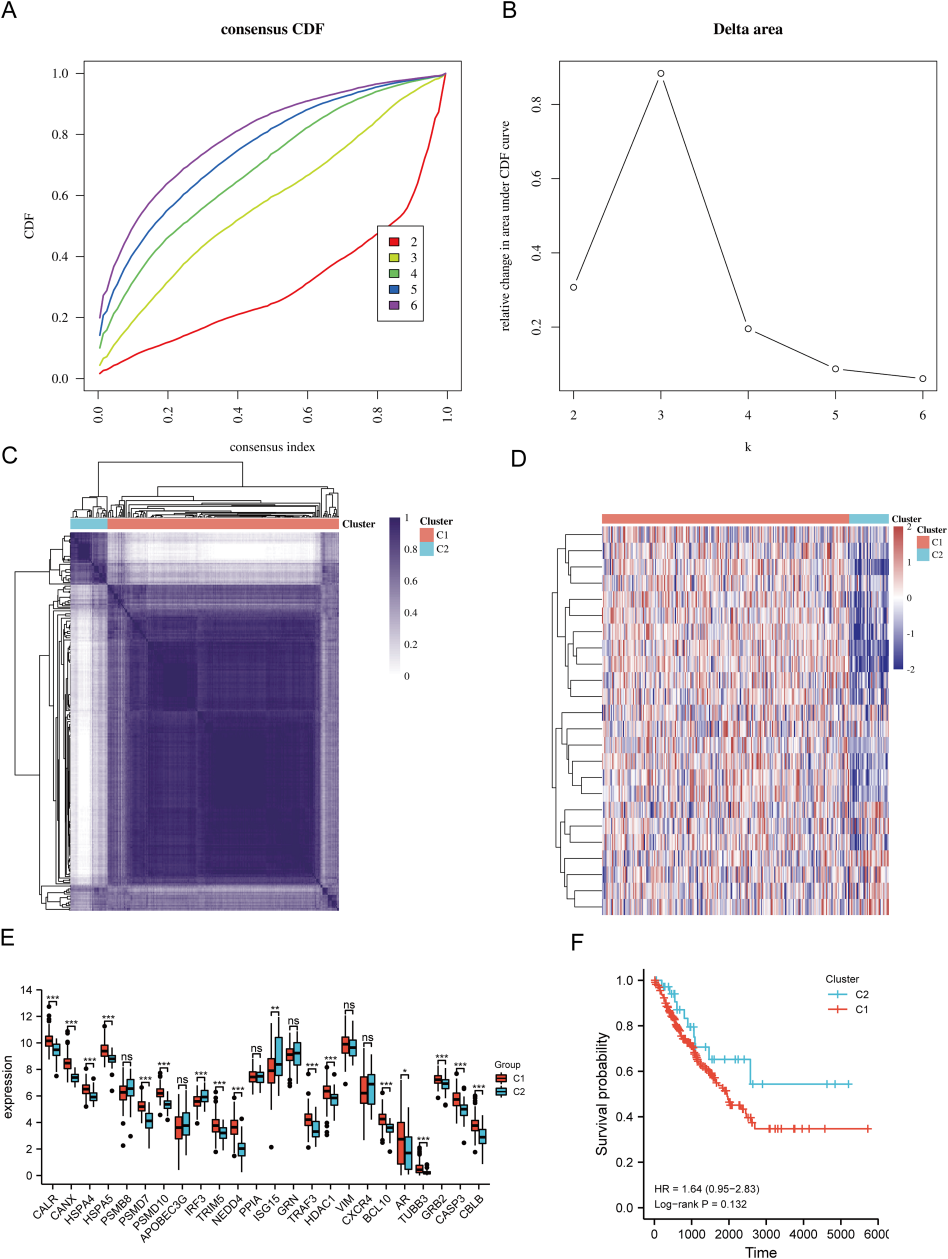
Identification of shared clusters based on the expression similarity of URGs. **(A)** Cumulative distribution function (CDF) (k = 2-6); **(B)** Relative change in area under the CDF curve (k = 2-6); **(C)** Consensus clustering matrix (k = 2); **(D)** Heatmap of URGs expression in different subtypes; **(E)** Expression of 24 URGs between C1 and C2 subgroups; **(F)** Kaplan-Meier survival analysis based on two clusters. *p < 0.05, **p < 0.01, ***p < 0.001. n.s., no significance.

Based on the expression levels of 24 URGs in SARC, consensus clustering was performed on 260 SARC samples from the TCGA database. The optimal number of clusters was determined to be 2 (k=2), classifying SARC patients into two clusters: C1 (N=224) and C2 (N=36) (**Figures 3A–D**). Expression differences of the 24 URGs between the two subgroups were validated using the TCGA dataset. Compared to the C2 group, the C1 group showed upregulation of CALR, CANX, HSPA4, HSPA5, PSMD7, PSMD10, TRIM5, NEDD4, TRAF3, HDAC1, BCL10, AR, TUBB3, GRB2, CASP3, and CBLB, while IRF3 and ISG15 were downregulated (**Figure 3E**). Kaplan-Meier survival analysis indicated worse overall survival (OS) for C1 patients compared to C2, although the difference was not statistically significant (**Figure 3F**).

### 3.3 Differential gene expression and functional enrichment in SARC subtypes

**Figure 4:**
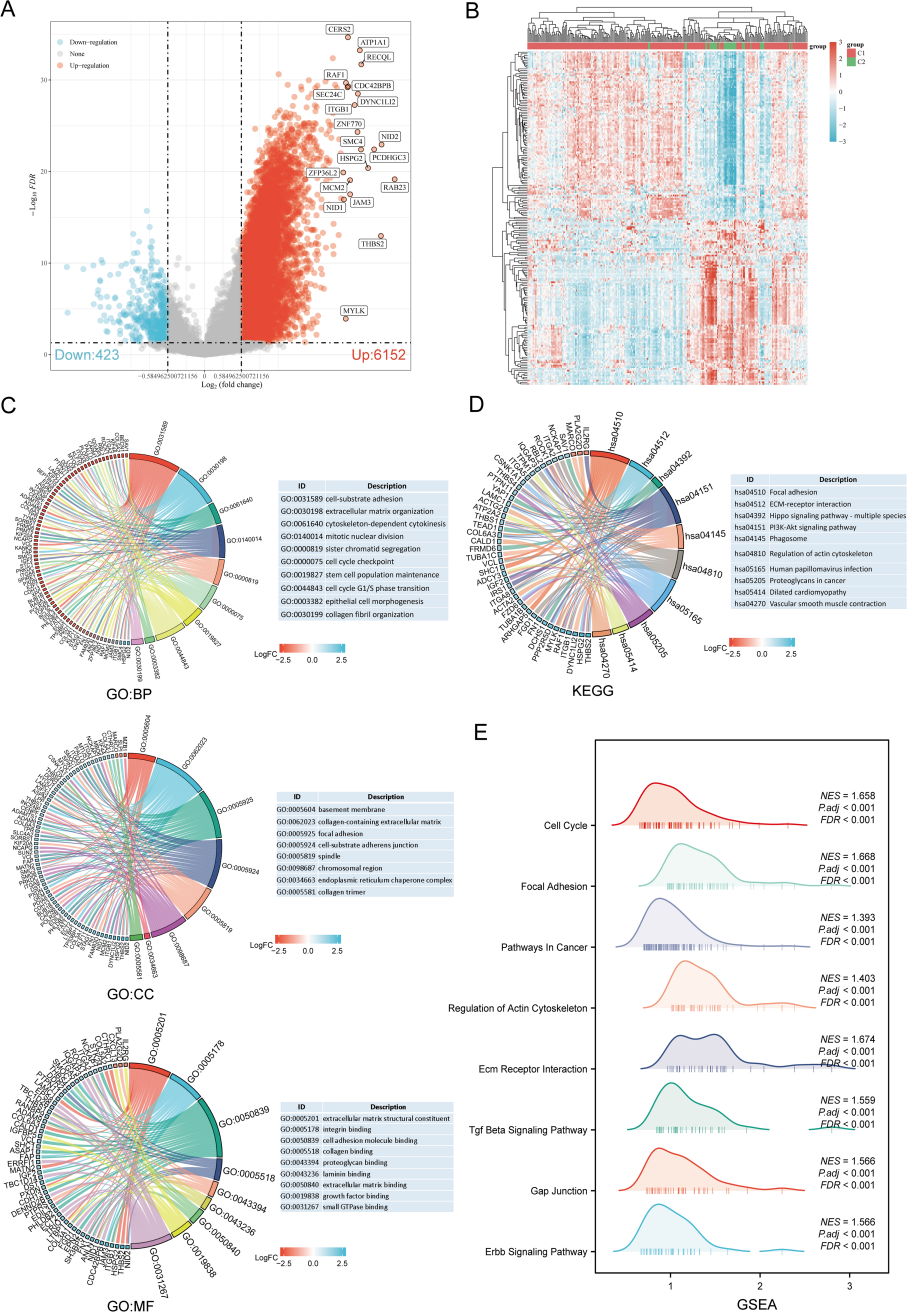
Identification and functional enrichment analysis of DEGs between URGs subtypes. **(A)** Volcano plot of DEGs between C1 and C2 subtypes; **(B)** Heatmap of DEGs between C1 and C2 subtypes; **(C, D)** GO and KEGG enrichment analysis of DEGs; **(E)** GSEA enrichment plot.

The DEGs between the two SARC subtypes (C1 and C2) included 6152 upregulated and 423 downregulated genes. These differences were visualized through a volcano plot (**Figure 4A**) and a heatmap (**Figure 4B**). GO and KEGG enrichment analyses of the identified DEGs were performed (**Supplementary Table 4**). GO enrichment analysis revealed that biological processes (BP) were mainly associated with cell-substrate adhesion, extracellular matrix organization, mitotic nuclear division, stem cell population maintenance, cell cycle G1/S phase transition, epithelial cell morphogenesis, and collagen fibril organization. Cellular components (CC) included collagen-containing extracellular matrix, focal adhesion, spindle, chromosomal region, endoplasmic reticulum chaperone complex, and collagen trimer. Molecular functions (MF) focused on extracellular matrix structural constituent, cell adhesion molecule binding, collagen binding, growth factor binding, and small GTPase binding (**Figure 4C**). KEGG pathway enrichment indicated involvement in pathways such as focal adhesion, ECM-receptor interaction, PI3K-Akt signaling pathway, phagosome, dilated cardiomyopathy, and vascular smooth muscle contraction (**Figure 4D**). GSEA pathway enrichment suggested associations of URG expression with cell cycle, focal adhesion, pathways in cancer, ECM receptor interaction, TGF-beta signaling pathway, GAP junction, ERBB signaling pathway, and regulation of actin cytoskeleton (**Figure 4E**, **Supplementary Table 5**).

### 3.4 Genetic alteration analysis

**Figure 5:**
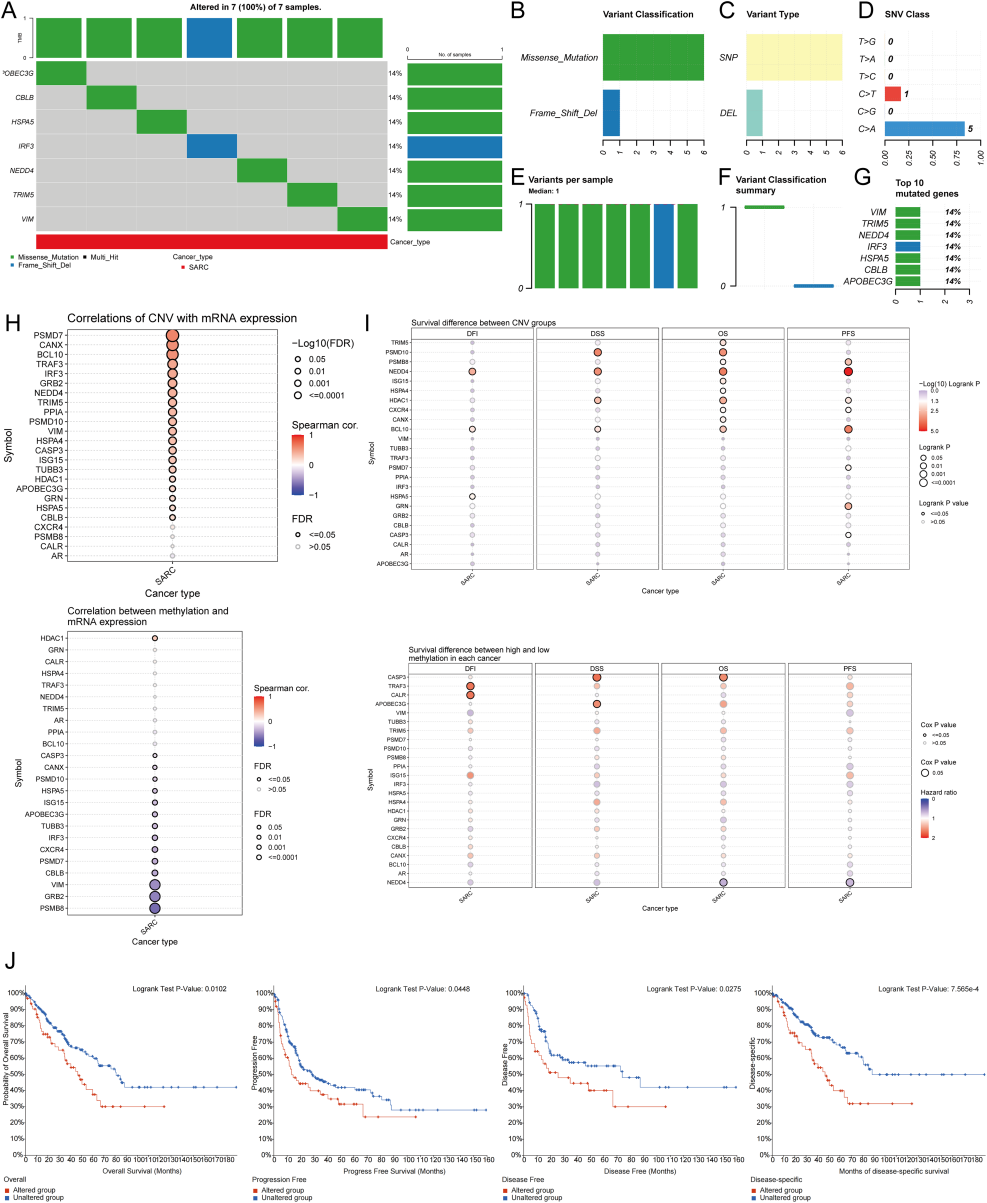
Analysis of genetic alterations associated with URGs in SARC. **(A)** Distribution of mutation types in the first 7 URGs in SARC; **(B-D)** Variant classification, variant type, SNV class; **(E)** Mutation burden per sample; **(F)** Variant classification summary; **(G)** Top 7 mutated genes in SARC; **(H)** Relationship between CNV, methylation, and URGs expression; **(I)** Correlation between URGs CNV, methylation, and survival rates; **(J)** Association between URGs alterations and shorter OS, PFS, DFS, and DSS in SARC patients.

Using the GSCA database, the percentage map of single nucleotide variants (SNVs) was analyzed, revealing a high mutation frequency for FLNA. Oncoplot displayed SNVs for the top 7 genes among URGs, with APOBEC3G, CBLB, HSPA5, IRF3, NEDD4, TRIM5, and VIM each showing a 14% mutation frequency (**Figure 5A**). Missense mutations were the most common type (**Figure 5B**). Single nucleotide polymorphisms (SNPs) were more frequent than deletions (**Figure 5C**), with C>T being the most common SNV type (**Figure 5D**). The median number of mutations per patient was found to be 1 (**Figure 5E**). A box plot showed the number of occurrences for each variant classification (**Figure 5F**). By recalculating the number of mutations and considering multiple hits, the top 7 mutated genes were identified (**Figure 5G**). Correlation analysis of URGs CNV and mRNA expression from the GSCA database indicated a significant positive correlation, while gene methylation levels showed a negative correlation with mRNA expression (**Figure 5H**). **Figure 5I** showed that for some URGs, CNV and methylation levels were significantly associated with poor prognosis in SARC patients. Survival analysis from the cBioPortal database indicated that genetic alterations in URGs were significantly associated with shorter OS (p=0.010), PFS (p=0.0448), DFS (p=0.0275), and DSS (p=7.565e-4) (**Figure 5J**).

### 3.5 Identification of prognostic URGs in SARC

**Figure 6:**
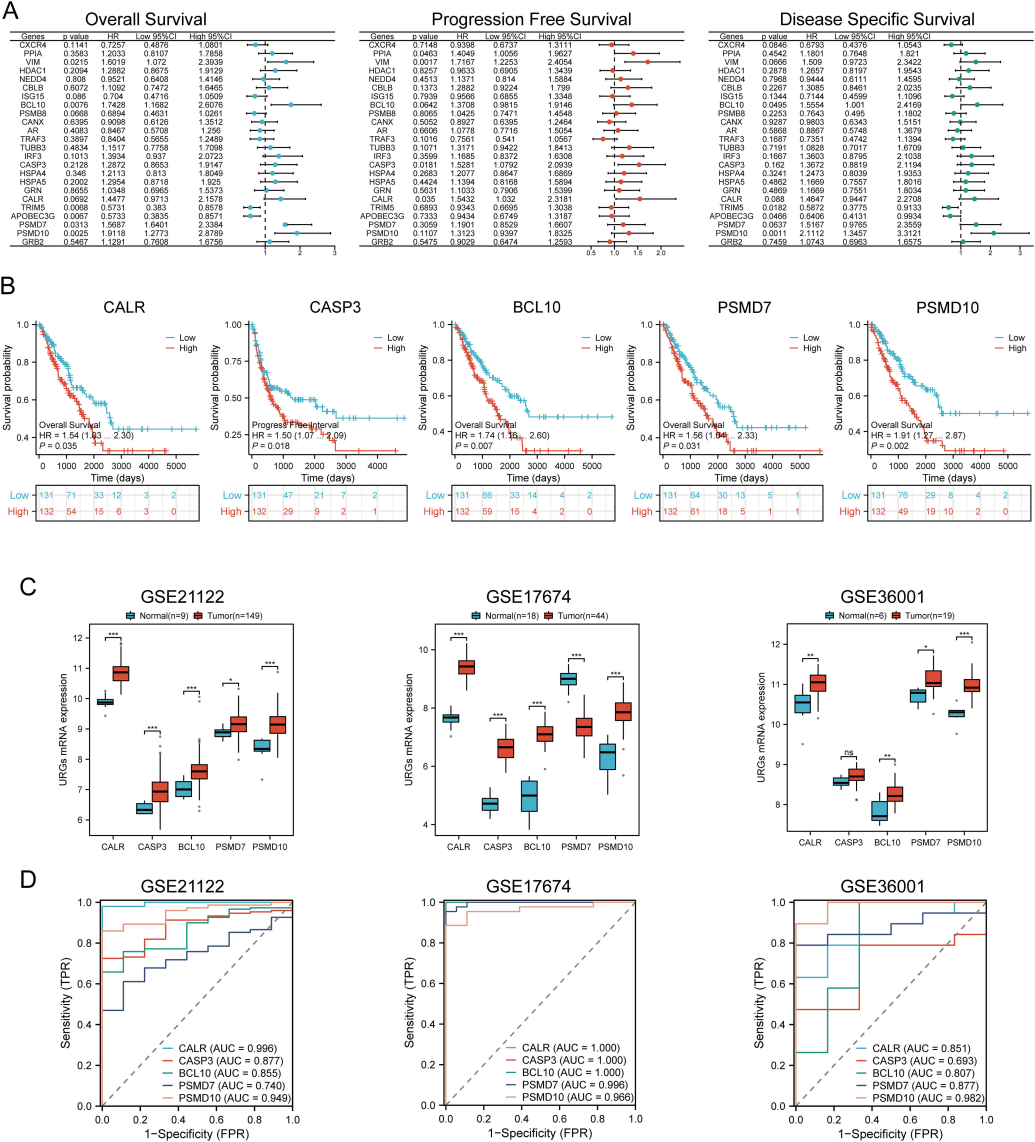
Identification and validation of prognostic URGs in SARC. **(A)** Univariate Cox regression analysis of prognostic URGs for OS, PFS, and DSS; **(B)** Survival curves of high and low expression groups of prognostic URGs; **(C, D)** mRNA expression of prognostic URGs and ROC curves to evaluate their diagnostic ability in SARC datasets. *p < 0.05, **p < 0.01, ***p < 0.001.

Univariate Cox regression analysis was used to predict and screen for prognostic URGs in SARC patients (**Figure 6A**). OS analysis showed that high expression of BCL10, PSMD7, PSMD10, and VIM was associated with lower survival rates. PFS analysis indicated that high expression of CALR, CASP3, PPIA, and VIM predicted poor prognosis in SARC. DSS analysis results demonstrated that high expression of BCL10 and PSMD10 was significantly associated with poor prognosis (**Figure 6B**). Kaplan-Meier survival curves showed that high expression of CALR (P=0.035, HR=1.54 (1.03-2.30)), CASP3 (P=0.018, HR=1.50 (1.07-2.09)), BCL10 (P=0.007, HR=1.703 (1.16-2.60)), PSMD7 (P=0.031, HR=1.56 (1.04-2.33)), and PSMD10 (P=0.002, HR=1.91 (1.27-2.87)) was associated with lower survival rates in SARC patients. The expression levels and diagnostic efficacy of prognostic URGs were further validated using the GEO database. High expression levels of prognostic URGs were significantly upregulated in the GSE21122, GSE17674, and GSE36001 datasets compared to the low-expression group (**Figure 6C**). In the GSE21122 dataset, the AUC values for CALR, CASP3, BCL10, PSMD7, and PSMD10 were 0.996, 0.877, 0.855, 0.740, and 0.949, respectively. In the GSE17674 dataset, the AUC values were 1.000, 1.000, 1.000, 0.996, and 0.966, respectively. In the GSE36001 dataset, the AUC values were 0.851, 0.693, 0.807, 0.877, and 0.982, respectively (**Figure 6D**). The five prognostic URGs (CALR, CASP3, BCL10, PSMD7, and PSMD10) demonstrated consistently good sensitivity and specificity in diagnosing SARC. In conclusion, high expression levels of CALR, CASP3, BCL10, PSMD7, and PSMD10 were identified as potential prognostic biomarkers for SARC.

### 3.6 Prognostic model construction

**Figure 7:**
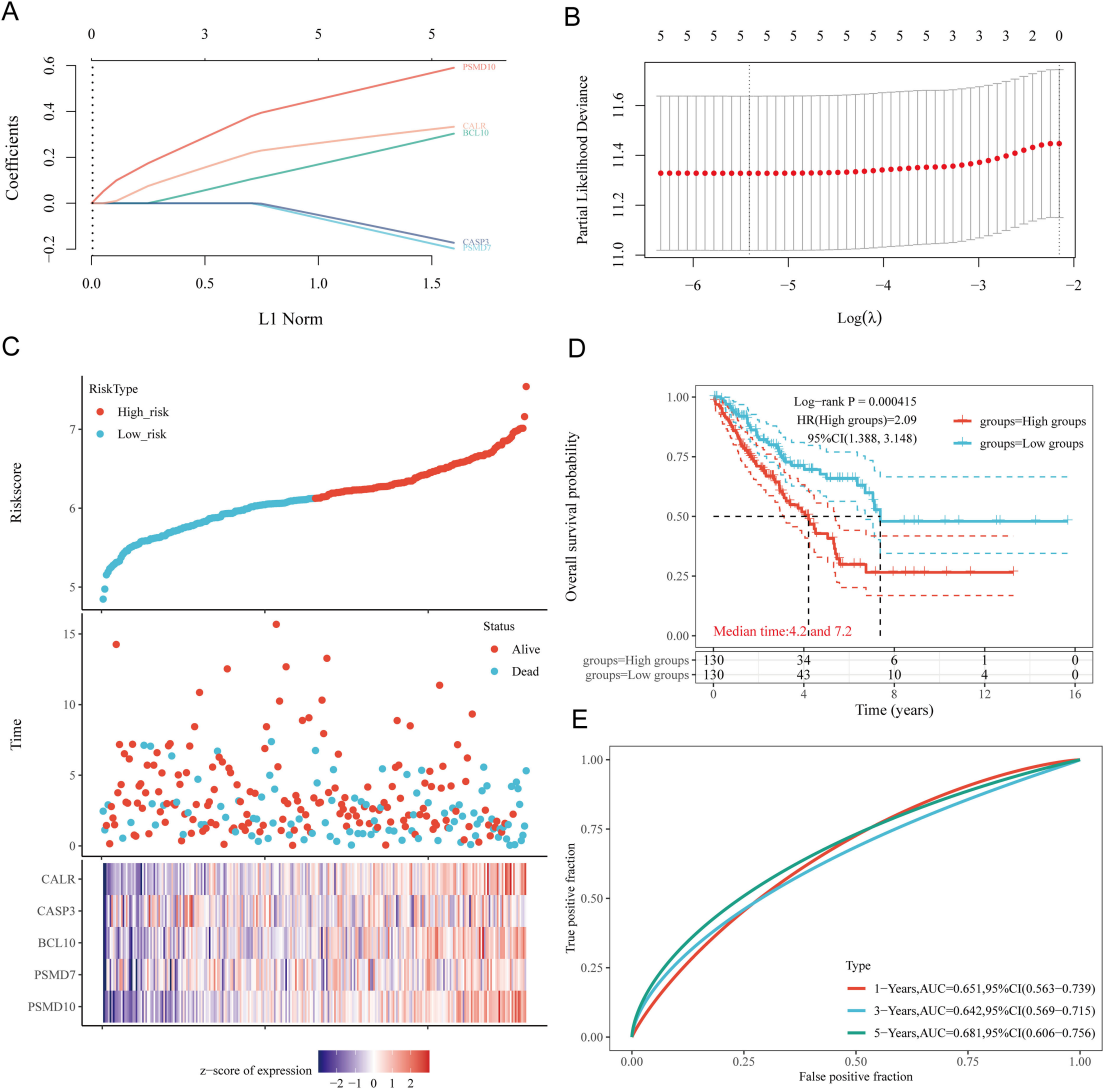
Construction of prognostic model based on URGs in SARC tissues. **(A)** LASSO coefficient curves of 5 URGs; **(B)** Ten-fold cross-validation error rate plot; **(C)** Risk score plot for each SARC patient and distribution of survival time and expression of 5 URGs; **(D)** Overall survival curves for high/low-risk groups of SARC patients; **(E)** Time-dependent ROC curves for OS at 1, 3, and 5 years.

Based on the expression profiles of potential prognostic biomarkers, a prognostic model for sarcoma (SARC) patients was constructed using LASSO Cox regression analysis, selecting Lambda.min to produce the model with higher accuracy (**Figures 7A, B**). The risk score for overall survival (OS) in SARC patients was calculated using the formula: Riskscore=(0.3239)×CALR+(−0.1553)×CASP3+(0.2845)×BCL10+(−0.1792)×PSMD7+(0.5715)×PSMD10. SARC patients were divided into two groups based on their risk scores. Higher risk scores correlated with increased mortality risk and decreased survival time (**Figure 7C**). Kaplan-Meier curves indicated that patients with high-risk scores had significantly lower OS compared to low-risk patients (median survival: 4.2 years vs. 7.2 years, log-rank p = 0.000415, HR = 2.09 (1.388-3.148)) (**Figure 7D**). The 1-year, 3-year, and 5-year ROC curves had AUCs of 0.651, 0.642, and 0.681, respectively (**Figure 7E**). The same analysis was conducted for progression-free survival (PFS) and disease-specific survival (DSS). The PFS risk score formula was: Riskscore=(0.3954)×CALR+(0.0999)×CASP3+(0.144)×BCL10+(−0.2606)×PSMD7+(0.2986)×PSMD10. Higher risk scores were associated with shorter PFS (median survival: 1.3 years vs. 5 years, log-rank p = 0.000354, HR = 1.859 (1.323-2.611)), with 1-year, 3-year, and 5-year ROC AUCs of 0.634, 0.7, and 0.664, respectively (**Supplementary Figure 1A**). For DSS, the risk score formula was:

Riskscore=(0.3292)×CALR+(0.1625)×BCL10+(−0.2173)×PSMD7+(0.6185)×PSMD10. Patients with high risk scores had worse DSS (median survival: 4.2 years, log-rank p = 3.76e-05, HR = 2.632 (1.661-4.169)). The 1-year, 3-year, and 5-year ROC AUCs were 0.668, 0.648, and 0.682, respectively (**Supplementary Figure 1B**). Thus, our URG-related risk characteristics are significantly associated with SARC patient survival.

### 3.7 Internal and external validation of the URGs prognostic signature

To verify the predictive value of the five gene characteristics, we divided the entire TCGA dataset into validation sets. We found that URGs exhibited a high predictive accuracy for overall survival (OS) in both TCGA Validation Set 1 and TCGA Validation Set 2. Risk scores were calculated for each patient in TCGA Validation Sets 1 and 2, and patients were categorized into low-risk and high-risk groups based on the median score. The distribution of risk scores, survival time, and URGs expression for each SARC patient is illustrated in **Supplementary Figures 2A, B**. In the validation sets, patients in the high-risk group had significantly poorer OS compared to those in the low-risk group (**Supplementary Figures 2C, D**). Finally, the one-year, three-year, and five-year survival AUCs for TCGA Validation Set 1 were 0.676, 0.655, and 0.698, respectively (**Supplementary Figure 2E**), while the AUCs for Validation Set 2 were 0.633, 0.614, and 0.661, respectively (**Supplementary Figure 2F**). Then, the GSE21257, GSE17674, and GSE16091 datasets were used as external validation cohorts. Risk scores were calculated for each patient using the same formula, yielding results consistent with the TCGA cohort. Risk score distribution, survival time, and URGs expression for each SARC patient were visualized (**Supplementary Figures 2G–I**). In the validation set, patients in the high-risk group had significantly worse OS than those in the low-risk group (p = 0.040, p = 0.013, and p = 0.041) (**Supplementary Figures 2J–L**). The AUCs for 1-year, 3-year, and 5-year OS were 0.658, 0.654, and 0.679 in the GSE21257 dataset, 0.628, 0.730, and 0.763 in the GSE17674 dataset, and 0.829, 0.587, and 0.424 in the GSE16091 dataset, respectively (**Supplementary Figures 2M–O**). These results confirm the effectiveness of our risk scoring model in predicting SARC patient survival.

### 3.8 Construction of predictive nomogram

**Figure 8:**
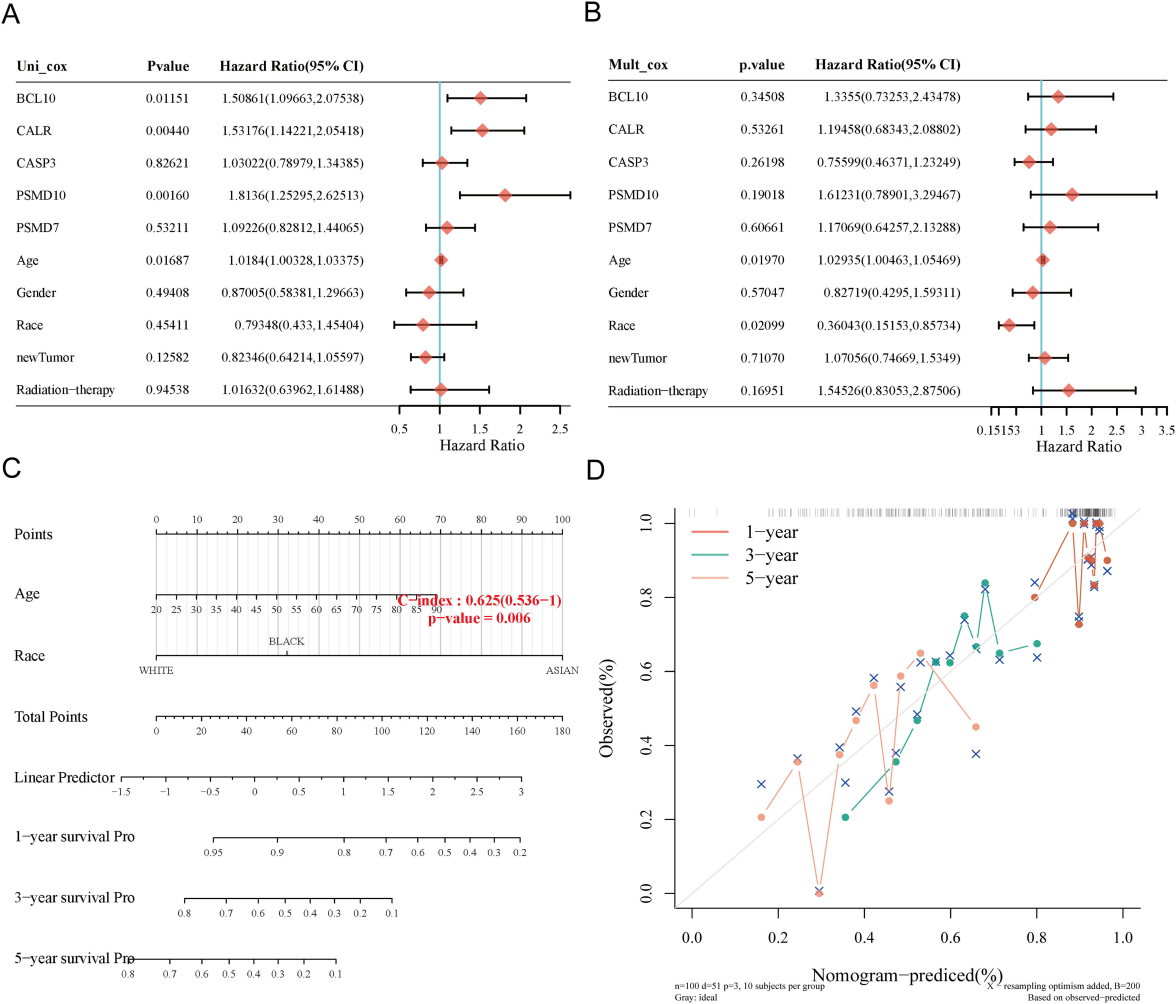
Construction of prognostic nomogram for OS. **(A, B)** Univariate and multivariate Cox regression analysis of clinicopathological features and URGs for OS; **(C)** Nomogram for predicting 1-, 3-, and 5-year OS of SARC patients; **(D)** Calibration curve of OS nomogram model in the discovery group.

We constructed a predictive nomogram to estimate survival probability. Univariate and multivariate regression analyses identified age and race as independent prognostic factors for SARC patients (**Figures 8A, B**). The nomogram’s predictions for 1-year, 3-year, and 5-year OS showed a good fit with the actual outcomes in the entire cohort, with a C-index of 0.625 (0.536-1), p = 0.006 for OS and 0.62 (0.527-1), p = 0.012 for DSS (**Figures 8C, D**, **Supplementary Figure 3**).

### 3.9 Correlation of risk score with clinicopathological characteristics

We explored the correlation between high and low-risk groups and clinicopathological characteristics (**Supplementary Table 6**). Subgroup survival analysis indicated that high-risk significantly impacted survival in patients older than 60 years (P < 0.001), male patients (P < 0.001), white patients (P = 0.002), those with metastasis (P = 0.044), no radiation treatment (P < 0.001), those receiving chemotherapy and hormone therapy (P < 0.001), and those without neoadjuvant treatment (P < 0.001). However, no significant correlation was found in females (P = 0.083), patients aged ≤60 (P = 0.174), Asian+Black patients (P = 0.088), primary+recurrence cases (P = 0.325), and those receiving radiation (P = 0.295) (**Supplementary Figures 6A–L**).

### 3.10 Immune cell infiltration analysis

**Figure 9:**
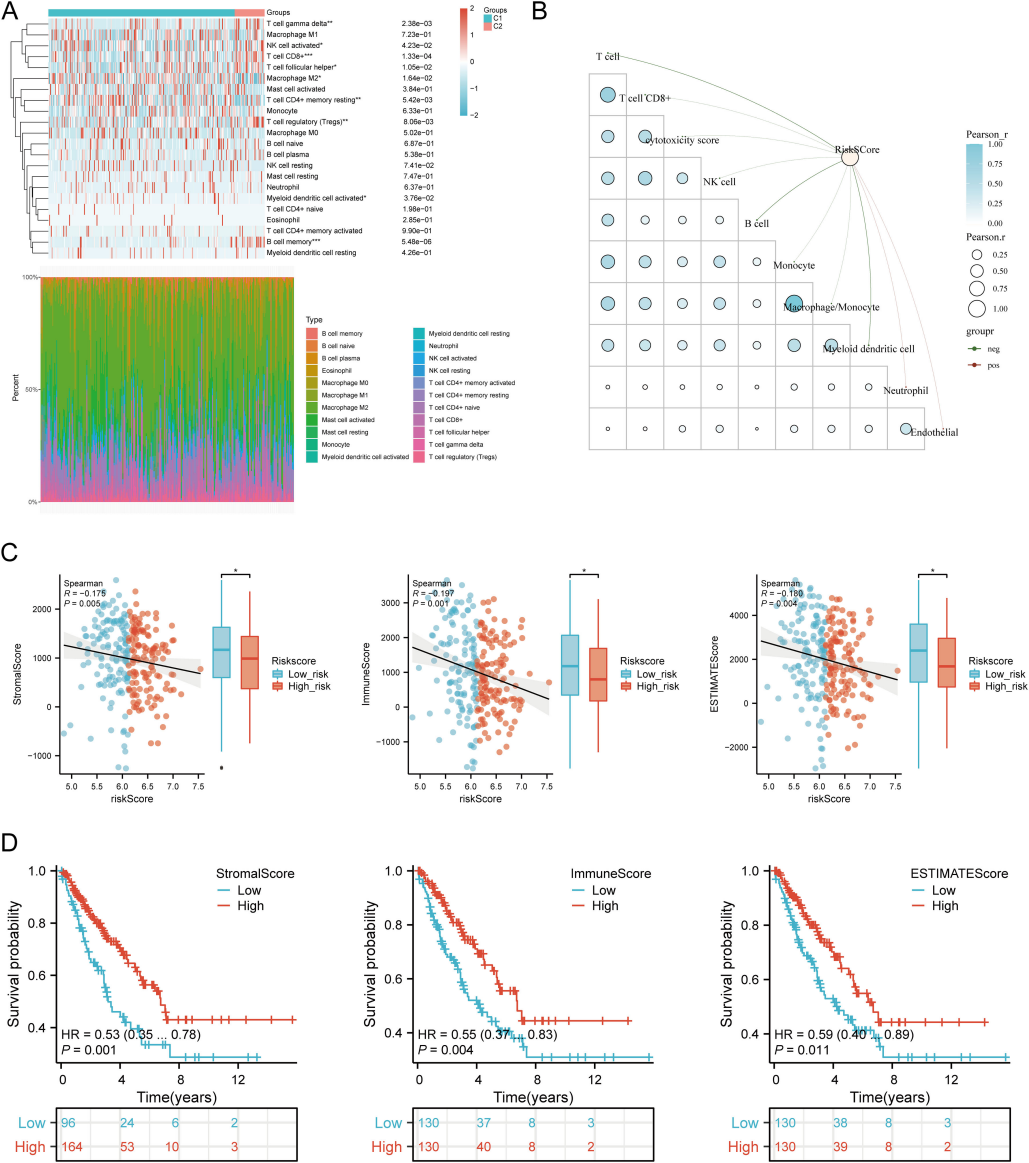
Relationship between URGs expression levels in tumor microenvironment and immune infiltration. **(A)** Comparison of immune scores between C1 and C2 subtypes in TCGA; **(B)** Correlation analysis between Riskscore and Immunocore; **(C)** Correlation between Riskscore and three ESTIMATE; **(D)** Kaplan-Meier curves of high and low ESTIMATE groups in SARC. *p < 0.05, **p < 0.01, ***p < 0.001.

Using six algorithms, we observed differences in immune cell infiltration between SARC subtypes C1 and C2. The CIBERSORT algorithm indicated significant differences in T cell gamma delta, NK cell activated, T cell CD8+, T cell follicular helper, macrophage M2, T cell CD4+ memory resting, T cell regulatory (Tregs), myeloid dendritic cell activated, and B cell memory (**Figure 9A**). Other algorithms (TIMER, xCell, MCP-counter, quanTIseq, EPIC) also showed significant differences in immune infiltration scores between the subtypes (**Supplementary Figures 4A–E**). Correlation analysis between risk scores and immunological scores using the quanTIseq algorithm revealed significant associations with various immune cell populations. Risk scores were negatively correlated with B cells (p = 0.003, cor = -0.186), monocytes (p = 0.015, cor = -0.151), and positively correlated with neutrophils (p < 0.001, cor = 0.269) and uncharacterized cells (p = 0.032, cor = 0.133) (**Figure 9B**, **Supplementary Figures 5A–E**). The ssGSEA method showed significant differences in immune cell infiltration between high and low expression groups of CALR, CASP3, BCL10, PSMD7, and PSMD10 (**Supplementary Figure 7A**). Correlation analysis demonstrated positive correlations between CALR and TFH, macrophages, neutrophils, and Th2 cells, and negative correlations with pDC, NK cells, Tgd, mast cells, cytotoxic cells, and B cells. Similar patterns were observed for the other URGs (**Supplementary Figure 7B**). Further analysis of the correlation between risk scores and three ESTIMATE scores indicated significant negative correlations between risk scores and ImmuneScore (P = 0.001, Cor = -0.197), StromalScore (P = 0.005, Cor = -0.175), and ESTIMATE scores (P = 0.004, Cor = -0.180) (**Figure 9C**). Low ImmuneScore and ESTIMATE scores were associated with poor prognosis (**Figure 9D**), suggesting a correlation between URGs and tumor immune infiltration.

### 3.11 Immunotherapy response analysis

**Figure 10:**
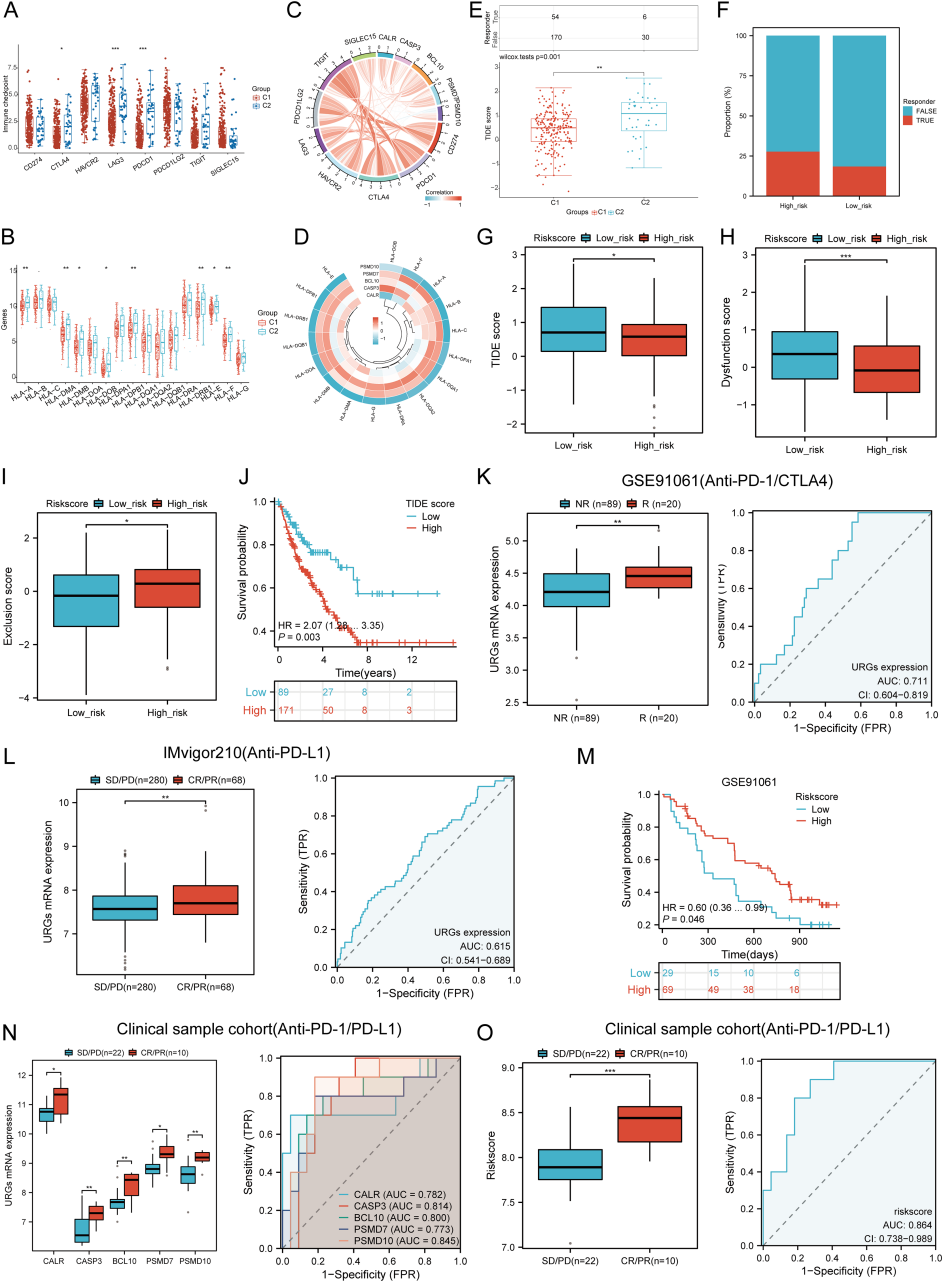
Correlation between expression of prognostic URGs and immunogenicity. **(A)** Differences in immune checkpoint-related genes in SARC subtypes; **(B)** Differences in HLA members between C1 and C2 subtypes; **(C)** Correlation between prognostic URGs in SARC and immune checkpoint-related genes; **(D)** Association between prognostic URGs and HLA members; **(E)** Differential responses to immune checkpoint blocking in TIDE score between C1 and C2 subtypes; **(F)** Prediction of response rates of immunotherapies in patients with URGs high and low riskscore; **(G)** Differential reactions of URGs high and low riskscore groups to immune checkpoint blocking in TIDE score; **(H, I)** Differences of URGs high and low riskscore groups in TIDE Dysfunction score and TIDE Exclusion score; **(J)** Correlation of TIDE scores with prognosis of SARC patients; **(K, L)** Prediction of immune response and ROC analysis of URGs riskscore for prediction of ICI responsiveness in GSE91061, IMvigor210 dataset; **(M)** Kaplan-Meier plots of overall survival for high and low risk patients in GSE91061 dataset; **(N, O)** URGs expression differences and ROC analysis for prediction of ICI responsiveness in clinical tissue samples cohort; Riskscore differences and ROC analysis for prediction of ICI responsiveness in clinical tissue samples cohort. *p < 0.05, **p < 0.01, ***p < 0.001.

We analyzed differences in the expression of eight immune checkpoint-related genes between the two subtypes. Results showed that CTLA4 (P < 0.05), LAG3 (P < 0.001), and PDCD1 (P < 0.001) were significantly higher in the C2 subtype compared to the C1 subtype (**Figure 10A**). Additionally, HLA members (HLA-A, HLA-DMA, HLA-DMB, HLA-DOB, HLA-DPB1, HLA-DRB1, HLA-E, and HLA-F) were more highly expressed in the C2 group compared to the C1 group (**Figure 10B**). Further analysis revealed significant positive correlations between prognostic URGs and several immune checkpoints and HLA members (**Figures 10C, D**). CALR correlated positively with HAVCR2 (P = 0.00841, cor = 0.162) and SIGLEC15 (P = 0.000217, cor = 0.226). CASP3 showed positive correlations with PDCD1LG2 (P = 0.01107, cor = 0.156) and SIGLEC15 (P = 0.00111, cor = 0.200). BCL10 correlated positively with CD274 (P = 0.0424, cor = 0.125), CTLA4 (P = 0.00354, cor = 0.179), HAVCR2 (P = 1.12e-08, cor = 0.343), PDCD1LG2 (P = 0.000496, cor = 0.213), TIGIT (P = 8.59e-05, cor = 0.24), and SIGLEC15 (P = 3.54e-08, cor = 0.332). PSMD7 correlated positively with CD274 (P = 0.0143, cor = 0.151), CTLA4 (P = 0.0129, cor = 0.153), PDCD1LG2 (P = 0.0366, cor = 0.129), and SIGLEC15 (P = 5.14e-06, cor = 0.277). PSMD10 showed negative correlations with PDCD1 (P = 0.00751, cor = -0.164) and HAVCR2 (P = 0.00209, cor = -0.189). Additionally, URGs expression correlated positively with most HLA members in SARC (**Figure 10D**). Using the TIDE database and GSE91061, IMvigor210 datasets, we predicted URGs’ response to immunotherapy. C2 subtype had a better response to immune checkpoint blockade compared to the C1 subtype (**Figure 10E**). High-risk score patients showed higher predicted response rates to immunotherapy than low-risk score patients (**Figure 10F**). The high-risk group responded better to immune checkpoint blocking than the low-risk group (**Figure 10G**). TIDE Dysfunction scores were higher in the low-risk group (**Figure 10H**), and TIDE Exclusion scores were lower in the low-risk group (**Figure 10I**). Kaplan-Meier analysis showed that higher TIDE scores were significantly correlated with poorer overall survival (**Figure 10J**). In the GSE91061 and IMvigor210 datasets, URGs accurately predicted immune therapy response with AUC values of 0.711 and 0.615, respectively (**Figures 10K, L**). High-risk patients in the GSE91061 cohort had worse overall survival compared to low-risk patients (p = 0.046, HR = 0.60 [0.36 - 0.99]) (**Figure 10M**). Validation in 32 advanced sarcoma patients treated with anti-PD-1/PD-L1 showed that high expression of prognostic URGs and high-risk scores correlated with better immunotherapy response. ROC analysis confirmed the predictive efficacy of the risk score (**Figures 10N, O**). These findings suggest that low-risk score groups are more likely to respond to immunotherapy.

### 3.12 TMB, MSI, and mRNAsi analysis

**Figure 11:**
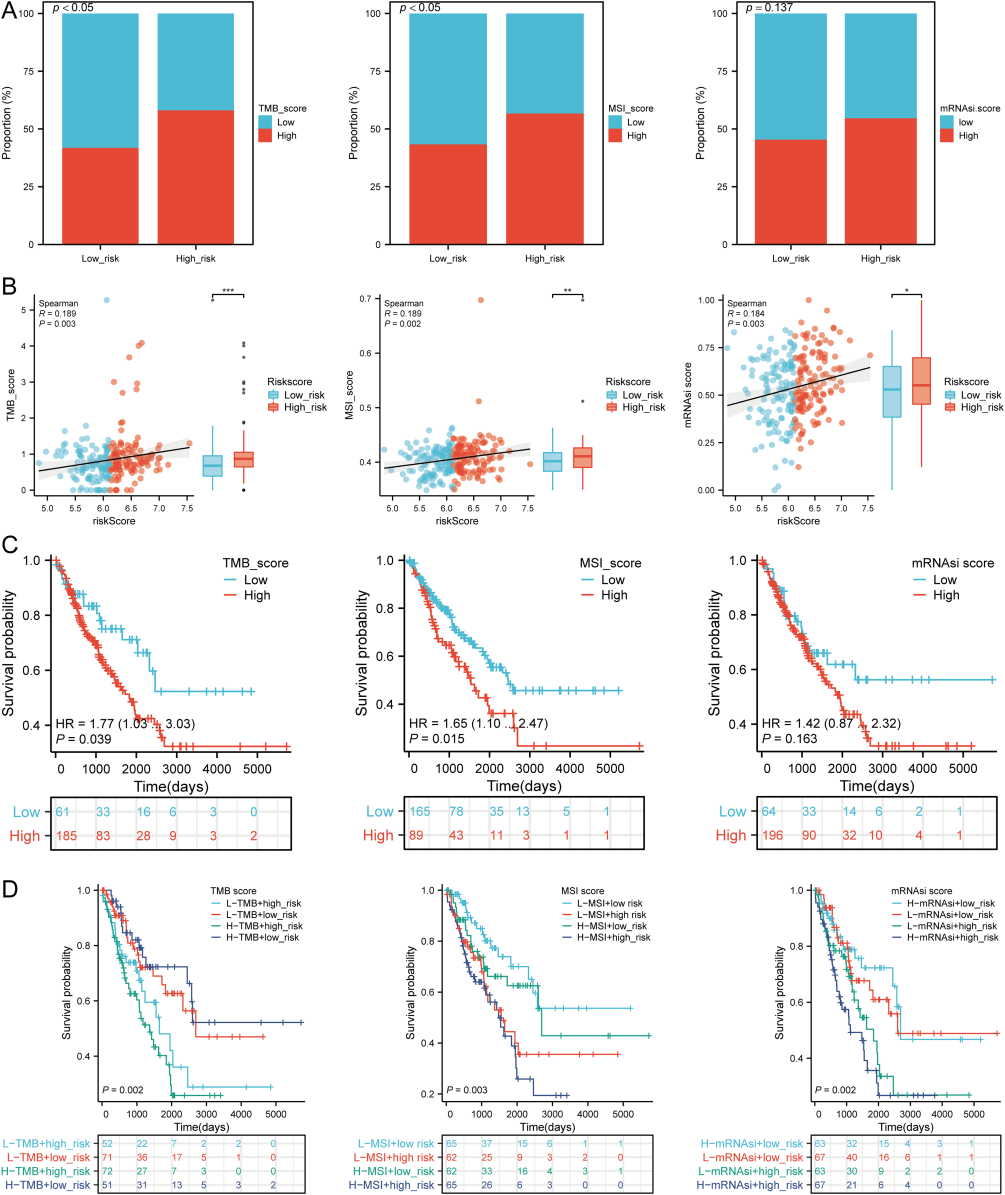
Analysis of TMB, MSI, and mRNAsi. **(A)** Distributions of patients with TMB, MSI, and mRNAsi scores in high and low risk groups; **(B)** Correlation between risk scores model and TMB, MSI, mRNAsi; **(C)** Kaplan-Meier curves of high and low TMB, MSI, mRNAsi groups in SARC; **(D)** Kaplan-Meier curves of four groups classified by risk score and TMB, MSI, mRNAsi in SARC. *p < 0.05, **p < 0.01, ***p < 0.001.

We assessed the correlation between risk scores and TMB, MSI, ESTIMATE, and mRNAsi scores. High-risk patients had higher TMB, MSI, and mRNAsi scores compared to low-risk groups (**Figures 11A, B**). Risk scores positively correlated with TMB (R = 0.189, p = 0.003), MSI (R = 0.189, p = 0.002), and mRNAsi (R = 0.184, p = 0.003) (**Figure 11B**). Survival analysis indicated that high TMB (p = 0.039, HR = 1.77 [1.03 - 3.03]) and MSI scores (p = 0.015, HR = 1.65 [1.10 - 2.47]) were associated with poor OS, but mRNAsi scores were not significantly associated with prognosis (p = 0.163, HR = 1.42 [0.87 - 2.32]) (**Figure 11C**). Combining risk scores with TMB, MSI, and mRNAsi, we observed that patients with low TMB + low risk score had better OS compared to those with high TMB + high risk score (p = 0.002). Similarly, high MSI + high risk patients had worse prognosis compared to low MSI + low risk patients (p = 0.003), and low mRNAsi + low risk patients had better OS compared to high mRNAsi + high risk patients (p < 0.001) (**Figure 11D**). These results suggest that high-risk groups are more likely to have an immune response and respond to immunotherapy.

### 3.13 Drug sensitivity analysis

**Figure 12:**
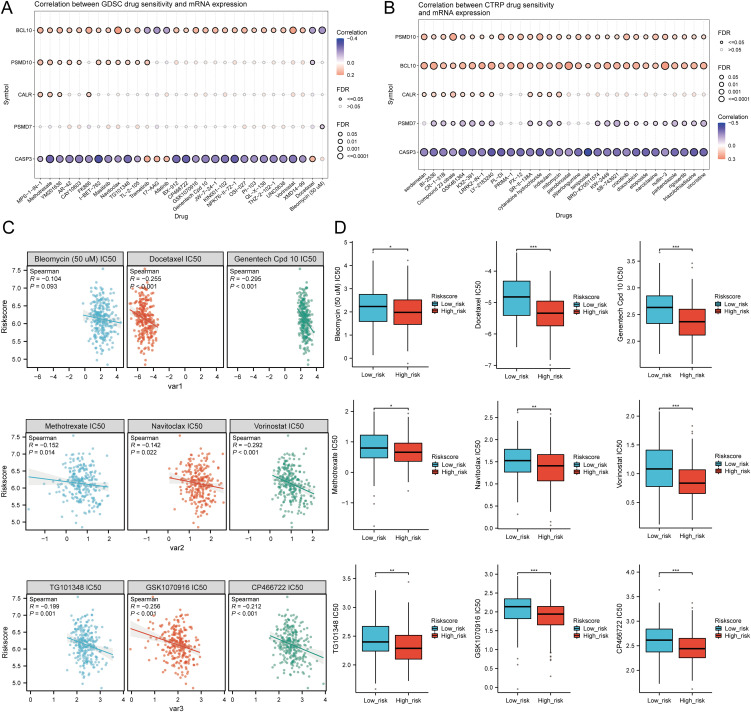
Drug sensitivity analysis. **(A, B)** Predictive antitumor drugs based on the risk scores model in SARC from the GDSC and CTRP datasets; **(C, D)** Correlation analysis of IC50 score and risk scores model, and distribution of IC50 scores in the high and low groups. *p < 0.05, **p < 0.01, ***p < 0.001.

We analyzed drug sensitivity using the GDSC and CTRP databases and found significant correlations between risk scores and drug sensitivity (**Figures 12A, B**). Spearman correlation analysis showed negative correlations between risk scores and the sensitivity to Bleomycin (50 uM), CP466722, Docetaxel, Genentech Cpd 10, GSK1070916, Methotrexate, Navitoclax, TG101348, and Vorinostat (**Figure 12C**). High-risk SARC showed significantly higher sensitivity to these drugs compared to the low-risk group (**Figure 12D**). These drugs may be potential therapeutic options for SARC.

### 3.14 Single-cell RNA data analysis

**Figure 13:**
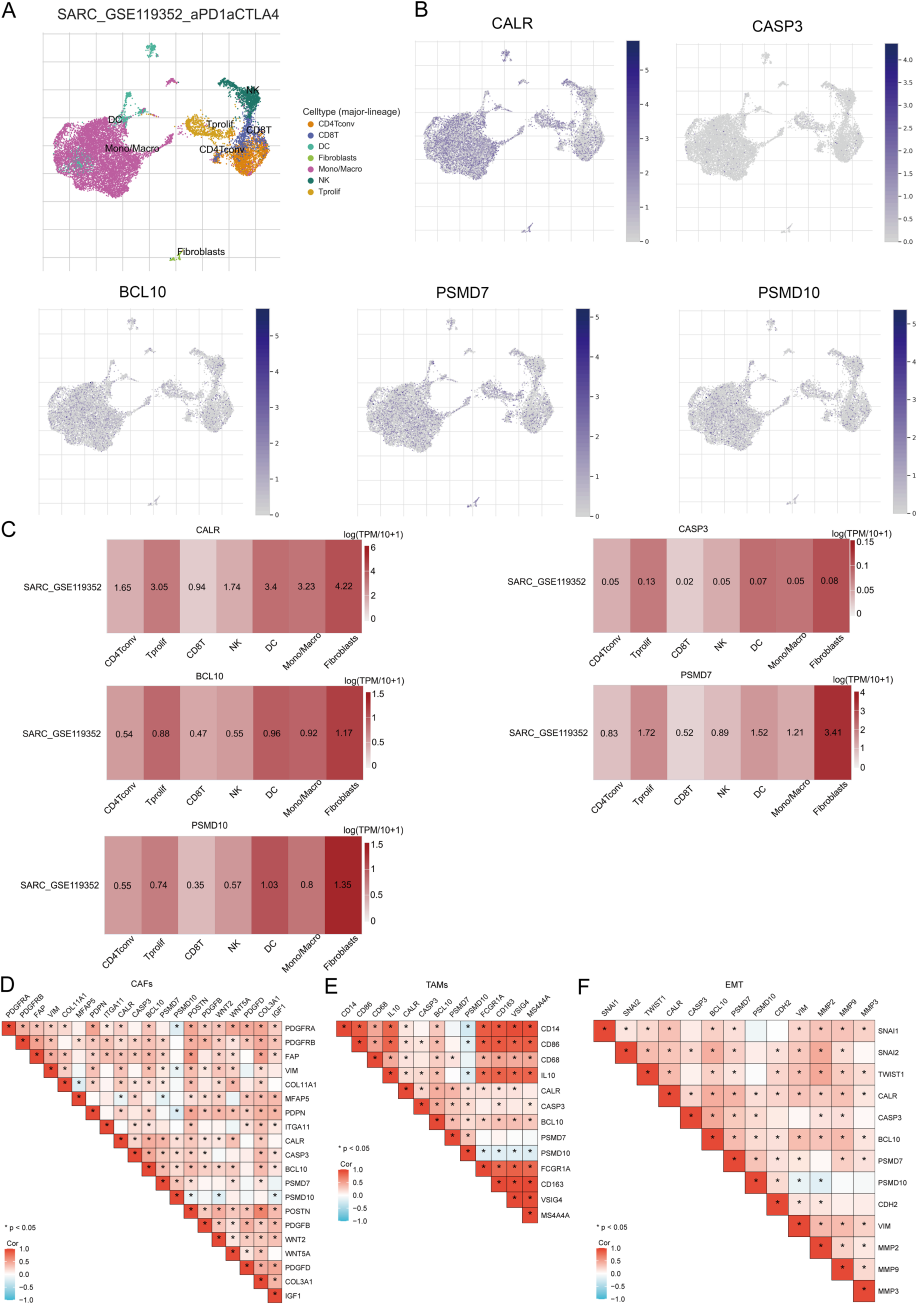
Expression of prognostic URGs in different immune cell types in SARC. **(A)** Clustering of cell types in scRNA-seq data, t-SNE plot showing the expression of different immune cells in SARC tissues; **(B)** Feature plots of CALR, CASP3, BCL10, PSMD7, PSMD10 obtained from scRNA-seq data; **(C)** Heatmap of CALR, CASP3, BCL10, PSMD7, PSMD10 from scRNA-seq data; **(D-F)** Correlation between URGs and CAFs, TAMs, and EMT-related markers.

The tumor microenvironment (TME) consists of extracellular matrix (ECM), cancer-associated fibroblasts (CAFs), myofibroblasts, immune cells, and other factors. Using two GSE datasets (SARC_GSE119352_aPD1aCTLA4), we evaluated the expression of CALR, CASP3, BCL10, PSMD7, and PSMD10 at the single-cell level. The single-cell RNA sequencing analysis annotated various immune cells, including Conventional CD4 T cells (CD4Tconv), proliferating T cells (Tprolif), CD8 T cells (CD8T), Natural killer cells (NK), Dendritic cells (DC), Monocytes/Macrophages (Mono/Macro), and Fibroblasts (**Figures 13A, B**). Prognostic URGs were expressed in all immune cells, with the highest expression in Fibroblasts, followed by Tprolif, Mono/Macro, and DC (**Figure 13C**). We further explored the relationship between URGs and cancer-associated fibroblasts (CAFs), tumor-associated macrophages (TAMs), and their biomarkers, finding extensive correlations (**Figures 13D, E**). Additionally, we analyzed the impact of URGs expression on EMT by examining correlations with EMT-related markers (SNAI1, SNAI2, TWIST1, CDH2, VIM, MMP2, MMP9, MMP3), revealing significant associations (**Figure 13F**). These results suggest that URGs-mediated EMT may be related to fibroblast activation.

### 3.15 Pan-RNA epigenetic modification-related gene expression

We investigated whether URGs expression was associated with pan-RNA epigenetic modifications, analyzing the differential expression of related genes between high- and low-risk groups. Results showed significant differences in m6A, m5C, m1A, and m7G modification genes between the groups, with higher expression in the high-risk group (P < 0.01, **Supplementary Figure 8A**). TCGA dataset analysis revealed significant correlations between prognostic URGs and these modification genes, notably with DNMT3B, EIF4G3, HNRNPA2B1, HNRNPC, IGF2BP3, ZBTB33, YTHDF2, WDR4, UNG, UHRF1, and YTHDF3 (**Supplementary Figure 8B**), all of which were associated with poor SARC prognosis (**Supplementary Figure 8C**). This indicates a close relationship between URGs expression and methylation modifications in SARC.

### 3.16 Prediction and validation of upstream key miRNAs

**Figure 14:**
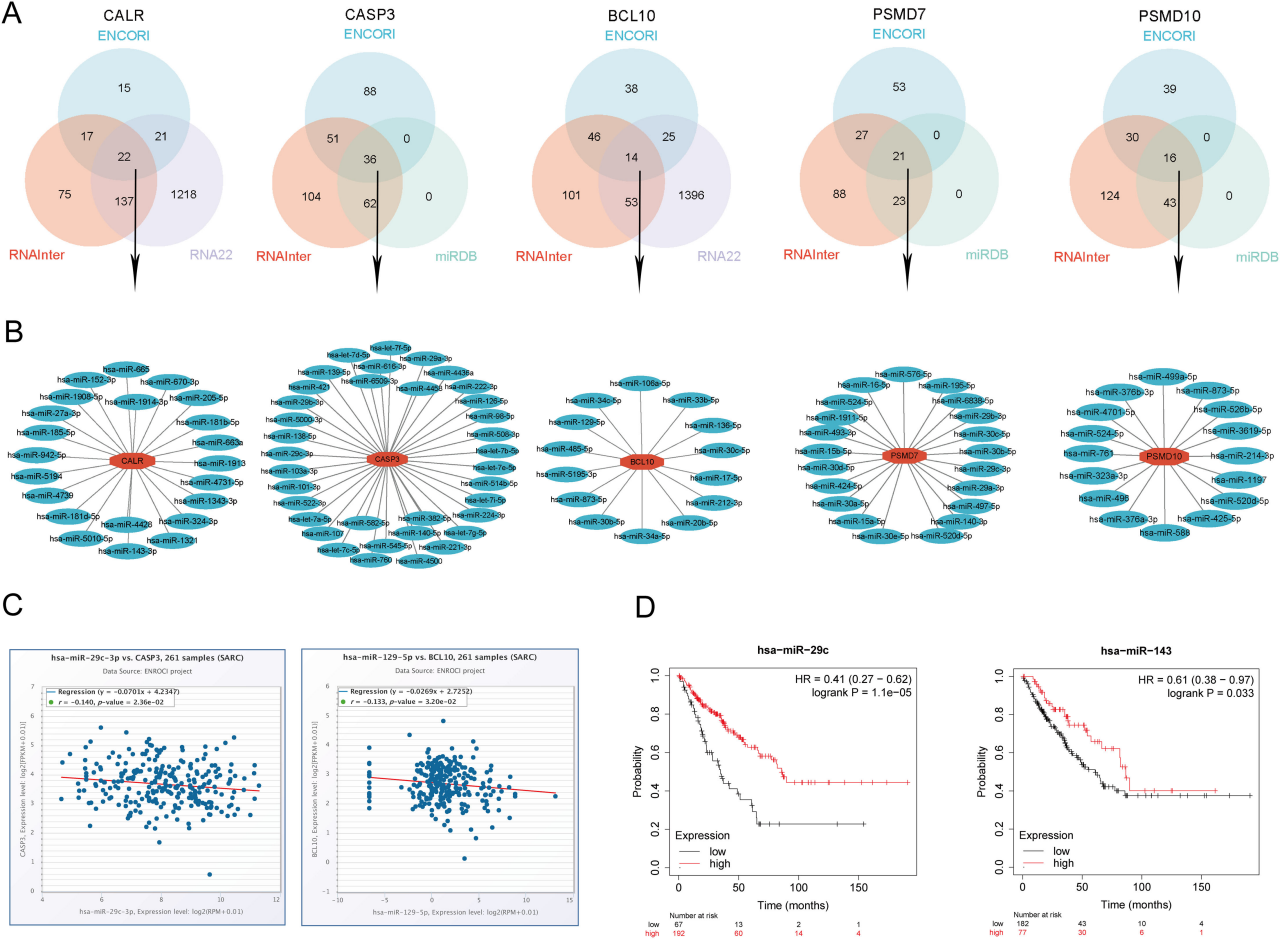
Identification of the most promising miRNAs associated with SARC prognosis. **(A)** Prediction of potential miRNAs for prognostic URGs from ENCORI, RNA22, RNAInter, and miRDB databases; **(B)** Construction of potential miRNAs gene network using Cytoscape software; **(C)** Correlation of potential miRNAs (hsa-miR-29c-3p, hsa-miR-143-3p) with prognostic URGs (CALR, CASP3); **(D)** Prognostic value of miRNAs (hsa-miR-29c-3p, hsa-miR-143-3p).

In this study, we conducted a comprehensive prediction and validation of upstream regulatory miRNAs for CALR, CASP3, BCL10, PSMD7, and PSMD10 by integrating data from various miRNA research databases. Initially, we identified 22 pairs of CALR-miRNA and 14 pairs of BCL10-miRNA by intersecting the ENCORI, RNA22, and RNAInter databases. Subsequently, through the intersection of ENCORI, RNAInter, and miRDB databases, we obtained 36 pairs of CASP3-miRNA, 21 pairs of PSMD7-miRNA, and 16 pairs of PSMD10-miRNA. These data were utilized to construct a potential miRNA-gene network using Cytoscape software (**Figures 14A, B**). Furthermore, based on the classical mechanism of miRNA-mediated gene expression regulation, we expected a negative correlation between mRNA-miRNA interactions. Subsequently, we employed the Pan-cancer subproject of ENCORI database to screen for expression correlations and prognostic implications of these candidate miRNAs in SARC. Our analysis revealed significant negative correlations for 1 pair of miRNA-BCL10, 2 pairs of miRNAs-CALR, 2 pairs of miRNAs-CASP3, and 5 pairs of miRNAs-PSMD10 (**Figure 14C**, **Supplementary Figure 9**). Additionally, we validated the prognostic significance of these potential miRNAs in SARC using the Kaplan-Meier Plotter database. The results demonstrated that low expression of hsa-miR-29c-3p and hsa-miR-143-3p was significantly associated with adverse prognosis (**Figure 14D**). Combining the results of correlation analysis and survival rate analysis, we identified hsa-miR-29c-3p and hsa-miR-143-3p as potential miRNAs in SARC. Collectively, these findings suggest that CALR-hsa-miR-29c-3p and CASP3-hsa-miR-143-3p may serve as crucial pathways mediating SARC development and correlating with patient prognosis.

### 3.17 Prediction and validation of key lncRNAs binding to potential miRNAs

Previous studies have indicated that lncRNAs can bind to miRNAs, thereby modulating the expression of target genes and exerting biological effects. Building upon these findings, we further predicted upstream lncRNA targets of miRNAs to establish a miRNA-lncRNA axis. Through the intersection of ENCORI and miRNet databases, we predicted potential lncRNAs binding to hsa-miR-29c-3p and hsa-miR-143-3p, identifying 52 lncRNAs targeting hsa-miR-29c-3p and 54 lncRNAs targeting hsa-miR-143-3p (**Supplementary Figure 10A**). To enhance visualization, we constructed a miRNA-lncRNA regulatory network using Cytoscape software (**Supplementary Figure 10B**). According to the competitive endogenous RNA (ceRNA) hypothesis, lncRNAs can competitively bind to miRNAs to increase mRNA expression. Therefore, we examined the expression correlation of lncRNAs with hsa-miR-29c-3p and hsa-miR-143-3p using the ENCORI database. Our analysis revealed 1 lncRNA (LINC00943) significantly correlated with hsa-miR-29c-3p and CALR, and 4 lncRNAs (LINC00944, LINC01806, SMIM25, MIR503HG) significantly correlated with hsa-miR-143-3p and CASP3 (**Supplementary Table 7**, **Supplementary Figures 10C, D**). Subsequently, using the TCGA database, we assessed the prognostic value of these lncRNAs in SARC. The results indicated that high expression of LINC00943, LINC00944, and MIR503HG was associated with adverse prognosis (**Supplementary Figure 10E**). Finally, we established a key mRNA-miRNA-lncRNA triple regulatory network associated with SARC prognosis, comprising 2 mRNAs (CALR, CASP3), 2 miRNAs (hsa-miR-29c-3p, hsa-miR-143-3p), and 3 lncRNAs (LINC00943, LINC00944, MIR503HG) (**Supplementary Figure 10F**).

### 3.18 Cellular experiments and clinical sample validation

**Figure 15:**
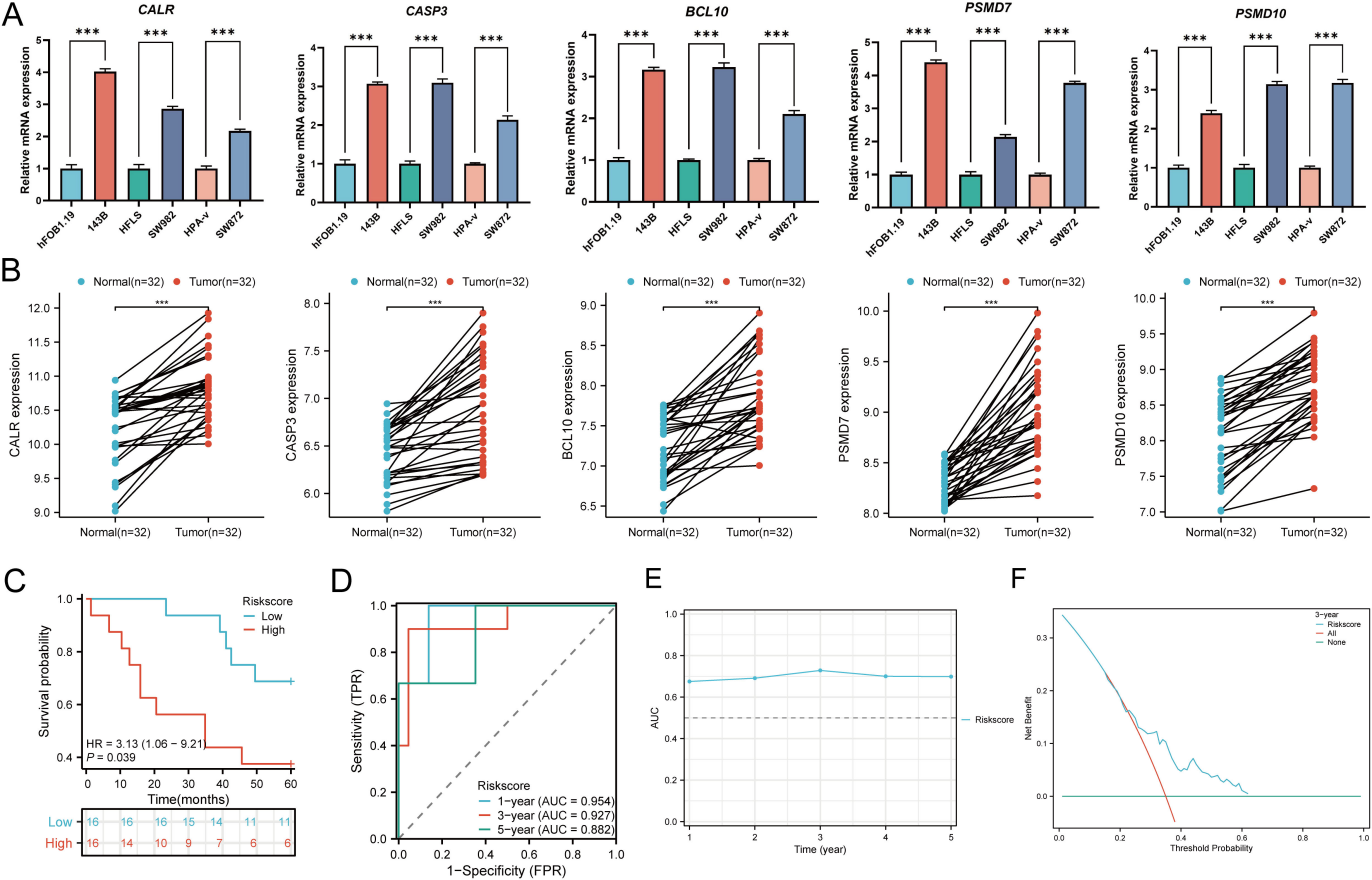
Cellular experiments and clinical sample validation. **(A)** Differential expression of five prognostic URGs in sarcoma cell lines and normal cell lines; **(B)** Relative expression of prognostic URGs in normal tissues and SARC tissues; **(C)** Overall survival curve of SARC patients in high/low-risk groups; **(D)** Time-dependent ROC curve for 1-, 3-, and 5-year OS for URGs; **(E)** Time-dependent AUC curve showing URGs prognostic signature performance in the clinical sample group; **(F)** DCA curves for URGs prognostic signature in the clinical sample group. *p < 0.05, **p < 0.01, ***p < 0.001.

In cellular experiments, we observed significantly upregulated mRNA expression of CALR, CASP3, BCL10, PSMD7, and PSMD10 in sarcoma cell lines compared to their corresponding normal cell lines (**Figure 15A**). Furthermore, we employed RT-qPCR to assess the expression of the five prognostic URGs in 32 SARC tissues and normal tissues. Consistent with cellular expression levels, we found significant upregulation of CALR, CASP3, BCL10, PSMD7, and PSMD10 in SARC tissues compared to normal tissues (**Figure 15B**). Lastly, based on the prognostic model constructed from the TCGA-SARC dataset, we validated the predictive performance of this model using clinical tissue samples. Patients were stratified into high-risk and low-risk groups based on calculated risk scores. Survival analysis revealed that patients with higher risk scores had shorter overall survival compared to those with lower risk scores (p=0.039, HR= 3.13 (1.06 - 9.21), (**Figure 15C**), consistent with results from TCGA and GEO databases. The area under the ROC curve (AUC) for 1, 3, and 5 years were 0.954, 0.927, and 0.882, respectively (**Figure 15D**). Furthermore, time-dependent AUC curve analysis demonstrated the performance of the URGs prognostic signature in predicting OS in the clinical sample validation cohort (**Figure 15E**). Finally, decision curve analysis (DCA) showed the clinical utility of the URGs prognostic signature in predicting survival rates (**Figure 15F**). These findings collectively confirm the predictive performance and clinical utility of the prognostic model constructed above, indicating its enhanced credibility and effectiveness in predicting prognosis for SARC patients.

## 4 Discussion

Ubiquitination and deubiquitination, as a reversible process, can modulate the lifespan and function of substrate proteins, thus widely participating in various physiological processes such as cell proliferation, apoptosis, autophagy, endocytosis, DNA damage repair, and immune responses, among others, with aberrant dysregulation implicated in tumorigenesis (47–49). Many key enzymes in the ubiquitination process are considered potential therapeutic targets for cancer treatment (50, 51). However, the mechanistic role of ubiquitination regulatory factors in SARC (sarcoma) remains less elucidated. To gain deeper insights into the expression patterns, prognostic value, and potential mechanisms of Ubiquitination-Related Genes (URGs) in SARC, we conducted bioinformatics analysis on public sequencing data and performed experimental validation using RT-qPCR to guide future research on URGs in SARC.

In this study, we first screened differentially expressed URGs related to immunity, categorized into two subtypes, C1 and C2, and obtained Differentially Expressed Genes (DEGs) between these subtypes, further exploring the potential oncogenic mechanisms of URGs. We found that URGs participate in signaling pathways such as cell cycle, focal adhesion, ECM-receptor interaction, PI3K-Akt signaling pathway, and TGF BETA signaling pathway. These pathways have been reported to be closely associated with tumor invasion and metastasis. Increasing evidence suggests that the cell cycle regulation pathway integrates with other hallmarks of cancer, including metabolic rewiring and immune evasion (52). β-Carboline dimers inhibit tumor proliferation by suppressing cell cycle arrest via insertion into cell cycle protein-A2 (53). Focal adhesion turnover plays a crucial role in cell migration (54), and Focal adhesion kinase mediates pro-migration and anti-apoptotic properties, serving as a potential therapeutic target in sarcomas (55). The ECM-receptor interaction pathway is the most enriched signaling pathway, playing crucial roles in tumor shedding, adhesion, degradation, motility, and proliferation, involved in colorectal cancer development and metastasis (56). The PI3K/Akt pathway is considered one of the most important oncogenic pathways in human cancers, with ZIP10 driving osteosarcoma proliferation and chemoresistance through activation of the PI3K/AKT pathway mediated by ITGA10 and highly correlated with clinical prognosis (57, 58). Transforming growth factor-beta (TGFβ) is a multifunctional cytokine that regulates crucial cellular processes, including epithelial-mesenchymal transition (EMT) (59). GDF15 may promote osteosarcoma cell metastasis by regulating the TGF-β signaling pathway, associated with poor prognosis and serving as a potential prognostic and lung metastasis-predictive biomarker (60). CALR is a multifunctional protein that plays critical roles in calcium homeostasis and protein folding within the endoplasmic reticulum. Additionally, CALR is crucial in immune cell death and macrophage migration. Its exposure on the surface of dying tumor cells acts as an “eat-me” signal for macrophages, promoting phagocytosis and immune clearance of cancer cells. Dysregulation of CALR can impair this immune recognition, leading to immune evasion by tumor cells (61). Moreover, CALR’s role in modulating immune responses, such as macrophage recruitment and polarization, further emphasizes its involvement in immune-related cancer processes (62). CALR dysregulation is also linked to its interaction with TGF-β signaling pathways, influencing immune suppression and tumor progression (63). CASP3 is one of the most critical proteins in executing apoptosis. It not only mediates cell death but also contributes to tumor cell invasion and metastasis by regulating matrix metalloproteinases (MMPs), which degrade the extracellular matrix, promoting cancer cell migration (64). CASP3 is also implicated in modulating the tumor microenvironment through interactions with immune cells, making it a potential target for enhancing immune therapies (65). BCL10 is involved in activating the NF-κB signaling pathway, a key regulator of cell proliferation and survival. In cancer, BCL10 dysregulation leads to enhanced tumor growth and immune evasion by promoting pro-survival signals. For example, BCL10 has been implicated in the activation of AKT signaling, which supports cancer cell survival and migration, particularly in liver and gastric cancers (66). Additionally, BCL10 has been shown to contribute to the inflammatory tumor microenvironment by modulating immune responses, making it a candidate for targeted therapies (67). PSMD7 and PSMD10 are subunits of the 26S proteasome complex, essential for degrading ubiquitinated proteins. Dysregulation of these proteins leads to impaired degradation of proteins involved in cell cycle regulation and DNA repair, thereby promoting cancer progression. PSMD7 is particularly important in maintaining chemoresistance, as seen in its role in stabilizing RAD23B in gastric cancer, contributing to resistance against cisplatin treatment (68). PSMD10, on the other hand, plays a role in EMT and cell migration, further promoting cancer cell invasion and metastasis, especially in breast cancer (69).

The prognostic model of URGs we constructed is closely associated with the clinical outcomes of SARC, identifying five prognostic ubiquitination-related genes (CALR, CASP3, BCL10, PSMD7, PSMD10). Some studies have shown that these hallmark genes are closely related to tumors. CALR is a widely expressed and highly conserved protein involved in tumorigenesis, proliferation, migration, adhesion, and mediates phagocytosis, signal transduction, and immune cell death (70, 71). Additionally, mediating macrophage migration and polarization can significantly predict immunotherapy response (72). CASP3 is an important component of cell apoptosis, and its aberrant function may play a key role in cancer pathogenesis (73), involved in regulating the migration, invasion, and metastasis of colon cancer cells (74). One of the functions of BCL10 protein is to activate NF-κB, thereby promoting cell growth, and its aberrant expression is associated with the occurrence and development of lymphoma (75). Studies have shown that high expression of BCL10 promotes liver cell proliferation and migration via activation of the AKT signaling pathway, reducing cell apoptosis, and is associated with poor prognosis of the tumor microenvironment (76). Previous research has reported that the overexpression of the deubiquitinase PSMD7 promotes gastric cancer cell proliferation, invasion, and cisplatin resistance by stabilizing RAD23B (77). PSMD7 is associated with cell cycle regulation and disease progression in breast cancer, indicating poor prognosis (78). PSMD10 is an important regulatory factor in EMT and cell migration (79). In recent studies, dysregulation of PSMD10 promotes tumor progression and significantly affects tumor cell proliferation and migration (80, 81).

Additionally, we constructed a predictive model using LASSO Cox regression for the five selected genes. Kaplan-Meier curves showed that patients with high-risk scores in the model had poorer prognosis compared to those with low-risk scores. ROC curves for 1-year, 3-year, and 5-year survival probabilities also revealed the good specificity and sensitivity of this prognostic model. According to univariate and multivariate Cox analysis, the model was confirmed as an independent prognostic factor for SARC. Furthermore, we developed a signature-based predictive nomogram to forecast clinical outcomes of SARC patients.

Targeting the tumor microenvironment (TME) is a hot topic in cancer therapy, with many studies reporting the role of ubiquitination in tumor immunology (82, 83). Increasing evidence suggests that immune cell infiltration plays a crucial role in the development and metastasis of SARC (84, 85). Therefore, we evaluated the potential of URGs to reflect TME and the prognostic value of different types of immune cells. In this study, immune-related prognostic URGs were positively correlated with the abundance of certain immune cells, including B.cells.memory, NK.cells.activated, Monocytes, Macrophages, and Mast.cells.resting, which were associated with improved clinical prognosis in SARC. Thus, the mechanisms by which URGs and immune cell phenotypes influence the prognosis of SARC patients may require further evidence and discussion. Tumor-associated macrophages (TAMs) typically resemble M2 macrophages and suppress anti-tumor immunity through various mechanisms, including inhibiting T-cell responses (86). TAM abundance in the TME is often associated with poor prognosis in various solid tumors (87, 88). Accumulating preclinical and clinical evidence suggests that targeting TAMs can significantly enhance the efficacy of conventional and immunotherapies, making them important targets for cancer treatment (89). Additionally, cancer-associated fibroblasts (CAFs) are a key stromal cell type in the TME, composed of heterogeneous and plastic populations that promote tumor growth and metastasis (90). CAFs have emerged as therapeutic targets to improve anticancer treatments (91). Epithelial-mesenchymal transition (EMT) is equally critical in the metastatic cascade (92). CAFs promote tumor EMT through interaction with cancer cells, playing a crucial role in tumor metastasis and dissemination (93, 94). In our study, we found that prognostic URGs were closely correlated with TAMs, CAFs-related markers, and significantly positively correlated with EMT-related markers. Therefore, URGs may affect invasion, metastasis, and chemotherapy resistance of SARC patients by altering the expression of TAMs, CAFs, and other immune cells in the TME.

In recent years, immunotherapy has rapidly developed, with immune checkpoint inhibitors (ICIs) as representatives widely used in the treatment of various cancers. In some SARC patients, antibodies targeting PD-1 and CTLA-4 have shown significant efficacy in clinical applications (9, 95). In this study, we explored the correlation between URG expression and immune checkpoint genes and HLA members. HLA loss leads to weakened antigen presentation ability, which may promote immune escape (96). Our study found that CTLA4, LAG3, and PDCD1 were highly expressed in the C2 group, and some HLA members were significantly higher in the C2 group than in the C1 group, consistent with the infiltration of antigen-presenting cells observed in this study. Moreover, we found that patients with high scores in the C2 group had higher TIDE scores, indicating that patients with high scores in the C2 group might benefit from ICI treatment. Overall, these results strongly suggest that URGs influence immune cell infiltration and are associated with the efficacy of SARC immunotherapy. Therefore, URGs could serve as potential targets for SARC immunotherapy. However, further exploration and analysis of predictive biomarkers in clinical patients are needed to improve the clinical outcomes of SARC.

Some sarcomas respond to immune checkpoint inhibitors, but predictive biomarkers are not well understood. Tumor mutational burden (TMB) and microsatellite instability (MSI) in the tumor microenvironment are associated with anti-tumor immunity and can predict the efficacy of tumor immunotherapy. Given that we have demonstrated the correlation between URGs and the prognosis and immune cell infiltration of SARC patients, we assessed the association between URGs and TMB and MSI. In our study, we found that TMB and MSI scores were significantly higher in the high-risk group than in the low-risk group, and patients with high TMB and MSI scores had shorter overall survival in SARC, suggesting that high TMB and MSI scores were unfavorable for the overall survival of SARC patients. Additionally, prognostic URGs were positively or negatively correlated with various chemotherapy and targeted drugs. However, further experiments are needed to verify these results. These findings provide new potential therapeutic targets for the treatment of SARC.

Disruption of DNA methylation regulatory mechanisms is highly associated with tumorigenesis and is becoming a novel biomarker for tumors (97, 98). Currently, there is a lack of exploration of the relationship between URG methylation and the prognosis of SARC patients, and the utility of DNA methylation analysis as a predictive biomarker for immunotherapy response in sarcomas is also unclear, which is one of the purposes of this study. We confirmed that URG methylation status was negatively correlated in SARC. Studies have shown that m6A, m5C, m1A, and m7G modifications are reversible epigenetic RNA processes that play important roles in the development of malignant tumors (99). We further explored the correlation between URGs and genes related to pan-RNA epigenetic modifications. URGs were positively correlated with the expression of most pan-RNA epigenetic modification regulatory genes and were associated with poor prognosis in SARC patients. In conclusion, URG methylation status may be a promising predictive factor for OS in SARC patients. However, more research is needed to confirm this result.

In this study, we made another significant finding by exploring the CALR/hsa-miR-29c-3p/LINC00943, CASP3/hsa-miR-143-3p/MEG3, and MIR503HG regulatory axes, which may contribute to the invasion and metastasis of SARC. It has been reported that the downregulation of hsa-miR-29c-3p is associated with the progression of squamous cell carcinoma of the throat, closely correlated with clinical pathological parameters and poor prognosis (100). Lu et al. (101) demonstrated that circCSNK1G3 can induce the expression of HOXA3 through the sponge effect of has-miR-143-3p, thereby promoting the occurrence and metastasis of lung adenocarcinoma. The upregulation of LINC00943 regulates the proliferation of gastric cancer cells and sensitivity to chemotherapy, and is also associated with poor prognosis in gastric cancer patients (102). Overexpression of LINC00944 promotes tumor occurrence and is significantly associated with the staging and poor prognosis of renal cell carcinoma (103). A previous study indicated that MIR503HG is involved in the metastasis of non-small cell lung cancer cells (104). Our study suggests that these mRNA-miRNA-lncRNA networks are associated with the prognosis of SARC patients. All of these pieces of evidence indicate that these regulatory axes may play important roles in the progression of SARC. However, further experimental research is warranted to confirm these results.

## 5 Conclusion

In summary, based on the URGs prognosis model constructed in this study, our results demonstrate a significant correlation between high URGs expression and the clinical prognosis of SARC patients, as well as with DNA methylation and immunotherapy. We have also identified related regulatory axes that may play crucial roles in the invasion and metastasis of SARC. These findings suggest that URGs could serve as potential prognostic biomarkers and predictive factors for immunotherapy in SARC patients.

## Data Availability

The datasets presented in this study can be found in online repositories. The names of the repository/repositories and accession number(s) can be found in the article/**Supplementary Material**.
