# Supplementary material for: Integration of ubiquitination-related genes in predictive signatures for prognosis and immunotherapy response in sarcoma
Source: Front Oncol. 2024 Oct 14;14:1446522. doi: 10.3389/fonc.2024.1446522 (PMC11513255; doi:10.3389/fonc.2024.1446522)
Supplement: Supplementary file 1 [file DataSheet1.zip › Supplementary Table 4.docx]

**Supplementary Table 4. Presentation of part of the GO and KEGG pathway enrichment analysis for the DEGs associated with cancer genesis and progress**

| Ontology | ID | Description | GeneRatio | BgRatio | pvalue | p.adjust | qvalue |
| --- | --- | --- | --- | --- | --- | --- | --- |
| BP | GO:0031589 | cell-substrate adhesion | 23/258 | 354/18670 | 8.72e-10 | 1.49e-06 | 1.26e-06 |
| BP | GO:0030198 | extracellular matrix organization | 20/258 | 368/18670 | 2.16e-07 | 2.45e-04 | 2.09e-04 |
| BP | GO:0061640 | cytoskeleton-dependent cytokinesis | 10/258 | 100/18670 | 1.24e-06 | 7.96e-04 | 6.78e-04 |
| BP | GO:0140014 | mitotic nuclear division | 15/258 | 264/18670 | 4.30e-06 | 0.001 | 0.001 |
| BP | GO:0000819 | sister chromatid segregation | 12/258 | 189/18670 | 1.31e-05 | 0.003 | 0.002 |
| BP | GO:0000075 | cell cycle checkpoint | 12/258 | 216/18670 | 4.91e-05 | 0.005 | 0.004 |
| BP | GO:0019827 | stem cell population maintenance | 10/258 | 157/18670 | 6.74e-05 | 0.006 | 0.005 |
| BP | GO:0044843 | cell cycle G1/S phase transition | 14/258 | 298/18670 | 7.33e-05 | 0.006 | 0.005 |
| BP | GO:0003382 | epithelial cell morphogenesis | 5/258 | 34/18670 | 9.72e-05 | 0.008 | 0.007 |
| BP | GO:0030199 | collagen fibril organization | 6/258 | 54/18670 | 9.74e-05 | 0.008 | 0.007 |

| Ontology | ID | Description | GeneRatio | BgRatio | pvalue | p.adjust | qvalue |
| --- | --- | --- | --- | --- | --- | --- | --- |
| CC | GO:0005604 | basement membrane | 12/270 | 95/19717 | 6.79e-09 | 2.17e-06 | 1.68e-06 |
| CC | GO:0062023 | collagen-containing extracellular matrix | 23/270 | 406/19717 | 1.03e-08 | 2.17e-06 | 1.68e-06 |
| CC | GO:0005925 | focal adhesion | 21/270 | 405/19717 | 2.05e-07 | 2.29e-05 | 1.77e-05 |
| CC | GO:0005924 | cell-substrate adherens junction | 21/270 | 408/19717 | 2.32e-07 | 2.29e-05 | 1.77e-05 |
| CC | GO:0005819 | spindle | 16/270 | 347/19717 | 2.56e-05 | 0.001 | 0.001 |
| CC | GO:0098687 | chromosomal region | 16/270 | 349/19717 | 2.74e-05 | 0.001 | 0.001 |
| CC | GO:0034663 | endoplasmic reticulum chaperone complex | 3/270 | 11/19717 | 3.86e-04 | 0.009 | 0.007 |
| CC | GO:0005581 | collagen trimer | 6/270 | 87/19717 | 0.001 | 0.017 | 0.014 |

| Ontology | ID | Description | GeneRatio | BgRatio | pvalue | p.adjust | qvalue |
| --- | --- | --- | --- | --- | --- | --- | --- |
| MF | GO:0005201 | extracellular matrix structural constituent | 15/255 | 163/17697 | 1.43e-08 | 7.52e-06 | 6.81e-06 |
| MF | GO:0005178 | integrin binding | 13/255 | 132/17697 | 5.98e-08 | 1.58e-05 | 1.43e-05 |
| MF | GO:0050839 | cell adhesion molecule binding | 22/255 | 499/17697 | 3.55e-06 | 4.67e-04 | 4.23e-04 |
| MF | GO:0005518 | collagen binding | 8/255 | 67/17697 | 5.21e-06 | 5.49e-04 | 4.97e-04 |
| MF | GO:0043394 | proteoglycan binding | 6/255 | 36/17697 | 1.14e-05 | 0.001 | 9.09e-04 |
| MF | GO:0043236 | laminin binding | 5/255 | 29/17697 | 5.34e-05 | 0.004 | 0.004 |
| MF | GO:0050840 | extracellular matrix binding | 6/255 | 57/17697 | 1.66e-04 | 0.011 | 0.010 |
| MF | GO:0019838 | growth factor binding | 8/255 | 137/17697 | 8.42e-04 | 0.030 | 0.027 |
| MF | GO:0031267 | small GTPase binding | 15/255 | 443/17697 | 0.002 | 0.048 | 0.043 |

| Ontology | ID | Description | GeneRatio | BgRatio | pvalue | p.adjust | qvalue |
| --- | --- | --- | --- | --- | --- | --- | --- |
| KEGG | hsa04510 | Focal adhesion | 16/128 | 201/8076 | 9.60e-08 | 1.32e-05 | 1.21e-05 |
| KEGG | hsa04512 | ECM-receptor interaction | 11/128 | 88/8076 | 1.17e-07 | 1.32e-05 | 1.21e-05 |
| KEGG | hsa04392 | Hippo signaling pathway - multiple species | 5/128 | 29/8076 | 8.09e-05 | 0.006 | 0.006 |
| KEGG | hsa04151 | PI3K-Akt signaling pathway | 16/128 | 354/8076 | 1.39e-04 | 0.006 | 0.006 |
| KEGG | hsa04145 | Phagosome | 10/128 | 152/8076 | 1.41e-04 | 0.006 | 0.006 |
| KEGG | hsa04810 | Regulation of actin cytoskeleton | 12/128 | 218/8076 | 1.66e-04 | 0.006 | 0.006 |
| KEGG | hsa05165 | Human papillomavirus infection | 15/128 | 331/8076 | 2.22e-04 | 0.007 | 0.007 |
| KEGG | hsa05205 | Proteoglycans in cancer | 11/128 | 205/8076 | 3.91e-04 | 0.011 | 0.010 |
| KEGG | hsa05414 | Dilated cardiomyopathy | 7/128 | 96/8076 | 7.89e-04 | 0.020 | 0.018 |
| KEGG | hsa04270 | Vascular smooth muscle contraction | 8/128 | 135/8076 | 0.001 | 0.030 | 0.027 |
