# Supplementary material for: Integration of ubiquitination-related genes in predictive signatures for prognosis and immunotherapy response in sarcoma
Source: Front Oncol. 2024 Oct 14;14:1446522. doi: 10.3389/fonc.2024.1446522 (PMC11513255; doi:10.3389/fonc.2024.1446522)
Supplement: Supplementary file 1 [file DataSheet1.zip › Supplementary Table 5.docx]

| ID | setSize | enrichmentScore | NES | pvalue | p.adjust | qvalue |
| --- | --- | --- | --- | --- | --- | --- |
| KEGG_ERBB_SIGNALING_PATHWAY | 87 | 0.5171526 | 1.565525 | 2e-05 | 0.0006 | 0.0004 |
| KEGG_GAP_JUNCTION | 83 | 0.5189980 | 1.566374 | 2e-05 | 0.0006 | 0.0004 |
| KEGG_TGF_BETA_SIGNALING_PATHWAY | 85 | 0.5157713 | 1.559020 | 3e-05 | 0.0008 | 0.0005 |
| KEGG_ECM_RECEPTOR_INTERACTION | 82 | 0.5550449 | 1.673882 | 1e-05 | 0.0004 | 0.0002 |
| KEGG_REGULATION_OF_ACTIN_CYTOSKELETON | 201 | 0.4452383 | 1.403070 | 4e-05 | 0.0010 | 0.0006 |
| KEGG_PATHWAYS_IN_CANCER | 317 | 0.4354323 | 1.392721 | 1e-05 | 0.0004 | 0.0002 |
| KEGG_FOCAL_ADHESION | 198 | 0.5294089 | 1.667532 | 1e-05 | 0.0004 | 0.0002 |
| KEGG_CELL_CYCLE | 124 | 0.5372272 | 1.658207 | 1e-05 | 0.0004 | 0.0002 |

**Supplementary Table 5. Gene sets enriched in phenotype high.**
