# Supplementary material for: Integration of ubiquitination-related genes in predictive signatures for prognosis and immunotherapy response in sarcoma
Source: Front Oncol. 2024 Oct 14;14:1446522. doi: 10.3389/fonc.2024.1446522 (PMC11513255; doi:10.3389/fonc.2024.1446522)
Supplement: Supplementary file 1 [file DataSheet1.zip › Supplementary Table 6.docx]

**Supplementary Table 6 The clinical characters of SARC patients in TCGA cohort**

|  | Age | Sex | Race | newTumor | Radiation | Neoadjuvant | Therapy | OS | OS.time | DSS | DSS.time | DFI | DFI.time | PFI | PFI.time | Groups | Risk | riskScore |
| --- | --- | --- | --- | --- | --- | --- | --- | --- | --- | --- | --- | --- | --- | --- | --- | --- | --- | --- |
| TCGA-3B-A9HI | 68 | MALE | WHITE | Recurrence | YES | No | Chemotherapy | 0 | 1521 | 0 | 1521 | NA | NA | 1 | 606 | C1 | High_risk | 6.242545078 |
| TCGA-3B-A9HJ | 68 | MALE | WHITE | NA | YES | No | NA | 0 | 1104 | 0 | 1104 | NA | NA | 0 | 1104 | C2 | Low_risk | 5.459974446 |
| TCGA-3B-A9HL | 67 | MALE | WHITE | Recurrence | NO | No | Chemotherapy | 1 | 599 | 0 | 599 | NA | NA | 1 | 229 | C1 | High_risk | 6.146558526 |
| TCGA-3B-A9HO | 75 | MALE | WHITE | Primary | NO | Yes | NA | 0 | 959 | 0 | 959 | NA | NA | 1 | 595 | C2 | High_risk | 6.24631909 |
| TCGA-3B-A9HP | 57 | FEMALE | WHITE | Metastasis | YES | No | Chemotherapy | 1 | 1627 | 1 | 1627 | NA | NA | 1 | 517 | C1 | High_risk | 6.126846901 |
| TCGA-3B-A9HQ | 66 | FEMALE | WHITE | NA | NO | No | NA | 0 | 2085 | 0 | 2085 | 0 | 2085 | 0 | 2085 | C1 | Low_risk | 5.587230002 |
| TCGA-3B-A9HR | 38 | FEMALE | BLACK | Metastasis | YES | No | Chemotherapy | 1 | 2694 | 1 | 2694 | 1 | 496 | 1 | 496 | C1 | Low_risk | 6.055045321 |
| TCGA-3B-A9HS | 60 | MALE | WHITE | Recurrence | YES | No | Chemotherapy | 1 | 1366 | 1 | 1366 | 1 | 139 | 1 | 139 | C1 | High_risk | 6.385476533 |
| TCGA-3B-A9HT | 53 | MALE | WHITE | Primary | YES | No | Chemotherapy | 0 | 1665 | 0 | 1665 | 0 | 1665 | 1 | 670 | C1 | High_risk | 6.594707602 |
| TCGA-3B-A9HU | 53 | FEMALE | BLACK | Metastasis | NO | No | NA | 0 | 768 | 0 | 768 | 1 | 631 | 1 | 631 | C1 | High_risk | 6.219763084 |
| TCGA-3B-A9HV | 48 | FEMALE | WHITE | Metastasis | NO | No | Chemotherapy | 1 | 437 | 1 | 437 | NA | NA | 1 | 41 | C1 | High_risk | 6.800195207 |
| TCGA-3B-A9HX | 70 | FEMALE | WHITE | NA | NO | No | NA | 0 | 1303 | 0 | 1303 | 0 | 1303 | 0 | 1303 | C1 | Low_risk | 6.09774081 |
| TCGA-3B-A9HY | 49 | MALE | WHITE | NA | NO | No | NA | 0 | 314 | 0 | 314 | 0 | 314 | 0 | 314 | C1 | Low_risk | 5.590489008 |
| TCGA-3B-A9HZ | 66 | MALE | WHITE | Metastasis | YES | No | Chemotherapy | 0 | 1170 | 0 | 1170 | NA | NA | 1 | 309 | C1 | Low_risk | 6.065370655 |
| TCGA-3B-A9I0 | 33 | MALE | WHITE | Metastasis | NO | No | Chemotherapy | 0 | 1096 | 0 | 1096 | 1 | 503 | 1 | 503 | C1 | Low_risk | 5.710047182 |
| TCGA-3B-A9I1 | 56 | FEMALE | BLACK | Metastasis | NO | No | NA | 1 | 567 | 1 | 567 | NA | NA | 1 | 192 | C1 | High_risk | 6.651330753 |
| TCGA-3B-A9I3 | 62 | MALE | WHITE | Primary | NO | No | NA | 0 | 808 | 0 | 808 | NA | NA | 1 | 87 | C1 | High_risk | 6.408604185 |
| TCGA-3R-A8YX | 66 | MALE | NA | NA | NO | No | NA | 1 | 17 | 1 | 17 | NA | NA | 1 | 17 | C1 | High_risk | 6.881496816 |
| TCGA-DX-A1KU | 82 | MALE | WHITE | Metastasis | NA | No | NA | 1 | 427 | 1 | 427 | NA | NA | 1 | 94 | C1 | High_risk | 6.257870999 |
| TCGA-DX-A1KW | 67 | MALE | WHITE | NA | NA | No | NA | 1 | 995 | NA | 995 | NA | NA | 0 | 995 | C1 | High_risk | 6.273061792 |
| TCGA-DX-A1KX | 67 | FEMALE | WHITE | NA | NA | No | NA | 0 | 2087 | 0 | 2087 | 0 | 2087 | 0 | 2087 | C1 | High_risk | 6.317174584 |
| TCGA-DX-A1KY | 66 | MALE | WHITE | Metastasis | NA | No | NA | 1 | 391 | 1 | 391 | NA | NA | 1 | 205 | C1 | High_risk | 6.344860896 |
| TCGA-DX-A1KZ | 66 | MALE | WHITE | Recurrence | NA | No | NA | 0 | 1372 | 0 | 1372 | NA | NA | 1 | 1294 | C1 | Low_risk | 5.586781121 |
| TCGA-DX-A1L0 | 58 | MALE | WHITE | Recurrence | NA | No | NA | 1 | 564 | 1 | 564 | NA | NA | 1 | 103 | C1 | Low_risk | 6.09128104 |
| TCGA-DX-A1L1 | 60 | MALE | WHITE | Recurrence | NA | No | NA | 1 | 1649 | 1 | 1649 | 1 | 349 | 1 | 349 | C1 | High_risk | 6.977936629 |
| TCGA-DX-A1L2 | 78 | MALE | WHITE | Recurrence | NA | No | NA | 0 | 2586 | 0 | 2586 | 1 | 1480 | 1 | 1480 | C1 | Low_risk | 5.689127109 |
| TCGA-DX-A1L3 | 60 | FEMALE | WHITE | Recurrence | NA | No | NA | 1 | 1020 | 1 | 1020 | NA | NA | 1 | 437 | C1 | Low_risk | 5.890924475 |
| TCGA-DX-A1L4 | 60 | MALE | WHITE | Recurrence | NA | No | NA | 0 | 2036 | 0 | 2036 | 1 | 340 | 1 | 340 | C1 | Low_risk | 5.876744233 |
| TCGA-DX-A23R | 55 | FEMALE | WHITE | NA | NA | No | NA | 0 | 2271 | 0 | 2271 | NA | NA | 0 | 2271 | C1 | High_risk | 6.605029707 |
| TCGA-DX-A23T | 40 | FEMALE | WHITE | Recurrence | NA | No | NA | 1 | 160 | 1 | 160 | NA | NA | 1 | 104 | C1 | Low_risk | 6.106942432 |
| TCGA-DX-A23U | 81 | MALE | WHITE | Recurrence | NA | No | NA | 1 | 2324 | 1 | 2324 | NA | NA | 1 | 873 | C1 | Low_risk | 5.697741401 |
| TCGA-DX-A23V | 57 | FEMALE | WHITE | Recurrence | NA | No | NA | 1 | 2034 | 1 | 2034 | NA | NA | 1 | 378 | C1 | High_risk | 6.666005654 |
| TCGA-DX-A23Y | 80 | MALE | WHITE | Recurrence | NA | No | NA | 1 | 191 | 1 | 191 | NA | NA | 1 | 172 | C1 | High_risk | 6.398337379 |
| TCGA-DX-A23Z | 74 | MALE | WHITE | Recurrence | NA | No | NA | 1 | 846 | 1 | 846 | NA | NA | 1 | 751 | C1 | High_risk | 6.500852897 |
| TCGA-DX-A240 | 51 | MALE | WHITE | NA | NA | No | NA | 0 | 1834 | 0 | 1834 | 0 | 1834 | 0 | 1834 | C1 | Low_risk | 5.721800896 |
| TCGA-DX-A2IZ | 60 | MALE | WHITE | NA | NA | No | NA | 0 | 261 | 0 | 261 | 0 | 261 | 0 | 261 | C1 | High_risk | 6.701558445 |
| TCGA-DX-A2J0 | 61 | MALE | WHITE | Recurrence | NA | No | NA | 1 | 295 | 1 | 295 | NA | NA | 1 | 213 | C1 | High_risk | 6.246063348 |
| TCGA-DX-A2J1 | 53 | MALE | WHITE | NA | NA | No | NA | 0 | 2901 | 0 | 2901 | NA | NA | 0 | 2901 | C2 | High_risk | 6.143733946 |
| TCGA-DX-A2J4 | 60 | MALE | WHITE | NA | NA | No | NA | 0 | 2408 | 0 | 2408 | 0 | 2408 | 0 | 2408 | C2 | Low_risk | 5.626005795 |
| TCGA-DX-A3LS | 78 | FEMALE | WHITE | Metastasis | NO | No | NA | 1 | 275 | 1 | 275 | NA | NA | 1 | 114 | C1 | High_risk | 7.012415302 |
| TCGA-DX-A3LT | 62 | MALE | WHITE | Recurrence | YES | No | NA | 0 | 1492 | 0 | 1492 | 1 | 568 | 1 | 568 | C1 | High_risk | 6.498434961 |
| TCGA-DX-A3LU | 60 | MALE | WHITE | Recurrence | NO | No | Chemotherapy | 0 | 2641 | 0 | 2641 | NA | NA | 1 | 290 | C2 | Low_risk | 5.689704086 |
| TCGA-DX-A3LW | 43 | MALE | WHITE | Recurrence | NO | No | NA | 0 | 4150 | 0 | 4150 | NA | NA | 1 | 1125 | C1 | High_risk | 6.495899888 |
| TCGA-DX-A3LY | 66 | MALE | WHITE | NA | NO | No | NA | 0 | 1017 | 0 | 1017 | 0 | 1017 | 0 | 1017 | C1 | Low_risk | 5.251562242 |
| TCGA-DX-A3M1 | 79 | MALE | WHITE | Recurrence | NO | No | NA | 0 | 1359 | 0 | 1359 | NA | NA | 1 | 1212 | C1 | Low_risk | 6.029110865 |
| TCGA-DX-A3M2 | 59 | MALE | WHITE | NA | NO | No | NA | 0 | 1055 | 0 | 1055 | NA | NA | 0 | 1055 | C2 | Low_risk | 5.710091757 |
| TCGA-DX-A3U5 | 52 | MALE | WHITE | NA | NO | No | NA | 0 | 969 | 0 | 969 | NA | NA | 0 | 969 | C1 | High_risk | 6.418889899 |
| TCGA-DX-A3U6 | 63 | FEMALE | WHITE | Recurrence | NO | No | Chemotherapy | 1 | 1088 | 1 | 1088 | 1 | 160 | 1 | 160 | C2 | Low_risk | 5.611569232 |
| TCGA-DX-A3U7 | 67 | MALE | WHITE | Metastasis | NO | No | Chemotherapy | 1 | 1552 | 1 | 1552 | 1 | 349 | 1 | 349 | C1 | High_risk | 6.688040257 |
| TCGA-DX-A3U8 | 43 | MALE | NA | Metastasis | NO | No | NA | 1 | 138 | 1 | 138 | 1 | 128 | 1 | 128 | C1 | High_risk | 7.011802551 |
| TCGA-DX-A3U9 | 84 | MALE | WHITE | Recurrence | NO | No | NA | 1 | 456 | 1 | 456 | NA | NA | 1 | 203 | C1 | High_risk | 6.524227159 |
| TCGA-DX-A3UA | 47 | FEMALE | WHITE | NA | NO | No | NA | 1 | 325 | NA | 325 | 0 | 325 | 0 | 325 | C1 | Low_risk | 6.12300655 |
| TCGA-DX-A3UB | 54 | MALE | WHITE | NA | NO | No | NA | 0 | 2247 | 0 | 2247 | 0 | 2247 | 0 | 2247 | C2 | Low_risk | 5.492173651 |
| TCGA-DX-A3UC | 37 | FEMALE | WHITE | Metastasis | NO | No | Chemotherapy | 0 | 2085 | 0 | 2085 | 1 | 176 | 1 | 176 | C1 | Low_risk | 5.617503146 |
| TCGA-DX-A3UD | 56 | MALE | WHITE | Recurrence | NO | No | NA | 0 | 1534 | 0 | 1534 | 1 | 603 | 1 | 603 | C1 | High_risk | 6.29170589 |
| TCGA-DX-A3UE | 66 | FEMALE | WHITE | Metastasis | YES | No | NA | 1 | 1061 | 1 | 1061 | 1 | 224 | 1 | 224 | C2 | Low_risk | 5.189904023 |
| TCGA-DX-A3UF | 65 | FEMALE | WHITE | Recurrence | NO | No | Chemotherapy | 1 | 2599 | 1 | 2599 | NA | NA | 1 | 964 | C1 | Low_risk | 5.582651504 |
| TCGA-DX-A48J | 44 | MALE | BLACK | Recurrence | NO | No | NA | 1 | 296 | 1 | 296 | 1 | 97 | 1 | 97 | C1 | Low_risk | 5.751420047 |
| TCGA-DX-A48K | 65 | MALE | WHITE | NA | NO | No | NA | 0 | 1399 | 0 | 1399 | 0 | 1399 | 0 | 1399 | C1 | Low_risk | 5.626724741 |
| TCGA-DX-A48L | 49 | FEMALE | WHITE | Metastasis | YES | No | NA | 1 | 738 | 1 | 738 | 1 | 316 | 1 | 316 | C1 | Low_risk | 5.931266011 |
| TCGA-DX-A48N | 47 | MALE | WHITE | NA | NO | No | NA | 0 | 1073 | 0 | 1073 | NA | NA | 0 | 1073 | C1 | High_risk | 6.206456057 |
| TCGA-DX-A48O | 63 | FEMALE | WHITE | NA | NO | No | NA | 0 | 1605 | 0 | 1605 | 0 | 1605 | 0 | 1605 | C2 | Low_risk | 5.573980857 |
| TCGA-DX-A48P | 54 | FEMALE | WHITE | NA | NO | No | NA | 0 | 1400 | 0 | 1400 | 0 | 1400 | 0 | 1400 | C1 | Low_risk | 6.012170076 |
| TCGA-DX-A48R | 41 | MALE | WHITE | Recurrence | NO | No | NA | 0 | 805 | 0 | 805 | NA | NA | 1 | 315 | C1 | Low_risk | 6.108239271 |
| TCGA-DX-A48U | 57 | MALE | WHITE | NA | NO | No | NA | 0 | 3310 | 0 | 3310 | 0 | 3310 | 0 | 3310 | C1 | Low_risk | 6.120231187 |
| TCGA-DX-A6B7 | 55 | MALE | WHITE | NA | NO | No | Chemotherapy | 0 | 1143 | 0 | 1143 | 0 | 1143 | 0 | 1143 | C1 | Low_risk | 5.968826429 |
| TCGA-DX-A6B8 | 80 | MALE | WHITE | Metastasis | YES | No | Chemotherapy | 1 | 485 | 1 | 485 | 1 | 42 | 1 | 42 | C1 | High_risk | 6.848959632 |
| TCGA-DX-A6B9 | 45 | FEMALE | BLACK | NA | NO | No | NA | 0 | 1138 | 0 | 1138 | 0 | 1138 | 0 | 1138 | C1 | Low_risk | 5.867708228 |
| TCGA-DX-A6BA | 53 | FEMALE | WHITE | Recurrence | NO | No | NA | 0 | 2515 | 0 | 2515 | 1 | 408 | 1 | 408 | C1 | Low_risk | 6.043878403 |
| TCGA-DX-A6BB | 74 | MALE | WHITE | NA | NO | No | NA | 0 | 1105 | 0 | 1105 | NA | NA | 0 | 1105 | C2 | Low_risk | 5.537884012 |
| TCGA-DX-A6BE | 77 | MALE | WHITE | NA | NO | No | NA | 0 | 881 | 0 | 881 | 0 | 881 | 0 | 881 | C1 | Low_risk | 5.630504775 |
| TCGA-DX-A6BF | 82 | FEMALE | WHITE | Recurrence | NO | No | NA | 0 | 972 | 0 | 972 | NA | NA | 1 | 947 | C2 | Low_risk | 5.582288825 |
| TCGA-DX-A6BG | 51 | MALE | WHITE | Recurrence | NO | No | Chemotherapy | 0 | 1121 | 0 | 1121 | 1 | 149 | 1 | 149 | C1 | High_risk | 6.274529656 |
| TCGA-DX-A6BH | 34 | MALE | WHITE | Recurrence | NO | No | NA | 0 | 821 | 0 | 821 | NA | NA | 1 | 232 | C1 | Low_risk | 5.754372855 |
| TCGA-DX-A6YQ | 86 | FEMALE | NA | NA | NO | No | NA | 1 | 93 | 0 | 93 | 0 | 93 | 0 | 93 | C1 | High_risk | 6.581309569 |
| TCGA-DX-A6YR | 75 | MALE | WHITE | NA | YES | No | NA | 0 | 1247 | 0 | 1247 | 0 | 1247 | 0 | 1247 | C1 | High_risk | 6.216800517 |
| TCGA-DX-A6YS | 61 | MALE | WHITE | NA | YES | No | NA | 0 | 2381 | 0 | 2381 | 0 | 2381 | 0 | 2381 | C1 | Low_risk | 5.437126437 |
| TCGA-DX-A6YT | 31 | FEMALE | WHITE | NA | NO | No | NA | 0 | 2044 | 0 | 2044 | 0 | 2044 | 0 | 2044 | C1 | Low_risk | 6.059810489 |
| TCGA-DX-A6YU | 50 | FEMALE | WHITE | NA | NO | No | NA | 0 | 1045 | 0 | 1045 | 0 | 1045 | 0 | 1045 | C1 | Low_risk | 5.848695968 |
| TCGA-DX-A6YV | 74 | MALE | WHITE | Metastasis | NO | No | NA | 1 | 1536 | 1 | 1536 | 1 | 516 | 1 | 516 | C1 | High_risk | 6.902584925 |
| TCGA-DX-A6YX | 68 | FEMALE | NA | Metastasis | YES | No | Chemotherapy | 1 | 513 | 1 | 513 | 1 | 200 | 1 | 200 | C1 | High_risk | 7.160373344 |
| TCGA-DX-A6YZ | 59 | MALE | WHITE | NA | YES | No | NA | 0 | 2625 | 0 | 2625 | 0 | 2625 | 0 | 2625 | C1 | Low_risk | 5.513396788 |
| TCGA-DX-A6Z0 | 78 | FEMALE | WHITE | NA | YES | No | NA | 1 | 1953 | 0 | 1953 | NA | NA | 0 | 1953 | C1 | High_risk | 6.416742735 |
| TCGA-DX-A6Z2 | 82 | FEMALE | WHITE | Metastasis | NO | No | NA | 0 | 3079 | 0 | 3079 | 1 | 2659 | 1 | 2659 | C1 | Low_risk | 6.0503424 |
| TCGA-DX-A7EF | 88 | FEMALE | WHITE | Metastasis | YES | No | NA | 1 | 648 | 1 | 648 | 1 | 294 | 1 | 294 | C1 | High_risk | 6.256410197 |
| TCGA-DX-A7EI | 78 | FEMALE | WHITE | Recurrence | NO | No | Chemotherapy | 1 | 1049 | 1 | 1049 | NA | NA | 1 | 828 | C1 | Low_risk | 5.64875311 |
| TCGA-DX-A7EL | 56 | FEMALE | WHITE | Metastasis | NO | No | NA | 0 | 2291 | 0 | 2291 | 1 | 2249 | 1 | 2249 | C1 | Low_risk | 5.88540929 |
| TCGA-DX-A7EM | 61 | MALE | WHITE | NA | NO | No | NA | 0 | 1892 | 0 | 1892 | 0 | 1892 | 0 | 1892 | C1 | Low_risk | 5.763065695 |
| TCGA-DX-A7EN | 53 | FEMALE | WHITE | Metastasis | NO | No | Chemotherapy | 0 | 923 | 0 | 923 | 1 | 352 | 1 | 352 | C1 | High_risk | 6.68665376 |
| TCGA-DX-A7EO | 20 | FEMALE | WHITE | NA | YES | No | NA | 0 | 4627 | 0 | 4627 | 0 | 4627 | 0 | 4627 | C2 | Low_risk | 6.071498098 |
| TCGA-DX-A7EQ | 72 | MALE | WHITE | Metastasis | YES | No | NA | 1 | 897 | 1 | 897 | 1 | 251 | 1 | 251 | C2 | Low_risk | 4.847112518 |
| TCGA-DX-A7ER | 69 | MALE | WHITE | NA | NO | No | NA | 1 | 1466 | 0 | 1466 | 0 | 1466 | 0 | 1466 | C2 | Low_risk | 6.058167918 |
| TCGA-DX-A7ES | 50 | FEMALE | WHITE | NA | NO | No | NA | 0 | 610 | 0 | 610 | 0 | 610 | 0 | 610 | C2 | Low_risk | 5.650421621 |
| TCGA-DX-A7ET | 71 | MALE | BLACK | NA | NO | No | NA | 0 | 463 | 0 | 463 | 0 | 463 | 0 | 463 | C2 | Low_risk | 5.943589991 |
| TCGA-DX-A7EU | 79 | FEMALE | WHITE | NA | NO | No | NA | 0 | 721 | 0 | 721 | 0 | 721 | 0 | 721 | C1 | Low_risk | 5.27475665 |
| TCGA-DX-A8BG | 83 | FEMALE | WHITE | Metastasis | YES | No | NA | 0 | 546 | 0 | 546 | 1 | 330 | 1 | 330 | C2 | Low_risk | 5.291406956 |
| TCGA-DX-A8BH | 86 | MALE | WHITE | Metastasis | NO | No | Chemotherapy | 1 | 439 | 1 | 439 | 1 | 272 | 1 | 272 | C1 | High_risk | 6.729258645 |
| TCGA-DX-A8BJ | 76 | MALE | WHITE | NA | YES | No | NA | 1 | 284 | 0 | 284 | 0 | 284 | 0 | 284 | C1 | Low_risk | 6.087048737 |
| TCGA-DX-A8BK | 61 | FEMALE | WHITE | NA | YES | No | NA | 0 | 1885 | 0 | 1885 | 0 | 1885 | 0 | 1885 | C2 | Low_risk | 5.648296819 |
| TCGA-DX-A8BL | 59 | MALE | WHITE | NA | NO | No | NA | 0 | 1373 | 0 | 1373 | 0 | 1373 | 0 | 1373 | C2 | Low_risk | 5.317554827 |
| TCGA-DX-A8BM | 60 | MALE | WHITE | NA | YES | No | NA | 0 | 1456 | 0 | 1456 | 0 | 1456 | 0 | 1456 | C1 | Low_risk | 5.878513254 |
| TCGA-DX-A8BN | 78 | FEMALE | WHITE | NA | YES | No | NA | 0 | 1161 | 0 | 1161 | NA | NA | 0 | 1161 | C1 | Low_risk | 6.101016522 |
| TCGA-DX-A8BO | 67 | FEMALE | WHITE | Recurrence | NO | No | NA | 0 | 1240 | 0 | 1240 | NA | NA | 1 | 333 | C1 | Low_risk | 6.123318872 |
| TCGA-DX-A8BP | 85 | MALE | WHITE | Metastasis | NO | No | NA | 0 | 428 | 0 | 428 | NA | NA | 1 | 216 | C1 | High_risk | 6.464118545 |
| TCGA-DX-A8BQ | 63 | MALE | WHITE | NA | YES | No | NA | 0 | 1579 | 0 | 1579 | 0 | 1579 | 0 | 1579 | C1 | Low_risk | 5.963655867 |
| TCGA-DX-A8BR | 63 | FEMALE | WHITE | NA | YES | No | NA | 0 | 4573 | 0 | 4573 | 0 | 4573 | 0 | 4573 | C1 | Low_risk | 5.932558894 |
| TCGA-DX-A8BS | 68 | FEMALE | BLACK | NA | YES | No | NA | 1 | 850 | 0 | 850 | 0 | 850 | 0 | 850 | C1 | High_risk | 6.639253137 |
| TCGA-DX-A8BT | 63 | FEMALE | WHITE | Recurrence | YES | No | Chemotherapy | 0 | 1230 | 0 | 1230 | NA | NA | 1 | 668 | C1 | High_risk | 6.318430349 |
| TCGA-DX-A8BU | 58 | MALE | WHITE | Metastasis | YES | No | Chemotherapy | 1 | 1116 | 1 | 1116 | NA | NA | 1 | 107 | C1 | High_risk | 6.434993254 |
| TCGA-DX-A8BV | 57 | MALE | WHITE | Recurrence | YES | No | Chemotherapy | 1 | 1845 | 1 | 1845 | 1 | 357 | 1 | 357 | C1 | High_risk | 6.341425861 |
| TCGA-DX-A8BX | 52 | MALE | WHITE | NA | NO | No | NA | 1 | 591 | 1 | 591 | NA | NA | 1 | 591 | C1 | Low_risk | 6.05650227 |
| TCGA-DX-A8BZ | 78 | FEMALE | BLACK | Metastasis | NO | No | NA | 1 | 200 | 1 | 200 | 1 | 90 | 1 | 90 | C2 | Low_risk | 5.413812204 |
| TCGA-DX-AATS | 72 | MALE | WHITE | NA | NO | No | NA | 1 | 35 | 0 | 35 | NA | NA | 0 | 35 | C1 | High_risk | 6.619260602 |
| TCGA-DX-AB2E | 60 | MALE | WHITE | Metastasis | NO | No | Chemotherapy | 0 | 541 | 0 | 541 | 1 | 132 | 1 | 132 | C1 | High_risk | 6.68817282 |
| TCGA-DX-AB2F | 76 | FEMALE | WHITE | NA | NO | No | NA | 0 | 365 | 0 | 365 | 0 | 365 | 0 | 365 | C1 | Low_risk | 6.037036737 |
| TCGA-DX-AB2G | 36 | MALE | WHITE | NA | NO | No | NA | 0 | 478 | 0 | 478 | 0 | 478 | 0 | 478 | C1 | High_risk | 6.267601426 |
| TCGA-DX-AB2H | 80 | FEMALE | WHITE | NA | NO | No | NA | 0 | 482 | 0 | 482 | 0 | 482 | 0 | 482 | C1 | High_risk | 6.760738149 |
| TCGA-DX-AB2J | 71 | FEMALE | WHITE | NA | NO | No | NA | 1 | 1235 | 1 | 1235 | NA | NA | 1 | 1235 | C1 | High_risk | 6.230081125 |
| TCGA-DX-AB2L | 35 | MALE | WHITE | NA | NO | No | NA | 0 | 2007 | 0 | 2007 | NA | NA | 0 | 2007 | C1 | Low_risk | 5.933600702 |
| TCGA-DX-AB2O | 78 | FEMALE | WHITE | NA | NO | No | NA | 0 | 2619 | 0 | 2619 | 0 | 2619 | 0 | 2619 | C1 | Low_risk | 5.329356161 |
| TCGA-DX-AB2P | 79 | MALE | WHITE | Recurrence | YES | No | NA | 1 | 367 | 1 | 367 | 1 | 161 | 1 | 161 | C1 | High_risk | 6.220378998 |
| TCGA-DX-AB2Q | 65 | FEMALE | WHITE | Metastasis | YES | No | NA | 0 | 2030 | 0 | 2030 | 1 | 490 | 1 | 490 | C1 | High_risk | 6.311675826 |
| TCGA-DX-AB2S | 53 | FEMALE | WHITE | NA | YES | No | NA | 0 | 280 | 0 | 280 | 0 | 280 | 0 | 280 | C2 | Low_risk | 5.526729566 |
| TCGA-DX-AB2T | 54 | FEMALE | BLACK | Metastasis | NO | No | NA | 0 | 2369 | 0 | 2369 | 1 | 784 | 1 | 784 | C1 | High_risk | 6.198798587 |
| TCGA-DX-AB2V | 81 | MALE | WHITE | NA | YES | No | NA | 0 | 3241 | 0 | 3241 | 0 | 3241 | 0 | 3241 | C1 | Low_risk | 6.106261758 |
| TCGA-DX-AB2W | 62 | FEMALE | WHITE | NA | YES | No | NA | 0 | 3238 | 0 | 3238 | 0 | 3238 | 0 | 3238 | C1 | High_risk | 6.313276125 |
| TCGA-DX-AB2X | 73 | FEMALE | WHITE | Recurrence | NO | No | NA | 1 | 404 | 1 | 404 | 1 | 111 | 1 | 111 | C1 | High_risk | 6.28128417 |
| TCGA-DX-AB2Z | 87 | FEMALE | WHITE | NA | NO | No | NA | 0 | 1533 | 0 | 1533 | 0 | 1533 | 0 | 1533 | C1 | High_risk | 6.452542153 |
| TCGA-DX-AB30 | 53 | FEMALE | WHITE | Metastasis | YES | No | NA | 1 | 695 | 1 | 695 | 1 | 504 | 1 | 504 | C1 | Low_risk | 6.103220974 |
| TCGA-DX-AB32 | 51 | MALE | WHITE | NA | YES | No | NA | 0 | 3102 | 0 | 3102 | 0 | 3102 | 0 | 3102 | C1 | High_risk | 6.344064616 |
| TCGA-DX-AB35 | 65 | MALE | WHITE | Recurrence | NO | No | NA | 0 | 928 | 0 | 928 | 1 | 284 | 1 | 284 | C1 | High_risk | 6.229708872 |
| TCGA-DX-AB36 | 86 | MALE | WHITE | NA | NO | No | NA | 1 | 1825 | NA | 1825 | NA | NA | 0 | 1825 | C1 | Low_risk | 6.054156865 |
| TCGA-DX-AB37 | 73 | FEMALE | WHITE | NA | NO | No | NA | 1 | 2575 | NA | 2575 | 0 | 2575 | 0 | 2575 | C2 | Low_risk | 5.611404733 |
| TCGA-DX-AB3A | 80 | MALE | WHITE | Recurrence | NO | No | NA | 1 | 978 | 1 | 978 | 1 | 533 | 1 | 533 | C1 | High_risk | 6.544807897 |
| TCGA-DX-AB3B | 28 | FEMALE | WHITE | NA | NO | No | Chemotherapy | 0 | 1548 | 0 | 1548 | 0 | 1548 | 0 | 1548 | C1 | High_risk | 6.539798885 |
| TCGA-DX-AB3C | 27 | MALE | WHITE | Metastasis | YES | No | Chemotherapy | 0 | 2169 | 0 | 2169 | 1 | 567 | 1 | 567 | C2 | Low_risk | 5.923205857 |
| TCGA-FX-A2QS | 61 | FEMALE | WHITE | NA | NA | No | NA | 0 | 1495 | 0 | 1495 | 0 | 1495 | 0 | 1495 | C1 | High_risk | 6.191943816 |
| TCGA-FX-A3NJ | 54 | MALE | WHITE | NA | NA | No | NA | 0 | 947 | 0 | 947 | 0 | 947 | 0 | 947 | C1 | High_risk | 6.208349416 |
| TCGA-FX-A3NK | 53 | MALE | WHITE | NA | NA | No | NA | 0 | 505 | 0 | 505 | 0 | 505 | 0 | 505 | C1 | Low_risk | 5.974783475 |
| TCGA-FX-A3RE | 65 | FEMALE | WHITE | NA | NA | No | Chemotherapy | 0 | 660 | 0 | 660 | NA | NA | 0 | 660 | C1 | High_risk | 6.323260381 |
| TCGA-FX-A3TO | 87 | MALE | WHITE | NA | NA | No | NA | 0 | 618 | 0 | 618 | NA | NA | 0 | 618 | C1 | High_risk | 6.291081252 |
| TCGA-FX-A48G | 56 | FEMALE | WHITE | Metastasis | NA | No | Chemotherapy | 1 | 605 | 1 | 605 | NA | NA | 1 | 168 | C2 | Low_risk | 5.504127543 |
| TCGA-FX-A76Y | 49 | FEMALE | WHITE | NA | NO | No | NA | 0 | 471 | 0 | 471 | 0 | 471 | 0 | 471 | C1 | High_risk | 6.517258242 |
| TCGA-FX-A8OO | 31 | FEMALE | WHITE | NA | NO | No | Chemotherapy | 1 | 245 | 0 | 245 | 0 | 245 | 0 | 245 | C1 | High_risk | 6.178576962 |
| TCGA-HB-A2OT | 78 | FEMALE | BLACK | NA | NA | No | NA | 0 | 2057 | 0 | 2057 | 0 | 2057 | 0 | 2057 | C1 | High_risk | 6.499762826 |
| TCGA-HB-A3L4 | 46 | FEMALE | WHITE | Metastasis | NA | No | NA | 1 | 1061 | 1 | 1061 | 1 | 326 | 1 | 326 | C1 | High_risk | 6.573295464 |
| TCGA-HB-A3YV | 75 | MALE | WHITE | NA | YES | No | NA | 1 | 139 | 1 | 139 | NA | NA | 1 | 139 | C1 | High_risk | 6.478510676 |
| TCGA-HB-A43Z | 58 | FEMALE | WHITE | Metastasis | YES | No | Chemotherapy | 0 | 1126 | 0 | 1126 | 1 | 894 | 1 | 894 | C1 | High_risk | 6.128673539 |
| TCGA-HB-A5W3 | 58 | FEMALE | WHITE | NA | NO | No | NA | 1 | 384 | 1 | 384 | NA | NA | 1 | 384 | C1 | High_risk | 6.306687519 |
| TCGA-HS-A5N7 | 67 | FEMALE | NA | NA | YES | No | NA | 0 | 1013 | 0 | 1013 | 0 | 1013 | 0 | 1013 | C1 | High_risk | 6.238778257 |
| TCGA-HS-A5N8 | 69 | FEMALE | NA | NA | NA | No | NA | 1 | 22 | 1 | 22 | NA | NA | 1 | 22 | C1 | High_risk | 6.220413688 |
| TCGA-HS-A5N9 | 58 | FEMALE | NA | NA | NO | No | Chemotherapy | 0 | 1585 | 0 | 1585 | 0 | 1585 | 0 | 1585 | C1 | Low_risk | 5.375976891 |
| TCGA-HS-A5NA | 65 | FEMALE | NA | NA | YES | No | NA | 0 | 298 | 0 | 298 | NA | NA | 0 | 298 | C1 | High_risk | 6.384181072 |
| TCGA-IE-A3OV | 42 | MALE | WHITE | Metastasis | NA | No | NA | 1 | 2448 | 1 | 2448 | 1 | 490 | 1 | 490 | C1 | Low_risk | 5.880362805 |
| TCGA-IE-A4EH | 35 | FEMALE | WHITE | NA | NO | No | Chemotherapy | 0 | 819 | 0 | 819 | 0 | 819 | 0 | 819 | C1 | High_risk | 6.951459264 |
| TCGA-IE-A4EI | 67 | FEMALE | WHITE | NA | NO | No | NA | 0 | 594 | 0 | 594 | 0 | 594 | 0 | 594 | C1 | Low_risk | 5.996903549 |
| TCGA-IE-A4EJ | 84 | FEMALE | WHITE | NA | NA | No | NA | 0 | 98 | 0 | 98 | NA | NA | 0 | 98 | C1 | High_risk | 6.663807306 |
| TCGA-IE-A4EK | 54 | MALE | WHITE | NA | NA | No | NA | 0 | 42 | 0 | 42 | NA | NA | 0 | 42 | C1 | Low_risk | 5.927134273 |
| TCGA-IE-A6BZ | 65 | FEMALE | WHITE | NA | NO | No | NA | 0 | 605 | 0 | 605 | 0 | 605 | 0 | 605 | C1 | Low_risk | 5.869217842 |
| TCGA-IF-A3RQ | 70 | FEMALE | WHITE | Recurrence | NO | No | Chemotherapy | 1 | 1129 | 1 | 1129 | 1 | 441 | 1 | 441 | C1 | Low_risk | 5.71764993 |
| TCGA-IF-A4AJ | 80 | MALE | WHITE | Metastasis | NO | No | Chemotherapy | 1 | 767 | 1 | 767 | 1 | 560 | 1 | 560 | C1 | High_risk | 6.582604345 |
| TCGA-IF-A4AK | 82 | FEMALE | WHITE | NA | YES | No | NA | 0 | 1590 | 0 | 1590 | 0 | 1590 | 0 | 1590 | C1 | Low_risk | 5.734887464 |
| TCGA-IS-A3K6 | 55 | FEMALE | WHITE | NA | NA | No | NA | 1 | 858 | 1 | 858 | NA | NA | 1 | 858 | C1 | Low_risk | 5.763897488 |
| TCGA-IS-A3K7 | 63 | FEMALE | WHITE | Metastasis | NA | No | Chemotherapy | 0 | 5204 | 0 | 5204 | 1 | 119 | 1 | 119 | C2 | Low_risk | 5.312714848 |
| TCGA-IS-A3K8 | 44 | FEMALE | WHITE | NA | NA | No | NA | 0 | 4845 | 0 | 4845 | 0 | 4845 | 0 | 4845 | C2 | High_risk | 6.150482528 |
| TCGA-IS-A3KA | 73 | FEMALE | WHITE | Metastasis | NA | No | Hormone Therapy | 1 | 413 | 1 | 413 | 1 | 76 | 1 | 76 | C2 | Low_risk | 4.974349832 |
| TCGA-IW-A3M4 | 48 | FEMALE | WHITE | Recurrence | NA | No | Chemotherapy | 0 | 2615 | 0 | 2615 | 1 | 2207 | 1 | 2207 | C1 | High_risk | 6.56911874 |
| TCGA-IW-A3M5 | 76 | FEMALE | WHITE | Metastasis | NA | No | Chemotherapy | 1 | 180 | 1 | 180 | 1 | 9 | 1 | 9 | C1 | High_risk | 6.854532411 |
| TCGA-IW-A3M6 | 59 | FEMALE | ASIAN | NA | NA | No | Chemotherapy | 0 | 688 | 0 | 688 | 0 | 688 | 0 | 688 | C1 | Low_risk | 6.063276368 |
| TCGA-JV-A5VE | 60 | FEMALE | BLACK | NA | NO | No | Chemotherapy | 0 | 499 | 0 | 499 | 0 | 499 | 0 | 499 | C1 | High_risk | 6.605047651 |
| TCGA-JV-A5VF | 69 | FEMALE | BLACK | NA | NO | No | NA | 1 | 32 | 1 | 32 | NA | NA | 1 | 32 | C1 | High_risk | 6.971360893 |
| TCGA-JV-A75J | 43 | FEMALE | WHITE | NA | NO | No | Chemotherapy | 1 | 119 | 1 | 119 | NA | NA | 1 | 119 | C1 | Low_risk | 6.033733392 |
| TCGA-K1-A3PN | 64 | FEMALE | WHITE | NA | NA | No | Chemotherapy | 0 | 3080 | 0 | 3080 | 1 | 1033 | 1 | 1033 | C1 | Low_risk | 5.788025525 |
| TCGA-K1-A3PO | 42 | MALE | WHITE | NA | NA | No | NA | 0 | 1622 | 0 | 1622 | 0 | 1622 | 0 | 1622 | C1 | High_risk | 6.424316007 |
| TCGA-K1-A42W | 65 | FEMALE | WHITE | Metastasis | NO | No | NA | 0 | 1891 | 0 | 1891 | 1 | 826 | 1 | 826 | C1 | Low_risk | 5.933846766 |
| TCGA-K1-A42X | 62 | FEMALE | WHITE | Recurrence | NO | No | Chemotherapy | 0 | 3765 | 0 | 3765 | 1 | 2036 | 1 | 2036 | C1 | High_risk | 6.142815787 |
| TCGA-K1-A6RT | 48 | MALE | WHITE | Metastasis | YES | No | Chemotherapy | 0 | 528 | 0 | 528 | 1 | 129 | 1 | 129 | C2 | Low_risk | 5.154865816 |
| TCGA-K1-A6RU | 66 | FEMALE | WHITE | Metastasis | YES | No | Chemotherapy | 1 | 711 | 1 | 711 | NA | NA | 1 | 142 | C1 | Low_risk | 6.019106374 |
| TCGA-K1-A6RV | 67 | MALE | WHITE | NA | YES | No | NA | 0 | 158 | 0 | 158 | NA | NA | 0 | 158 | C1 | High_risk | 6.586668091 |
| TCGA-KD-A5QS | 55 | FEMALE | WHITE | Metastasis | NA | No | NA | 1 | 1941 | 1 | 1941 | NA | NA | 1 | 351 | C1 | High_risk | 7.541649696 |
| TCGA-KD-A5QT | 69 | FEMALE | NA | NA | NA | No | NA | 0 | 499 | 0 | 499 | 0 | 499 | 0 | 499 | C1 | High_risk | 6.329213571 |
| TCGA-KD-A5QU | 41 | FEMALE | WHITE | Metastasis | NA | No | Chemotherapy | 1 | 1073 | 1 | 1073 | 1 | 1060 | 1 | 1060 | C1 | High_risk | 7.004913816 |
| TCGA-KF-A41W | 71 | FEMALE | WHITE | NA | NO | No | Chemotherapy | 0 | 356 | 0 | 356 | NA | NA | 0 | 356 | C1 | High_risk | 6.801739552 |
| TCGA-LI-A67I | 75 | FEMALE | WHITE | Metastasis | YES | No | Chemotherapy | 0 | 922 | 0 | 922 | NA | NA | 1 | 574 | C1 | High_risk | 6.271075628 |
| TCGA-LI-A9QH | 72 | FEMALE | WHITE | Metastasis | YES | No | NA | 1 | 540 | 0 | 540 | NA | NA | 1 | 426 | C1 | Low_risk | 5.621689227 |
| TCGA-MB-A5Y8 | 60 | FEMALE | WHITE | NA | YES | No | NA | 0 | 773 | 0 | 773 | 0 | 773 | 0 | 773 | C1 | High_risk | 6.230952211 |
| TCGA-MB-A5Y9 | 90 | MALE | WHITE | NA | NO | No | NA | 0 | 252 | 0 | 252 | NA | NA | 0 | 252 | C2 | Low_risk | 5.53009396 |
| TCGA-MB-A5YA | 63 | MALE | WHITE | NA | NO | No | NA | 0 | 15 | 0 | 15 | 0 | 15 | 0 | 15 | C1 | Low_risk | 6.091806274 |
| TCGA-MB-A8JK | 49 | MALE | WHITE | NA | YES | No | NA | 0 | 661 | 0 | 661 | NA | NA | 0 | 661 | C1 | High_risk | 6.517243526 |
| TCGA-MB-A8JL | 53 | FEMALE | WHITE | NA | NO | No | NA | 0 | 600 | 0 | 600 | 0 | 600 | 0 | 600 | C1 | High_risk | 6.467118763 |
| TCGA-MJ-A68H | 57 | FEMALE | BLACK | NA | NO | No | NA | 0 | 456 | 0 | 456 | 0 | 456 | 0 | 456 | C1 | Low_risk | 5.832480697 |
| TCGA-MJ-A68J | 55 | FEMALE | BLACK | Primary | YES | No | NA | 0 | 547 | 0 | 547 | NA | NA | 1 | 268 | C1 | Low_risk | 5.880161983 |
| TCGA-MJ-A850 | 28 | MALE | WHITE | NA | YES | No | NA | 0 | 681 | 0 | 681 | NA | NA | 0 | 681 | C2 | Low_risk | 5.779345393 |
| TCGA-MO-A47P | 37 | FEMALE | ASIAN | NA | NO | No | NA | 0 | 352 | 0 | 352 | 0 | 352 | 0 | 352 | C1 | Low_risk | 6.120974115 |
| TCGA-MO-A47R | 75 | FEMALE | ASIAN | Metastasis | NO | No | NA | 1 | 318 | 1 | 318 | 1 | 259 | 1 | 259 | C1 | High_risk | 6.236502816 |
| TCGA-N1-A6IA | 41 | FEMALE | BLACK | Metastasis | NO | No | NA | 1 | 224 | 1 | 224 | NA | NA | 1 | 116 | C1 | High_risk | 6.79775667 |
| TCGA-PC-A5DK | 63 | MALE | WHITE | NA | NO | No | NA | 0 | 3740 | 0 | 3740 | 0 | 3740 | 0 | 3740 | C1 | Low_risk | 6.060437766 |
| TCGA-PC-A5DL | 67 | MALE | WHITE | NA | NO | No | NA | 1 | 1142 | NA | 1142 | NA | NA | 0 | 1142 | C1 | High_risk | 6.232784413 |
| TCGA-PC-A5DM | 73 | MALE | WHITE | NA | YES | No | NA | 1 | 193 | NA | 193 | NA | NA | 0 | 193 | C1 | Low_risk | 6.053169722 |
| TCGA-PC-A5DN | 77 | FEMALE | WHITE | NA | NO | No | NA | 1 | 1175 | 0 | 1175 | NA | NA | 0 | 1175 | C1 | High_risk | 6.189974603 |
| TCGA-PC-A5DO | 59 | FEMALE | WHITE | NA | NO | No | NA | 1 | 2464 | 1 | 2464 | NA | NA | 1 | 2464 | C1 | High_risk | 6.124906269 |
| TCGA-PC-A5DP | 72 | FEMALE | WHITE | NA | NO | No | NA | 1 | 550 | 1 | 550 | NA | NA | 1 | 550 | C1 | Low_risk | 5.831570767 |
| TCGA-PT-A8TR | 57 | FEMALE | WHITE | NA | NO | No | Chemotherapy | 0 | 813 | 0 | 813 | NA | NA | 0 | 813 | C1 | Low_risk | 5.992572152 |
| TCGA-QC-A6FX | 68 | MALE | WHITE | NA | YES | No | NA | 0 | 637 | 0 | 637 | 0 | 637 | 0 | 637 | C1 | High_risk | 6.777279272 |
| TCGA-QC-A7B5 | 75 | MALE | WHITE | Primary | NO | No | NA | 0 | 398 | 0 | 398 | NA | NA | 1 | 259 | C1 | Low_risk | 6.061320875 |
| TCGA-QC-AA9N | 53 | FEMALE | BLACK | NA | NO | No | NA | 0 | 537 | 0 | 537 | NA | NA | 0 | 537 | C1 | High_risk | 6.475512521 |
| TCGA-QQ-A5V2 | 42 | MALE | WHITE | NA | NA | No | NA | 0 | 56 | 0 | 56 | NA | NA | 0 | 56 | C2 | Low_risk | 5.232710129 |
| TCGA-QQ-A5V9 | 76 | MALE | WHITE | Metastasis | NO | No | NA | 1 | 1424 | 1 | 1424 | 1 | 405 | 1 | 405 | C1 | High_risk | 6.665951801 |
| TCGA-QQ-A5VA | 60 | FEMALE | WHITE | NA | NO | No | NA | 1 | 550 | 1 | 550 | NA | NA | 1 | 550 | C2 | Low_risk | 5.813789982 |
| TCGA-QQ-A5VB | 53 | FEMALE | WHITE | Recurrence | NO | No | Chemotherapy | 1 | 1478 | 1 | 1478 | NA | NA | 1 | 111 | C1 | High_risk | 6.987248316 |
| TCGA-QQ-A5VC | 63 | FEMALE | WHITE | NA | NO | No | NA | 0 | 1092 | 0 | 1092 | 0 | 1092 | 0 | 1092 | C1 | Low_risk | 5.672279791 |
| TCGA-QQ-A5VD | 52 | MALE | WHITE | NA | NO | No | NA | 0 | 1129 | 0 | 1129 | 0 | 1129 | 0 | 1129 | C1 | Low_risk | 5.459558924 |
| TCGA-QQ-A8VB | 68 | FEMALE | WHITE | Primary | NO | No | NA | 0 | 5723 | 0 | 5723 | NA | NA | 1 | 2682 | C1 | Low_risk | 6.059236596 |
| TCGA-QQ-A8VD | 56 | MALE | WHITE | Recurrence | NO | No | NA | 0 | 3964 | 0 | 3964 | NA | NA | 1 | 499 | C1 | Low_risk | 5.809149575 |
| TCGA-QQ-A8VF | 70 | MALE | WHITE | NA | NO | No | NA | 0 | 3406 | 0 | 3406 | NA | NA | 0 | 3406 | C1 | High_risk | 6.632171156 |
| TCGA-QQ-A8VG | 52 | MALE | WHITE | Primary | NO | No | NA | 1 | 1970 | 1 | 1970 | NA | NA | 1 | 1852 | C1 | High_risk | 6.19057931 |
| TCGA-QQ-A8VH | 31 | FEMALE | WHITE | Recurrence | YES | No | NA | 1 | 320 | 1 | 320 | NA | NA | 1 | 197 | C1 | High_risk | 6.232030853 |
| TCGA-RN-A68Q | 62 | FEMALE | WHITE | NA | NO | No | NA | 0 | 22 | 0 | 22 | NA | NA | 0 | 22 | C1 | High_risk | 6.274642711 |
| TCGA-RN-AAAQ | 52 | MALE | WHITE | Recurrence | YES | No | Chemotherapy | 0 | 510 | 0 | 510 | NA | NA | 1 | 307 | C1 | High_risk | 6.219003214 |
| TCGA-SG-A6Z4 | 47 | MALE | WHITE | Metastasis | YES | No | Chemotherapy | 0 | 576 | 0 | 576 | NA | NA | 1 | 105 | C1 | High_risk | 6.244888005 |
| TCGA-SG-A6Z7 | 44 | FEMALE | WHITE | NA | NO | No | NA | 1 | 118 | 0 | 118 | NA | NA | 0 | 118 | C1 | High_risk | 6.198234297 |
| TCGA-SG-A849 | 78 | MALE | WHITE | NA | YES | No | NA | 0 | 533 | 0 | 533 | NA | NA | 0 | 533 | C1 | Low_risk | 6.043669825 |
| TCGA-SI-A71O | 29 | MALE | WHITE | Metastasis | YES | No | NA | 1 | 694 | 1 | 694 | NA | NA | 1 | 264 | C1 | High_risk | 6.707273417 |
| TCGA-SI-A71O.1 | 29 | MALE | WHITE | Metastasis | YES | No | NA | 1 | 694 | 1 | 694 | NA | NA | 1 | 264 | C1 | Low_risk | 6.029537931 |
| TCGA-SI-A71P | 50 | MALE | WHITE | NA | YES | No | Chemotherapy | 0 | 771 | 0 | 771 | NA | NA | 0 | 771 | C1 | High_risk | 6.425344917 |
| TCGA-SI-A71Q | 34 | FEMALE | BLACK | NA | YES | No | NA | 0 | 996 | 0 | 996 | 0 | 996 | 0 | 996 | C1 | High_risk | 6.27706184 |
| TCGA-SI-AA8B | 80 | FEMALE | WHITE | NA | NO | No | NA | 1 | 262 | 1 | 262 | NA | NA | 1 | 262 | C1 | High_risk | 6.651317971 |
| TCGA-SI-AA8C | 20 | FEMALE | WHITE | Metastasis | YES | No | NA | 0 | 597 | 0 | 597 | NA | NA | 1 | 273 | C1 | High_risk | 6.232268161 |
| TCGA-UE-A6QT | 50 | FEMALE | ASIAN | NA | NO | No | NA | 0 | 402 | 0 | 402 | 0 | 402 | 0 | 402 | C1 | Low_risk | 5.990226208 |
| TCGA-UE-A6QU | 90 | FEMALE | ASIAN | NA | NO | No | NA | 0 | 408 | 0 | 408 | 0 | 408 | 0 | 408 | C1 | Low_risk | 5.934522487 |
| TCGA-VT-A80G | 66 | MALE | WHITE | Metastasis | YES | No | NA | 0 | 326 | 0 | 326 | 1 | 26 | 1 | 26 | C1 | Low_risk | 5.778473479 |
| TCGA-VT-A80J | 49 | FEMALE | WHITE | Metastasis | NO | No | NA | 0 | 796 | 0 | 796 | 1 | 355 | 1 | 355 | C1 | High_risk | 6.343875171 |
| TCGA-VT-AB3D | 71 | MALE | WHITE | NA | YES | No | NA | 0 | 379 | 0 | 379 | 0 | 379 | 0 | 379 | C2 | Low_risk | 5.824512595 |
| TCGA-WK-A8XO | 66 | FEMALE | WHITE | Recurrence | NO | No | NA | 1 | 363 | 1 | 363 | 1 | 105 | 1 | 105 | C1 | Low_risk | 5.962419848 |
| TCGA-WK-A8XQ | 57 | MALE | WHITE | Recurrence | NO | No | NA | 1 | 146 | 1 | 146 | NA | NA | 1 | 27 | C1 | High_risk | 6.323007032 |
| TCGA-WK-A8XS | 58 | FEMALE | WHITE | NA | NO | No | NA | 0 | 2579 | 0 | 2579 | 0 | 2579 | 0 | 2579 | C1 | Low_risk | 5.767086279 |
| TCGA-WK-A8XT | 40 | FEMALE | WHITE | Recurrence | NO | No | NA | 1 | 1262 | 1 | 1262 | 1 | 599 | 1 | 599 | C1 | Low_risk | 6.039250083 |
| TCGA-WK-A8XX | 70 | MALE | WHITE | Recurrence | NO | No | NA | 1 | 1164 | 1 | 1164 | 1 | 111 | 1 | 111 | C1 | Low_risk | 5.967052771 |
| TCGA-WK-A8XY | 53 | FEMALE | WHITE | Recurrence | NO | No | NA | 1 | 1991 | 1 | 1991 | NA | NA | 1 | 103 | C1 | High_risk | 6.273352447 |
| TCGA-WK-A8XZ | 56 | FEMALE | WHITE | Metastasis | YES | No | NA | 1 | 1722 | 1 | 1722 | 1 | 89 | 1 | 89 | C1 | Low_risk | 5.808051321 |
| TCGA-WK-A8Y0 | 49 | FEMALE | WHITE | Metastasis | YES | No | NA | 1 | 688 | 1 | 688 | 1 | 111 | 1 | 111 | C1 | High_risk | 6.665344941 |
| TCGA-WP-A9GB | 59 | FEMALE | WHITE | Metastasis | NO | No | NA | 1 | 261 | 1 | 261 | NA | NA | 1 | 214 | C1 | Low_risk | 6.018294576 |
| TCGA-X2-A95T | 49 | FEMALE | WHITE | NA | NO | No | Chemotherapy | 0 | 1927 | 0 | 1927 | 0 | 1927 | 0 | 1927 | C1 | High_risk | 6.450095011 |
| TCGA-X6-A7W8 | 89 | MALE | WHITE | Metastasis | YES | No | NA | 1 | 804 | 1 | 804 | NA | NA | 1 | 146 | C2 | High_risk | 6.358254219 |
| TCGA-X6-A7WA | 90 | FEMALE | WHITE | NA | NO | No | NA | 0 | 1092 | 0 | 1092 | NA | NA | 0 | 1092 | C1 | Low_risk | 6.079069134 |
| TCGA-X6-A7WB | 58 | FEMALE | WHITE | Metastasis | NO | No | NA | 1 | 550 | 1 | 550 | NA | NA | 1 | 104 | C1 | High_risk | 6.864351959 |
| TCGA-X6-A7WC | 74 | MALE | WHITE | Metastasis | YES | No | Chemotherapy | 0 | 1379 | 0 | 1379 | 1 | 301 | 1 | 301 | C1 | High_risk | 6.173099707 |
| TCGA-X6-A7WD | 63 | FEMALE | WHITE | Metastasis | NO | No | NA | 0 | 938 | 0 | 938 | 1 | 558 | 1 | 558 | C1 | Low_risk | 6.104651584 |
| TCGA-X6-A8C2 | 56 | MALE | WHITE | Primary | YES | No | Chemotherapy | 0 | 1070 | 0 | 1070 | NA | NA | 1 | 7 | C1 | Low_risk | 5.801334166 |
| TCGA-X6-A8C3 | 59 | FEMALE | ASIAN | NA | YES | No | NA | 0 | 583 | 0 | 583 | NA | NA | 0 | 583 | C1 | High_risk | 6.851672743 |
| TCGA-X6-A8C4 | 70 | FEMALE | WHITE | Metastasis | YES | No | NA | 1 | 628 | 1 | 628 | NA | NA | 1 | 71 | C1 | High_risk | 6.561691322 |
| TCGA-X6-A8C5 | 63 | FEMALE | BLACK | NA | NO | No | NA | 0 | 1547 | 0 | 1547 | 0 | 1547 | 0 | 1547 | C2 | Low_risk | 5.547882926 |
| TCGA-X6-A8C6 | 55 | MALE | WHITE | Metastasis | YES | No | Chemotherapy | 1 | 1067 | 1 | 1067 | NA | NA | 1 | 408 | C1 | High_risk | 6.238980345 |
| TCGA-X6-A8C7 | 24 | FEMALE | WHITE | NA | YES | No | NA | 0 | 896 | 0 | 896 | 0 | 896 | 0 | 896 | C1 | High_risk | 6.564422623 |
| TCGA-X9-A971 | 52 | FEMALE | WHITE | Metastasis | NO | No | Chemotherapy | 0 | 831 | 0 | 831 | 1 | 224 | 1 | 224 | C1 | High_risk | 6.201090616 |
| TCGA-X9-A973 | 55 | MALE | WHITE | Metastasis | NO | No | Chemotherapy | 0 | 1108 | 0 | 1108 | 1 | 119 | 1 | 119 | C1 | High_risk | 6.128161471 |
| TCGA-Z4-A8JB | 24 | FEMALE | WHITE | NA | NO | No | NA | 0 | 133 | 0 | 133 | 0 | 133 | 0 | 133 | C1 | Low_risk | 5.73764462 |
| TCGA-Z4-A9VC | 37 | MALE | WHITE | NA | NO | No | NA | 0 | 407 | 0 | 407 | 0 | 407 | 0 | 407 | C1 | Low_risk | 6.097203134 |
| TCGA-Z4-AAPF | 35 | FEMALE | WHITE | NA | NO | No | NA | 0 | 485 | 0 | 485 | 0 | 485 | 0 | 485 | C1 | Low_risk | 5.974959483 |
| TCGA-Z4-AAPG | 64 | FEMALE | WHITE | NA | NO | No | NA | 0 | 486 | 0 | 486 | 0 | 486 | 0 | 486 | C1 | Low_risk | 6.112681404 |
