# Supplementary material for: Integration of ubiquitination-related genes in predictive signatures for prognosis and immunotherapy response in sarcoma
Source: Front Oncol. 2024 Oct 14;14:1446522. doi: 10.3389/fonc.2024.1446522 (PMC11513255; doi:10.3389/fonc.2024.1446522)
Supplement: Supplementary file 1 [file DataSheet1.zip › Supplementary Table 7.docx]

**Supplementary Table 7. Correlation analysis between lncRNA and hsa-miR-29c-3p，hsa-miR-143-3p or lncRNA and CALR,CASP3 in SARC determined by ENCORI databases.**

| **LncRNA** | **miRNA** | **R value** | **p value** |
| --- | --- | --- | --- |
| LINC00943 | hsa-miR-29c-3p | -0.166 | 7.14E-03 |
| LINC00944 | hsa-miR-143-3p | -0.440 | 8.69E-14 |
| SMIM25 | hsa-miR-143-3p | -0.268 | 1.10E-05 |
| MIR503HG | hsa-miR-143-3p | -0.375 | 4.06E-10 |
| LINC01806 | hsa-miR-143-3p | -0.126 | 4.21E-02 |
| **LncRNA** | **mRNA** | **R value** | **p value** |
| LINC00943 | CALR | 0.242 | 7.43E-05 |
| LINC01806 | CASP3 | 0.169 | 5.99E-03 |
| LINC00944 | CASP3 | 0.154 | 1.27E-02 |
| SMIM25 | CASP3 | 0.167 | 6.64E-03 |
| MIR503HG | CASP3 | 0.167 | 6.79E-035 |
