# Supplementary material for: Integration of ubiquitination-related genes in predictive signatures for prognosis and immunotherapy response in sarcoma
Source: Front Oncol. 2024 Oct 14;14:1446522. doi: 10.3389/fonc.2024.1446522 (PMC11513255; doi:10.3389/fonc.2024.1446522)
Supplement: Supplementary file 1 [file DataSheet1.zip › Supplementary Table 8.docx]

**Supplementary Table 8. Primer sequences of genes**

| Real-time quantitative PCR primer sequence | |
| --- | --- |
| Gene | Sequence (5’- 3’ on minus strand) |
| *CALR* | Fwd: CCTGCCGTCTACTTCAAGGAG |
|  | Rev: GAACTTGCCGGAACTGAGAAC |
| *CASP3* | Fwd: CATGGAAGCGAATCAATGGACT |
|  | Rev: CTGTACCAGACCGAGATGTCA |
| *BCL10* | Fwd: GTGAAGAAGGACGCCTTAGAAA |
|  | Rev: TCAACAAGGGTGTCCAGACCT |
| *PSMD7* | Fwd: GCTGCCTACAGAAGCGTACAT |
|  | Rev: TCTCGTAACAAGTGTTCAACTCC |
| *PSMD10* | Fwd: GGGTGTGTGTCTAACCTAATGG |
|  | Rev: GGCCAGAATACTCTCCTTCAACT |
