# Supplementary material for: Integration of ubiquitination-related genes in predictive signatures for prognosis and immunotherapy response in sarcoma
Source: Front Oncol. 2024 Oct 14;14:1446522. doi: 10.3389/fonc.2024.1446522 (PMC11513255; doi:10.3389/fonc.2024.1446522)
Supplement: Supplementary file 1 [file DataSheet1.zip › Supplementary Table 1.docx]

**Supplementary Table 1 Differentially expressed genes between SARC and normal samples in the GSE17674 dataset**

| id | logFC | AveExpr | t | P.Value | adj.P.Val | B | Gene.Symbol |
| --- | --- | --- | --- | --- | --- | --- | --- |
| 209742_s_at | -9.573608877 | 7.078774533 | -20.8223123 | 7.27E-30 | 3.86E-28 | 57.61699973 | MYL2 |
| 204631_at | -9.557357248 | 6.784557854 | -20.7797486 | 8.13E-30 | 4.27E-28 | 57.50400395 | MYH2 |
| 209904_at | -9.102964828 | 7.105037732 | -21.32890363 | 1.93E-30 | 1.15E-28 | 58.9485069 | TNNC1 |
| 214087_s_at | -9.082406413 | 6.895865939 | -20.49990467 | 1.71E-29 | 8.37E-28 | 56.75668918 | MYBPC1 |
| 204810_s_at | -9.075305232 | 7.504450196 | -17.79983333 | 3.33E-26 | 9.22E-25 | 49.13177342 | CKM |
| 203872_at | -9.045498117 | 7.660825872 | -17.81141426 | 3.22E-26 | 8.95E-25 | 49.16616571 | ACTA1 |
| 219728_at | -8.569867372 | 6.592760648 | -21.10971835 | 3.42E-30 | 1.90E-28 | 58.37541562 | MYOT |
| 209888_s_at | -8.538255225 | 7.594701694 | -16.42043137 | 2.17E-24 | 4.56E-23 | 44.92428075 | MYL1 |
| 205054_at | -8.466270747 | 7.379976728 | -15.9471972 | 9.59E-24 | 1.80E-22 | 43.4287122 | NEB |
| 205577_at | -8.437529718 | 6.854276176 | -56.42053628 | 1.84E-55 | 4.42E-52 | 116.1253763 | PYGM |
| 205374_at | -8.402139085 | 7.652125106 | -20.95204926 | 5.17E-30 | 2.80E-28 | 57.96033706 | SLN |
| 205388_at | -8.290663801 | 7.823390396 | -17.22253554 | 1.86E-25 | 4.59E-24 | 47.39791335 | TNNC2 |
| 204179_at | -8.278234591 | 7.904171196 | -15.48816595 | 4.16E-23 | 7.13E-22 | 41.95184142 | MB |
| 208195_at | -8.207766788 | 8.058223728 | -13.58372192 | 2.42E-20 | 2.74E-19 | 35.54181163 | TTN |
| 228794_at | -8.104919436 | 6.309484223 | -20.2933203 | 2.97E-29 | 1.37E-27 | 56.20006325 | XIRP2 |
| 204865_at | -7.978873348 | 5.944750358 | -22.90108622 | 3.69E-32 | 2.77E-30 | 62.92978952 | CA3 |
| 205444_at | -7.957974239 | 6.395425681 | -42.25057109 | 8.69E-48 | 6.33E-45 | 98.8402158 | ATP2A1 |
| 206121_at | -7.860521908 | 6.193046366 | -32.72976101 | 3.92E-41 | 1.16E-38 | 83.62905256 | AMPD1 |
| 205951_at | -7.844524011 | 6.130266612 | -14.1539792 | 3.42E-21 | 4.39E-20 | 37.50956252 | MYH1 |
| 219772_s_at | -7.810049085 | 6.128521386 | -23.29005657 | 1.43E-32 | 1.13E-30 | 63.88113371 | SMPX |
| 222827_s_at | -7.779266474 | 7.057639582 | -28.59199179 | 1.11E-37 | 2.11E-35 | 75.68338912 | KLHL41 |
| 205163_at | -7.773940773 | 8.111227104 | -24.38532402 | 1.06E-33 | 1.01E-31 | 66.4922146 | MYLPF |
| 216265_x_at | -7.773477033 | 6.750887524 | -61.62108802 | 8.03E-58 | 2.90E-54 | 121.3566337 | MYH7 |
| 213201_s_at | -7.707757051 | 8.212083309 | -20.41556433 | 2.14E-29 | 1.02E-27 | 56.5299516 | TNNT1 |
| 203296_s_at | -7.672873232 | 6.535164465 | -31.53016541 | 3.57E-40 | 9.91E-38 | 81.42377207 | ATP1A2 |
| 239057_at | -7.646831034 | 6.202502165 | -28.83070455 | 6.84E-38 | 1.33E-35 | 76.16897898 | LMOD2 |
| 205177_at | -7.62365383 | 6.663835052 | -25.0283345 | 2.41E-34 | 2.65E-32 | 67.98037962 | TNNI1 |
| 235313_at | -7.599118587 | 6.166199651 | -36.04671043 | 1.24E-43 | 4.90E-41 | 89.3579692 | NRAP |
| 219645_at | -7.592953603 | 5.788566758 | -68.56122002 | 1.10E-60 | 1.19E-56 | 127.6393788 | CASQ1 |
| 233520_s_at | -7.565242799 | 7.839654912 | -24.61718151 | 6.21E-34 | 6.25E-32 | 67.03254147 | CMYA5 |
| 235981_at | -7.486151881 | 5.726411351 | -36.63041212 | 4.75E-44 | 2.06E-41 | 90.31449619 | C8orf22 |
| 204483_at | -7.484240786 | 7.617421139 | -36.9186796 | 2.97E-44 | 1.34E-41 | 90.78155513 | ENO3 |
| 205295_at | -7.456217544 | 6.166521191 | -38.02134753 | 5.07E-45 | 2.74E-42 | 92.5365566 | CKMT2 |
| 205553_s_at | -7.32278745 | 6.407079626 | -17.43101483 | 9.96E-26 | 2.56E-24 | 48.02848363 | CSRP3 |
| 207148_x_at | -7.314759429 | 5.297929591 | -37.50405223 | 1.15E-44 | 5.44E-42 | 91.7193826 | MYOZ2 |
| 206393_at | -7.270732812 | 7.314417878 | -19.73209497 | 1.36E-28 | 5.63E-27 | 54.66625649 | TNNI2 |
| 205589_at | -7.203963788 | 6.844234328 | -65.05794516 | 2.81E-59 | 1.52E-55 | 124.559556 | MYL3 |
| 226325_at | -7.180009474 | 6.291507633 | -34.53178963 | 1.62E-42 | 5.56E-40 | 86.80542239 | ADSSL1 |
| 222919_at | -7.048384664 | 6.007754889 | -24.54893581 | 7.27E-34 | 7.19E-32 | 66.87393982 | TRDN |
| 206394_at | -7.024394549 | 6.437148329 | -17.17964162 | 2.12E-25 | 5.20E-24 | 47.2675497 | MYBPC2 |
| 1552732_at | -6.958203099 | 4.828516983 | -55.36804024 | 5.85E-55 | 1.15E-51 | 115.0044866 | ABRA |
| 235503_at | -6.892637735 | 5.336905482 | -20.7536592 | 8.71E-30 | 4.57E-28 | 57.43465628 | ASB5 |
| 235004_at | -6.853860898 | 5.72900106 | -26.51480405 | 8.81E-36 | 1.25E-33 | 71.30126364 | RBM24 |
| 1569986_x_at | -6.831006877 | 7.532140765 | -26.06800777 | 2.34E-35 | 3.06E-33 | 70.32006305 | TNNT3 |
| 235465_at | 6.798299397 | 8.414563563 | 15.99590452 | 8.22E-24 | 1.56E-22 | 43.5839006 | AMER2 |
| 205736_at | -6.771716087 | 5.655520154 | -62.2792239 | 4.17E-58 | 1.80E-54 | 121.9847325 | PGAM2 |
| 206915_at | 6.744780076 | 7.684119117 | 19.48132872 | 2.72E-28 | 1.06E-26 | 53.97051411 | NKX2-2 |
| 219509_at | -6.69129499 | 7.990365866 | -29.70655141 | 1.19E-38 | 2.71E-36 | 77.92059843 | MYOZ1 |
| 206353_at | -6.549414553 | 6.412052862 | -41.03644457 | 5.08E-47 | 3.33E-44 | 97.09590697 | COX6A2 |
| 227532_at | -6.535539629 | 6.002560899 | -42.37822722 | 7.24E-48 | 5.60E-45 | 99.02074347 | LRRC39 |
| 227834_at | -6.533552453 | 4.852329514 | -49.86835073 | 3.58E-52 | 5.16E-49 | 108.7606308 | TXLNB |
| 243346_at | -6.515302068 | 4.99112626 | -34.62577274 | 1.38E-42 | 4.80E-40 | 86.96681257 | LMOD3 |
| 209621_s_at | -6.50117787 | 8.286322668 | -22.90493797 | 3.65E-32 | 2.76E-30 | 62.93927364 | PDLIM3 |
| 219926_at | -6.417939896 | 5.603220353 | -38.43867074 | 2.63E-45 | 1.50E-42 | 93.18806064 | POPDC3 |
| 204939_s_at | -6.389540437 | 7.351839532 | -20.83526091 | 7.02E-30 | 3.75E-28 | 57.65134015 | PLN |
| 236523_at | -6.368198107 | 5.302453334 | -28.508129 | 1.32E-37 | 2.48E-35 | 75.51194533 | LOC285556 |
| 205766_at | -6.363286636 | 7.262377778 | -47.59733646 | 6.18E-51 | 7.04E-48 | 105.9726066 | TCAP |
| 231051_at | -6.320330455 | 5.369041895 | -53.74946844 | 3.62E-54 | 6.03E-51 | 113.2363486 | LINC00948 |
| 236972_at | -6.269872104 | 5.754479621 | -23.21931992 | 1.70E-32 | 1.32E-30 | 63.7090843 | TRIM63 |
| 210809_s_at | 6.239532909 | 8.55232577 | 13.29741012 | 6.55E-20 | 6.98E-19 | 34.53816797 | POSTN |
| 226103_at | -6.190556952 | 6.740009381 | -22.3526478 | 1.43E-31 | 9.72E-30 | 61.56617039 | NEXN |
| 207173_x_at | 6.185431103 | 8.330640188 | 21.02054716 | 4.32E-30 | 2.37E-28 | 58.14095674 | CDH11 |
| 226228_at | -6.130370591 | 5.051311468 | -15.76033358 | 1.74E-23 | 3.15E-22 | 42.83063947 | AQP4 |
| 208712_at | 6.111300122 | 8.658606709 | 24.04508141 | 2.36E-33 | 2.08E-31 | 65.69156232 | CCND1 |
| 235617_x_at | -6.048275702 | 5.000161221 | -44.46804629 | 3.89E-49 | 3.25E-46 | 101.9019376 | LOC100507537 |
| 222895_s_at | 6.040141388 | 8.790266583 | 18.5708928 | 3.54E-27 | 1.16E-25 | 51.38883619 | BCL11B |
| 206160_at | -5.993092855 | 7.074203792 | -29.86016963 | 8.78E-39 | 2.04E-36 | 78.22306446 | APOBEC2 |
| 235261_at | -5.975517792 | 5.32420981 | -28.94891501 | 5.39E-38 | 1.08E-35 | 76.40812755 | UNC45B |
| 212730_at | -5.973578414 | 7.776782315 | -24.49438508 | 8.25E-34 | 7.94E-32 | 66.74690159 | SYNM |
| 206116_s_at | -5.95865185 | 8.672054042 | -20.6378846 | 1.18E-29 | 6.09E-28 | 57.12611832 | TPM1 |
| 206895_at | -5.951409608 | 4.840457089 | -55.67500653 | 4.16E-55 | 9.01E-52 | 115.3337007 | PPP1R3A |
| 230653_at | 5.940549514 | 8.342474815 | 23.84724205 | 3.77E-33 | 3.21E-31 | 65.22171527 | LOC102724356 |
| 241749_at | -5.918139496 | 5.197081354 | -29.07898956 | 4.14E-38 | 8.44E-36 | 76.6702799 | MURC |
| 239398_at | -5.851443706 | 5.473185396 | -41.60294603 | 2.22E-47 | 1.55E-44 | 97.91600753 | KLHL31 |
| 204284_at | -5.850781997 | 8.046394279 | -22.1711544 | 2.26E-31 | 1.49E-29 | 61.10905711 | PPP1R3C |
| 205440_s_at | 5.845954882 | 7.642078299 | 11.8905731 | 1.02E-17 | 8.02E-17 | 29.4558335 | NPY1R |
| 1559867_at | -5.723516199 | 5.306590133 | -53.4985712 | 4.82E-54 | 7.46E-51 | 112.9572941 | AK056982 |
| 201291_s_at | 5.70286965 | 7.534160847 | 22.62613073 | 7.25E-32 | 5.13E-30 | 62.24944391 | TOP2A |
| 228621_at | -5.699183899 | 5.498503056 | -34.27902852 | 2.51E-42 | 8.22E-40 | 86.36932656 | HFE2 |
| 205610_at | -5.639691366 | 6.499434903 | -36.28957451 | 8.32E-44 | 3.53E-41 | 89.75772994 | MYOM1 |
| 223572_at | -5.625848373 | 5.803634333 | -37.82567876 | 6.91E-45 | 3.56E-42 | 92.22871857 | HHATL |
| 225864_at | 5.617260575 | 8.343172163 | 16.66889444 | 1.01E-24 | 2.23E-23 | 45.6986837 | FAM84B |
| 202222_s_at | -5.616067689 | 7.484061162 | -15.18991539 | 1.09E-22 | 1.75E-21 | 40.97825346 | DES |
| 206844_at | -5.599311395 | 5.448736856 | -66.23915236 | 9.24E-60 | 6.67E-56 | 125.6179642 | FBP2 |
| 235944_at | 5.589582792 | 8.210289251 | 15.62626696 | 2.67E-23 | 4.69E-22 | 42.39889918 | HMCN1 |
| 205542_at | 5.548682233 | 8.200780547 | 14.20613026 | 2.87E-21 | 3.70E-20 | 37.68744212 | STEAP1 |
| 227915_at | -5.545039841 | 6.503466753 | -54.1458462 | 2.30E-54 | 4.16E-51 | 113.6744534 | ASB2 |
| 227848_at | -5.538452353 | 5.988384568 | -23.86286571 | 3.63E-33 | 3.13E-31 | 65.25893577 | PEBP4 |
| 226304_at | -5.531488069 | 5.977430044 | -26.54481045 | 8.26E-36 | 1.19E-33 | 71.36665172 | HSPB6 |
| 206372_at | -5.522336894 | 5.842540837 | -26.32926574 | 1.32E-35 | 1.83E-33 | 70.8955358 | MYF6 |
| 205485_at | -5.467292855 | 6.993159982 | -18.61222296 | 3.14E-27 | 1.03E-25 | 51.50796362 | RYR1 |
| 209685_s_at | 5.434689029 | 8.187789387 | 14.09235308 | 4.22E-21 | 5.34E-20 | 37.29891715 | PRKCB |
| 207302_at | -5.432484764 | 5.529706764 | -32.16775275 | 1.09E-40 | 3.16E-38 | 82.60527557 | SGCG |
| 218934_s_at | -5.428031825 | 5.910209668 | -19.55987781 | 2.19E-28 | 8.69E-27 | 54.18914638 | HSPB7 |
| 201565_s_at | 5.371924613 | 9.237488254 | 24.22405504 | 1.55E-33 | 1.43E-31 | 66.11387598 | ID2 |
| 228057_at | -5.34174194 | 5.912779035 | -21.11060026 | 3.41E-30 | 1.90E-28 | 58.37773065 | DDIT4L |
| 222954_at | -5.337767891 | 5.548946449 | -56.64928209 | 1.43E-55 | 3.87E-52 | 116.3660713 | FBXO40 |
| 229309_at | 5.300728773 | 6.84489801 | 11.51856499 | 4.05E-17 | 2.92E-16 | 28.07108508 | ADRB1 |
| 206935_at | 5.231458661 | 6.532034414 | 9.717722292 | 3.97E-14 | 1.96E-13 | 21.15128658 | PCDH8 |
| 206768_at | -5.184213754 | 6.221482944 | -48.07565945 | 3.35E-51 | 4.03E-48 | 106.5709211 | RPL3L |
| 235377_at | -5.179981186 | 5.406705179 | -21.13943224 | 3.16E-30 | 1.78E-28 | 58.45337408 | MLIP |
| 201666_at | 5.15745969 | 9.844335317 | 17.06542964 | 3.00E-25 | 7.19E-24 | 46.91938818 | TIMP1 |
| 238867_at | -5.155639181 | 5.22494445 | -46.61870277 | 2.19E-50 | 2.26E-47 | 104.7292641 | TMEM182 |
| 226085_at | 5.147648577 | 8.705349855 | 35.08290788 | 6.29E-43 | 2.31E-40 | 87.74600458 | CBX5 |
| 206002_at | 5.143489252 | 7.6337505 | 8.003472496 | 3.63E-11 | 1.25E-10 | 14.32307899 | GPR64 |
| 243313_at | -5.134596531 | 6.496416516 | -27.33565567 | 1.51E-36 | 2.36E-34 | 73.06747416 | SYNPO2L |
| 205738_s_at | -5.130102317 | 6.501097179 | -34.49821614 | 1.71E-42 | 5.80E-40 | 86.74766942 | FABP3 |
| 227401_at | -5.126829246 | 5.715747586 | -29.23660343 | 3.02E-38 | 6.41E-36 | 76.98654363 | IL17D |
| 243737_at | -5.112581257 | 4.723400956 | -45.64544964 | 7.93E-50 | 7.81E-47 | 103.4663651 | ATP1B4 |
| 235874_at | 5.104624217 | 6.797387952 | 10.5091386 | 1.84E-15 | 1.07E-14 | 24.23352199 | PRSS35 |
| 1556499_s_at | 5.096006185 | 10.4888411 | 17.68125859 | 4.73E-26 | 1.29E-24 | 48.77876158 | COL1A1 |
| 225792_at | 5.091102018 | 7.00938554 | 12.12782493 | 4.28E-18 | 3.55E-17 | 30.33023416 | HOOK1 |
| 204170_s_at | 5.082755512 | 7.490629304 | 26.29056143 | 1.44E-35 | 1.96E-33 | 70.81059039 | CKS2 |
| 202589_at | 5.080709708 | 8.122257372 | 23.11758483 | 2.17E-32 | 1.68E-30 | 63.46089486 | TYMS |
| 206025_s_at | 5.070477036 | 7.843693194 | 12.39735928 | 1.61E-18 | 1.41E-17 | 31.31520223 | TNFAIP6 |
| 201952_at | 5.060989065 | 7.620059616 | 17.86359264 | 2.76E-26 | 7.76E-25 | 49.32093337 | ALCAM |
| 235183_at | -5.024900077 | 5.504210704 | -23.68897169 | 5.49E-33 | 4.52E-31 | 64.84353637 | FILIP1 |
| 203358_s_at | 5.023646267 | 7.353527811 | 19.7461219 | 1.31E-28 | 5.44E-27 | 54.70498201 | EZH2 |
| 202825_at | -5.00763304 | 8.6473724 | -45.12287948 | 1.60E-49 | 1.44E-46 | 102.7770664 | SLC25A4 |
| 219908_at | 4.994304093 | 7.260386872 | 10.00073726 | 1.31E-14 | 6.88E-14 | 22.26016556 | DKK2 |
| 224940_s_at | 4.988240363 | 7.658689206 | 12.38029992 | 1.71E-18 | 1.50E-17 | 31.25312914 | PAPPA |
| 204540_at | -4.972883174 | 7.728492058 | -14.04172285 | 5.01E-21 | 6.31E-20 | 37.12549399 | EEF1A2 |
| 210944_s_at | -4.961866259 | 7.414045164 | -27.94166209 | 4.24E-37 | 7.40E-35 | 74.34213619 | CAPN3 |
| 200953_s_at | 4.958621751 | 7.904547684 | 20.0582057 | 5.61E-29 | 2.47E-27 | 55.56138318 | CCND2 |
| 209283_at | -4.918284103 | 9.172812828 | -14.48609178 | 1.12E-21 | 1.55E-20 | 38.63642188 | CRYAB |
| 210038_at | -4.896651469 | 5.756446704 | -28.68325981 | 9.22E-38 | 1.78E-35 | 75.86946815 | PRKCQ |
| 243792_x_at | 4.895597039 | 7.227318963 | 15.03673032 | 1.80E-22 | 2.78E-21 | 40.47388068 | PTPN13 |
| 204979_s_at | -4.888858869 | 7.029289635 | -27.15118925 | 2.24E-36 | 3.42E-34 | 72.67459224 | SH3BGR |
| 212551_at | -4.877280804 | 7.4830425 | -19.3123091 | 4.36E-28 | 1.66E-26 | 53.49788466 | CAP2 |
| 212361_s_at | -4.871128411 | 9.825168512 | -30.67638695 | 1.80E-39 | 4.53E-37 | 79.80709485 | ATP2A2 |
| 206375_s_at | -4.870426068 | 5.693870881 | -21.82933164 | 5.35E-31 | 3.43E-29 | 60.24007036 | HSPB3 |
| 241961_at | -4.866844819 | 3.840250347 | -24.58315482 | 6.71E-34 | 6.67E-32 | 66.95350983 | TECRL |
| 228347_at | -4.86070562 | 6.937997658 | -15.13659161 | 1.30E-22 | 2.06E-21 | 40.80301527 | SIX1 |
| 202289_s_at | -4.858484328 | 6.714909024 | -18.52155824 | 4.08E-27 | 1.32E-25 | 51.24639318 | TACC2 |
| 231781_s_at | -4.851538326 | 5.903490494 | -32.78154831 | 3.57E-41 | 1.07E-38 | 83.72257445 | LRRC2 |
| 205391_x_at | -4.845988931 | 6.612324832 | -25.26340969 | 1.41E-34 | 1.64E-32 | 68.51646672 | ANK1 |
| 1552491_at | -4.838309217 | 5.957493958 | -37.53006296 | 1.11E-44 | 5.33E-42 | 91.76072906 | IDI2 |
| 202503_s_at | 4.832827578 | 7.857231178 | 16.24967578 | 3.70E-24 | 7.47E-23 | 44.38777204 | KIAA0101 |
| 232252_at | -4.830384442 | 5.429224855 | -25.00161619 | 2.57E-34 | 2.79E-32 | 67.91918196 | DUSP27 |
| 209875_s_at | 4.823136485 | 7.314042234 | 9.291567808 | 2.12E-13 | 9.60E-13 | 19.46929876 | SPP1 |
| 206645_s_at | 4.785674396 | 7.439733993 | 9.542215727 | 7.90E-14 | 3.77E-13 | 20.46026252 | NR0B1 |
| 232549_at | 4.782505883 | 6.84473725 | 10.38354648 | 2.98E-15 | 1.70E-14 | 23.74838944 | RBM11 |
| 203881_s_at | -4.76698395 | 5.800788151 | -14.87417705 | 3.08E-22 | 4.60E-21 | 39.93543472 | DMD |
| 207317_s_at | -4.76645022 | 6.763798676 | -17.634296 | 5.44E-26 | 1.47E-24 | 48.63850548 | CASQ2 |
| 204914_s_at | 4.76205939 | 7.295046109 | 10.69358472 | 9.09E-16 | 5.47E-15 | 24.94305485 | SOX11 |
| 220948_s_at | 4.751400866 | 9.842414122 | 16.99296185 | 3.73E-25 | 8.83E-24 | 46.69768664 | ATP1A1 |
| 204612_at | -4.743973637 | 7.448874242 | -11.33612167 | 8.00E-17 | 5.52E-16 | 27.38597827 | PKIA |
| 206891_at | -4.736276059 | 5.553882679 | -13.88716896 | 8.49E-21 | 1.03E-19 | 36.59407522 | ACTN3 |
| 227892_at | -4.728177063 | 5.459794601 | -18.02934676 | 1.70E-26 | 4.98E-25 | 49.81054338 | PRKAA2 |
| 1556638_at | 4.719197978 | 8.562662727 | 12.42610694 | 1.45E-18 | 1.28E-17 | 31.4197228 | PAX7 |
| 241793_at | -4.712236795 | 6.035756941 | -34.68947335 | 1.23E-42 | 4.38E-40 | 87.0759677 | MSS51 |
| 235173_at | -4.708393605 | 6.668484543 | -45.31168109 | 1.24E-49 | 1.17E-46 | 103.027025 | MBNL1-AS1 |
| 214850_at | 4.675613486 | 7.625709283 | 20.60040891 | 1.31E-29 | 6.63E-28 | 57.02596537 | SMA4 |
| 203139_at | 4.670921254 | 8.263653051 | 15.17478114 | 1.15E-22 | 1.84E-21 | 40.92855392 | DAPK1 |
| 230367_at | -4.663851035 | 6.119324809 | -31.19073947 | 6.76E-40 | 1.81E-37 | 80.78586658 | SMTNL1 |
| 225207_at | -4.653732268 | 7.430208514 | -11.4312942 | 5.60E-17 | 3.96E-16 | 27.74385264 | PDK4 |
| 225275_at | 4.652641535 | 7.012497473 | 11.43279998 | 5.57E-17 | 3.94E-16 | 27.74950633 | EDIL3 |
| 201540_at | -4.646635842 | 10.15397396 | -13.56509732 | 2.58E-20 | 2.91E-19 | 35.47684334 | FHL1 |
| 226208_at | 4.626004666 | 8.291530391 | 24.67915201 | 5.38E-34 | 5.55E-32 | 67.17624351 | ZSWIM6 |
| 203861_s_at | -4.624906312 | 9.106002588 | -12.41500653 | 1.51E-18 | 1.33E-17 | 31.37937623 | ACTN2 |
| 211709_s_at | 4.617287755 | 7.71608776 | 13.97740063 | 6.24E-21 | 7.75E-20 | 36.90469928 | CLEC11A |
| 219963_at | -4.613183763 | 6.056894971 | -41.5160828 | 2.51E-47 | 1.70E-44 | 97.79097288 | DUSP13 |
| 209291_at | 4.607812535 | 7.108123551 | 11.46585234 | 4.92E-17 | 3.52E-16 | 27.87353936 | ID4 |
| 210147_at | -4.596151153 | 6.548596921 | -16.70025129 | 9.14E-25 | 2.05E-23 | 45.79589165 | ART3 |
| 213717_at | -4.589818104 | 6.906247073 | -39.07742749 | 9.74E-46 | 6.03E-43 | 94.17217279 | LDB3 |
| 205498_at | -4.583908003 | 5.331670312 | -17.47153516 | 8.83E-26 | 2.29E-24 | 48.15045899 | GHR |
| 204165_at | 4.572387408 | 7.857094008 | 16.07707699 | 6.36E-24 | 1.23E-22 | 43.84188349 | WASF1 |
| 230915_at | -4.566638023 | 5.201768821 | -43.47337249 | 1.54E-48 | 1.23E-45 | 100.5477463 | DHRS7C |
| 217515_s_at | -4.558533644 | 5.478874348 | -34.00315275 | 4.06E-42 | 1.29E-39 | 85.88990821 | CACNA1S |
| 1552301_a_at | -4.551412753 | 6.06503876 | -25.07076464 | 2.19E-34 | 2.43E-32 | 68.0774526 | CORO6 |
| 205384_at | -4.542499693 | 6.962653935 | -24.19882852 | 1.64E-33 | 1.50E-31 | 66.05450636 | FXYD1 |
| 226785_at | 4.532860148 | 7.601408333 | 24.74939213 | 4.57E-34 | 4.79E-32 | 67.33875926 | ATP11C |
| 218883_s_at | 4.531053565 | 7.163090297 | 19.27892436 | 4.78E-28 | 1.81E-26 | 53.40417669 | CENPU |
| 229400_at | 4.530156827 | 7.455225436 | 12.68348664 | 5.75E-19 | 5.36E-18 | 32.35088959 | HOXD10 |
| 226290_at | 4.523208554 | 8.607760315 | 11.30118763 | 9.12E-17 | 6.24E-16 | 27.25435358 | BDP1 |
| 209199_s_at | -4.521654835 | 8.33666025 | -14.55464091 | 8.89E-22 | 1.25E-20 | 38.86726098 | MEF2C |
| 222431_at | 4.521168934 | 8.44486141 | 28.10418275 | 3.03E-37 | 5.42E-35 | 74.67987117 | SPIN1 |
| 202565_s_at | -4.518864454 | 8.38568454 | -18.07727125 | 1.48E-26 | 4.38E-25 | 49.95152913 | SVIL |
| 243835_at | 4.503815591 | 7.663538735 | 12.20193545 | 3.27E-18 | 2.75E-17 | 30.60195548 | ZDHHC21 |
| 229313_at | -4.496368661 | 6.09452376 | -23.3832453 | 1.14E-32 | 9.13E-31 | 64.10714769 | ANO5 |
| 227558_at | 4.486403041 | 7.466331937 | 24.61358625 | 6.26E-34 | 6.27E-32 | 67.02419529 | CBX4 |
| 219825_at | 4.476710484 | 6.938747185 | 8.4731748 | 5.51E-12 | 2.10E-11 | 16.20696886 | CYP26B1 |
| 206584_at | 4.464987403 | 7.020466295 | 15.0781782 | 1.57E-22 | 2.45E-21 | 40.61064153 | LY96 |
| 214468_at | -4.453639837 | 5.151562038 | -46.64684563 | 2.11E-50 | 2.26E-47 | 104.7653861 | MYH6 |
| 209242_at | 4.447626816 | 6.955771456 | 7.320456979 | 5.64E-10 | 1.68E-09 | 11.58317076 | PEG3 |
| 203856_at | 4.442702101 | 7.339045654 | 21.72658412 | 6.96E-31 | 4.35E-29 | 59.97678064 | VRK1 |
| 222077_s_at | 4.434300351 | 7.071909439 | 24.76027351 | 4.46E-34 | 4.69E-32 | 67.36390134 | RACGAP1 |
| 1554062_at | 4.432138721 | 6.931747505 | 9.475095852 | 1.03E-13 | 4.83E-13 | 20.19535143 | XG |
| 1554579_a_at | -4.424954599 | 5.023324171 | -29.6199144 | 1.41E-38 | 3.15E-36 | 77.74939727 | MYO18B |
| 45288_at | 4.418825401 | 7.602644038 | 13.82139571 | 1.06E-20 | 1.27E-19 | 36.36699342 | ABHD6 |
| 232456_at | -4.417312681 | 5.631150332 | -26.79582288 | 4.80E-36 | 7.07E-34 | 71.9111693 | C10orf71 |
| 211275_s_at | -4.415292422 | 8.406106432 | -14.71851015 | 5.15E-22 | 7.46E-21 | 39.41667292 | GYG1 |
| 235042_at | -4.409862328 | 6.707655252 | -19.68326853 | 1.56E-28 | 6.41E-27 | 54.5312987 | XIRP1 |
| 212651_at | -4.373065605 | 6.108842952 | -17.37120831 | 1.19E-25 | 3.01E-24 | 47.84810643 | RHOBTB1 |
| 209598_at | 4.365823375 | 6.953266256 | 12.07912953 | 5.12E-18 | 4.20E-17 | 30.15132744 | PNMA2 |
| 228367_at | -4.361988816 | 5.080401109 | -30.19146944 | 4.59E-39 | 1.11E-36 | 78.87065715 | ALPK2 |
| 204187_at | -4.348523455 | 5.378159441 | -33.81104191 | 5.68E-42 | 1.78E-39 | 85.55391022 | GMPR |
| 205066_s_at | 4.345072157 | 7.91443701 | 8.878801177 | 1.09E-12 | 4.51E-12 | 17.82826394 | ENPP1 |
| 230195_at | -4.343977828 | 6.155069863 | -10.53197745 | 1.69E-15 | 9.86E-15 | 24.32157064 | LINC01405 |
| 216442_x_at | 4.34298803 | 10.94150726 | 17.50481884 | 7.99E-26 | 2.09E-24 | 48.25050902 | FN1 |
| 204457_s_at | 4.341146624 | 9.413959616 | 12.27742895 | 2.48E-18 | 2.12E-17 | 30.87804941 | GAS1 |
| 235718_at | -4.33707516 | 6.845226389 | -42.24386899 | 8.77E-48 | 6.33E-45 | 98.83072304 | SRL |
| 1559429_a_at | -4.336567655 | 4.498139597 | -59.42657248 | 7.51E-57 | 2.32E-53 | 119.2088853 | C3orf43 |
| 213089_at | 4.335260647 | 8.565716734 | 17.10920392 | 2.62E-25 | 6.34E-24 | 47.05300907 | LOC100272216 |
| 203560_at | 4.331129781 | 7.022457032 | 20.82914302 | 7.14E-30 | 3.80E-28 | 57.6351172 | GGH |
| 231179_at | -4.307975475 | 4.960150721 | -20.60771422 | 1.28E-29 | 6.53E-28 | 57.04549945 | IP6K3 |
| 201563_at | 4.306547746 | 7.636197405 | 13.99556362 | 5.86E-21 | 7.31E-20 | 36.96709979 | SORD |
| 214451_at | 4.297915328 | 6.957209934 | 6.462812574 | 1.74E-08 | 4.39E-08 | 8.174500144 | TFAP2B |
| 229578_at | -4.288733392 | 5.681240428 | -37.8593929 | 6.55E-45 | 3.46E-42 | 92.28186853 | JPH2 |
| 205334_at | -4.282433345 | 6.072169903 | -25.85398127 | 3.76E-35 | 4.69E-33 | 69.84495379 | S100A1 |
| 229730_at | -4.254239617 | 5.702550453 | -22.34854784 | 1.45E-31 | 9.79E-30 | 61.55587663 | SMTNL2 |
| 224663_s_at | -4.248246848 | 8.224445566 | -18.84777243 | 1.61E-27 | 5.62E-26 | 52.18335518 | CFL2 |
| 230467_at | -4.237683021 | 5.005877496 | -38.79785876 | 1.50E-45 | 9.03E-43 | 93.74337537 | TMEM52 |
| 218970_s_at | -4.229527118 | 7.236774219 | -29.25592888 | 2.91E-38 | 6.23E-36 | 77.02521719 | CUTC |
| 221667_s_at | -4.222870911 | 8.343947194 | -13.29881671 | 6.51E-20 | 6.95E-19 | 34.54312427 | HSPB8 |
| 210967_x_at | -4.206181261 | 5.842794743 | -27.37348845 | 1.40E-36 | 2.21E-34 | 73.14776721 | CACNB1 |
| 243332_at | 4.193513632 | 6.890421697 | 16.03758373 | 7.21E-24 | 1.38E-22 | 43.7164662 | RP11-124L9.5 |
| 1554803_s_at | -4.19031555 | 5.956411081 | -36.18352512 | 9.92E-44 | 4.13E-41 | 89.58348277 | TRIM72 |
| 224505_s_at | -4.189970095 | 5.017486252 | -48.36634998 | 2.32E-51 | 2.96E-48 | 106.9315871 | PLCD4 |
| 1558645_at | -4.18834143 | 4.416149456 | -23.33265544 | 1.29E-32 | 1.03E-30 | 63.98454101 | MIR133A1HG |
| 217979_at | 4.178298815 | 9.25801468 | 9.958385653 | 1.55E-14 | 8.03E-14 | 22.09467552 | TSPAN13 |
| 201745_at | 4.173030552 | 6.987295856 | 35.76910245 | 1.98E-43 | 7.51E-41 | 88.8978825 | TWF1 |
| 201417_at | 4.169321866 | 7.911741675 | 17.82874491 | 3.06E-26 | 8.52E-25 | 49.21760479 | SOX4 |
| 203755_at | 4.167430452 | 6.604642334 | 16.4908285 | 1.75E-24 | 3.72E-23 | 45.14444364 | BUB1B |
| 228783_at | -4.163749806 | 5.463019903 | -36.09735055 | 1.14E-43 | 4.59E-41 | 89.44153442 | BVES |
| 222803_at | 4.16001448 | 6.425080782 | 17.28326048 | 1.55E-25 | 3.88E-24 | 47.58210317 | PRTFDC1 |
| 226096_at | -4.153904607 | 6.479020505 | -13.21665112 | 8.69E-20 | 9.09E-19 | 34.25317987 | FNDC5 |
| 232892_at | -4.148831029 | 8.038171931 | -48.9750634 | 1.08E-51 | 1.46E-48 | 107.6797355 | C20orf166 |
| 228218_at | 4.147088467 | 6.580229388 | 10.83494937 | 5.30E-16 | 3.28E-15 | 25.48442778 | LSAMP |
| 1555044_a_at | -4.139044747 | 5.083623047 | -24.63886416 | 5.90E-34 | 6.00E-32 | 67.08285507 | KLHL40 |
| 226834_at | 4.136064119 | 6.509370741 | 13.21399645 | 8.77E-20 | 9.16E-19 | 34.24379778 | CLMP |
| 218009_s_at | 4.131265292 | 6.405075909 | 18.77388979 | 1.98E-27 | 6.84E-26 | 51.97215764 | PRC1 |
| 203335_at | -4.129783104 | 8.548493392 | -14.92472394 | 2.60E-22 | 3.94E-21 | 40.10322442 | PHYH |
| 219117_s_at | 4.126120002 | 7.088708746 | 21.52676918 | 1.16E-30 | 7.00E-29 | 59.46196565 | FKBP11 |
| 218585_s_at | 4.124884411 | 6.166364155 | 14.76894588 | 4.36E-22 | 6.39E-21 | 39.58508622 | DTL |
| 1553873_at | -4.116361341 | 4.114652604 | -25.22033386 | 1.56E-34 | 1.79E-32 | 68.41854681 | KLHL34 |
| 213943_at | 4.10641035 | 5.749312122 | 8.729678221 | 1.98E-12 | 7.92E-12 | 17.23305482 | TWIST1 |
| 223557_s_at | 4.101185686 | 6.536170618 | 6.389313987 | 2.33E-08 | 5.79E-08 | 7.885647476 | TMEFF2 |
| 219195_at | -4.101112995 | 4.724372983 | -24.41292332 | 9.97E-34 | 9.55E-32 | 66.55675541 | PPARGC1A |
| 213131_at | 4.089056765 | 8.231505382 | 11.4877487 | 4.54E-17 | 3.26E-16 | 27.95563769 | OLFM1 |
| 210201_x_at | -4.088357139 | 8.510622895 | -19.93880625 | 7.75E-29 | 3.34E-27 | 55.23490558 | BIN1 |
| 224596_at | 4.080156824 | 7.007284582 | 20.99049951 | 4.67E-30 | 2.56E-28 | 58.06178063 | SLC44A1 |
| 204570_at | -4.074687882 | 9.650211982 | -13.37874274 | 4.93E-20 | 5.37E-19 | 34.82433862 | COX7A1 |
| 240490_at | -4.073275214 | 4.408436808 | -24.36840062 | 1.11E-33 | 1.05E-31 | 66.45260942 | LOC101929592 |
| 226743_at | 4.063283089 | 6.751270572 | 14.10183728 | 4.08E-21 | 5.19E-20 | 37.33136686 | SLFN11 |
| 1558953_s_at | 4.055227944 | 7.47793847 | 38.70888025 | 1.72E-45 | 1.01E-42 | 93.60627556 | CEP164 |
| 217947_at | 4.051926573 | 8.162848159 | 31.02934336 | 9.18E-40 | 2.39E-37 | 80.48032772 | CMTM6 |
| 209773_s_at | 4.050324143 | 7.272593372 | 12.66054968 | 6.24E-19 | 5.78E-18 | 32.26824453 | RRM2 |
| 201968_s_at | -4.039376913 | 8.917853221 | -18.14915063 | 1.20E-26 | 3.60E-25 | 50.16250467 | PGM1 |
| 221276_s_at | -4.02527246 | 5.301910122 | -22.61505384 | 7.46E-32 | 5.26E-30 | 62.22189729 | SYNC |
| 221232_s_at | -4.021493707 | 5.483834052 | -13.75509353 | 1.34E-20 | 1.57E-19 | 36.13752525 | ANKRD2 |
| 201963_at | -4.016254563 | 7.326920817 | -13.00037754 | 1.86E-19 | 1.85E-18 | 33.48588392 | ACSL1 |
| 228242_at | 4.015084369 | 6.437970894 | 21.83848121 | 5.23E-31 | 3.36E-29 | 60.26346908 | N4BP2 |
| 211138_s_at | 4.011436891 | 7.243096551 | 9.566611998 | 7.18E-14 | 3.44E-13 | 20.55646474 | KMO |
| 237222_at | -4.002767145 | 5.067272799 | -68.67371371 | 9.91E-61 | 1.19E-56 | 127.7353772 | FSD2 |
| 219427_at | 4.001415949 | 7.676728951 | 13.04283527 | 1.60E-19 | 1.61E-18 | 33.63698531 | FAT4 |
| 201505_at | 3.997733562 | 7.333162247 | 15.82940972 | 1.39E-23 | 2.56E-22 | 43.05222324 | LAMB1 |
| 207876_s_at | -3.995080929 | 7.722204005 | -21.6252985 | 9.01E-31 | 5.55E-29 | 59.71628482 | FLNC |
| 236352_at | -3.993076476 | 5.157151566 | -32.30316663 | 8.52E-41 | 2.49E-38 | 82.85344565 | VGLL2 |
| 209295_at | 3.992757773 | 7.151579464 | 17.89247466 | 2.54E-26 | 7.18E-25 | 49.40646872 | TNFRSF10B |
| 213880_at | -3.985065362 | 4.762153151 | -13.12167074 | 1.21E-19 | 1.24E-18 | 33.91694189 | LGR5 |
| 202391_at | 3.978758303 | 7.917480695 | 15.13515195 | 1.31E-22 | 2.06E-21 | 40.79827917 | BASP1 |
| 205872_x_at | -3.9762387 | 8.545954698 | -10.85883195 | 4.84E-16 | 3.02E-15 | 25.57567646 | PDE4DIP |
| 209043_at | 3.968315122 | 8.90164306 | 30.65776324 | 1.87E-39 | 4.64E-37 | 79.77137748 | PAPSS1 |
| 218168_s_at | -3.966881508 | 8.17781694 | -33.14552408 | 1.85E-41 | 5.73E-39 | 84.37605067 | ADCK3 |
| 225016_at | 3.963186872 | 7.653038045 | 9.65998871 | 4.97E-14 | 2.43E-13 | 20.92424498 | APCDD1 |
| 230720_at | 3.956361121 | 6.317716056 | 11.17102615 | 1.49E-16 | 9.86E-16 | 26.76269696 | RNF182 |
| 225541_at | 3.944164434 | 7.937398985 | 25.43949351 | 9.51E-35 | 1.11E-32 | 68.91528544 | RPL22L1 |
| 241436_at | 3.934835573 | 5.998524796 | 8.995399738 | 6.86E-13 | 2.92E-12 | 18.2928535 | SCNN1G |
| 213552_at | 3.928112696 | 6.555190564 | 13.80023114 | 1.14E-20 | 1.35E-19 | 36.29380516 | GLCE |
| 230097_at | 3.924366376 | 7.17827142 | 23.11199506 | 2.20E-32 | 1.70E-30 | 63.44723273 | GART |
| 203786_s_at | -3.917892437 | 6.462017819 | -23.30066595 | 1.39E-32 | 1.10E-30 | 63.90690192 | TPD52L1 |
| 228095_at | 3.915271157 | 7.448279707 | 26.52224889 | 8.67E-36 | 1.24E-33 | 71.31749289 | PHF14 |
| 226190_at | 3.910014539 | 7.273408848 | 28.63564604 | 1.02E-37 | 1.95E-35 | 75.7724576 | MAP3K13 |
| 244771_at | -3.896156241 | 4.712045141 | -24.58957269 | 6.61E-34 | 6.60E-32 | 66.96842315 | KBTBD12 |
| 230611_at | -3.894549936 | 6.05453926 | -31.68872201 | 2.65E-40 | 7.46E-38 | 81.71961682 | SYPL2 |
| 206306_at | -3.884089879 | 4.195462853 | -19.40406532 | 3.37E-28 | 1.30E-26 | 53.75483337 | RYR3 |
| 200762_at | 3.881990115 | 10.4487816 | 18.34641299 | 6.75E-27 | 2.12E-25 | 50.73854264 | DPYSL2 |
| 225665_at | -3.881472023 | 9.101274063 | -16.65221443 | 1.06E-24 | 2.34E-23 | 45.64692704 | ZAK |
| 228640_at | 3.863615801 | 5.581783884 | 8.737015353 | 1.92E-12 | 7.69E-12 | 17.26236517 | PCDH7 |
| 212840_at | 3.855060227 | 7.357161085 | 28.88680314 | 6.10E-38 | 1.20E-35 | 76.28257866 | UBXN7 |
| 213385_at | 3.853088343 | 6.776628298 | 11.32462424 | 8.35E-17 | 5.74E-16 | 27.34267374 | CHN2 |
| 201976_s_at | 3.847490292 | 8.320032822 | 9.984002575 | 1.40E-14 | 7.32E-14 | 22.19479366 | MYO10 |
| 217871_s_at | 3.843152975 | 9.529220485 | 21.61400394 | 9.28E-31 | 5.66E-29 | 59.6871776 | MIF |
| 219918_s_at | 3.840203437 | 6.429698116 | 13.50865922 | 3.13E-20 | 3.50E-19 | 35.27969896 | ASPM |
| 207066_at | -3.839319257 | 5.912601674 | -28.91241376 | 5.80E-38 | 1.15E-35 | 76.33437525 | HRC |
| 223068_at | 3.838609456 | 7.1007139 | 23.04972968 | 2.56E-32 | 1.95E-30 | 63.29486764 | EML4 |
| 215076_s_at | 3.829416756 | 10.67890993 | 10.34435871 | 3.47E-15 | 1.96E-14 | 23.59669218 | COL3A1 |
| 238549_at | 3.829217331 | 8.152226207 | 21.22161691 | 2.55E-30 | 1.47E-28 | 58.66855941 | CBFA2T2 |
| 229567_at | -3.823255766 | 7.230958935 | -27.891692 | 4.70E-37 | 8.09E-35 | 74.23794737 | FITM1 |
| 221854_at | 3.819374245 | 7.387929469 | 7.012524454 | 1.94E-09 | 5.46E-09 | 10.35290704 | PKP1 |
| 223764_x_at | -3.81860028 | 4.903475594 | -18.92530469 | 1.29E-27 | 4.60E-26 | 52.40435347 | NIPSNAP3B |
| 223475_at | 3.816434734 | 5.887937306 | 11.29702207 | 9.26E-17 | 6.33E-16 | 27.23864916 | CRISPLD1 |
| 235128_at | -3.807240497 | 7.304772104 | -25.78809559 | 4.36E-35 | 5.33E-33 | 69.69802409 | SYNPO |
| 201645_at | 3.805417861 | 6.507914571 | 9.985898163 | 1.39E-14 | 7.27E-14 | 22.20219981 | TNC |
| 223122_s_at | 3.795634952 | 7.068165946 | 5.726998178 | 3.11E-07 | 6.88E-07 | 5.319605972 | SFRP2 |
| 34408_at | -3.790930805 | 7.157420284 | -27.81552933 | 5.51E-37 | 9.26E-35 | 74.078832 | RTN2 |
| 225803_at | -3.788214314 | 7.684461809 | -11.07284901 | 2.15E-16 | 1.40E-15 | 26.39058068 | FBXO32 |
| 228737_at | 3.782304077 | 5.579396422 | 10.07180838 | 9.97E-15 | 5.30E-14 | 22.53751115 | TOX2 |
| 200783_s_at | 3.773330159 | 7.011514558 | 24.32512246 | 1.22E-33 | 1.14E-31 | 66.35122364 | STMN1 |
| 213658_at | -3.760598413 | 5.687922554 | -27.79138942 | 5.80E-37 | 9.66E-35 | 74.02832061 | AK055981 |
| 242178_at | 3.759614691 | 6.394481069 | 6.971384995 | 2.29E-09 | 6.38E-09 | 10.18897747 | LIPI |
| 214710_s_at | 3.759450143 | 6.297036624 | 14.38156277 | 1.59E-21 | 2.14E-20 | 38.28327011 | CCNB1 |
| 238914_at | 3.759106285 | 5.719697837 | 9.467118222 | 1.06E-13 | 4.98E-13 | 20.16384233 | DCC |
| 208204_s_at | -3.756310098 | 6.685820391 | -36.14456483 | 1.06E-43 | 4.32E-41 | 89.51934639 | CAV3 |
| 208950_s_at | 3.754338106 | 7.779401869 | 12.25776742 | 2.67E-18 | 2.27E-17 | 30.80621148 | ALDH7A1 |
| 218736_s_at | -3.751354762 | 6.341032038 | -15.77052149 | 1.68E-23 | 3.06E-22 | 42.86335738 | PALMD |
| 225710_at | 3.741350159 | 7.756836839 | 16.24933726 | 3.70E-24 | 7.47E-23 | 44.38670493 | GNB4 |
| 225290_at | 3.741092872 | 6.678215286 | 20.96271915 | 5.02E-30 | 2.73E-28 | 57.98850173 | ETNK1 |
| 239203_at | -3.738128728 | 4.877675604 | -37.69251837 | 8.55E-45 | 4.30E-42 | 92.01834769 | LSMEM1 |
| 203964_at | 3.733267707 | 7.470547243 | 15.53408226 | 3.58E-23 | 6.21E-22 | 42.10074323 | NMI |
| 223642_at | 3.732161954 | 6.447204256 | 8.580460865 | 3.59E-12 | 1.39E-11 | 16.63645598 | ZIC2 |
| 212886_at | -3.73151155 | 5.234619837 | -19.42898164 | 3.15E-28 | 1.22E-26 | 53.82445536 | CCDC69 |
| 204041_at | -3.728506928 | 5.990515938 | -23.05778288 | 2.51E-32 | 1.92E-30 | 63.31459268 | MAOB |
| 221729_at | 3.728218255 | 7.620745408 | 10.50253256 | 1.89E-15 | 1.10E-14 | 24.20804432 | COL5A2 |
| 223694_at | -3.718952158 | 5.26029379 | -44.46573031 | 3.90E-49 | 3.25E-46 | 101.8988197 | TRIM7 |
| 201667_at | 3.71290081 | 8.422807073 | 10.07470013 | 9.86E-15 | 5.25E-14 | 22.54878598 | GJA1 |
| 230270_at | 3.710013986 | 6.495988454 | 22.7459534 | 5.40E-32 | 3.91E-30 | 62.54673971 | PRPF38B |
| 226150_at | 3.706510893 | 6.588150889 | 20.07687212 | 5.33E-29 | 2.36E-27 | 55.61229296 | PPAPDC1B |
| 209633_at | -3.70061427 | 7.533077455 | -26.94880019 | 3.45E-36 | 5.19E-34 | 72.240871 | PPP2R3A |
| 217028_at | 3.700324777 | 6.771905517 | 10.90545972 | 4.06E-16 | 2.56E-15 | 25.75364911 | CXCR4 |
| 217963_s_at | 3.697911991 | 10.3876394 | 28.45799934 | 1.46E-37 | 2.73E-35 | 75.40925097 | NGFRAP1 |
| 210632_s_at | -3.695396886 | 5.292480157 | -27.63282031 | 8.08E-37 | 1.32E-34 | 73.69556852 | SGCA |
| 200644_at | 3.692472801 | 8.765285321 | 25.44257205 | 9.44E-35 | 1.11E-32 | 68.92223743 | MARCKSL1 |
| 227623_at | -3.686583776 | 8.099367021 | -10.85697174 | 4.88E-16 | 3.04E-15 | 25.56857131 | CACNA2D1 |
| 203566_s_at | -3.685425333 | 8.760272019 | -19.39799405 | 3.43E-28 | 1.32E-26 | 53.73785899 | AGL |
| 201584_s_at | 3.676189754 | 7.835418439 | 29.96953566 | 7.08E-39 | 1.67E-36 | 78.43755223 | DDX39A |
| 39966_at | 3.670983291 | 7.758199411 | 9.558969885 | 7.40E-14 | 3.54E-13 | 20.52633445 | CSPG5 |
| 202242_at | -3.67096287 | 6.314999136 | -14.29682908 | 2.11E-21 | 2.78E-20 | 37.99597585 | TSPAN7 |
| 226683_at | 3.664910301 | 8.664534679 | 12.51286225 | 1.06E-18 | 9.53E-18 | 31.73452102 | SNX18 |
| 225698_at | 3.661728878 | 7.934510556 | 15.35528764 | 6.38E-23 | 1.07E-21 | 41.5194537 | EPB41L4A-AS1 |
| 231577_s_at | 3.660555537 | 7.491053333 | 12.91000575 | 2.56E-19 | 2.50E-18 | 33.16350016 | GBP1 |
| 227850_x_at | 3.658634745 | 6.07997209 | 12.1366633 | 4.15E-18 | 3.44E-17 | 30.36267504 | CDC42EP5 |
| 221933_at | 3.655826858 | 5.944969971 | 11.21206707 | 1.27E-16 | 8.54E-16 | 26.91792901 | NLGN4X |
| 203413_at | 3.653931222 | 7.969412989 | 7.896003198 | 5.58E-11 | 1.88E-10 | 13.89162158 | NELL2 |
| 238706_at | 3.653413211 | 7.133977822 | 23.38600198 | 1.13E-32 | 9.10E-31 | 64.11382245 | PAPD4 |
| 212510_at | -3.651120355 | 7.837210172 | -19.63875027 | 1.76E-28 | 7.15E-27 | 54.4080347 | GPD1L |
| 226227_x_at | 3.649964721 | 10.09040231 | 21.7802461 | 6.07E-31 | 3.83E-29 | 60.11441007 | ZFAS1 |
| 212582_at | 3.647766663 | 8.704994969 | 24.05160632 | 2.32E-33 | 2.05E-31 | 65.70700414 | OSBPL8 |
| 226612_at | -3.646613943 | 6.114320541 | -10.53968903 | 1.64E-15 | 9.58E-15 | 24.35128834 | UBE2QL1 |
| 219829_at | -3.646595563 | 5.072442191 | -38.36740245 | 2.94E-45 | 1.63E-42 | 93.07728351 | ITGB1BP2 |
| 204451_at | 3.64592916 | 7.491253124 | 16.06265738 | 6.66E-24 | 1.28E-22 | 43.79611372 | FZD1 |
| 201242_s_at | -3.645354899 | 7.948844625 | -7.851277902 | 6.68E-11 | 2.24E-10 | 13.71205989 | ATP1B1 |
| 211963_s_at | 3.6405322 | 8.909190042 | 26.52766288 | 8.57E-36 | 1.23E-33 | 71.32929257 | ARPC5 |
| 202587_s_at | -3.637080685 | 8.32175316 | -19.71320342 | 1.44E-28 | 5.92E-27 | 54.61406876 | AK1 |
| 227561_at | 3.636537487 | 8.276201327 | 16.83594442 | 6.03E-25 | 1.39E-23 | 46.21520099 | DDR2 |
| 226362_at | 3.6340506 | 8.287396083 | 14.01126523 | 5.56E-21 | 6.95E-20 | 37.02101004 | RP11-846E15.2 |
| 225295_at | 3.626716204 | 6.33324617 | 19.58844082 | 2.03E-28 | 8.14E-27 | 54.26848939 | SLC39A10 |
| 212774_at | -3.623594697 | 6.993215914 | -13.93090291 | 7.31E-21 | 8.99E-20 | 36.74476013 | ZBTB18 |
| 215440_s_at | 3.623426172 | 6.812397681 | 15.57331303 | 3.16E-23 | 5.52E-22 | 42.22775731 | BEX4 |
| 223100_s_at | 3.621879584 | 7.560969842 | 28.12863337 | 2.88E-37 | 5.19E-35 | 74.73053391 | NUDT5 |
| 228186_s_at | -3.616817188 | 4.980749485 | -19.57533411 | 2.10E-28 | 8.39E-27 | 54.23209175 | RSPO3 |
| 224694_at | 3.610414903 | 8.505676102 | 14.43138681 | 1.34E-21 | 1.83E-20 | 38.45177432 | ANTXR1 |
| 218332_at | 3.60878804 | 8.717907744 | 8.244472906 | 1.38E-11 | 5.00E-11 | 15.29024634 | BEX1 |
| 208623_s_at | 3.60536999 | 7.529606211 | 14.37186771 | 1.64E-21 | 2.21E-20 | 38.25044484 | EZR |
| 218224_at | 3.60442054 | 7.12760123 | 17.58800664 | 6.24E-26 | 1.66E-24 | 48.50001349 | PNMA1 |
| 219855_at | 3.601346705 | 7.131779654 | 14.18591251 | 3.07E-21 | 3.95E-20 | 37.61852357 | NUDT11 |
| 213547_at | -3.59406273 | 6.893514367 | -23.41736877 | 1.05E-32 | 8.47E-31 | 64.18972598 | CAND2 |
| 202870_s_at | 3.591633697 | 6.504124944 | 16.62484905 | 1.15E-24 | 2.54E-23 | 45.56194271 | CDC20 |
| 218831_s_at | 3.590653027 | 9.392353182 | 15.23289491 | 9.50E-23 | 1.54E-21 | 41.11923793 | FCGRT |
| 237802_at | 3.588247671 | 6.685149655 | 6.895799766 | 3.10E-09 | 8.49E-09 | 9.888111157 | XKR4 |
| 207558_s_at | -3.58322919 | 4.458042012 | -13.42808432 | 4.15E-20 | 4.55E-19 | 34.99753615 | PITX2 |
| 202403_s_at | 3.583089325 | 11.01129754 | 11.0891166 | 2.02E-16 | 1.32E-15 | 26.45231402 | COL1A2 |
| 213040_s_at | 3.577605581 | 6.341187761 | 12.03383349 | 6.04E-18 | 4.90E-17 | 29.98464859 | NPTXR |
| 201088_at | 3.57668642 | 7.964211833 | 18.77792529 | 1.96E-27 | 6.77E-26 | 51.98370854 | KPNA2 |
| 228333_at | 3.567716055 | 6.881654451 | 14.76063197 | 4.48E-22 | 6.54E-21 | 39.55734683 | ZEB2 |
| 204026_s_at | 3.560980788 | 6.689677135 | 17.64562697 | 5.26E-26 | 1.42E-24 | 48.67236903 | ZWINT |
| 225516_at | -3.555308379 | 6.700866179 | -12.72977876 | 4.87E-19 | 4.57E-18 | 32.51748448 | SLC7A2 |
| 228891_at | -3.554904974 | 5.292020687 | -22.40605199 | 1.25E-31 | 8.61E-30 | 61.70011589 | SEMA4D |
| 226254_s_at | 3.553397404 | 8.085168004 | 23.69105196 | 5.46E-33 | 4.51E-31 | 64.84852044 | KIAA1430 |
| 208813_at | -3.552931615 | 7.57677766 | -21.89718218 | 4.51E-31 | 2.91E-29 | 60.41340617 | GOT1 |
| 203661_s_at | -3.551269532 | 5.888221741 | -22.20517585 | 2.07E-31 | 1.38E-29 | 61.19496889 | TMOD1 |
| 230852_at | -3.550665098 | 6.436871992 | -24.98154356 | 2.69E-34 | 2.91E-32 | 67.87317004 | STAC3 |
| 224937_at | 3.545441726 | 6.709168385 | 15.28874848 | 7.92E-23 | 1.30E-21 | 41.30210744 | PTGFRN |
| 218313_s_at | 3.543755668 | 6.563565869 | 13.54085843 | 2.80E-20 | 3.15E-19 | 35.39222417 | GALNT7 |
| 1556060_a_at | 3.541180222 | 7.663056502 | 27.34797781 | 1.47E-36 | 2.31E-34 | 73.09363618 | ZNF451 |
| 205249_at | 3.535486685 | 7.493977709 | 8.396145366 | 7.50E-12 | 2.81E-11 | 15.89836728 | EGR2 |
| 1552474_a_at | -3.53250845 | 8.038281715 | -27.61688769 | 8.36E-37 | 1.35E-34 | 73.66204243 | GAMT |
| 226939_at | 3.530651687 | 7.802995379 | 12.17719521 | 3.58E-18 | 2.99E-17 | 30.51132238 | CPEB2 |
| 218818_at | -3.528098786 | 7.430406022 | -27.87610212 | 4.86E-37 | 8.29E-35 | 74.20540869 | FHL3 |
| 222557_at | 3.523592469 | 7.828135798 | 18.14638447 | 1.21E-26 | 3.62E-25 | 50.15439629 | STMN3 |
| 211538_s_at | -3.518800515 | 6.94394477 | -13.96546496 | 6.50E-21 | 8.06E-20 | 36.8636703 | HSPA2 |
| 211764_s_at | -3.516701401 | 7.37025745 | -25.96230604 | 2.96E-35 | 3.79E-33 | 70.08583608 | UBE2D1 |
| 209129_at | 3.515768469 | 7.874708761 | 21.97417994 | 3.71E-31 | 2.41E-29 | 60.60960117 | TRIP6 |
| 201074_at | 3.513893956 | 8.195684539 | 20.87877418 | 6.26E-30 | 3.37E-28 | 57.76662082 | SMARCC1 |
| 200636_s_at | 3.502195527 | 7.293356784 | 17.79850102 | 3.34E-26 | 9.25E-25 | 49.12781584 | PTPRF |
| 226035_at | 3.499397921 | 8.15955346 | 12.73553517 | 4.77E-19 | 4.48E-18 | 32.53818157 | USP31 |
| 202263_at | -3.498969961 | 7.893030989 | -25.64489807 | 6.00E-35 | 7.22E-33 | 69.37758298 | CYB5R1 |
| 209681_at | -3.496604112 | 6.356165692 | -14.40062592 | 1.49E-21 | 2.01E-20 | 38.34777874 | SLC19A2 |
| 225878_at | -3.490189166 | 8.382551815 | -19.67744716 | 1.59E-28 | 6.48E-27 | 54.51519185 | KIF1B |
| 200967_at | 3.489806427 | 8.98104208 | 22.93634815 | 3.38E-32 | 2.56E-30 | 63.01656702 | PPIB |
| 222073_at | -3.48758029 | 4.867529327 | -9.993179634 | 1.35E-14 | 7.08E-14 | 22.23064581 | COL4A3 |
| 204083_s_at | -3.485369329 | 10.69735494 | -13.24090398 | 7.98E-20 | 8.40E-19 | 34.33885254 | TPM2 |
| 219983_at | -3.484759588 | 5.023475191 | -16.93839573 | 4.41E-25 | 1.04E-23 | 46.53034456 | HRASLS |
| 226756_at | 3.481613756 | 5.775553302 | 16.91304419 | 4.76E-25 | 1.11E-23 | 46.45247776 | CCDC71L |
| 203625_x_at | 3.476702792 | 7.405684219 | 15.66241063 | 2.37E-23 | 4.23E-22 | 42.5155127 | SKP2 |
| 219144_at | -3.475812532 | 6.502289339 | -17.91396946 | 2.38E-26 | 6.79E-25 | 49.4700654 | DUSP26 |
| 205047_s_at | 3.475120879 | 6.658419574 | 14.67058568 | 6.04E-22 | 8.67E-21 | 39.25634666 | ASNS |
| 1553959_a_at | 3.471219083 | 7.011874072 | 28.3768643 | 1.72E-37 | 3.17E-35 | 75.24270162 | B3GALT6 |
| 204035_at | 3.470973648 | 6.146787218 | 5.763447883 | 2.70E-07 | 6.02E-07 | 5.458784019 | SCG2 |
| 228614_at | -3.467035192 | 7.403404898 | -29.07576212 | 4.17E-38 | 8.44E-36 | 76.66378793 | LINC00116 |
| 216733_s_at | -3.46594928 | 6.600145575 | -11.89745789 | 9.96E-18 | 7.84E-17 | 29.48130438 | GATM |
| 212621_at | 3.460694145 | 7.286429698 | 20.60136582 | 1.30E-29 | 6.63E-28 | 57.02852441 | TMEM194A |
| 220952_s_at | 3.456762296 | 6.087677025 | 16.48252783 | 1.79E-24 | 3.81E-23 | 45.11851474 | PLEKHA5 |
| 209014_at | 3.455979855 | 10.35959652 | 24.83134023 | 3.79E-34 | 4.06E-32 | 67.52787906 | MAGED1 |
| 233952_s_at | 3.451685292 | 6.737514036 | 21.43624565 | 1.47E-30 | 8.77E-29 | 59.22751336 | ZBTB21 |
| 219866_at | -3.450118832 | 6.794257682 | -15.68048284 | 2.24E-23 | 4.01E-22 | 42.57376012 | CLIC5 |
| 208700_s_at | 3.449820117 | 7.496592402 | 27.86618124 | 4.96E-37 | 8.39E-35 | 74.18469387 | TKT |
| 209568_s_at | 3.448134922 | 8.407526283 | 16.73762278 | 8.15E-25 | 1.84E-23 | 45.9115925 | RGL1 |
| 206102_at | 3.438383994 | 6.019964324 | 14.21850163 | 2.75E-21 | 3.55E-20 | 37.72958812 | GINS1 |
| 208148_at | -3.43807574 | 4.182956891 | -15.49156949 | 4.11E-23 | 7.07E-22 | 41.96288774 | MYH4 |
| 206326_at | 3.436994034 | 6.860788597 | 5.919896361 | 1.47E-07 | 3.38E-07 | 6.059149634 | GRP |
| 215691_x_at | 3.435999314 | 8.034121969 | 24.53952506 | 7.43E-34 | 7.28E-32 | 66.85204069 | HSPB11 |
| 203936_s_at | 3.433729345 | 8.254236933 | 6.71535343 | 6.37E-09 | 1.69E-08 | 9.171734028 | MMP9 |
| 205888_s_at | 3.430957747 | 7.399339562 | 11.33688234 | 7.97E-17 | 5.51E-16 | 27.38884279 | JAKMIP2 |
| 222549_at | 3.429470026 | 7.111732584 | 5.879263546 | 1.72E-07 | 3.93E-07 | 5.902771644 | CLDN1 |
| 202557_at | 3.427965809 | 6.627799588 | 22.56191296 | 8.51E-32 | 5.97E-30 | 62.08959389 | HSPA13 |
| 214352_s_at | 3.427915784 | 8.202659967 | 21.25845959 | 2.32E-30 | 1.35E-28 | 58.76481715 | KRAS |
| 203997_at | -3.427248087 | 5.565793594 | -17.73748145 | 4.00E-26 | 1.10E-24 | 48.94634331 | PTPN3 |
| 205508_at | -3.426566979 | 6.707779974 | -26.80394374 | 4.71E-36 | 6.99E-34 | 71.92871236 | SCN1B |
| 205827_at | 3.420723115 | 6.801195011 | 5.697753278 | 3.48E-07 | 7.67E-07 | 5.208137891 | CCK |
| 225017_at | 3.418375494 | 7.516021784 | 19.95757042 | 7.37E-29 | 3.19E-27 | 55.28630878 | CCDC14 |
| 201589_at | 3.408906643 | 7.452952283 | 21.74668871 | 6.61E-31 | 4.15E-29 | 60.02837488 | SMC1A |
| 239153_at | 3.408740186 | 5.518632621 | 8.825622983 | 1.35E-12 | 5.52E-12 | 17.61613426 | HOTAIR |
| 205990_s_at | 3.407876399 | 7.481647001 | 8.406296289 | 7.20E-12 | 2.70E-11 | 15.93904508 | WNT5A |
| 206385_s_at | -3.406554937 | 6.22470349 | -9.281833427 | 2.21E-13 | 9.95E-13 | 19.43072212 | ANK3 |
| 210976_s_at | -3.405539445 | 9.520745829 | -17.05371117 | 3.11E-25 | 7.43E-24 | 46.88357948 | PFKM |
| 212368_at | 3.404980425 | 8.634126546 | 31.36714221 | 4.85E-40 | 1.33E-37 | 81.11817591 | ZNF292 |
| 200923_at | 3.40300649 | 7.802707025 | 14.60583676 | 7.49E-22 | 1.06E-20 | 39.03927356 | LGALS3BP |
| 225619_at | 3.398023223 | 5.402794234 | 8.546586897 | 4.11E-12 | 1.59E-11 | 16.50089667 | SLAIN1 |
| 215695_s_at | 3.393533814 | 6.745221303 | 9.954849511 | 1.57E-14 | 8.13E-14 | 22.08085065 | GYG2 |
| 200943_at | 3.387184141 | 9.387208809 | 23.75447782 | 4.70E-33 | 3.94E-31 | 65.00030965 | HMGN1 |
| 215157_x_at | 3.384778144 | 11.25708414 | 30.3534827 | 3.35E-39 | 8.16E-37 | 79.1850236 | PABPC1 |
| 218039_at | 3.383145837 | 7.392284043 | 14.10403863 | 4.05E-21 | 5.15E-20 | 37.33889703 | NUSAP1 |
| 219288_at | 3.381757348 | 6.671077012 | 17.90574314 | 2.44E-26 | 6.95E-25 | 49.44573235 | C3orf14 |
| 227404_s_at | 3.380255969 | 9.160305832 | 5.95200087 | 1.30E-07 | 2.99E-07 | 6.182920502 | EGR1 |
| 208966_x_at | 3.376125583 | 8.144661127 | 15.67966016 | 2.25E-23 | 4.02E-22 | 42.57110946 | IFI16 |
| 225763_at | -3.373851499 | 5.736390539 | -16.62225741 | 1.16E-24 | 2.56E-23 | 45.55388965 | RCSD1 |
| 212190_at | 3.371440083 | 7.857383892 | 9.481299837 | 1.00E-13 | 4.72E-13 | 20.21985189 | SERPINE2 |
| 221645_s_at | 3.370000645 | 6.127044942 | 16.58908247 | 1.29E-24 | 2.81E-23 | 45.45073335 | ZNF83 |
| 202761_s_at | -3.36569766 | 7.549842262 | -15.5208489 | 3.74E-23 | 6.46E-22 | 42.05785567 | SYNE2 |
| 214722_at | 3.362375872 | 9.110911778 | 22.04156275 | 3.13E-31 | 2.05E-29 | 60.78085449 | NOTCH2NL |
| 222735_at | -3.36155394 | 6.694449464 | -16.62079102 | 1.17E-24 | 2.57E-23 | 45.54933271 | TMEM38B |
| 213217_at | -3.360354675 | 6.661645633 | -14.44311155 | 1.29E-21 | 1.77E-20 | 38.4913813 | ADCY2 |
| 204620_s_at | 3.35970949 | 7.98339556 | 7.04452073 | 1.71E-09 | 4.82E-09 | 10.48048327 | VCAN |
| 228151_at | 3.359509788 | 6.973658092 | 20.11668858 | 4.78E-29 | 2.13E-27 | 55.72076875 | ARL15 |
| 213423_x_at | 3.359345641 | 7.289472737 | 13.49002482 | 3.34E-20 | 3.72E-19 | 35.21451752 | TUSC3 |
| 203085_s_at | 3.350961733 | 6.735876046 | 16.38007601 | 2.46E-24 | 5.09E-23 | 44.79780293 | TGFB1 |
| 225627_s_at | 3.348626826 | 7.186087179 | 15.04986613 | 1.73E-22 | 2.67E-21 | 40.5172468 | CACHD1 |
| 235425_at | 3.348086619 | 5.754622687 | 13.74771118 | 1.37E-20 | 1.60E-19 | 36.11194055 | SGOL2 |
| 200677_at | 3.342842181 | 10.08808935 | 15.47992707 | 4.27E-23 | 7.31E-22 | 41.92509588 | PTTG1IP |
| 208794_s_at | 3.338940631 | 8.113826787 | 24.63151004 | 6.00E-34 | 6.08E-32 | 67.06579431 | SMARCA4 |
| 225747_at | -3.338068888 | 7.057814835 | -24.33587778 | 1.19E-33 | 1.12E-31 | 66.37643357 | COQ10A |
| 228409_at | -3.336701233 | 5.949549901 | -25.77819434 | 4.46E-35 | 5.42E-33 | 69.67591608 | PLIN4 |
| 226269_at | 3.332802665 | 6.291276629 | 12.03700867 | 5.97E-18 | 4.85E-17 | 29.99634071 | GDAP1 |
| 223361_at | 3.332163109 | 5.803258724 | 13.93942546 | 7.10E-21 | 8.75E-20 | 36.77409601 | ABRACL |
| 232235_at | 3.331243079 | 5.086417806 | 10.55839836 | 1.52E-15 | 8.95E-15 | 24.42336195 | DSEL |
| 203845_at | -3.327976597 | 5.667696115 | -15.12247938 | 1.36E-22 | 2.14E-21 | 40.75657849 | KAT2B |
| 236841_at | 3.327184249 | 6.554488983 | 20.32493775 | 2.73E-29 | 1.27E-27 | 56.28552876 | LOC100134445 |
| 232935_at | 3.327162587 | 7.588161617 | 12.25962021 | 2.65E-18 | 2.26E-17 | 30.81298312 | AK021977 |
| 227970_at | -3.325829757 | 6.573114251 | -29.21621709 | 3.15E-38 | 6.62E-36 | 76.94572239 | GPR157 |
| 206453_s_at | -3.325383388 | 7.728678749 | -19.76554545 | 1.24E-28 | 5.17E-27 | 54.75857318 | NDRG2 |
| 221802_s_at | 3.3231231 | 6.601713978 | 12.40800509 | 1.55E-18 | 1.36E-17 | 31.35392024 | KIAA1598 |
| 244881_at | 3.322894768 | 7.217343241 | 19.96843027 | 7.15E-29 | 3.10E-27 | 55.3160423 | LMLN |
| 224506_s_at | -3.322824769 | 6.297200089 | -25.92096728 | 3.24E-35 | 4.13E-33 | 69.99401183 | PPAPDC3 |
| 209191_at | 3.321897409 | 7.713540218 | 15.36382315 | 6.21E-23 | 1.04E-21 | 41.54729445 | TUBB6 |
| 204396_s_at | 3.321467009 | 7.806351293 | 13.75464893 | 1.34E-20 | 1.57E-19 | 36.13598464 | GRK5 |
| 236681_at | 3.318772966 | 7.385797822 | 9.630619055 | 5.58E-14 | 2.71E-13 | 20.80864255 | HOXD13 |
| 202431_s_at | 3.31740557 | 8.304732787 | 12.16332432 | 3.76E-18 | 3.14E-17 | 30.46047486 | MYC |
| 218858_at | -3.316832365 | 7.312144662 | -14.50884484 | 1.04E-21 | 1.44E-20 | 38.71310901 | DEPTOR |
| 204162_at | 3.314579412 | 5.966621452 | 15.02349646 | 1.88E-22 | 2.89E-21 | 40.43016889 | NDC80 |
| 202747_s_at | 3.314059914 | 10.43285662 | 8.649288378 | 2.73E-12 | 1.08E-11 | 16.9117572 | ITM2A |
| 208158_s_at | -3.306978462 | 7.412360911 | -17.96077404 | 2.07E-26 | 5.98E-25 | 49.608366 | OSBPL1A |
| 202074_s_at | -3.305518616 | 8.220682552 | -21.79659444 | 5.82E-31 | 3.70E-29 | 60.15628681 | OPTN |
| 242871_at | 3.299628588 | 6.038958826 | 7.625469323 | 1.66E-10 | 5.26E-10 | 12.80571907 | PAQR5 |
| 201312_s_at | 3.299094034 | 8.897971303 | 16.3831372 | 2.44E-24 | 5.07E-23 | 44.80740388 | SH3BGRL |
| 223170_at | 3.296112475 | 7.952913274 | 18.74336648 | 2.16E-27 | 7.35E-26 | 51.88473303 | TMEM98 |
| 212488_at | 3.295505845 | 8.308858228 | 11.04309886 | 2.41E-16 | 1.56E-15 | 26.27760615 | COL5A1 |
| 226217_at | 3.293840734 | 6.902590578 | 34.48628304 | 1.75E-42 | 5.83E-40 | 86.72712948 | SLC30A7 |
| 212826_s_at | 3.293194381 | 10.43029198 | 35.51352477 | 3.03E-43 | 1.13E-40 | 88.47131302 | SLC25A6 |
| 201697_s_at | 3.291716584 | 6.954688936 | 22.38319026 | 1.33E-31 | 9.06E-30 | 61.64280636 | DNMT1 |
| 203999_at | 3.289338031 | 6.591993512 | 7.818275824 | 7.63E-11 | 2.54E-10 | 13.57956826 | SYT1 |
| 205412_at | -3.288152224 | 8.816940609 | -25.23745781 | 1.50E-34 | 1.73E-32 | 68.45748974 | ACAT1 |
| 235165_at | 3.287635649 | 5.149076613 | 10.33086904 | 3.66E-15 | 2.06E-14 | 23.54443797 | PARD6B |
| 208659_at | 3.286826082 | 8.799097161 | 28.22516165 | 2.36E-37 | 4.29E-35 | 74.93016797 | CLIC1 |
| 227556_at | -3.285919275 | 7.264955945 | -8.722968749 | 2.03E-12 | 8.12E-12 | 17.2062497 | NME7 |
| 232001_at | -3.28459246 | 4.085384506 | -29.81937723 | 9.51E-39 | 2.19E-36 | 78.14288249 | PRKCQ-AS1 |
| 224518_s_at | 3.284140561 | 6.642940874 | 20.79540263 | 7.80E-30 | 4.11E-28 | 57.54558184 | ZNF559 |
| 206481_s_at | 3.278851806 | 7.831474351 | 10.82678324 | 5.47E-16 | 3.38E-15 | 25.45321311 | LDB2 |
| 224840_at | -3.275519786 | 7.858497791 | -11.57301875 | 3.30E-17 | 2.42E-16 | 28.2748122 | FKBP5 |
| 217733_s_at | 3.274404025 | 11.60193468 | 29.11235915 | 3.88E-38 | 7.99E-36 | 76.73736529 | TMSB10 |
| 225297_at | 3.272449214 | 6.741886301 | 19.00687345 | 1.03E-27 | 3.71E-26 | 52.63616164 | HAUS1 |
| 220940_at | 3.269675249 | 7.714636549 | 13.48025427 | 3.46E-20 | 3.84E-19 | 35.18032326 | ANKRD36B |
| 242539_at | 3.267757533 | 5.93128767 | 17.85794287 | 2.81E-26 | 7.87E-25 | 49.30419031 | DIS3L2 |
| 201995_at | 3.266017638 | 7.757521769 | 21.5705107 | 1.04E-30 | 6.29E-29 | 59.57498056 | EXT1 |
| 223366_at | 3.265492417 | 8.505398023 | 14.68299273 | 5.80E-22 | 8.33E-21 | 39.29788106 | ZNF704 |
| 216237_s_at | 3.265356876 | 6.512241388 | 17.89956303 | 2.48E-26 | 7.06E-25 | 49.42744686 | MCM5 |
| 227621_at | 3.257659051 | 8.371442946 | 18.30398487 | 7.63E-27 | 2.38E-25 | 50.61500974 | WTAP |
| 219757_s_at | 3.256605641 | 6.725108431 | 29.62975086 | 1.38E-38 | 3.12E-36 | 77.76885737 | TMEM260 |
| 213106_at | -3.255594023 | 5.994923693 | -12.88294629 | 2.82E-19 | 2.73E-18 | 33.06676915 | ATP8A1 |
| 209118_s_at | 3.254520977 | 10.29495607 | 18.08936091 | 1.42E-26 | 4.24E-25 | 49.98705428 | TUBA1A |
| 224932_at | -3.253267177 | 8.836817455 | -25.9884162 | 2.79E-35 | 3.60E-33 | 70.14376955 | CHCHD10 |
| 223306_at | 3.25263361 | 7.395898255 | 17.83714243 | 2.98E-26 | 8.34E-25 | 49.2425172 | EBPL |
| 211765_x_at | 3.250357134 | 11.49375908 | 40.15293716 | 1.89E-46 | 1.21E-43 | 95.7945194 | PPIA |
| 202779_s_at | 3.247378341 | 6.960392062 | 18.04392507 | 1.63E-26 | 4.81E-25 | 49.85345753 | UBE2S |
| 238877_at | -3.247322621 | 5.213727631 | -10.25337025 | 4.93E-15 | 2.73E-14 | 23.24388883 | EYA4 |
| 203476_at | 3.24658507 | 7.310044858 | 11.00167155 | 2.82E-16 | 1.81E-15 | 26.12012368 | TPBG |
| 230029_x_at | -3.242279399 | 8.256897412 | -18.76466456 | 2.04E-27 | 6.98E-26 | 51.94574545 | UBR3 |
| 201128_s_at | 3.241068596 | 7.978984864 | 27.19901832 | 2.02E-36 | 3.11E-34 | 72.77668158 | ACLY |
| 200989_at | 3.23899339 | 8.773514776 | 20.33204539 | 2.68E-29 | 1.25E-27 | 56.30472779 | HIF1A |
| 229138_at | 3.235415214 | 6.067181333 | 12.83228562 | 3.38E-19 | 3.23E-18 | 32.88542004 | PARP11 |
| 203362_s_at | 3.221200948 | 5.957134372 | 12.14229852 | 4.06E-18 | 3.38E-17 | 30.38335385 | MAD2L1 |
| 217908_s_at | -3.219968333 | 8.410084435 | -25.59655532 | 6.68E-35 | 8.00E-33 | 69.26906171 | DCAF6 |
| 213139_at | 3.217800463 | 6.843420896 | 9.085547547 | 4.80E-13 | 2.09E-12 | 18.65151982 | SNAI2 |
| 217781_s_at | -3.215353275 | 8.715334363 | -20.51271009 | 1.65E-29 | 8.13E-28 | 56.79105338 | ZNF106 |
| 222258_s_at | 3.209003304 | 7.174723408 | 13.3858741 | 4.80E-20 | 5.24E-19 | 34.84939017 | SH3BP4 |
| 213238_at | 3.207579803 | 6.207770719 | 15.04709937 | 1.74E-22 | 2.69E-21 | 40.50811452 | ATP10D |
| 209815_at | 3.204980887 | 6.575812344 | 15.92701894 | 1.02E-23 | 1.92E-22 | 43.36433636 | PTCH1 |
| 205231_s_at | -3.203777045 | 6.310096141 | -34.13097511 | 3.24E-42 | 1.05E-39 | 86.11248813 | EPM2A |
| 225655_at | 3.203616386 | 6.675443868 | 12.49453571 | 1.13E-18 | 1.01E-17 | 31.66810026 | UHRF1 |
| 228153_at | -3.202393668 | 6.313737066 | -13.86439824 | 9.18E-21 | 1.11E-19 | 36.51552209 | RNF144B |
| 218824_at | 3.195340977 | 5.931062293 | 9.219241148 | 2.82E-13 | 1.26E-12 | 19.18252015 | PNMAL1 |
| 229041_s_at | 3.193765302 | 6.93497757 | 8.451697773 | 6.01E-12 | 2.28E-11 | 16.12094451 | ITGB2-AS1 |
| 224968_at | 3.193258876 | 6.872367382 | 19.8155461 | 1.09E-28 | 4.58E-27 | 54.89635102 | CCDC104 |
| 202718_at | 3.191913403 | 7.524288951 | 9.756779522 | 3.40E-14 | 1.70E-13 | 21.30472583 | IGFBP2 |
| 226433_at | -3.190841762 | 4.931945997 | -27.20563643 | 1.99E-36 | 3.08E-34 | 72.79079545 | RNF157 |
| 201909_at | 3.188498416 | 7.80772196 | 4.084949807 | 0.000127363 | 0.000214484 | -0.557875019 | RPS4Y1 |
| 209496_at | 3.188332028 | 8.14121017 | 7.95760649 | 4.36E-11 | 1.49E-10 | 14.13894494 | RARRES2 |
| 222691_at | 3.18422127 | 7.162397017 | 19.17004532 | 6.48E-28 | 2.41E-26 | 53.09774647 | SLC35B3 |
| 202469_s_at | 3.182607529 | 7.07258122 | 24.67126822 | 5.48E-34 | 5.60E-32 | 67.15797863 | CPSF6 |
| 219426_at | 3.179666269 | 6.792823363 | 21.2938606 | 2.12E-30 | 1.25E-28 | 58.85718732 | AGO3 |
| 222544_s_at | 3.172334833 | 8.879113883 | 26.29193133 | 1.43E-35 | 1.96E-33 | 70.81359876 | WHSC1L1 |
| 228315_at | 3.170929542 | 7.466230976 | 13.20664761 | 9.00E-20 | 9.40E-19 | 34.21782087 | ZMAT3 |
| 222379_at | 3.168436225 | 6.605342575 | 8.705847395 | 2.17E-12 | 8.67E-12 | 17.1378387 | KCNE4 |
| 205656_at | 3.167138912 | 8.053045331 | 9.011878827 | 6.43E-13 | 2.74E-12 | 18.35845359 | PCDH17 |
| 202075_s_at | 3.166215487 | 7.270712322 | 13.15673496 | 1.07E-19 | 1.11E-18 | 34.04120586 | PLTP |
| 218204_s_at | -3.164576297 | 7.606952289 | -17.78375934 | 3.49E-26 | 9.63E-25 | 49.08401271 | FYCO1 |
| 213699_s_at | 3.164517613 | 10.27505647 | 30.88308494 | 1.21E-39 | 3.12E-37 | 80.20219842 | YWHAQ |
| 215963_x_at | 3.162904339 | 11.49715972 | 31.2574119 | 5.96E-40 | 1.61E-37 | 80.9116652 | RPL3 |
| 219787_s_at | 3.161711831 | 5.265023328 | 14.42323783 | 1.38E-21 | 1.88E-20 | 38.4242362 | ECT2 |
| 202920_at | -3.159844385 | 6.137926269 | -11.14104271 | 1.66E-16 | 1.10E-15 | 26.64916754 | ANK2 |
| 231792_at | -3.157097713 | 6.603630143 | -35.06255141 | 6.51E-43 | 2.35E-40 | 87.71151014 | MYLK2 |
| 235619_at | -3.157060294 | 4.721947493 | -29.55890592 | 1.59E-38 | 3.48E-36 | 77.62857138 | ASB4 |
| 200891_s_at | 3.152590598 | 9.560912772 | 25.43476924 | 9.61E-35 | 1.12E-32 | 68.90461566 | SSR1 |
| 242438_at | 3.151863086 | 7.120699649 | 24.31583849 | 1.25E-33 | 1.16E-31 | 66.3294551 | ASXL1 |
| 204036_at | 3.149395188 | 6.698196274 | 11.93503587 | 8.67E-18 | 6.88E-17 | 29.62022587 | LPAR1 |
| 242856_at | 3.149334556 | 6.298778446 | 8.436454075 | 6.38E-12 | 2.41E-11 | 16.05987826 | AL132709.8 |
| 221984_s_at | 3.146751156 | 7.849474421 | 30.47535888 | 2.65E-39 | 6.52E-37 | 79.42051483 | FAM134A |
| 218574_s_at | -3.140910188 | 7.695731491 | -13.09004304 | 1.36E-19 | 1.38E-18 | 33.80472222 | LMCD1 |
| 200703_at | 3.138452557 | 9.834185691 | 30.76716316 | 1.51E-39 | 3.85E-37 | 79.9809094 | DYNLL1 |
| 206488_s_at | -3.136581752 | 6.988845144 | -8.926842397 | 9.01E-13 | 3.77E-12 | 18.01977519 | CD36 |
| 226126_at | 3.135243438 | 6.962052851 | 21.24292663 | 2.42E-30 | 1.40E-28 | 58.72425033 | TBCK |
| 201558_at | 3.134607395 | 7.909566328 | 24.28013329 | 1.36E-33 | 1.26E-31 | 66.2456714 | RAE1 |
| 205480_s_at | -3.133062142 | 8.865602465 | -18.87627699 | 1.48E-27 | 5.25E-26 | 52.26467978 | UGP2 |
| 213103_at | 3.131970117 | 7.880800816 | 10.87119985 | 4.62E-16 | 2.89E-15 | 25.62290636 | STARD13 |
| 208788_at | 3.129800052 | 7.685678168 | 15.7161677 | 2.00E-23 | 3.60E-22 | 42.68865532 | ELOVL5 |
| 238124_at | -3.128304096 | 5.217380793 | -20.52923235 | 1.58E-29 | 7.83E-28 | 56.8353681 | MYOM3 |
| 243296_at | 3.127511992 | 7.123095675 | 8.979060068 | 7.32E-13 | 3.10E-12 | 18.22779306 | NAMPT |
| 209596_at | 3.127179796 | 7.066727276 | 7.002583783 | 2.02E-09 | 5.67E-09 | 10.31328541 | MXRA5 |
| 236029_at | 3.123415933 | 6.456265635 | 9.012526448 | 6.41E-13 | 2.74E-12 | 18.36103133 | FAT3 |
| 218980_at | -3.123323169 | 5.158284532 | -16.51117533 | 1.64E-24 | 3.52E-23 | 45.20796633 | FHOD3 |
| 210046_s_at | -3.122804426 | 8.207026383 | -16.35007206 | 2.70E-24 | 5.56E-23 | 44.70364029 | IDH2 |
| 241425_at | 3.120782931 | 7.499194539 | 14.47632001 | 1.15E-21 | 1.59E-20 | 38.60346683 | NUPL1 |
| 224617_at | 3.119831875 | 7.663006874 | 15.98517435 | 8.50E-24 | 1.62E-22 | 43.54973766 | PTBP3 |
| 208113_x_at | 3.119738755 | 10.61997721 | 29.5349314 | 1.67E-38 | 3.61E-36 | 77.58102943 | PABPC3 |
| 212820_at | 3.119262434 | 5.848730713 | 13.5463147 | 2.75E-20 | 3.09E-19 | 35.41127882 | DMXL2 |
| 200011_s_at | 3.113986897 | 7.692052843 | 26.80833288 | 4.67E-36 | 6.97E-34 | 71.93819209 | ARF3 |
| 223175_s_at | -3.111597307 | 7.507348509 | -35.93553803 | 1.50E-43 | 5.79E-41 | 89.17412427 | FEM1A |
| 201443_s_at | 3.106959268 | 8.785039471 | 22.07268264 | 2.89E-31 | 1.90E-29 | 60.85980675 | ATP6AP2 |
| 229580_at | 3.106623008 | 6.925626707 | 6.661005641 | 7.91E-09 | 2.08E-08 | 8.956555719 | CLSTN2 |
| 222620_s_at | 3.105149448 | 8.371191598 | 21.33501312 | 1.90E-30 | 1.13E-28 | 58.96441594 | DNAJC1 |
| 232024_at | 3.105148399 | 5.817670877 | 9.148000195 | 3.75E-13 | 1.65E-12 | 18.89970856 | GIMAP2 |
| 208636_at | 3.105005625 | 7.908405462 | 14.26780939 | 2.33E-21 | 3.05E-20 | 37.8973726 | ACTN1 |
| 211935_at | 3.102700914 | 8.344329734 | 21.77288104 | 6.18E-31 | 3.89E-29 | 60.09553624 | ARL6IP1 |
| 202419_at | 3.102632011 | 8.37356015 | 10.84255287 | 5.15E-16 | 3.20E-15 | 25.51348537 | KDSR |
| 228280_at | 3.102088669 | 7.172900916 | 20.95282523 | 5.16E-30 | 2.80E-28 | 57.9623857 | ZC3HAV1L |
| 218847_at | 3.101015129 | 7.595258727 | 14.02326118 | 5.34E-21 | 6.71E-20 | 37.06217595 | IGF2BP2 |
| 218662_s_at | 3.097815381 | 6.215933406 | 12.37698679 | 1.73E-18 | 1.51E-17 | 31.24106959 | NCAPG |
| 204137_at | 3.093641057 | 5.642779442 | 10.98448554 | 3.01E-16 | 1.92E-15 | 26.0547365 | GPR137B |
| 219360_s_at | 3.093268403 | 7.211460906 | 12.17236798 | 3.64E-18 | 3.04E-17 | 30.49362955 | TRPM4 |
| 202252_at | 3.090526806 | 9.806407984 | 23.45348882 | 9.65E-33 | 7.82E-31 | 64.27702961 | RAB13 |
| 233949_s_at | -3.08955213 | 5.44940908 | -11.28252576 | 9.78E-17 | 6.66E-16 | 27.18398162 | MYH7B |
| 219148_at | 3.088045827 | 6.290889336 | 11.15305697 | 1.59E-16 | 1.05E-15 | 26.69467058 | PBK |
| 211696_x_at | -3.085855579 | 9.345850653 | -5.385204985 | 1.15E-06 | 2.41E-06 | 4.02883217 | HBB |
| 214121_x_at | -3.085038972 | 7.176014191 | -16.51598107 | 1.61E-24 | 3.48E-23 | 45.22296254 | PDLIM7 |
| 203087_s_at | 3.084841671 | 5.877732473 | 15.8485975 | 1.31E-23 | 2.42E-22 | 43.11367003 | KIF2A |
| 201494_at | 3.084061133 | 8.119313738 | 18.05779433 | 1.56E-26 | 4.62E-25 | 49.89426235 | PRCP |
| 222896_at | -3.083984605 | 7.214074289 | -12.86443418 | 3.02E-19 | 2.91E-18 | 33.00053939 | TMEM38A |
| 201029_s_at | 3.078933543 | 10.9749024 | 24.11909449 | 1.98E-33 | 1.78E-31 | 65.86651947 | CD99 |
| 235367_at | -3.078793788 | 6.65187 | -15.76665762 | 1.70E-23 | 3.09E-22 | 42.85095027 | MYPN |
| 218380_at | 3.077247494 | 7.940002495 | 8.501736863 | 4.92E-12 | 1.88E-11 | 16.32134807 | LOC728392 |
| 201786_s_at | 3.072121286 | 9.140307544 | 32.81513187 | 3.36E-41 | 1.02E-38 | 83.78314967 | ADAR |
| 204464_s_at | 3.071711106 | 7.269888807 | 8.344555253 | 9.22E-12 | 3.41E-11 | 15.69158529 | EDNRA |
| 203430_at | -3.070483489 | 7.676823952 | -11.23597698 | 1.16E-16 | 7.86E-16 | 27.00827673 | HEBP2 |
| 227628_at | 3.068611342 | 6.468682653 | 13.06525078 | 1.48E-19 | 1.49E-18 | 33.71666664 | GPX8 |
| 218819_at | 3.066692892 | 7.544380044 | 19.9407183 | 7.71E-29 | 3.33E-27 | 55.24014515 | INTS6 |
| 203253_s_at | 3.065606493 | 6.25737651 | 20.40887307 | 2.18E-29 | 1.03E-27 | 56.51193293 | PPIP5K2 |
| 201306_s_at | 3.065272238 | 9.799262087 | 23.80207256 | 4.19E-33 | 3.55E-31 | 65.11399489 | ANP32B |
| 223077_at | 3.065127759 | 5.482064446 | 17.52479557 | 7.53E-26 | 1.97E-24 | 48.31049752 | TMOD3 |
| 219892_at | -3.063685322 | 4.970572596 | -17.44871461 | 9.45E-26 | 2.44E-24 | 48.08178724 | TM6SF1 |
| 206612_at | -3.059322606 | 5.537602271 | -25.77030436 | 4.53E-35 | 5.49E-33 | 69.65829379 | CACNG1 |
| 202502_at | -3.058003651 | 8.882550677 | -20.39304241 | 2.27E-29 | 1.07E-27 | 56.46928539 | ACADM |
| 208611_s_at | 3.057868315 | 7.947276177 | 18.08419391 | 1.45E-26 | 4.30E-25 | 49.97187318 | SPTAN1 |
| 229941_at | -3.057506878 | 5.052416046 | -21.62234041 | 9.08E-31 | 5.57E-29 | 59.70866267 | FAM166B |
| 1568983_a_at | 3.057386346 | 7.818134436 | 10.6347132 | 1.14E-15 | 6.78E-15 | 24.71697209 | GABPB1-AS1 |
| 228973_at | 3.057143651 | 6.233876581 | 8.620948975 | 3.05E-12 | 1.19E-11 | 16.79842627 | DLG2 |
| 208890_s_at | 3.056645807 | 7.30979394 | 26.26581079 | 1.52E-35 | 2.05E-33 | 70.75621342 | PLXNB2 |
| 228988_at | 3.054074274 | 6.458340895 | 10.0076768 | 1.28E-14 | 6.70E-14 | 22.28726654 | ZNF711 |
| 226237_at | 3.045932873 | 6.2497642 | 5.650312459 | 4.18E-07 | 9.12E-07 | 5.027701965 | COL8A1 |
| 227385_at | 3.044897483 | 6.125993903 | 12.06518827 | 5.38E-18 | 4.40E-17 | 30.10005365 | PPAPDC2 |
| 238740_at | -3.041462038 | 4.105226758 | -37.67659941 | 8.77E-45 | 4.31E-42 | 91.99315078 | PTGES3L |
| 220576_at | 3.04135073 | 5.80178394 | 9.26919965 | 2.32E-13 | 1.04E-12 | 19.38064572 | PGAP1 |
| 228948_at | 3.040646162 | 7.378514075 | 9.843495948 | 2.43E-14 | 1.23E-13 | 21.64493728 | EPHA4 |
| 213164_at | 3.038122544 | 7.310766292 | 15.02910982 | 1.85E-22 | 2.84E-21 | 40.44871267 | SLC5A3 |
| 229160_at | -3.038122036 | 4.128371436 | -21.93889523 | 4.05E-31 | 2.63E-29 | 60.5197606 | MUM1L1 |
| 209612_s_at | -3.037460821 | 4.489644992 | -8.768017535 | 1.70E-12 | 6.84E-12 | 17.38618508 | ADH1B |
| 209146_at | 3.035029243 | 6.437460053 | 13.9458606 | 6.95E-21 | 8.57E-20 | 36.79624057 | MSMO1 |
| 223059_s_at | 3.034264749 | 6.705581567 | 12.73865088 | 4.72E-19 | 4.44E-18 | 32.54938233 | FAM107B |
| 213593_s_at | 3.034243341 | 7.842224521 | 22.75408368 | 5.29E-32 | 3.84E-30 | 62.56686664 | TRA2A |
| 213920_at | 3.034014526 | 5.91361989 | 8.316874636 | 1.03E-11 | 3.79E-11 | 15.58060747 | CUX2 |
| 202897_at | 3.033955567 | 6.971305036 | 10.70688098 | 8.64E-16 | 5.21E-15 | 24.99406544 | SIRPA |
| 201752_s_at | 3.030486113 | 8.472118362 | 17.16476867 | 2.22E-25 | 5.42E-24 | 47.22229761 | ADD3 |
| 220917_s_at | 3.029243962 | 6.467352129 | 19.50192792 | 2.57E-28 | 1.01E-26 | 54.02791165 | WDR19 |
| 223381_at | 3.028875441 | 5.165998766 | 9.541233427 | 7.93E-14 | 3.78E-13 | 20.45638803 | NUF2 |
| 219410_at | 3.02832234 | 5.766925402 | 10.31572921 | 3.88E-15 | 2.18E-14 | 23.48577024 | TMEM45A |
| 224985_at | 3.023290327 | 7.280315763 | 21.82084094 | 5.47E-31 | 3.48E-29 | 60.21834974 | NRAS |
| 208891_at | 3.023201791 | 7.087322275 | 8.300873455 | 1.10E-11 | 4.03E-11 | 15.51644645 | DUSP6 |
| 207966_s_at | 3.023119367 | 9.945523515 | 17.28087135 | 1.56E-25 | 3.90E-24 | 47.57486459 | GLG1 |
| 229854_at | -3.02254494 | 6.815055085 | -18.70319702 | 2.43E-27 | 8.17E-26 | 51.76952632 | OBSCN |
| 1555868_at | -3.021751923 | 4.396939032 | -17.12157376 | 2.53E-25 | 6.12E-24 | 47.09072735 | LOC100507477 |
| 203102_s_at | 3.020714047 | 6.858388918 | 22.09292593 | 2.75E-31 | 1.81E-29 | 60.91111771 | MGAT2 |
| 230671_at | -3.017353128 | 4.858761476 | -25.53615204 | 7.65E-35 | 9.11E-33 | 69.13322288 | CTD-2083E4.7 |
| 231880_at | -3.016150938 | 4.810739759 | -14.36084209 | 1.70E-21 | 2.29E-20 | 38.21310002 | STRIP2 |
| 201012_at | 3.015399343 | 8.452310957 | 9.343295909 | 1.73E-13 | 7.90E-13 | 19.67418339 | ANXA1 |
| 226032_at | 3.014761463 | 7.222367793 | 28.39499897 | 1.66E-37 | 3.08E-35 | 75.27996378 | CASP2 |
| 226674_at | -3.013806384 | 6.245096444 | -24.36095439 | 1.13E-33 | 1.06E-31 | 66.43517608 | SHISA4 |
| 214844_s_at | -3.012445644 | 6.122912437 | -9.732620087 | 3.74E-14 | 1.86E-13 | 21.20982868 | DOK5 |
| 227003_at | 3.010829288 | 7.103903297 | 18.63414875 | 2.95E-27 | 9.78E-26 | 51.57108554 | RAB28 |
| 200881_s_at | 3.010302622 | 8.75405349 | 21.12987378 | 3.24E-30 | 1.82E-28 | 58.42830533 | DNAJA1 |
| 200657_at | 3.00855383 | 9.22777254 | 21.63224236 | 8.85E-31 | 5.46E-29 | 59.73417399 | SLC25A5 |
| 230438_at | -3.005138578 | 5.641162226 | -17.99776016 | 1.86E-26 | 5.43E-25 | 49.71748017 | TBX15 |
| 219032_x_at | 3.005032668 | 7.388014202 | 11.86339264 | 1.13E-17 | 8.81E-17 | 29.35522093 | OPN3 |
| 201664_at | 3.0050053 | 7.483717319 | 17.91987704 | 2.34E-26 | 6.69E-25 | 49.48753503 | SMC4 |
| 212288_at | 3.000109926 | 8.421275324 | 11.35944534 | 7.33E-17 | 5.09E-16 | 27.47377887 | FNBP1 |
| 204400_at | 2.99999412 | 6.736417133 | 10.07410741 | 9.88E-15 | 5.26E-14 | 22.54647506 | EFS |
| 200625_s_at | 2.991856139 | 8.838885162 | 22.6639285 | 6.61E-32 | 4.72E-30 | 62.34336057 | CAP1 |
| 212952_at | 2.990807703 | 8.059384948 | 18.19357428 | 1.05E-26 | 3.21E-25 | 50.29260587 | CTC-425F1.4 |
| 201818_at | 2.988372241 | 6.881330207 | 17.12325433 | 2.51E-25 | 6.11E-24 | 47.09585038 | LPCAT1 |
| 226800_at | 2.987484369 | 5.687335031 | 15.25754199 | 8.77E-23 | 1.42E-21 | 41.19998273 | EFCAB7 |
| 201426_s_at | 2.986545586 | 11.46934707 | 11.13670477 | 1.69E-16 | 1.11E-15 | 26.63273389 | VIM |
| 237244_at | -2.986078326 | 4.614511862 | -36.66888477 | 4.46E-44 | 1.97E-41 | 90.37703257 | C10orf71-AS1 |
| 202605_at | 2.984624467 | 8.143997907 | 27.55811564 | 9.46E-37 | 1.52E-34 | 73.53822595 | GUSB |
| 214723_x_at | 2.982388509 | 5.158076473 | 14.68499683 | 5.76E-22 | 8.28E-21 | 39.30458826 | ANKRD36 |
| 223062_s_at | 2.978045199 | 6.301659188 | 9.680410624 | 4.59E-14 | 2.26E-13 | 21.00458686 | PSAT1 |
| 208319_s_at | 2.978016993 | 7.374323302 | 14.51004599 | 1.03E-21 | 1.43E-20 | 38.71715552 | RBM3 |
| 243110_x_at | 2.977481044 | 5.053906265 | 7.769958163 | 9.27E-11 | 3.05E-10 | 13.38560165 | NPW |
| 225721_at | -2.977164573 | 6.554409431 | -12.64855657 | 6.52E-19 | 6.02E-18 | 32.22500533 | SYNPO2 |
| 203044_at | 2.977048026 | 7.592592084 | 19.40981206 | 3.32E-28 | 1.28E-26 | 53.77089686 | CHSY1 |
| 228523_at | -2.976465843 | 4.574340749 | -13.82839272 | 1.04E-20 | 1.24E-19 | 36.39117687 | NANOS1 |
| 203429_s_at | 2.976389341 | 6.071690731 | 23.43854367 | 1.00E-32 | 8.08E-31 | 64.24091982 | SUCO |
| 1559977_a_at | -2.974299337 | 6.37056146 | -30.0741012 | 5.77E-39 | 1.37E-36 | 78.64196992 | SLC25A34 |
| 203344_s_at | 2.971703912 | 6.136681054 | 14.55726402 | 8.81E-22 | 1.24E-20 | 38.87608243 | RBBP8 |
| 214948_s_at | 2.971233917 | 7.831255366 | 16.92470281 | 4.60E-25 | 1.08E-23 | 46.48829641 | TMF1 |
| 212848_s_at | -2.97108741 | 7.474922815 | -15.90721997 | 1.09E-23 | 2.03E-22 | 43.30112209 | C9orf3 |
| 232338_at | 2.96978671 | 6.307960096 | 18.31906659 | 7.31E-27 | 2.29E-25 | 50.65894419 | ZNF431 |
| 221868_at | -2.969696482 | 6.716462348 | -16.32404028 | 2.93E-24 | 6.02E-23 | 44.62185569 | PAIP2B |
| 210105_s_at | 2.969251477 | 7.760649069 | 21.27904215 | 2.20E-30 | 1.28E-28 | 58.81853665 | FYN |
| 225656_at | 2.965074965 | 6.482466687 | 16.54849329 | 1.46E-24 | 3.17E-23 | 45.32434377 | EFHC1 |
| 211270_x_at | 2.964518432 | 8.41905387 | 29.59725642 | 1.47E-38 | 3.26E-36 | 77.70454963 | PTBP1 |
| 200745_s_at | 2.963779091 | 9.09677145 | 25.4677855 | 8.92E-35 | 1.06E-32 | 68.97914804 | GNB1 |
| 201648_at | 2.960934031 | 10.49903241 | 12.78256604 | 4.04E-19 | 3.83E-18 | 32.70712373 | JAK1 |
| 200665_s_at | 2.958055484 | 10.51407397 | 12.17023442 | 3.67E-18 | 3.06E-17 | 30.48580868 | SPARC |
| 212915_at | -2.95527342 | 6.238719865 | -8.388075287 | 7.75E-12 | 2.89E-11 | 15.86602592 | PDZRN3 |
| 202088_at | 2.954980501 | 8.186165049 | 18.24516651 | 9.05E-27 | 2.78E-25 | 50.44342584 | SLC39A6 |
| 201970_s_at | 2.953043858 | 7.41281198 | 17.89385792 | 2.53E-26 | 7.16E-25 | 49.41056296 | NASP |
| 205826_at | -2.949562804 | 8.117708112 | -5.950448384 | 1.31E-07 | 3.01E-07 | 6.176930995 | MYOM2 |
| 244692_at | 2.948879923 | 7.118668292 | 7.14535989 | 1.14E-09 | 3.28E-09 | 10.88297377 | CYP4F22 |
| 203729_at | 2.945769648 | 7.956007391 | 14.4412465 | 1.30E-21 | 1.78E-20 | 38.4850822 | EMP3 |
| 211964_at | 2.943835536 | 9.665618557 | 9.840658475 | 2.45E-14 | 1.24E-13 | 21.63381532 | COL4A2 |
| 201202_at | 2.94200303 | 8.34523258 | 16.34104074 | 2.78E-24 | 5.72E-23 | 44.67527566 | PCNA |
| 202806_at | 2.941688251 | 7.409176683 | 17.42661626 | 1.01E-25 | 2.59E-24 | 48.01523154 | DBN1 |
| 202544_at | 2.937708882 | 8.097342169 | 26.17914994 | 1.84E-35 | 2.41E-33 | 70.56547468 | GMFB |
| 218252_at | 2.936276414 | 6.55076613 | 18.32135748 | 7.26E-27 | 2.28E-25 | 50.66561557 | CKAP2 |
| 200708_at | -2.935991954 | 8.894508768 | -22.87829188 | 3.90E-32 | 2.92E-30 | 62.8736369 | GOT2 |
| 201897_s_at | 2.935258475 | 7.815003164 | 18.46579556 | 4.79E-27 | 1.53E-25 | 51.08506962 | CKS1B |
| 227654_at | 2.933239152 | 6.881579989 | 8.672379941 | 2.49E-12 | 9.85E-12 | 17.00407697 | FAM65C |
| 201209_at | 2.932481271 | 7.896832597 | 19.27670849 | 4.81E-28 | 1.82E-26 | 53.3979528 | HDAC1 |
| 222685_at | 2.929864881 | 6.102148978 | 16.09246238 | 6.06E-24 | 1.18E-22 | 43.89069085 | HAUS6 |
| 201231_s_at | 2.926853953 | 9.764549021 | 20.41422328 | 2.15E-29 | 1.02E-27 | 56.5263407 | ENO1 |
| 202705_at | 2.924938836 | 6.766846777 | 12.01068335 | 6.57E-18 | 5.31E-17 | 29.89936442 | CCNB2 |
| 229742_at | -2.924849569 | 7.981874322 | -18.11563651 | 1.32E-26 | 3.94E-25 | 50.06420806 | C15orf61 |
| 1554526_at | 2.924836528 | 5.444747662 | 6.281675332 | 3.56E-08 | 8.67E-08 | 7.463893575 | OLFM3 |
| 202594_at | 2.92453465 | 7.51383689 | 20.62660744 | 1.22E-29 | 6.26E-28 | 57.09599484 | LEPROTL1 |
| 225384_at | 2.924337309 | 6.454736725 | 16.71587199 | 8.71E-25 | 1.96E-23 | 45.84427301 | DOCK7 |
| 212845_at | -2.922939302 | 7.780664656 | -12.19575244 | 3.34E-18 | 2.81E-17 | 30.57931178 | SAMD4A |
| 218432_at | -2.922565906 | 6.94353994 | -14.69284697 | 5.61E-22 | 8.09E-21 | 39.33085564 | FBXO3 |
| 201560_at | 2.920330766 | 8.721637772 | 17.12207644 | 2.52E-25 | 6.12E-24 | 47.09225973 | CLIC4 |
| 223524_s_at | -2.916828687 | 5.277456833 | -20.54147783 | 1.53E-29 | 7.66E-28 | 56.86819465 | TMEM108 |
| 223592_s_at | 2.916348538 | 7.058765424 | 12.92364379 | 2.44E-19 | 2.39E-18 | 33.21221763 | RNF135 |
| 203340_s_at | -2.916023936 | 7.511182188 | -25.0378041 | 2.36E-34 | 2.61E-32 | 68.0020564 | SLC25A12 |
| 235381_at | 2.913479288 | 7.678445811 | 26.4187263 | 1.09E-35 | 1.52E-33 | 71.09146925 | RSF1 |
| 217764_s_at | 2.907883432 | 7.138394082 | 9.12699335 | 4.07E-13 | 1.78E-12 | 18.81625381 | RAB31 |
| 224740_at | 2.907451645 | 7.421619856 | 19.67190593 | 1.61E-28 | 6.56E-27 | 54.49985689 | SMIM15 |
| 200886_s_at | 2.90723831 | 9.542327881 | 22.77882386 | 4.98E-32 | 3.65E-30 | 62.62807685 | PGAM1 |
| 223638_at | 2.906548739 | 6.584580395 | 9.082592611 | 4.85E-13 | 2.11E-12 | 18.63977086 | NBPF3 |
| 31874_at | 2.906433137 | 6.563104898 | 16.20297319 | 4.28E-24 | 8.53E-23 | 44.24041995 | GAS2L1 |
| 225676_s_at | 2.904703092 | 7.149633103 | 16.60013709 | 1.24E-24 | 2.73E-23 | 45.48512189 | DCAF13 |
| 203549_s_at | -2.902584572 | 7.15954228 | -8.182366947 | 1.77E-11 | 6.32E-11 | 15.04108866 | LPL |
| 226388_at | -2.902537105 | 8.45294649 | -11.66494867 | 2.35E-17 | 1.75E-16 | 28.61795135 | TCEA3 |
| 212012_at | 2.901894816 | 7.794745344 | 11.62439374 | 2.73E-17 | 2.02E-16 | 28.46669913 | PXDN |
| 201746_at | 2.90118381 | 5.685090733 | 16.54787516 | 1.46E-24 | 3.17E-23 | 45.32241747 | TP53 |
| 200998_s_at | 2.90022083 | 7.94309339 | 16.52884387 | 1.55E-24 | 3.35E-23 | 45.26308711 | CKAP4 |
| 204822_at | 2.898383564 | 5.404327712 | 8.947498683 | 8.30E-13 | 3.49E-12 | 18.10208102 | TTK |
| 222731_at | -2.89826699 | 5.556606258 | -10.43815843 | 2.42E-15 | 1.39E-14 | 23.95953834 | ZDHHC2 |
| 225127_at | 2.896153808 | 7.46583401 | 19.30256413 | 4.48E-28 | 1.70E-26 | 53.47054352 | TMEM181 |
| 228806_at | -2.891056371 | 6.039867613 | -26.32153243 | 1.34E-35 | 1.85E-33 | 70.87857183 | RORC |
| 201816_s_at | -2.889620442 | 9.041897794 | -20.55896902 | 1.46E-29 | 7.34E-28 | 56.91505781 | GBAS |
| 1558956_s_at | 2.888987631 | 6.416353755 | 16.3021376 | 3.14E-24 | 6.40E-23 | 44.5529802 | IFT80 |
| 202704_at | -2.887797077 | 8.342545676 | -14.58086102 | 8.14E-22 | 1.14E-20 | 38.95539933 | TOB1 |
| 225365_at | 2.886644307 | 8.266484498 | 20.51596636 | 1.64E-29 | 8.08E-28 | 56.79978922 | ZDHHC20 |
| 225871_at | 2.886108641 | 7.198615778 | 7.481366665 | 2.96E-10 | 9.12E-10 | 12.22778036 | STEAP2 |
| 212328_at | -2.884508187 | 6.873674577 | -9.365561707 | 1.58E-13 | 7.26E-13 | 19.76231589 | LIMCH1 |
| 202620_s_at | 2.884215761 | 6.198333597 | 11.07642337 | 2.12E-16 | 1.38E-15 | 26.40414744 | PLOD2 |
| 209903_s_at | 2.881064449 | 6.725090788 | 23.81332024 | 4.08E-33 | 3.47E-31 | 65.14083404 | ATR |
| 205176_s_at | 2.880347358 | 7.017713897 | 20.13987477 | 4.49E-29 | 2.01E-27 | 55.78386352 | ITGB3BP |
| 201577_at | 2.877800875 | 8.121391419 | 18.15526429 | 1.18E-26 | 3.54E-25 | 50.18042247 | NME1 |
| 201261_x_at | 2.877745423 | 8.347871717 | 10.45346188 | 2.28E-15 | 1.31E-14 | 24.01865298 | BGN |
| 218597_s_at | -2.876030893 | 7.750940066 | -20.65652163 | 1.13E-29 | 5.85E-28 | 57.17587418 | CISD1 |
| 218519_at | 2.875424951 | 6.269668812 | 20.32473033 | 2.73E-29 | 1.27E-27 | 56.2849684 | SLC35A5 |
| 209272_at | 2.873819346 | 7.101289605 | 15.03426577 | 1.82E-22 | 2.80E-21 | 40.46574188 | NAB1 |
| 208612_at | 2.873426908 | 8.82744973 | 27.9169935 | 4.46E-37 | 7.73E-35 | 74.29072198 | PDIA3 |
| 208807_s_at | 2.872798227 | 7.542970517 | 19.85790102 | 9.67E-29 | 4.10E-27 | 55.01286069 | CHD3 |
| 219188_s_at | -2.869842891 | 7.573946612 | -19.57380675 | 2.11E-28 | 8.41E-27 | 54.22784907 | MACROD1 |
| 202214_s_at | 2.868854535 | 7.726792674 | 22.35702636 | 1.42E-31 | 9.64E-30 | 61.57716201 | CUL4B |
| 234983_at | 2.867551852 | 6.100523014 | 17.67266701 | 4.85E-26 | 1.32E-24 | 48.75312122 | C12orf49 |
| 212168_at | 2.866317839 | 6.475263702 | 23.50656727 | 8.49E-33 | 6.91E-31 | 64.40512444 | RBM12 |
| 203020_at | 2.865694528 | 6.349449975 | 19.61457309 | 1.88E-28 | 7.60E-27 | 54.34100616 | RABGAP1L |
| 1554667_s_at | 2.864092841 | 6.396716787 | 20.53633358 | 1.55E-29 | 7.72E-28 | 56.85440622 | METTL8 |
| 214141_x_at | 2.863371381 | 9.360796021 | 18.52792525 | 4.00E-27 | 1.30E-25 | 51.26479154 | SRSF7 |
| 221753_at | 2.863264803 | 7.604516835 | 12.24587071 | 2.79E-18 | 2.36E-17 | 30.76272084 | SSH1 |
| 202998_s_at | 2.863049631 | 7.107321013 | 8.330585286 | 9.76E-12 | 3.60E-11 | 15.63557899 | LOXL2 |
| 201307_at | 2.862937945 | 7.528700313 | 14.07390199 | 4.49E-21 | 5.68E-20 | 37.2357548 | SEPT11 |
| 229513_at | 2.862090065 | 6.427246201 | 7.96314438 | 4.26E-11 | 1.46E-10 | 14.16117791 | STRBP |
| 212928_at | 2.862078696 | 7.080584089 | 18.4955676 | 4.39E-27 | 1.41E-25 | 51.17124367 | TSPYL4 |
| 207956_x_at | 2.861902372 | 7.361511195 | 15.39923753 | 5.54E-23 | 9.35E-22 | 41.66271053 | PDS5B |
| 225649_s_at | 2.860978607 | 8.416034191 | 15.90020655 | 1.11E-23 | 2.08E-22 | 43.27871808 | STK35 |
| 208798_x_at | 2.860426129 | 8.121025842 | 10.59059222 | 1.35E-15 | 7.95E-15 | 24.54729701 | GOLGA8A |
| 219654_at | -2.859409446 | 5.824635536 | -13.70537688 | 1.59E-20 | 1.83E-19 | 35.96508967 | PTPLA |
| 220085_at | 2.859073685 | 5.541550235 | 13.49852415 | 3.25E-20 | 3.62E-19 | 35.2442529 | HELLS |
| 236254_at | 2.859044316 | 7.355161828 | 19.6681793 | 1.63E-28 | 6.62E-27 | 54.48954192 | VPS13B |
| 217776_at | 2.858521168 | 7.956813918 | 26.04806575 | 2.45E-35 | 3.18E-33 | 70.27593501 | RDH11 |
| 212936_at | 2.858028558 | 7.03164328 | 22.04833716 | 3.07E-31 | 2.02E-29 | 60.79804886 | FAM172A |
| 222925_at | 2.856661031 | 6.249332252 | 7.660779732 | 1.44E-10 | 4.61E-10 | 12.94740338 | DCDC2 |
| 212314_at | 2.855829669 | 7.276927509 | 8.584345924 | 3.53E-12 | 1.37E-11 | 16.65200071 | SEL1L3 |
| 212372_at | 2.854054115 | 8.1818605 | 11.71004353 | 1.99E-17 | 1.50E-16 | 28.78590519 | MYH10 |
| 225681_at | 2.852324547 | 6.706439967 | 6.057070407 | 8.61E-08 | 2.02E-07 | 6.589261947 | CTHRC1 |
| 204023_at | 2.852273372 | 6.879405349 | 17.57550713 | 6.47E-26 | 1.72E-24 | 48.46257446 | RFC4 |
| 209628_at | 2.851707666 | 5.889100694 | 10.89945369 | 4.15E-16 | 2.61E-15 | 25.73073809 | NXT2 |
| 225343_at | 2.84666437 | 6.999260491 | 20.04569796 | 5.80E-29 | 2.55E-27 | 55.52725057 | TMED8 |
| 221648_s_at | -2.846287342 | 4.729505083 | -12.68367946 | 5.75E-19 | 5.36E-18 | 32.35158409 | AGMAT |
| 203980_at | -2.843805288 | 6.883685528 | -6.232262108 | 4.32E-08 | 1.05E-07 | 7.270819261 | FABP4 |
| 227647_at | 2.842564201 | 7.021735823 | 6.454940447 | 1.79E-08 | 4.52E-08 | 8.143530277 | KCNE3 |
| 225633_at | 2.8416334 | 6.284320183 | 17.49907915 | 8.13E-26 | 2.12E-24 | 48.23326473 | DPY19L3 |
| 228977_at | -2.840710975 | 5.558542672 | -12.89494583 | 2.71E-19 | 2.63E-18 | 33.10967606 | LOC729680 |
| 217975_at | 2.84016069 | 8.184057827 | 15.66343902 | 2.37E-23 | 4.22E-22 | 42.51882832 | WBP5 |
| 223095_at | 2.839043565 | 6.911760127 | 20.23792127 | 3.45E-29 | 1.55E-27 | 56.05007295 | MARVELD1 |
| 217985_s_at | 2.833111611 | 6.949493 | 15.70624135 | 2.06E-23 | 3.70E-22 | 42.65671105 | BAZ1A |
| 214769_at | -2.830412785 | 5.52801423 | -14.29041285 | 2.16E-21 | 2.84E-20 | 37.97418399 | CLCN4 |
| 225623_at | -2.829783023 | 6.976496292 | -16.50317973 | 1.68E-24 | 3.59E-23 | 45.18301003 | CIPC |
| 230303_at | 2.82900871 | 5.909077393 | 4.488891334 | 3.13E-05 | 5.62E-05 | 0.80049493 | SYNPR |
| 213682_at | 2.827985842 | 7.454031515 | 27.06860004 | 2.67E-36 | 4.04E-34 | 72.49794164 | NUP50 |
| 201525_at | -2.827489319 | 7.759278964 | -7.166101874 | 1.05E-09 | 3.03E-09 | 10.96583751 | APOD |
| 200621_at | 2.826750863 | 8.615072242 | 16.32036745 | 2.97E-24 | 6.08E-23 | 44.6103101 | CSRP1 |
| 213170_at | 2.821244549 | 6.230603208 | 14.98595042 | 2.13E-22 | 3.25E-21 | 40.30603345 | GPX7 |
| 225501_at | 2.820321547 | 7.651236203 | 19.99690896 | 6.62E-29 | 2.88E-27 | 55.39395809 | PHF6 |
| 225005_at | 2.816569291 | 7.651433774 | 23.88027302 | 3.48E-33 | 3.02E-31 | 65.300382 | PHF13 |
| 227113_at | -2.814308444 | 6.74639656 | -26.19706774 | 1.76E-35 | 2.33E-33 | 70.60495556 | ADHFE1 |
| 230728_at | 2.813077528 | 6.669824676 | 19.41056397 | 3.31E-28 | 1.28E-26 | 53.77299836 | FKBP14 |
| 204127_at | 2.812877534 | 6.233232185 | 14.30377337 | 2.06E-21 | 2.72E-20 | 38.01955525 | RFC3 |
| 202068_s_at | 2.812359094 | 6.228355193 | 11.368963 | 7.07E-17 | 4.92E-16 | 27.50958942 | LDLR |
| 230972_at | -2.812317564 | 7.632916511 | -13.04588168 | 1.58E-19 | 1.59E-18 | 33.64781825 | ANKRD9 |
| 222209_s_at | 2.811401498 | 6.609971445 | 12.94307444 | 2.28E-19 | 2.24E-18 | 33.28158658 | TMEM135 |
| 201586_s_at | 2.808661768 | 8.641733891 | 24.6711749 | 5.48E-34 | 5.60E-32 | 67.1577624 | SFPQ |
| 203989_x_at | 2.80786471 | 6.048401846 | 7.526611718 | 2.47E-10 | 7.68E-10 | 12.40918489 | F2R |
| 235709_at | 2.805937922 | 6.924316942 | 11.27373427 | 1.01E-16 | 6.87E-16 | 27.15081595 | GAS2L3 |
| 226077_at | 2.804817699 | 8.190768482 | 16.16637484 | 4.81E-24 | 9.47E-23 | 44.12476302 | RNF145 |
| 202040_s_at | 2.801889284 | 7.46928315 | 19.41640436 | 3.26E-28 | 1.26E-26 | 53.78931964 | KDM5A |
| 201160_s_at | -2.801658252 | 10.86353253 | -13.32205071 | 6.00E-20 | 6.45E-19 | 34.62495566 | YBX3 |
| 210052_s_at | 2.801308468 | 6.58994828 | 12.31924916 | 2.13E-18 | 1.84E-17 | 31.0306896 | TPX2 |
| 201013_s_at | 2.801046806 | 7.988843284 | 16.45026323 | 1.98E-24 | 4.19E-23 | 45.01765084 | PAICS |
| 207828_s_at | 2.799334522 | 7.520139431 | 11.00764371 | 2.75E-16 | 1.77E-15 | 26.14283813 | CENPF |
| 221829_s_at | 2.797988716 | 10.44090836 | 18.16094785 | 1.16E-26 | 3.51E-25 | 50.19707599 | TNPO1 |
| 201975_at | -2.79667386 | 7.591514334 | -16.63097675 | 1.13E-24 | 2.50E-23 | 45.58098029 | CLIP1 |
| 201920_at | 2.794284537 | 7.590120132 | 17.76665162 | 3.67E-26 | 1.01E-24 | 49.03314829 | SLC20A1 |
| 213581_at | 2.792713514 | 5.750600491 | 16.3805962 | 2.46E-24 | 5.09E-23 | 44.7994345 | PDCD2 |
| 212713_at | 2.790153388 | 8.268577874 | 8.433347361 | 6.46E-12 | 2.44E-11 | 16.04743187 | MFAP4 |
| 209695_at | -2.789455445 | 7.423541142 | -16.86216586 | 5.56E-25 | 1.29E-23 | 46.29597702 | PTP4A3 |
| 205436_s_at | 2.788698055 | 7.63130597 | 16.09954503 | 5.93E-24 | 1.15E-22 | 43.91314957 | H2AFX |
| 205992_s_at | -2.788195387 | 3.810398596 | -16.70451748 | 9.02E-25 | 2.03E-23 | 45.80910802 | IL15 |
| 227295_at | 2.787910776 | 5.171984661 | 14.78391286 | 4.15E-22 | 6.10E-21 | 39.63500151 | IKBIP |
| 225174_at | 2.785851607 | 7.28562206 | 17.32664651 | 1.36E-25 | 3.44E-24 | 47.71343869 | DNAJC10 |
| 244370_at | 2.785567536 | 4.738617757 | 6.483968217 | 1.60E-08 | 4.05E-08 | 8.257765998 | KIAA2022 |
| 207382_at | -2.784967526 | 5.193656161 | -25.89278837 | 3.45E-35 | 4.37E-33 | 69.93134771 | TP63 |
| 230419_at | -2.783260549 | 4.383558111 | -18.74490849 | 2.15E-27 | 7.33E-26 | 51.88915206 | SOX9-AS1 |
| 202783_at | -2.782952507 | 7.547223488 | -15.21966006 | 9.92E-23 | 1.60E-21 | 41.07584868 | NNT |
| 212612_at | 2.781227576 | 7.467833324 | 11.5054729 | 4.25E-17 | 3.05E-16 | 28.02205169 | RCOR1 |
| 218561_s_at | 2.780682153 | 7.091350591 | 17.79305007 | 3.40E-26 | 9.39E-25 | 49.11162187 | LYRM4 |
| 200618_at | 2.780188204 | 7.977562574 | 22.64464131 | 6.93E-32 | 4.94E-30 | 62.29545303 | LASP1 |
| 227680_at | 2.778442498 | 7.198568784 | 18.76998353 | 2.01E-27 | 6.90E-26 | 51.96097498 | ZNF326 |
| 213706_at | -2.778224302 | 5.533440697 | -18.90290308 | 1.38E-27 | 4.88E-26 | 52.34056615 | GPD1 |
| 225941_at | -2.775552238 | 5.783676888 | -17.41695954 | 1.04E-25 | 2.66E-24 | 47.98612983 | EIF4E3 |
| 206981_at | -2.774327648 | 5.234317599 | -23.70496015 | 5.28E-33 | 4.40E-31 | 64.88183357 | SCN4A |
| 1556136_at | -2.774144364 | 4.679119252 | -17.55721919 | 6.84E-26 | 1.80E-24 | 48.40776546 | MYLK4 |
| 204604_at | 2.772195822 | 7.554982389 | 18.15711019 | 1.17E-26 | 3.53E-25 | 50.18583156 | CDK14 |
| 227870_at | -2.770589834 | 5.059929037 | -10.19085247 | 6.28E-15 | 3.43E-14 | 23.00101307 | IGDCC4 |
| 236088_at | 2.769948056 | 6.704324013 | 8.324689964 | 9.99E-12 | 3.68E-11 | 15.61194285 | NTNG1 |
| 218989_x_at | 2.769545289 | 6.826385569 | 16.68672262 | 9.53E-25 | 2.13E-23 | 45.75396638 | SLC30A5 |
| 214749_s_at | 2.767075868 | 8.572033436 | 16.26927045 | 3.48E-24 | 7.06E-23 | 44.4495168 | ARMCX6 |
| 213012_at | -2.766412998 | 6.429400963 | -15.26544311 | 8.54E-23 | 1.39E-21 | 41.22585104 | NEDD4 |
| 223374_s_at | 2.765006705 | 4.937733487 | 13.29743191 | 6.54E-20 | 6.98E-19 | 34.53824473 | B3GALNT1 |
| 203404_at | 2.764645469 | 7.804334846 | 8.447549549 | 6.11E-12 | 2.32E-11 | 16.10432745 | ARMCX2 |
| 224819_at | 2.763659871 | 6.577830396 | 16.41971681 | 2.18E-24 | 4.56E-23 | 44.92204295 | TCEAL8 |
| 221514_at | 2.759518094 | 6.610383972 | 20.48517798 | 1.78E-29 | 8.63E-28 | 56.7171491 | UTP14A |
| 202145_at | 2.758471738 | 7.599796079 | 12.85472799 | 3.12E-19 | 3.00E-18 | 32.96579672 | LY6E |
| 216834_at | 2.75843059 | 5.120944332 | 6.179224772 | 5.33E-08 | 1.28E-07 | 7.063982629 | RGS1 |
| 238763_at | -2.754289914 | 4.123537017 | -12.9385674 | 2.32E-19 | 2.27E-18 | 33.26550037 | RBM20 |
| 227099_s_at | 2.7527968 | 7.456016111 | 8.547165262 | 4.10E-12 | 1.58E-11 | 16.50321157 | C11orf96 |
| 209049_s_at | 2.752600254 | 7.9765072 | 11.35234479 | 7.53E-17 | 5.21E-16 | 27.44705594 | ZMYND8 |
| 213088_s_at | 2.751227711 | 8.172736318 | 14.6667904 | 6.12E-22 | 8.77E-21 | 39.24363752 | DNAJC9 |
| 212573_at | -2.748216533 | 6.275418922 | -13.86715374 | 9.10E-21 | 1.10E-19 | 36.52503139 | ENDOD1 |
| 214911_s_at | 2.746586155 | 8.644017767 | 19.51532996 | 2.48E-28 | 9.74E-27 | 54.06523135 | BRD2 |
| 222405_at | 2.745674279 | 8.643930157 | 24.74472028 | 4.62E-34 | 4.81E-32 | 67.32796187 | PTPLAD1 |
| 218053_at | 2.743682177 | 9.287312555 | 23.25930052 | 1.54E-32 | 1.20E-30 | 63.80637922 | PRPF40A |
| 200732_s_at | -2.743242331 | 8.662936232 | -20.11183185 | 4.85E-29 | 2.16E-27 | 55.70754564 | PTP4A1 |
| 225732_at | 2.741445124 | 6.917833032 | 21.71239953 | 7.21E-31 | 4.50E-29 | 59.94035645 | KLHL42 |
| 208808_s_at | 2.741388051 | 9.42565275 | 11.86722145 | 1.11E-17 | 8.69E-17 | 29.3693993 | HMGB2 |
| 212160_at | 2.736842386 | 7.59236339 | 16.26226912 | 3.56E-24 | 7.19E-23 | 44.42746028 | XPOT |
| 217989_at | 2.736760329 | 7.23579494 | 12.65372869 | 6.40E-19 | 5.91E-18 | 32.2436548 | HSD17B11 |
| 214636_at | 2.735093449 | 6.658397344 | 5.023591465 | 4.49E-06 | 8.81E-06 | 2.695718401 | CALCB |
| 204453_at | 2.734766375 | 6.819783161 | 18.42609612 | 5.37E-27 | 1.70E-25 | 50.97000972 | ZNF84 |
| 208146_s_at | 2.734249438 | 7.125327833 | 12.49509554 | 1.13E-18 | 1.01E-17 | 31.67012988 | CPVL |
| 206245_s_at | 2.733607203 | 7.978057432 | 12.46430425 | 1.26E-18 | 1.12E-17 | 31.55844066 | IVNS1ABP |
| 203743_s_at | 2.732521748 | 7.33936786 | 19.44315644 | 3.03E-28 | 1.18E-26 | 53.8640341 | TDG |
| 202954_at | 2.730292648 | 7.546401803 | 12.01174809 | 6.55E-18 | 5.30E-17 | 29.90328833 | UBE2C |
| 238520_at | 2.730093872 | 5.469528768 | 15.21413096 | 1.01E-22 | 1.63E-21 | 41.05771555 | TRERF1 |
| 235371_at | 2.728045467 | 6.032590303 | 6.354963411 | 2.66E-08 | 6.59E-08 | 7.750884741 | GXYLT2 |
| 230332_at | 2.726762824 | 7.130571339 | 20.5312151 | 1.57E-29 | 7.81E-28 | 56.84068429 | ZCCHC7 |
| 200925_at | 2.726369972 | 9.629121461 | 22.42608069 | 1.19E-31 | 8.22E-30 | 61.7502855 | COX6A1 |
| 226066_at | -2.724998873 | 6.677371608 | -12.4528699 | 1.32E-18 | 1.17E-17 | 31.51693466 | MITF |
| 209369_at | -2.724311516 | 4.205490929 | -11.21988031 | 1.24E-16 | 8.31E-16 | 26.94745988 | ANXA3 |
| 236798_at | -2.723287117 | 5.581786141 | -15.35765075 | 6.34E-23 | 1.06E-21 | 41.52716248 | LINC00888 |
| 201180_s_at | 2.721026172 | 8.601723033 | 20.38788823 | 2.31E-29 | 1.08E-27 | 56.45539476 | GNAI3 |
| 226907_at | -2.718021257 | 5.491924541 | -14.28882105 | 2.17E-21 | 2.85E-20 | 37.96877685 | PPP1R14C |
| 212096_s_at | -2.716725586 | 7.570775825 | -9.021213127 | 6.19E-13 | 2.65E-12 | 18.39560469 | MTUS1 |
| 224709_s_at | 2.714001429 | 8.335352694 | 12.78072669 | 4.06E-19 | 3.85E-18 | 32.70052176 | CDC42SE2 |
| 201348_at | -2.712139715 | 7.219974239 | -6.705780148 | 6.61E-09 | 1.75E-08 | 9.133809762 | GPX3 |
| 211980_at | 2.71129155 | 9.758953881 | 9.500566722 | 9.31E-14 | 4.40E-13 | 20.29592116 | COL4A1 |
| 211962_s_at | 2.71108915 | 8.855409615 | 19.803198 | 1.12E-28 | 4.71E-27 | 54.86234937 | ZFP36L1 |
| 208579_x_at | 2.710281316 | 6.460490885 | 10.64952303 | 1.08E-15 | 6.42E-15 | 24.77388026 | H2BFS |
| 209581_at | -2.709923777 | 5.916853869 | -13.14479529 | 1.12E-19 | 1.15E-18 | 33.9989105 | PLA2G16 |
| 223681_s_at | -2.709503293 | 5.372022403 | -11.42155438 | 5.81E-17 | 4.10E-16 | 27.70727669 | INADL |
| 201398_s_at | 2.707896726 | 9.279153383 | 20.36470213 | 2.45E-29 | 1.15E-27 | 56.39287503 | TRAM1 |
| 224839_s_at | -2.707680406 | 5.43244581 | -19.51579135 | 2.48E-28 | 9.74E-27 | 54.06651583 | GPT2 |
| 222875_at | 2.706048773 | 7.598450299 | 23.85060387 | 3.74E-33 | 3.21E-31 | 65.22972587 | DHX33 |
| 205000_at | 2.704935946 | 5.31212667 | 4.099809987 | 0.000121088 | 0.000204298 | -0.509174086 | DDX3Y |
| 202606_s_at | 2.703876337 | 7.221222648 | 16.73411149 | 8.24E-25 | 1.86E-23 | 45.90072873 | TLK1 |
| 209250_at | 2.702527239 | 8.273253284 | 19.56101876 | 2.18E-28 | 8.68E-27 | 54.19231737 | DEGS1 |
| 202769_at | 2.702347694 | 7.584939852 | 13.30318775 | 6.41E-20 | 6.86E-19 | 34.55852454 | CCNG2 |
| 218625_at | 2.700945864 | 7.304813703 | 6.897730489 | 3.07E-09 | 8.44E-09 | 9.895790972 | NRN1 |
| 209806_at | 2.700367469 | 7.695071362 | 10.75858297 | 7.09E-16 | 4.33E-15 | 25.1922394 | HIST1H2BK |
| 229694_at | 2.699943549 | 6.888032234 | 17.11512167 | 2.58E-25 | 6.24E-24 | 47.07105578 | WDR11 |
| 227748_at | 2.699019037 | 6.372283459 | 20.70722835 | 9.85E-30 | 5.14E-28 | 57.31107565 | RBMXL1 |
| 203282_at | -2.698711188 | 7.235002727 | -12.56631579 | 8.75E-19 | 7.96E-18 | 31.92801175 | GBE1 |
| 223391_at | 2.698604842 | 6.117650308 | 16.08679538 | 6.17E-24 | 1.19E-22 | 43.87271666 | SGPP1 |
| 1566342_at | -2.697885588 | 6.609867158 | -12.47951952 | 1.20E-18 | 1.06E-17 | 31.61364583 | SOD2 |
| 204236_at | 2.697076788 | 6.009576151 | 15.1461773 | 1.26E-22 | 2.00E-21 | 40.83454305 | FLI1 |
| 225009_at | 2.696594851 | 6.496781471 | 16.77099302 | 7.36E-25 | 1.67E-23 | 46.01476535 | CMTM4 |
| 208690_s_at | -2.69614949 | 8.199269169 | -8.950580658 | 8.20E-13 | 3.45E-12 | 18.11435927 | PDLIM1 |
| 228920_at | 2.695199013 | 6.509212988 | 20.60956058 | 1.28E-29 | 6.52E-28 | 57.05043572 | ZNF260 |
| 225564_at | 2.694635337 | 6.199651407 | 10.00318148 | 1.30E-14 | 6.81E-14 | 22.26971147 | SPATA13 |
| 214853_s_at | 2.69417741 | 8.988887958 | 16.39137879 | 2.38E-24 | 4.95E-23 | 44.83324675 | SHC1 |
| 206656_s_at | 2.693562444 | 7.374574949 | 19.03847384 | 9.38E-28 | 3.41E-26 | 52.7257749 | APMAP |
| 209015_s_at | -2.692327359 | 6.645720564 | -14.65065468 | 6.45E-22 | 9.19E-21 | 39.18958391 | DNAJB6 |
| 222976_s_at | 2.692245218 | 9.649352218 | 15.96925324 | 8.94E-24 | 1.69E-22 | 43.4990218 | TPM3 |
| 212775_at | 2.690385829 | 7.353185831 | 10.38539183 | 2.96E-15 | 1.69E-14 | 23.7555291 | OBSL1 |
| 218550_s_at | -2.689122665 | 6.488155971 | -21.13929606 | 3.17E-30 | 1.78E-28 | 58.453017 | LRRC20 |
| 213134_x_at | 2.687263737 | 7.040077792 | 10.20541522 | 5.94E-15 | 3.25E-14 | 23.05762155 | BTG3 |
| 226281_at | 2.683117582 | 5.47520564 | 6.390503498 | 2.31E-08 | 5.76E-08 | 7.89031687 | DNER |
| 214247_s_at | 2.682213356 | 6.975318019 | 7.584171021 | 1.96E-10 | 6.16E-10 | 12.64003827 | DKK3 |
| 1553955_at | 2.681030446 | 6.132215732 | 13.62620811 | 2.09E-20 | 2.39E-19 | 35.68985042 | PPP1R21 |
| 202857_at | 2.680447618 | 8.93500478 | 26.25441544 | 1.56E-35 | 2.09E-33 | 70.73116321 | CNPY2 |
| 219237_s_at | 2.679588562 | 6.963359436 | 21.23526475 | 2.47E-30 | 1.42E-28 | 58.70423171 | DNAJB14 |
| 208901_s_at | 2.678347714 | 8.663634237 | 25.85112519 | 3.79E-35 | 4.69E-33 | 69.83859112 | TOP1 |
| 227140_at | 2.676527738 | 5.25913683 | 6.561919671 | 1.17E-08 | 3.02E-08 | 8.565024841 | INHBA |
| 201669_s_at | 2.675488297 | 8.379014389 | 7.780966006 | 8.87E-11 | 2.92E-10 | 13.42979004 | MARCKS |
| 225412_at | 2.674893996 | 6.019198634 | 13.82360213 | 1.06E-20 | 1.26E-19 | 36.37462006 | TMEM87B |
| 204256_at | 2.67392392 | 4.982324567 | 11.19385845 | 1.36E-16 | 9.11E-16 | 26.84908087 | ELOVL6 |
| 208836_at | 2.671142438 | 8.342481746 | 15.88984228 | 1.15E-23 | 2.14E-22 | 43.24559891 | ATP1B3 |
| 206976_s_at | 2.670959464 | 8.891081839 | 13.72411319 | 1.49E-20 | 1.73E-19 | 36.03011122 | HSPH1 |
| 225716_at | 2.670905043 | 6.49590659 | 15.91754368 | 1.05E-23 | 1.97E-22 | 43.33408968 | BRI3BP |
| 207714_s_at | 2.670342493 | 7.872495645 | 15.08956953 | 1.52E-22 | 2.36E-21 | 40.6481904 | SERPINH1 |
| 212287_at | 2.668988012 | 7.738060337 | 25.08532841 | 2.12E-34 | 2.38E-32 | 68.1107403 | SUZ12 |
| 200723_s_at | 2.668413228 | 9.757671415 | 11.93932177 | 8.54E-18 | 6.78E-17 | 29.63605946 | CAPRIN1 |
| 233669_s_at | -2.666613205 | 5.478756327 | -21.02832626 | 4.23E-30 | 2.33E-28 | 58.16144073 | TRIM54 |
| 221766_s_at | 2.665882587 | 7.772358135 | 8.719397047 | 2.06E-12 | 8.23E-12 | 17.1919795 | FAM46A |
| 206364_at | 2.664932016 | 6.027031747 | 10.64566672 | 1.09E-15 | 6.51E-15 | 24.75906424 | KIF14 |
| 55872_at | 2.664761926 | 7.11563465 | 13.92820265 | 7.38E-21 | 9.07E-20 | 36.7354635 | ZNF512B |
| 218976_at | 2.657065483 | 8.178744249 | 8.551260681 | 4.03E-12 | 1.56E-11 | 16.51960311 | DNAJC12 |
| 224934_at | 2.656842618 | 7.41941841 | 18.28152465 | 8.15E-27 | 2.53E-25 | 50.54953441 | YIPF5 |
| 206928_at | 2.654757486 | 5.970261443 | 13.24795013 | 7.78E-20 | 8.22E-19 | 34.36372883 | ZNF124 |
| 214846_s_at | -2.654603067 | 5.853703198 | -16.67737548 | 9.81E-25 | 2.18E-23 | 45.72498693 | ALPK3 |
| 223468_s_at | -2.653346403 | 6.508233323 | -16.57405251 | 1.35E-24 | 2.94E-23 | 45.40395491 | RGMA |
| 229700_at | 2.651756915 | 5.324535275 | 15.35749299 | 6.34E-23 | 1.06E-21 | 41.52664788 | ZNF738 |
| 205382_s_at | -2.651027766 | 6.997005865 | -8.899210647 | 1.01E-12 | 4.18E-12 | 17.90963915 | CFD |
| 202715_at | 2.649877269 | 7.48700944 | 15.82999442 | 1.39E-23 | 2.56E-22 | 43.05409635 | CAD |
| 224944_at | 2.649870021 | 7.799077643 | 13.82739915 | 1.04E-20 | 1.25E-19 | 36.38774322 | TMPO |
| 218149_s_at | 2.648139739 | 7.74189166 | 15.12603814 | 1.35E-22 | 2.12E-21 | 40.76829106 | ZNF395 |
| 218595_s_at | 2.648018991 | 7.300125721 | 15.71463564 | 2.01E-23 | 3.61E-22 | 42.68372575 | HEATR1 |
| 225406_at | 2.64670765 | 6.778027925 | 13.75672962 | 1.33E-20 | 1.56E-19 | 36.14319446 | TWSG1 |
| 230083_at | -2.646508449 | 5.641960335 | -12.03941978 | 5.92E-18 | 4.81E-17 | 30.00521844 | USP53 |
| 213795_s_at | 2.646355667 | 6.466589339 | 23.99205529 | 2.67E-33 | 2.33E-31 | 65.56594315 | PTPRA |
| 200005_at | 2.64599779 | 9.297752518 | 18.83915569 | 1.65E-27 | 5.75E-26 | 52.15875406 | EIF3D |
| 210559_s_at | 2.645731076 | 6.568776526 | 12.32232502 | 2.11E-18 | 1.82E-17 | 31.04190764 | CDK1 |
| 225303_at | 2.643569194 | 6.658650396 | 9.888690651 | 2.03E-14 | 1.04E-13 | 21.82199145 | KIRREL |
| 207981_s_at | -2.643107954 | 3.925448017 | -15.32339468 | 7.08E-23 | 1.17E-21 | 41.41534618 | ESRRG |
| 208895_s_at | 2.642220074 | 8.217142018 | 19.08467822 | 8.24E-28 | 3.03E-26 | 52.85661097 | DDX18 |
| 203313_s_at | 2.64152829 | 6.160348816 | 13.98028896 | 6.18E-21 | 7.68E-20 | 36.91462521 | TGIF1 |
| 224726_at | 2.64025548 | 7.499671279 | 20.88763176 | 6.12E-30 | 3.30E-28 | 57.790065 | MIB1 |
| 204444_at | 2.636831348 | 5.117780234 | 10.67416128 | 9.79E-16 | 5.87E-15 | 24.86850392 | KIF11 |
| 215245_x_at | 2.634754181 | 7.288527083 | 16.50784659 | 1.66E-24 | 3.55E-23 | 45.1975774 | FMR1 |
| 222889_at | 2.633762214 | 6.129784236 | 15.69713948 | 2.12E-23 | 3.80E-22 | 42.62740938 | DCLRE1B |
| 213664_at | 2.630610877 | 5.728562796 | 8.04121685 | 3.12E-11 | 1.08E-10 | 14.47459807 | SLC1A1 |
| 212338_at | 2.628270902 | 5.346897126 | 12.09874127 | 4.76E-18 | 3.93E-17 | 30.22341597 | MYO1D |
| 205051_s_at | 2.628184064 | 6.832046286 | 8.470993553 | 5.56E-12 | 2.12E-11 | 16.19823276 | KIT |
| 217771_at | 2.626048669 | 6.608752208 | 12.81164113 | 3.64E-19 | 3.47E-18 | 32.81142608 | GOLM1 |
| 201473_at | 2.625689698 | 6.913745086 | 8.92545877 | 9.06E-13 | 3.79E-12 | 18.01426124 | JUNB |
| 210312_s_at | 2.625174612 | 7.029539468 | 27.40390589 | 1.31E-36 | 2.09E-34 | 73.21225271 | IFT20 |
| 213836_s_at | -2.623855507 | 6.150279149 | -11.57000855 | 3.34E-17 | 2.44E-16 | 28.2635593 | WIPI1 |
| 206765_at | -2.622697605 | 5.895863493 | -10.18033388 | 6.54E-15 | 3.56E-14 | 22.96011251 | KCNJ2 |
| 216449_x_at | 2.620737095 | 8.199211786 | 15.55261109 | 3.38E-23 | 5.88E-22 | 42.16075621 | HSP90B1 |
| 51228_at | 2.619050802 | 5.965553003 | 16.84585687 | 5.85E-25 | 1.35E-23 | 46.24574618 | RBM12B |
| 203302_at | 2.6188844 | 6.258262109 | 15.49069857 | 4.12E-23 | 7.08E-22 | 41.96006128 | DCK |
| 226294_x_at | 2.618510963 | 6.609436151 | 21.04468705 | 4.05E-30 | 2.24E-28 | 58.20450313 | FAM91A1 |
| 1554067_at | 2.616290884 | 5.014575812 | 13.7559334 | 1.33E-20 | 1.56E-19 | 36.14043553 | C12orf66 |
| 213272_s_at | -2.613932519 | 6.961738115 | -16.64498111 | 1.08E-24 | 2.40E-23 | 45.62447235 | TMEM159 |
| 209430_at | 2.61379273 | 6.907816534 | 15.8752352 | 1.20E-23 | 2.23E-22 | 43.19889943 | BTAF1 |
| 200773_x_at | 2.613300937 | 10.74417787 | 37.48619327 | 1.19E-44 | 5.48E-42 | 91.69097824 | PTMA |
| 225326_at | 2.612208755 | 8.327271984 | 9.745662577 | 3.56E-14 | 1.77E-13 | 21.26106504 | RBM27 |
| 226592_at | 2.611940746 | 7.271479386 | 15.88425424 | 1.17E-23 | 2.17E-22 | 43.22773678 | ZNF618 |
| 202911_at | 2.611815416 | 7.697847162 | 16.95705858 | 4.17E-25 | 9.81E-24 | 46.58761877 | MSH6 |
| 214375_at | 2.611336612 | 6.971430453 | 12.17941004 | 3.55E-18 | 2.97E-17 | 30.51943925 | PPFIBP1 |
| 1560750_at | -2.609818748 | 4.003593861 | -15.31132365 | 7.36E-23 | 1.21E-21 | 41.37590985 | LOC151121 |
| 203516_at | -2.609225842 | 7.292182676 | -25.19554064 | 1.65E-34 | 1.88E-32 | 68.36212315 | SNTA1 |
| 220784_s_at | 2.609206347 | 4.879566224 | 3.452212449 | 0.001001055 | 0.001530489 | -2.528798449 | UTS2 |
| 1560676_at | 2.606751089 | 4.779625186 | 6.386706892 | 2.35E-08 | 5.84E-08 | 7.875414032 | SIAH3 |
| 203764_at | 2.605221436 | 5.213478069 | 8.195226971 | 1.68E-11 | 6.02E-11 | 15.09268657 | DLGAP5 |
| 220755_s_at | 2.603575786 | 9.522464769 | 15.54988644 | 3.41E-23 | 5.92E-22 | 42.15193403 | C6orf48 |
| 226742_at | -2.602705638 | 7.857428811 | -17.74966517 | 3.86E-26 | 1.06E-24 | 48.98261154 | SAR1B |
| 212274_at | -2.601102592 | 7.38848427 | -18.40159099 | 5.76E-27 | 1.82E-25 | 50.89890061 | LPIN1 |
| 201675_at | -2.601054052 | 7.954976917 | -14.84432132 | 3.40E-22 | 5.06E-21 | 39.83617755 | AKAP1 |
| 215470_at | 2.600905229 | 7.349339827 | 8.629906454 | 2.94E-12 | 1.16E-11 | 16.83425132 | GTF2H2B |
| 226425_at | -2.59931619 | 6.876407874 | -11.36721349 | 7.12E-17 | 4.95E-16 | 27.50300759 | CLIP4 |
| 218171_at | 2.59919836 | 7.63533905 | 19.44181271 | 3.04E-28 | 1.18E-26 | 53.86028305 | VPS4B |
| 225889_at | 2.598284884 | 6.813669982 | 15.85880291 | 1.27E-23 | 2.35E-22 | 43.14633332 | AEBP2 |
| 228201_at | 2.596632954 | 5.784868645 | 17.30177424 | 1.47E-25 | 3.69E-24 | 47.63817359 | ARL13B |
| 219827_at | -2.596533291 | 6.456528443 | -29.1706934 | 3.45E-38 | 7.18E-36 | 76.85447509 | UCP3 |
| 203423_at | 2.594409196 | 6.906917445 | 7.046018002 | 1.70E-09 | 4.80E-09 | 10.48645488 | RBP1 |
| 205356_at | -2.593673389 | 8.391388184 | -15.82414086 | 1.42E-23 | 2.59E-22 | 43.03534237 | USP13 |
| 61734_at | 2.593139481 | 7.846876381 | 10.96258843 | 3.27E-16 | 2.08E-15 | 25.97137777 | RCN3 |
| 226630_at | 2.592453889 | 6.120370788 | 16.67235767 | 9.96E-25 | 2.21E-23 | 45.70942566 | MIS18BP1 |
| 217911_s_at | -2.591840966 | 8.356297959 | -13.4904738 | 3.34E-20 | 3.71E-19 | 35.21608852 | BAG3 |
| 242817_at | 2.58900383 | 6.2660078 | 7.251950979 | 7.43E-10 | 2.19E-09 | 11.30904432 | PGLYRP2 |
| 201930_at | 2.587823398 | 8.172774876 | 16.20942165 | 4.20E-24 | 8.39E-23 | 44.26078135 | MCM6 |
| 208999_at | 2.586843843 | 7.138021383 | 18.94939907 | 1.21E-27 | 4.32E-26 | 52.47290077 | SEPT8 |
| 208837_at | 2.586693632 | 6.1036531 | 21.28559497 | 2.16E-30 | 1.27E-28 | 58.83563081 | TMED3 |
| 212907_at | 2.585537275 | 7.225299653 | 15.34413158 | 6.62E-23 | 1.10E-21 | 41.48305165 | SLC30A1 |
| 218024_at | -2.584831193 | 8.727301149 | -20.05863103 | 5.60E-29 | 2.47E-27 | 55.56254362 | MPC1 |
| 202930_s_at | -2.583199201 | 8.335217632 | -15.65668117 | 2.42E-23 | 4.30E-22 | 42.49703801 | SUCLA2 |
| 212794_s_at | 2.583034794 | 8.042718782 | 22.3044872 | 1.61E-31 | 1.09E-29 | 61.44515898 | KIAA1033 |
| 209251_x_at | 2.582138091 | 11.20205709 | 18.137525 | 1.24E-26 | 3.70E-25 | 50.12842101 | TUBA1C |
| 238346_s_at | 2.581528252 | 6.849127668 | 19.87003138 | 9.35E-29 | 3.97E-27 | 55.04619505 | TGS1 |
| 231867_at | 2.579410599 | 5.283118146 | 6.211442062 | 4.69E-08 | 1.13E-07 | 7.189574544 | TENM2 |
| 201719_s_at | 2.579217439 | 8.319805194 | 11.46361454 | 4.97E-17 | 3.54E-16 | 27.86514578 | EPB41L2 |
| 222872_x_at | 2.578393717 | 6.132663712 | 8.181736163 | 1.77E-11 | 6.33E-11 | 15.03855771 | NABP1 |
| 200859_x_at | 2.57829578 | 8.514206887 | 17.40519307 | 1.08E-25 | 2.74E-24 | 47.95065556 | FLNA |
| 224578_at | 2.577696599 | 7.714197973 | 20.25856445 | 3.26E-29 | 1.49E-27 | 56.10599923 | RCC2 |
| 224959_at | 2.577690356 | 7.246255396 | 10.26312584 | 4.75E-15 | 2.64E-14 | 23.28175435 | SLC26A2 |
| 213226_at | 2.576928397 | 5.683805413 | 14.29261056 | 2.14E-21 | 2.82E-20 | 37.98164882 | CCNA2 |
| 213939_s_at | 2.576426608 | 7.754457461 | 13.32669052 | 5.91E-20 | 6.36E-19 | 34.64128904 | RUFY3 |
| 211700_s_at | 2.574717082 | 6.502336082 | 15.07766458 | 1.58E-22 | 2.45E-21 | 40.60894814 | TRO |
| 202680_at | 2.573826004 | 6.710777732 | 20.64250395 | 1.17E-29 | 6.06E-28 | 57.13845391 | GTF2E2 |
| 224714_at | 2.573794842 | 8.059519063 | 16.51750677 | 1.61E-24 | 3.46E-23 | 45.22772289 | NIFK |
| 229860_x_at | 2.573665503 | 6.868923537 | 11.77193506 | 1.58E-17 | 1.21E-16 | 29.01602107 | C4orf48 |
| 205450_at | -2.569097862 | 5.420810257 | -14.91126805 | 2.72E-22 | 4.11E-21 | 40.05858928 | PHKA1 |
| 218388_at | 2.567768117 | 7.271347724 | 20.27805776 | 3.10E-29 | 1.42E-27 | 56.1587712 | PGLS |
| 218248_at | 2.566920131 | 7.320441957 | 21.21369068 | 2.61E-30 | 1.50E-28 | 58.64783399 | FAM111A |
| 209318_x_at | 2.566711113 | 6.576185047 | 7.109947261 | 1.31E-09 | 3.75E-09 | 10.74155761 | PLAGL1 |
| 201957_at | -2.565831194 | 6.909918729 | -21.29637173 | 2.10E-30 | 1.24E-28 | 58.86373501 | PPP1R12B |
| 211058_x_at | 2.565525235 | 11.71603773 | 18.76782402 | 2.02E-27 | 6.93E-26 | 51.95479214 | TUBA1B |
| 225563_at | 2.564481733 | 7.928718155 | 16.13134114 | 5.36E-24 | 1.05E-22 | 44.01389821 | PAN3 |
| 217967_s_at | -2.56447212 | 7.267157681 | -7.326349481 | 5.51E-10 | 1.64E-09 | 11.60675915 | FAM129A |
| 215009_s_at | 2.564084721 | 6.575387226 | 14.95910744 | 2.33E-22 | 3.54E-21 | 40.21717584 | THAP9-AS1 |
| 227719_at | 2.563092803 | 6.935598291 | 7.427715719 | 3.67E-10 | 1.12E-09 | 12.01275225 | SMAD9 |
| 223254_s_at | 2.56284048 | 6.145244212 | 19.5757857 | 2.10E-28 | 8.39E-27 | 54.23334613 | G2E3 |
| 223125_s_at | -2.562071843 | 6.789210771 | -11.69278958 | 2.12E-17 | 1.59E-16 | 28.72167232 | C1orf21 |
| 221051_s_at | -2.561903844 | 5.688037716 | -14.91421595 | 2.70E-22 | 4.08E-21 | 40.06836985 | NMRK2 |
| 204430_s_at | -2.560850709 | 5.179725567 | -13.47288786 | 3.55E-20 | 3.92E-19 | 35.15453479 | SLC2A5 |
| 204197_s_at | 2.55921337 | 6.757033472 | 11.2530743 | 1.09E-16 | 7.40E-16 | 27.0728418 | RUNX3 |
| 1553099_at | 2.559080251 | 5.773250421 | 13.21488842 | 8.74E-20 | 9.14E-19 | 34.24695026 | TIGD1 |
| 214835_s_at | -2.558565408 | 7.668003922 | -16.09436939 | 6.03E-24 | 1.17E-22 | 43.89673848 | SUCLG2 |
| 206216_at | -2.554910432 | 6.209546681 | -13.60710053 | 2.23E-20 | 2.54E-19 | 35.62330059 | SRPK3 |
| 212852_s_at | 2.553877939 | 9.170644438 | 25.88769754 | 3.49E-35 | 4.40E-33 | 69.92002058 | TROVE2 |
| 212753_at | 2.552392581 | 6.650257807 | 20.45690524 | 1.92E-29 | 9.24E-28 | 56.64117891 | PCGF3 |
| 209714_s_at | 2.55139954 | 6.344841367 | 10.77553979 | 6.65E-16 | 4.07E-15 | 25.25717274 | CDKN3 |
| 230741_at | 2.548513814 | 5.066966772 | 7.874840697 | 6.08E-11 | 2.04E-10 | 13.80665857 | P2RX7 |
| 205132_at | -2.548005846 | 6.689365836 | -5.619636716 | 4.71E-07 | 1.02E-06 | 4.911290462 | ACTC1 |
| 218755_at | 2.547264665 | 5.97779889 | 10.3698317 | 3.15E-15 | 1.78E-14 | 23.69531642 | KIF20A |
| 202555_s_at | 2.547088954 | 7.43237547 | 10.10302485 | 8.83E-15 | 4.72E-14 | 22.65918207 | MYLK |
| 228716_at | -2.546423449 | 4.318197159 | -17.38869876 | 1.13E-25 | 2.87E-24 | 47.90090057 | THRB |
| 226936_at | 2.544505422 | 6.506891212 | 12.24644038 | 2.78E-18 | 2.36E-17 | 30.76480379 | CENPW |
| 205934_at | -2.542705885 | 4.431002808 | -12.75745162 | 4.41E-19 | 4.17E-18 | 32.61694363 | PLCL1 |
| 231579_s_at | 2.54233158 | 8.864177567 | 9.863697736 | 2.24E-14 | 1.14E-13 | 21.72410153 | TIMP2 |
| 204141_at | 2.541382215 | 8.474930188 | 13.32634524 | 5.92E-20 | 6.37E-19 | 34.64007366 | TUBB2A |
| 221761_at | 2.540956141 | 6.136361081 | 19.63038618 | 1.80E-28 | 7.29E-27 | 54.38485304 | ADSS |
| 205854_at | 2.538385662 | 7.199407307 | 24.76827418 | 4.38E-34 | 4.63E-32 | 67.38238147 | TULP3 |
| 202277_at | 2.536425413 | 7.795788366 | 20.04308737 | 5.84E-29 | 2.56E-27 | 55.5201245 | SPTLC1 |
| 205713_s_at | 2.535694114 | 6.301516203 | 4.486359303 | 3.16E-05 | 5.67E-05 | 0.791768247 | COMP |
| 235379_at | -2.535503889 | 4.255854696 | -25.85547198 | 3.75E-35 | 4.69E-33 | 69.84827451 | LOC100506114 |
| 217972_at | -2.535010673 | 7.814113574 | -18.39101767 | 5.94E-27 | 1.87E-25 | 50.86819849 | CHCHD3 |
| 200970_s_at | 2.532418063 | 7.504460217 | 20.25967271 | 3.25E-29 | 1.49E-27 | 56.10900052 | SERP1 |
| 227492_at | 2.531081312 | 6.549304291 | 7.483789706 | 2.93E-10 | 9.04E-10 | 12.23749378 | OCLN |
| 209156_s_at | 2.53089211 | 8.459244081 | 7.127309736 | 1.23E-09 | 3.52E-09 | 10.81088354 | COL6A2 |
| 202666_s_at | 2.529759992 | 6.884253451 | 16.80228177 | 6.68E-25 | 1.53E-23 | 46.11138272 | ACTL6A |
| 221208_s_at | 2.529326939 | 6.724320109 | 15.80702331 | 1.50E-23 | 2.73E-22 | 42.98047599 | MSANTD2 |
| 201508_at | 2.5293224 | 8.389567899 | 10.63970604 | 1.12E-15 | 6.65E-15 | 24.73616013 | IGFBP4 |
| 222484_s_at | -2.527752595 | 6.905531163 | -4.662903706 | 1.68E-05 | 3.11E-05 | 1.406134938 | CXCL14 |
| 225887_at | 2.527517204 | 6.232944564 | 12.67592164 | 5.91E-19 | 5.49E-18 | 32.3236392 | PROSER1 |
| 231716_at | 2.525915507 | 8.095452087 | 24.90392962 | 3.21E-34 | 3.46E-32 | 67.69496649 | RC3H2 |
| 201792_at | 2.524462337 | 7.614460002 | 8.098684622 | 2.47E-11 | 8.69E-11 | 14.70526913 | AEBP1 |
| 241117_at | 2.523872161 | 6.588157269 | 5.85750672 | 1.88E-07 | 4.26E-07 | 5.81916651 | LOXHD1 |
| 213791_at | 2.523687615 | 7.029695943 | 3.752191223 | 0.000385319 | 0.000616163 | -1.620470607 | PENK |
| 222240_s_at | 2.523641301 | 6.982054795 | 10.8087033 | 5.86E-16 | 3.61E-15 | 25.38407762 | ISYNA1 |
| 226030_at | -2.522820528 | 5.775243594 | -15.20567772 | 1.04E-22 | 1.67E-21 | 41.02998503 | ACADSB |
| 225082_at | 2.522372047 | 7.205810147 | 17.80875855 | 3.24E-26 | 9.01E-25 | 49.15828031 | CPSF3 |
| 209472_at | 2.520438653 | 7.105304247 | 16.3104825 | 3.06E-24 | 6.26E-23 | 44.57922852 | CCBL2 |
| 210762_s_at | 2.52041547 | 7.93095288 | 14.28474678 | 2.20E-21 | 2.89E-20 | 37.9549356 | DLC1 |
| 225688_s_at | 2.519728452 | 6.760367904 | 7.312407399 | 5.83E-10 | 1.74E-09 | 11.5509497 | PHLDB2 |
| 215446_s_at | 2.517628958 | 5.111535184 | 6.106461508 | 7.09E-08 | 1.68E-07 | 6.780919785 | LOX |
| 218014_at | 2.517537008 | 6.706301674 | 19.38448327 | 3.56E-28 | 1.37E-26 | 53.70007103 | NUP85 |
| 201313_at | 2.51733111 | 6.107681495 | 8.810951258 | 1.43E-12 | 5.82E-12 | 17.55758311 | ENO2 |
| 203126_at | -2.516858632 | 6.539085117 | -11.71023412 | 1.99E-17 | 1.50E-16 | 28.78661453 | IMPA2 |
| 218051_s_at | 2.516553071 | 6.02450439 | 12.52263703 | 1.02E-18 | 9.21E-18 | 31.76993047 | NT5DC2 |
| 228927_at | 2.515877761 | 6.951299595 | 21.6827104 | 7.78E-31 | 4.84E-29 | 59.8640585 | ZNF397 |
| 225356_at | 2.514855726 | 7.461237484 | 21.11103649 | 3.41E-30 | 1.90E-28 | 58.37887575 | SFT2D2 |
| 214004_s_at | 2.513848905 | 7.902237925 | 21.15997963 | 3.00E-30 | 1.70E-28 | 58.5072338 | VGLL4 |
| 230784_at | 2.513758116 | 6.223915537 | 4.915851168 | 6.68E-06 | 1.29E-05 | 2.305984565 | PRAC1 |
| 228176_at | 2.51369747 | 6.817399835 | 7.539224707 | 2.34E-10 | 7.32E-10 | 12.45976495 | S1PR3 |
| 211714_x_at | 2.513645328 | 10.33670797 | 15.86704075 | 1.24E-23 | 2.29E-22 | 43.17268991 | TUBB |
| 222244_s_at | 2.512718202 | 9.554351217 | 22.76436482 | 5.16E-32 | 3.77E-30 | 62.5923099 | TUG1 |
| 213524_s_at | -2.512549086 | 7.814501438 | -4.80134469 | 1.02E-05 | 1.92E-05 | 1.895907484 | G0S2 |
| 204685_s_at | -2.511654164 | 5.419366308 | -13.87936682 | 8.72E-21 | 1.06E-19 | 36.56716732 | ATP2B2 |
| 218503_at | 2.511290242 | 7.014938387 | 12.75423974 | 4.46E-19 | 4.21E-18 | 32.60540475 | FOCAD |
| 218750_at | 2.507628161 | 6.470897889 | 13.57924257 | 2.45E-20 | 2.77E-19 | 35.52619033 | TAF1D |
| 1557895_at | 2.506151727 | 4.65790872 | 13.68797385 | 1.68E-20 | 1.94E-19 | 35.90465483 | FLJ35934 |
| 218738_s_at | 2.505648339 | 6.790567523 | 16.35923071 | 2.63E-24 | 5.42E-23 | 44.73239478 | RNF138 |
| 200600_at | 2.503207383 | 8.847793893 | 15.22024448 | 9.90E-23 | 1.60E-21 | 41.07776511 | MSN |
| 223299_at | 2.503014554 | 8.120896336 | 11.21373463 | 1.27E-16 | 8.49E-16 | 26.92423228 | SEC11C |
| 203401_at | 2.50246088 | 5.485723744 | 16.76051157 | 7.60E-25 | 1.72E-23 | 45.98237346 | PRPS2 |
| 239288_at | -2.502079834 | 4.752556923 | -23.97185034 | 2.80E-33 | 2.44E-31 | 65.5180175 | TNIK |
| 204821_at | 2.50100639 | 6.409209833 | 12.3280938 | 2.07E-18 | 1.78E-17 | 31.06294392 | BTN3A3 |
| 202413_s_at | 2.498522846 | 8.241734619 | 15.02400125 | 1.88E-22 | 2.89E-21 | 40.43183665 | USP1 |
| 223502_s_at | 2.497581536 | 6.013683823 | 8.160062815 | 1.93E-11 | 6.88E-11 | 14.95159181 | TNFSF13B |
| 203497_at | 2.497139984 | 7.63469683 | 23.10346434 | 2.25E-32 | 1.73E-30 | 63.4263774 | MED1 |
| 213812_s_at | 2.496842517 | 7.987963809 | 18.57507611 | 3.50E-27 | 1.15E-25 | 51.40090237 | CAMKK2 |
| 211031_s_at | 2.495490616 | 5.842238312 | 20.25288102 | 3.31E-29 | 1.51E-27 | 56.09060599 | CLIP2 |
| 203304_at | 2.495445307 | 6.674293541 | 7.23813392 | 7.86E-10 | 2.31E-09 | 11.2537816 | BAMBI |
| 209301_at | -2.495311396 | 5.788158512 | -7.581131441 | 1.98E-10 | 6.23E-10 | 12.62784547 | CA2 |
| 222606_at | 2.495148606 | 6.607103704 | 17.88532825 | 2.59E-26 | 7.32E-25 | 49.3853131 | ZWILCH |
| 1563641_a_at | -2.494796346 | 5.109128358 | -24.0595316 | 2.28E-33 | 2.02E-31 | 65.72575542 | SNX20 |
| 225081_s_at | 2.494000112 | 6.158529584 | 9.375782079 | 1.52E-13 | 6.99E-13 | 19.80275834 | CDCA7L |
| 201204_s_at | 2.493949101 | 7.032062239 | 10.67639017 | 9.71E-16 | 5.82E-15 | 24.87706087 | RRBP1 |
| 213900_at | -2.492574629 | 5.619704759 | -12.71075442 | 5.21E-19 | 4.88E-18 | 32.44905291 | FAM189A2 |
| 211784_s_at | 2.490867881 | 9.689264575 | 20.2405311 | 3.43E-29 | 1.55E-27 | 56.05714582 | SRSF1 |
| 223112_s_at | -2.490429488 | 9.841270833 | -18.61500107 | 3.12E-27 | 1.03E-25 | 51.51596438 | NDUFB10 |
| 202539_s_at | 2.489168855 | 6.220991491 | 12.55046265 | 9.26E-19 | 8.40E-18 | 31.87066406 | HMGCR |
| 226272_at | 2.48881871 | 6.221246963 | 11.32524893 | 8.33E-17 | 5.73E-16 | 27.34502699 | RCAN3 |
| 224639_at | 2.488671954 | 7.561625565 | 20.44168613 | 2.00E-29 | 9.59E-28 | 56.60025171 | SPPL3 |
| 202326_at | 2.488565675 | 6.52784955 | 22.54394528 | 8.89E-32 | 6.19E-30 | 62.04480409 | EHMT2 |
| 225040_s_at | 2.486619762 | 6.352801914 | 22.00805085 | 3.40E-31 | 2.22E-29 | 60.69573552 | RPE |
| 209524_at | 2.486323595 | 5.964446977 | 7.900136882 | 5.49E-11 | 1.86E-10 | 13.9082175 | HDGFRP3 |
| 236649_at | 2.482427154 | 6.528215894 | 15.65232916 | 2.45E-23 | 4.35E-22 | 42.48300219 | DTWD1 |
| 207541_s_at | 2.482094561 | 7.285828455 | 22.64266634 | 6.96E-32 | 4.94E-30 | 62.29054558 | EXOSC10 |
| 205944_s_at | -2.480812848 | 5.356689389 | -19.60165864 | 1.95E-28 | 7.86E-27 | 54.30517754 | CLTCL1 |
| 203284_s_at | 2.480707967 | 6.052322195 | 13.60997087 | 2.21E-20 | 2.51E-19 | 35.63330067 | HS2ST1 |
| 218709_s_at | 2.480268672 | 6.669836556 | 20.63930111 | 1.18E-29 | 6.08E-28 | 57.12990121 | IFT52 |
| 206858_s_at | 2.477920076 | 9.486244312 | 15.27628309 | 8.25E-23 | 1.35E-21 | 41.26132846 | HOXC6 |
| 222939_s_at | -2.477799716 | 5.739770203 | -8.974291045 | 7.46E-13 | 3.16E-12 | 18.20880116 | SLC16A10 |
| 218901_at | 2.477133171 | 6.706567856 | 6.302791886 | 3.27E-08 | 8.02E-08 | 7.546508876 | PLSCR4 |
| 223275_at | 2.476263945 | 5.999700788 | 12.37466532 | 1.75E-18 | 1.52E-17 | 31.23261882 | PRMT6 |
| 229872_s_at | 2.475837629 | 6.065904882 | 8.171318423 | 1.85E-11 | 6.59E-11 | 14.99675673 | LOC100996740 |
| 202816_s_at | 2.472773972 | 6.102140608 | 15.8905404 | 1.15E-23 | 2.14E-22 | 43.24783018 | SS18 |
| 229337_at | -2.471994892 | 5.084244514 | -21.21065126 | 2.63E-30 | 1.51E-28 | 58.63988496 | USP2 |
| 201955_at | 2.470941141 | 7.801594019 | 14.77846847 | 4.22E-22 | 6.21E-21 | 39.61684758 | CCNC |
| 221203_s_at | 2.470097508 | 6.66165987 | 24.53462304 | 7.51E-34 | 7.30E-32 | 66.84063079 | YEATS2 |
| 222212_s_at | 2.468913372 | 7.998551876 | 24.53953622 | 7.43E-34 | 7.28E-32 | 66.85206667 | CERS2 |
| 218247_s_at | 2.468660198 | 5.964380153 | 12.94091016 | 2.30E-19 | 2.26E-18 | 33.27386231 | MEX3C |
| 201136_at | 2.46796063 | 8.402525872 | 13.13043966 | 1.18E-19 | 1.21E-18 | 33.94803272 | PLP2 |
| 222750_s_at | 2.467629868 | 6.786677143 | 10.75429553 | 7.21E-16 | 4.40E-15 | 25.17581646 | SRD5A3 |
| 204146_at | 2.467167489 | 5.416959865 | 10.41523858 | 2.64E-15 | 1.51E-14 | 23.87095866 | RAD51AP1 |
| 221773_at | 2.466766953 | 7.226476681 | 11.33455833 | 8.04E-17 | 5.55E-16 | 27.38009093 | ELK3 |
| 222608_s_at | 2.466305491 | 4.925506465 | 8.938413317 | 8.61E-13 | 3.61E-12 | 18.06588288 | ANLN |
| 203628_at | 2.463229492 | 7.051091721 | 11.22380352 | 1.22E-16 | 8.20E-16 | 26.96228537 | IGF1R |
| 229097_at | 2.463149918 | 6.969296271 | 8.57312573 | 3.69E-12 | 1.43E-11 | 16.60710537 | DIAPH3 |
| 228455_at | 2.46224394 | 6.063068278 | 9.158866401 | 3.59E-13 | 1.58E-12 | 18.94286623 | RP5-1074L1.4 |
| 209444_at | 2.45929248 | 6.567470796 | 19.27224025 | 4.87E-28 | 1.84E-26 | 53.38540091 | RAP1GDS1 |
| 226416_at | 2.458863298 | 5.825509612 | 18.55632626 | 3.69E-27 | 1.21E-25 | 51.3468061 | ERI1 |
| 213108_at | -2.455812574 | 5.258818447 | -28.00833815 | 3.69E-37 | 6.50E-35 | 74.48090368 | CAMK2A |
| 204472_at | 2.455250408 | 6.447772903 | 8.16513069 | 1.89E-11 | 6.75E-11 | 14.97192772 | GEM |
| 208002_s_at | 2.454914289 | 6.833549421 | 15.63602996 | 2.58E-23 | 4.56E-22 | 42.43041433 | ACOT7 |
| 224788_at | 2.454651557 | 8.180933064 | 17.81121242 | 3.22E-26 | 8.95E-25 | 49.16556644 | ARF6 |
| 224588_at | -2.453280114 | 7.852732265 | -2.404186449 | 0.019175337 | 0.025373781 | -5.25967605 | XIST |
| 215165_x_at | 2.452564307 | 6.441681347 | 19.9280599 | 7.98E-29 | 3.43E-27 | 55.20545055 | UMPS |
| 233893_s_at | 2.452498447 | 7.462840489 | 18.49327657 | 4.42E-27 | 1.42E-25 | 51.16461582 | UVSSA |
| 225222_at | 2.449692185 | 7.737813514 | 17.59329518 | 6.14E-26 | 1.64E-24 | 48.51584854 | HIAT1 |
| 237116_at | -2.448463144 | 4.509239919 | -13.12520614 | 1.20E-19 | 1.23E-18 | 33.92947808 | LOC646903 |
| 206052_s_at | 2.448382342 | 7.490541678 | 15.25144139 | 8.94E-23 | 1.45E-21 | 41.18000401 | SLBP |
| 203038_at | 2.448019612 | 6.81135443 | 8.463674421 | 5.72E-12 | 2.18E-11 | 16.16891777 | PTPRK |
| 209523_at | 2.447526418 | 7.400116487 | 18.03461304 | 1.67E-26 | 4.92E-25 | 49.82604848 | TAF2 |
| 214604_at | 2.447359459 | 6.669376079 | 8.156446953 | 1.96E-11 | 6.97E-11 | 14.93708217 | HOXD11 |
| 228206_at | 2.446786061 | 7.971754955 | 8.047722608 | 3.04E-11 | 1.05E-10 | 14.50071331 | HS3ST4 |
| 225898_at | 2.446548353 | 7.134088598 | 14.23045648 | 2.64E-21 | 3.42E-20 | 37.77029659 | WDR54 |
| 204808_s_at | 2.445723029 | 8.148272234 | 17.98731143 | 1.92E-26 | 5.57E-25 | 49.68667057 | TMEM5 |
| 203498_at | -2.444046676 | 6.085604322 | -10.28733533 | 4.33E-15 | 2.41E-14 | 23.37568157 | RCAN2 |
| 218025_s_at | -2.443922789 | 7.443139959 | -9.707038684 | 4.14E-14 | 2.04E-13 | 21.10929314 | ECI2 |
| 219922_s_at | 2.441660032 | 7.365678953 | 12.16788834 | 3.70E-18 | 3.09E-17 | 30.47720816 | LTBP3 |
| 222999_s_at | 2.440498905 | 9.027060231 | 13.97973422 | 6.19E-21 | 7.69E-20 | 36.9127189 | CCNL2 |
| 229655_at | 2.439414511 | 6.725127729 | 9.64248553 | 5.33E-14 | 2.60E-13 | 20.85535877 | FAM19A5 |
| 215016_x_at | -2.438976057 | 8.056509888 | -8.187949761 | 1.73E-11 | 6.19E-11 | 15.06348878 | DST |
| 228191_at | 2.438514339 | 5.484062386 | 13.93347942 | 7.25E-21 | 8.92E-20 | 36.75362984 | FLVCR1 |
| 218203_at | 2.438247645 | 7.778245451 | 19.58248774 | 2.06E-28 | 8.26E-27 | 54.25195975 | ALG5 |
| 222848_at | 2.437981143 | 4.249991061 | 9.60044681 | 6.28E-14 | 3.03E-13 | 20.68980904 | CENPK |
| 200920_s_at | 2.437164797 | 8.665137902 | 12.42399806 | 1.46E-18 | 1.29E-17 | 31.41205885 | BTG1 |
| 229553_at | 2.436451272 | 7.29462409 | 8.616690673 | 3.10E-12 | 1.21E-11 | 16.78139425 | PGM2L1 |
| 226979_at | 2.436321484 | 8.5649098 | 16.99860564 | 3.67E-25 | 8.71E-24 | 46.71497488 | MAP3K2 |
| 203148_s_at | 2.434544869 | 5.63526331 | 11.38278918 | 6.72E-17 | 4.69E-16 | 27.56159222 | TRIM14 |
| 1566558_x_at | 2.434236384 | 6.545565739 | 18.52976249 | 3.98E-27 | 1.30E-25 | 51.27009966 | BAIAP2-AS1 |
| 201037_at | 2.434011301 | 7.490215576 | 10.60983065 | 1.25E-15 | 7.42E-15 | 24.6213068 | PFKP |
| 225401_at | 2.433830594 | 6.500085434 | 18.99140206 | 1.07E-27 | 3.87E-26 | 52.59224858 | C1orf85 |
| 225802_at | 2.433456728 | 6.493841049 | 12.34799414 | 1.92E-18 | 1.67E-17 | 31.13548025 | TOP1MT |
| 201004_at | 2.432417723 | 9.605570561 | 20.49245395 | 1.74E-29 | 8.50E-28 | 56.73668721 | SSR4 |
| 226337_at | 2.432202865 | 5.649306096 | 17.58236727 | 6.34E-26 | 1.69E-24 | 48.48312445 | GORAB |
| 202185_at | 2.432078846 | 7.874289591 | 17.62152801 | 5.65E-26 | 1.51E-24 | 48.60032972 | PLOD3 |
| 243045_at | -2.431810377 | 5.725162261 | -23.84829688 | 3.76E-33 | 3.21E-31 | 65.22422884 | SMYD1 |
| 201614_s_at | 2.431632785 | 6.874804441 | 15.07201099 | 1.61E-22 | 2.49E-21 | 40.59030598 | RUVBL1 |
| 218641_at | 2.43084991 | 7.129620155 | 19.77325742 | 1.22E-28 | 5.10E-27 | 54.77984038 | C11orf95 |
| 201743_at | 2.430671583 | 6.807546176 | 7.299092751 | 6.15E-10 | 1.83E-09 | 11.49765956 | CD14 |
| 1553979_at | 2.430199529 | 7.866482393 | 18.15985513 | 1.16E-26 | 3.51E-25 | 50.19387448 | ZNF121 |
| 225073_at | 2.428697622 | 8.145698062 | 16.48254598 | 1.79E-24 | 3.81E-23 | 45.11857145 | PPHLN1 |
| 203245_s_at | 2.423051634 | 5.025988797 | 13.12040691 | 1.22E-19 | 1.25E-18 | 33.91246009 | LINC00094 |
| 1552628_a_at | 2.422678685 | 7.489976829 | 17.9007842 | 2.47E-26 | 7.04E-25 | 49.43106035 | HERPUD2 |
| 201118_at | 2.4224966 | 7.151508165 | 13.66245049 | 1.84E-20 | 2.11E-19 | 35.81595069 | PGD |
| 224796_at | 2.422010351 | 7.761108052 | 15.1539937 | 1.23E-22 | 1.95E-21 | 40.86024302 | ASAP1 |
| 212689_s_at | 2.421338893 | 7.992461849 | 11.83989077 | 1.23E-17 | 9.56E-17 | 29.26815313 | KDM3A |
| 208909_at | -2.421120296 | 10.20602855 | -21.18785941 | 2.79E-30 | 1.59E-28 | 58.5802493 | UQCRFS1 |
| 211623_s_at | 2.421029867 | 9.146260379 | 16.50516794 | 1.67E-24 | 3.58E-23 | 45.18921644 | FBL |
| 1565999_at | 2.419758148 | 5.076940227 | 5.964384528 | 1.24E-07 | 2.86E-07 | 6.230712255 | BX648501 |
| 226850_at | 2.419384433 | 7.192678037 | 21.18529396 | 2.81E-30 | 1.60E-28 | 58.57353362 | SUMF1 |
| 218277_s_at | 2.419117598 | 7.185428976 | 17.50129503 | 8.07E-26 | 2.11E-24 | 48.23992256 | DHX40 |
| 214830_at | 2.418644551 | 5.044931178 | 13.27553448 | 7.07E-20 | 7.50E-19 | 34.46105388 | SLC38A6 |
| 205771_s_at | 2.417993549 | 8.274077982 | 7.084979625 | 1.45E-09 | 4.13E-09 | 10.64189685 | AKAP7 |
| 212854_x_at | 2.417712989 | 9.716340594 | 15.6704817 | 2.31E-23 | 4.13E-22 | 42.54153105 | NBPF1 |
| 235729_at | 2.417254945 | 6.862075202 | 11.95915134 | 7.94E-18 | 6.34E-17 | 29.70928751 | ZNF514 |
| 228597_at | 2.415985343 | 5.28433494 | 13.73897145 | 1.41E-20 | 1.65E-19 | 36.08164262 | MIS18A |
| 222304_x_at | -2.415252289 | 5.311351664 | -22.385781 | 1.32E-31 | 9.03E-30 | 61.64930314 | OR7E47P |
| 202633_at | 2.415030617 | 6.955808756 | 18.02041708 | 1.74E-26 | 5.11E-25 | 49.78424533 | TOPBP1 |
| 212533_at | 2.414830118 | 6.897687487 | 15.64243304 | 2.53E-23 | 4.48E-22 | 42.4510772 | WEE1 |
| 218162_at | 2.414297954 | 7.91615048 | 6.963028849 | 2.37E-09 | 6.59E-09 | 10.1556951 | OLFML3 |
| 209284_s_at | 2.413356536 | 7.223026358 | 17.78276731 | 3.50E-26 | 9.65E-25 | 49.08106413 | FAM208A |
| 230384_at | -2.411137542 | 5.242103845 | -18.67276905 | 2.65E-27 | 8.81E-26 | 51.68214192 | ANKRD23 |
| 202797_at | 2.410964628 | 7.972084055 | 14.63345168 | 6.83E-22 | 9.70E-21 | 39.13191862 | SACM1L |
| 209109_s_at | 2.409635258 | 6.880895131 | 11.62023937 | 2.77E-17 | 2.05E-16 | 28.45119406 | TSPAN6 |
| 228984_at | -2.409576268 | 6.014631279 | -18.61308756 | 3.14E-27 | 1.03E-25 | 51.5104537 | CARNS1 |
| 214078_at | 2.407371788 | 5.153871283 | 7.352857335 | 4.96E-10 | 1.49E-09 | 11.71289084 | AF070581 |
| 201448_at | 2.406906206 | 8.353675806 | 14.63481743 | 6.80E-22 | 9.67E-21 | 39.13649806 | TIA1 |
| 202436_s_at | 2.40669593 | 6.679345157 | 6.524547286 | 1.36E-08 | 3.48E-08 | 8.417628145 | CYP1B1 |
| 204766_s_at | 2.405830054 | 6.74085694 | 14.13605242 | 3.64E-21 | 4.65E-20 | 37.4483367 | NUDT1 |
| 212956_at | 2.403566584 | 6.290611741 | 7.820163123 | 7.57E-11 | 2.52E-10 | 13.58714495 | TBC1D9 |
| 208694_at | 2.403563556 | 8.120892195 | 11.89008085 | 1.02E-17 | 8.03E-17 | 29.45401217 | PRKDC |
| 1564679_at | -2.403238054 | 4.685243271 | -25.07198214 | 2.19E-34 | 2.43E-32 | 68.08023599 | ASB15 |
| 218437_s_at | 2.40010912 | 5.545313094 | 13.96413683 | 6.53E-21 | 8.09E-20 | 36.85910369 | LZTFL1 |
| 226287_at | 2.398585726 | 6.219581572 | 13.67037765 | 1.79E-20 | 2.06E-19 | 35.84350975 | CCDC34 |
| 226479_at | 2.394357208 | 5.757902816 | 14.41858817 | 1.40E-21 | 1.90E-20 | 38.40851966 | KBTBD6 |
| 202741_at | 2.393479756 | 7.807668302 | 12.02144594 | 6.32E-18 | 5.12E-17 | 29.93902154 | PRKACB |
| 223046_at | -2.392101701 | 8.402286011 | -17.48426886 | 8.50E-26 | 2.21E-24 | 48.18875127 | EGLN1 |
| 221524_s_at | -2.39065025 | 7.828233398 | -11.25653157 | 1.08E-16 | 7.31E-16 | 27.08589351 | RRAGD |
| 229997_at | 2.390460921 | 6.338119174 | 15.30851471 | 7.43E-23 | 1.22E-21 | 41.36673039 | VANGL1 |
| 204900_x_at | 2.390237253 | 6.71007461 | 11.60082024 | 2.98E-17 | 2.19E-16 | 28.37869035 | SAP30 |
| 225188_at | 2.38959049 | 7.443918947 | 8.658800268 | 2.62E-12 | 1.04E-11 | 16.94978834 | RAPH1 |
| 225827_at | 2.389541604 | 7.352666842 | 11.39031416 | 6.53E-17 | 4.57E-16 | 27.58988571 | AGO2 |
| 204319_s_at | 2.389483382 | 6.998884653 | 11.10914513 | 1.88E-16 | 1.23E-15 | 26.52827881 | RGS10 |
| 227767_at | 2.388902123 | 7.113580231 | 15.91902365 | 1.05E-23 | 1.97E-22 | 43.33881472 | CSNK1G3 |
| 229796_at | -2.38751427 | 6.080312018 | -12.98334108 | 1.98E-19 | 1.96E-18 | 33.42518899 | SIX4 |
| 200990_at | 2.387220661 | 8.816311953 | 16.24097732 | 3.80E-24 | 7.65E-23 | 44.36034744 | TRIM28 |
| 232231_at | 2.387175904 | 5.326008679 | 6.489566046 | 1.56E-08 | 3.97E-08 | 8.279807276 | RUNX2 |
| 202478_at | 2.386172217 | 7.343758856 | 8.841450322 | 1.27E-12 | 5.20E-12 | 17.67928513 | TRIB2 |
| 214429_at | 2.386169735 | 7.613716116 | 12.08945382 | 4.93E-18 | 4.05E-17 | 30.18928318 | MTMR6 |
| 36553_at | 2.3854556 | 7.266186694 | 14.47711331 | 1.15E-21 | 1.59E-20 | 38.60614266 | ASMTL |
| 221502_at | -2.385389563 | 8.522671678 | -20.81880054 | 7.33E-30 | 3.88E-28 | 57.60768354 | KPNA3 |
| 202450_s_at | 2.385012253 | 7.910756868 | 6.807608996 | 4.41E-09 | 1.19E-08 | 9.537638017 | CTSK |
| 227601_at | 2.383419167 | 6.646498855 | 22.33722324 | 1.49E-31 | 1.00E-29 | 61.52743616 | METTL14 |
| 228776_at | 2.383071255 | 6.880285958 | 8.134455932 | 2.14E-11 | 7.57E-11 | 14.84883282 | GJC1 |
| 201721_s_at | 2.382165616 | 7.860673903 | 6.969105684 | 2.31E-09 | 6.44E-09 | 10.17989852 | LAPTM5 |
| 228843_at | 2.380879589 | 6.755715046 | 11.82366879 | 1.31E-17 | 1.01E-16 | 29.20801627 | ARL10 |
| 201010_s_at | -2.379293013 | 10.38326028 | -9.527118762 | 8.38E-14 | 3.98E-13 | 20.40070731 | TXNIP |
| 204224_s_at | 2.379255433 | 5.117194503 | 8.391653359 | 7.64E-12 | 2.86E-11 | 15.88036551 | GCH1 |
| 212247_at | 2.378937539 | 6.716988183 | 16.62592411 | 1.15E-24 | 2.54E-23 | 45.56528304 | NUP205 |
| 244623_at | -2.377407112 | 4.529349729 | -9.644836069 | 5.28E-14 | 2.57E-13 | 20.86461108 | KCNQ5 |
| 212900_at | 2.376318207 | 7.160834268 | 20.2142709 | 3.68E-29 | 1.65E-27 | 55.98594721 | SEC24A |
| 209101_at | 2.376282868 | 7.066766385 | 5.471650646 | 8.30E-07 | 1.76E-06 | 4.352703204 | CTGF |
| 215071_s_at | 2.376018798 | 8.236949934 | 6.305030851 | 3.25E-08 | 7.95E-08 | 7.555272125 | HIST1H2AC |
| 241937_s_at | 2.375630962 | 5.607034898 | 11.51317057 | 4.13E-17 | 2.97E-16 | 28.05088397 | WDR4 |
| 220731_s_at | 2.375554489 | 6.773985768 | 26.44378329 | 1.03E-35 | 1.45E-33 | 71.14624637 | NECAP2 |
| 225367_at | 2.374109034 | 5.818493184 | 17.5588282 | 6.80E-26 | 1.79E-24 | 48.41258921 | PGM2 |
| 213491_x_at | 2.373782941 | 9.729601705 | 16.90596915 | 4.87E-25 | 1.13E-23 | 46.43073337 | RPN2 |
| 213253_at | 2.371948634 | 5.989750171 | 13.30554349 | 6.36E-20 | 6.81E-19 | 34.56682343 | SMC2 |
| 202370_s_at | 2.371835493 | 7.77629344 | 11.88115029 | 1.06E-17 | 8.28E-17 | 29.42096371 | CBFB |
| 225105_at | -2.371476355 | 6.112941521 | -8.077128844 | 2.70E-11 | 9.43E-11 | 14.61875003 | C12orf75 |
| 217080_s_at | -2.371365387 | 5.484261288 | -13.44242559 | 3.95E-20 | 4.34E-19 | 35.04781813 | HOMER2 |
| 229116_at | -2.370213353 | 3.961755602 | -10.05914291 | 1.05E-14 | 5.55E-14 | 22.4881198 | CNKSR2 |
| 207537_at | -2.369484124 | 4.950252169 | -24.53466419 | 7.51E-34 | 7.30E-32 | 66.84072657 | PFKFB1 |
| 200801_x_at | 2.369186163 | 12.15792244 | 24.1129804 | 2.01E-33 | 1.80E-31 | 65.85208332 | ACTB |
| 209433_s_at | 2.369082652 | 6.278962149 | 12.04934418 | 5.70E-18 | 4.65E-17 | 30.04175262 | PPAT |
| 209642_at | 2.36775646 | 5.048729135 | 8.965547102 | 7.73E-13 | 3.26E-12 | 18.17397643 | BUB1 |
| 221734_at | 2.36622215 | 7.276025158 | 18.74957276 | 2.13E-27 | 7.26E-26 | 51.90251718 | PRRC1 |
| 1553415_at | 2.366152777 | 4.669022311 | 3.88787007 | 0.000246775 | 0.000402858 | -1.19383923 | SLC17A8 |
| 201331_s_at | 2.365872951 | 8.096769863 | 15.45072019 | 4.69E-23 | 8.00E-22 | 41.83021482 | STAT6 |
| 204851_s_at | 2.365198284 | 4.854740232 | 4.832396496 | 9.07E-06 | 1.72E-05 | 2.006678185 | DCX |
| 221704_s_at | 2.365107658 | 6.477152656 | 13.44556212 | 3.90E-20 | 4.30E-19 | 35.05881163 | VPS37B |
| 205932_s_at | 2.363810904 | 6.838330185 | 6.500686117 | 1.50E-08 | 3.81E-08 | 8.323603197 | MSX1 |
| 202720_at | 2.362987609 | 7.078415845 | 11.02166194 | 2.61E-16 | 1.68E-15 | 26.19613935 | TES |
| 212757_s_at | -2.362693707 | 6.884889356 | -20.92193524 | 5.59E-30 | 3.02E-28 | 57.88078772 | CAMK2G |
| 211048_s_at | 2.362094064 | 7.002805205 | 19.7712941 | 1.23E-28 | 5.11E-27 | 54.77442675 | PDIA4 |
| 216331_at | -2.361578753 | 6.787278228 | -11.27810079 | 9.94E-17 | 6.77E-16 | 27.16728962 | ITGA7 |
| 202834_at | -2.356942933 | 5.364125645 | -10.6722868 | 9.86E-16 | 5.91E-15 | 24.86130718 | AGT |
| 224480_s_at | -2.356744731 | 5.115933209 | -10.05233168 | 1.08E-14 | 5.69E-14 | 22.46155203 | AGPAT9 |
| 209560_s_at | -2.355680487 | 5.457758367 | -5.337470311 | 1.38E-06 | 2.86E-06 | 3.850801676 | DLK1 |
| 202596_at | 2.354537027 | 8.21419485 | 21.78249485 | 6.03E-31 | 3.82E-29 | 60.12017176 | ENSA |
| 217823_s_at | 2.35301382 | 6.685861512 | 15.13211727 | 1.32E-22 | 2.08E-21 | 40.788295 | UBE2J1 |
| 221599_at | -2.352390987 | 7.647884203 | -20.75141976 | 8.76E-30 | 4.58E-28 | 57.42870059 | AAMDC |
| 203554_x_at | 2.352278467 | 7.765072562 | 10.71688272 | 8.31E-16 | 5.03E-15 | 25.03242438 | PTTG1 |
| 236033_at | -2.352061153 | 6.273339475 | -24.19248329 | 1.67E-33 | 1.52E-31 | 66.03956509 | ASB12 |
| 204019_s_at | 2.351123336 | 7.780146302 | 12.42822782 | 1.44E-18 | 1.27E-17 | 31.42742979 | SH3YL1 |
| 225364_at | 2.35034642 | 7.228102231 | 16.61675131 | 1.18E-24 | 2.59E-23 | 45.5367777 | STK4 |
| 202803_s_at | 2.350099377 | 6.762617349 | 7.19044271 | 9.51E-10 | 2.77E-09 | 11.0631085 | ITGB2 |
| 228396_at | -2.348827711 | 7.087736168 | -10.37623841 | 3.07E-15 | 1.74E-14 | 23.72011123 | PRKG1 |
| 208919_s_at | 2.348197158 | 7.002321223 | 18.70703075 | 2.40E-27 | 8.09E-26 | 51.78052909 | NADK |
| 222988_s_at | 2.346730353 | 7.988470875 | 16.35299814 | 2.68E-24 | 5.52E-23 | 44.71282809 | TMEM9 |
| 228654_at | 2.346647473 | 4.746890069 | 14.05880802 | 4.73E-21 | 5.97E-20 | 37.18405225 | SPIN4 |
| 209189_at | 2.346470056 | 7.707755115 | 4.460486224 | 3.46E-05 | 6.19E-05 | 0.702741689 | FOS |
| 213194_at | 2.342333917 | 7.335107775 | 7.728398221 | 1.10E-10 | 3.56E-10 | 13.21877951 | ROBO1 |
| 213048_s_at | 2.342260155 | 11.03607147 | 13.5344299 | 2.87E-20 | 3.22E-19 | 35.36976925 | Y16709 |
| 206028_s_at | 2.342034997 | 6.358192123 | 7.239245227 | 7.82E-10 | 2.30E-09 | 11.25822605 | MERTK |
| 209185_s_at | 2.340193512 | 10.11421913 | 8.251252246 | 1.34E-11 | 4.87E-11 | 15.31743923 | IRS2 |
| 231698_at | -2.339247893 | 4.608707907 | -21.49653296 | 1.25E-30 | 7.55E-29 | 59.3837402 | FLJ36848 |
| 225353_s_at | 2.338550862 | 6.691468238 | 6.934679893 | 2.65E-09 | 7.33E-09 | 10.04281966 | C1QC |
| 1564281_at | -2.338231613 | 6.930953933 | -20.31327274 | 2.82E-29 | 1.31E-27 | 56.25400853 | LINC00491 |
| 214257_s_at | 2.337921769 | 8.200608582 | 15.33425352 | 6.83E-23 | 1.13E-21 | 41.4508067 | SEC22B |
| 203417_at | 2.337346065 | 6.686216696 | 6.983573895 | 2.18E-09 | 6.09E-09 | 10.23753458 | MFAP2 |
| 229632_s_at | 2.336803598 | 7.269770681 | 17.04297119 | 3.21E-25 | 7.66E-24 | 46.85074669 | INTS10 |
| 202561_at | 2.336698372 | 7.768075881 | 11.17978244 | 1.44E-16 | 9.56E-16 | 26.7958326 | TNKS |
| 218109_s_at | 2.334898733 | 7.709356266 | 12.35231587 | 1.89E-18 | 1.64E-17 | 31.15122633 | MFSD1 |
| 202941_at | -2.33447693 | 9.276884507 | -17.2844325 | 1.55E-25 | 3.87E-24 | 47.5856539 | NDUFV2 |
| 225232_at | 2.333775943 | 7.016788381 | 18.90940581 | 1.35E-27 | 4.80E-26 | 52.35908786 | MTMR12 |
| 222938_x_at | 2.333676122 | 7.104444055 | 7.486578521 | 2.90E-10 | 8.95E-10 | 12.24867372 | ENPP3 |
| 222231_s_at | 2.330222871 | 7.092110874 | 18.46150654 | 4.85E-27 | 1.54E-25 | 51.0726472 | LRRC59 |
| 209682_at | 2.329726087 | 6.976132775 | 13.16936389 | 1.03E-19 | 1.06E-18 | 34.08592313 | CBLB |
| 203133_at | 2.329407762 | 9.489227841 | 25.1153853 | 1.98E-34 | 2.23E-32 | 68.17938872 | SEC61B |
| 204641_at | 2.32926525 | 5.032370104 | 8.727174125 | 2.00E-12 | 7.99E-12 | 17.2230509 | NEK2 |
| 205596_s_at | 2.329137696 | 6.503935086 | 10.93078363 | 3.69E-16 | 2.33E-15 | 25.85020773 | SMURF2 |
| 224707_at | -2.328689348 | 8.669542518 | -12.98530035 | 1.96E-19 | 1.95E-18 | 33.43217108 | CYSTM1 |
| 230669_at | 2.328674752 | 7.262561268 | 14.0138296 | 5.51E-21 | 6.90E-20 | 37.02981159 | RASA2 |
| 204135_at | -2.32765475 | 6.361352135 | -7.308784533 | 5.92E-10 | 1.76E-09 | 11.53644889 | FILIP1L |
| 1553703_at | 2.327223696 | 7.679475777 | 18.84067057 | 1.64E-27 | 5.73E-26 | 52.1630797 | ZNF791 |
| 201555_at | 2.326572219 | 7.361567214 | 14.30650483 | 2.04E-21 | 2.70E-20 | 38.02882831 | MCM3 |
| 203228_at | 2.325782634 | 7.308690092 | 12.98492711 | 1.97E-19 | 1.95E-18 | 33.43084102 | PAFAH1B3 |
| 203028_s_at | 2.322841631 | 7.698564638 | 12.82339293 | 3.49E-19 | 3.33E-18 | 32.85355348 | CYBA |
| 224759_s_at | 2.322438688 | 7.422158205 | 13.94818282 | 6.89E-21 | 8.51E-20 | 36.80423048 | TMEM263 |
| 226329_s_at | 2.322052845 | 5.503719062 | 12.10541301 | 4.65E-18 | 3.84E-17 | 30.24792906 | MITD1 |
| 226143_at | 2.320673697 | 7.266881538 | 17.23506968 | 1.79E-25 | 4.44E-24 | 47.43596681 | RAI1 |
| 203415_at | 2.320442594 | 7.385394943 | 23.58946943 | 6.96E-33 | 5.69E-31 | 64.60472337 | PDCD6 |
| 218744_s_at | -2.320436488 | 5.473780388 | -14.59407618 | 7.79E-22 | 1.10E-20 | 38.9997887 | PACSIN3 |
| 218418_s_at | 2.320043529 | 8.006579213 | 10.21212797 | 5.79E-15 | 3.17E-14 | 23.08370861 | KANK2 |
| 201948_at | 2.317551575 | 7.041688054 | 15.88461942 | 1.17E-23 | 2.17E-22 | 43.22890421 | GNL2 |
| 242821_at | 2.317505556 | 6.266729221 | 6.754071408 | 5.46E-09 | 1.46E-08 | 9.32520259 | CCDC171 |
| 222116_s_at | 2.31748721 | 6.611703191 | 17.87143111 | 2.70E-26 | 7.61E-25 | 49.34415667 | TBC1D16 |
| 206812_at | 2.317066322 | 6.788328774 | 7.744724483 | 1.03E-10 | 3.36E-10 | 13.2843112 | ADRB3 |
| 225164_s_at | 2.316828192 | 6.825195681 | 17.22848884 | 1.83E-25 | 4.52E-24 | 47.41598976 | EIF2AK4 |
| 227931_at | 2.314617048 | 6.94502542 | 14.96994792 | 2.24E-22 | 3.42E-21 | 40.25307173 | INO80D |
| 227803_at | -2.314277095 | 3.510399009 | -11.08867934 | 2.03E-16 | 1.32E-15 | 26.45065509 | ENPP5 |
| 219317_at | 2.313522086 | 5.327539126 | 10.37644858 | 3.07E-15 | 1.74E-14 | 23.72092454 | POLI |
| 227776_at | 2.311906772 | 6.02587265 | 12.97270998 | 2.05E-19 | 2.03E-18 | 33.38729545 | ACER3 |
| 226682_at | -2.310031332 | 6.929770492 | -7.822209914 | 7.51E-11 | 2.50E-10 | 13.59536196 | RORA |
| 224428_s_at | 2.309278954 | 5.935855548 | 6.925761373 | 2.75E-09 | 7.59E-09 | 10.00732165 | CDCA7 |
| 228628_at | 2.309066393 | 6.493902412 | 16.77375105 | 7.30E-25 | 1.66E-23 | 46.02328663 | SRGAP2C |
| 204112_s_at | 2.308556659 | 6.911896025 | 7.472962853 | 3.06E-10 | 9.42E-10 | 12.19409274 | HNMT |
| 225029_at | 2.308398395 | 7.64356999 | 21.82367556 | 5.43E-31 | 3.47E-29 | 60.22560192 | LINC01420 |
| 223513_at | 2.30824352 | 5.552079162 | 13.32018779 | 6.04E-20 | 6.49E-19 | 34.61839687 | CENPJ |
| 220532_s_at | 2.307805477 | 6.969252534 | 7.395611219 | 4.17E-10 | 1.26E-09 | 11.88412581 | TMEM176B |
| 205690_s_at | 2.306551815 | 7.480725841 | 20.17238228 | 4.12E-29 | 1.85E-27 | 55.87223267 | BUD31 |
| 224657_at | 2.304709951 | 7.393459095 | 9.290178762 | 2.13E-13 | 9.65E-13 | 19.46379447 | ERRFI1 |
| 235048_at | 2.303851521 | 5.151518397 | 9.834229693 | 2.51E-14 | 1.27E-13 | 21.60861402 | FAM169A |
| 202690_s_at | 2.303839568 | 8.01763053 | 13.93654695 | 7.17E-21 | 8.83E-20 | 36.76418879 | SNRPD1 |
| 225045_at | 2.302589248 | 6.312910656 | 13.29210149 | 6.67E-20 | 7.10E-19 | 34.51946004 | CCDC88A |
| 223002_s_at | 2.300999345 | 7.016391001 | 17.15660031 | 2.27E-25 | 5.55E-24 | 47.19743374 | XRN2 |
| 204881_s_at | 2.300137831 | 7.038985331 | 11.87599555 | 1.08E-17 | 8.43E-17 | 29.40188367 | UGCG |
| 226431_at | 2.298825668 | 5.455122955 | 12.26226697 | 2.63E-18 | 2.24E-17 | 30.82265584 | FAM117B |
| 226633_at | 2.298464241 | 5.95393032 | 12.63884305 | 6.75E-19 | 6.22E-18 | 32.18997151 | RAB8B |
| 1556507_at | -2.298122163 | 7.945585877 | -31.03613686 | 9.06E-40 | 2.39E-37 | 80.49321754 | LINC01210 |
| 219306_at | 2.298042096 | 5.588951561 | 8.760454756 | 1.75E-12 | 7.05E-12 | 17.35598416 | KIF15 |
| 204067_at | -2.296854526 | 6.047872124 | -15.62981315 | 2.64E-23 | 4.64E-22 | 42.41034772 | SUOX |
| 224893_at | 2.296334999 | 8.158977114 | 13.77323032 | 1.26E-20 | 1.48E-19 | 36.2003516 | ATL3 |
| 221918_at | 2.295662923 | 8.136547429 | 15.17567514 | 1.14E-22 | 1.83E-21 | 40.93149054 | CDK17 |
| 238054_at | -2.295031129 | 6.432033393 | -16.05418482 | 6.84E-24 | 1.31E-22 | 43.76920882 | ADPRHL1 |
| 202251_at | 2.294851431 | 6.552813599 | 16.90280001 | 4.92E-25 | 1.14E-23 | 46.42099143 | PRPF3 |
| 235542_at | 2.293899264 | 5.530308396 | 13.29474355 | 6.61E-20 | 7.04E-19 | 34.52877127 | TET3 |
| 225579_at | 2.293046958 | 6.208643731 | 9.621461859 | 5.79E-14 | 2.81E-13 | 20.77258457 | PQLC3 |
| 225098_at | 2.291906741 | 8.066915643 | 16.09861072 | 5.95E-24 | 1.15E-22 | 43.91018726 | ABI2 |
| 212596_s_at | 2.291781053 | 7.142788895 | 22.82285161 | 4.47E-32 | 3.32E-30 | 62.73687539 | HMGXB4 |
| 202151_s_at | -2.291562803 | 7.624439 | -21.88500057 | 4.65E-31 | 3.00E-29 | 60.38231717 | UBAC1 |
| 206159_at | 2.289694141 | 5.991343742 | 3.917210233 | 0.000223867 | 0.000367204 | -1.100356207 | GDF10 |
| 231897_at | 2.289693194 | 7.309451658 | 12.20388567 | 3.25E-18 | 2.73E-17 | 30.60909668 | PTGR1 |
| 224772_at | 2.288200623 | 7.356304697 | 10.88705484 | 4.35E-16 | 2.73E-15 | 25.68342821 | NAV1 |
| 230078_at | 2.287976372 | 6.92878834 | 12.9636868 | 2.12E-19 | 2.09E-18 | 33.35512193 | RAPGEF6 |
| 222995_s_at | 2.287087038 | 7.747869832 | 13.09500353 | 1.33E-19 | 1.36E-18 | 33.82233117 | RHBDD2 |
| 213101_s_at | 2.287020474 | 9.697619561 | 19.15685258 | 6.73E-28 | 2.49E-26 | 53.06053159 | ACTR3 |
| 226771_at | 2.285950943 | 6.123214419 | 14.88422264 | 2.98E-22 | 4.46E-21 | 39.9688066 | ATP8B2 |
| 203739_at | 2.285924618 | 5.554512805 | 9.077416577 | 4.96E-13 | 2.15E-12 | 18.61918942 | ZNF217 |
| 202081_at | 2.285314559 | 8.439833033 | 9.158224971 | 3.60E-13 | 1.59E-12 | 18.94031885 | IER2 |
| 225621_at | 2.284440353 | 7.179482114 | 18.94769859 | 1.21E-27 | 4.33E-26 | 52.46806505 | ALG2 |
| 227934_at | 2.284119901 | 7.121817373 | 14.5104547 | 1.03E-21 | 1.43E-20 | 38.71853238 | KPNA5 |
| 204424_s_at | 2.283738902 | 6.676038331 | 5.283545273 | 1.69E-06 | 3.47E-06 | 3.650400328 | LMO3 |
| 212946_at | -2.282174886 | 7.111900951 | -12.36602694 | 1.80E-18 | 1.57E-17 | 31.20116683 | VWA8 |
| 224002_s_at | 2.282055567 | 5.963261041 | 13.96012416 | 6.62E-21 | 8.19E-20 | 36.84530535 | FKBP7 |
| 212765_at | 2.281746761 | 7.509407613 | 14.81078572 | 3.79E-22 | 5.61E-21 | 39.72455216 | CAMSAP2 |
| 200675_at | 2.281514845 | 9.05305778 | 20.64162736 | 1.17E-29 | 6.06E-28 | 57.13611322 | CD81 |
| 229241_at | -2.27897549 | 4.138784544 | -32.05682752 | 1.34E-40 | 3.82E-38 | 82.40127031 | LDHD |
| 206309_at | 2.278633043 | 7.233108693 | 6.215349604 | 4.62E-08 | 1.11E-07 | 7.204817805 | LECT1 |
| 204630_s_at | 2.275432602 | 7.818660324 | 26.65222015 | 6.54E-36 | 9.51E-34 | 71.60019346 | GOSR1 |
| 228268_at | -2.274875219 | 6.036510358 | -7.883215934 | 5.88E-11 | 1.98E-10 | 13.84028333 | FMO2 |
| 203455_s_at | 2.274620275 | 8.590302903 | 8.221062025 | 1.51E-11 | 5.46E-11 | 15.19633479 | SAT1 |
| 204345_at | 2.273665648 | 7.219553336 | 7.813686933 | 7.77E-11 | 2.58E-10 | 13.56114591 | COL16A1 |
| 219647_at | -2.272229676 | 4.742800073 | -16.84393149 | 5.88E-25 | 1.36E-23 | 46.23981403 | POPDC2 |
| 212055_at | 2.271844973 | 6.793886524 | 13.84632685 | 9.77E-21 | 1.17E-19 | 36.4531332 | TPGS2 |
| 224511_s_at | 2.271828459 | 8.107054486 | 18.46615234 | 4.78E-27 | 1.53E-25 | 51.08610288 | TXNDC17 |
| 222101_s_at | 2.271505542 | 7.264402187 | 9.783861159 | 3.06E-14 | 1.54E-13 | 21.41104295 | DCHS1 |
| 204215_at | 2.271290174 | 5.501090744 | 12.77775232 | 4.11E-19 | 3.89E-18 | 32.68984496 | TMEM243 |
| 205483_s_at | 2.269609718 | 7.412456132 | 7.201355488 | 9.11E-10 | 2.65E-09 | 11.1067284 | ISG15 |
| 200777_s_at | 2.269473878 | 8.388668424 | 20.41848501 | 2.12E-29 | 1.01E-27 | 56.53781522 | BZW1 |
| 235067_at | 2.267767014 | 6.705306043 | 13.15419688 | 1.08E-19 | 1.12E-18 | 34.03221644 | MKLN1 |
| 203693_s_at | 2.267192427 | 6.891101327 | 13.30917248 | 6.28E-20 | 6.73E-19 | 34.57960634 | E2F3 |
| 224715_at | 2.266926648 | 6.736610684 | 14.34773408 | 1.78E-21 | 2.38E-20 | 38.16868179 | WDR34 |
| 204844_at | 2.266714176 | 5.985296931 | 6.214378288 | 4.64E-08 | 1.12E-07 | 7.201028506 | ENPEP |
| 202642_s_at | 2.266248197 | 6.605917412 | 18.69911258 | 2.45E-27 | 8.24E-26 | 51.75780229 | TRRAP |
| 201923_at | 2.265852586 | 8.365035898 | 13.18299463 | 9.78E-20 | 1.01E-18 | 34.13416484 | PRDX4 |
| 228435_at | -2.264530718 | 5.631701235 | -12.78599679 | 3.99E-19 | 3.79E-18 | 32.7194366 | AC005523.2 |
| 224810_s_at | 2.263167805 | 7.538612141 | 17.68198275 | 4.72E-26 | 1.29E-24 | 48.78092235 | ANKRD13A |
| 229520_s_at | 2.262786469 | 6.439237293 | 17.85851895 | 2.80E-26 | 7.87E-25 | 49.30589767 | GPATCH2L |
| 218288_s_at | 2.262404732 | 7.983270671 | 11.66513707 | 2.35E-17 | 1.75E-16 | 28.61865355 | CCDC90B |
| 227964_at | 2.261947937 | 6.489853347 | 18.80812081 | 1.80E-27 | 6.23E-26 | 52.07008209 | FRMD8 |
| 208682_s_at | 2.258684762 | 7.857937659 | 16.1895826 | 4.47E-24 | 8.88E-23 | 44.19812231 | MAGED2 |
| 202166_s_at | -2.258089243 | 7.28323619 | -12.15816501 | 3.83E-18 | 3.19E-17 | 30.44155597 | PPP1R2 |
| 239413_at | 2.257492294 | 5.464688949 | 10.55095278 | 1.57E-15 | 9.20E-15 | 24.39468382 | CEP152 |
| 236331_at | 2.256806211 | 5.269819907 | 5.741512565 | 2.94E-07 | 6.53E-07 | 5.37499436 | CDKL2 |
| 220147_s_at | 2.25673277 | 6.988439718 | 9.429532811 | 1.23E-13 | 5.72E-13 | 20.01532811 | FAM60A |
| 207725_at | 2.256144748 | 4.838035455 | 4.360005737 | 4.93E-05 | 8.67E-05 | 0.359556973 | POU4F2 |
| 1553074_at | -2.2550399 | 3.912102628 | -17.62878972 | 5.53E-26 | 1.48E-24 | 48.62204421 | ASB11 |
| 201931_at | -2.254521312 | 8.420106273 | -10.91017659 | 3.98E-16 | 2.51E-15 | 25.77163956 | ETFA |
| 200915_x_at | -2.254479085 | 9.618382061 | -15.1340581 | 1.31E-22 | 2.07E-21 | 40.79468051 | KTN1 |
| 215089_s_at | 2.254429947 | 7.211329288 | 24.50359793 | 8.07E-34 | 7.80E-32 | 66.76837302 | RBM10 |
| 218052_s_at | 2.254406756 | 6.667901333 | 19.02580095 | 9.72E-28 | 3.53E-26 | 52.68984957 | ATP13A1 |
| 218518_at | 2.251637007 | 6.47677656 | 13.02761798 | 1.69E-19 | 1.69E-18 | 33.58285536 | FAM13B |
| 201121_s_at | 2.251483234 | 8.235948933 | 13.1919335 | 9.47E-20 | 9.87E-19 | 34.16578828 | PGRMC1 |
| 202603_at | 2.250963619 | 8.063302002 | 15.30252691 | 7.58E-23 | 1.25E-21 | 41.34715924 | ADAM10 |
| 214687_x_at | -2.250683885 | 11.5169094 | -16.20401659 | 4.27E-24 | 8.51E-23 | 44.24371491 | ALDOA |
| 1552701_a_at | 2.250364523 | 5.286177473 | 8.154502758 | 1.98E-11 | 7.02E-11 | 14.92928046 | CARD16 |
| 219525_at | -2.249989871 | 5.156625066 | -12.00303842 | 6.76E-18 | 5.45E-17 | 29.87118644 | SLC47A1 |
| 226811_at | -2.249477005 | 5.083283212 | -7.873155274 | 6.12E-11 | 2.06E-10 | 13.79989198 | FAM46C |
| 1553954_at | 2.248011668 | 5.603614367 | 14.76765061 | 4.38E-22 | 6.41E-21 | 39.58076511 | ALG14 |
| 212812_at | 2.247639173 | 6.916089683 | 11.96508672 | 7.77E-18 | 6.21E-17 | 29.7311968 | SERINC5 |
| 202522_at | 2.247175874 | 8.664151058 | 18.99706675 | 1.05E-27 | 3.81E-26 | 52.60832989 | PITPNB |
| 226311_at | 2.247027991 | 6.349658799 | 6.527962643 | 1.34E-08 | 3.44E-08 | 8.431091741 | ADAMTS2 |
| 217758_s_at | 2.245177203 | 7.950631578 | 17.66471042 | 4.97E-26 | 1.35E-24 | 48.72936837 | TM9SF3 |
| 228728_at | -2.24512529 | 7.004916838 | -8.392034665 | 7.63E-12 | 2.85E-11 | 15.88189362 | CPED1 |
| 1559280_a_at | 2.244687086 | 5.034230148 | 4.830390495 | 9.14E-06 | 1.73E-05 | 1.999512319 | APELA |
| 225706_at | 2.243939367 | 6.192962401 | 8.381525318 | 7.95E-12 | 2.97E-11 | 15.83977514 | GLCCI1 |
| 1554616_at | -2.243602438 | 5.237546574 | -23.69978651 | 5.35E-33 | 4.44E-31 | 64.86944344 | SERPINB8 |
| 218652_s_at | 2.242775525 | 6.96619202 | 15.94629231 | 9.62E-24 | 1.81E-22 | 43.42582635 | PIGG |
| 225556_at | 2.242701899 | 7.671327308 | 16.89174266 | 5.08E-25 | 1.18E-23 | 46.38699188 | VMA21 |
| 228189_at | 2.241814345 | 7.615485821 | 13.31640262 | 6.12E-20 | 6.57E-19 | 34.60506911 | BAG4 |
| 212038_s_at | -2.241072803 | 9.314979787 | -12.86754265 | 2.98E-19 | 2.88E-18 | 33.01166342 | VDAC1 |
| 218532_s_at | -2.240353853 | 7.236534035 | -7.787567903 | 8.63E-11 | 2.85E-10 | 13.45629227 | FAM134B |
| 209460_at | 2.240276287 | 5.457403496 | 8.741961982 | 1.88E-12 | 7.55E-12 | 17.28212455 | ABAT |
| 228440_at | -2.238845038 | 3.64049921 | -20.43420274 | 2.04E-29 | 9.76E-28 | 56.580119 | RP11-164P12.4 |
| 212568_s_at | -2.23779679 | 7.252956871 | -13.79769271 | 1.15E-20 | 1.36E-19 | 36.28502326 | DLAT |
| 219013_at | 2.23729555 | 8.070668125 | 15.44016495 | 4.85E-23 | 8.26E-22 | 41.79589911 | GALNT11 |
| 225273_at | 2.236800975 | 7.157700956 | 13.48478703 | 3.40E-20 | 3.78E-19 | 35.1961882 | WWC3 |
| 234672_s_at | 2.235593398 | 7.352251381 | 13.73254177 | 1.44E-20 | 1.68E-19 | 36.05934667 | NDC1 |
| 202441_at | 2.235419827 | 7.443659564 | 17.6868191 | 4.65E-26 | 1.27E-24 | 48.79535166 | ERLIN1 |
| 208978_at | 2.233192895 | 7.362931732 | 7.809777779 | 7.90E-11 | 2.62E-10 | 13.54545251 | CRIP2 |
| 207165_at | 2.23268186 | 5.55885698 | 9.897167634 | 1.97E-14 | 1.01E-13 | 21.85518089 | HMMR |
| 200052_s_at | 2.230072015 | 8.074901691 | 15.09595834 | 1.48E-22 | 2.32E-21 | 40.6692425 | ILF2 |
| 225724_at | 2.22993198 | 6.486898712 | 10.31909417 | 3.83E-15 | 2.15E-14 | 23.49881161 | FLJ31306 |
| 225470_at | 2.229375767 | 6.799493483 | 12.83762243 | 3.32E-19 | 3.18E-18 | 32.90453944 | NUP35 |
| 206001_at | 2.229148373 | 7.236615934 | 4.674505858 | 1.61E-05 | 2.99E-05 | 1.446918166 | NPY |
| 225640_at | 2.228389933 | 8.041699283 | 17.87948917 | 2.63E-26 | 7.44E-25 | 49.36802328 | EBLN3 |
| 201319_at | -2.224858294 | 9.395857872 | -14.89175501 | 2.90E-22 | 4.37E-21 | 39.99382107 | MYL12A |
| 204244_s_at | 2.224604096 | 5.446050025 | 10.40599265 | 2.74E-15 | 1.56E-14 | 23.8352104 | DBF4 |
| 220244_at | -2.224018481 | 5.312374194 | -11.64943496 | 2.49E-17 | 1.85E-16 | 28.56011511 | LINC00312 |
| 201730_s_at | 2.222496836 | 8.979661525 | 16.97809533 | 3.91E-25 | 9.22E-24 | 46.65212907 | TPR |
| 224367_at | 2.222008967 | 6.268849961 | 5.602247517 | 5.03E-07 | 1.09E-06 | 4.845392685 | BEX2 |
| 225221_at | 2.220723374 | 8.234198899 | 13.92204863 | 7.54E-21 | 9.23E-20 | 36.71427255 | ZKSCAN1 |
| 206240_s_at | 2.220517746 | 6.480252552 | 17.9768528 | 1.98E-26 | 5.71E-25 | 49.65581948 | ZNF136 |
| 218205_s_at | -2.220276251 | 10.10418548 | -20.25019219 | 3.34E-29 | 1.52E-27 | 56.08332234 | MKNK2 |
| 208736_at | 2.217909816 | 8.85265442 | 18.87831115 | 1.47E-27 | 5.23E-26 | 52.27047997 | ARPC3 |
| 224812_at | -2.216929551 | 7.227230318 | -12.45089317 | 1.33E-18 | 1.18E-17 | 31.5097576 | HIBADH |
| 213229_at | 2.216876574 | 9.496909118 | 16.2465793 | 3.74E-24 | 7.52E-23 | 44.37801049 | DICER1 |
| 212640_at | 2.215578149 | 6.663800699 | 14.89561769 | 2.87E-22 | 4.32E-21 | 40.00664599 | PTPLB |
| 203024_s_at | 2.215511375 | 8.301763159 | 20.51973861 | 1.62E-29 | 8.01E-28 | 56.80990803 | C5orf15 |
| 208923_at | 2.215257348 | 8.340805546 | 17.98065565 | 1.96E-26 | 5.66E-25 | 49.66703862 | CYFIP1 |
| 226932_at | -2.214807886 | 7.281858402 | -6.76689796 | 5.18E-09 | 1.39E-08 | 9.37607452 | SSPN |
| 211779_x_at | 2.213310501 | 7.762428794 | 16.15710798 | 4.95E-24 | 9.72E-23 | 44.09545237 | AP2A2 |
| 225479_at | 2.2128126 | 7.184541982 | 20.42358148 | 2.10E-29 | 1.00E-27 | 56.5515349 | LRRC58 |
| 200759_x_at | -2.212564268 | 9.055833051 | -23.7916681 | 4.30E-33 | 3.62E-31 | 65.08915858 | NFE2L1 |
| 227383_at | 2.212289662 | 6.298137721 | 8.77050209 | 1.68E-12 | 6.78E-12 | 17.39610621 | LOC727820 |
| 202451_at | 2.21185746 | 7.295034261 | 16.38081259 | 2.46E-24 | 5.09E-23 | 44.80011318 | GTF2H1 |
| 212665_at | 2.211787816 | 7.689752926 | 11.43267479 | 5.57E-17 | 3.94E-16 | 27.74903628 | TIPARP |
| 1554447_at | 2.210841134 | 5.987112529 | 10.41142552 | 2.68E-15 | 1.53E-14 | 23.85621698 | JPX |
| 226370_at | 2.210175505 | 6.262368204 | 13.39502472 | 4.65E-20 | 5.09E-19 | 34.88152561 | KLHL15 |
| 233642_s_at | 2.209759427 | 6.238969084 | 18.4123516 | 5.58E-27 | 1.77E-25 | 50.93013395 | HEATR5B |
| 212474_at | 2.209231276 | 6.340957538 | 14.40888869 | 1.45E-21 | 1.96E-20 | 38.37572514 | AVL9 |
| 229265_at | 2.208678456 | 7.479823658 | 17.80836299 | 3.25E-26 | 9.01E-25 | 49.15710573 | SKI |
| 214686_at | 2.207253053 | 7.559214287 | 16.58229723 | 1.32E-24 | 2.87E-23 | 45.42961867 | ZNF266 |
| 227796_at | 2.206734184 | 6.867443306 | 15.86825624 | 1.23E-23 | 2.28E-22 | 43.17657813 | ZFP62 |
| 200057_s_at | 2.206634365 | 10.50182902 | 24.02553703 | 2.47E-33 | 2.17E-31 | 65.64528808 | NONO |
| 208345_s_at | 2.206053555 | 6.794255986 | 8.160881107 | 1.93E-11 | 6.86E-11 | 14.95487541 | POU3F1 |
| 222450_at | 2.20604782 | 7.918379342 | 9.39861431 | 1.39E-13 | 6.42E-13 | 19.89307934 | PMEPA1 |
| 212909_at | 2.205606945 | 6.093116718 | 7.270113126 | 6.91E-10 | 2.04E-09 | 11.38169943 | LYPD1 |
| 201425_at | -2.205573739 | 7.993379505 | -8.294394431 | 1.13E-11 | 4.12E-11 | 15.49046532 | ALDH2 |
| 220703_at | -2.204994947 | 3.931777311 | -22.73814608 | 5.50E-32 | 3.97E-30 | 62.52740688 | IDI2-AS1 |
| 218381_s_at | 2.20456183 | 7.613153399 | 20.58469391 | 1.36E-29 | 6.90E-28 | 56.98392641 | U2AF2 |
| 225664_at | 2.203622879 | 8.131258691 | 4.503109306 | 2.98E-05 | 5.36E-05 | 0.849544215 | COL12A1 |
| 238723_at | 2.200183613 | 5.886314029 | 14.59199916 | 7.85E-22 | 1.11E-20 | 38.99281352 | ATXN3 |
| 204872_at | -2.200136116 | 5.176504412 | -10.94451955 | 3.50E-16 | 2.22E-15 | 25.90255255 | TLE4 |
| 228993_s_at | 2.19995761 | 7.050900607 | 13.22571646 | 8.41E-20 | 8.83E-19 | 34.28521178 | BBIP1 |
| 40225_at | 2.199716789 | 7.606787919 | 24.20876808 | 1.61E-33 | 1.47E-31 | 66.07790482 | GAK |
| 227624_at | 2.199231382 | 6.309406105 | 9.723340964 | 3.88E-14 | 1.92E-13 | 21.17336777 | TET2 |
| 207826_s_at | 2.198982099 | 7.924011846 | 7.480171995 | 2.97E-10 | 9.16E-10 | 12.22299126 | ID3 |
| 209317_at | 2.198615833 | 6.796052092 | 18.54979414 | 3.76E-27 | 1.23E-25 | 51.32795087 | POLR1C |
| 210427_x_at | 2.198023951 | 10.41188284 | 10.02726442 | 1.19E-14 | 6.24E-14 | 22.36373837 | ANXA2 |
| 204354_at | 2.197261809 | 6.446665566 | 17.57209548 | 6.54E-26 | 1.73E-24 | 48.45235266 | POT1 |
| 206703_at | -2.197197952 | 5.556011582 | -12.796842 | 3.84E-19 | 3.65E-18 | 32.7583499 | CHRNB1 |
| 244132_x_at | 2.194959693 | 6.280763303 | 14.98683757 | 2.12E-22 | 3.25E-21 | 40.3089686 | ZNF518A |
| 225051_at | 2.19495485 | 8.130654012 | 7.679046369 | 1.34E-10 | 4.30E-10 | 13.02070716 | EPB41 |
| 200652_at | 2.193108095 | 9.080485808 | 20.80610665 | 7.58E-30 | 4.01E-28 | 57.57399856 | SSR2 |
| 221269_s_at | 2.192609548 | 7.959368312 | 13.10555767 | 1.28E-19 | 1.31E-18 | 33.85978629 | SH3BGRL3 |
| 200606_at | 2.192540551 | 5.190065533 | 4.5801554 | 2.26E-05 | 4.13E-05 | 1.116702682 | DSP |
| 1552863_a_at | -2.191818103 | 6.382270679 | -25.84570558 | 3.83E-35 | 4.72E-33 | 69.82651589 | CACNG6 |
| 201847_at | 2.191749758 | 7.895563374 | 9.137590288 | 3.90E-13 | 1.71E-12 | 18.85835617 | LIPA |
| 217794_at | 2.191279662 | 8.17342025 | 23.3224823 | 1.32E-32 | 1.05E-30 | 63.95985996 | PRR13 |
| 213044_at | 2.190856255 | 7.997848222 | 17.41257065 | 1.05E-25 | 2.68E-24 | 47.9728998 | ROCK1 |
| 227864_s_at | 2.190703572 | 6.346658837 | 18.58083669 | 3.44E-27 | 1.13E-25 | 51.41751485 | MVB12A |
| 204160_s_at | -2.188405947 | 5.092369654 | -9.319587885 | 1.90E-13 | 8.64E-13 | 19.58030381 | ENPP4 |
| 229551_x_at | 2.187624385 | 4.971087041 | 12.87162988 | 2.94E-19 | 2.84E-18 | 33.02628823 | ZNF367 |
| 226616_s_at | -2.186531756 | 7.994883418 | -17.45239047 | 9.34E-26 | 2.41E-24 | 48.09285271 | NDUFV3 |
| 232028_at | 2.186531151 | 6.287154552 | 10.17784927 | 6.61E-15 | 3.59E-14 | 22.95044978 | ZNF678 |
| 200743_s_at | 2.185650316 | 8.548122622 | 14.25086743 | 2.47E-21 | 3.21E-20 | 37.83975745 | TPP1 |
| 209748_at | 2.185533637 | 6.276679268 | 12.37153299 | 1.77E-18 | 1.54E-17 | 31.2212152 | SPAST |
| 235626_at | 2.184959727 | 7.313942075 | 10.0097858 | 1.27E-14 | 6.66E-14 | 22.29550193 | CAMK1D |
| 208986_at | 2.183865479 | 8.245011322 | 12.72632958 | 4.93E-19 | 4.62E-18 | 32.50508096 | TCF12 |
| 202432_at | -2.180462798 | 9.270886887 | -19.14173459 | 7.02E-28 | 2.59E-26 | 53.01786316 | PPP3CB |
| 218924_s_at | 2.180143533 | 5.802243725 | 16.38860822 | 2.40E-24 | 4.99E-23 | 44.82456008 | CTBS |
| 203474_at | 2.17882009 | 5.517421163 | 9.192974272 | 3.13E-13 | 1.39E-12 | 19.07828447 | IQGAP2 |
| 222600_s_at | 2.178318745 | 7.169295397 | 14.29933825 | 2.09E-21 | 2.76E-20 | 38.00449647 | UBA6 |
| 213677_s_at | 2.178173697 | 6.980226329 | 14.87444298 | 3.07E-22 | 4.60E-21 | 39.93631832 | PMS1 |
| 208956_x_at | 2.177908558 | 9.633869544 | 18.02929903 | 1.70E-26 | 4.98E-25 | 49.81040285 | DUT |
| 228602_at | -2.177455441 | 6.320000645 | -8.077010547 | 2.70E-11 | 9.44E-11 | 14.61827521 | SGCD |
| 221830_at | 2.177178965 | 8.441986959 | 14.52082755 | 9.95E-22 | 1.39E-20 | 38.75346919 | RAP2A |
| 211752_s_at | -2.175814041 | 7.718466046 | -24.74285066 | 4.64E-34 | 4.81E-32 | 67.32364039 | NDUFS7 |
| 209340_at | 2.175604783 | 9.281384631 | 9.324055985 | 1.87E-13 | 8.50E-13 | 19.59799971 | UAP1 |
| 216849_at | -2.175541398 | 5.071007672 | -21.61778083 | 9.19E-31 | 5.62E-29 | 59.69691234 | AC007389.3 |
| 221563_at | -2.17461365 | 5.226757813 | -12.06733196 | 5.34E-18 | 4.37E-17 | 30.10793936 | DUSP10 |
| 223297_at | 2.173305108 | 6.59735168 | 16.21261965 | 4.16E-24 | 8.32E-23 | 44.27087734 | AMMECR1L |
| 228438_at | 2.173283189 | 5.276847915 | 9.129510538 | 4.03E-13 | 1.77E-12 | 18.82625541 | LOC100132891 |
| 201606_s_at | 2.173272356 | 8.930972869 | 22.72720276 | 5.65E-32 | 4.07E-30 | 62.50029959 | PWP1 |
| 235142_at | 2.17175337 | 6.108221463 | 10.03970312 | 1.13E-14 | 5.96E-14 | 22.41228199 | ZBTB8A |
| 219818_s_at | 2.171693504 | 6.196230986 | 20.39316797 | 2.27E-29 | 1.07E-27 | 56.46962375 | GPATCH1 |
| 225044_at | 2.171562642 | 7.871118546 | 23.28612275 | 1.44E-32 | 1.13E-30 | 63.87157676 | NT5C3B |
| 204069_at | 2.171268537 | 6.983511537 | 5.92329718 | 1.45E-07 | 3.33E-07 | 6.072251753 | MEIS1 |
| 215983_s_at | 2.171154986 | 6.721139532 | 14.58699942 | 7.98E-22 | 1.12E-20 | 38.97602079 | UBXN8 |
| 210694_s_at | 2.170100102 | 6.282792892 | 12.36938885 | 1.78E-18 | 1.55E-17 | 31.21340852 | MID1 |
| 204320_at | 2.169808315 | 6.771010687 | 4.052050126 | 0.000142392 | 0.000238643 | -0.66533091 | COL11A1 |
| 206277_at | -2.169343827 | 4.788010517 | -20.24632429 | 3.37E-29 | 1.53E-27 | 56.07284346 | P2RY2 |
| 231835_at | 2.16903951 | 6.081475088 | 15.11405922 | 1.40E-22 | 2.19E-21 | 40.72885977 | FAM213B |
| 218082_s_at | 2.168340203 | 7.415891558 | 19.34001294 | 4.03E-28 | 1.54E-26 | 53.57555792 | UBP1 |
| 202227_s_at | 2.16827405 | 8.091884482 | 18.52615184 | 4.02E-27 | 1.30E-25 | 51.25966748 | BRD8 |
| 202388_at | 2.167802666 | 6.632033269 | 5.898100041 | 1.60E-07 | 3.66E-07 | 5.97522691 | RGS2 |
| 205081_at | 2.167644408 | 7.761955098 | 6.290371723 | 3.44E-08 | 8.40E-08 | 7.497909328 | CRIP1 |
| 211202_s_at | 2.166501457 | 7.585388369 | 12.53420195 | 9.82E-19 | 8.87E-18 | 31.81180929 | KDM5B |
| 231714_s_at | 2.166225094 | 7.420085193 | 13.36883921 | 5.10E-20 | 5.55E-19 | 34.78953801 | AP4B1 |
| 229450_at | 2.165549695 | 6.791772651 | 6.136231878 | 6.31E-08 | 1.50E-07 | 6.896631686 | IFIT3 |
| 202771_at | 2.165200205 | 6.917071182 | 11.28674505 | 9.62E-17 | 6.57E-16 | 27.1998956 | PIEZO1 |
| 205263_at | 2.165052345 | 6.475139418 | 17.43900729 | 9.73E-26 | 2.50E-24 | 48.05255771 | BCL10 |
| 222445_at | 2.163696309 | 7.69488841 | 19.99231778 | 6.70E-29 | 2.92E-27 | 55.3814025 | SLC39A9 |
| 213233_s_at | 2.163027133 | 8.115807785 | 17.12279107 | 2.52E-25 | 6.11E-24 | 47.09443823 | KLHL9 |
| 239132_at | -2.163018329 | 6.584456492 | -3.970963593 | 0.000187091 | 0.000309697 | -0.927987709 | NOS1 |
| 221606_s_at | 2.162851939 | 5.776035478 | 7.839217105 | 7.02E-11 | 2.34E-10 | 13.66363951 | HMGN5 |
| 201744_s_at | 2.162642374 | 8.543588037 | 4.064245428 | 0.000136632 | 0.000229379 | -0.625558003 | LUM |
| 213478_at | 2.161740737 | 7.220993909 | 10.751061 | 7.30E-16 | 4.45E-15 | 25.16342536 | KAZN |
| 204825_at | 2.158290717 | 6.639866861 | 9.194843618 | 3.11E-13 | 1.38E-12 | 19.08570415 | MELK |
| 225308_s_at | 2.157967393 | 5.767235051 | 8.467980621 | 5.63E-12 | 2.14E-11 | 16.18616541 | TANC1 |
| 208795_s_at | 2.157757411 | 7.335432674 | 14.69120646 | 5.64E-22 | 8.12E-21 | 39.32536698 | MCM7 |
| 208374_s_at | 2.155772423 | 9.165088582 | 17.21546487 | 1.90E-25 | 4.68E-24 | 47.37643886 | CAPZA1 |
| 218445_at | 2.155408277 | 8.232715578 | 9.846447039 | 2.40E-14 | 1.22E-13 | 21.65650386 | H2AFY2 |
| 219110_at | 2.155342952 | 6.585328695 | 18.54058575 | 3.86E-27 | 1.26E-25 | 51.30136259 | GAR1 |
| 232099_at | 2.154824597 | 5.378028234 | 6.665653644 | 7.76E-09 | 2.04E-08 | 8.974947052 | PCDHB16 |
| 218132_s_at | 2.15449897 | 7.073715355 | 19.63250088 | 1.79E-28 | 7.26E-27 | 54.39071475 | TSEN34 |
| 235532_at | 2.153347503 | 4.90687865 | 15.82233789 | 1.43E-23 | 2.61E-22 | 43.02956506 | RP11-226L15.5 |
| 211535_s_at | 2.152895765 | 7.733603418 | 10.2613333 | 4.78E-15 | 2.65E-14 | 23.27479745 | FGFR1 |
| 214280_x_at | 2.152659561 | 9.327377164 | 14.14378249 | 3.54E-21 | 4.54E-20 | 37.47474243 | HNRNPA1 |
| 214093_s_at | 2.15215579 | 7.496456756 | 11.51368027 | 4.12E-17 | 2.97E-16 | 28.05279285 | FUBP1 |
| 202375_at | 2.152142779 | 4.716430621 | 9.432913768 | 1.21E-13 | 5.65E-13 | 20.02869183 | SEC24D |
| 218577_at | 2.151712457 | 7.465738064 | 13.76553131 | 1.29E-20 | 1.52E-19 | 36.17368714 | LRRC40 |
| 209561_at | 2.148870702 | 6.242531683 | 11.7316464 | 1.84E-17 | 1.39E-16 | 28.86627798 | THBS3 |
| 226587_at | 2.147149723 | 8.114932856 | 7.172179712 | 1.02E-09 | 2.96E-09 | 10.99012282 | PWAR6 |
| 200903_s_at | 2.146726306 | 8.676813217 | 11.91714091 | 9.26E-18 | 7.31E-17 | 29.55409164 | AHCY |
| 213311_s_at | 2.146691306 | 8.153879364 | 16.50323448 | 1.68E-24 | 3.59E-23 | 45.18318093 | TCF25 |
| 200614_at | 2.146679284 | 10.28544802 | 20.14085432 | 4.48E-29 | 2.01E-27 | 55.7865279 | CLTC |
| 218370_s_at | 2.145813997 | 6.333995136 | 18.23338832 | 9.37E-27 | 2.87E-25 | 50.40902061 | S100PBP |
| 212300_at | 2.145433889 | 5.959041989 | 17.07774787 | 2.89E-25 | 6.94E-24 | 46.95701222 | TXLNA |
| 228106_at | 2.144036951 | 6.006086547 | 14.2119712 | 2.81E-21 | 3.63E-20 | 37.70734311 | DCAF16 |
| 226335_at | -2.143954819 | 6.667838509 | -8.963026841 | 7.81E-13 | 3.29E-12 | 18.16393813 | RPS6KA3 |
| 217885_at | 2.143786525 | 8.319278583 | 17.17399103 | 2.16E-25 | 5.28E-24 | 47.25036036 | IPO9 |
| 208801_at | 2.142702078 | 8.450931876 | 15.29863964 | 7.67E-23 | 1.26E-21 | 41.33445129 | SRP72 |
| 204849_at | 2.141131762 | 6.550377433 | 11.65983302 | 2.39E-17 | 1.78E-16 | 28.59888299 | TCFL5 |
| 227442_at | 2.140293683 | 6.917103139 | 17.15214531 | 2.30E-25 | 5.62E-24 | 47.18386979 | COX18 |
| 202892_at | 2.13968471 | 6.11265606 | 16.13452998 | 5.31E-24 | 1.04E-22 | 44.02399551 | CDC23 |
| 241368_at | -2.139344809 | 6.303453213 | -16.85209425 | 5.74E-25 | 1.33E-23 | 46.2649607 | PLIN5 |
| 225837_at | 2.139109799 | 6.78904361 | 15.56223894 | 3.27E-23 | 5.71E-22 | 42.19192302 | RHNO1 |
| 226825_s_at | 2.138624418 | 7.423420754 | 12.17604282 | 3.59E-18 | 3.00E-17 | 30.5070989 | TMEM165 |
| 218696_at | 2.138116464 | 5.842893043 | 10.67894957 | 9.61E-16 | 5.77E-15 | 24.88688601 | EIF2AK3 |
| 225278_at | -2.137974491 | 7.690608408 | -11.56290093 | 3.43E-17 | 2.50E-16 | 28.23698499 | PRKAB2 |
| 221479_s_at | 2.137904536 | 8.947227053 | 16.2399007 | 3.82E-24 | 7.67E-23 | 44.35695241 | BNIP3L |
| 209421_at | 2.137882583 | 6.007294191 | 11.56114213 | 3.45E-17 | 2.52E-16 | 28.23040819 | MSH2 |
| 204803_s_at | -2.13744139 | 5.434755776 | -5.659686423 | 4.03E-07 | 8.82E-07 | 5.063316355 | RRAD |
| 225383_at | 2.137371976 | 7.210918838 | 9.866726831 | 2.22E-14 | 1.13E-13 | 21.73596851 | ZNF275 |
| 212228_s_at | -2.137354889 | 8.499177917 | -19.64520224 | 1.73E-28 | 7.04E-27 | 54.42591185 | COQ9 |
| 204740_at | -2.137291972 | 4.864669419 | -15.03671024 | 1.80E-22 | 2.78E-21 | 40.47381436 | CNKSR1 |
| 229710_at | 2.133999718 | 5.986007239 | 18.93759152 | 1.25E-27 | 4.45E-26 | 52.43931666 | ERCC6L2 |
| 223412_at | 2.133946471 | 5.798791133 | 12.4340879 | 1.41E-18 | 1.24E-17 | 31.44872163 | KBTBD7 |
| 233759_s_at | 2.133453286 | 7.73771972 | 17.27274604 | 1.60E-25 | 4.00E-24 | 47.55024162 | SMEK2 |
| 223904_at | -2.132110259 | 5.143380446 | -15.33401481 | 6.84E-23 | 1.13E-21 | 41.45002736 | PRKAG3 |
| 203166_at | 2.131957487 | 7.561537972 | 15.16957808 | 1.17E-22 | 1.87E-21 | 40.91146089 | CFDP1 |
| 229569_at | -2.12939671 | 5.838256945 | -10.47435569 | 2.10E-15 | 1.22E-14 | 24.09932389 | RP1-193H18.2 |
| 224964_s_at | 2.128661426 | 5.78101951 | 6.791220715 | 4.70E-09 | 1.27E-08 | 9.47258278 | GNG2 |
| 227933_at | 2.128435808 | 8.431546337 | 11.34104881 | 7.85E-17 | 5.43E-16 | 27.40453146 | LINGO1 |
| 208936_x_at | 2.127750884 | 6.88372106 | 16.79762691 | 6.78E-25 | 1.55E-23 | 46.09701619 | LGALS8 |
| 201296_s_at | 2.127470055 | 8.855500958 | 13.14978043 | 1.10E-19 | 1.13E-18 | 34.0165722 | WSB1 |
| 222978_at | 2.12735365 | 8.272443198 | 17.92295178 | 2.32E-26 | 6.64E-25 | 49.49662597 | SURF4 |
| 218373_at | -2.127304034 | 6.804062718 | -19.8776109 | 9.16E-29 | 3.91E-27 | 55.06701603 | AKTIP |
| 209040_s_at | 2.126288864 | 6.402294423 | 8.38519453 | 7.84E-12 | 2.92E-11 | 15.85448065 | PSMB8 |
| 205848_at | -2.125908039 | 4.225098143 | -10.99297174 | 2.91E-16 | 1.87E-15 | 26.08702784 | GAS2 |
| 202012_s_at | 2.125740064 | 6.934790721 | 14.7131336 | 5.24E-22 | 7.59E-21 | 39.39870072 | EXT2 |
| 219494_at | 2.125351395 | 6.160312977 | 11.24253894 | 1.14E-16 | 7.68E-16 | 27.03306081 | RAD54B |
| 234464_s_at | 2.123653454 | 6.071031266 | 12.93828068 | 2.32E-19 | 2.27E-18 | 33.26447693 | EME1 |
| 213326_at | 2.123148608 | 6.952315763 | 6.453009273 | 1.81E-08 | 4.55E-08 | 8.135933966 | VAMP1 |
| 235070_at | -2.122428771 | 6.26718851 | -14.59981257 | 7.64E-22 | 1.08E-20 | 39.01905018 | RBFOX1 |
| 1553145_at | 2.122427289 | 5.330457268 | 10.31066742 | 3.95E-15 | 2.22E-14 | 23.46615048 | TAPT1-AS1 |
| 201850_at | 2.122231264 | 6.332531511 | 9.273765629 | 2.28E-13 | 1.03E-12 | 19.39874511 | CAPG |
| 228796_at | 2.122123868 | 6.035491137 | 4.297684886 | 6.13E-05 | 0.000106638 | 0.148802962 | CPNE4 |
| 203062_s_at | 2.122081243 | 6.467122388 | 21.13858915 | 3.17E-30 | 1.78E-28 | 58.45116329 | MDC1 |
| 205968_at | -2.121437887 | 5.720644898 | -9.83415467 | 2.52E-14 | 1.27E-13 | 21.60831991 | KCNS3 |
| 202016_at | 2.120936996 | 6.553134801 | 5.344401609 | 1.35E-06 | 2.79E-06 | 3.876615975 | MEST |
| 203660_s_at | -2.120307402 | 7.681739808 | -16.135692 | 5.29E-24 | 1.04E-22 | 44.02767469 | PCNT |
| 202664_at | 2.120080744 | 5.662663468 | 7.474338693 | 3.04E-10 | 9.37E-10 | 12.19960781 | WIPF1 |
| 219374_s_at | 2.118359877 | 5.946716499 | 14.45432083 | 1.24E-21 | 1.71E-20 | 38.52923066 | ALG9 |
| 232174_at | 2.117753219 | 6.139580627 | 11.43317912 | 5.56E-17 | 3.94E-16 | 27.7509298 | AK025288 |
| 210155_at | -2.117474432 | 5.006777856 | -17.626932 | 5.56E-26 | 1.49E-24 | 48.6164897 | MYOC |
| 225505_s_at | 2.116816647 | 7.038296368 | 18.37633309 | 6.19E-27 | 1.95E-25 | 50.8255379 | PCED1A |
| 218319_at | 2.11679872 | 6.430877319 | 8.644376127 | 2.78E-12 | 1.09E-11 | 16.8921152 | PELI1 |
| 219762_s_at | 2.116624737 | 10.7112173 | 21.24873416 | 2.38E-30 | 1.38E-28 | 58.7394203 | RPL36 |
| 222047_s_at | 2.1164492 | 8.637665439 | 21.11413061 | 3.38E-30 | 1.89E-28 | 58.38699711 | SRRT |
| 234980_at | -2.116103076 | 4.359102095 | -19.24556179 | 5.25E-28 | 1.98E-26 | 53.31041371 | TMEM56 |
| 204957_at | 2.115991582 | 6.768190213 | 13.73231039 | 1.45E-20 | 1.68E-19 | 36.05854424 | ORC5 |
| 233461_x_at | 2.11536127 | 6.435997159 | 16.31625145 | 3.01E-24 | 6.16E-23 | 44.59736945 | ZNF226 |
| 226751_at | 2.115289931 | 6.640701386 | 9.579312416 | 6.83E-14 | 3.28E-13 | 20.60652813 | CNRIP1 |
| 212990_at | 2.114606451 | 5.900238841 | 13.06516179 | 1.48E-19 | 1.49E-18 | 33.71635043 | SYNJ1 |
| 226925_at | 2.114471262 | 5.8543377 | 11.55773451 | 3.50E-17 | 2.55E-16 | 28.21766482 | PXYLP1 |
| 203570_at | 2.113374874 | 5.956839173 | 5.813087511 | 2.23E-07 | 5.01E-07 | 5.648758906 | LOXL1 |
| 204064_at | 2.113078849 | 7.941440842 | 13.17379088 | 1.01E-19 | 1.05E-18 | 34.10159367 | THOC1 |
| 213125_at | 2.112423165 | 6.578798706 | 5.670923528 | 3.86E-07 | 8.46E-07 | 5.106034447 | OLFML2B |
| 203320_at | 2.111808735 | 7.122851578 | 7.620777432 | 1.69E-10 | 5.36E-10 | 12.7868944 | SH2B3 |
| 212709_at | 2.110920701 | 5.622386111 | 12.23500393 | 2.90E-18 | 2.45E-17 | 30.72298001 | NUP160 |
| 205478_at | -2.110403721 | 9.438745485 | -6.332320035 | 2.91E-08 | 7.18E-08 | 7.662136638 | PPP1R1A |
| 43544_at | 2.110385531 | 6.981308071 | 18.69658365 | 2.47E-27 | 8.28E-26 | 51.75054229 | MED16 |
| 218757_s_at | 2.108709718 | 7.033166878 | 14.45316151 | 1.25E-21 | 1.71E-20 | 38.52531683 | UPF3B |
| 201036_s_at | -2.10831469 | 8.515212345 | -14.72692615 | 5.01E-22 | 7.26E-21 | 39.44479773 | HADH |
| 228992_at | 2.108298964 | 7.470166579 | 13.84678976 | 9.75E-21 | 1.17E-19 | 36.45473186 | MED28 |
| 219506_at | 2.107276573 | 8.182823651 | 12.18765098 | 3.44E-18 | 2.89E-17 | 30.54963515 | C1orf54 |
| 218869_at | -2.107234118 | 6.134957761 | -18.74019536 | 2.18E-27 | 7.41E-26 | 51.87564458 | MLYCD |
| 202307_s_at | 2.107112564 | 6.978019103 | 10.77818402 | 6.58E-16 | 4.04E-15 | 25.26729558 | TAP1 |
| 1564807_at | -2.107104562 | 3.464399505 | -12.7586696 | 4.39E-19 | 4.16E-18 | 32.62131898 | RP11-504A18.1 |
| 209771_x_at | 2.106700034 | 6.141576658 | 4.239718409 | 7.49E-05 | 0.000129184 | -0.045738881 | CD24 |
| 236291_at | -2.106696301 | 4.996174072 | -20.96949906 | 4.93E-30 | 2.69E-28 | 58.00639259 | RDH5 |
| 200660_at | 2.1063897 | 7.432499003 | 7.076457775 | 1.50E-09 | 4.27E-09 | 10.60788977 | S100A11 |
| 204412_s_at | 2.106349058 | 6.692896405 | 8.147325324 | 2.03E-11 | 7.21E-11 | 14.9004782 | NEFH |
| 200791_s_at | 2.106108979 | 8.399574989 | 14.04966029 | 4.88E-21 | 6.16E-20 | 37.15270368 | IQGAP1 |
| 221739_at | 2.105720413 | 7.582883929 | 24.10682794 | 2.04E-33 | 1.82E-31 | 65.83755354 | C19orf10 |
| 225857_s_at | 2.105584608 | 7.101840014 | 11.18652575 | 1.40E-16 | 9.35E-16 | 26.82134474 | SNHG17 |
| 202484_s_at | 2.105362244 | 7.04121595 | 14.38425156 | 1.57E-21 | 2.12E-20 | 38.29237164 | MBD2 |
| 201433_s_at | 2.103743619 | 8.803323791 | 12.59744111 | 7.83E-19 | 7.14E-18 | 32.04051367 | PTDSS1 |
| 226334_s_at | 2.103393617 | 7.44521124 | 8.692634071 | 2.29E-12 | 9.12E-12 | 17.08503393 | AHSA2 |
| 203083_at | 2.103022984 | 7.403378427 | 4.942026704 | 6.07E-06 | 1.18E-05 | 2.400331665 | THBS2 |
| 226513_at | 2.102776348 | 5.369117008 | 17.41519142 | 1.04E-25 | 2.66E-24 | 47.9808002 | ASB7 |
| 217716_s_at | 2.102132769 | 8.049882813 | 21.65487729 | 8.36E-31 | 5.18E-29 | 59.79245637 | SEC61A1 |
| 208905_at | -2.102082687 | 10.22285449 | -14.07737936 | 4.44E-21 | 5.62E-20 | 37.24766194 | CYCS |
| 205474_at | 2.10185603 | 6.93145213 | 18.03101638 | 1.69E-26 | 4.97E-25 | 49.81545945 | CRLF3 |
| 65517_at | -2.101805513 | 5.270879012 | -17.55565771 | 6.87E-26 | 1.80E-24 | 48.40308393 | AP1M2 |
| 236814_at | 2.100070772 | 8.799220814 | 18.27981218 | 8.19E-27 | 2.54E-25 | 50.54453999 | MDM4 |
| 222843_at | 2.09961456 | 6.325143326 | 14.11709623 | 3.88E-21 | 4.94E-20 | 37.38355046 | FIGNL1 |
| 228974_at | 2.099592146 | 5.988545759 | 12.17980026 | 3.54E-18 | 2.97E-17 | 30.52086926 | ZNF677 |
| 208638_at | 2.099367352 | 8.768311877 | 11.0920624 | 2.00E-16 | 1.31E-15 | 26.46348977 | PDIA6 |
| 219972_s_at | 2.099049203 | 6.125794993 | 12.50357391 | 1.10E-18 | 9.83E-18 | 31.70086259 | PCNXL4 |
| 209044_x_at | 2.098865456 | 7.194390522 | 22.56127673 | 8.52E-32 | 5.97E-30 | 62.08800838 | SF3B4 |
| 204784_s_at | -2.097873509 | 6.690221122 | -9.967894144 | 1.49E-14 | 7.76E-14 | 22.13184427 | MLF1 |
| 209822_s_at | -2.097306874 | 4.657584125 | -9.207712774 | 2.96E-13 | 1.31E-12 | 19.13677731 | VLDLR |
| 200735_x_at | 2.096942232 | 11.99499876 | 20.2419211 | 3.41E-29 | 1.54E-27 | 56.06091257 | NACA |
| 218559_s_at | 2.096864115 | 8.856256074 | 7.483970896 | 2.93E-10 | 9.03E-10 | 12.23822014 | MAFB |
| 203067_at | -2.09676299 | 7.472802799 | -19.94634364 | 7.60E-29 | 3.28E-27 | 55.25555805 | PDHX |
| 200613_at | 2.095882904 | 8.56037302 | 20.49805157 | 1.72E-29 | 8.40E-28 | 56.75171493 | AP2M1 |
| 226408_at | 2.095428169 | 6.922209629 | 15.58537829 | 3.04E-23 | 5.32E-22 | 42.26678158 | TEAD2 |
| 244050_at | 2.095162719 | 5.529058752 | 8.491212334 | 5.13E-12 | 1.96E-11 | 16.2792049 | PTPLAD2 |
| 218846_at | 2.094546047 | 6.903337492 | 17.14186109 | 2.38E-25 | 5.79E-24 | 47.152549 | MED23 |
| 202069_s_at | -2.094450457 | 6.422791124 | -10.60553108 | 1.27E-15 | 7.53E-15 | 24.60476983 | IDH3A |
| 1558097_at | 2.094249288 | 7.132523916 | 22.25693859 | 1.82E-31 | 1.22E-29 | 61.32548225 | PRR14L |
| 213016_at | 2.093574992 | 7.437577371 | 11.01920424 | 2.64E-16 | 1.70E-15 | 26.18679608 | BBX |
| 219939_s_at | -2.093174159 | 10.08552123 | -18.25155547 | 8.89E-27 | 2.74E-25 | 50.46208216 | CSDE1 |
| 201876_at | 2.091943739 | 8.043984694 | 11.40812411 | 6.11E-17 | 4.30E-16 | 27.65682388 | PON2 |
| 227052_at | 2.091541895 | 7.015051369 | 12.78567747 | 3.99E-19 | 3.79E-18 | 32.71829063 | SMIM14 |
| 208864_s_at | 2.091416496 | 8.696061648 | 12.90053427 | 2.65E-19 | 2.58E-18 | 33.12965248 | TXN |
| 225180_at | 2.090622598 | 7.124236702 | 10.66668873 | 1.01E-15 | 6.03E-15 | 24.83981222 | TTC14 |
| 232449_at | -2.090456996 | 4.935972078 | -18.16103273 | 1.16E-26 | 3.51E-25 | 50.19732468 | BCO2 |
| 214145_s_at | -2.090383639 | 5.855500917 | -13.75260069 | 1.35E-20 | 1.58E-19 | 36.1288867 | SPTB |
| 230528_s_at | 2.08984239 | 6.899796569 | 13.39761504 | 4.61E-20 | 5.04E-19 | 34.89062043 | CENPBD1P1 |
| 219174_at | 2.089125443 | 5.566216025 | 13.60643288 | 2.23E-20 | 2.54E-19 | 35.62097437 | IFT74 |
| 209244_s_at | -2.088691553 | 6.788732006 | -17.18542766 | 2.08E-25 | 5.12E-24 | 47.28514718 | KIF1C |
| 224876_at | 2.087870813 | 8.840586619 | 15.91164765 | 1.07E-23 | 2.01E-22 | 43.31526299 | C5orf24 |
| 238021_s_at | 2.087858852 | 6.59182027 | 6.850805569 | 3.71E-09 | 1.01E-08 | 9.709222437 | CRNDE |
| 222673_x_at | 2.087651994 | 6.313148403 | 9.139252975 | 3.88E-13 | 1.70E-12 | 18.8649615 | FAM122B |
| 221676_s_at | 2.087257653 | 7.11945494 | 14.32907077 | 1.89E-21 | 2.52E-20 | 38.10540091 | CORO1C |
| 211959_at | -2.087025094 | 9.419801548 | -6.788218774 | 4.76E-09 | 1.28E-08 | 9.460668774 | IGFBP5 |
| 226545_at | 2.085938045 | 6.189482405 | 6.919377786 | 2.82E-09 | 7.77E-09 | 9.981917007 | CD109 |
| 200068_s_at | 2.084884451 | 10.03942775 | 24.16850535 | 1.76E-33 | 1.60E-31 | 65.98307441 | CANX |
| 203564_at | 2.084673145 | 6.508696806 | 15.47000245 | 4.41E-23 | 7.53E-22 | 41.89286679 | FANCG |
| 209406_at | -2.084191868 | 5.315183187 | -9.696284393 | 4.31E-14 | 2.13E-13 | 21.06701236 | BAG2 |
| 241804_at | 2.083969499 | 5.298881511 | 6.617548025 | 9.40E-09 | 2.45E-08 | 8.784708419 | LOC102724275 |
| 225478_at | 2.083655574 | 6.873245512 | 13.85047992 | 9.63E-21 | 1.16E-19 | 36.46747479 | MFHAS1 |
| 203140_at | -2.083277565 | 8.7556281 | -7.679948965 | 1.33E-10 | 4.29E-10 | 13.0243294 | BCL6 |
| 204559_s_at | 2.078972961 | 8.331385927 | 19.54044083 | 2.31E-28 | 9.14E-27 | 54.13510552 | LSM7 |
| 209476_at | 2.078649344 | 7.661783313 | 13.65428151 | 1.89E-20 | 2.17E-19 | 35.78754256 | TMX1 |
| 224721_at | 2.078316099 | 6.375244482 | 16.97028182 | 4.00E-25 | 9.43E-24 | 46.6281746 | WDR75 |
| 209513_s_at | -2.077753867 | 7.892953577 | -13.32426883 | 5.96E-20 | 6.41E-19 | 34.63276439 | HSDL2 |
| 223266_at | -2.076486926 | 7.743030632 | -14.75402849 | 4.58E-22 | 6.67E-21 | 39.53530811 | STRADB |
| 218932_at | 2.076137652 | 6.492177549 | 14.41192449 | 1.43E-21 | 1.94E-20 | 38.38599062 | ZNHIT6 |
| 206287_s_at | -2.076050396 | 5.858496164 | -12.92491754 | 2.43E-19 | 2.38E-18 | 33.21676649 | ITIH4 |
| 217574_at | 2.075864857 | 5.85957438 | 5.606513467 | 4.95E-07 | 1.07E-06 | 4.861552594 | CDH8 |
| 209141_at | -2.074984662 | 8.526672601 | -15.12556871 | 1.35E-22 | 2.12E-21 | 40.76674617 | UBE2G1 |
| 212690_at | 2.074870554 | 7.445622866 | 11.88549208 | 1.04E-17 | 8.16E-17 | 29.43703217 | DDHD2 |
| 205259_at | -2.074259346 | 5.145490324 | -7.647902644 | 1.51E-10 | 4.84E-10 | 12.89573106 | NR3C2 |
| 219489_s_at | -2.073598242 | 8.013660904 | -6.92649541 | 2.74E-09 | 7.57E-09 | 10.01024308 | NXN |
| 209803_s_at | 2.07304096 | 4.713406272 | 7.531883617 | 2.41E-10 | 7.53E-10 | 12.43032553 | PHLDA2 |
| 200833_s_at | 2.071944703 | 9.541006193 | 17.10004814 | 2.70E-25 | 6.50E-24 | 47.02507964 | RAP1B |
| 201903_at | -2.071669934 | 9.520604861 | -20.47477824 | 1.83E-29 | 8.85E-28 | 56.6892137 | UQCRC1 |
| 200040_at | 2.070089913 | 9.264527258 | 24.36875438 | 1.10E-33 | 1.05E-31 | 66.45343755 | KHDRBS1 |
| 202239_at | 2.070070456 | 7.733890281 | 13.76181874 | 1.31E-20 | 1.53E-19 | 36.1608265 | PARP4 |
| 201770_at | 2.069440751 | 8.003190022 | 16.57341349 | 1.35E-24 | 2.95E-23 | 45.40196546 | SNRPA |
| 236635_at | 2.067760847 | 6.147894073 | 10.6972999 | 8.96E-16 | 5.39E-15 | 24.9573099 | ZNF667 |
| 208079_s_at | 2.067450866 | 5.903807396 | 7.169968459 | 1.03E-09 | 2.99E-09 | 10.98128705 | AURKA |
| 243610_at | -2.067442159 | 3.700483191 | -11.73100196 | 1.84E-17 | 1.40E-16 | 28.86388116 | C9orf135 |
| 213283_s_at | 2.06611221 | 7.029027778 | 9.845157751 | 2.41E-14 | 1.22E-13 | 21.65145068 | SALL2 |
| 202953_at | 2.066057117 | 6.094015036 | 5.739407769 | 2.97E-07 | 6.58E-07 | 5.366959542 | C1QB |
| 223096_at | 2.065201357 | 9.020457243 | 15.0277734 | 1.86E-22 | 2.85E-21 | 40.44429814 | NOP58 |
| 222805_at | 2.064820408 | 4.565645471 | 13.34030641 | 5.63E-20 | 6.08E-19 | 34.68920484 | MANEA |
| 206254_at | -2.064683781 | 5.41457352 | -5.994475231 | 1.10E-07 | 2.55E-07 | 6.346953364 | EGF |
| 227952_at | 2.06364697 | 5.330748439 | 5.581250847 | 5.45E-07 | 1.18E-06 | 4.765914604 | ZNF595 |
| 202738_s_at | -2.06238725 | 6.723971223 | -13.55243904 | 2.69E-20 | 3.03E-19 | 35.43266202 | PHKB |
| 229139_at | -2.062121039 | 9.440360064 | -5.376250064 | 1.19E-06 | 2.48E-06 | 3.995389405 | JPH1 |
| 205339_at | 2.061906911 | 5.775086003 | 12.99416662 | 1.90E-19 | 1.89E-18 | 33.46376086 | STIL |
| 219469_at | 2.060101356 | 5.809577713 | 12.8642111 | 3.02E-19 | 2.91E-18 | 32.999741 | DYNC2H1 |
| 217815_at | 2.059523403 | 7.970561906 | 14.91669184 | 2.67E-22 | 4.04E-21 | 40.07658352 | SUPT16H |
| 226197_at | -2.059043809 | 5.659290314 | -12.28171447 | 2.45E-18 | 2.09E-17 | 30.89370116 | AR |
| 206829_x_at | 2.057986707 | 6.804732841 | 16.44535416 | 2.01E-24 | 4.25E-23 | 45.00229342 | ZNF430 |
| 204805_s_at | 2.05713578 | 7.793189728 | 14.60827583 | 7.43E-22 | 1.05E-20 | 39.04746027 | H1FX |
| 229943_at | 2.056993475 | 7.002687266 | 11.68583699 | 2.17E-17 | 1.63E-16 | 28.69577921 | TRIM13 |
| 227657_at | -2.056783562 | 5.497264151 | -10.7097086 | 8.55E-16 | 5.16E-15 | 25.00491108 | RNF150 |
| 203545_at | 2.055858055 | 6.745705911 | 16.42380064 | 2.15E-24 | 4.52E-23 | 44.93483151 | ALG8 |
| 237035_at | 2.054542888 | 5.185925848 | 6.740420295 | 5.76E-09 | 1.54E-08 | 9.271076931 | RP11-319G9.3 |
| 209337_at | 2.054064765 | 8.586872647 | 12.23977785 | 2.85E-18 | 2.42E-17 | 30.74044046 | PSIP1 |
| 221230_s_at | 2.053987457 | 7.43841813 | 16.37520111 | 2.50E-24 | 5.17E-23 | 44.7825112 | ARID4B |
| 226178_at | 2.052915759 | 6.046156724 | 12.36917128 | 1.78E-18 | 1.55E-17 | 31.21261632 | SOCS4 |
| 218809_at | 2.052471308 | 7.133236577 | 19.56724016 | 2.15E-28 | 8.55E-27 | 54.20960578 | PANK2 |
| 204122_at | 2.052228826 | 6.93203219 | 6.601820253 | 1.00E-08 | 2.60E-08 | 8.722563623 | TYROBP |
| 222754_at | 2.051811175 | 6.303694181 | 15.06217714 | 1.66E-22 | 2.57E-21 | 40.55787025 | TRNT1 |
| 201673_s_at | -2.051084744 | 7.680770817 | -11.89122804 | 1.02E-17 | 8.01E-17 | 29.45825678 | GYS1 |
| 212616_at | 2.050974754 | 8.545229306 | 12.81118831 | 3.64E-19 | 3.48E-18 | 32.80980248 | CHD9 |
| 226015_at | 2.050544841 | 7.317361251 | 15.20581405 | 1.04E-22 | 1.67E-21 | 41.03043233 | ZNF12 |
| 204824_at | -2.050221781 | 6.969368311 | -13.43102045 | 4.11E-20 | 4.51E-19 | 35.00783266 | ENDOG |
| 232936_at | -2.050086756 | 4.381968388 | -22.7832619 | 4.92E-32 | 3.63E-30 | 62.63905149 | KCNA7 |
| 205964_at | 2.049379602 | 5.778652197 | 15.73123228 | 1.91E-23 | 3.44E-22 | 42.73711185 | ZNF426 |
| 226000_at | 2.048870843 | 6.395031122 | 14.74875816 | 4.66E-22 | 6.78E-21 | 39.51771469 | CTTNBP2NL |
| 229033_s_at | 2.047576142 | 6.75459213 | 14.40669731 | 1.46E-21 | 1.98E-20 | 38.36831428 | MUM1 |
| 210027_s_at | 2.047371183 | 9.930901937 | 15.31617039 | 7.25E-23 | 1.20E-21 | 41.39174644 | APEX1 |
| 208642_s_at | 2.046419448 | 9.369112861 | 16.16873284 | 4.77E-24 | 9.42E-23 | 44.13221959 | XRCC5 |
| 224817_at | 2.046210464 | 8.300872008 | 11.11762411 | 1.82E-16 | 1.19E-15 | 26.56042453 | SH3PXD2A |
| 203721_s_at | 2.04610144 | 7.880956337 | 14.35303996 | 1.75E-21 | 2.34E-20 | 38.18666409 | UTP18 |
| 204285_s_at | 2.045010649 | 4.826137042 | 5.681915017 | 3.70E-07 | 8.12E-07 | 5.147845127 | PMAIP1 |
| 212739_s_at | 2.044988037 | 8.471497164 | 12.1351884 | 4.17E-18 | 3.46E-17 | 30.35726216 | NME4 |
| 200713_s_at | 2.044666256 | 8.803998917 | 19.15423383 | 6.78E-28 | 2.50E-26 | 53.05314226 | MAPRE1 |
| 226038_at | 2.044338347 | 8.574511929 | 6.900768849 | 3.04E-09 | 8.34E-09 | 9.907877215 | LONRF1 |
| 201387_s_at | 2.043621104 | 7.594184183 | 5.518002434 | 6.95E-07 | 1.48E-06 | 4.527112736 | UCHL1 |
| 202778_s_at | 2.041856201 | 7.070561013 | 15.35122681 | 6.47E-23 | 1.08E-21 | 41.50620508 | ZMYM2 |
| 202184_s_at | 2.041779716 | 7.273721489 | 15.7204278 | 1.97E-23 | 3.55E-22 | 42.70236115 | NUP133 |
| 209549_s_at | 2.041440958 | 7.535363226 | 22.18569626 | 2.17E-31 | 1.44E-29 | 61.14579129 | DGUOK |
| 225806_at | 2.041307207 | 4.791716323 | 8.443084041 | 6.22E-12 | 2.35E-11 | 16.08643879 | AJUBA |
| 208766_s_at | 2.041092249 | 9.505403837 | 19.48125018 | 2.72E-28 | 1.06E-26 | 53.97029518 | HNRNPR |
| 204510_at | 2.04046989 | 5.180146827 | 8.65395308 | 2.67E-12 | 1.06E-11 | 16.93040843 | CDC7 |
| 220770_s_at | 2.040466769 | 5.097076316 | 10.57444473 | 1.43E-15 | 8.43E-15 | 24.4851483 | ZBED8 |
| 226188_at | -2.040323137 | 7.627742575 | -10.90845701 | 4.01E-16 | 2.53E-15 | 25.76508125 | LGALSL |
| 226255_at | 2.039935998 | 7.770176518 | 16.51794205 | 1.61E-24 | 3.46E-23 | 45.22908098 | ZBTB33 |
| 223061_at | 2.039478366 | 7.246468664 | 15.58736181 | 3.02E-23 | 5.30E-22 | 42.2731954 | CHID1 |
| 218882_s_at | 2.039306753 | 7.09856392 | 15.29533253 | 7.75E-23 | 1.27E-21 | 41.32363848 | WDR3 |
| 201603_at | 2.038151534 | 8.064715721 | 13.52910238 | 2.92E-20 | 3.27E-19 | 35.35115617 | PPP1R12A |
| 236656_s_at | -2.037182016 | 4.792713936 | -10.72273983 | 8.13E-16 | 4.93E-15 | 25.05488276 | LOC100288911 |
| 201773_at | 2.037172828 | 8.069783062 | 14.70889823 | 5.32E-22 | 7.69E-21 | 39.38454059 | ADNP |
| 219572_at | 2.036960743 | 7.062466164 | 6.512048373 | 1.43E-08 | 3.65E-08 | 8.368367934 | CADPS2 |
| 218232_at | 2.036536306 | 6.836514763 | 5.783043441 | 2.50E-07 | 5.59E-07 | 5.533718869 | C1QA |
| 216515_x_at | 2.035879371 | 9.924736058 | 22.85788066 | 4.10E-32 | 3.06E-30 | 62.8233169 | MIR1244-3 |
| 213039_at | 2.03563921 | 6.506822448 | 18.65145998 | 2.81E-27 | 9.32E-26 | 51.62088568 | ARHGEF18 |
| 226093_at | 2.035102894 | 6.417699 | 14.03154273 | 5.19E-21 | 6.53E-20 | 37.0905846 | DCP1B |
| 224598_at | 2.033259013 | 7.183651018 | 14.35806988 | 1.72E-21 | 2.30E-20 | 38.20370783 | MGAT4B |
| 219087_at | 2.032809929 | 7.692204814 | 3.736095564 | 0.000406011 | 0.000648056 | -1.670450465 | ASPN |
| 201046_s_at | -2.032724846 | 9.454394049 | -19.3537104 | 3.88E-28 | 1.49E-26 | 53.61393174 | RAD23A |
| 213065_at | 2.032706075 | 6.949640672 | 13.84545553 | 9.80E-21 | 1.18E-19 | 36.45012404 | ZFC3H1 |
| 208546_x_at | 2.032437149 | 5.756258772 | 9.05473749 | 5.42E-13 | 2.34E-12 | 18.52899164 | HIST1H2BH |
| 228387_at | 2.030726634 | 6.211185161 | 9.414781745 | 1.30E-13 | 6.04E-13 | 19.95701253 | LINC01355 |
| 202590_s_at | -2.030306262 | 6.97148657 | -18.9814805 | 1.10E-27 | 3.97E-26 | 52.56407437 | PDK2 |
| 221493_at | -2.029284734 | 8.596792681 | -12.50160597 | 1.10E-18 | 9.88E-18 | 31.69372995 | TSPYL1 |
| 202246_s_at | 2.029203004 | 8.510838709 | 16.36787303 | 2.56E-24 | 5.28E-23 | 44.7595189 | CDK4 |
| 203039_s_at | -2.029048779 | 8.516071084 | -18.36906102 | 6.33E-27 | 1.99E-25 | 50.80440283 | NDUFS1 |
| 227801_at | 2.028791383 | 4.866214283 | 10.90217526 | 4.11E-16 | 2.59E-15 | 25.74112046 | TRIM59 |
| 210054_at | 2.028543459 | 6.220832159 | 15.25192287 | 8.93E-23 | 1.45E-21 | 41.18158096 | HAUS3 |
| 214431_at | 2.028503825 | 7.985215408 | 17.00107 | 3.64E-25 | 8.65E-24 | 46.72252263 | GMPS |
| 202178_at | 2.028060863 | 5.886784201 | 8.429217823 | 6.57E-12 | 2.48E-11 | 16.03088729 | PRKCZ |
| 201690_s_at | 2.027210326 | 7.354165165 | 6.890625459 | 3.16E-09 | 8.66E-09 | 9.867530813 | TPD52 |
| 225609_at | 2.026388955 | 7.164030065 | 10.94045519 | 3.55E-16 | 2.26E-15 | 25.88706626 | GSR |
| 218721_s_at | 2.025273394 | 7.127835514 | 14.6607358 | 6.24E-22 | 8.92E-21 | 39.22335887 | C1orf27 |
| 228334_x_at | 2.024770396 | 6.423752991 | 10.49597605 | 1.94E-15 | 1.13E-14 | 24.18275328 | CEP44 |
| 228774_at | 2.024565194 | 6.39339743 | 12.04323024 | 5.83E-18 | 4.75E-17 | 30.0192471 | CEP78 |
| 202483_s_at | 2.024555463 | 8.343461652 | 13.44921133 | 3.85E-20 | 4.25E-19 | 35.07160048 | RANBP1 |
| 202236_s_at | -2.024380522 | 7.143598541 | -8.371677766 | 8.27E-12 | 3.08E-11 | 15.80030611 | SLC16A1 |
| 202365_at | 2.023954988 | 7.494561491 | 17.06484825 | 3.00E-25 | 7.20E-24 | 46.91761197 | UNC119B |
| 221139_s_at | 2.023814445 | 7.20137669 | 8.830873745 | 1.32E-12 | 5.41E-12 | 17.63708612 | CSAD |
| 225010_at | 2.023534696 | 7.665692332 | 11.77065157 | 1.59E-17 | 1.22E-16 | 29.01125366 | CCDC6 |
| 227084_at | -2.022673373 | 6.274744913 | -6.625724422 | 9.10E-09 | 2.37E-08 | 8.817025947 | DTNA |
| 212451_at | 2.021975235 | 7.545622467 | 10.06195144 | 1.04E-14 | 5.49E-14 | 22.49907347 | SECISBP2L |
| 213302_at | 2.021887254 | 6.236510193 | 13.82581577 | 1.05E-20 | 1.25E-19 | 36.38227102 | PFAS |
| 209344_at | 2.020070329 | 6.993932308 | 9.638868161 | 5.40E-14 | 2.63E-13 | 20.84111903 | TPM4 |
| 213414_s_at | 2.019890989 | 12.60973407 | 20.6692105 | 1.09E-29 | 5.67E-28 | 57.20973061 | RPS19 |
| 202641_at | 2.01983555 | 7.201323815 | 18.19405646 | 1.05E-26 | 3.21E-25 | 50.2940168 | ARL3 |
| 200837_at | 2.019821488 | 8.001150248 | 15.88941722 | 1.15E-23 | 2.14E-22 | 43.24424035 | BCAP31 |
| 220046_s_at | 2.01959752 | 8.283660633 | 12.05637259 | 5.56E-18 | 4.54E-17 | 30.06761867 | CCNL1 |
| 209431_s_at | 2.019315228 | 6.647449403 | 16.83044525 | 6.13E-25 | 1.41E-23 | 46.19825026 | PATZ1 |
| 221011_s_at | 2.019164706 | 6.626225304 | 7.110688557 | 1.31E-09 | 3.74E-09 | 10.74451714 | LBH |
| 200009_at | 2.018594882 | 10.25958121 | 18.95883018 | 1.17E-27 | 4.22E-26 | 52.49971488 | GDI2 |
| 228318_s_at | 2.018277005 | 6.842545193 | 11.76646269 | 1.61E-17 | 1.23E-16 | 28.99569303 | CRIPAK |
| 235849_at | -2.017715869 | 5.627496 | -14.04485315 | 4.96E-21 | 6.26E-20 | 37.13622567 | SCARA5 |
| 213413_at | 2.016450514 | 6.176505442 | 6.79863787 | 4.57E-09 | 1.23E-08 | 9.502023149 | STON1 |
| 212897_at | 2.015489876 | 7.234098467 | 17.69831518 | 4.50E-26 | 1.23E-24 | 48.82963966 | CDK19 |
| 224943_at | 2.015442363 | 7.560818282 | 12.83824068 | 3.31E-19 | 3.17E-18 | 32.90675413 | BTBD7 |
| 201579_at | 2.015268844 | 6.34685767 | 6.699806764 | 6.77E-09 | 1.79E-08 | 9.110150856 | FAT1 |
| 205206_at | -2.014761643 | 6.165879762 | -5.337005599 | 1.38E-06 | 2.86E-06 | 3.849071393 | KAL1 |
| 202423_at | 2.013914286 | 8.603689746 | 16.27959252 | 3.37E-24 | 6.85E-23 | 44.48202401 | KAT6A |
| 220419_s_at | -2.013895442 | 7.496402198 | -8.389300764 | 7.71E-12 | 2.88E-11 | 15.87093722 | USP25 |
| 213145_at | 2.012792038 | 6.472975755 | 14.43264394 | 1.34E-21 | 1.83E-20 | 38.45602182 | FBXL14 |
| 221059_s_at | 2.012535541 | 6.348129436 | 8.17529265 | 1.82E-11 | 6.49E-11 | 15.01270345 | COTL1 |
| 1558592_at | 2.012237619 | 4.830914888 | 8.818491271 | 1.39E-12 | 5.66E-12 | 17.58767474 | AX747507 |
| 205593_s_at | 2.011304082 | 6.585091926 | 10.29000359 | 4.28E-15 | 2.39E-14 | 23.38603031 | PDE9A |
| 204008_at | 2.010532377 | 6.83473255 | 8.120097555 | 2.27E-11 | 8.00E-11 | 14.79120931 | DNAL4 |
| 227087_at | 2.010464487 | 6.996154937 | 15.16527839 | 1.18E-22 | 1.89E-21 | 40.89733305 | INPP4A |
| 1555616_at | -2.008990944 | 5.034523641 | -14.31208917 | 2.01E-21 | 2.66E-20 | 38.04778357 | AC010524.4 |
| 209407_s_at | 2.00787601 | 6.547976286 | 10.78693805 | 6.37E-16 | 3.91E-15 | 25.30080315 | DEAF1 |
| 225603_s_at | 2.007446768 | 6.193788699 | 12.65629209 | 6.34E-19 | 5.86E-18 | 32.25289657 | TRIQK |
| 201844_s_at | 2.00720741 | 7.755243443 | 14.38044133 | 1.59E-21 | 2.15E-20 | 38.27947379 | RYBP |
| 200728_at | 2.006411336 | 9.134451263 | 18.68135335 | 2.58E-27 | 8.63E-26 | 51.70680485 | ACTR2 |
| 221571_at | 2.006020016 | 6.251299194 | 11.6214049 | 2.76E-17 | 2.04E-16 | 28.45554431 | TRAF3 |
| 201947_s_at | 2.005763371 | 10.20633728 | 14.50135807 | 1.06E-21 | 1.47E-20 | 38.68788281 | CCT2 |
| 230577_at | -2.005217854 | 3.612408896 | -9.01857977 | 6.26E-13 | 2.68E-12 | 18.38512427 | LINC00844 |
| 218061_at | 2.00360645 | 8.49811139 | 22.75427822 | 5.29E-32 | 3.84E-30 | 62.56734815 | MEA1 |
| 214131_at | 2.003505857 | 5.144974672 | 4.330527495 | 5.47E-05 | 9.57E-05 | 0.259664816 | TXLNGY |
| 205883_at | -2.003316272 | 6.831885976 | -6.260369172 | 3.87E-08 | 9.40E-08 | 7.38060016 | ZBTB16 |
| 224939_at | 2.003214005 | 8.299449044 | 15.08030167 | 1.56E-22 | 2.43E-21 | 40.61764232 | NUFIP2 |
| 203409_at | 2.003031654 | 6.22980619 | 9.271357741 | 2.30E-13 | 1.03E-12 | 19.3892005 | DDB2 |
| 204523_at | 2.002441876 | 6.032010069 | 15.74698215 | 1.81E-23 | 3.28E-22 | 42.78774274 | ZNF140 |
| 41047_at | 2.002362755 | 7.496877539 | 15.76946466 | 1.69E-23 | 3.07E-22 | 42.85996401 | C9orf16 |
| 210213_s_at | 2.002290035 | 7.369361251 | 19.73205349 | 1.36E-28 | 5.63E-27 | 54.66614194 | EIF6 |
| 200782_at | 2.001382648 | 9.227470117 | 10.20817759 | 5.88E-15 | 3.21E-14 | 23.06835718 | ANXA5 |
| 1557828_a_at | 2.001011104 | 5.200620917 | 12.07248407 | 5.24E-18 | 4.30E-17 | 30.12688944 | C5orf28 |
| 200893_at | 2.000382342 | 9.921421906 | 19.81747676 | 1.08E-28 | 4.57E-27 | 54.90166585 | TRA2B |
| 204163_at | 1.998679606 | 6.244563479 | 8.069423739 | 2.78E-11 | 9.71E-11 | 14.58782256 | EMILIN1 |
| 218384_at | 1.998503108 | 8.303904772 | 9.442648361 | 1.17E-13 | 5.45E-13 | 20.06716453 | CARHSP1 |
| 223392_s_at | 1.997701393 | 6.422621078 | 6.152179837 | 5.93E-08 | 1.41E-07 | 6.958676053 | TSHZ3 |
| 203136_at | 1.997541076 | 8.374180525 | 13.1203461 | 1.22E-19 | 1.25E-18 | 33.91224441 | RABAC1 |
| 202947_s_at | -1.997336879 | 7.034038169 | -10.33061709 | 3.66E-15 | 2.06E-14 | 23.54346182 | GYPC |
| 228620_at | 1.995894891 | 5.262582391 | 9.570724324 | 7.06E-14 | 3.39E-13 | 20.57267637 | LOC101927027 |
| 214807_at | 1.995866324 | 6.579494519 | 8.363659011 | 8.54E-12 | 3.17E-11 | 15.76816494 | PLXDC2 |
| 209095_at | -1.995734371 | 8.686624449 | -12.03345939 | 6.05E-18 | 4.91E-17 | 29.98327094 | DLD |
| 230288_at | 1.995610021 | 5.844997619 | 5.681690806 | 3.71E-07 | 8.13E-07 | 5.146991986 | FGF14 |
| 238429_at | 1.995539212 | 5.443013258 | 6.177332964 | 5.37E-08 | 1.29E-07 | 7.056612721 | TMEM71 |
| 218350_s_at | 1.995346067 | 7.226747642 | 9.691641253 | 4.39E-14 | 2.16E-13 | 21.0487548 | GMNN |
| 222476_at | 1.99490827 | 6.584212587 | 13.15401371 | 1.08E-19 | 1.12E-18 | 34.03156767 | CNOT6 |
| 221751_at | 1.994698939 | 7.594306073 | 14.66096514 | 6.24E-22 | 8.92E-21 | 39.22412708 | PANK3 |
| 1558801_at | -1.993845985 | 6.428473292 | -10.35783162 | 3.30E-15 | 1.87E-14 | 23.64886353 | NNT-AS1 |
| 209710_at | 1.992021365 | 7.335763172 | 6.032380914 | 9.48E-08 | 2.22E-07 | 6.493608188 | GATA2 |
| 207808_s_at | 1.991640832 | 5.891236282 | 7.68711273 | 1.29E-10 | 4.17E-10 | 13.05307899 | PROS1 |
| 225430_at | 1.991542534 | 7.337266187 | 17.89553754 | 2.51E-26 | 7.13E-25 | 49.41553406 | GATC |
| 203633_at | 1.991027964 | 7.552668333 | 14.24748389 | 2.49E-21 | 3.25E-20 | 37.82824653 | CPT1A |
| 206188_at | 1.990269351 | 4.676817463 | 14.41901399 | 1.40E-21 | 1.90E-20 | 38.40995912 | ZNF623 |
| 1555913_at | 1.990180419 | 6.35790274 | 15.16262366 | 1.19E-22 | 1.90E-21 | 40.88860904 | GON4L |
| 209678_s_at | 1.990178901 | 8.264142644 | 18.28828598 | 7.99E-27 | 2.48E-25 | 50.56925072 | PRKCI |
| 1555851_s_at | -1.989912201 | 10.56296408 | -15.30992837 | 7.40E-23 | 1.22E-21 | 41.37135027 | SEPW1 |
| 221517_s_at | 1.989656691 | 6.730272397 | 13.25108154 | 7.70E-20 | 8.14E-19 | 34.37478217 | MED17 |
| 201900_s_at | 1.98958827 | 8.049771888 | 18.86572261 | 1.53E-27 | 5.36E-26 | 52.23457792 | AKR1A1 |
| 227318_at | 1.989531553 | 6.456332044 | 5.890998442 | 1.65E-07 | 3.76E-07 | 5.947902549 | TMEM178B |
| 1553715_s_at | -1.988900961 | 7.043795195 | -15.43194712 | 4.98E-23 | 8.45E-22 | 41.76917286 | FAM195A |
| 226861_at | -1.988665426 | 7.357403076 | -20.10894933 | 4.89E-29 | 2.17E-27 | 55.69969649 | ASB8 |
| 204521_at | 1.987243224 | 6.170334721 | 9.409469585 | 1.33E-13 | 6.16E-13 | 19.936008 | FAM216A |
| 221024_s_at | 1.987166776 | 5.059399613 | 7.075777676 | 1.51E-09 | 4.28E-09 | 10.60517598 | SLC2A10 |
| 226995_at | 1.987075988 | 6.214968452 | 10.92295262 | 3.80E-16 | 2.40E-15 | 25.82035606 | LOC642852 |
| 200960_x_at | 1.986954807 | 9.746772488 | 23.67184606 | 5.72E-33 | 4.69E-31 | 64.80249192 | CLTA |
| 1557133_at | 1.986204642 | 5.127323469 | 4.888630579 | 7.38E-06 | 1.42E-05 | 2.208106458 | LINC00632 |
| 218242_s_at | 1.984719089 | 8.202638565 | 17.86803381 | 2.72E-26 | 7.67E-25 | 49.33409223 | SUV420H1 |
| 208723_at | 1.984315176 | 8.591261207 | 14.80746763 | 3.84E-22 | 5.67E-21 | 39.71349995 | USP11 |
| 204352_at | 1.98372873 | 5.898135881 | 8.39930585 | 7.41E-12 | 2.78E-11 | 15.91103261 | TRAF5 |
| 1553971_a_at | -1.983625965 | 5.69891078 | -12.66938433 | 6.05E-19 | 5.61E-18 | 32.30008489 | GATS |
| 224951_at | 1.98334412 | 7.416398591 | 25.13201429 | 1.91E-34 | 2.16E-32 | 68.21733891 | CERS5 |
| 203651_at | 1.983146513 | 7.181627583 | 11.30927557 | 8.84E-17 | 6.06E-16 | 27.2848399 | ZFYVE16 |
| 239650_at | 1.983084861 | 3.975017439 | 8.285746982 | 1.17E-11 | 4.27E-11 | 15.4557872 | NCKAP5 |
| 200619_at | 1.982961207 | 7.881767171 | 23.19056474 | 1.82E-32 | 1.41E-30 | 63.63902331 | SF3B2 |
| 224675_at | 1.981948733 | 7.543982994 | 14.93393943 | 2.53E-22 | 3.83E-21 | 40.13378038 | MESDC2 |
| 216941_s_at | 1.981546068 | 5.506881583 | 16.90217326 | 4.92E-25 | 1.15E-23 | 46.41906466 | TAF1B |
| 212082_s_at | 1.979411987 | 10.79870454 | 18.70016484 | 2.45E-27 | 8.23E-26 | 51.76082289 | MYL6 |
| 205573_s_at | 1.979186783 | 6.11991733 | 5.601662122 | 5.04E-07 | 1.09E-06 | 4.84317546 | SNX7 |
| 207400_at | 1.978813308 | 5.972472412 | 4.088621527 | 0.000125784 | 0.000211874 | -0.545851283 | NPY5R |
| 203145_at | 1.978806622 | 6.329678775 | 9.619499556 | 5.83E-14 | 2.83E-13 | 20.76485681 | SPAG5 |
| 210095_s_at | 1.978796091 | 7.808607572 | 7.084652685 | 1.45E-09 | 4.13E-09 | 10.64059209 | IGFBP3 |
| 226318_at | 1.978208979 | 7.169607251 | 15.22362412 | 9.79E-23 | 1.58E-21 | 41.0888468 | TBRG1 |
| 228030_at | 1.977715223 | 7.435695077 | 8.06143203 | 2.87E-11 | 1.00E-10 | 14.55574404 | RBM6 |
| 200021_at | 1.977080209 | 11.80384964 | 26.23029356 | 1.64E-35 | 2.19E-33 | 70.67810583 | CFL1 |
| 227833_s_at | 1.976419701 | 7.127924415 | 14.75964383 | 4.49E-22 | 6.56E-21 | 39.55404934 | MBD6 |
| 203286_at | 1.976396196 | 6.977669059 | 15.73863568 | 1.86E-23 | 3.36E-22 | 42.76091527 | RNF44 |
| 203701_s_at | 1.975720606 | 6.808470236 | 12.97886918 | 2.01E-19 | 1.99E-18 | 33.4092511 | TRMT1 |
| 236175_at | -1.974957597 | 3.473126862 | -19.13847636 | 7.08E-28 | 2.61E-26 | 53.00866406 | TRIM55 |
| 202465_at | 1.974945232 | 7.474179124 | 6.623163653 | 9.19E-09 | 2.39E-08 | 8.806903655 | PCOLCE |
| 212282_at | 1.974062834 | 7.120590629 | 8.40114745 | 7.35E-12 | 2.76E-11 | 15.91841251 | TMEM97 |
| 202710_at | 1.974017124 | 6.665007723 | 13.01489435 | 1.77E-19 | 1.77E-18 | 33.53757309 | BET1 |
| 223032_x_at | 1.973952521 | 7.988485677 | 21.57682716 | 1.02E-30 | 6.21E-29 | 59.59128567 | PRELID1 |
| 227894_at | 1.973559943 | 5.791597108 | 13.87294996 | 8.92E-21 | 1.08E-19 | 36.5450311 | WDR90 |
| 222028_at | 1.973009719 | 5.673376728 | 15.52809718 | 3.65E-23 | 6.32E-22 | 42.08134907 | ZNF45 |
| 230886_at | 1.972632996 | 5.735978496 | 14.34869981 | 1.77E-21 | 2.37E-20 | 38.17195504 | RP11-182L21.5 |
| 218100_s_at | 1.972191766 | 6.090810249 | 9.310117326 | 1.97E-13 | 8.95E-13 | 19.54279109 | IFT57 |
| 212571_at | 1.972190575 | 7.346880352 | 18.87168427 | 1.50E-27 | 5.31E-26 | 52.2515825 | CHD8 |
| 200793_s_at | -1.971056987 | 9.066012287 | -16.14619018 | 5.12E-24 | 1.00E-22 | 44.06090649 | ACO2 |
| 207559_s_at | 1.971051379 | 7.148034322 | 15.36304639 | 6.23E-23 | 1.04E-21 | 41.54476122 | ZMYM3 |
| 212442_s_at | 1.970014354 | 7.089980808 | 9.315736849 | 1.93E-13 | 8.77E-13 | 19.56505068 | CERS6 |
| 209550_at | 1.969832662 | 7.649474263 | 9.461935569 | 1.08E-13 | 5.07E-13 | 20.14336995 | NDN |
| 219937_at | 1.969446765 | 5.018112136 | 6.639030393 | 8.63E-09 | 2.26E-08 | 8.869633222 | TRHDE |
| 218689_at | 1.96902038 | 6.250487795 | 15.64014371 | 2.55E-23 | 4.51E-22 | 42.44369008 | FANCF |
| 228171_s_at | 1.968792234 | 6.225822634 | 10.41645054 | 2.63E-15 | 1.50E-14 | 23.8756439 | PLEKHG4 |
| 225842_at | 1.968097254 | 5.901408762 | 7.427768665 | 3.67E-10 | 1.12E-09 | 12.01296441 | PHLDA1 |
| 219258_at | 1.966369312 | 5.593058717 | 15.31049721 | 7.38E-23 | 1.22E-21 | 41.37320918 | TIPIN |
| 221558_s_at | 1.966342003 | 5.736392155 | 6.145870157 | 6.08E-08 | 1.45E-07 | 6.934123949 | LEF1 |
| 212875_s_at | 1.964486156 | 6.082101932 | 8.471964171 | 5.54E-12 | 2.11E-11 | 16.2021202 | C2CD2 |
| 212119_at | -1.964391174 | 9.053625814 | -14.46496649 | 1.20E-21 | 1.65E-20 | 38.5651621 | RHOQ |
| 202758_s_at | 1.963992315 | 7.597410783 | 16.67766163 | 9.80E-25 | 2.18E-23 | 45.72587428 | RFXANK |
| 1556474_a_at | 1.963399947 | 4.788912313 | 5.586227886 | 5.35E-07 | 1.16E-06 | 4.78474503 | FLJ38379 |
| 228697_at | -1.962729931 | 7.934724172 | -6.155543289 | 5.85E-08 | 1.40E-07 | 6.971766387 | HINT3 |
| 201216_at | 1.962718055 | 8.252639406 | 19.06429576 | 8.72E-28 | 3.19E-26 | 52.79892235 | ERP29 |
| 201288_at | 1.962487379 | 8.188395705 | 9.689449909 | 4.43E-14 | 2.18E-13 | 21.04013746 | ARHGDIB |
| 226691_at | 1.962404786 | 7.344380007 | 16.08520491 | 6.20E-24 | 1.20E-22 | 43.86767142 | TNRC18 |
| 217945_at | -1.961936691 | 9.026019859 | -13.41453465 | 4.35E-20 | 4.77E-19 | 34.95000544 | BTBD1 |
| 204258_at | 1.961278834 | 6.948027159 | 12.72716979 | 4.92E-19 | 4.61E-18 | 32.50810258 | CHD1 |
| 223092_at | -1.961239348 | 8.052694253 | -6.74329791 | 5.70E-09 | 1.52E-08 | 9.282485029 | ANKH |
| 218905_at | 1.961037183 | 8.014940746 | 12.83369515 | 3.36E-19 | 3.22E-18 | 32.89047011 | INTS8 |
| 218768_at | 1.959748366 | 7.622661498 | 14.0244677 | 5.31E-21 | 6.68E-20 | 37.06631527 | NUP107 |
| 218308_at | 1.959524872 | 6.011267408 | 8.757782822 | 1.77E-12 | 7.11E-12 | 17.34531352 | TACC3 |
| 202443_x_at | 1.959476583 | 7.419638807 | 9.944456586 | 1.64E-14 | 8.45E-14 | 22.04021209 | NOTCH2 |
| 204526_s_at | -1.958891996 | 7.152159829 | -13.23452499 | 8.16E-20 | 8.58E-19 | 34.31632618 | TBC1D8 |
| 34764_at | 1.958532499 | 6.202652783 | 14.30118368 | 2.08E-21 | 2.75E-20 | 38.01076265 | LARS2 |
| 219848_s_at | 1.958027881 | 6.519007271 | 13.89934702 | 8.15E-21 | 9.92E-20 | 36.63605922 | ZNF432 |
| 212080_at | 1.956825786 | 7.551596296 | 18.17262553 | 1.12E-26 | 3.40E-25 | 50.23128173 | KMT2A |
| 226795_at | 1.956652135 | 5.984194871 | 14.02249642 | 5.35E-21 | 6.72E-20 | 37.05955209 | LRCH1 |
| 218729_at | 1.956635677 | 6.295392558 | 7.306765972 | 5.96E-10 | 1.77E-09 | 11.52836969 | LXN |
| 214751_at | 1.956405938 | 4.209161562 | 14.79155083 | 4.04E-22 | 5.96E-21 | 39.66046342 | ZNF468 |
| 212813_at | 1.955894757 | 7.659077899 | 7.230779183 | 8.09E-10 | 2.37E-09 | 11.22436936 | JAM3 |
| 213387_at | 1.955016841 | 5.693417217 | 13.0008606 | 1.86E-19 | 1.85E-18 | 33.48760436 | ATAD2B |
| 204372_s_at | 1.954870341 | 7.839125744 | 17.41594559 | 1.04E-25 | 2.66E-24 | 47.98307352 | KHSRP |
| 204767_s_at | 1.954622807 | 7.273345629 | 10.24815076 | 5.03E-15 | 2.78E-14 | 23.22362602 | FEN1 |
| 203117_s_at | 1.954168508 | 6.898915876 | 11.79932817 | 1.43E-17 | 1.10E-16 | 29.11772306 | PAN2 |
| 238653_at | 1.953864966 | 6.724133435 | 13.95326968 | 6.78E-21 | 8.37E-20 | 36.82173009 | LRIG2 |
| 223054_at | 1.952212314 | 7.524177808 | 14.80495947 | 3.87E-22 | 5.71E-21 | 39.70514459 | DNAJB11 |
| 227286_at | 1.951706103 | 6.526820355 | 17.03921584 | 3.24E-25 | 7.74E-24 | 46.83926313 | INO80E |
| 228329_at | 1.951637948 | 5.859274541 | 4.514207727 | 2.86E-05 | 5.16E-05 | 0.887886673 | DAB1 |
| 1554452_a_at | 1.949717393 | 7.062991913 | 12.49717578 | 1.12E-18 | 1.00E-17 | 31.67767125 | HILPDA |
| 225019_at | -1.94886036 | 7.240671693 | -7.723223935 | 1.12E-10 | 3.64E-10 | 13.19801117 | CAMK2D |
| 228661_s_at | 1.948336263 | 5.488843034 | 10.45068327 | 2.31E-15 | 1.33E-14 | 24.00792142 | LOC102606465 |
| 202107_s_at | 1.94755754 | 7.142116774 | 9.477219199 | 1.02E-13 | 4.80E-13 | 20.20373717 | MCM2 |
| 218235_s_at | -1.946605058 | 8.268820491 | -17.63052045 | 5.50E-26 | 1.48E-24 | 48.62721869 | UTP11L |
| 201358_s_at | 1.946298147 | 9.835651152 | 23.71346148 | 5.18E-33 | 4.33E-31 | 64.90218823 | COPB1 |
| 201389_at | 1.945581925 | 7.459249377 | 12.43597631 | 1.40E-18 | 1.24E-17 | 31.45558204 | ITGA5 |
| 202202_s_at | 1.944815023 | 6.440919066 | 5.985967374 | 1.14E-07 | 2.64E-07 | 6.314071161 | LAMA4 |
| 237687_at | -1.944671309 | 4.023336404 | -12.74854818 | 4.56E-19 | 4.29E-18 | 32.58495431 | LOC283737 |
| 229211_at | -1.944654604 | 6.84146702 | -15.57067887 | 3.19E-23 | 5.57E-22 | 42.21923487 | DUSP28 |
| 205728_at | 1.944373566 | 4.595368601 | 4.854566856 | 8.37E-06 | 1.59E-05 | 2.085965646 | TENM1 |
| 201795_at | 1.94131865 | 7.679896635 | 11.20368924 | 1.31E-16 | 8.81E-16 | 26.88625653 | LBR |
| 208782_at | 1.940343834 | 9.026198854 | 6.920917141 | 2.80E-09 | 7.73E-09 | 9.988042867 | FSTL1 |
| 201944_at | 1.940273588 | 7.905708244 | 11.97554754 | 7.47E-18 | 5.99E-17 | 29.76980042 | HEXB |
| 229269_x_at | 1.93929269 | 7.502763529 | 16.47345026 | 1.84E-24 | 3.92E-23 | 45.09014957 | SSBP4 |
| 226621_at | 1.939285342 | 6.285942583 | 7.922018158 | 5.03E-11 | 1.71E-10 | 13.99606644 | OSMR |
| 219357_at | 1.938973466 | 6.89703723 | 16.54620513 | 1.47E-24 | 3.18E-23 | 45.31721283 | GTPBP1 |
| 242201_at | 1.938164483 | 5.037885105 | 13.37263427 | 5.03E-20 | 5.48E-19 | 34.80287517 | PMS2P5 |
| 225493_at | 1.937934877 | 7.786789294 | 14.73825401 | 4.82E-22 | 7.01E-21 | 39.4826393 | CCNT1 |
| 234969_s_at | 1.937544411 | 8.746710539 | 13.19111654 | 9.50E-20 | 9.89E-19 | 34.16289851 | EPC1 |
| 218477_at | 1.937391221 | 6.42561925 | 9.645750199 | 5.26E-14 | 2.57E-13 | 20.8682092 | TMEM14A |
| 230304_at | 1.937242856 | 5.167161495 | 9.621107458 | 5.79E-14 | 2.81E-13 | 20.77118892 | CTB-31O20.2 |
| 205330_at | -1.937147192 | 7.081080178 | -5.669951822 | 3.88E-07 | 8.49E-07 | 5.102339411 | MN1 |
| 228049_x_at | 1.937015882 | 7.90088998 | 9.135487288 | 3.94E-13 | 1.73E-12 | 18.85000137 | SNHG19 |
| 51774_s_at | -1.936318574 | 7.28789772 | -25.00944037 | 2.52E-34 | 2.76E-32 | 67.93710874 | UBE2D4 |
| 229113_s_at | 1.936004116 | 6.325463987 | 13.4949092 | 3.29E-20 | 3.66E-19 | 35.23160692 | C1orf86 |
| 202026_at | -1.934979411 | 8.922748904 | -15.47836956 | 4.29E-23 | 7.34E-22 | 41.92003886 | SDHD |
| 218979_at | 1.934953845 | 6.302263938 | 11.12153312 | 1.79E-16 | 1.18E-15 | 26.5752417 | RMI1 |
| 219530_at | 1.934370692 | 6.111910088 | 18.07455378 | 1.49E-26 | 4.41E-25 | 49.94354169 | PALB2 |
| 217884_at | 1.933706212 | 7.110524367 | 14.37999836 | 1.60E-21 | 2.15E-20 | 38.2779742 | NAT10 |
| 203789_s_at | -1.933617895 | 6.173327785 | -4.304782726 | 5.98E-05 | 0.000104161 | 0.172723298 | SEMA3C |
| 202613_at | 1.931729938 | 6.051946474 | 9.641442839 | 5.35E-14 | 2.61E-13 | 20.85125433 | CTPS1 |
| 227943_at | 1.93135725 | 4.79919475 | 8.314478142 | 1.04E-11 | 3.82E-11 | 15.57099849 | RP11-196G18.24 |
| 227126_at | 1.931208188 | 5.767225878 | 7.479620946 | 2.98E-10 | 9.18E-10 | 12.22078228 | PTPRG |
| 208820_at | 1.930878967 | 8.145476084 | 15.3904273 | 5.70E-23 | 9.62E-22 | 41.63401244 | PTK2 |
| 218515_at | 1.930742623 | 7.276168066 | 11.44672938 | 5.29E-17 | 3.76E-16 | 27.80179366 | PAXBP1 |
| 212345_s_at | 1.930742078 | 7.365627957 | 8.855405875 | 1.20E-12 | 4.92E-12 | 17.73495708 | CREB3L2 |
| 224900_at | 1.930314523 | 7.523592883 | 16.03386372 | 7.29E-24 | 1.39E-22 | 43.70464289 | ANKFY1 |
| 227926_s_at | 1.930275858 | 5.500019228 | 11.75715031 | 1.67E-17 | 1.28E-16 | 28.96109242 | NBPF20 |
| 229484_at | -1.930176907 | 5.219221731 | -21.65140288 | 8.43E-31 | 5.22E-29 | 59.78351325 | PPM1J |
| 208675_s_at | 1.930082246 | 8.543441999 | 15.26090299 | 8.67E-23 | 1.41E-21 | 41.21098761 | DDOST |
| 206209_s_at | -1.929482996 | 4.801537917 | -14.89294636 | 2.89E-22 | 4.35E-21 | 39.99777682 | CA4 |
| 1570289_at | -1.929100248 | 3.807588676 | -15.97241082 | 8.85E-24 | 1.68E-22 | 43.50908258 | LOC646736 |
| 1569003_at | 1.928579715 | 6.143079007 | 7.163367762 | 1.06E-09 | 3.06E-09 | 10.95491344 | VMP1 |
| 204114_at | 1.927207294 | 6.381825157 | 5.017877207 | 4.58E-06 | 8.99E-06 | 2.674956568 | NID2 |
| 213476_x_at | 1.926842018 | 8.560126723 | 11.5923971 | 3.07E-17 | 2.26E-16 | 28.3472276 | TUBB3 |
| 217759_at | 1.926328464 | 7.758365585 | 16.80148243 | 6.70E-25 | 1.53E-23 | 46.10891585 | TRIM44 |
| 201553_s_at | 1.926067479 | 10.05859093 | 13.57206648 | 2.51E-20 | 2.84E-19 | 35.50115907 | LAMP1 |
| 225469_at | -1.926014174 | 8.173008447 | -13.84491961 | 9.82E-21 | 1.18E-19 | 36.44827315 | LYRM5 |
| 218304_s_at | -1.925755862 | 6.94808037 | -11.80828743 | 1.38E-17 | 1.07E-16 | 29.15096639 | OSBPL11 |
| 221816_s_at | 1.924897672 | 7.718222522 | 8.814935045 | 1.41E-12 | 5.73E-12 | 17.57348245 | PHF11 |
| 205345_at | 1.923669456 | 6.787037297 | 9.919749335 | 1.80E-14 | 9.27E-14 | 21.9435628 | BARD1 |
| 202396_at | 1.923411414 | 8.352892207 | 12.37147711 | 1.77E-18 | 1.54E-17 | 31.22101176 | TCERG1 |
| 223190_s_at | 1.923142958 | 7.573491225 | 16.28208134 | 3.34E-24 | 6.81E-23 | 44.4898601 | KMT2E |
| 227792_at | 1.923104015 | 7.959461419 | 9.616635908 | 5.90E-14 | 2.85E-13 | 20.75357889 | ITPRIPL2 |
| 1553447_at | -1.92288034 | 3.798230839 | -16.15423686 | 4.99E-24 | 9.80E-23 | 44.08636904 | AGBL1 |
| 201385_at | 1.922685169 | 9.488382195 | 19.81211172 | 1.10E-28 | 4.62E-27 | 54.88689569 | DHX15 |
| 226782_at | -1.922437803 | 6.838754895 | -11.21393961 | 1.26E-16 | 8.49E-16 | 26.92500708 | SLC25A30 |
| 38340_at | 1.920850613 | 7.55963448 | 9.654481076 | 5.08E-14 | 2.48E-13 | 20.90257159 | HIP1R |
| 218088_s_at | 1.920821918 | 7.778282461 | 18.18821131 | 1.07E-26 | 3.26E-25 | 50.27691127 | RRAGC |
| 203766_s_at | -1.919179663 | 5.543311099 | -9.859279589 | 2.28E-14 | 1.16E-13 | 21.70679127 | LMOD1 |
| 212770_at | 1.919064602 | 6.557707193 | 14.67791107 | 5.89E-22 | 8.47E-21 | 39.28087184 | TLE3 |
| 227139_s_at | 1.918864451 | 5.800108121 | 13.10663263 | 1.28E-19 | 1.31E-18 | 33.86360037 | HPS3 |
| 212097_at | 1.918600063 | 10.44069556 | 5.974178228 | 1.19E-07 | 2.76E-07 | 6.268528025 | CAV1 |
| 203013_at | 1.918302481 | 6.807800121 | 9.049839476 | 5.53E-13 | 2.38E-12 | 18.50950754 | ECD |
| 209392_at | 1.918294177 | 6.878859075 | 5.043743454 | 4.16E-06 | 8.21E-06 | 2.769017385 | ENPP2 |
| 202645_s_at | 1.918081211 | 6.892948793 | 17.11030239 | 2.62E-25 | 6.32E-24 | 47.05635927 | MEN1 |
| 209382_at | 1.91797196 | 6.260043454 | 15.13235932 | 1.32E-22 | 2.08E-21 | 40.78909138 | POLR3C |
| 227109_at | 1.917270719 | 6.208213093 | 12.37988568 | 1.71E-18 | 1.50E-17 | 31.25162143 | CYP2R1 |
| 228174_at | 1.915665558 | 5.547407887 | 10.62873463 | 1.16E-15 | 6.93E-15 | 24.69399237 | SCAI |
| 212971_at | 1.915408879 | 8.87768772 | 16.99400597 | 3.72E-25 | 8.81E-24 | 46.70088529 | CARS |
| 1555533_at | 1.915301783 | 4.8190055 | 4.194580017 | 8.76E-05 | 0.000149803 | -0.196213569 | QRFPR |
| 227717_at | -1.914175515 | 5.585072262 | -9.955647136 | 1.57E-14 | 8.11E-14 | 22.08396913 | ARHGEF37 |
| 213316_at | 1.913140617 | 5.375584882 | 6.110563484 | 6.98E-08 | 1.65E-07 | 6.796855001 | KIAA1462 |
| 38269_at | 1.911835716 | 6.674185789 | 17.45424394 | 9.29E-26 | 2.40E-24 | 48.09843166 | PRKD2 |
| 213923_at | 1.911787099 | 6.429577275 | 12.91094287 | 2.56E-19 | 2.50E-18 | 33.16684847 | RAP2B |
| 203022_at | 1.911735929 | 7.358178341 | 10.71926061 | 8.24E-16 | 4.99E-15 | 25.04154253 | RNASEH2A |
| 204749_at | 1.911130536 | 4.316515861 | 6.271711886 | 3.70E-08 | 9.01E-08 | 7.424934867 | NAP1L3 |
| 206029_at | -1.910323937 | 3.792260096 | -8.877815176 | 1.10E-12 | 4.53E-12 | 17.82433208 | ANKRD1 |
| 213373_s_at | 1.909104754 | 5.672432597 | 9.017201074 | 6.29E-13 | 2.69E-12 | 18.37963709 | CASP8 |
| 225285_at | 1.907183875 | 5.143418063 | 5.196224929 | 2.35E-06 | 4.76E-06 | 3.327560546 | BCAT1 |
| 208851_s_at | 1.907114942 | 7.925471209 | 7.729708899 | 1.09E-10 | 3.55E-10 | 13.2240403 | THY1 |
| 214672_at | 1.905886899 | 6.160818154 | 16.16625928 | 4.81E-24 | 9.47E-23 | 44.12439757 | TTLL5 |
| 206698_at | -1.904828802 | 3.757558226 | -10.66500375 | 1.01E-15 | 6.06E-15 | 24.83334172 | XK |
| 203912_s_at | -1.904277504 | 6.930580464 | -11.40730615 | 6.13E-17 | 4.31E-16 | 27.65375041 | DNASE1L1 |
| 226571_s_at | 1.90412104 | 6.734177812 | 12.50468035 | 1.09E-18 | 9.80E-18 | 31.7048726 | PTPRS |
| 228531_at | 1.902479172 | 5.323086116 | 9.21138156 | 2.91E-13 | 1.30E-12 | 19.15133544 | SAMD9 |
| 201097_s_at | 1.902253277 | 9.410445775 | 14.88249512 | 2.99E-22 | 4.48E-21 | 39.96306861 | ARF4 |
| 201289_at | 1.901133208 | 7.304934665 | 4.213685362 | 8.20E-05 | 0.000140674 | -0.132633081 | CYR61 |
| 224738_x_at | 1.900728447 | 9.474732364 | 18.66240336 | 2.72E-27 | 9.05E-26 | 51.65235028 | RPL7L1 |
| 201438_at | 1.900271323 | 9.36211779 | 5.422271522 | 1.00E-06 | 2.10E-06 | 4.167475494 | COL6A3 |
| 202744_at | -1.90007431 | 5.334839185 | -12.97303469 | 2.05E-19 | 2.03E-18 | 33.38845305 | SLC20A2 |
| 224806_at | 1.897770634 | 7.995279699 | 11.17960844 | 1.44E-16 | 9.56E-16 | 26.79517422 | TRIM25 |
| 201063_at | 1.897688092 | 7.397078046 | 8.079248497 | 2.67E-11 | 9.36E-11 | 14.62725798 | RCN1 |
| 226582_at | 1.897655385 | 7.211995745 | 10.16383401 | 6.98E-15 | 3.78E-14 | 22.89593308 | LOC400043 |
| 205443_at | 1.896199017 | 6.24190194 | 12.66363591 | 6.17E-19 | 5.72E-18 | 32.27936853 | SNAPC1 |
| 201892_s_at | 1.894923964 | 9.921737191 | 10.02552998 | 1.19E-14 | 6.28E-14 | 22.35696838 | IMPDH2 |
| 237257_at | -1.893309879 | 5.783814985 | -21.30553079 | 2.05E-30 | 1.22E-28 | 58.88761184 | RAB4B |
| 209897_s_at | 1.892792414 | 7.001660466 | 6.521514147 | 1.38E-08 | 3.52E-08 | 8.405672406 | SLIT2 |
| 201916_s_at | 1.891986822 | 7.425257633 | 12.94510492 | 2.26E-19 | 2.23E-18 | 33.28883277 | SEC63 |
| 226194_at | 1.891871301 | 6.908400591 | 14.84228817 | 3.42E-22 | 5.09E-21 | 39.82941412 | CHAMP1 |
| 212587_s_at | 1.891774339 | 5.559107118 | 5.091796124 | 3.48E-06 | 6.91E-06 | 2.944296563 | PTPRC |
| 212094_at | 1.890478398 | 5.937289754 | 4.430111151 | 3.85E-05 | 6.86E-05 | 0.598565097 | PEG10 |
| 202406_s_at | 1.890397542 | 8.304085231 | 16.70754536 | 8.94E-25 | 2.01E-23 | 45.81848688 | TIAL1 |
| 203250_at | 1.889995886 | 7.240576275 | 16.08932676 | 6.12E-24 | 1.19E-22 | 43.88074599 | SCAF8 |
| 242560_at | 1.889311901 | 5.465180963 | 9.93832697 | 1.68E-14 | 8.64E-14 | 22.01623945 | FANCD2 |
| 235158_at | 1.88855389 | 5.887528509 | 10.29846751 | 4.14E-15 | 2.32E-14 | 23.41885263 | TMEM209 |
| 202621_at | 1.888529095 | 7.049555477 | 16.26320718 | 3.55E-24 | 7.17E-23 | 44.43041581 | IRF3 |
| 213924_at | -1.888309908 | 5.017533531 | -11.50676364 | 4.23E-17 | 3.04E-16 | 28.02688673 | GNAL |
| 203449_s_at | 1.886601035 | 7.007906092 | 14.04305412 | 4.99E-21 | 6.29E-20 | 37.13005818 | TERF1 |
| 206316_s_at | 1.88646193 | 5.911470593 | 11.0644821 | 2.22E-16 | 1.44E-15 | 26.35881782 | KNTC1 |
| 224777_s_at | 1.886315774 | 8.006024792 | 17.00150455 | 3.64E-25 | 8.65E-24 | 46.72385346 | PAFAH1B2 |
| 238620_at | 1.886145377 | 5.772836239 | 6.760649679 | 5.31E-09 | 1.42E-08 | 9.351291076 | RP11-846E15.4 |
| 202763_at | 1.885954226 | 6.059693725 | 16.08900181 | 6.13E-24 | 1.19E-22 | 43.87971533 | CASP3 |
| 231935_at | -1.8858676 | 5.784212014 | -4.704992661 | 1.44E-05 | 2.69E-05 | 1.554315923 | ARPP21 |
| 225767_at | 1.8855525 | 6.42657477 | 7.113863556 | 1.29E-09 | 3.70E-09 | 10.75719328 | RNA45S5 |
| 212754_s_at | 1.88492654 | 6.868971533 | 16.40319435 | 2.29E-24 | 4.79E-23 | 44.87028216 | MON2 |
| 1556271_at | -1.883006478 | 4.486221365 | -19.06078368 | 8.81E-28 | 3.22E-26 | 52.78897763 | LOC101928896 |
| 224933_s_at | 1.882898488 | 7.600333478 | 12.52570351 | 1.01E-18 | 9.12E-18 | 31.78103641 | JMJD1C |
| 228195_at | -1.880443665 | 4.560357329 | -13.36390907 | 5.19E-20 | 5.63E-19 | 34.77220903 | C2orf88 |
| 223165_s_at | 1.880426709 | 7.303644354 | 11.43765681 | 5.47E-17 | 3.88E-16 | 27.76774018 | IP6K2 |
| 225439_at | 1.880142692 | 6.898422603 | 10.78586703 | 6.39E-16 | 3.92E-15 | 25.29670409 | NUDCD1 |
| 210444_at | -1.880106021 | 4.99042361 | -21.17053092 | 2.92E-30 | 1.66E-28 | 58.5348757 | NPY6R |
| 225350_s_at | -1.879906582 | 8.697426672 | -17.98661558 | 1.92E-26 | 5.58E-25 | 49.68461832 | ZYG11B |
| 50374_at | 1.879534285 | 6.646123498 | 14.53723304 | 9.42E-22 | 1.32E-20 | 38.80869658 | OXLD1 |
| 218802_at | 1.878323717 | 6.171585395 | 9.103773141 | 4.46E-13 | 1.95E-12 | 18.72397386 | CCDC109B |
| 208974_x_at | 1.877429687 | 9.68636279 | 19.17150424 | 6.46E-28 | 2.40E-26 | 53.10186075 | KPNB1 |
| 211509_s_at | -1.877055976 | 10.65217393 | -12.53635909 | 9.75E-19 | 8.81E-18 | 31.81961885 | RTN4 |
| 208804_s_at | 1.876877317 | 8.800832128 | 14.23773721 | 2.58E-21 | 3.35E-20 | 37.79507987 | SRSF6 |
| 56256_at | 1.876683971 | 7.868775558 | 11.72140394 | 1.91E-17 | 1.44E-16 | 28.82817821 | SIDT2 |
| 202379_s_at | 1.876628996 | 9.340693573 | 15.14413629 | 1.27E-22 | 2.01E-21 | 40.82783104 | NKTR |
| 227102_at | 1.876407048 | 6.634736627 | 7.686686776 | 1.30E-10 | 4.18E-10 | 13.05136953 | TRIM35 |
| 212738_at | 1.876136789 | 6.217870867 | 14.87195712 | 3.10E-22 | 4.63E-21 | 39.92805829 | ARHGAP19 |
| 203349_s_at | 1.87567265 | 7.010030077 | 5.568580376 | 5.73E-07 | 1.23E-06 | 4.71800183 | ETV5 |
| 209408_at | 1.875361371 | 6.416587177 | 8.338783616 | 9.44E-12 | 3.49E-11 | 15.66844712 | KIF2C |
| 212077_at | 1.875205827 | 9.153147366 | 6.839302381 | 3.88E-09 | 1.05E-08 | 9.663514368 | CALD1 |
| 201304_at | -1.87515701 | 9.044916795 | -12.52395824 | 1.02E-18 | 9.18E-18 | 31.77471566 | NDUFA5 |
| 213074_at | 1.87377481 | 8.610631009 | 15.05578491 | 1.69E-22 | 2.62E-21 | 40.53677975 | PHIP |
| 204566_at | 1.873361077 | 5.770034841 | 9.781139092 | 3.09E-14 | 1.55E-13 | 21.40035946 | PPM1D |
| 202809_s_at | 1.873104905 | 8.133025904 | 17.01932409 | 3.45E-25 | 8.21E-24 | 46.77840827 | INTS3 |
| 215696_s_at | 1.872283909 | 6.78198523 | 16.95025465 | 4.25E-25 | 1.00E-23 | 46.56674303 | SEC16A |
| 209630_s_at | 1.87203552 | 6.784325477 | 13.56395058 | 2.59E-20 | 2.92E-19 | 35.4728417 | FBXW2 |
| 218886_at | 1.871336824 | 6.865169057 | 12.05956348 | 5.50E-18 | 4.49E-17 | 30.0793598 | PAK1IP1 |
| 211475_s_at | -1.871291624 | 7.383454245 | -17.32628822 | 1.36E-25 | 3.44E-24 | 47.712355 | BAG1 |
| 232113_at | 1.871234931 | 6.233120096 | 4.389313865 | 4.45E-05 | 7.86E-05 | 0.459230976 | AK021804 |
| 204491_at | -1.871140426 | 7.341131297 | -8.426642651 | 6.64E-12 | 2.51E-11 | 16.02056986 | PDE4D |
| 203278_s_at | 1.870943882 | 7.278247944 | 15.19084987 | 1.09E-22 | 1.75E-21 | 40.98132127 | PHF21A |
| 203316_s_at | 1.870913647 | 9.680838501 | 10.39954917 | 2.81E-15 | 1.60E-14 | 23.81029241 | SNRPE |
| 216241_s_at | 1.870836394 | 9.11937376 | 13.8186964 | 1.07E-20 | 1.28E-19 | 36.35766224 | TCEA1 |
| 228330_at | 1.87004316 | 5.265659647 | 13.55966283 | 2.63E-20 | 2.96E-19 | 35.45787778 | ZUFSP |
| 227536_at | 1.869993854 | 8.372577558 | 7.880638891 | 5.94E-11 | 2.00E-10 | 13.82993705 | ZC3H13 |
| 214602_at | -1.869503635 | 4.054614185 | -9.246636096 | 2.53E-13 | 1.13E-12 | 19.29118383 | COL4A4 |
| 201050_at | 1.868929848 | 8.296139226 | 11.00132004 | 2.82E-16 | 1.81E-15 | 26.11878664 | PLD3 |
| 211767_at | 1.868184846 | 5.603227775 | 8.312423971 | 1.05E-11 | 3.85E-11 | 15.56276196 | GINS4 |
| 242947_at | -1.868055834 | 3.363166164 | -18.51258802 | 4.18E-27 | 1.35E-25 | 51.220465 | RP4-680D5.8 |
| 211034_s_at | 1.867658711 | 7.277431236 | 11.78560946 | 1.50E-17 | 1.16E-16 | 29.0668011 | HECTD4 |
| 212890_at | 1.86737413 | 6.588002043 | 14.49152606 | 1.10E-21 | 1.52E-20 | 38.6547436 | SLC38A10 |
| 209030_s_at | 1.867121237 | 7.428844399 | 5.720738683 | 3.19E-07 | 7.04E-07 | 5.295732594 | CADM1 |
| 206314_at | 1.866460534 | 5.399156068 | 8.520887956 | 4.55E-12 | 1.75E-11 | 16.39802458 | ZKSCAN7 |
| 212589_at | -1.86620302 | 6.334010992 | -8.202729138 | 1.63E-11 | 5.85E-11 | 15.12278594 | RRAS2 |
| 213400_s_at | 1.866180487 | 6.539652488 | 10.64770474 | 1.08E-15 | 6.46E-15 | 24.76689453 | TBL1X |
| 200978_at | -1.865993471 | 10.17790206 | -13.81074954 | 1.10E-20 | 1.31E-19 | 36.33018553 | MDH1 |
| 230434_at | 1.865634147 | 5.839080142 | 9.938159488 | 1.68E-14 | 8.64E-14 | 22.01558439 | PHOSPHO2 |
| 235457_at | 1.865167478 | 5.402395859 | 6.147568689 | 6.03E-08 | 1.44E-07 | 6.940732631 | MAML2 |
| 209780_at | -1.864981161 | 8.343929106 | -13.98543545 | 6.07E-21 | 7.55E-20 | 36.93230881 | PHTF2 |
| 1554126_at | -1.864829615 | 6.951875411 | -27.6833074 | 7.27E-37 | 1.20E-34 | 73.80169437 | MSRB3 |
| 218008_at | 1.864818018 | 7.06433746 | 14.56895623 | 8.47E-22 | 1.19E-20 | 38.91539245 | TMEM248 |
| 223081_at | 1.864649781 | 7.13067849 | 18.00694921 | 1.81E-26 | 5.30E-25 | 49.74456532 | PHF23 |
| 224689_at | 1.863940526 | 7.072658672 | 15.81352452 | 1.47E-23 | 2.68E-22 | 43.00131838 | MANBAL |
| 206070_s_at | 1.862919525 | 5.251964013 | 4.829740846 | 9.16E-06 | 1.74E-05 | 1.997191923 | EPHA3 |
| 211967_at | 1.862013526 | 7.750185355 | 10.88148723 | 4.44E-16 | 2.79E-15 | 25.66217859 | TMEM123 |
| 227334_at | -1.860642534 | 7.193796289 | -10.30391881 | 4.06E-15 | 2.27E-14 | 23.4399886 | USP54 |
| 225050_at | 1.860272345 | 7.371122374 | 11.09880957 | 1.95E-16 | 1.28E-15 | 26.48908342 | ZNF512 |
| 244741_s_at | 1.858515767 | 6.149620239 | 8.781256578 | 1.61E-12 | 6.51E-12 | 17.43904684 | ZNF667-AS1 |
| 201098_at | 1.858463781 | 8.064061467 | 16.41918709 | 2.18E-24 | 4.57E-23 | 44.92038397 | COPB2 |
| 226169_at | 1.857492953 | 6.745117924 | 11.0695204 | 2.18E-16 | 1.42E-15 | 26.37794541 | SBF2 |
| 227810_at | 1.856010658 | 6.315766394 | 13.406842 | 4.47E-20 | 4.89E-19 | 34.92301006 | ZNF558 |
| 239752_at | -1.855765745 | 5.271549925 | -6.454227178 | 1.80E-08 | 4.53E-08 | 8.140724568 | CECR2 |
| 212846_at | 1.855337336 | 7.922849094 | 11.55169787 | 3.58E-17 | 2.60E-16 | 28.19508635 | RRP1B |
| 201112_s_at | 1.854803713 | 8.987146028 | 13.24358779 | 7.90E-20 | 8.33E-19 | 34.34832844 | CSE1L |
| 201583_s_at | 1.854191586 | 6.345334454 | 13.84820665 | 9.71E-21 | 1.17E-19 | 36.45962488 | SEC23B |
| 202023_at | 1.854095668 | 6.578978965 | 10.01651046 | 1.24E-14 | 6.49E-14 | 22.32175828 | EFNA1 |
| 223253_at | -1.853741546 | 7.585648291 | -8.568687663 | 3.76E-12 | 1.46E-11 | 16.58934602 | EPDR1 |
| 232524_x_at | 1.853165538 | 7.751759881 | 11.81445118 | 1.35E-17 | 1.05E-16 | 29.17383131 | ANAPC4 |
| 224673_at | 1.853028451 | 6.065028421 | 9.226584552 | 2.74E-13 | 1.22E-12 | 19.21165309 | LENG8 |
| 209509_s_at | 1.852956455 | 6.036493034 | 17.60628104 | 5.91E-26 | 1.58E-24 | 48.5547175 | DPAGT1 |
| 224452_s_at | -1.852708387 | 8.579831978 | -15.10031733 | 1.46E-22 | 2.29E-21 | 40.68360307 | FAM220A |
| 202679_at | 1.852070949 | 5.842022089 | 16.53690699 | 1.51E-24 | 3.27E-23 | 45.28822931 | NPC1 |
| 212476_at | 1.851618584 | 6.645872567 | 13.59014132 | 2.36E-20 | 2.68E-19 | 35.56419418 | ACAP2 |
| 222986_s_at | 1.851591734 | 7.760232074 | 17.56371862 | 6.70E-26 | 1.77E-24 | 48.42724864 | SHISA5 |
| 200015_s_at | 1.851384032 | 9.786596613 | 22.68378365 | 6.29E-32 | 4.51E-30 | 62.39264489 | SEPT2 |
| 218130_at | 1.851281901 | 5.804412215 | 15.76005612 | 1.74E-23 | 3.15E-22 | 42.82974824 | C17orf62 |
| 229442_at | 1.849064082 | 5.594413426 | 10.45603939 | 2.26E-15 | 1.30E-14 | 24.02860711 | C18orf54 |
| 213338_at | 1.848981666 | 6.141197046 | 5.638550416 | 4.38E-07 | 9.53E-07 | 4.983041681 | TMEM158 |
| 202854_at | 1.848923348 | 7.628197449 | 11.49536224 | 4.41E-17 | 3.17E-16 | 27.98417077 | HPRT1 |
| 201007_at | -1.848349616 | 9.852542954 | -13.59397234 | 2.33E-20 | 2.65E-19 | 35.57754932 | HADHB |
| 225205_at | 1.847747251 | 6.964575408 | 16.82530138 | 6.23E-25 | 1.43E-23 | 46.18239151 | KIF3B |
| 230918_at | 1.847602586 | 6.145690218 | 12.37511332 | 1.74E-18 | 1.52E-17 | 31.23424972 | GALK2 |
| 212063_at | 1.847491115 | 7.545461057 | 5.146281422 | 2.84E-06 | 5.69E-06 | 3.143869283 | CD44 |
| 218333_at | 1.847227426 | 7.170719713 | 16.78473057 | 7.05E-25 | 1.61E-23 | 46.05720021 | DERL2 |
| 230964_at | -1.847149019 | 3.687104239 | -7.342376809 | 5.17E-10 | 1.55E-09 | 11.6709257 | FREM2 |
| 221096_s_at | 1.846756396 | 5.475720337 | 11.17274793 | 1.48E-16 | 9.80E-16 | 26.76921323 | TMCO6 |
| 230151_at | -1.846465192 | 4.171506844 | -18.15806515 | 1.17E-26 | 3.52E-25 | 50.18862978 | SPRYD7 |
| 209124_at | 1.846398163 | 7.705423264 | 17.09852691 | 2.71E-25 | 6.52E-24 | 47.02043822 | MYD88 |
| 218566_s_at | 1.845743766 | 7.490358139 | 8.325566889 | 9.95E-12 | 3.66E-11 | 15.61545876 | CHORDC1 |
| 203832_at | 1.844969438 | 8.245540854 | 12.90340534 | 2.63E-19 | 2.56E-18 | 33.13991387 | SNRPF |
| 232676_x_at | 1.844812848 | 6.967272235 | 7.512605816 | 2.61E-10 | 8.10E-10 | 12.35302389 | MYEF2 |
| 212865_s_at | 1.844644759 | 5.528633838 | 4.253297233 | 7.15E-05 | 0.00012352 | -0.000297242 | COL14A1 |
| 212918_at | 1.844157295 | 7.310181774 | 14.83018614 | 3.56E-22 | 5.28E-21 | 39.789145 | RECQL |
| 209572_s_at | 1.843761469 | 6.916464327 | 14.48190652 | 1.13E-21 | 1.57E-20 | 38.62230868 | EED |
| 202939_at | 1.843482752 | 7.659141796 | 15.5403597 | 3.51E-23 | 6.10E-22 | 42.12107998 | ZMPSTE24 |
| 201751_at | 1.843140618 | 7.567243127 | 17.26937356 | 1.62E-25 | 4.03E-24 | 47.5400194 | JOSD1 |
| 205667_at | 1.843091693 | 5.96466918 | 14.14052674 | 3.58E-21 | 4.59E-20 | 37.46362179 | WRN |
| 203690_at | 1.842572097 | 6.975609702 | 12.32421349 | 2.10E-18 | 1.80E-17 | 31.04879454 | TUBGCP3 |
| 219588_s_at | 1.841809536 | 5.939228914 | 11.405224 | 6.18E-17 | 4.34E-16 | 27.64592642 | NCAPG2 |
| 223110_at | 1.841338213 | 7.107793789 | 14.51074908 | 1.03E-21 | 1.43E-20 | 38.71952407 | KIAA1429 |
| 204711_at | 1.840789087 | 6.540737031 | 17.55389351 | 6.90E-26 | 1.81E-24 | 48.39779426 | KIAA0753 |
| 206227_at | -1.840429152 | 5.932716361 | -5.909230598 | 1.53E-07 | 3.51E-07 | 6.018072106 | CILP |
| 218355_at | 1.840378286 | 5.652868935 | 7.843203832 | 6.90E-11 | 2.31E-10 | 13.67964493 | KIF4A |
| 201462_at | 1.839640835 | 5.792886493 | 7.127322715 | 1.23E-09 | 3.52E-09 | 10.81093537 | SCRN1 |
| 225061_at | -1.839589939 | 7.409733335 | -10.14689066 | 7.45E-15 | 4.03E-14 | 22.83000177 | DNAJA4 |
| 214295_at | 1.839344887 | 7.094790399 | 7.772806989 | 9.16E-11 | 3.02E-10 | 13.3970375 | KIAA0485 |
| 225634_at | 1.839176058 | 7.280577639 | 10.10502278 | 8.76E-15 | 4.69E-14 | 22.66696618 | ZC3HAV1 |
| 209019_s_at | -1.838719904 | 7.965432714 | -19.03828237 | 9.39E-28 | 3.41E-26 | 52.72523225 | PINK1 |
| 218513_at | 1.838399427 | 4.072823355 | 8.604524135 | 3.26E-12 | 1.27E-11 | 16.73272747 | TMA16 |
| 208581_x_at | -1.838214171 | 8.481415043 | -7.818919806 | 7.61E-11 | 2.53E-10 | 13.58215356 | MT1X |
| 212502_at | 1.837920703 | 7.250898382 | 9.353261799 | 1.66E-13 | 7.61E-13 | 19.71363473 | ADO |
| 203732_at | 1.837517019 | 6.604468788 | 13.71268989 | 1.55E-20 | 1.79E-19 | 35.99047369 | TRIP4 |
| 203791_at | 1.837075714 | 6.763824066 | 9.195588195 | 3.10E-13 | 1.38E-12 | 19.08865941 | DMXL1 |
| 216210_x_at | 1.836272879 | 8.323545951 | 22.49215936 | 1.01E-31 | 7.02E-30 | 61.91555319 | TRIOBP |
| 223395_at | 1.835642243 | 6.415352036 | 4.530792205 | 2.70E-05 | 4.88E-05 | 0.94527156 | ABI3BP |
| 212696_s_at | 1.835477427 | 7.960232484 | 21.28255693 | 2.18E-30 | 1.28E-28 | 58.82770606 | RNF4 |
| 1555844_s_at | 1.835357692 | 9.146727355 | 17.6308564 | 5.49E-26 | 1.48E-24 | 48.62822307 | HNRNPM |
| 202963_at | 1.835175502 | 7.336945491 | 19.18848127 | 6.16E-28 | 2.30E-26 | 53.14972087 | RFX5 |
| 1553253_at | -1.834623576 | 6.222854211 | -24.81440304 | 3.94E-34 | 4.20E-32 | 67.48883412 | ASB16 |
| 242208_at | 1.833438714 | 5.244567899 | 12.50436172 | 1.09E-18 | 9.80E-18 | 31.70371783 | ZNF37BP |
| 202220_at | 1.83294679 | 8.414154676 | 11.35011971 | 7.59E-17 | 5.25E-16 | 27.43868065 | KIAA0907 |
| 233375_at | -1.8327964 | 4.19137128 | -11.81889236 | 1.33E-17 | 1.03E-16 | 29.19030341 | EFCAB2 |
| 218830_at | 1.832544848 | 8.607035078 | 15.85290033 | 1.29E-23 | 2.39E-22 | 43.12744319 | RPL26L1 |
| 223915_at | 1.831927743 | 6.490928983 | 9.732924979 | 3.74E-14 | 1.85E-13 | 21.21102659 | BCOR |
| 218740_s_at | 1.83180676 | 7.866396599 | 15.47043058 | 4.40E-23 | 7.53E-22 | 41.89425736 | CDK5RAP3 |
| 235242_at | 1.831426504 | 6.887330636 | 10.47974099 | 2.06E-15 | 1.19E-14 | 24.12010932 | REL |
| 218231_at | 1.831400027 | 7.409999018 | 14.83543267 | 3.50E-22 | 5.20E-21 | 39.80660495 | NAGK |
| 201853_s_at | 1.83125322 | 7.254757005 | 10.35754296 | 3.30E-15 | 1.87E-14 | 23.6477459 | CDC25B |
| 209890_at | 1.83055702 | 5.814361854 | 6.312232968 | 3.15E-08 | 7.74E-08 | 7.583465721 | TSPAN5 |
| 205197_s_at | 1.830377163 | 6.209196845 | 12.06446988 | 5.40E-18 | 4.41E-17 | 30.09741087 | ATP7A |
| 204076_at | 1.830231687 | 7.559301046 | 13.67235832 | 1.78E-20 | 2.05E-19 | 35.85039438 | ENTPD4 |
| 201176_s_at | 1.829270266 | 8.311641185 | 17.29778754 | 1.49E-25 | 3.73E-24 | 47.6261029 | ARCN1 |
| 205812_s_at | 1.829089542 | 8.780965631 | 16.83481651 | 6.05E-25 | 1.39E-23 | 46.21172459 | TMED9 |
| 223000_s_at | 1.828650672 | 6.841818845 | 6.191434069 | 5.08E-08 | 1.22E-07 | 7.111559426 | F11R |
| 212412_at | 1.827581585 | 7.385148802 | 5.216365648 | 2.18E-06 | 4.42E-06 | 3.401837576 | PDLIM5 |
| 226106_at | 1.825895084 | 8.751267161 | 5.464695597 | 8.52E-07 | 1.80E-06 | 4.326577759 | RNF141 |
| 225625_at | 1.825850951 | 6.628275698 | 11.76810862 | 1.60E-17 | 1.23E-16 | 29.00180749 | ALKBH2 |
| 218708_at | 1.825834625 | 6.691298745 | 17.22468788 | 1.85E-25 | 4.57E-24 | 47.40444911 | NXT1 |
| 224413_s_at | 1.825665565 | 7.630626168 | 15.36358757 | 6.21E-23 | 1.04E-21 | 41.54652619 | TM2D2 |
| 1558605_at | 1.824782275 | 5.745179319 | 9.339955099 | 1.75E-13 | 8.00E-13 | 19.66095677 | CTD-2292M16.8 |
| 224088_at | -1.824596567 | 4.905629938 | -21.33514307 | 1.90E-30 | 1.13E-28 | 58.96475431 | NMUR2 |
| 220206_at | 1.824305892 | 4.713000013 | 15.13206649 | 1.32E-22 | 2.08E-21 | 40.78812794 | ZMYM1 |
| 33494_at | -1.823564253 | 6.260894409 | -11.5272164 | 3.92E-17 | 2.84E-16 | 28.10347584 | ETFDH |
| 223363_at | 1.823383892 | 6.83999508 | 15.12445598 | 1.35E-22 | 2.12E-21 | 40.76308408 | PSMG3 |
| 224686_x_at | 1.823095405 | 8.503425426 | 12.99721385 | 1.88E-19 | 1.87E-18 | 33.47461558 | LRRC37A2 |
| 218181_s_at | 1.823047233 | 7.248371956 | 10.98661066 | 2.98E-16 | 1.91E-15 | 26.06282368 | MAP4K4 |
| 206170_at | -1.822992786 | 4.311921831 | -11.48404589 | 4.60E-17 | 3.30E-16 | 27.94175829 | ADRB2 |
| 203276_at | 1.822309632 | 6.027876662 | 9.872313005 | 2.17E-14 | 1.11E-13 | 21.75785117 | LMNB1 |
| 225565_at | 1.821643369 | 7.608994749 | 19.91967485 | 8.17E-29 | 3.50E-27 | 55.18245951 | CREB1 |
| 1556103_at | -1.82129946 | 6.670134282 | -11.93639322 | 8.63E-18 | 6.85E-17 | 29.62524062 | RP11-480A16.1 |
| 212629_s_at | 1.82054316 | 7.819840468 | 14.63916893 | 6.70E-22 | 9.53E-21 | 39.15108729 | PKN2 |
| 202333_s_at | -1.820539542 | 9.36934317 | -18.78297771 | 1.93E-27 | 6.68E-26 | 51.99816772 | UBE2B |
| 203537_at | 1.819985691 | 6.854210607 | 15.53024862 | 3.63E-23 | 6.28E-22 | 42.08832115 | PRPSAP2 |
| 221564_at | 1.819316089 | 7.281174776 | 11.95162689 | 8.16E-18 | 6.50E-17 | 29.68150633 | PRMT2 |
| 210844_x_at | 1.81922442 | 9.427400971 | 16.74528021 | 7.96E-25 | 1.80E-23 | 45.93527913 | CTNNA1 |
| 216457_s_at | 1.819086823 | 7.587944256 | 15.5936899 | 2.96E-23 | 5.20E-22 | 42.29365442 | SF3A1 |
| 225416_at | 1.818670942 | 7.600463084 | 13.70353742 | 1.60E-20 | 1.84E-19 | 35.95870366 | RLIM |
| 230292_at | 1.818507813 | 6.485393863 | 10.54987397 | 1.58E-15 | 9.23E-15 | 24.39052807 | LOC100996412 |
| 218086_at | 1.817448278 | 7.587620401 | 11.7164532 | 1.94E-17 | 1.47E-16 | 28.809758 | NPDC1 |
| 218542_at | 1.81707953 | 4.95321275 | 7.984021493 | 3.92E-11 | 1.35E-10 | 14.24499211 | CEP55 |
| 229640_x_at | 1.815753498 | 6.507642735 | 15.43564704 | 4.92E-23 | 8.36E-22 | 41.7812069 | LINC00621 |
| 218820_at | 1.815663937 | 7.121084349 | 7.375247397 | 4.53E-10 | 1.37E-09 | 11.80255741 | C14orf132 |
| 223113_at | 1.815640707 | 6.235056851 | 15.6630876 | 2.37E-23 | 4.22E-22 | 42.51769532 | TMEM138 |
| 200825_s_at | 1.815038806 | 7.595336166 | 12.0225614 | 6.29E-18 | 5.10E-17 | 29.94313086 | HYOU1 |
| 208527_x_at | 1.814705586 | 5.990295317 | 8.594302289 | 3.39E-12 | 1.32E-11 | 16.69183502 | HIST1H2BE |
| 1555832_s_at | 1.814358895 | 8.326762106 | 8.192829936 | 1.70E-11 | 6.07E-11 | 15.08306923 | KLF6 |
| 215000_s_at | -1.813940979 | 8.592551137 | -8.76134252 | 1.74E-12 | 7.02E-12 | 17.35952947 | FEZ2 |
| 223028_s_at | 1.813775967 | 7.107228349 | 10.9936519 | 2.90E-16 | 1.86E-15 | 26.08961559 | SNX9 |
| 200750_s_at | 1.813390785 | 9.854876498 | 12.9145667 | 2.52E-19 | 2.47E-18 | 33.17979532 | RAN |
| 229393_at | 1.813313079 | 6.147544073 | 9.506265341 | 9.10E-14 | 4.31E-13 | 20.31841498 | L3MBTL3 |
| 208845_at | -1.812837673 | 10.87655563 | -18.30947501 | 7.51E-27 | 2.34E-25 | 50.63100595 | VDAC3 |
| 212522_at | 1.812663744 | 6.904041447 | 8.771977425 | 1.67E-12 | 6.75E-12 | 17.40199727 | PDE8A |
| 204297_at | 1.812493157 | 7.08634991 | 12.56626884 | 8.75E-19 | 7.96E-18 | 31.92784196 | PIK3C3 |
| 201594_s_at | 1.812365987 | 7.497561339 | 14.82652812 | 3.60E-22 | 5.34E-21 | 39.77696943 | PPP4R1 |
| 208815_x_at | 1.812309986 | 8.333725749 | 12.237453 | 2.87E-18 | 2.43E-17 | 30.73193777 | HSPA4 |
| 210858_x_at | 1.812183478 | 7.493454091 | 11.4192426 | 5.86E-17 | 4.13E-16 | 27.69859366 | ATM |
| 204151_x_at | -1.81125079 | 7.482498398 | -7.859617559 | 6.46E-11 | 2.17E-10 | 13.74554134 | AKR1C1 |
| 219025_at | 1.811245497 | 8.144226727 | 8.937053747 | 8.65E-13 | 3.63E-12 | 18.06046566 | CD248 |
| 202858_at | 1.810311625 | 9.212067291 | 16.30973873 | 3.07E-24 | 6.27E-23 | 44.57688939 | U2AF1 |
| 201700_at | 1.809554811 | 7.548186427 | 12.63439237 | 6.85E-19 | 6.31E-18 | 32.17391522 | CCND3 |
| 205010_at | 1.80952711 | 6.602986354 | 10.15215985 | 7.30E-15 | 3.95E-14 | 22.85050858 | GNL3L |
| 206652_at | 1.809276712 | 5.449345968 | 10.85762211 | 4.86E-16 | 3.04E-15 | 25.57105548 | ZMYM5 |
| 218190_s_at | -1.80832245 | 10.28432423 | -15.99073971 | 8.36E-24 | 1.59E-22 | 43.56745853 | UQCR10 |
| 209974_s_at | 1.808024759 | 8.09419606 | 18.95684938 | 1.18E-27 | 4.24E-26 | 52.49408396 | BUB3 |
| 200902_at | 1.807999773 | 8.558741519 | 11.71846901 | 1.93E-17 | 1.46E-16 | 28.81725857 | 15-Sep |
| 234295_at | 1.80783638 | 5.72852147 | 14.47730582 | 1.15E-21 | 1.59E-20 | 38.606792 | DBR1 |
| 213623_at | 1.806296738 | 5.603839037 | 11.99125822 | 7.06E-18 | 5.68E-17 | 29.82775249 | KIF3A |
| 214737_x_at | 1.806089747 | 9.79490144 | 17.704805 | 4.41E-26 | 1.21E-24 | 48.84898943 | HNRNPC |
| 1557480_a_at | -1.8060607 | 5.899852517 | -13.98909485 | 6.00E-21 | 7.47E-20 | 36.94488061 | PPP1R27 |
| 223437_at | -1.80490175 | 6.079584903 | -10.15906807 | 7.11E-15 | 3.85E-14 | 22.87739023 | PPARA |
| 221619_s_at | 1.804732367 | 9.699702053 | 20.50169027 | 1.70E-29 | 8.35E-28 | 56.76148191 | MTCH1 |
| 227689_at | 1.804702921 | 5.684212908 | 11.74563193 | 1.74E-17 | 1.33E-16 | 28.91828084 | ZNF227 |
| 213735_s_at | -1.804127283 | 10.62992617 | -16.18394591 | 4.55E-24 | 9.02E-23 | 44.18031086 | COX5B |
| 209167_at | 1.803838282 | 6.484330377 | 5.737139052 | 2.99E-07 | 6.64E-07 | 5.358300002 | GPM6B |
| 233546_at | -1.803529121 | 3.723339236 | -14.44206785 | 1.30E-21 | 1.77E-20 | 38.4878563 | LOC283075 |
| 202176_at | 1.802963197 | 6.554613636 | 16.50804625 | 1.66E-24 | 3.55E-23 | 45.19820058 | ERCC3 |
| 219765_at | 1.802785936 | 5.577952756 | 12.34758734 | 1.93E-18 | 1.67E-17 | 31.13399796 | ZNF329 |
| 206718_at | -1.802081731 | 4.979871795 | -14.96710453 | 2.27E-22 | 3.45E-21 | 40.2436579 | LMO1 |
| 203883_s_at | 1.801961042 | 6.929348003 | 11.28767346 | 9.59E-17 | 6.55E-16 | 27.20339704 | RAB11FIP2 |
| 203373_at | 1.801297433 | 6.172252168 | 5.571710033 | 5.66E-07 | 1.22E-06 | 4.729833069 | SOCS2 |
| 225687_at | 1.800032939 | 6.074341015 | 7.001431259 | 2.03E-09 | 5.69E-09 | 10.3086921 | FAM83D |
| 219532_at | 1.798452986 | 4.601282979 | 8.052416431 | 2.98E-11 | 1.04E-10 | 14.5195549 | ELOVL4 |
| 226301_at | 1.798446594 | 6.055826278 | 9.544375104 | 7.83E-14 | 3.74E-13 | 20.46877949 | SLC18B1 |
| 201622_at | 1.79820744 | 7.577952491 | 18.81543311 | 1.76E-27 | 6.13E-26 | 52.09098391 | SND1 |
| 222745_s_at | 1.797301283 | 6.920740886 | 16.67776758 | 9.80E-25 | 2.18E-23 | 45.72620281 | KATNBL1 |
| 210788_s_at | -1.796002952 | 7.547307705 | -6.77860472 | 4.95E-09 | 1.33E-08 | 9.422518209 | DHRS7 |
| 227568_at | 1.795907108 | 6.186768787 | 6.924189043 | 2.76E-09 | 7.63E-09 | 10.00106399 | HECTD2 |
| 228323_at | 1.795641431 | 5.05204511 | 7.816402456 | 7.69E-11 | 2.55E-10 | 13.57204751 | CASC5 |
| 208688_x_at | 1.79419891 | 8.698933594 | 11.91396486 | 9.37E-18 | 7.39E-17 | 29.54234991 | EIF3B |
| 213471_at | 1.793544279 | 5.220758862 | 11.35199461 | 7.54E-17 | 5.22E-16 | 27.44573788 | NPHP4 |
| 212458_at | 1.793400995 | 6.229958857 | 8.412443314 | 7.03E-12 | 2.64E-11 | 15.96367658 | SPRED2 |
| 222127_s_at | 1.793294356 | 7.495319135 | 13.59786344 | 2.30E-20 | 2.61E-19 | 35.59111195 | EXOC1 |
| 200957_s_at | 1.792971425 | 7.69492318 | 14.12454464 | 3.78E-21 | 4.83E-20 | 37.40901223 | SSRP1 |
| 205089_at | 1.792528935 | 6.384872925 | 12.62411869 | 7.11E-19 | 6.53E-18 | 32.1368424 | ZNF7 |
| 218667_at | 1.792463042 | 7.479674886 | 11.93565662 | 8.65E-18 | 6.87E-17 | 29.62251926 | PJA1 |
| 209785_s_at | -1.792427725 | 7.151647383 | -7.762235709 | 9.56E-11 | 3.14E-10 | 13.35460232 | PLA2G4C |
| 212674_s_at | 1.792420571 | 7.423733902 | 16.56082751 | 1.41E-24 | 3.06E-23 | 45.36277181 | DHX30 |
| 202266_at | 1.792043821 | 7.698001844 | 12.28055951 | 2.46E-18 | 2.10E-17 | 30.88948321 | TDP2 |
| 227284_at | 1.791033908 | 6.597422691 | 16.44405975 | 2.02E-24 | 4.26E-23 | 44.99824351 | ZNF766 |
| 238440_at | -1.790443547 | 5.526103187 | -13.02299315 | 1.72E-19 | 1.72E-18 | 33.56639838 | CLYBL |
| 203940_s_at | 1.790160305 | 5.863356876 | 11.74884588 | 1.72E-17 | 1.31E-16 | 28.93022805 | VASH1 |
| 212204_at | 1.790083052 | 7.803225532 | 16.06591273 | 6.59E-24 | 1.27E-22 | 43.80644883 | TMEM87A |
| 227461_at | -1.789706208 | 4.530064182 | -11.29551797 | 9.31E-17 | 6.37E-16 | 27.23297809 | STON2 |
| 222237_s_at | 1.789292373 | 4.954150574 | 10.07575673 | 9.82E-15 | 5.23E-14 | 22.55290546 | ZNF112 |
| 224885_s_at | 1.789145599 | 8.786841364 | 16.20669373 | 4.23E-24 | 8.45E-23 | 44.25216839 | KRTCAP2 |
| 222039_at | 1.788886356 | 6.291954038 | 7.719461596 | 1.14E-10 | 3.69E-10 | 13.18291025 | KIF18B |
| 228190_at | 1.788841951 | 5.851297982 | 11.29858409 | 9.21E-17 | 6.30E-16 | 27.24453831 | ATG4C |
| 209036_s_at | -1.788779954 | 9.884389946 | -14.60829481 | 7.43E-22 | 1.05E-20 | 39.04752396 | MDH2 |
| 212186_at | 1.788164205 | 7.198453599 | 12.06260796 | 5.43E-18 | 4.44E-17 | 30.09056108 | ACACA |
| 202735_at | 1.787983494 | 7.047833276 | 10.86487938 | 4.73E-16 | 2.96E-15 | 25.59877216 | EBP |
| 218660_at | -1.786801668 | 7.391733194 | -10.16992912 | 6.81E-15 | 3.70E-14 | 22.91964419 | DYSF |
| 242260_at | 1.785850004 | 5.667716328 | 7.49264352 | 2.83E-10 | 8.74E-10 | 12.27298816 | MATR3 |
| 214070_s_at | -1.784724709 | 4.736896468 | -12.02989211 | 6.13E-18 | 4.97E-17 | 29.97013336 | ATP10B |
| 228606_at | 1.784505304 | 5.903954777 | 9.395568995 | 1.41E-13 | 6.49E-13 | 19.88103469 | TCTEX1D2 |
| 204629_at | -1.784460611 | 6.04626161 | -10.02480673 | 1.20E-14 | 6.29E-14 | 22.35414527 | PARVB |
| 200037_s_at | 1.784289679 | 9.244692594 | 14.83246171 | 3.53E-22 | 5.24E-21 | 39.79671831 | CBX3 |
| 213761_at | 1.783816078 | 4.697575483 | 12.33023086 | 2.05E-18 | 1.77E-17 | 31.07073583 | MDM1 |
| 202052_s_at | 1.783217629 | 5.311701397 | 6.365415968 | 2.56E-08 | 6.34E-08 | 7.791875386 | RAI14 |
| 214788_x_at | -1.782887409 | 6.404395455 | -11.96723073 | 7.71E-18 | 6.17E-17 | 29.73910994 | DDN |
| 204017_at | 1.781870209 | 6.02432203 | 5.234798895 | 2.04E-06 | 4.14E-06 | 3.469916734 | KDELR3 |
| 212718_at | 1.78105461 | 8.925248122 | 17.24278235 | 1.75E-25 | 4.34E-24 | 47.45937326 | PAPOLA |
| 214239_x_at | 1.780394805 | 7.062092348 | 10.99230112 | 2.92E-16 | 1.87E-15 | 26.08447632 | PCGF2 |
| 200063_s_at | 1.779835814 | 11.89259455 | 20.54104883 | 1.53E-29 | 7.66E-28 | 56.86704488 | NPM1 |
| 223288_at | -1.779721452 | 7.298934257 | -11.41174075 | 6.03E-17 | 4.24E-16 | 27.6704124 | USP38 |
| 200075_s_at | 1.779053611 | 8.093446807 | 17.45797602 | 9.19E-26 | 2.38E-24 | 48.10966398 | GUK1 |
| 227840_at | 1.778836775 | 5.51795223 | 11.24513323 | 1.13E-16 | 7.61E-16 | 27.04285789 | C2orf76 |
| 213477_x_at | 1.778644052 | 12.8550211 | 26.72279104 | 5.61E-36 | 8.22E-34 | 71.75319641 | EEF1A1 |
| 225912_at | 1.77852629 | 7.292131032 | 9.01147462 | 6.44E-13 | 2.75E-12 | 18.35684471 | TP53INP1 |
| 215049_x_at | 1.778192829 | 6.482264839 | 4.398810804 | 4.30E-05 | 7.61E-05 | 0.49160503 | CD163 |
| 235007_at | 1.777865755 | 5.373660505 | 11.75299845 | 1.70E-17 | 1.29E-16 | 28.94566259 | BBS7 |
| 218527_at | 1.777553263 | 6.367411573 | 15.50145432 | 3.98E-23 | 6.88E-22 | 41.99496116 | APTX |
| 209357_at | 1.776455593 | 8.946916495 | 5.277570157 | 1.73E-06 | 3.55E-06 | 3.628242743 | CITED2 |
| 212643_at | 1.776109808 | 9.533411461 | 13.82164188 | 1.06E-20 | 1.27E-19 | 36.36784436 | MAPK1IP1L |
| 213546_at | 1.776004477 | 7.388378979 | 10.23187175 | 5.36E-15 | 2.95E-14 | 23.16041161 | DKFZP586I1420 |
| 202460_s_at | 1.775639652 | 7.256214188 | 10.23351714 | 5.33E-15 | 2.93E-14 | 23.16680213 | LPIN2 |
| 221063_x_at | -1.775603355 | 7.038530866 | -20.03513876 | 5.97E-29 | 2.61E-27 | 55.49842307 | RNF123 |
| 212513_s_at | 1.775217549 | 8.243396703 | 13.2324169 | 8.22E-20 | 8.64E-19 | 34.30888067 | USP33 |
| 228946_at | 1.774975904 | 4.592494854 | 7.575978527 | 2.02E-10 | 6.36E-10 | 12.60717584 | INTU |
| 232671_at | -1.774890106 | 3.975317632 | -22.7874148 | 4.87E-32 | 3.60E-30 | 62.64931944 | SLFNL1-AS1 |
| 212685_s_at | 1.774885304 | 7.547655753 | 15.38596112 | 5.78E-23 | 9.74E-22 | 41.61946079 | TBL2 |
| 214096_s_at | 1.774287935 | 8.739636035 | 11.70230998 | 2.05E-17 | 1.54E-16 | 28.7571192 | SHMT2 |
| 211936_at | 1.774051966 | 9.623814503 | 10.47770088 | 2.08E-15 | 1.20E-14 | 24.11223555 | HSPA5 |
| 200687_s_at | 1.772942794 | 7.577223074 | 16.11907164 | 5.58E-24 | 1.09E-22 | 43.97503584 | SF3B3 |
| 226661_at | 1.772834882 | 5.934893299 | 7.287393891 | 6.45E-10 | 1.91E-09 | 11.45084279 | CDCA2 |
| 213041_s_at | -1.772753643 | 8.932209275 | -15.28909587 | 7.91E-23 | 1.30E-21 | 41.30324358 | ATP5D |
| 213271_s_at | 1.772512615 | 6.767703988 | 12.3668219 | 1.80E-18 | 1.56E-17 | 31.20406166 | DOPEY1 |
| 200632_s_at | 1.772361877 | 8.144741499 | 6.594122802 | 1.03E-08 | 2.67E-08 | 8.692158465 | NDRG1 |
| 202466_at | 1.772031173 | 7.498123298 | 15.05898108 | 1.68E-22 | 2.60E-21 | 40.54732584 | PAPD7 |
| 226518_at | 1.771816237 | 7.489296075 | 17.33040001 | 1.35E-25 | 3.40E-24 | 47.7247908 | KCTD10 |
| 205824_at | -1.771037699 | 7.554703371 | -7.529298892 | 2.44E-10 | 7.60E-10 | 12.41996053 | HSPB2 |
| 222997_s_at | 1.770265489 | 9.290887527 | 15.34329388 | 6.64E-23 | 1.10E-21 | 41.4803176 | MRPS21 |
| 224450_s_at | 1.770185972 | 6.534355309 | 13.02707951 | 1.69E-19 | 1.69E-18 | 33.5809394 | RIOK1 |
| 209022_at | 1.769912903 | 8.780443533 | 8.544990462 | 4.13E-12 | 1.59E-11 | 16.49450689 | STAG2 |
| 200678_x_at | 1.768737114 | 8.122788843 | 12.10119726 | 4.72E-18 | 3.89E-17 | 30.23244032 | GRN |
| 218917_s_at | 1.768168585 | 8.182184707 | 15.4376229 | 4.89E-23 | 8.32E-22 | 41.7876327 | ARID1A |
| 207508_at | -1.76801834 | 10.14892856 | -13.85276047 | 9.56E-21 | 1.15E-19 | 36.47534914 | ATP5G3 |
| 203306_s_at | 1.76800647 | 7.828439928 | 8.443264219 | 6.21E-12 | 2.35E-11 | 16.0871606 | SLC35A1 |
| 223590_at | 1.767535578 | 5.206529677 | 11.98917245 | 7.11E-18 | 5.72E-17 | 29.82006045 | ZNF700 |
| 202910_s_at | 1.767392273 | 6.822329326 | 11.48387617 | 4.60E-17 | 3.30E-16 | 27.9411221 | CD97 |
| 219595_at | 1.76682851 | 6.508386844 | 13.11077447 | 1.26E-19 | 1.29E-18 | 33.8782947 | ZNF26 |
| 212058_at | 1.76640374 | 8.028993909 | 14.34601605 | 1.79E-21 | 2.39E-20 | 38.16285839 | U2SURP |
| 222550_at | 1.76638236 | 8.810523325 | 14.58323374 | 8.08E-22 | 1.14E-20 | 38.96337085 | ARMC1 |
| 227471_at | 1.766108766 | 5.416999776 | 14.25221091 | 2.45E-21 | 3.20E-20 | 37.84432763 | HACE1 |
| 216952_s_at | 1.76548811 | 6.49188885 | 11.42839975 | 5.66E-17 | 4.00E-16 | 27.73298428 | LMNB2 |
| 202110_at | -1.7653103 | 10.75653833 | -14.84962197 | 3.34E-22 | 4.98E-21 | 39.85380809 | COX7B |
| 200897_s_at | -1.763947492 | 8.65242543 | -4.204921694 | 8.45E-05 | 0.000144758 | -0.161817681 | PALLD |
| 200809_x_at | 1.763551533 | 12.56461777 | 18.14095413 | 1.23E-26 | 3.67E-25 | 50.13847599 | RPL12 |
| 212943_at | 1.76238552 | 7.903060169 | 11.3846142 | 6.67E-17 | 4.67E-16 | 27.5684548 | C2CD5 |
| 231726_at | 1.76227782 | 4.376157783 | 7.650038124 | 1.50E-10 | 4.80E-10 | 12.90429998 | PCDHB14 |
| 212919_at | 1.76223325 | 7.916785321 | 10.71425026 | 8.40E-16 | 5.08E-15 | 25.02232934 | DCP2 |
| 201896_s_at | 1.761898959 | 5.707583985 | 8.03202679 | 3.23E-11 | 1.12E-10 | 14.43770695 | PSRC1 |
| 218961_s_at | 1.761492118 | 6.303774231 | 15.35454197 | 6.40E-23 | 1.07E-21 | 41.51702106 | PNKP |
| 202291_s_at | 1.761297276 | 9.206109882 | 5.013892041 | 4.65E-06 | 9.12E-06 | 2.660483076 | MGP |
| 219649_at | 1.76120167 | 7.037105263 | 8.541834336 | 4.19E-12 | 1.61E-11 | 16.48187411 | ALG6 |
| 213483_at | 1.761116451 | 6.491953335 | 12.38066751 | 1.71E-18 | 1.49E-17 | 31.25446705 | PPWD1 |
| 224794_s_at | 1.761026288 | 6.668731522 | 7.191925632 | 9.46E-10 | 2.75E-09 | 11.06903558 | CERCAM |
| 226165_at | 1.760525895 | 9.033875114 | 15.08209359 | 1.55E-22 | 2.42E-21 | 40.62354954 | C8orf59 |
| 213005_s_at | -1.760187726 | 6.619081905 | -7.51708829 | 2.56E-10 | 7.96E-10 | 12.37099719 | KANK1 |
| 226604_at | 1.760135502 | 6.024145038 | 10.99404258 | 2.90E-16 | 1.86E-15 | 26.09110198 | TMTC3 |
| 230200_at | 1.760115316 | 6.72427053 | 8.215102737 | 1.55E-11 | 5.59E-11 | 15.1724277 | NSUN6 |
| 202228_s_at | 1.759853694 | 9.485074588 | 10.09664316 | 9.05E-15 | 4.83E-14 | 22.63431581 | NPTN |
| 222106_at | 1.759590094 | 5.60906678 | 3.715407664 | 0.000434172 | 0.000691069 | -1.73448926 | PRND |
| 217773_s_at | -1.759338689 | 10.93012422 | -13.16793732 | 1.03E-19 | 1.07E-18 | 34.08087287 | NDUFA4 |
| 224729_s_at | -1.759228961 | 7.787137466 | -15.2206304 | 9.89E-23 | 1.60E-21 | 41.0790306 | ATPAF1 |
| 202675_at | -1.758895236 | 8.180411953 | -13.10761444 | 1.27E-19 | 1.30E-18 | 33.86708381 | SDHB |
| 223001_at | 1.758706327 | 7.999754677 | 10.29440441 | 4.21E-15 | 2.35E-14 | 23.40309718 | OSTC |
| 237003_at | -1.75869208 | 4.468662644 | -13.73153381 | 1.45E-20 | 1.69E-19 | 36.05585094 | BEST3 |
| 219611_s_at | -1.758655013 | 6.726067486 | -22.81326076 | 4.57E-32 | 3.39E-30 | 62.71318942 | CEP85 |
| 202905_x_at | 1.758365259 | 7.638303314 | 10.45870985 | 2.24E-15 | 1.29E-14 | 24.03891952 | NBN |
| 235767_x_at | 1.75809235 | 6.641937817 | 8.526845686 | 4.45E-12 | 1.71E-11 | 16.4218753 | PHAX |
| 212584_at | 1.757391884 | 6.717408398 | 16.14416647 | 5.15E-24 | 1.01E-22 | 44.05450151 | AQR |
| 209140_x_at | 1.757120664 | 10.62555825 | 7.446274405 | 3.41E-10 | 1.04E-09 | 12.08712344 | HLA-B |
| 203710_at | 1.756848096 | 4.855720719 | 8.265682832 | 1.27E-11 | 4.61E-11 | 15.37531913 | ITPR1 |
| 235977_at | -1.756620603 | 4.277745389 | -10.73791846 | 7.67E-16 | 4.67E-15 | 25.11306645 | LONRF2 |
| 227305_s_at | 1.755949855 | 7.059722345 | 19.792471 | 1.16E-28 | 4.84E-27 | 54.83279893 | SMCR8 |
| 203911_at | 1.7556464 | 6.837328156 | 7.113270591 | 1.30E-09 | 3.71E-09 | 10.75482583 | RAP1GAP |
| 223730_at | 1.755546477 | 6.511518464 | 7.861029188 | 6.43E-11 | 2.15E-10 | 13.75120866 | GPC6 |
| 212737_at | 1.755218696 | 7.291840208 | 10.86735333 | 4.69E-16 | 2.93E-15 | 25.60821925 | GM2A |
| 223042_s_at | -1.755164192 | 9.419519005 | -16.70086229 | 9.13E-25 | 2.05E-23 | 45.79778462 | FUNDC2 |
| 239237_at | 1.754792426 | 5.835665347 | 3.980763301 | 0.000181045 | 0.000300263 | -0.896411697 | TRG-AS1 |
| 203257_s_at | 1.754616719 | 7.005230181 | 20.97397235 | 4.88E-30 | 2.67E-28 | 58.01819431 | C11orf49 |
| 203323_at | 1.754152845 | 8.012614656 | 4.473175418 | 3.31E-05 | 5.93E-05 | 0.746370709 | CAV2 |
| 231257_at | 1.753913866 | 4.745494324 | 4.630634232 | 1.89E-05 | 3.47E-05 | 1.292962244 | TCERG1L |
| 219083_at | 1.753320537 | 7.065464076 | 16.59899989 | 1.25E-24 | 2.73E-23 | 45.48158499 | SHQ1 |
| 212953_x_at | 1.75267521 | 8.870189821 | 18.73268841 | 2.23E-27 | 7.56E-26 | 51.85412521 | CALR |
| 229275_at | -1.752510375 | 5.181775112 | -6.637859764 | 8.67E-09 | 2.27E-08 | 8.865004217 | IGFN1 |
| 217734_s_at | 1.752365475 | 7.77226638 | 13.28643094 | 6.80E-20 | 7.23E-19 | 34.49947274 | WDR6 |
| 203291_at | 1.752115887 | 6.400138591 | 16.51480143 | 1.62E-24 | 3.49E-23 | 45.21928177 | CNOT4 |
| 225219_at | 1.751979695 | 7.807191918 | 15.11592868 | 1.39E-22 | 2.18E-21 | 40.73501471 | SMAD5 |
| 221530_s_at | -1.751546909 | 6.962067333 | -4.37008066 | 4.76E-05 | 8.39E-05 | 0.393780697 | BHLHE41 |
| 223295_s_at | 1.751248719 | 7.682893011 | 12.01912509 | 6.37E-18 | 5.16E-17 | 29.93047105 | LUC7L |
| 213396_s_at | 1.750956282 | 6.600378459 | 12.30331546 | 2.26E-18 | 1.94E-17 | 30.97255856 | AKAP10 |
| 205449_at | 1.750857206 | 6.513780815 | 11.56022125 | 3.47E-17 | 2.53E-16 | 28.22696454 | SAC3D1 |
| 225376_at | 1.750806621 | 7.216942418 | 14.89039851 | 2.92E-22 | 4.38E-21 | 39.98931675 | GID8 |
| 217946_s_at | 1.750284391 | 7.120081192 | 11.44280622 | 5.37E-17 | 3.81E-16 | 27.78706946 | SAE1 |
| 228745_at | 1.750097341 | 5.805824034 | 9.843062872 | 2.43E-14 | 1.23E-13 | 21.64323981 | SGTB |
| 208979_at | 1.74990812 | 7.339735948 | 18.00096156 | 1.84E-26 | 5.38E-25 | 49.72691752 | NCOA6 |
| 218656_s_at | 1.749884119 | 8.167166486 | 6.32856058 | 2.96E-08 | 7.27E-08 | 7.647408566 | LHFP |
| 213434_at | 1.749636536 | 6.993644377 | 14.66458639 | 6.16E-22 | 8.82E-21 | 39.23625618 | STX2 |
| 229019_at | -1.749270476 | 4.309839928 | -9.285963854 | 2.17E-13 | 9.80E-13 | 19.44709149 | ZNF385B |
| 220301_at | 1.748832921 | 4.416876091 | 5.518808859 | 6.93E-07 | 1.48E-06 | 4.530151625 | CCDC102B |
| 208829_at | 1.748612554 | 7.842168886 | 11.93168979 | 8.78E-18 | 6.96E-17 | 29.60786278 | TAPBP |
| 226930_at | 1.74822682 | 5.324288451 | 4.160693711 | 9.84E-05 | 0.000167469 | -0.308583401 | FNDC1 |
| 202786_at | 1.747908132 | 6.012370348 | 6.735975029 | 5.86E-09 | 1.56E-08 | 9.253455513 | STK39 |
| 230068_s_at | 1.747442441 | 5.70834474 | 4.564974879 | 2.39E-05 | 4.34E-05 | 1.063883793 | PEG3-AS1 |
| 206773_at | 1.747321444 | 4.770170016 | 4.83830489 | 8.88E-06 | 1.69E-05 | 2.027792124 | LY6H |
| 213535_s_at | 1.747318152 | 8.563319083 | 18.01641136 | 1.76E-26 | 5.16E-25 | 49.77244551 | UBE2I |
| 225386_s_at | 1.747111929 | 7.10986428 | 10.24281463 | 5.14E-15 | 2.84E-14 | 23.20290765 | HNRNPLL |
| 205954_at | -1.746889843 | 4.717616329 | -10.99555578 | 2.88E-16 | 1.85E-15 | 26.09685895 | RXRG |
| 223139_s_at | 1.746138108 | 8.885822613 | 18.4783141 | 4.62E-27 | 1.48E-25 | 51.12131591 | DHX36 |
| 213168_at | 1.745795095 | 7.933108538 | 13.54098463 | 2.80E-20 | 3.15E-19 | 35.39266496 | SP3 |
| 225880_at | 1.744310074 | 6.826398691 | 11.86137935 | 1.14E-17 | 8.87E-17 | 29.3477649 | TOR1AIP2 |
| 202686_s_at | 1.743390377 | 7.022825078 | 6.512084867 | 1.43E-08 | 3.65E-08 | 8.368511735 | AXL |
| 225172_at | 1.742457152 | 6.642753942 | 18.76636853 | 2.03E-27 | 6.95E-26 | 51.95062466 | CRAMP1L |
| 201221_s_at | 1.74197778 | 8.422863773 | 11.26910758 | 1.03E-16 | 6.98E-16 | 27.13335832 | SNRNP70 |
| 225626_at | 1.741556156 | 6.116920746 | 8.283036099 | 1.18E-11 | 4.31E-11 | 15.44491563 | PAG1 |
| 202126_at | 1.740595796 | 7.734630459 | 14.25251461 | 2.45E-21 | 3.20E-20 | 37.84536068 | PRPF4B |
| 208775_at | 1.740484589 | 9.497075558 | 15.1988285 | 1.06E-22 | 1.70E-21 | 41.00750989 | XPO1 |
| 226303_at | -1.740443482 | 4.964645766 | -12.25641288 | 2.68E-18 | 2.28E-17 | 30.80126061 | PGM5 |
| 218659_at | 1.740207747 | 7.94297039 | 13.96390697 | 6.53E-21 | 8.09E-20 | 36.85831335 | ASXL2 |
| 209959_at | -1.739842257 | 5.963668334 | -6.351164052 | 2.70E-08 | 6.69E-08 | 7.7359888 | NR4A3 |
| 218331_s_at | 1.739482747 | 7.025432804 | 13.09227983 | 1.35E-19 | 1.37E-18 | 33.81266286 | FAM208B |
| 221958_s_at | 1.739336035 | 6.301793008 | 5.2709898 | 1.78E-06 | 3.64E-06 | 3.603851885 | WLS |
| 204072_s_at | -1.739221419 | 5.788461718 | -7.188814254 | 9.58E-10 | 2.78E-09 | 11.05659987 | FRY |
| 201185_at | 1.739219136 | 7.956990382 | 5.153231766 | 2.77E-06 | 5.55E-06 | 3.169389742 | HTRA1 |
| 213502_x_at | 1.739138281 | 7.123494598 | 10.52070802 | 1.76E-15 | 1.03E-14 | 24.27813126 | GUSBP11 |
| 201596_x_at | 1.739118788 | 6.28119468 | 5.758341982 | 2.76E-07 | 6.13E-07 | 5.439271453 | KRT18 |
| 226965_at | 1.738436671 | 7.171975945 | 13.7045524 | 1.59E-20 | 1.84E-19 | 35.9622274 | DENND6A |
| 221825_at | 1.738168528 | 7.488576441 | 11.67696565 | 2.25E-17 | 1.68E-16 | 28.6627318 | ANGEL2 |
| 218269_at | 1.738069212 | 7.547386925 | 16.05261388 | 6.87E-24 | 1.32E-22 | 43.76421932 | DROSHA |
| 200022_at | 1.737989329 | 11.96219508 | 18.67785177 | 2.61E-27 | 8.70E-26 | 51.69674569 | RPL18 |
| 216032_s_at | 1.737335857 | 8.546323376 | 14.22732389 | 2.67E-21 | 3.45E-20 | 37.75963128 | ERGIC3 |
| 229310_at | 1.736670237 | 6.747670312 | 6.701639501 | 6.72E-09 | 1.78E-08 | 9.11740945 | KLHL29 |
| 222464_s_at | 1.735907233 | 7.337396502 | 13.25998059 | 7.46E-20 | 7.90E-19 | 34.40618747 | MCMBP |
| 201328_at | 1.735273324 | 7.594115332 | 6.621103341 | 9.27E-09 | 2.41E-08 | 8.798760085 | ETS2 |
| 212836_at | 1.73499442 | 6.724572777 | 15.95739861 | 9.29E-24 | 1.75E-22 | 43.46123936 | POLD3 |
| 223805_at | -1.734785085 | 5.939923067 | -6.908713315 | 2.94E-09 | 8.10E-09 | 9.93948277 | OSBPL6 |
| 203060_s_at | 1.734629121 | 6.526486573 | 4.941906895 | 6.07E-06 | 1.18E-05 | 2.399899323 | PAPSS2 |
| 209166_s_at | 1.734587198 | 6.556791124 | 11.68683059 | 2.17E-17 | 1.63E-16 | 28.69947995 | MAN2B1 |
| 1554154_at | 1.734548575 | 6.336805394 | 11.70100417 | 2.06E-17 | 1.55E-16 | 28.75225798 | GDAP2 |
| 213309_at | -1.734363713 | 6.200165948 | -9.167797957 | 3.46E-13 | 1.53E-12 | 18.97833436 | PLCL2 |
| 217819_at | 1.734103662 | 8.554582138 | 16.46411087 | 1.90E-24 | 4.02E-23 | 45.06095593 | GOLGA7 |
| 225074_at | 1.733754369 | 6.647434846 | 13.48563924 | 3.39E-20 | 3.77E-19 | 35.19917068 | RAB2B |
| 216221_s_at | 1.733587885 | 8.573392786 | 15.56601853 | 3.23E-23 | 5.64E-22 | 42.20415495 | PUM2 |
| 209268_at | 1.732577301 | 7.201373068 | 13.78999962 | 1.19E-20 | 1.40E-19 | 36.25840344 | VPS45 |
| 205250_s_at | 1.73255582 | 6.008506101 | 12.57527493 | 8.47E-19 | 7.71E-18 | 31.96040688 | CEP290 |
| 218017_s_at | 1.7324387 | 7.574259183 | 11.77671659 | 1.55E-17 | 1.19E-16 | 29.03377987 | HGSNAT |
| 204719_at | -1.731963606 | 5.508617658 | -3.331041485 | 0.001453642 | 0.002183437 | -2.8811834 | ABCA8 |
| 203569_s_at | 1.731573226 | 6.902707465 | 12.18284343 | 3.50E-18 | 2.94E-17 | 30.53202067 | OFD1 |
| 212788_x_at | 1.731476878 | 12.26652422 | 14.32512664 | 1.92E-21 | 2.55E-20 | 38.09202205 | FTL |
| 227179_at | -1.730546646 | 6.161979418 | -7.385156448 | 4.35E-10 | 1.31E-09 | 11.84224675 | STAU2 |
| 207181_s_at | 1.73009353 | 6.752028777 | 9.9462115 | 1.62E-14 | 8.40E-14 | 22.04707485 | CASP7 |
| 227891_s_at | 1.729609925 | 7.386335642 | 11.33152736 | 8.14E-17 | 5.61E-16 | 27.36867586 | TAF15 |
| 201303_at | 1.729428804 | 8.158807089 | 14.3091499 | 2.03E-21 | 2.68E-20 | 38.03780713 | EIF4A3 |
| 211754_s_at | 1.729420396 | 5.86206651 | 12.61178718 | 7.43E-19 | 6.80E-18 | 32.09232629 | SLC25A17 |
| 202230_s_at | 1.728941258 | 7.973024362 | 13.88342298 | 8.60E-21 | 1.04E-19 | 36.5811571 | CHERP |
| 236700_at | 1.728749469 | 4.607944765 | 10.79664356 | 6.13E-16 | 3.77E-15 | 25.33794302 | EIF3C |
| 201170_s_at | -1.728504324 | 7.637324848 | -5.819627742 | 2.17E-07 | 4.89E-07 | 5.673825399 | BHLHE40 |
| 1570064_at | -1.72799676 | 4.31989321 | -13.49125943 | 3.33E-20 | 3.71E-19 | 35.21883742 | RP11-440I14.2 |
| 201212_at | 1.727939416 | 7.796441818 | 7.098591835 | 1.38E-09 | 3.92E-09 | 10.69622665 | LGMN |
| 220933_s_at | 1.727782404 | 7.133796 | 12.62611971 | 7.06E-19 | 6.49E-18 | 32.14406417 | ZCCHC6 |
| 223824_at | -1.726900387 | 4.577248199 | -14.36352042 | 1.69E-21 | 2.27E-20 | 38.22217319 | RNLS |
| 218347_at | 1.726219114 | 7.369830122 | 9.157358119 | 3.61E-13 | 1.59E-12 | 18.93687619 | TYW1 |
| 212242_at | -1.725755147 | 7.813184307 | -7.412916761 | 3.89E-10 | 1.18E-09 | 11.95345589 | TUBA4A |
| 203868_s_at | 1.72553167 | 4.868393649 | 5.846575023 | 1.96E-07 | 4.43E-07 | 5.777193273 | VCAM1 |
| 228636_at | 1.724465645 | 6.124359785 | 4.198112406 | 8.65E-05 | 0.00014808 | -0.184470364 | BHLHE22 |
| 208216_at | 1.724380671 | 5.663367159 | 4.214453619 | 8.18E-05 | 0.000140335 | -0.130073028 | DLX4 |
| 226525_at | 1.723848699 | 6.45819867 | 7.285434905 | 6.50E-10 | 1.92E-09 | 11.44300386 | STK17B |
| 208692_at | 1.723750612 | 12.0348541 | 15.82682753 | 1.41E-23 | 2.57E-22 | 43.04395061 | RPS3 |
| 1561391_at | -1.723536705 | 4.441802295 | -20.57397103 | 1.40E-29 | 7.08E-28 | 56.95522788 | STAU2-AS1 |
| 212265_at | 1.723266317 | 8.923274096 | 8.63871151 | 2.84E-12 | 1.12E-11 | 16.86946355 | QKI |
| 203632_s_at | -1.723148595 | 6.075036995 | -8.375839446 | 8.14E-12 | 3.03E-11 | 15.81698647 | GPRC5B |
| 218229_s_at | 1.722964302 | 6.966900012 | 11.96939817 | 7.65E-18 | 6.12E-17 | 29.747109 | POGK |
| 239106_at | 1.722562617 | 5.574841859 | 10.08388311 | 9.51E-15 | 5.07E-14 | 22.58458495 | CA5BP1 |
| 206583_at | 1.722083339 | 5.544195703 | 14.95121106 | 2.39E-22 | 3.62E-21 | 40.19101939 | KRBOX4 |
| 225317_at | 1.722034364 | 6.997889797 | 15.41626892 | 5.24E-23 | 8.86E-22 | 41.71816049 | ACBD6 |
| 214761_at | -1.721696396 | 6.895376754 | -6.297537512 | 3.34E-08 | 8.17E-08 | 7.525946121 | ZNF423 |
| 225314_at | 1.721674253 | 7.304109323 | 9.641582224 | 5.35E-14 | 2.60E-13 | 20.85180301 | OCIAD2 |
| 218670_at | 1.721001393 | 6.227618091 | 12.03522218 | 6.01E-18 | 4.88E-17 | 29.98976236 | PUS1 |
| 225989_at | 1.720797881 | 7.040423324 | 7.301419931 | 6.09E-10 | 1.81E-09 | 11.50697324 | HERC4 |
| 214453_s_at | 1.720581903 | 6.07957069 | 5.42904677 | 9.76E-07 | 2.05E-06 | 4.19285478 | IFI44 |
| 228144_at | 1.720471566 | 6.498636991 | 8.385932468 | 7.81E-12 | 2.92E-11 | 15.85743812 | ZNF300 |
| 218220_at | 1.72042392 | 6.952795141 | 11.77034509 | 1.59E-17 | 1.22E-16 | 29.01011521 | C12orf10 |
| 203781_at | -1.72008762 | 8.472814206 | -18.7086549 | 2.39E-27 | 8.07E-26 | 51.78518987 | MRPL33 |
| 217957_at | 1.719780671 | 7.382059229 | 11.95512766 | 8.06E-18 | 6.43E-17 | 29.69443247 | C16orf80 |
| 211570_s_at | -1.719359656 | 3.909078448 | -19.75391852 | 1.29E-28 | 5.33E-27 | 54.72649818 | RAPSN |
| 226826_at | 1.719206287 | 5.903485423 | 8.433367523 | 6.46E-12 | 2.44E-11 | 16.04751264 | LSM11 |
| 229893_at | -1.718385845 | 5.685572166 | -10.42443153 | 2.55E-15 | 1.46E-14 | 23.90649354 | FRMD3 |
| 225170_at | 1.717618507 | 6.368392852 | 12.69447789 | 5.53E-19 | 5.16E-18 | 32.39046909 | WDR5 |
| 227413_at | 1.717534461 | 6.463462455 | 12.72004466 | 5.04E-19 | 4.72E-18 | 32.48247608 | UBLCP1 |
| 202442_at | 1.717530681 | 8.63309792 | 13.96145209 | 6.59E-21 | 8.16E-20 | 36.84987193 | AP3S1 |
| 201954_at | 1.716519463 | 7.36463524 | 8.101883326 | 2.44E-11 | 8.59E-11 | 14.7181074 | ARPC1B |
| 1562411_at | -1.715783496 | 4.807904175 | -22.23173391 | 1.94E-31 | 1.30E-29 | 61.26196175 | MYLK3 |
| 224872_at | 1.715452339 | 5.911872168 | 11.70904588 | 2.00E-17 | 1.50E-16 | 28.78219212 | DIP2B |
| 206414_s_at | 1.71530158 | 5.850956334 | 6.793534137 | 4.66E-09 | 1.26E-08 | 9.481764756 | ASAP2 |
| 201324_at | 1.715269736 | 7.402079898 | 4.428747905 | 3.87E-05 | 6.89E-05 | 0.59389831 | EMP1 |
| 214430_at | 1.715256751 | 7.466042767 | 18.12411349 | 1.29E-26 | 3.84E-25 | 50.08908281 | GLA |
| 218005_at | 1.714880204 | 7.677021526 | 14.95232934 | 2.38E-22 | 3.61E-21 | 40.19472413 | ZNF22 |
| 201960_s_at | 1.713088173 | 8.437168306 | 16.69094937 | 9.41E-25 | 2.10E-23 | 45.76706739 | MYCBP2 |
| 227916_x_at | 1.711331465 | 6.156563966 | 12.25797606 | 2.67E-18 | 2.27E-17 | 30.80697404 | EXOSC3 |
| 200686_s_at | 1.711079831 | 10.14047863 | 13.07164967 | 1.45E-19 | 1.46E-18 | 33.73940128 | SRSF11 |
| 227586_at | 1.710995423 | 5.925331215 | 10.08697652 | 9.40E-15 | 5.01E-14 | 22.59664259 | TMEM170A |
| 218364_at | -1.710405702 | 6.671502614 | -15.15767508 | 1.21E-22 | 1.93E-21 | 40.8723446 | LRRFIP2 |
| 218103_at | 1.709941481 | 7.133765219 | 14.58981627 | 7.90E-22 | 1.11E-20 | 38.98548219 | FTSJ3 |
| 1553709_a_at | 1.709507592 | 6.828235687 | 12.61452781 | 7.36E-19 | 6.74E-18 | 32.10222149 | PRPF38A |
| 203663_s_at | -1.708929207 | 10.5206789 | -14.91284746 | 2.71E-22 | 4.09E-21 | 40.0638296 | COX5A |
| 206854_s_at | 1.708873995 | 6.623456875 | 13.00180211 | 1.85E-19 | 1.85E-18 | 33.49095749 | MAP3K7 |
| 238727_at | 1.708470855 | 4.795662764 | 4.410923945 | 4.12E-05 | 7.31E-05 | 0.532950985 | LOC440934 |
| 203815_at | -1.708112174 | 6.039213892 | -5.879721358 | 1.72E-07 | 3.92E-07 | 5.904531845 | GSTT1 |
| 219069_at | 1.707825538 | 6.69576811 | 13.25256507 | 7.66E-20 | 8.10E-19 | 34.38001835 | ANKRD49 |
| 200945_s_at | 1.707570944 | 9.221426751 | 14.26333181 | 2.36E-21 | 3.10E-20 | 37.88214909 | SEC31A |
| 201989_s_at | 1.706732988 | 7.492107618 | 12.70006406 | 5.42E-19 | 5.06E-18 | 32.41057905 | CREBL2 |
| 210395_x_at | -1.706346039 | 5.249766079 | -17.2369964 | 1.78E-25 | 4.41E-24 | 47.44181466 | MYL4 |
| 219644_at | 1.706255264 | 5.329027621 | 12.12909585 | 4.26E-18 | 3.53E-17 | 30.33489963 | CEP83 |
| 224691_at | 1.706221661 | 9.129591135 | 8.855830275 | 1.20E-12 | 4.92E-12 | 17.73664995 | UHMK1 |
| 213282_at | -1.706211066 | 7.015145237 | -10.20755972 | 5.89E-15 | 3.22E-14 | 23.06595595 | APOOL |
| 224492_s_at | 1.706025886 | 6.754589225 | 10.76590968 | 6.90E-16 | 4.22E-15 | 25.22029958 | ZNF627 |
| 224564_s_at | 1.705983288 | 8.017979752 | 13.36723196 | 5.13E-20 | 5.58E-19 | 34.783889 | RTN3 |
| 201506_at | 1.705856742 | 8.197515085 | 4.957599054 | 5.73E-06 | 1.11E-05 | 2.456564932 | TGFBI |
| 224308_s_at | 1.705305518 | 5.206338889 | 11.85721585 | 1.15E-17 | 8.99E-17 | 29.33234413 | INTS2 |
| 226345_at | 1.705038544 | 6.905890207 | 11.94479961 | 8.37E-18 | 6.65E-17 | 29.65629318 | ARL5B |
| 1556797_at | -1.704756475 | 5.261271648 | -15.22851329 | 9.64E-23 | 1.56E-21 | 41.10487563 | RNF144A-AS1 |
| 208993_s_at | 1.704222546 | 8.136860487 | 12.84651376 | 3.21E-19 | 3.09E-18 | 32.9363851 | PPIG |
| 205359_at | -1.703649939 | 4.746134371 | -17.04508538 | 3.19E-25 | 7.62E-24 | 46.85721097 | AKAP6 |
| 235609_at | 1.70280503 | 5.356275349 | 8.093159265 | 2.53E-11 | 8.88E-11 | 14.68309234 | BRIP1 |
| 203459_s_at | 1.702036781 | 6.961367961 | 16.37011102 | 2.54E-24 | 5.25E-23 | 44.76654144 | VPS16 |
| 225906_at | -1.700357449 | 7.713576388 | -16.07037104 | 6.50E-24 | 1.25E-22 | 43.82060106 | GATSL2 |
| 228785_at | 1.699417226 | 7.479732423 | 10.21730238 | 5.67E-15 | 3.11E-14 | 23.10381437 | ZNF281 |
| 209687_at | -1.698654293 | 6.870025452 | -4.073445104 | 0.000132436 | 0.000222594 | -0.59550876 | CXCL12 |
| 205527_s_at | 1.698372593 | 6.433373029 | 12.9409171 | 2.30E-19 | 2.26E-18 | 33.27388705 | GEMIN4 |
| 203858_s_at | -1.698149001 | 6.335484895 | -19.80577926 | 1.11E-28 | 4.69E-27 | 54.86945842 | COX10 |
| 210705_s_at | 1.69747042 | 3.894498961 | 15.05647651 | 1.69E-22 | 2.62E-21 | 40.53906187 | TRIM5 |
| 200826_at | 1.697462263 | 10.43083328 | 14.77134721 | 4.32E-22 | 6.35E-21 | 39.59309662 | SNRPD2 |
| 226434_at | 1.69734729 | 6.624043757 | 14.76837252 | 4.37E-22 | 6.40E-21 | 39.58317347 | PPP1R35 |
| 209076_s_at | 1.697288643 | 7.877991079 | 19.30004114 | 4.51E-28 | 1.71E-26 | 53.46346324 | WDR45B |
| 212893_at | 1.696981501 | 6.902567847 | 11.66122063 | 2.38E-17 | 1.77E-16 | 28.60405556 | ZZZ3 |
| 215136_s_at | 1.696708409 | 6.83622557 | 11.73612785 | 1.81E-17 | 1.37E-16 | 28.88294406 | EXOSC8 |
| 200634_at | 1.696630855 | 9.441749895 | 14.88350985 | 2.98E-22 | 4.47E-21 | 39.96643911 | PFN1 |
| 227370_at | 1.696465083 | 4.857601177 | 6.397526869 | 2.25E-08 | 5.62E-08 | 7.917890638 | FAM171B |
| 217892_s_at | 1.695720405 | 8.159880207 | 11.43475182 | 5.53E-17 | 3.92E-16 | 27.75683438 | LIMA1 |
| 221827_at | 1.695454792 | 7.168232096 | 13.28141531 | 6.92E-20 | 7.36E-19 | 34.48179045 | RBCK1 |
| 229982_at | 1.695251087 | 8.116787659 | 7.662341077 | 1.43E-10 | 4.58E-10 | 12.95366883 | QSER1 |
| 204033_at | 1.695232897 | 5.851446897 | 8.208507685 | 1.59E-11 | 5.72E-11 | 15.1459693 | TRIP13 |
| 203301_s_at | 1.695141786 | 7.884539224 | 9.535820633 | 8.10E-14 | 3.86E-13 | 20.435037 | DMTF1 |
| 229428_at | -1.69478118 | 6.584846897 | -10.354392 | 3.34E-15 | 1.89E-14 | 23.63554595 | TIMM23B |
| 212034_s_at | 1.694212454 | 7.524024292 | 20.29682861 | 2.94E-29 | 1.36E-27 | 56.20955151 | EXOC7 |
| 218827_s_at | 1.693103178 | 5.672134166 | 11.68129553 | 2.21E-17 | 1.66E-16 | 28.6788626 | CEP192 |
| 204542_at | -1.692550306 | 6.017051723 | -8.010207733 | 3.53E-11 | 1.22E-10 | 14.35011735 | ST6GALNAC2 |
| 218346_s_at | -1.692127955 | 7.81257533 | -8.12764338 | 2.20E-11 | 7.77E-11 | 14.82149282 | SESN1 |
| 227611_at | -1.691405274 | 6.574320795 | -8.878624611 | 1.09E-12 | 4.52E-12 | 17.82755985 | TARSL2 |
| 226566_at | 1.690865254 | 5.856529268 | 18.14509532 | 1.21E-26 | 3.63E-25 | 50.15061716 | TRIM11 |
| 221260_s_at | 1.690447903 | 6.360711373 | 11.41308211 | 6.00E-17 | 4.22E-16 | 27.67545178 | CSRNP2 |
| 219165_at | -1.68982725 | 6.736239707 | -7.231207501 | 8.08E-10 | 2.37E-09 | 11.22608217 | PDLIM2 |
| 209194_at | 1.689280187 | 7.599457982 | 13.18373627 | 9.75E-20 | 1.01E-18 | 34.13678896 | CETN2 |
| 213410_at | 1.688819392 | 6.617022998 | 11.6846106 | 2.18E-17 | 1.64E-16 | 28.69121121 | EDRF1 |
| 224436_s_at | 1.688661675 | 5.977084254 | 9.433083203 | 1.21E-13 | 5.65E-13 | 20.02936153 | NIPSNAP3A |
| 203239_s_at | 1.688544617 | 5.514006725 | 13.4669181 | 3.62E-20 | 4.00E-19 | 35.13363063 | CNOT3 |
| 209763_at | 1.688300738 | 6.728658967 | 3.862956936 | 0.000267979 | 0.000435831 | -1.27287946 | CHRDL1 |
| 1562719_at | -1.688261431 | 5.241782801 | -12.77957595 | 4.08E-19 | 3.87E-18 | 32.6963912 | LOC101928476 |
| 218244_at | 1.686725872 | 7.174388087 | 13.51660761 | 3.05E-20 | 3.40E-19 | 35.30748817 | NOL8 |
| 221952_x_at | 1.686323091 | 9.991317366 | 14.11774424 | 3.87E-21 | 4.93E-20 | 37.38576591 | TRMT5 |
| 221249_s_at | 1.686225807 | 6.335306535 | 9.385966424 | 1.46E-13 | 6.73E-13 | 19.84305078 | FAM117A |
| 220424_at | -1.686049716 | 4.537830435 | -22.55146503 | 8.73E-32 | 6.10E-30 | 62.06355276 | NPHS2 |
| 214594_x_at | 1.684819611 | 8.979614476 | 12.73200637 | 4.83E-19 | 4.54E-18 | 32.52549431 | ATP8B1 |
| 213175_s_at | 1.684294393 | 8.922335367 | 11.46838331 | 4.88E-17 | 3.49E-16 | 27.88303186 | SNRPB |
| 218218_at | 1.683962582 | 5.478257135 | 10.64227258 | 1.11E-15 | 6.59E-15 | 24.74602258 | APPL2 |
| 211946_s_at | 1.683288773 | 9.089069329 | 14.75544677 | 4.56E-22 | 6.65E-21 | 39.540042 | PRRC2C |
| 231252_at | 1.682876045 | 5.934281882 | 10.86739541 | 4.69E-16 | 2.93E-15 | 25.60837994 | KANSL1L |
| 203974_at | -1.682823412 | 6.69700462 | -9.673649153 | 4.71E-14 | 2.31E-13 | 20.97799031 | HDHD1 |
| 1555575_a_at | 1.681474701 | 9.442959545 | 17.48856788 | 8.39E-26 | 2.18E-24 | 48.2016749 | KDELR1 |
| 71933_at | -1.680529357 | 5.803754486 | -23.07900678 | 2.39E-32 | 1.83E-30 | 63.36655074 | WNT6 |
| 201133_s_at | 1.680296168 | 8.188826939 | 10.23401121 | 5.32E-15 | 2.93E-14 | 23.16872099 | PJA2 |
| 209027_s_at | 1.680121676 | 6.948262341 | 7.669112572 | 1.39E-10 | 4.47E-10 | 12.98084231 | ABI1 |
| 244427_at | 1.680006703 | 5.695697013 | 7.409081002 | 3.95E-10 | 1.20E-09 | 11.93808802 | KIF23 |
| 209099_x_at | 1.679662885 | 8.411488962 | 6.626250647 | 9.08E-09 | 2.37E-08 | 8.819106112 | JAG1 |
| 223294_at | 1.679477107 | 6.934153924 | 13.47944713 | 3.47E-20 | 3.84E-19 | 35.17749796 | PBDC1 |
| 235072_s_at | -1.679354083 | 7.645775152 | -8.881031563 | 1.08E-12 | 4.48E-12 | 17.83715785 | KIF13A |
| 203303_at | 1.678941551 | 7.998958307 | 8.308042676 | 1.07E-11 | 3.92E-11 | 15.54519413 | DYNLT3 |
| 227951_s_at | 1.678762652 | 6.299570679 | 11.95466968 | 8.07E-18 | 6.44E-17 | 29.6927415 | FAM98C |
| 223358_s_at | -1.678285023 | 6.699381772 | -8.672249447 | 2.49E-12 | 9.85E-12 | 17.00355532 | PDE7A |
| 209265_s_at | 1.678040641 | 8.000467191 | 10.72954066 | 7.92E-16 | 4.81E-15 | 25.08095516 | METTL3 |
| 219038_at | 1.677559414 | 6.011181148 | 13.04272588 | 1.60E-19 | 1.61E-18 | 33.63659631 | MORC4 |
| 235648_at | 1.677481288 | 5.582067029 | 12.00642678 | 6.67E-18 | 5.39E-17 | 29.88367626 | ZNF567 |
| 218066_at | 1.677334033 | 7.475715086 | 9.516047469 | 8.76E-14 | 4.15E-13 | 20.35702162 | SLC12A7 |
| 242584_at | 1.676588194 | 5.200242406 | 9.17975646 | 3.30E-13 | 1.46E-12 | 19.02581479 | FAM161A |
| 209791_at | -1.676497113 | 8.133174666 | -4.229075831 | 7.77E-05 | 0.000133811 | -0.081298075 | PADI2 |
| 239742_at | 1.676294697 | 6.934931127 | 6.466327252 | 1.71E-08 | 4.33E-08 | 8.188329727 | LOC101930415 |
| 221539_at | 1.674846626 | 7.830579015 | 8.650378219 | 2.71E-12 | 1.07E-11 | 16.91611488 | EIF4EBP1 |
| 223434_at | 1.674756193 | 5.052680148 | 4.625562393 | 1.92E-05 | 3.53E-05 | 1.27520965 | GBP3 |
| 204392_at | 1.674435081 | 5.670976988 | 12.99374364 | 1.91E-19 | 1.89E-18 | 33.46225406 | CAMK1 |
| 1570210_x_at | 1.673906342 | 6.939351065 | 11.67221419 | 2.29E-17 | 1.71E-16 | 28.64502787 | PPP6R2 |
| 225128_at | 1.673314297 | 6.523752371 | 10.72200346 | 8.15E-16 | 4.94E-15 | 25.05205947 | KDELC2 |
| 229618_at | 1.673218932 | 5.732771694 | 10.03792135 | 1.14E-14 | 6.00E-14 | 22.40532926 | SNX16 |
| 228495_at | 1.673207465 | 6.03531222 | 13.65294831 | 1.90E-20 | 2.18E-19 | 35.78290546 | GPATCH11 |
| 209734_at | 1.672307045 | 7.261773171 | 4.864009599 | 8.08E-06 | 1.54E-05 | 2.119785518 | NCKAP1L |
| 235027_at | 1.671900353 | 5.702498905 | 12.66964879 | 6.04E-19 | 5.61E-18 | 32.30103786 | ZNF736 |
| 219077_s_at | 1.671513064 | 6.372063081 | 6.540142449 | 1.28E-08 | 3.28E-08 | 8.47911629 | WWOX |
| 231015_at | -1.67142217 | 6.754399024 | -12.00212922 | 6.78E-18 | 5.47E-17 | 29.86783481 | KLF15 |
| 213294_at | 1.671322026 | 7.505828673 | 8.960322638 | 7.89E-13 | 3.33E-12 | 18.15316678 | EIF2AK2 |
| 227367_at | 1.671022443 | 6.415950098 | 7.136670862 | 1.18E-09 | 3.39E-09 | 10.84826847 | SLCO3A1 |
| 201561_s_at | 1.670967098 | 7.94676638 | 14.45459935 | 1.24E-21 | 1.71E-20 | 38.53017091 | CLSTN1 |
| 1554588_a_at | 1.669815521 | 4.061811889 | 10.55127656 | 1.57E-15 | 9.19E-15 | 24.39593102 | TTC30B |
| 210073_at | 1.669478212 | 4.236139795 | 4.689190903 | 1.53E-05 | 2.84E-05 | 1.49860825 | ST8SIA1 |
| 213058_at | 1.669146451 | 6.603241582 | 10.00372424 | 1.30E-14 | 6.80E-14 | 22.27183114 | TTC28 |
| 209409_at | -1.668559731 | 6.684108316 | -6.504022134 | 1.48E-08 | 3.76E-08 | 8.336744793 | GRB10 |
| 215543_s_at | -1.668433197 | 6.249468982 | -16.52777955 | 1.56E-24 | 3.36E-23 | 45.25976779 | LARGE |
| 1553544_at | -1.668088319 | 4.772881845 | -17.95932217 | 2.08E-26 | 5.99E-25 | 49.60407966 | GPR101 |
| 204951_at | 1.667450777 | 6.502531271 | 4.700590786 | 1.47E-05 | 2.73E-05 | 1.538788497 | RHOH |
| 226112_at | -1.667203469 | 8.39516992 | -11.98190547 | 7.30E-18 | 5.86E-17 | 29.79325659 | SGCB |
| 222432_s_at | -1.665892159 | 7.795088975 | -12.55851432 | 9.00E-19 | 8.17E-18 | 31.8997944 | CCDC47 |
| 203211_s_at | 1.665890148 | 6.802037007 | 11.51984181 | 4.03E-17 | 2.91E-16 | 28.07586603 | MTMR2 |
| 222811_at | 1.665731066 | 5.291006302 | 8.758704346 | 1.76E-12 | 7.09E-12 | 17.34899376 | CMTR2 |
| 234973_at | 1.665618102 | 6.63300454 | 6.324028359 | 3.01E-08 | 7.40E-08 | 7.629655653 | SLC38A5 |
| 206857_s_at | 1.665323196 | 4.824531476 | 6.959173352 | 2.40E-09 | 6.68E-09 | 10.14034042 | FKBP1B |
| 222398_s_at | 1.665297025 | 8.23941017 | 16.38250945 | 2.44E-24 | 5.07E-23 | 44.80543511 | EFTUD2 |
| 202488_s_at | -1.665108433 | 5.85393693 | -19.76558175 | 1.24E-28 | 5.17E-27 | 54.75867329 | FXYD3 |
| 209379_s_at | -1.665015733 | 6.901815051 | -11.35093698 | 7.57E-17 | 5.24E-16 | 27.44175693 | CCSER2 |
| 209759_s_at | -1.664562045 | 7.031596491 | -11.28663697 | 9.63E-17 | 6.57E-16 | 27.19948801 | ECI1 |
| 208334_at | 1.663924369 | 5.792300383 | 4.991697337 | 5.05E-06 | 9.86E-06 | 2.579965507 | NDST4 |
| 222572_at | -1.663823953 | 7.755445667 | -7.001697846 | 2.03E-09 | 5.69E-09 | 10.30975456 | PDP1 |
| 212108_at | 1.663082998 | 7.824415211 | 20.54017936 | 1.53E-29 | 7.66E-28 | 56.86471453 | FAF2 |
| 225475_at | 1.662949812 | 6.549156287 | 12.36414521 | 1.81E-18 | 1.58E-17 | 31.1943143 | MIER1 |
| 224831_at | -1.662885923 | 6.579746819 | -8.033369091 | 3.22E-11 | 1.11E-10 | 14.44309531 | CPEB4 |
| 222752_s_at | 1.662353506 | 5.697117849 | 8.082272288 | 2.64E-11 | 9.25E-11 | 14.63939491 | TMEM206 |
| 240554_at | 1.662035569 | 6.582692643 | 10.60673719 | 1.27E-15 | 7.50E-15 | 24.60940896 | AKAP8L |
| 222649_at | -1.661455702 | 6.746392376 | -13.08229588 | 1.39E-19 | 1.41E-18 | 33.77721474 | XPO4 |
| 226650_at | 1.660992632 | 6.450814944 | 12.54256001 | 9.53E-19 | 8.63E-18 | 31.84206499 | ZFAND2A |
| 221912_s_at | -1.660991228 | 7.071785626 | -14.31124958 | 2.01E-21 | 2.67E-20 | 38.04493396 | CCDC28B |
| 217750_s_at | 1.66076113 | 8.030714722 | 15.60814039 | 2.83E-23 | 4.97E-22 | 42.34035497 | UBE2Z |
| 226354_at | -1.660526055 | 6.666623834 | -10.2612293 | 4.79E-15 | 2.65E-14 | 23.27439383 | LACTB |
| 1559946_s_at | 1.659377365 | 8.479462478 | 13.60391166 | 2.25E-20 | 2.56E-19 | 35.61218953 | RUVBL2 |
| 228077_at | 1.659304506 | 8.105162656 | 10.38270908 | 2.99E-15 | 1.70E-14 | 23.74514942 | MRI1 |
| 223893_at | -1.659075055 | 4.306712239 | -20.42386787 | 2.09E-29 | 1.00E-27 | 56.55230577 | ENAM |
| 203408_s_at | -1.658834527 | 7.764486332 | -4.543416877 | 2.58E-05 | 4.67E-05 | 0.989026274 | SATB1 |
| 202655_at | 1.658599282 | 7.96525908 | 13.3215899 | 6.01E-20 | 6.46E-19 | 34.62333333 | MANF |
| 202123_s_at | 1.658541618 | 7.35155425 | 11.18563046 | 1.41E-16 | 9.37E-16 | 26.81795786 | ABL1 |
| 225144_at | 1.657787608 | 8.119669675 | 8.591056197 | 3.44E-12 | 1.34E-11 | 16.67884819 | BMPR2 |
| 207186_s_at | 1.657596769 | 8.515490681 | 13.18974514 | 9.55E-20 | 9.94E-19 | 34.15804736 | BPTF |
| 203454_s_at | 1.657456279 | 7.816896194 | 14.43745165 | 1.32E-21 | 1.80E-20 | 38.47226393 | ATOX1 |
| 206060_s_at | 1.656527812 | 4.909442904 | 4.666329262 | 1.66E-05 | 3.07E-05 | 1.418171157 | PTPN22 |
| 216060_s_at | 1.656325891 | 6.283595816 | 7.624729586 | 1.66E-10 | 5.28E-10 | 12.80275109 | DAAM1 |
| 204012_s_at | 1.655950514 | 6.128295546 | 13.53128489 | 2.90E-20 | 3.25E-19 | 35.35878179 | LCMT2 |
| 212803_at | 1.655704845 | 6.82023168 | 10.98798357 | 2.97E-16 | 1.90E-15 | 26.06804803 | NAB2 |
| 221652_s_at | 1.655622831 | 7.341872778 | 11.57494284 | 3.28E-17 | 2.40E-16 | 28.28200437 | ASUN |
| 235435_at | 1.655481247 | 6.676244095 | 11.37958893 | 6.80E-17 | 4.75E-16 | 27.54955745 | AASDH |
| 223307_at | 1.655216968 | 6.331015398 | 7.594932541 | 1.87E-10 | 5.91E-10 | 12.68320802 | CDCA3 |
| 203643_at | 1.655171703 | 6.586995894 | 13.17599535 | 1.00E-19 | 1.04E-18 | 34.10939603 | ERF |
| 227395_at | 1.655125685 | 5.676528501 | 12.16298724 | 3.77E-18 | 3.14E-17 | 30.45923892 | TBCEL |
| 208959_s_at | 1.654831913 | 7.630038997 | 14.80615227 | 3.85E-22 | 5.69E-21 | 39.70911824 | ERP44 |
| 213620_s_at | 1.654415785 | 7.871703491 | 8.658020821 | 2.63E-12 | 1.04E-11 | 16.94667204 | ICAM2 |
| 208816_x_at | 1.654393236 | 9.495294535 | 8.537540497 | 4.26E-12 | 1.64E-11 | 16.46468692 | ANXA2P2 |
| 214104_at | 1.653748021 | 5.788192962 | 11.18977558 | 1.39E-16 | 9.24E-16 | 26.83363806 | GPR161 |
| 211452_x_at | 1.653624423 | 10.49217301 | 15.2837102 | 8.05E-23 | 1.32E-21 | 41.28562766 | LRRFIP1 |
| 212792_at | 1.65334891 | 5.927468532 | 8.150216923 | 2.01E-11 | 7.13E-11 | 14.91208197 | DPY19L1 |
| 203712_at | 1.652523412 | 6.84178805 | 15.9062775 | 1.09E-23 | 2.04E-22 | 43.29811177 | KIAA0020 |
| 223312_at | -1.651417691 | 6.716766618 | -12.54007848 | 9.62E-19 | 8.70E-18 | 31.83308291 | PRADC1 |
| 226962_at | 1.651389496 | 5.970571945 | 10.02304058 | 1.20E-14 | 6.33E-14 | 22.34725109 | ZBTB41 |
| 217728_at | 1.651157721 | 8.948214638 | 6.586848459 | 1.06E-08 | 2.75E-08 | 8.66343046 | S100A6 |
| 218108_at | 1.650684777 | 7.114354457 | 10.05549704 | 1.06E-14 | 5.62E-14 | 22.47389933 | UBR7 |
| 204249_s_at | 1.650495503 | 8.466707248 | 5.018417662 | 4.57E-06 | 8.97E-06 | 2.676919792 | LMO2 |
| 225414_at | 1.650306582 | 7.172914809 | 13.0318206 | 1.67E-19 | 1.67E-18 | 33.59780759 | RNF149 |
| 226200_at | 1.650177859 | 7.570715591 | 15.36500649 | 6.19E-23 | 1.04E-21 | 41.55115351 | VARS2 |
| 201069_at | 1.650130135 | 7.73967313 | 4.511901419 | 2.88E-05 | 5.20E-05 | 0.879914957 | MMP2 |
| 211481_at | -1.649944433 | 4.181521552 | -12.44077416 | 1.38E-18 | 1.22E-17 | 31.47301006 | SLCO1A2 |
| 202491_s_at | 1.649874714 | 7.500732375 | 9.286229442 | 2.17E-13 | 9.79E-13 | 19.44814401 | IKBKAP |
| 204917_s_at | 1.647981283 | 6.38671694 | 7.171894347 | 1.02E-09 | 2.97E-09 | 10.98898254 | MLLT3 |
| 225770_at | 1.647784092 | 7.194506673 | 18.1966 | 1.04E-26 | 3.19E-25 | 50.30145915 | RSPRY1 |
| 201631_s_at | 1.647390008 | 7.508512243 | 7.267925912 | 6.97E-10 | 2.06E-09 | 11.37294897 | IER3 |
| 204759_at | 1.646950366 | 7.120839538 | 8.233919698 | 1.44E-11 | 5.20E-11 | 15.24791404 | RCBTB2 |
| 222430_s_at | 1.646832188 | 8.592493114 | 15.1574151 | 1.21E-22 | 1.93E-21 | 40.87149002 | YTHDF2 |
| 204205_at | 1.646237327 | 6.590770125 | 6.145096704 | 6.09E-08 | 1.45E-07 | 6.931114732 | APOBEC3G |
| 229232_at | 1.646085125 | 5.269392997 | 10.74922127 | 7.35E-16 | 4.48E-15 | 25.15637712 | LRRC57 |
| 204202_at | 1.645894216 | 5.091867493 | 10.47237847 | 2.12E-15 | 1.23E-14 | 24.09169173 | IQCE |
| 203737_s_at | 1.645644818 | 6.671177055 | 13.86163959 | 9.27E-21 | 1.12E-19 | 36.50600094 | PPRC1 |
| 214626_s_at | 1.645389084 | 8.550975961 | 20.45239873 | 1.94E-29 | 9.34E-28 | 56.6290624 | GANAB |
| 205692_s_at | -1.645166541 | 4.610294207 | -8.548742927 | 4.07E-12 | 1.57E-11 | 16.5095261 | CD38 |
| 201591_s_at | 1.644955432 | 8.568199749 | 14.09816095 | 4.14E-21 | 5.24E-20 | 37.31878984 | NISCH |
| 213008_at | 1.644836874 | 6.586996347 | 9.042741874 | 5.69E-13 | 2.44E-12 | 18.48127103 | FANCI |
| 200662_s_at | 1.644828338 | 9.732001532 | 11.22027071 | 1.24E-16 | 8.30E-16 | 26.94893522 | TOMM20 |
| 217711_at | -1.644039733 | 5.182228914 | -27.67576945 | 7.39E-37 | 1.21E-34 | 73.78586001 | TEK |
| 225840_at | -1.643392142 | 6.13496524 | -13.85460297 | 9.50E-21 | 1.15E-19 | 36.48171049 | TEF |
| 221236_s_at | 1.643369046 | 5.583385262 | 3.325635706 | 0.001477773 | 0.002217377 | -2.896700568 | STMN4 |
| 201054_at | 1.64291342 | 9.26412174 | 11.14455403 | 1.64E-16 | 1.08E-15 | 26.66246807 | HNRNPA0 |
| 218078_s_at | 1.642668236 | 7.845645629 | 6.474089823 | 1.66E-08 | 4.21E-08 | 8.218879237 | ZDHHC3 |
| 242136_x_at | 1.642583053 | 4.881701209 | 8.804203511 | 1.47E-12 | 5.97E-12 | 17.53065099 | MGC70870 |
| 202116_at | 1.641369779 | 7.071287969 | 12.03776363 | 5.95E-18 | 4.84E-17 | 29.99912055 | DPF2 |
| 1552257_a_at | 1.641284145 | 6.501232562 | 12.21592158 | 3.11E-18 | 2.62E-17 | 30.65315862 | TTLL12 |
| 223542_at | 1.640894391 | 5.404071848 | 9.035043097 | 5.86E-13 | 2.52E-12 | 18.45063949 | ANKRD32 |
| 205034_at | 1.640692569 | 4.550813286 | 7.196088112 | 9.30E-10 | 2.71E-09 | 11.08567318 | CCNE2 |
| 226642_s_at | 1.640376371 | 7.30357481 | 9.37907393 | 1.50E-13 | 6.90E-13 | 19.81578274 | NUDCD2 |
| 233993_at | -1.640289783 | 4.417627661 | -17.25807373 | 1.67E-25 | 4.17E-24 | 47.50575919 | MUC3 |
| 221543_s_at | 1.640098863 | 7.122591014 | 10.23923655 | 5.21E-15 | 2.87E-14 | 23.18901366 | ERLIN2 |
| 236926_at | -1.639684424 | 6.108342393 | -8.148096278 | 2.03E-11 | 7.19E-11 | 14.90357199 | TBX1 |
| 214061_at | 1.639583825 | 5.425582503 | 9.633075245 | 5.53E-14 | 2.69E-13 | 20.81831307 | TBC1D31 |
| 226435_at | 1.638060999 | 4.941496659 | 3.931840067 | 0.000213221 | 0.000350513 | -1.053583655 | PAPLN |
| 226777_at | 1.637882981 | 5.030717501 | 4.183332237 | 9.10E-05 | 0.00015544 | -0.23356911 | ADAM12 |
| 222516_at | 1.637605516 | 6.819509746 | 11.30904339 | 8.85E-17 | 6.07E-16 | 27.28396484 | AP3M1 |
| 217840_at | 1.636909779 | 6.978861188 | 13.90566359 | 7.97E-21 | 9.73E-20 | 36.65782816 | DDX41 |
| 238590_x_at | 1.636863662 | 6.689850396 | 12.19564176 | 3.34E-18 | 2.81E-17 | 30.57890644 | TMEM107 |
| 203675_at | 1.636789758 | 6.941447243 | 6.893743054 | 3.12E-09 | 8.56E-09 | 9.879930514 | NUCB2 |
| 209082_s_at | 1.636649696 | 6.757704959 | 6.505746362 | 1.47E-08 | 3.74E-08 | 8.343537564 | COL18A1 |
| 202347_s_at | 1.636518624 | 8.063869815 | 13.74956492 | 1.36E-20 | 1.59E-19 | 36.11836564 | UBE2K |
| 200602_at | 1.636517495 | 7.914444635 | 5.59509103 | 5.17E-07 | 1.12E-06 | 4.818292268 | APP |
| 203733_at | -1.636204411 | 7.045523862 | -20.49160233 | 1.75E-29 | 8.50E-28 | 56.73440064 | DEXI |
| 227732_at | 1.635754824 | 6.276540995 | 11.05634316 | 2.29E-16 | 1.48E-15 | 26.32791289 | ATXN7L1 |
| 222785_x_at | 1.635665818 | 6.434051789 | 8.240244515 | 1.40E-11 | 5.08E-11 | 15.27328518 | C11orf1 |
| 232266_x_at | 1.635646428 | 10.19357301 | 12.18406771 | 3.49E-18 | 2.93E-17 | 30.53650659 | CDK13 |
| 203552_at | 1.63563784 | 6.087214657 | 9.705697932 | 4.16E-14 | 2.05E-13 | 21.10402245 | MAP4K5 |
| 218189_s_at | 1.635514092 | 6.528866942 | 12.99922437 | 1.87E-19 | 1.86E-18 | 33.48177672 | NANS |
| 1568594_s_at | 1.635238029 | 5.615448758 | 10.90334266 | 4.09E-16 | 2.58E-15 | 25.74557365 | TRIM52 |
| 223240_at | 1.634805323 | 5.76590723 | 16.20815774 | 4.21E-24 | 8.42E-23 | 44.25679086 | FBXO8 |
| 232323_s_at | 1.633442586 | 7.312201872 | 12.05195686 | 5.65E-18 | 4.61E-17 | 30.05136856 | TTC17 |
| 219946_x_at | -1.633314216 | 5.952252419 | -12.97629418 | 2.03E-19 | 2.01E-18 | 33.40007259 | MYH14 |
| 205694_at | -1.633147195 | 3.472737136 | -9.767847875 | 3.26E-14 | 1.63E-13 | 21.34818546 | TYRP1 |
| 235266_at | 1.632846627 | 6.214270067 | 9.419840572 | 1.28E-13 | 5.93E-13 | 19.97701345 | ATAD2 |
| 209579_s_at | 1.632695208 | 8.715777037 | 16.99465871 | 3.72E-25 | 8.80E-24 | 46.70288489 | MBD4 |
| 211686_s_at | 1.631659279 | 6.95219763 | 12.38461855 | 1.68E-18 | 1.47E-17 | 31.26884656 | MAK16 |
| 223712_at | -1.631232321 | 5.915280676 | -12.23950581 | 2.85E-18 | 2.42E-17 | 30.73944558 | PCBD2 |
| 226668_at | 1.631032645 | 5.683220257 | 8.196294013 | 1.67E-11 | 6.00E-11 | 15.0969677 | WDSUB1 |
| 209336_at | 1.630922319 | 6.360253196 | 12.78332079 | 4.02E-19 | 3.82E-18 | 32.70983264 | PWP2 |
| 58696_at | 1.629390162 | 7.200984482 | 11.41033326 | 6.06E-17 | 4.26E-16 | 27.66512434 | EXOSC4 |
| 206158_s_at | -1.629157553 | 10.50158657 | -13.77757478 | 1.24E-20 | 1.46E-19 | 36.21539464 | CNBP |
| 212799_at | 1.628484465 | 6.692011363 | 17.66404884 | 4.98E-26 | 1.35E-24 | 48.72739304 | STX6 |
| 223340_at | 1.628444761 | 5.758612577 | 6.931142224 | 2.69E-09 | 7.43E-09 | 10.02873811 | ATL1 |
| 235721_at | 1.628297894 | 7.782229425 | 13.61973641 | 2.13E-20 | 2.44E-19 | 35.66731534 | DTX3 |
| 217849_s_at | 1.628073535 | 5.932800144 | 11.07581034 | 2.13E-16 | 1.39E-15 | 26.40182076 | CDC42BPB |
| 209326_at | 1.627324181 | 5.370736098 | 14.15528363 | 3.41E-21 | 4.37E-20 | 37.51401597 | SLC35A2 |
| 225302_at | 1.627198625 | 7.103799739 | 10.41211659 | 2.67E-15 | 1.53E-14 | 23.85888882 | TMX3 |
| 221931_s_at | 1.626058373 | 7.936206755 | 9.748554072 | 3.52E-14 | 1.75E-13 | 21.27242212 | SEH1L |
| 208939_at | 1.626020115 | 7.105222285 | 11.52826387 | 3.90E-17 | 2.83E-16 | 28.10739694 | SEPHS1 |
| 201705_at | -1.625878131 | 7.791426636 | -13.74761451 | 1.37E-20 | 1.60E-19 | 36.1116055 | PSMD7 |
| 225242_s_at | 1.625331416 | 6.1380187 | 3.895810769 | 0.000240362 | 0.000392892 | -1.168580999 | CCDC80 |
| 212761_at | 1.625118746 | 7.815396386 | 9.316225771 | 1.93E-13 | 8.75E-13 | 19.56698725 | TCF7L2 |
| 1556203_a_at | 1.624870768 | 5.76273438 | 10.21979819 | 5.62E-15 | 3.09E-14 | 23.11351126 | SRGAP2 |
| 244024_at | 1.624866184 | 6.229287563 | 11.19582959 | 1.35E-16 | 9.05E-16 | 26.85653571 | ZNF182 |
| 204544_at | 1.624548938 | 6.411927882 | 13.71917736 | 1.51E-20 | 1.75E-19 | 36.0129865 | HPS5 |
| 212898_at | 1.624403734 | 6.600081267 | 12.32936866 | 2.06E-18 | 1.78E-17 | 31.06759224 | TTI1 |
| 1553984_s_at | 1.624264934 | 7.490693168 | 10.83887385 | 5.22E-16 | 3.24E-15 | 25.49942637 | DTYMK |
| 204617_s_at | 1.623957675 | 6.770696608 | 14.89043186 | 2.92E-22 | 4.38E-21 | 39.98942751 | ACD |
| 227728_at | -1.623831891 | 7.799608759 | -9.702122772 | 4.22E-14 | 2.08E-13 | 21.08996728 | PPM1A |
| 226931_at | -1.623332474 | 5.672106694 | -6.42800852 | 2.00E-08 | 5.01E-08 | 8.037634462 | TMTC1 |
| 220750_s_at | 1.622535729 | 7.070712582 | 12.39972835 | 1.60E-18 | 1.40E-17 | 31.32381956 | LEPRE1 |
| 212057_at | 1.622424123 | 7.255406338 | 7.303396194 | 6.05E-10 | 1.80E-09 | 11.51488269 | GSE1 |
| 209035_at | 1.621881229 | 6.590922706 | 6.654787395 | 8.10E-09 | 2.13E-08 | 8.931954628 | MDK |
| 205227_at | 1.621772364 | 6.46002469 | 5.856098443 | 1.89E-07 | 4.28E-07 | 5.813758018 | IL1RAP |
| 208614_s_at | 1.621536667 | 7.909113598 | 11.54092702 | 3.72E-17 | 2.71E-16 | 28.15479017 | FLNB |
| 240715_at | 1.621393837 | 6.644320631 | 4.352038929 | 5.07E-05 | 8.90E-05 | 0.332524234 | TBX5 |
| 219067_s_at | 1.62135829 | 6.291782742 | 11.34448381 | 7.75E-17 | 5.36E-16 | 27.41746431 | NSMCE4A |
| 202117_at | 1.621333017 | 8.359471414 | 14.83847474 | 3.46E-22 | 5.15E-21 | 39.81672706 | ARHGAP1 |
| 227129_x_at | 1.621304192 | 7.055927331 | 7.108594559 | 1.32E-09 | 3.77E-09 | 10.73615721 | LINC01000 |
| 208384_s_at | -1.621256321 | 5.765231529 | -18.8712549 | 1.50E-27 | 5.31E-26 | 52.2503579 | MID2 |
| 217949_s_at | 1.620025469 | 7.877125337 | 12.01571468 | 6.45E-18 | 5.22E-17 | 29.91790524 | VKORC1 |
| 200959_at | 1.619785809 | 8.640569222 | 15.2933962 | 7.80E-23 | 1.28E-21 | 41.31730689 | FUS |
| 202111_at | 1.619737432 | 6.674827858 | 14.2556471 | 2.43E-21 | 3.17E-20 | 37.85601557 | SLC4A2 |
| 213122_at | 1.619657883 | 7.448692181 | 6.34774991 | 2.74E-08 | 6.77E-08 | 7.722604803 | TSPYL5 |
| 215537_x_at | 1.619540023 | 6.846205214 | 11.85054516 | 1.18E-17 | 9.20E-17 | 29.30763293 | DDAH2 |
| 200700_s_at | 1.618740508 | 8.177737604 | 9.089662249 | 4.72E-13 | 2.06E-12 | 18.66787919 | KDELR2 |
| 205978_at | 1.618595937 | 4.02371261 | 4.175499598 | 9.35E-05 | 0.000159427 | -0.259549097 | KL |
| 218256_s_at | 1.618360565 | 7.698117468 | 13.19674176 | 9.32E-20 | 9.72E-19 | 34.18279446 | NUP54 |
| 210739_x_at | -1.618138736 | 4.443335185 | -16.90705418 | 4.85E-25 | 1.13E-23 | 46.43406845 | SLC4A4 |
| 206625_at | -1.618067676 | 5.357832534 | -12.16267116 | 3.77E-18 | 3.14E-17 | 30.45807995 | PRPH2 |
| 219351_at | 1.61799692 | 5.99286946 | 9.428089571 | 1.24E-13 | 5.75E-13 | 20.00962323 | TRAPPC2 |
| 212870_at | -1.617990061 | 7.413960493 | -12.34930139 | 1.91E-18 | 1.66E-17 | 31.14024343 | SOS2 |
| 225769_at | 1.61796322 | 6.122741474 | 9.076806906 | 4.97E-13 | 2.15E-12 | 18.61676508 | COG6 |
| 212297_at | 1.617695332 | 6.667668241 | 10.1400332 | 7.65E-15 | 4.13E-14 | 22.80330976 | ATP13A3 |
| 1556043_a_at | -1.616896016 | 5.035802654 | -8.559154705 | 3.91E-12 | 1.51E-11 | 16.55119646 | TTN-AS1 |
| 212222_at | -1.616706198 | 7.12522083 | -12.87551701 | 2.90E-19 | 2.80E-18 | 33.04019505 | PSME4 |
| 209165_at | 1.616550198 | 7.194270552 | 17.99384154 | 1.88E-26 | 5.48E-25 | 49.70592698 | AATF |
| 231440_at | -1.616525243 | 4.602522434 | -14.53367443 | 9.53E-22 | 1.33E-20 | 38.79671977 | LINC00943 |
| 228233_at | 1.616305818 | 6.463604634 | 4.161194415 | 9.82E-05 | 0.000167195 | -0.306926777 | FREM1 |
| 224257_s_at | -1.615879986 | 3.751833223 | -13.06128659 | 1.50E-19 | 1.51E-18 | 33.70257961 | PBOV1 |
| 210243_s_at | 1.615777211 | 7.011470098 | 11.30934596 | 8.84E-17 | 6.06E-16 | 27.28510519 | B4GALT3 |
| 205698_s_at | -1.615770416 | 6.858414416 | -7.382028369 | 4.41E-10 | 1.33E-09 | 11.82971726 | MAP2K6 |
| 203263_s_at | 1.61494968 | 5.487038184 | 7.715705324 | 1.15E-10 | 3.74E-10 | 13.16783385 | ARHGEF9 |
| 226588_at | 1.614840862 | 6.464266703 | 13.84506293 | 9.81E-21 | 1.18E-19 | 36.44876814 | CWC22 |
| 201395_at | 1.614753677 | 9.014443769 | 12.75084639 | 4.52E-19 | 4.26E-18 | 32.59321254 | RBM5 |
| 204279_at | 1.614610495 | 5.950981102 | 5.553791579 | 6.06E-07 | 1.30E-06 | 4.662125212 | PSMB9 |
| 203947_at | 1.613897059 | 7.348988782 | 12.96414079 | 2.12E-19 | 2.09E-18 | 33.35674093 | CSTF3 |
| 235921_at | -1.613685507 | 4.659969212 | -18.72733876 | 2.26E-27 | 7.66E-26 | 51.83878621 | LOC102723721 |
| 203221_at | -1.613313145 | 6.38915998 | -7.120067437 | 1.26E-09 | 3.61E-09 | 10.78196391 | TLE1 |
| 225945_at | 1.613302433 | 7.764562478 | 7.18776032 | 9.62E-10 | 2.79E-09 | 11.05238757 | ZNF655 |
| 227917_at | 1.613247474 | 7.205357907 | 7.212265676 | 8.72E-10 | 2.54E-09 | 11.15034409 | LOC100506990 |
| 210849_s_at | 1.613232325 | 6.711573902 | 16.7502452 | 7.84E-25 | 1.77E-23 | 45.95063356 | VPS41 |
| 216071_x_at | 1.61308901 | 7.010988691 | 17.25543663 | 1.69E-25 | 4.20E-24 | 47.49776157 | MED12 |
| 212984_at | 1.613059796 | 7.211441252 | 13.14390795 | 1.12E-19 | 1.15E-18 | 33.99576643 | ATF2 |
| 218902_at | 1.612889479 | 6.518274514 | 11.20586285 | 1.30E-16 | 8.74E-16 | 26.89447464 | NOTCH1 |
| 222662_at | -1.612023134 | 7.808176694 | -6.319505017 | 3.07E-08 | 7.53E-08 | 7.611940308 | PPP1R3B |
| 223024_at | 1.61148811 | 7.255785281 | 13.43661091 | 4.03E-20 | 4.43E-19 | 35.02743444 | AP1M1 |
| 242455_at | 1.61055146 | 6.117332069 | 3.606770028 | 0.000615418 | 0.000963343 | -2.067004188 | POU3F2 |
| 226910_at | 1.610511274 | 6.520043467 | 12.30864311 | 2.22E-18 | 1.90E-17 | 30.99199897 | COMMD2 |
| 213428_s_at | 1.610443297 | 9.307172655 | 5.153553693 | 2.76E-06 | 5.55E-06 | 3.170572139 | COL6A1 |
| 219526_at | 1.609055402 | 6.264059352 | 14.64580368 | 6.56E-22 | 9.33E-21 | 39.17332692 | C14orf169 |
| 210428_s_at | 1.609007147 | 7.454859936 | 13.86009993 | 9.32E-21 | 1.12E-19 | 36.50068655 | HGS |
| 212469_at | 1.608853543 | 7.525565345 | 19.0797289 | 8.35E-28 | 3.07E-26 | 52.84260692 | NIPBL |
| 219761_at | -1.608452424 | 4.110651812 | -11.46679853 | 4.91E-17 | 3.51E-16 | 27.87708818 | CLEC1A |
| 1555724_s_at | 1.608340012 | 8.262255029 | 4.622481665 | 1.94E-05 | 3.57E-05 | 1.26443106 | TAGLN |
| 202869_at | 1.60815176 | 6.785232176 | 5.687250927 | 3.63E-07 | 7.97E-07 | 5.168151792 | OAS1 |
| 220761_s_at | 1.607895471 | 6.517013458 | 9.774176892 | 3.18E-14 | 1.59E-13 | 21.37303156 | TAOK3 |
| 224676_at | 1.607706913 | 8.276687047 | 8.394742108 | 7.54E-12 | 2.82E-11 | 15.89274377 | TMED4 |
| 226975_at | 1.606962979 | 7.782517752 | 8.512077657 | 4.72E-12 | 1.81E-11 | 16.36275178 | RNPC3 |
| 212825_at | 1.606352358 | 6.112768831 | 11.51485176 | 4.10E-17 | 2.96E-16 | 28.05718009 | PAXIP1 |
| 232542_at | -1.606060053 | 5.333569686 | -18.81466646 | 1.77E-27 | 6.13E-26 | 52.08879276 | COL9A2 |
| 1564063_a_at | -1.605565276 | 6.102997859 | -17.06346345 | 3.01E-25 | 7.22E-24 | 46.91338113 | ATP11B |
| 200885_at | 1.605453822 | 7.864650282 | 10.03861758 | 1.13E-14 | 5.98E-14 | 22.40804609 | RHOC |
| 220340_at | -1.605437684 | 4.872191349 | -9.741346474 | 3.62E-14 | 1.80E-13 | 21.24411115 | GREB1L |
| 201774_s_at | 1.604937872 | 6.04859584 | 9.381911113 | 1.49E-13 | 6.83E-13 | 19.8270076 | NCAPD2 |
| 225494_at | -1.604834133 | 7.49759965 | -15.07403626 | 1.60E-22 | 2.48E-21 | 40.59698456 | DYNLL2 |
| 232080_at | 1.604780944 | 5.331559664 | 6.167854197 | 5.57E-08 | 1.33E-07 | 7.019694578 | HECW2 |
| 205395_s_at | 1.604668731 | 5.779471865 | 12.12250893 | 4.37E-18 | 3.61E-17 | 30.3107174 | MRE11A |
| 228011_at | 1.604636374 | 7.160011104 | 10.1189805 | 8.30E-15 | 4.46E-14 | 22.72133654 | FAM92A1 |
| 224733_at | 1.604243813 | 7.29755681 | 11.60079014 | 2.98E-17 | 2.19E-16 | 28.37857791 | CMTM3 |
| 214198_s_at | 1.604161473 | 6.442000955 | 16.84163759 | 5.93E-25 | 1.36E-23 | 46.2327459 | DGCR2 |
| 203046_s_at | 1.604159305 | 6.221594514 | 7.183092407 | 9.80E-10 | 2.84E-09 | 11.03373184 | TIMELESS |
| 202420_s_at | 1.602753458 | 7.703684949 | 14.32071642 | 1.95E-21 | 2.59E-20 | 38.07705981 | DHX9 |
| 208437_at | -1.602443493 | 5.42874905 | -14.24022765 | 2.56E-21 | 3.32E-20 | 37.80355566 | CLCN1 |
| 222062_at | 1.60095027 | 5.307649243 | 8.628884974 | 2.96E-12 | 1.16E-11 | 16.83016612 | IL27RA |
| 1552402_at | -1.600000288 | 3.851327432 | -10.98037216 | 3.05E-16 | 1.95E-15 | 26.03908159 | CALML6 |
| 218713_at | 1.599618278 | 5.738984411 | 11.45758001 | 5.08E-17 | 3.62E-16 | 27.8425084 | ICE2 |
| 221211_s_at | -1.59953297 | 4.19252825 | -11.39252305 | 6.48E-17 | 4.54E-16 | 27.59818976 | MAP3K7CL |
| 200975_at | 1.599479051 | 8.52192334 | 10.25391172 | 4.92E-15 | 2.73E-14 | 23.24599073 | PPT1 |
| 224909_s_at | 1.599362484 | 6.613168204 | 6.462379855 | 1.74E-08 | 4.40E-08 | 8.172797583 | PREX1 |
| 227646_at | 1.599200883 | 6.414500452 | 5.79244504 | 2.41E-07 | 5.41E-07 | 5.569698684 | EBF1 |
| 219097_x_at | 1.599195876 | 7.560702772 | 20.25211162 | 3.32E-29 | 1.51E-27 | 56.08852186 | SMIM7 |
| 208758_at | 1.598678562 | 8.398145144 | 10.07121866 | 9.99E-15 | 5.31E-14 | 22.53521176 | ATIC |
| 213390_at | 1.598329661 | 7.959403082 | 15.33824185 | 6.75E-23 | 1.12E-21 | 41.46382727 | ZC3H4 |
| 212267_at | 1.598201081 | 7.091004042 | 13.92458731 | 7.47E-21 | 9.16E-20 | 36.72301491 | WAPAL |
| 200980_s_at | -1.597881763 | 8.785222043 | -15.97349244 | 8.82E-24 | 1.67E-22 | 43.51252859 | PDHA1 |
| 202355_s_at | 1.597755551 | 7.402137539 | 14.27183405 | 2.30E-21 | 3.01E-20 | 37.91105401 | GTF2F1 |
| 201788_at | 1.597417835 | 8.13006416 | 16.91425799 | 4.75E-25 | 1.11E-23 | 46.45620763 | DDX42 |
| 208859_s_at | 1.596535125 | 8.122885074 | 9.000109162 | 6.74E-13 | 2.86E-12 | 18.3116024 | ATRX |
| 226153_s_at | 1.596425942 | 7.845512884 | 11.31717091 | 8.59E-17 | 5.90E-16 | 27.31459295 | CNOT6L |
| 243198_at | 1.596337259 | 3.685077641 | 7.184021818 | 9.76E-10 | 2.84E-09 | 11.03744622 | TEX9 |
| 206717_at | -1.596170859 | 4.108818189 | -7.469637928 | 3.10E-10 | 9.54E-10 | 12.18076499 | MYH8 |
| 207170_s_at | 1.595929195 | 7.840926073 | 11.51823615 | 4.05E-17 | 2.93E-16 | 28.06985372 | LETMD1 |
| 1562869_at | -1.59572889 | 5.699285114 | -24.1455833 | 1.86E-33 | 1.68E-31 | 65.92902817 | LOC101929406 |
| 205917_at | 1.59549627 | 6.473367612 | 10.02117376 | 1.21E-14 | 6.37E-14 | 22.33996364 | ZNF264 |
| 207088_s_at | -1.595009166 | 7.437122953 | -11.19992757 | 1.33E-16 | 8.93E-16 | 26.87203285 | SLC25A11 |
| 225876_at | 1.594937903 | 6.283700207 | 10.52396668 | 1.74E-15 | 1.02E-14 | 24.29069351 | NIPAL3 |
| 241682_at | 1.594861809 | 5.967227231 | 8.101589058 | 2.44E-11 | 8.60E-11 | 14.71692634 | KLHL23 |
| 222401_s_at | 1.593362259 | 8.939336315 | 10.12734725 | 8.04E-15 | 4.32E-14 | 22.75391927 | TMEM50A |
| 231845_at | 1.593203399 | 6.822749511 | 15.46373498 | 4.50E-23 | 7.68E-22 | 41.8725076 | AARS2 |
| 201397_at | 1.593140834 | 7.174193925 | 7.503210976 | 2.71E-10 | 8.39E-10 | 12.31535535 | PHGDH |
| 226568_at | 1.592737583 | 5.217612733 | 6.469919408 | 1.69E-08 | 4.27E-08 | 8.202465713 | FAM102B |
| 204870_s_at | 1.592709262 | 5.035604193 | 4.577225437 | 2.28E-05 | 4.16E-05 | 1.106501399 | PCSK2 |
| 201564_s_at | 1.592315792 | 6.936654817 | 12.79869255 | 3.81E-19 | 3.63E-18 | 32.76498828 | FSCN1 |
| 234995_at | 1.591904037 | 4.517175989 | 12.19359597 | 3.37E-18 | 2.83E-17 | 30.57141316 | SPICE1 |
| 209259_s_at | 1.591552579 | 7.433203413 | 9.882684607 | 2.08E-14 | 1.07E-13 | 21.79847252 | SMC3 |
| 201263_at | 1.590991154 | 8.151175741 | 11.45741293 | 5.08E-17 | 3.62E-16 | 27.84188158 | TARS |
| 228479_at | 1.590399298 | 6.69006423 | 11.24896614 | 1.11E-16 | 7.51E-16 | 27.05733111 | SOAT1 |
| 201536_at | -1.590027247 | 8.430999943 | -17.99223426 | 1.89E-26 | 5.50E-25 | 49.70118778 | DUSP3 |
| 1554840_at | -1.589991827 | 4.131411494 | -24.7901926 | 4.16E-34 | 4.42E-32 | 67.43298372 | LOC280665 |
| 204905_s_at | 1.58987505 | 7.115690691 | 9.13108648 | 4.01E-13 | 1.76E-12 | 18.83251693 | EEF1E1 |
| 223389_s_at | 1.589194991 | 7.377646524 | 10.22064868 | 5.60E-15 | 3.08E-14 | 23.11681548 | ZNF581 |
| 225519_at | 1.588685363 | 7.23530674 | 11.17145113 | 1.48E-16 | 9.85E-16 | 26.76430538 | PPP4R2 |
| 200877_at | 1.588074001 | 10.28495774 | 12.06673723 | 5.35E-18 | 4.38E-17 | 30.10575165 | CCT4 |
| 209684_at | 1.588045165 | 7.402193171 | 6.926877494 | 2.74E-09 | 7.56E-09 | 10.01176377 | RIN2 |
| 213136_at | 1.587844863 | 7.897232681 | 15.26147297 | 8.66E-23 | 1.41E-21 | 41.21285375 | PTPN2 |
| 218552_at | -1.587791384 | 7.602515136 | -5.474061715 | 8.22E-07 | 1.74E-06 | 4.361762705 | ECHDC2 |
| 225881_at | 1.587681242 | 6.078914159 | 8.798233845 | 1.50E-12 | 6.10E-12 | 17.50682254 | SLC35B4 |
| 202325_s_at | -1.587515371 | 10.65981542 | -15.37139376 | 6.06E-23 | 1.02E-21 | 41.57198035 | ATP5J |
| 229544_at | -1.587498863 | 6.501032801 | -7.399689658 | 4.11E-10 | 1.24E-09 | 11.90046404 | RP11-391M1.4 |
| 239355_at | 1.586405367 | 5.924946269 | 10.98929327 | 2.95E-16 | 1.89E-15 | 26.07303167 | GMCL1 |
| 212322_at | 1.586242059 | 6.415846316 | 10.06543924 | 1.02E-14 | 5.42E-14 | 22.51267536 | SGPL1 |
| 219464_at | -1.586105667 | 4.905228352 | -18.74965614 | 2.12E-27 | 7.26E-26 | 51.90275606 | CA14 |
| 220355_s_at | 1.585902461 | 8.075764748 | 20.6171941 | 1.25E-29 | 6.40E-28 | 57.07084043 | PBRM1 |
| 217722_s_at | -1.585870781 | 9.662498348 | -14.39060363 | 1.54E-21 | 2.08E-20 | 38.31386966 | NGRN |
| 223197_s_at | 1.585508531 | 6.426830663 | 13.91006472 | 7.85E-21 | 9.60E-20 | 36.67299288 | SMARCAD1 |
| 225167_at | 1.585391968 | 6.422766621 | 5.73067611 | 3.07E-07 | 6.79E-07 | 5.333637212 | FRMD4A |
| 203391_at | 1.585389804 | 7.738713867 | 18.51301874 | 4.18E-27 | 1.35E-25 | 51.22171019 | FKBP2 |
| 222199_s_at | 1.585302842 | 6.862733632 | 14.10096306 | 4.10E-21 | 5.20E-20 | 37.32837624 | BIN3 |
| 203254_s_at | 1.585262026 | 6.107221345 | 11.18928489 | 1.39E-16 | 9.26E-16 | 26.83178198 | TLN1 |
| 225060_at | 1.585231153 | 6.020369336 | 7.310636361 | 5.87E-10 | 1.75E-09 | 11.54386091 | LRP11 |
| 225407_at | -1.584562051 | 5.983689074 | -9.872685114 | 2.16E-14 | 1.11E-13 | 21.75930874 | MBP |
| 223529_at | 1.584051761 | 6.03518229 | 3.07286728 | 0.003135778 | 0.004537302 | -3.602066095 | SYT4 |
| 209149_s_at | 1.583998229 | 6.040906505 | 12.2582601 | 2.66E-18 | 2.27E-17 | 30.80801219 | TM9SF1 |
| 219061_s_at | 1.583929826 | 7.394701945 | 11.37885263 | 6.82E-17 | 4.76E-16 | 27.54678839 | LAGE3 |
| 212240_s_at | 1.583558861 | 7.981169113 | 7.51281948 | 2.61E-10 | 8.09E-10 | 12.35388061 | PIK3R1 |
| 219507_at | 1.583152073 | 6.036771859 | 11.92866888 | 8.88E-18 | 7.03E-17 | 29.59669993 | RSRC1 |
| 396_f_at | -1.583122006 | 7.404516926 | -15.58826144 | 3.01E-23 | 5.29E-22 | 42.27610427 | EPOR |
| 227497_at | 1.582821194 | 7.936316839 | 6.402967337 | 2.20E-08 | 5.51E-08 | 7.939254274 | SOX6 |
| 225617_at | 1.582689199 | 5.099103477 | 11.83054341 | 1.27E-17 | 9.89E-17 | 29.2335052 | ODF2 |
| 225921_at | 1.582183281 | 7.643341693 | 8.794706089 | 1.53E-12 | 6.19E-12 | 17.49274038 | NIN |
| 209161_at | 1.582179373 | 7.276305996 | 13.00519472 | 1.83E-19 | 1.82E-18 | 33.50303914 | PRPF4 |
| 227064_at | -1.581991907 | 7.324568899 | -19.05959975 | 8.84E-28 | 3.22E-26 | 52.78562494 | ANKRD40 |
| 242195_x_at | 1.581511412 | 9.932749529 | 15.3753043 | 5.98E-23 | 1.01E-21 | 41.58472884 | NUMBL |
| 234177_at | -1.581502385 | 4.678079637 | -18.26796069 | 8.47E-27 | 2.62E-25 | 50.50996611 | LOC101927950 |
| 206349_at | -1.58065956 | 3.400283501 | -14.2855561 | 2.19E-21 | 2.88E-20 | 37.9576852 | LGI1 |
| 203422_at | 1.580625419 | 5.779044451 | 10.77109286 | 6.76E-16 | 4.14E-15 | 25.24014692 | POLD1 |
| 228240_at | 1.5796837 | 7.219245608 | 9.327020776 | 1.84E-13 | 8.40E-13 | 19.60974099 | AGAP1 |
| 230815_at | 1.579578127 | 5.025957903 | 9.045137409 | 5.63E-13 | 2.42E-12 | 18.49080156 | LOC389765 |
| 227337_at | -1.578631989 | 5.730705295 | -7.732067476 | 1.08E-10 | 3.52E-10 | 13.2335072 | ANKRD37 |
| 54632_at | 1.578249356 | 6.667978521 | 14.3463888 | 1.79E-21 | 2.39E-20 | 38.16412189 | THADA |
| 220746_s_at | 1.577874304 | 6.689067105 | 13.14647322 | 1.11E-19 | 1.15E-18 | 34.00485553 | UIMC1 |
| 32099_at | 1.577868072 | 6.94174814 | 11.4694388 | 4.86E-17 | 3.48E-16 | 27.8869903 | SAFB2 |
| 211071_s_at | 1.577776362 | 7.825617628 | 5.113659892 | 3.21E-06 | 6.40E-06 | 3.024276112 | MLLT11 |
| 217783_s_at | 1.577217546 | 8.794354334 | 9.311637781 | 1.96E-13 | 8.90E-13 | 19.54881401 | YPEL5 |
| 217984_at | 1.577152489 | 7.951973235 | 6.963033077 | 2.37E-09 | 6.59E-09 | 10.15571193 | RNASET2 |
| 203956_at | 1.576501168 | 7.645158615 | 13.68490537 | 1.70E-20 | 1.96E-19 | 35.89399499 | MORC2 |
| 218456_at | 1.574865658 | 6.804961942 | 7.684153681 | 1.31E-10 | 4.22E-10 | 13.04120365 | CAPRIN2 |
| 228504_at | -1.574698922 | 4.337171475 | -4.825837105 | 9.29E-06 | 1.76E-05 | 1.98325168 | SCN7A |
| 231843_at | 1.574593454 | 5.867807651 | 12.84627025 | 3.22E-19 | 3.09E-18 | 32.93551308 | DDX55 |
| 227345_at | 1.574164367 | 5.73867822 | 6.860877014 | 3.56E-09 | 9.70E-09 | 9.749250434 | TNFRSF10D |
| 210115_at | 1.574135958 | 5.280081693 | 9.813334064 | 2.73E-14 | 1.38E-13 | 21.52667705 | RPL39L |
| 203322_at | 1.573876085 | 6.881239029 | 13.69743519 | 1.63E-20 | 1.88E-19 | 35.93751567 | ADNP2 |
| 201972_at | 1.573477533 | 8.47943223 | 12.52281739 | 1.02E-18 | 9.21E-18 | 31.77058371 | ATP6V1A |
| 225524_at | 1.572970434 | 6.355397856 | 5.893208589 | 1.63E-07 | 3.73E-07 | 5.95640538 | ANTXR2 |
| 219569_s_at | 1.572786354 | 6.583819688 | 6.258246569 | 3.90E-08 | 9.48E-08 | 7.37230569 | SLC35G2 |
| 201201_at | 1.57235715 | 9.581326854 | 12.13220416 | 4.21E-18 | 3.49E-17 | 30.34630916 | CSTB |
| 218854_at | 1.571741967 | 6.599772628 | 6.121366769 | 6.69E-08 | 1.59E-07 | 6.838836129 | DSE |
| 223187_s_at | 1.571578892 | 7.841516838 | 12.15404022 | 3.89E-18 | 3.24E-17 | 30.42642822 | ORMDL1 |
| 212329_at | 1.571457066 | 7.35594811 | 15.83456905 | 1.37E-23 | 2.52E-22 | 43.06874988 | SCAP |
| 203318_s_at | 1.571244792 | 8.291509502 | 14.76170962 | 4.46E-22 | 6.53E-21 | 39.56094292 | ZNF148 |
| 201939_at | 1.571217785 | 5.310386857 | 5.684865297 | 3.66E-07 | 8.04E-07 | 5.15907214 | PLK2 |
| 208398_s_at | 1.570594278 | 6.437937425 | 8.356125151 | 8.81E-12 | 3.26E-11 | 15.73796574 | TBPL1 |
| 208819_at | 1.570490402 | 7.199630481 | 17.84808752 | 2.89E-26 | 8.08E-25 | 49.2749754 | RAB8A |
| 202580_x_at | 1.569281707 | 5.987099854 | 6.3984235 | 2.24E-08 | 5.60E-08 | 7.921411266 | FOXM1 |
| 225136_at | 1.569213158 | 7.416899395 | 11.13686973 | 1.69E-16 | 1.11E-15 | 26.63335888 | PLEKHA2 |
| 213452_at | 1.569185829 | 5.612190706 | 12.18760951 | 3.44E-18 | 2.89E-17 | 30.54948322 | ZNF184 |
| 212829_at | 1.568857062 | 6.667194499 | 10.24802275 | 5.04E-15 | 2.78E-14 | 23.22312901 | PIP4K2A |
| 232785_at | -1.568374636 | 3.873555981 | -13.74986816 | 1.36E-20 | 1.59E-19 | 36.11941663 | RGAG1 |
| 201057_s_at | 1.568308356 | 7.192342939 | 12.59312608 | 7.95E-19 | 7.25E-18 | 32.02492434 | GOLGB1 |
| 218395_at | 1.568071539 | 6.678472837 | 8.278124464 | 1.20E-11 | 4.39E-11 | 15.42521785 | ACTR6 |
| 209593_s_at | 1.567815815 | 7.062065604 | 12.85245639 | 3.15E-19 | 3.03E-18 | 32.95766396 | TOR1B |
| 226888_at | 1.567807904 | 6.225600953 | 12.92090127 | 2.47E-19 | 2.41E-18 | 33.20242277 | CSNK1G1 |
| 228299_at | 1.567237078 | 7.749458719 | 17.10615645 | 2.65E-25 | 6.39E-24 | 47.04371393 | KCTD20 |
| 219130_at | 1.566810851 | 6.448811216 | 9.298679782 | 2.06E-13 | 9.35E-13 | 19.49747888 | TRMT13 |
| 203819_s_at | 1.566646843 | 4.390585834 | 3.602746598 | 0.000623354 | 0.000974849 | -2.079195503 | IGF2BP3 |
| 201574_at | 1.565808158 | 7.833093329 | 11.88135155 | 1.06E-17 | 8.27E-17 | 29.42170859 | ETF1 |
| 227983_at | 1.565669269 | 6.514844378 | 10.38555157 | 2.96E-15 | 1.68E-14 | 23.75614711 | RILPL2 |
| 204071_s_at | 1.565570872 | 6.859020328 | 11.26683004 | 1.04E-16 | 7.04E-16 | 27.12476369 | TOPORS |
| 213911_s_at | 1.565560196 | 9.837203783 | 8.299513867 | 1.10E-11 | 4.05E-11 | 15.51099453 | H2AFZ |
| 209443_at | -1.56526239 | 5.265816094 | -14.79970212 | 3.94E-22 | 5.80E-21 | 39.6876284 | SERPINA5 |
| 201022_s_at | 1.564941933 | 9.87744774 | 13.04747768 | 1.58E-19 | 1.59E-18 | 33.65349313 | DSTN |
| 60471_at | 1.564435425 | 6.431942042 | 10.5628186 | 1.50E-15 | 8.80E-15 | 24.44038471 | RIN3 |
| 216698_x_at | -1.564392837 | 5.430560641 | -13.94164836 | 7.05E-21 | 8.69E-20 | 36.78174603 | OR7E12P |
| 225123_at | 1.56369483 | 5.736368854 | 6.762618492 | 5.27E-09 | 1.41E-08 | 9.359099875 | SESN3 |
| 235146_at | -1.56322908 | 4.295570109 | -9.156088308 | 3.63E-13 | 1.60E-12 | 18.93183311 | TMCC3 |
| 202109_at | 1.563098619 | 7.100091665 | 15.44066549 | 4.84E-23 | 8.25E-22 | 41.7975267 | ARFIP2 |
| 217874_at | -1.56305319 | 9.323272287 | -16.0181724 | 7.66E-24 | 1.46E-22 | 43.65475257 | SUCLG1 |
| 222451_s_at | 1.562664429 | 6.592711825 | 9.278617175 | 2.23E-13 | 1.01E-12 | 19.41797492 | ZDHHC9 |
| 219185_at | -1.562320989 | 6.230679355 | -13.08350226 | 1.39E-19 | 1.41E-18 | 33.78149866 | SIRT5 |
| 217978_s_at | 1.562149816 | 8.145678881 | 16.65342603 | 1.06E-24 | 2.34E-23 | 45.65068767 | UBE2Q1 |
| 202414_at | 1.562066398 | 6.792769926 | 16.84583097 | 5.85E-25 | 1.35E-23 | 46.24566638 | ERCC5 |
| 212367_at | 1.561900367 | 6.827067579 | 11.68458468 | 2.18E-17 | 1.64E-16 | 28.69111467 | FEM1B |
| 204971_at | 1.561461803 | 5.135370395 | 4.760642159 | 1.18E-05 | 2.21E-05 | 1.751210802 | CSTA |
| 223239_at | 1.56128747 | 6.370946584 | 9.654746134 | 5.08E-14 | 2.48E-13 | 20.90361469 | GSKIP |
| 231940_at | 1.561267844 | 6.734629644 | 9.612940667 | 5.98E-14 | 2.89E-13 | 20.73902495 | ZNF529 |
| 213350_at | 1.561198485 | 8.01147957 | 10.93939455 | 3.57E-16 | 2.26E-15 | 25.88302463 | RPS11 |
| 236166_at | -1.56093878 | 5.013886533 | -8.286643603 | 1.16E-11 | 4.25E-11 | 15.45938293 | LOC285147 |
| 218394_at | -1.560757684 | 5.371104938 | -16.10739804 | 5.78E-24 | 1.13E-22 | 43.93804394 | ROGDI |
| 237351_at | -1.560601912 | 3.664835178 | -11.85319982 | 1.17E-17 | 9.12E-17 | 29.31746762 | LOC284825 |
| 201798_s_at | 1.560271091 | 6.401190518 | 4.436205109 | 3.77E-05 | 6.72E-05 | 0.619435655 | MYOF |
| 213594_x_at | 1.560135069 | 8.099551978 | 10.96992245 | 3.18E-16 | 2.02E-15 | 25.99930308 | SRSF10 |
| 222825_at | 1.560110554 | 5.164628175 | 9.513618711 | 8.84E-14 | 4.19E-13 | 20.34743684 | OTUD6B |
| 208668_x_at | 1.560076267 | 11.23743539 | 14.53003976 | 9.65E-22 | 1.35E-20 | 38.78448536 | HMGN2 |
| 218352_at | 1.559778386 | 5.40574425 | 8.65681479 | 2.64E-12 | 1.05E-11 | 16.94185018 | RCBTB1 |
| 218146_at | 1.559640788 | 8.483186912 | 16.18024713 | 4.60E-24 | 9.11E-23 | 44.16862093 | GLT8D1 |
| 209651_at | 1.559381974 | 6.46825478 | 5.752881976 | 2.81E-07 | 6.26E-07 | 5.418411532 | TGFB1I1 |
| 202519_at | -1.559117483 | 6.762692768 | -8.921251728 | 9.22E-13 | 3.85E-12 | 17.99749494 | MLXIP |
| 202408_s_at | 1.559012911 | 7.257530957 | 14.97118992 | 2.24E-22 | 3.41E-21 | 40.25718338 | PRPF31 |
| 227772_at | 1.559003694 | 6.511130798 | 12.45239853 | 1.32E-18 | 1.17E-17 | 31.51522326 | LATS1 |
| 222992_s_at | -1.558734833 | 10.62132209 | -11.94222895 | 8.45E-18 | 6.71E-17 | 29.64679827 | NDUFB9 |
| 243606_at | -1.558170539 | 6.1901251 | -10.87956796 | 4.48E-16 | 2.80E-15 | 25.65485263 | NXPE3 |
| 213334_x_at | 1.557669844 | 6.423880527 | 8.10679724 | 2.39E-11 | 8.43E-11 | 14.73782957 | HAUS7 |
| 1557684_at | 1.557533132 | 5.28592232 | 13.09458812 | 1.33E-19 | 1.36E-18 | 33.82085666 | ZNF286A |
| 212638_s_at | -1.557170051 | 9.0740183 | -9.10692745 | 4.41E-13 | 1.93E-12 | 18.73651143 | WWP1 |
| 242972_at | 1.556430036 | 6.753424709 | 11.97620345 | 7.46E-18 | 5.98E-17 | 29.77222045 | HCG18 |
| 202306_at | 1.55620793 | 8.338799159 | 16.04447812 | 7.05E-24 | 1.35E-22 | 43.73837416 | POLR2G |
| 227195_at | 1.555945369 | 5.535463646 | 5.469646311 | 8.36E-07 | 1.77E-06 | 4.345173062 | ZNF503 |
| 203288_at | 1.55551033 | 7.317584029 | 6.791336892 | 4.70E-09 | 1.27E-08 | 9.473043878 | KIAA0355 |
| 226763_at | 1.555382717 | 6.155564644 | 6.892295633 | 3.14E-09 | 8.61E-09 | 9.874173552 | SESTD1 |
| 242585_at | 1.5552033 | 5.142511501 | 10.28094719 | 4.43E-15 | 2.47E-14 | 23.35090262 | CKMT2-AS1 |
| 203542_s_at | -1.554823394 | 6.387717992 | -6.286623982 | 3.49E-08 | 8.52E-08 | 7.483248817 | KLF9 |
| 224736_at | 1.55462749 | 8.802247035 | 12.40067916 | 1.59E-18 | 1.39E-17 | 31.3272779 | CCAR1 |
| 200847_s_at | 1.55440374 | 10.09515425 | 12.56391477 | 8.83E-19 | 8.02E-18 | 31.91932824 | SARAF |
| 205070_at | 1.553910102 | 6.853104179 | 12.47452927 | 1.22E-18 | 1.08E-17 | 31.59554305 | ING3 |
| 212178_s_at | 1.552464415 | 6.434792178 | 12.99995223 | 1.86E-19 | 1.86E-18 | 33.48436913 | POM121C |
| 204228_at | 1.552174913 | 7.58855708 | 13.03413501 | 1.65E-19 | 1.66E-18 | 33.60604091 | PPIH |
| 1566908_at | -1.55215717 | 5.217510741 | -17.62570133 | 5.58E-26 | 1.49E-24 | 48.61280983 | C9orf173 |
| 208912_s_at | 1.552033273 | 7.717635816 | 16.30387089 | 3.12E-24 | 6.38E-23 | 44.55843282 | CNP |
| 1562826_at | -1.55171989 | 4.258980994 | -15.80016258 | 1.53E-23 | 2.79E-22 | 42.95847538 | LOC101927049 |
| 228063_s_at | 1.551605608 | 5.39615217 | 8.861214573 | 1.17E-12 | 4.82E-12 | 17.75812639 | NAP1L5 |
| 232090_at | 1.551341458 | 4.74370858 | 4.121443689 | 0.000112483 | 0.000190492 | -0.438092423 | DNM3OS |
| 218343_s_at | 1.550623699 | 7.057738076 | 10.33589355 | 3.59E-15 | 2.02E-14 | 23.56390327 | GTF3C3 |
| 231809_x_at | 1.550613645 | 7.311962836 | 13.72898409 | 1.46E-20 | 1.70E-19 | 36.04700757 | PDCD7 |
| 224103_at | -1.550359334 | 3.677682635 | -12.69732976 | 5.47E-19 | 5.11E-18 | 32.40073618 | LOC100132661 |
| 225312_at | 1.550030698 | 8.868910989 | 12.45981402 | 1.28E-18 | 1.14E-17 | 31.54214336 | COMMD6 |
| 219286_s_at | 1.549813657 | 7.75027751 | 13.87333451 | 8.91E-21 | 1.08E-19 | 36.54635784 | RBM15 |
| 225708_at | 1.549589266 | 5.195003014 | 13.86914558 | 9.04E-21 | 1.09E-19 | 36.53190467 | MED29 |
| 208928_at | 1.548786375 | 6.730454644 | 12.0705533 | 5.28E-18 | 4.33E-17 | 30.11978821 | POR |
| 203909_at | 1.548579381 | 6.949396474 | 11.74808218 | 1.73E-17 | 1.32E-16 | 28.92738927 | SLC9A6 |
| 212167_s_at | 1.548083438 | 6.831466769 | 13.92791675 | 7.39E-21 | 9.07E-20 | 36.73447915 | SMARCB1 |
| 224320_s_at | 1.547780679 | 5.806369684 | 7.173041605 | 1.02E-09 | 2.95E-09 | 10.99356686 | MCM8 |
| 201841_s_at | -1.547628842 | 10.77247998 | -9.535397095 | 8.11E-14 | 3.86E-13 | 20.43336624 | HSPB1 |
| 225068_at | 1.545603615 | 6.821348502 | 11.19668796 | 1.35E-16 | 9.02E-16 | 26.85978195 | KLHL12 |
| 228332_s_at | 1.545534717 | 9.724194445 | 15.97225217 | 8.86E-24 | 1.68E-22 | 43.50857709 | C11orf31 |
| 217736_s_at | 1.545233066 | 7.864348675 | 12.62266384 | 7.15E-19 | 6.56E-18 | 32.13159147 | EIF2AK1 |
| 218358_at | 1.544669421 | 8.154757881 | 12.82064889 | 3.52E-19 | 3.36E-18 | 32.84371832 | CRELD2 |
| 237329_at | -1.544443746 | 3.498360388 | -12.71722617 | 5.10E-19 | 4.77E-18 | 32.47233726 | RBMS3-AS3 |
| 209332_s_at | 1.544425626 | 8.300718855 | 15.27741267 | 8.22E-23 | 1.34E-21 | 41.26502453 | MAX |
| 213637_at | 1.544171484 | 5.471701135 | 14.76291452 | 4.45E-22 | 6.51E-21 | 39.56496345 | DDX52 |
| 227713_at | 1.543995885 | 6.916904921 | 8.410971521 | 7.07E-12 | 2.66E-11 | 15.95777912 | KATNAL1 |
| 223236_at | 1.543941418 | 7.577183942 | 13.90638048 | 7.95E-21 | 9.72E-20 | 36.66029847 | NSRP1 |
| 1552287_s_at | 1.543624081 | 5.804891322 | 8.720170724 | 2.05E-12 | 8.21E-12 | 17.19507066 | AFG3L1P |
| 209486_at | 1.543553902 | 7.206015487 | 13.5915495 | 2.35E-20 | 2.67E-19 | 35.56910339 | UTP3 |
| 207081_s_at | 1.542859266 | 6.049616698 | 10.20270625 | 6.00E-15 | 3.28E-14 | 23.04709275 | PI4KA |
| 201833_at | 1.542487254 | 8.222630563 | 9.117251843 | 4.23E-13 | 1.85E-12 | 18.77754395 | HDAC2 |
| 202336_s_at | -1.542242439 | 8.362185274 | -4.288645678 | 6.32E-05 | 0.000109894 | 0.118371177 | PAM |
| 221677_s_at | 1.542011556 | 5.867665787 | 10.3031394 | 4.07E-15 | 2.28E-14 | 23.4369668 | DONSON |
| 214677_x_at | 1.541472038 | 6.773511848 | 2.464136876 | 0.016488399 | 0.021995706 | -5.124363902 | IGLC1 |
| 203050_at | 1.541468062 | 7.706913008 | 16.23757375 | 3.84E-24 | 7.71E-23 | 44.34961412 | TP53BP1 |
| 208921_s_at | 1.541242903 | 8.168770965 | 10.03596106 | 1.15E-14 | 6.04E-14 | 22.39767961 | SRI |
| 235863_at | -1.54047818 | 5.780350986 | -12.69424475 | 5.53E-19 | 5.16E-18 | 32.38962972 | JSRP1 |
| 227230_s_at | 1.540145214 | 4.760488232 | 4.753368658 | 1.21E-05 | 2.27E-05 | 1.725414243 | KIAA1211 |
| 200594_x_at | 1.539885584 | 10.18938201 | 16.15798794 | 4.93E-24 | 9.70E-23 | 44.0982361 | HNRNPU |
| 218894_s_at | 1.539716883 | 4.988905934 | 10.72084631 | 8.19E-16 | 4.96E-15 | 25.04762267 | MAGOHB |
| 229002_at | 1.539639654 | 6.686657876 | 9.842073314 | 2.44E-14 | 1.24E-13 | 21.63936111 | FAM69B |
| 229802_at | 1.539045349 | 4.200040587 | 3.895514079 | 0.000240599 | 0.00039325 | -1.169525292 | WISP1 |
| 205602_x_at | -1.538910807 | 4.652189573 | -20.01418629 | 6.32E-29 | 2.76E-27 | 55.44118764 | PSG7 |
| 200701_at | 1.538820895 | 8.077784214 | 8.347817739 | 9.10E-12 | 3.37E-11 | 15.70466404 | NPC2 |
| 213701_at | 1.538781561 | 6.714117016 | 9.22302158 | 2.78E-13 | 1.24E-12 | 19.19751843 | C12orf29 |
| 212653_s_at | 1.538105142 | 7.214832383 | 10.2253434 | 5.50E-15 | 3.02E-14 | 23.13505364 | EHBP1 |
| 204142_at | 1.53802805 | 7.156650488 | 7.965973384 | 4.22E-11 | 1.45E-10 | 14.17253547 | ENOSF1 |
| 205241_at | 1.53797516 | 6.737706703 | 11.68027515 | 2.22E-17 | 1.66E-16 | 28.6750614 | SCO2 |
| 205756_s_at | -1.537782269 | 6.028773347 | -6.906059569 | 2.97E-09 | 8.18E-09 | 9.9289248 | F8 |
| 218604_at | 1.537613732 | 7.563342937 | 16.61758411 | 1.18E-24 | 2.59E-23 | 45.53936613 | LEMD3 |
| 218679_s_at | 1.53741587 | 9.008577327 | 11.10558054 | 1.90E-16 | 1.25E-15 | 26.51476222 | VPS28 |
| 201477_s_at | 1.536866433 | 8.104999506 | 8.754260183 | 1.79E-12 | 7.20E-12 | 17.33124499 | RRM1 |
| 202007_at | 1.536831413 | 6.523549125 | 3.984123327 | 0.000179016 | 0.00029708 | -0.885574548 | NID1 |
| 203229_s_at | 1.536499605 | 7.688579227 | 14.42983391 | 1.35E-21 | 1.84E-20 | 38.44652721 | CLK2 |
| 227186_s_at | -1.536497814 | 8.610612562 | -14.7291152 | 4.97E-22 | 7.22E-21 | 39.45211169 | MRPL41 |
| 212140_at | 1.535750638 | 7.883455465 | 13.34661733 | 5.51E-20 | 5.96E-19 | 34.71140559 | PDS5A |
| 212922_s_at | -1.534794993 | 7.729374104 | -7.707596029 | 1.19E-10 | 3.85E-10 | 13.13528651 | SMYD2 |
| 219981_x_at | 1.534728892 | 7.192899045 | 13.94798086 | 6.90E-21 | 8.51E-20 | 36.80353562 | ZNF587 |
| 237255_at | -1.534471027 | 4.645929188 | -14.70748897 | 5.34E-22 | 7.72E-21 | 39.37982848 | LINC01272 |
| 229622_at | 1.53428339 | 7.002338763 | 2.629497021 | 0.010743078 | 0.014632452 | -4.737060691 | FAM132B |
| 214323_s_at | 1.534247272 | 8.442330659 | 10.09529232 | 9.10E-15 | 4.86E-14 | 22.62905179 | UPF3A |
| 238424_at | -1.534202436 | 5.608137308 | -16.46604519 | 1.89E-24 | 4.00E-23 | 45.06700319 | ADAL |
| 1563528_at | -1.534086064 | 3.659480886 | -16.41066923 | 2.24E-24 | 4.69E-23 | 44.89370318 | RAPGEF4-AS1 |
| 227300_at | 1.534050346 | 5.389021058 | 4.577867574 | 2.28E-05 | 4.16E-05 | 1.108736855 | TMEM119 |
| 209158_s_at | 1.533905434 | 6.827063892 | 14.06513667 | 4.63E-21 | 5.85E-20 | 37.2057338 | CYTH2 |
| 225284_at | 1.533100644 | 6.916921948 | 10.07488647 | 9.85E-15 | 5.24E-14 | 22.5495125 | DNAJC3 |
| 212733_at | 1.533044217 | 6.277245892 | 9.900767612 | 1.94E-14 | 9.96E-14 | 21.86927375 | KIAA0226 |
| 225150_s_at | 1.532959541 | 5.436259501 | 9.015789798 | 6.33E-13 | 2.71E-12 | 18.37402012 | RTKN |
| 202734_at | -1.532216422 | 7.000121712 | -9.798707026 | 2.89E-14 | 1.46E-13 | 21.46929853 | TRIP10 |
| 222411_s_at | 1.532080656 | 8.975986302 | 9.171746522 | 3.41E-13 | 1.51E-12 | 18.99401289 | SSR3 |
| 226215_s_at | 1.531941452 | 7.046614363 | 12.35086895 | 1.90E-18 | 1.65E-17 | 31.14595477 | KDM2B |
| 218715_at | 1.531372372 | 7.578100136 | 15.06878878 | 1.62E-22 | 2.52E-21 | 40.57967925 | UTP6 |
| 202745_at | 1.53121545 | 6.514339173 | 11.13354355 | 1.71E-16 | 1.13E-15 | 26.62075676 | USP8 |
| 213532_at | 1.530983841 | 7.158666946 | 11.32590055 | 8.31E-17 | 5.72E-16 | 27.34748168 | ADAM17 |
| 1561218_s_at | -1.530819079 | 3.996143379 | -20.29749604 | 2.94E-29 | 1.36E-27 | 56.21135644 | LOC728099 |
| 203151_at | 1.530642966 | 6.409309455 | 10.34545058 | 3.46E-15 | 1.95E-14 | 23.60092091 | MAP1A |
| 1557091_at | -1.530053027 | 5.756398615 | -14.13615375 | 3.64E-21 | 4.65E-20 | 37.44868287 | MAMSTR |
| 218773_s_at | -1.529712134 | 8.293444224 | -8.700238842 | 2.22E-12 | 8.85E-12 | 17.11542603 | MSRB2 |
| 211464_x_at | 1.528984888 | 7.23838994 | 17.37409253 | 1.18E-25 | 2.99E-24 | 47.85681477 | CASP6 |
| 212354_at | 1.528718744 | 6.135744896 | 4.426323206 | 3.90E-05 | 6.95E-05 | 0.585599716 | SULF1 |
| 225256_at | 1.528183315 | 6.960725082 | 10.6695852 | 9.96E-16 | 5.96E-15 | 24.85093422 | CTC-444N24.11 |
| 225684_at | 1.527668647 | 7.578519319 | 8.22771062 | 1.47E-11 | 5.32E-11 | 15.2230064 | SKA2 |
| 201761_at | 1.527629678 | 7.710532489 | 6.67750433 | 7.40E-09 | 1.95E-08 | 9.021847929 | MTHFD2 |
| 223060_at | 1.526759215 | 7.660720247 | 12.72334585 | 4.99E-19 | 4.67E-18 | 32.49435006 | C14orf119 |
| 227158_at | 1.526447629 | 6.287381643 | 12.41910137 | 1.49E-18 | 1.31E-17 | 31.39426151 | DTD2 |
| 229285_at | 1.526316488 | 5.099599928 | 11.10813804 | 1.88E-16 | 1.23E-15 | 26.52446016 | RNASEL |
| 240729_at | -1.525907255 | 3.94654231 | -21.08834178 | 3.62E-30 | 2.00E-28 | 58.31927921 | ERICH6 |
| 203577_at | 1.5258208 | 5.890945876 | 9.988865765 | 1.38E-14 | 7.19E-14 | 22.2137937 | GTF2H4 |
| 212153_at | 1.525422888 | 8.258894575 | 12.50175652 | 1.10E-18 | 9.88E-18 | 31.6942756 | POGZ |
| 214862_x_at | 1.525415154 | 4.970833679 | 7.688247369 | 1.29E-10 | 4.15E-10 | 13.05763259 | RP11-119F7.5 |
| 220547_s_at | 1.525145079 | 7.499129386 | 10.22531208 | 5.50E-15 | 3.02E-14 | 23.13493199 | FAM35A |
| 201517_at | 1.524882725 | 7.751037306 | 12.54803912 | 9.35E-19 | 8.46E-18 | 31.86189431 | NCBP2 |
| 202726_at | 1.524812248 | 6.713342348 | 12.32580186 | 2.08E-18 | 1.80E-17 | 31.05458668 | LIG1 |
| 1559471_s_at | -1.524481959 | 4.871543342 | -19.5341904 | 2.35E-28 | 9.28E-27 | 54.11771903 | D21S2088E |
| 211684_s_at | 1.524390263 | 9.12424766 | 10.69759146 | 8.95E-16 | 5.39E-15 | 24.95842855 | DYNC1I2 |
| 228009_x_at | 1.524213294 | 7.589591343 | 14.9882423 | 2.11E-22 | 3.23E-21 | 40.31361597 | ZNRD1 |
| 219485_s_at | 1.522948767 | 7.387958177 | 9.228294833 | 2.73E-13 | 1.22E-12 | 19.21843765 | PSMD10 |
| 1557841_at | -1.521623853 | 4.574091812 | -17.71296106 | 4.31E-26 | 1.18E-24 | 48.8733004 | LOC101926915 |
| 226344_at | 1.521112329 | 4.97976521 | 5.013699994 | 4.65E-06 | 9.13E-06 | 2.659785715 | ZMAT1 |
| 205740_s_at | 1.521022896 | 7.261663072 | 13.48897348 | 3.36E-20 | 3.73E-19 | 35.21083868 | RBM42 |
| 228765_at | -1.520915254 | 6.645512863 | -11.07866324 | 2.11E-16 | 1.37E-15 | 26.41264834 | GTF2IRD2 |
| 225929_s_at | 1.520896684 | 7.266465446 | 8.644383972 | 2.78E-12 | 1.09E-11 | 16.89214656 | RNF213 |
| 227034_at | 1.520809685 | 6.300895677 | 7.879793199 | 5.96E-11 | 2.00E-10 | 13.82654178 | SOWAHC |
| 218452_at | 1.520440091 | 6.503427192 | 15.97700108 | 8.73E-24 | 1.66E-22 | 43.52370601 | SMARCAL1 |
| 220694_at | 1.520272892 | 6.164287754 | 9.069898527 | 5.11E-13 | 2.21E-12 | 18.58929255 | ASAP1-IT1 |
| 202180_s_at | 1.519781811 | 6.719937105 | 8.577812121 | 3.63E-12 | 1.41E-11 | 16.6258576 | MVP |
| 222333_at | -1.519032236 | 7.075164594 | -18.95457651 | 1.19E-27 | 4.26E-26 | 52.48762224 | ALS2CL |
| 226710_at | 1.518848465 | 7.018650174 | 9.09830553 | 4.56E-13 | 1.99E-12 | 18.70224005 | C8orf82 |
| 205062_x_at | 1.518781079 | 5.797904766 | 13.67136073 | 1.78E-20 | 2.05E-19 | 35.84692691 | ARID4A |
| 220079_s_at | 1.51875356 | 7.951593997 | 18.16767466 | 1.13E-26 | 3.45E-25 | 50.21678174 | USP48 |
| 221510_s_at | 1.518722488 | 6.852536549 | 9.494942982 | 9.51E-14 | 4.49E-13 | 20.27372049 | GLS |
| 221699_s_at | 1.518577336 | 7.721121984 | 11.80081349 | 1.42E-17 | 1.10E-16 | 29.12323501 | DDX50 |
| 213328_at | 1.518071891 | 7.081699844 | 13.18445182 | 9.73E-20 | 1.01E-18 | 34.1393207 | NEK1 |
| 201568_at | -1.518053718 | 10.46780356 | -14.46616432 | 1.19E-21 | 1.65E-20 | 38.56920412 | UQCRQ |
| 215001_s_at | -1.518024905 | 10.031753 | -9.973097174 | 1.46E-14 | 7.62E-14 | 22.1521795 | GLUL |
| 235607_at | 1.517890431 | 4.528159044 | 8.223568377 | 1.50E-11 | 5.41E-11 | 15.20638941 | LOC101928963 |
| 220116_at | -1.517036559 | 4.012789764 | -8.798647267 | 1.50E-12 | 6.10E-12 | 17.50847281 | KCNN2 |
| 224562_at | 1.51652812 | 8.156108437 | 16.78164642 | 7.12E-25 | 1.62E-23 | 46.04767532 | WASF2 |
| 229290_at | 1.516410943 | 5.179165914 | 3.049840493 | 0.003352359 | 0.004836142 | -3.664302307 | DAPL1 |
| 1569696_at | -1.516326945 | 5.648642733 | -18.04219077 | 1.63E-26 | 4.82E-25 | 49.84835352 | CSMD2-AS1 |
| 204028_s_at | 1.515996876 | 8.723790147 | 10.32529827 | 3.74E-15 | 2.10E-14 | 23.52285353 | RABGAP1 |
| 202421_at | 1.515889699 | 6.458981621 | 5.578640397 | 5.51E-07 | 1.19E-06 | 4.75604032 | IGSF3 |
| 229431_at | 1.515319952 | 5.00088501 | 8.242481158 | 1.39E-11 | 5.04E-11 | 15.28225697 | RFXAP |
| 209656_s_at | 1.515260002 | 9.581790678 | 3.733800236 | 0.000409047 | 0.000652709 | -1.677566749 | TMEM47 |
| 210589_s_at | 1.514594105 | 6.796408834 | 14.01772709 | 5.44E-21 | 6.82E-20 | 37.04318717 | GBAP1 |
| 222427_s_at | 1.514331649 | 8.721174329 | 9.076525195 | 4.97E-13 | 2.16E-12 | 18.61564486 | LARS |
| 217893_s_at | 1.513986045 | 7.184074115 | 11.6911784 | 2.13E-17 | 1.60E-16 | 28.7156724 | AKIRIN1 |
| 214440_at | 1.512790327 | 4.846332202 | 6.949832842 | 2.50E-09 | 6.92E-09 | 10.10314595 | NAT1 |
| 201119_s_at | -1.512607593 | 10.31893864 | -15.71002801 | 2.04E-23 | 3.66E-22 | 42.66889845 | COX8A |
| 225145_at | 1.511886513 | 5.911903968 | 13.18775819 | 9.61E-20 | 1.00E-18 | 34.15101837 | NCOA5 |
| 221475_s_at | 1.511640353 | 11.76246715 | 17.38268344 | 1.15E-25 | 2.92E-24 | 47.88274761 | RPL15 |
| 209222_s_at | 1.511600698 | 6.608535045 | 10.57203978 | 1.45E-15 | 8.51E-15 | 24.47588975 | OSBPL2 |
| 211023_at | -1.511413152 | 9.578633115 | -14.44321493 | 1.29E-21 | 1.77E-20 | 38.49173045 | PDHB |
| 225904_at | 1.511337708 | 6.23438253 | 6.726880571 | 6.08E-09 | 1.62E-08 | 9.217410066 | CCSAP |
| 1564336_at | -1.511334969 | 4.84066462 | -19.54227508 | 2.30E-28 | 9.11E-27 | 54.14020697 | AC009502.4 |
| 226195_at | 1.510599001 | 6.70374728 | 16.17826003 | 4.63E-24 | 9.15E-23 | 44.16234005 | IFT43 |
| 241359_at | 1.510161834 | 5.614856234 | 7.880010401 | 5.95E-11 | 2.00E-10 | 13.8274138 | TLCD2 |
| 225037_at | 1.510072383 | 6.558014613 | 12.35652745 | 1.87E-18 | 1.62E-17 | 31.16656887 | SLC35C2 |
| 202366_at | -1.510022418 | 6.069380084 | -18.06284801 | 1.54E-26 | 4.56E-25 | 49.90912547 | ACADS |
| 222122_s_at | 1.508270302 | 7.366656232 | 12.2475993 | 2.77E-18 | 2.35E-17 | 30.76904114 | THOC2 |
| 1568649_at | -1.508156869 | 6.394682934 | -20.28434878 | 3.04E-29 | 1.40E-27 | 56.17579405 | RP11-540O11.1 |
| 204638_at | 1.507970332 | 6.192573581 | 4.187526273 | 8.97E-05 | 0.000153336 | -0.219646689 | ACP5 |
| 227980_at | 1.507228788 | 6.986269341 | 14.9752106 | 2.21E-22 | 3.37E-21 | 40.27049261 | ZNF322 |
| 218206_x_at | 1.50667856 | 7.746056767 | 15.10236381 | 1.45E-22 | 2.28E-21 | 40.69034435 | SCAND1 |
| 213603_s_at | 1.506134976 | 7.032646557 | 6.008047057 | 1.04E-07 | 2.43E-07 | 6.399433504 | RAC2 |
| 201321_s_at | 1.505089625 | 7.110242425 | 13.83859701 | 1.00E-20 | 1.20E-19 | 36.42643429 | SMARCC2 |
| 200985_s_at | -1.50495471 | 9.007938691 | -7.516194658 | 2.57E-10 | 7.99E-10 | 12.36741396 | CD59 |
| 223049_at | 1.504677848 | 7.209719178 | 14.27917554 | 2.24E-21 | 2.94E-20 | 37.93600535 | GRB2 |
| 224836_at | -1.504469683 | 6.939289173 | -9.753357246 | 3.45E-14 | 1.72E-13 | 21.29128625 | TP53INP2 |
| 238623_at | 1.504293375 | 4.219154213 | 5.400105929 | 1.09E-06 | 2.28E-06 | 4.084525921 | RP3-428L16.2 |
| 203920_at | 1.503788842 | 5.542139447 | 5.843614003 | 1.98E-07 | 4.48E-07 | 5.765828122 | NR1H3 |
| 206243_at | -1.503676961 | 4.306834622 | -9.003019525 | 6.66E-13 | 2.83E-12 | 18.32318835 | TIMP4 |
| 218951_s_at | 1.503605161 | 7.055664039 | 7.82070856 | 7.56E-11 | 2.51E-10 | 13.58933465 | PLCXD1 |
| 214235_at | 1.503418782 | 5.093401675 | 4.146753571 | 0.000103164 | 0.000175245 | -0.354660226 | CYP3A5 |
| 234469_at | -1.503373075 | 4.024826149 | -11.67843446 | 2.23E-17 | 1.67E-16 | 28.66820403 | OR51B4 |
| 220840_s_at | 1.502518968 | 4.116816787 | 7.396762556 | 4.15E-10 | 1.26E-09 | 11.88873801 | C1orf112 |
| 226029_at | 1.502439089 | 5.189424484 | 8.677382968 | 2.44E-12 | 9.67E-12 | 17.02407602 | VANGL2 |
| 205361_s_at | 1.5014543 | 7.851548677 | 8.94178162 | 8.49E-13 | 3.56E-12 | 18.07930349 | PFDN4 |
| 211063_s_at | 1.501083731 | 7.138485076 | 8.866173986 | 1.15E-12 | 4.73E-12 | 17.77790677 | NCK1 |
| 1552400_a_at | -1.500965149 | 4.17064285 | -19.19334468 | 6.07E-28 | 2.27E-26 | 53.16342575 | C15orf27 |
| 224364_s_at | 1.500527861 | 7.824646468 | 8.989406821 | 7.03E-13 | 2.98E-12 | 18.26899298 | PPIL3 |
| 212137_at | 1.500500176 | 9.370570979 | 15.43408957 | 4.95E-23 | 8.40E-22 | 41.77614143 | LARP1 |
| 200639_s_at | 1.500358986 | 9.245720888 | 12.34797453 | 1.92E-18 | 1.67E-17 | 31.13540878 | YWHAZ |
| 200704_at | 1.500085219 | 7.601012065 | 5.380885256 | 1.17E-06 | 2.44E-06 | 4.012697294 | LITAF |
