# Supplementary material for: Integration of ubiquitination-related genes in predictive signatures for prognosis and immunotherapy response in sarcoma
Source: Front Oncol. 2024 Oct 14;14:1446522. doi: 10.3389/fonc.2024.1446522 (PMC11513255; doi:10.3389/fonc.2024.1446522)
Supplement: Supplementary file 1 [file DataSheet1.zip › Supplementary Table 2.docx]

**Supplementary Table 2. Ubiquitination related genes**

| Gene Symbol | Description | Category | Gifts | GC Id | Relevance score | GeneCards Link |
| --- | --- | --- | --- | --- | --- | --- |
| RPS27A | Ribosomal Protein S27a | Protein Coding | 49 | GC02P055231 | 38.09315872 | https://www.genecards.org/cgi-bin/carddisp.pl?gene=RPS27A |
| PRKN | Parkin RBR E3 Ubiquitin Protein Ligase | Protein Coding | 55 | GC06M161348 | 35.76257324 | https://www.genecards.org/cgi-bin/carddisp.pl?gene=PRKN |
| UBC | Ubiquitin C | Protein Coding | 50 | GC12M124911 | 34.5500946 | https://www.genecards.org/cgi-bin/carddisp.pl?gene=UBC |
| UBE2D1 | Ubiquitin Conjugating Enzyme E2 D1 | Protein Coding | 51 | GC10P058334 | 34.14750671 | https://www.genecards.org/cgi-bin/carddisp.pl?gene=UBE2D1 |
| UBE2D3 | Ubiquitin Conjugating Enzyme E2 D3 | Protein Coding | 52 | GC04M102794 | 33.50337982 | https://www.genecards.org/cgi-bin/carddisp.pl?gene=UBE2D3 |
| UBE2D2 | Ubiquitin Conjugating Enzyme E2 D2 | Protein Coding | 50 | GC05P139526 | 32.33262634 | https://www.genecards.org/cgi-bin/carddisp.pl?gene=UBE2D2 |
| VCP | Valosin Containing Protein | Protein Coding | 57 | GC09M035056 | 31.85129166 | https://www.genecards.org/cgi-bin/carddisp.pl?gene=VCP |
| ITCH | Itchy E3 Ubiquitin Protein Ligase | Protein Coding | 54 | GC20P034363 | 31.44257736 | https://www.genecards.org/cgi-bin/carddisp.pl?gene=ITCH |
| UBE2L3 | Ubiquitin Conjugating Enzyme E2 L3 | Protein Coding | 52 | GC22P021549 | 30.86452103 | https://www.genecards.org/cgi-bin/carddisp.pl?gene=UBE2L3 |
| UBE2N | Ubiquitin Conjugating Enzyme E2 N | Protein Coding | 54 | GC12M093406 | 30.24916267 | https://www.genecards.org/cgi-bin/carddisp.pl?gene=UBE2N |
| USP7 | Ubiquitin Specific Peptidase 7 | Protein Coding | 56 | GC16M008892 | 29.47817421 | https://www.genecards.org/cgi-bin/carddisp.pl?gene=USP7 |
| RBX1 | Ring-Box 1 | Protein Coding | 50 | GC22P040951 | 29.05633545 | https://www.genecards.org/cgi-bin/carddisp.pl?gene=RBX1 |
| UBE4B | Ubiquitination Factor E4B | Protein Coding | 48 | GC01P010032 | 28.44376755 | https://www.genecards.org/cgi-bin/carddisp.pl?gene=UBE4B |
| MDM2 | MDM2 Proto-Oncogene | Protein Coding | 59 | GC12P068808 | 28.4077549 | https://www.genecards.org/cgi-bin/carddisp.pl?gene=MDM2 |
| STUB1 | STIP1 Homology And U-Box Containing Protein 1 | Protein Coding | 53 | GC16P019817 | 27.81255913 | https://www.genecards.org/cgi-bin/carddisp.pl?gene=STUB1 |
| UBA1 | Ubiquitin Like Modifier Activating Enzyme 1 | Protein Coding | 56 | GC0XP047190 | 26.50860214 | https://www.genecards.org/cgi-bin/carddisp.pl?gene=UBA1 |
| NEDD4 | NEDD4 E3 Ubiquitin Protein Ligase | Protein Coding | 54 | GC15M055826 | 26.43677902 | https://www.genecards.org/cgi-bin/carddisp.pl?gene=NEDD4 |
| UBE2K | Ubiquitin Conjugating Enzyme E2 K | Protein Coding | 48 | GC04P039702 | 26.21509171 | https://www.genecards.org/cgi-bin/carddisp.pl?gene=UBE2K |
| UBE2C | Ubiquitin Conjugating Enzyme E2 C | Protein Coding | 52 | GC20P045812 | 26.11197472 | https://www.genecards.org/cgi-bin/carddisp.pl?gene=UBE2C |
| UBE4A | Ubiquitination Factor E4A | Protein Coding | 47 | GC11P118359 | 26.06382942 | https://www.genecards.org/cgi-bin/carddisp.pl?gene=UBE4A |
| UBE3A | Ubiquitin Protein Ligase E3A | Protein Coding | 53 | GC15M025333 | 25.86598969 | https://www.genecards.org/cgi-bin/carddisp.pl?gene=UBE3A |
| UBE2E1 | Ubiquitin Conjugating Enzyme E2 E1 | Protein Coding | 48 | GC03P023805 | 25.66685104 | https://www.genecards.org/cgi-bin/carddisp.pl?gene=UBE2E1 |
| UCHL1 | Ubiquitin C-Terminal Hydrolase L1 | Protein Coding | 59 | GC04P041256 | 25.66189575 | https://www.genecards.org/cgi-bin/carddisp.pl?gene=UCHL1 |
| CDC34 | Cell Division Cycle 34, Ubiqiutin Conjugating Enzyme | Protein Coding | 51 | GC19P005103 | 25.60669899 | https://www.genecards.org/cgi-bin/carddisp.pl?gene=CDC34 |
| NEDD4L | NEDD4 Like E3 Ubiquitin Protein Ligase | Protein Coding | 52 | GC18P058044 | 24.53695679 | https://www.genecards.org/cgi-bin/carddisp.pl?gene=NEDD4L |
| UBE2E3 | Ubiquitin Conjugating Enzyme E2 E3 | Protein Coding | 48 | GC02P180967 | 24.4715271 | https://www.genecards.org/cgi-bin/carddisp.pl?gene=UBE2E3 |
| UBE2I | Ubiquitin Conjugating Enzyme E2 I | Protein Coding | 55 | GC16P019824 | 24.35238457 | https://www.genecards.org/cgi-bin/carddisp.pl?gene=UBE2I |
| SQSTM1 | Sequestosome 1 | Protein Coding | 56 | GC05P179806 | 24.31902504 | https://www.genecards.org/cgi-bin/carddisp.pl?gene=SQSTM1 |
| HUWE1 | HECT, UBA And WWE Domain Containing E3 Ubiquitin Protein Ligase 1 | Protein Coding | 52 | GC0XM053532 | 24.04709053 | https://www.genecards.org/cgi-bin/carddisp.pl?gene=HUWE1 |
| UBB | Ubiquitin B | Protein Coding | 51 | GC17P016380 | 23.91206932 | https://www.genecards.org/cgi-bin/carddisp.pl?gene=UBB |
| USP5 | Ubiquitin Specific Peptidase 5 | Protein Coding | 51 | GC12P026349 | 23.77376938 | https://www.genecards.org/cgi-bin/carddisp.pl?gene=USP5 |
| UBE2S | Ubiquitin Conjugating Enzyme E2 S | Protein Coding | 48 | GC19M055399 | 23.72815323 | https://www.genecards.org/cgi-bin/carddisp.pl?gene=UBE2S |
| USP14 | Ubiquitin Specific Peptidase 14 | Protein Coding | 51 | GC18P000158 | 23.66464806 | https://www.genecards.org/cgi-bin/carddisp.pl?gene=USP14 |
| CUL1 | Cullin 1 | Protein Coding | 50 | GC07P148697 | 23.61956024 | https://www.genecards.org/cgi-bin/carddisp.pl?gene=CUL1 |
| UBE2B | Ubiquitin Conjugating Enzyme E2 B | Protein Coding | 50 | GC05P134371 | 23.49440956 | https://www.genecards.org/cgi-bin/carddisp.pl?gene=UBE2B |
| UBE2H | Ubiquitin Conjugating Enzyme E2 H | Protein Coding | 50 | GC07M129830 | 22.97687149 | https://www.genecards.org/cgi-bin/carddisp.pl?gene=UBE2H |
| UBE2G2 | Ubiquitin Conjugating Enzyme E2 G2 | Protein Coding | 48 | GC21M044768 | 22.92201233 | https://www.genecards.org/cgi-bin/carddisp.pl?gene=UBE2G2 |
| BTRC | Beta-Transducin Repeat Containing E3 Ubiquitin Protein Ligase | Protein Coding | 52 | GC10P101354 | 22.71395874 | https://www.genecards.org/cgi-bin/carddisp.pl?gene=BTRC |
| CBL | Cbl Proto-Oncogene | Protein Coding | 59 | GC11P119206 | 22.40759468 | https://www.genecards.org/cgi-bin/carddisp.pl?gene=CBL |
| UBE2G1 | Ubiquitin Conjugating Enzyme E2 G1 | Protein Coding | 49 | GC17M012392 | 22.33669662 | https://www.genecards.org/cgi-bin/carddisp.pl?gene=UBE2G1 |
| UBE2A | Ubiquitin Conjugating Enzyme E2 A | Protein Coding | 51 | GC0XP119734 | 22.1793766 | https://www.genecards.org/cgi-bin/carddisp.pl?gene=UBE2A |
| UCHL3 | Ubiquitin C-Terminal Hydrolase L3 | Protein Coding | 50 | GC13P075550 | 22.04996872 | https://www.genecards.org/cgi-bin/carddisp.pl?gene=UCHL3 |
| FBXW7 | F-Box And WD Repeat Domain Containing 7 | Protein Coding | 52 | GC04M152321 | 22.02902412 | https://www.genecards.org/cgi-bin/carddisp.pl?gene=FBXW7 |
| SMURF2 | SMAD Specific E3 Ubiquitin Protein Ligase 2 | Protein Coding | 51 | GC17M064542 | 21.96029472 | https://www.genecards.org/cgi-bin/carddisp.pl?gene=SMURF2 |
| UBE2L6 | Ubiquitin Conjugating Enzyme E2 L6 | Protein Coding | 46 | GC11M105797 | 21.6193924 | https://www.genecards.org/cgi-bin/carddisp.pl?gene=UBE2L6 |
| UBA52 | Ubiquitin A-52 Residue Ribosomal Protein Fusion Product 1 | Protein Coding | 47 | GC19P018563 | 21.58101463 | https://www.genecards.org/cgi-bin/carddisp.pl?gene=UBA52 |
| UBE2W | Ubiquitin Conjugating Enzyme E2 W | Protein Coding | 43 | GC08M073780 | 21.44153214 | https://www.genecards.org/cgi-bin/carddisp.pl?gene=UBE2W |
| UBE2T | Ubiquitin Conjugating Enzyme E2 T | Protein Coding | 51 | GC01M202332 | 21.38476944 | https://www.genecards.org/cgi-bin/carddisp.pl?gene=UBE2T |
| SUMO1 | Small Ubiquitin Like Modifier 1 | Protein Coding | 53 | GC02M202206 | 21.290905 | https://www.genecards.org/cgi-bin/carddisp.pl?gene=SUMO1 |
| UBA6 | Ubiquitin Like Modifier Activating Enzyme 6 | Protein Coding | 45 | GC04M067612 | 21.15822983 | https://www.genecards.org/cgi-bin/carddisp.pl?gene=UBA6 |
| ISG15 | ISG15 Ubiquitin Like Modifier | Protein Coding | 53 | GC01P001001 | 21.07527733 | https://www.genecards.org/cgi-bin/carddisp.pl?gene=ISG15 |
| USP9X | Ubiquitin Specific Peptidase 9 X-Linked | Protein Coding | 55 | GC0XP041085 | 20.95132637 | https://www.genecards.org/cgi-bin/carddisp.pl?gene=USP9X |
| OTUB1 | OTU Deubiquitinase, Ubiquitin Aldehyde Binding 1 | Protein Coding | 48 | GC11P063985 | 20.94807053 | https://www.genecards.org/cgi-bin/carddisp.pl?gene=OTUB1 |
| SMURF1 | SMAD Specific E3 Ubiquitin Protein Ligase 1 | Protein Coding | 52 | GC07M099027 | 20.94749069 | https://www.genecards.org/cgi-bin/carddisp.pl?gene=SMURF1 |
| USP8 | Ubiquitin Specific Peptidase 8 | Protein Coding | 53 | GC15P050424 | 20.90711212 | https://www.genecards.org/cgi-bin/carddisp.pl?gene=USP8 |
| SKP1 | S-Phase Kinase Associated Protein 1 | Protein Coding | 51 | GC05M134148 | 20.79914474 | https://www.genecards.org/cgi-bin/carddisp.pl?gene=SKP1 |
| RNF8 | Ring Finger Protein 8 | Protein Coding | 48 | GC06P100352 | 20.77965736 | https://www.genecards.org/cgi-bin/carddisp.pl?gene=RNF8 |
| TP53 | Tumor Protein P53 | Protein Coding | 61 | GC17M007661 | 20.71396637 | https://www.genecards.org/cgi-bin/carddisp.pl?gene=TP53 |
| TRAF6 | TNF Receptor Associated Factor 6 | Protein Coding | 53 | GC11M036467 | 20.63967514 | https://www.genecards.org/cgi-bin/carddisp.pl?gene=TRAF6 |
| ARIH1 | Ariadne RBR E3 Ubiquitin Protein Ligase 1 | Protein Coding | 46 | GC15P072474 | 20.44568062 | https://www.genecards.org/cgi-bin/carddisp.pl?gene=ARIH1 |
| CUL3 | Cullin 3 | Protein Coding | 55 | GC02M224470 | 20.38814735 | https://www.genecards.org/cgi-bin/carddisp.pl?gene=CUL3 |
| WWP1 | WW Domain Containing E3 Ubiquitin Protein Ligase 1 | Protein Coding | 48 | GC08P086342 | 20.37010956 | https://www.genecards.org/cgi-bin/carddisp.pl?gene=WWP1 |
| UBR5 | Ubiquitin Protein Ligase E3 Component N-Recognin 5 | Protein Coding | 47 | GC08M102252 | 20.30358124 | https://www.genecards.org/cgi-bin/carddisp.pl?gene=UBR5 |
| UBE2M | Ubiquitin Conjugating Enzyme E2 M | Protein Coding | 47 | GC19M058555 | 20.27430725 | https://www.genecards.org/cgi-bin/carddisp.pl?gene=UBE2M |
| UBE2J1 | Ubiquitin Conjugating Enzyme E2 J1 | Protein Coding | 48 | GC06M089326 | 20.14269066 | https://www.genecards.org/cgi-bin/carddisp.pl?gene=UBE2J1 |
| USP15 | Ubiquitin Specific Peptidase 15 | Protein Coding | 53 | GC12P062260 | 20.08381081 | https://www.genecards.org/cgi-bin/carddisp.pl?gene=USP15 |
| USP2 | Ubiquitin Specific Peptidase 2 | Protein Coding | 50 | GC11M119355 | 19.9698925 | https://www.genecards.org/cgi-bin/carddisp.pl?gene=USP2 |
| UBE3C | Ubiquitin Protein Ligase E3C | Protein Coding | 48 | GC07P157138 | 19.95764351 | https://www.genecards.org/cgi-bin/carddisp.pl?gene=UBE3C |
| USP4 | Ubiquitin Specific Peptidase 4 | Protein Coding | 50 | GC03M049277 | 19.92492676 | https://www.genecards.org/cgi-bin/carddisp.pl?gene=USP4 |
| RNF31 | Ring Finger Protein 31 | Protein Coding | 47 | GC14P024146 | 19.7518425 | https://www.genecards.org/cgi-bin/carddisp.pl?gene=RNF31 |
| GRN | Granulin Precursor | Protein Coding | 55 | GC17P044345 | 19.65295219 | https://www.genecards.org/cgi-bin/carddisp.pl?gene=GRN |
| SKP2 | S-Phase Kinase Associated Protein 2 | Protein Coding | 51 | GC05P036151 | 19.62606239 | https://www.genecards.org/cgi-bin/carddisp.pl?gene=SKP2 |
| UBE2J2 | Ubiquitin Conjugating Enzyme E2 J2 | Protein Coding | 47 | GC01M009114 | 19.24556541 | https://www.genecards.org/cgi-bin/carddisp.pl?gene=UBE2J2 |
| UBQLN2 | Ubiquilin 2 | Protein Coding | 50 | GC0XP056563 | 19.2327137 | https://www.genecards.org/cgi-bin/carddisp.pl?gene=UBQLN2 |
| AMFR | Autocrine Motility Factor Receptor | Protein Coding | 48 | GC16M056361 | 19.17562485 | https://www.genecards.org/cgi-bin/carddisp.pl?gene=AMFR |
| UBE2V1 | Ubiquitin Conjugating Enzyme E2 V1 | Protein Coding | 49 | GC20M050082 | 19.11627197 | https://www.genecards.org/cgi-bin/carddisp.pl?gene=UBE2V1 |
| WWP2 | WW Domain Containing E3 Ubiquitin Protein Ligase 2 | Protein Coding | 51 | GC16P069796 | 19.0170517 | https://www.genecards.org/cgi-bin/carddisp.pl?gene=WWP2 |
| COP1 | COP1 E3 Ubiquitin Ligase | Protein Coding | 48 | GC01M175944 | 19.00357819 | https://www.genecards.org/cgi-bin/carddisp.pl?gene=COP1 |
| USP1 | Ubiquitin Specific Peptidase 1 | Protein Coding | 51 | GC01P062436 | 18.99753952 | https://www.genecards.org/cgi-bin/carddisp.pl?gene=USP1 |
| RNF168 | Ring Finger Protein 168 | Protein Coding | 49 | GC03M196468 | 18.99357033 | https://www.genecards.org/cgi-bin/carddisp.pl?gene=RNF168 |
| UBE2D4 | Ubiquitin Conjugating Enzyme E2 D4 (Putative) | Protein Coding | 45 | GC07P043926 | 18.95079231 | https://www.genecards.org/cgi-bin/carddisp.pl?gene=UBE2D4 |
| UBE2E2 | Ubiquitin Conjugating Enzyme E2 E2 | Protein Coding | 47 | GC03P023221 | 18.86924171 | https://www.genecards.org/cgi-bin/carddisp.pl?gene=UBE2E2 |
| USP11 | Ubiquitin Specific Peptidase 11 | Protein Coding | 48 | GC0XP047232 | 18.84961891 | https://www.genecards.org/cgi-bin/carddisp.pl?gene=USP11 |
| VHL | Von Hippel-Lindau Tumor Suppressor | Protein Coding | 54 | GC03P014317 | 18.841959 | https://www.genecards.org/cgi-bin/carddisp.pl?gene=VHL |
| CBLB | Cbl Proto-Oncogene B | Protein Coding | 52 | GC03M105655 | 18.77040672 | https://www.genecards.org/cgi-bin/carddisp.pl?gene=CBLB |
| SIAH1 | Siah E3 Ubiquitin Protein Ligase 1 | Protein Coding | 52 | GC16M048357 | 18.57855415 | https://www.genecards.org/cgi-bin/carddisp.pl?gene=SIAH1 |
| UBE2R2 | Ubiquitin Conjugating Enzyme E2 R2 | Protein Coding | 49 | GC09P050932 | 18.54647827 | https://www.genecards.org/cgi-bin/carddisp.pl?gene=UBE2R2 |
| UBA7 | Ubiquitin Like Modifier Activating Enzyme 7 | Protein Coding | 47 | GC03M049805 | 18.33481026 | https://www.genecards.org/cgi-bin/carddisp.pl?gene=UBA7 |
| USP25 | Ubiquitin Specific Peptidase 25 | Protein Coding | 48 | GC21P015730 | 18.21228981 | https://www.genecards.org/cgi-bin/carddisp.pl?gene=USP25 |
| USP19 | Ubiquitin Specific Peptidase 19 | Protein Coding | 46 | GC03M052150 | 18.16582298 | https://www.genecards.org/cgi-bin/carddisp.pl?gene=USP19 |
| RBCK1 | RANBP2-Type And C3HC4-Type Zinc Finger Containing 1 | Protein Coding | 48 | GC20P000407 | 17.77719879 | https://www.genecards.org/cgi-bin/carddisp.pl?gene=RBCK1 |
| UBE2O | Ubiquitin Conjugating Enzyme E2 O | Protein Coding | 48 | GC17M076389 | 17.74401474 | https://www.genecards.org/cgi-bin/carddisp.pl?gene=UBE2O |
| NEDD8 | NEDD8 Ubiquitin Like Modifier | Protein Coding | 47 | GC14M024216 | 17.72441673 | https://www.genecards.org/cgi-bin/carddisp.pl?gene=NEDD8 |
| USP13 | Ubiquitin Specific Peptidase 13 | Protein Coding | 50 | GC03P179652 | 17.64603424 | https://www.genecards.org/cgi-bin/carddisp.pl?gene=USP13 |
| UBE2Z | Ubiquitin Conjugating Enzyme E2 Z | Protein Coding | 45 | GC17P048908 | 17.42637825 | https://www.genecards.org/cgi-bin/carddisp.pl?gene=UBE2Z |
| SIAH2 | Siah E3 Ubiquitin Protein Ligase 2 | Protein Coding | 49 | GC03M150741 | 17.38222122 | https://www.genecards.org/cgi-bin/carddisp.pl?gene=SIAH2 |
| UBE2V2 | Ubiquitin Conjugating Enzyme E2 V2 | Protein Coding | 47 | GC08P047998 | 17.33132362 | https://www.genecards.org/cgi-bin/carddisp.pl?gene=UBE2V2 |
| USP20 | Ubiquitin Specific Peptidase 20 | Protein Coding | 50 | GC09P129834 | 17.25568771 | https://www.genecards.org/cgi-bin/carddisp.pl?gene=USP20 |
| UBE2Q2 | Ubiquitin Conjugating Enzyme E2 Q2 | Protein Coding | 45 | GC15P075843 | 17.22831535 | https://www.genecards.org/cgi-bin/carddisp.pl?gene=UBE2Q2 |
| USP10 | Ubiquitin Specific Peptidase 10 | Protein Coding | 51 | GC16P084702 | 17.19701958 | https://www.genecards.org/cgi-bin/carddisp.pl?gene=USP10 |
| UBR2 | Ubiquitin Protein Ligase E3 Component N-Recognin 2 | Protein Coding | 47 | GC06P100387 | 17.16678238 | https://www.genecards.org/cgi-bin/carddisp.pl?gene=UBR2 |
| UBR1 | Ubiquitin Protein Ligase E3 Component N-Recognin 1 | Protein Coding | 50 | GC15M042942 | 17.15636063 | https://www.genecards.org/cgi-bin/carddisp.pl?gene=UBR1 |
| DDB1 | Damage Specific DNA Binding Protein 1 | Protein Coding | 52 | GC11M105899 | 17.12559891 | https://www.genecards.org/cgi-bin/carddisp.pl?gene=DDB1 |
| ARIH2 | Ariadne RBR E3 Ubiquitin Protein Ligase 2 | Protein Coding | 46 | GC03P048918 | 17.08104515 | https://www.genecards.org/cgi-bin/carddisp.pl?gene=ARIH2 |
| CUL2 | Cullin 2 | Protein Coding | 51 | GC10M035046 | 16.98381996 | https://www.genecards.org/cgi-bin/carddisp.pl?gene=CUL2 |
| CUL4A | Cullin 4A | Protein Coding | 50 | GC13P113208 | 16.9066124 | https://www.genecards.org/cgi-bin/carddisp.pl?gene=CUL4A |
| UBE2Q1 | Ubiquitin Conjugating Enzyme E2 Q1 | Protein Coding | 44 | GC01M154548 | 16.70597458 | https://www.genecards.org/cgi-bin/carddisp.pl?gene=UBE2Q1 |
| UBD | Ubiquitin D | Protein Coding | 44 | GC06M077772 | 16.64254951 | https://www.genecards.org/cgi-bin/carddisp.pl?gene=UBD |
| RAD18 | RAD18 E3 Ubiquitin Protein Ligase | Protein Coding | 48 | GC03M008775 | 16.62303734 | https://www.genecards.org/cgi-bin/carddisp.pl?gene=RAD18 |
| HERC2 | HECT And RLD Domain Containing E3 Ubiquitin Protein Ligase 2 | Protein Coding | 52 | GC15M028111 | 16.55125237 | https://www.genecards.org/cgi-bin/carddisp.pl?gene=HERC2 |
| UBA3 | Ubiquitin Like Modifier Activating Enzyme 3 | Protein Coding | 46 | GC03M069054 | 16.4715023 | https://www.genecards.org/cgi-bin/carddisp.pl?gene=UBA3 |
| UHRF1 | Ubiquitin Like With PHD And Ring Finger Domains 1 | Protein Coding | 46 | GC19P005393 | 16.41776276 | https://www.genecards.org/cgi-bin/carddisp.pl?gene=UHRF1 |
| SUMO2 | Small Ubiquitin Like Modifier 2 | Protein Coding | 46 | GC17M075165 | 16.37449265 | https://www.genecards.org/cgi-bin/carddisp.pl?gene=SUMO2 |
| RNF40 | Ring Finger Protein 40 | Protein Coding | 47 | GC16P045084 | 16.28058815 | https://www.genecards.org/cgi-bin/carddisp.pl?gene=RNF40 |
| USP21 | Ubiquitin Specific Peptidase 21 | Protein Coding | 45 | GC01P161159 | 16.25975227 | https://www.genecards.org/cgi-bin/carddisp.pl?gene=USP21 |
| TRIP12 | Thyroid Hormone Receptor Interactor 12 | Protein Coding | 51 | GC02M229763 | 16.24992561 | https://www.genecards.org/cgi-bin/carddisp.pl?gene=TRIP12 |
| TARDBP | TAR DNA Binding Protein | Protein Coding | 53 | GC01P011013 | 16.24650002 | https://www.genecards.org/cgi-bin/carddisp.pl?gene=TARDBP |
| BAP1 | BRCA1 Associated Protein 1 | Protein Coding | 54 | GC03M052401 | 16.23365021 | https://www.genecards.org/cgi-bin/carddisp.pl?gene=BAP1 |
| USP48 | Ubiquitin Specific Peptidase 48 | Protein Coding | 46 | GC01M021678 | 16.21523476 | https://www.genecards.org/cgi-bin/carddisp.pl?gene=USP48 |
| UFD1 | Ubiquitin Recognition Factor In ER Associated Degradation 1 | Protein Coding | 51 | GC22M019551 | 16.208395 | https://www.genecards.org/cgi-bin/carddisp.pl?gene=UFD1 |
| BRCA1 | BRCA1 DNA Repair Associated | Protein Coding | 57 | GC17M043044 | 16.2013855 | https://www.genecards.org/cgi-bin/carddisp.pl?gene=BRCA1 |
| XIAP | X-Linked Inhibitor Of Apoptosis | Protein Coding | 59 | GC0XP123859 | 16.13943672 | https://www.genecards.org/cgi-bin/carddisp.pl?gene=XIAP |
| UBE2U | Ubiquitin Conjugating Enzyme E2 U | Protein Coding | 39 | GC01P064203 | 15.9795332 | https://www.genecards.org/cgi-bin/carddisp.pl?gene=UBE2U |
| TRAF2 | TNF Receptor Associated Factor 2 | Protein Coding | 51 | GC09P136881 | 15.94446278 | https://www.genecards.org/cgi-bin/carddisp.pl?gene=TRAF2 |
| RNF5 | Ring Finger Protein 5 | Protein Coding | 48 | GC06P100230 | 15.94403839 | https://www.genecards.org/cgi-bin/carddisp.pl?gene=RNF5 |
| RNF20 | Ring Finger Protein 20 | Protein Coding | 47 | GC09P101533 | 15.93205929 | https://www.genecards.org/cgi-bin/carddisp.pl?gene=RNF20 |
| SYVN1 | Synoviolin 1 | Protein Coding | 47 | GC11M106081 | 15.74924755 | https://www.genecards.org/cgi-bin/carddisp.pl?gene=SYVN1 |
| UHRF2 | Ubiquitin Like With PHD And Ring Finger Domains 2 | Protein Coding | 47 | GC09P006413 | 15.72635555 | https://www.genecards.org/cgi-bin/carddisp.pl?gene=UHRF2 |
| RNF216 | Ring Finger Protein 216 | Protein Coding | 50 | GC07M005620 | 15.70435333 | https://www.genecards.org/cgi-bin/carddisp.pl?gene=RNF216 |
| BIRC2 | Baculoviral IAP Repeat Containing 2 | Protein Coding | 54 | GC11P102347 | 15.68502998 | https://www.genecards.org/cgi-bin/carddisp.pl?gene=BIRC2 |
| HECTD1 | HECT Domain E3 Ubiquitin Protein Ligase 1 | Protein Coding | 45 | GC14M031100 | 15.64820004 | https://www.genecards.org/cgi-bin/carddisp.pl?gene=HECTD1 |
| HACE1 | HECT Domain And Ankyrin Repeat Containing E3 Ubiquitin Protein Ligase 1 | Protein Coding | 48 | GC06M104728 | 15.63986301 | https://www.genecards.org/cgi-bin/carddisp.pl?gene=HACE1 |
| TRIM25 | Tripartite Motif Containing 25 | Protein Coding | 52 | GC17M056836 | 15.55361462 | https://www.genecards.org/cgi-bin/carddisp.pl?gene=TRIM25 |
| UBE3B | Ubiquitin Protein Ligase E3B | Protein Coding | 47 | GC12P109477 | 15.48333263 | https://www.genecards.org/cgi-bin/carddisp.pl?gene=UBE3B |
| USP33 | Ubiquitin Specific Peptidase 33 | Protein Coding | 46 | GC01M077695 | 15.41474056 | https://www.genecards.org/cgi-bin/carddisp.pl?gene=USP33 |
| RNF19A | Ring Finger Protein 19A, RBR E3 Ubiquitin Protein Ligase | Protein Coding | 47 | GC08M100257 | 15.37926579 | https://www.genecards.org/cgi-bin/carddisp.pl?gene=RNF19A |
| PSMD4 | Proteasome 26S Subunit Ubiquitin Receptor, Non-ATPase 4 | Protein Coding | 50 | GC01P153214 | 15.37546635 | https://www.genecards.org/cgi-bin/carddisp.pl?gene=PSMD4 |
| CCNF | Cyclin F | Protein Coding | 47 | GC16P002429 | 15.33763885 | https://www.genecards.org/cgi-bin/carddisp.pl?gene=CCNF |
| MUL1 | Mitochondrial E3 Ubiquitin Protein Ligase 1 | Protein Coding | 45 | GC01M020499 | 15.27324581 | https://www.genecards.org/cgi-bin/carddisp.pl?gene=MUL1 |
| MARCHF6 | Membrane Associated Ring-CH-Type Finger 6 | Protein Coding | 42 | GC05P010547 | 15.24729919 | https://www.genecards.org/cgi-bin/carddisp.pl?gene=MARCHF6 |
| OPTN | Optineurin | Protein Coding | 50 | GC10P013099 | 15.21683311 | https://www.genecards.org/cgi-bin/carddisp.pl?gene=OPTN |
| UCHL5 | Ubiquitin C-Terminal Hydrolase L5 | Protein Coding | 47 | GC01M193012 | 15.12043858 | https://www.genecards.org/cgi-bin/carddisp.pl?gene=UCHL5 |
| UBA5 | Ubiquitin Like Modifier Activating Enzyme 5 | Protein Coding | 48 | GC03P132654 | 15.09455681 | https://www.genecards.org/cgi-bin/carddisp.pl?gene=UBA5 |
| CUL5 | Cullin 5 | Protein Coding | 50 | GC11P108008 | 15.04967976 | https://www.genecards.org/cgi-bin/carddisp.pl?gene=CUL5 |
| USP16 | Ubiquitin Specific Peptidase 16 | Protein Coding | 46 | GC21P029024 | 14.97540665 | https://www.genecards.org/cgi-bin/carddisp.pl?gene=USP16 |
| USP47 | Ubiquitin Specific Peptidase 47 | Protein Coding | 44 | GC11P011842 | 14.9656601 | https://www.genecards.org/cgi-bin/carddisp.pl?gene=USP47 |
| TRIM63 | Tripartite Motif Containing 63 | Protein Coding | 48 | GC01M028497 | 14.95080662 | https://www.genecards.org/cgi-bin/carddisp.pl?gene=TRIM63 |
| UBR4 | Ubiquitin Protein Ligase E3 Component N-Recognin 4 | Protein Coding | 45 | GC01M019074 | 14.93264294 | https://www.genecards.org/cgi-bin/carddisp.pl?gene=UBR4 |
| AREL1 | Apoptosis Resistant E3 Ubiquitin Protein Ligase 1 | Protein Coding | 44 | GC14M074653 | 14.84709167 | https://www.genecards.org/cgi-bin/carddisp.pl?gene=AREL1 |
| MIB1 | MIB E3 Ubiquitin Protein Ligase 1 | Protein Coding | 51 | GC18P021704 | 14.79762554 | https://www.genecards.org/cgi-bin/carddisp.pl?gene=MIB1 |
| HNRNPA1 | Heterogeneous Nuclear Ribonucleoprotein A1 | Protein Coding | 55 | GC12P054280 | 14.7937355 | https://www.genecards.org/cgi-bin/carddisp.pl?gene=HNRNPA1 |
| HECW2 | HECT, C2 And WW Domain Containing E3 Ubiquitin Protein Ligase 2 | Protein Coding | 49 | GC02M196194 | 14.7536087 | https://www.genecards.org/cgi-bin/carddisp.pl?gene=HECW2 |
| USP36 | Ubiquitin Specific Peptidase 36 | Protein Coding | 43 | GC17M078787 | 14.72057343 | https://www.genecards.org/cgi-bin/carddisp.pl?gene=USP36 |
| TRIM21 | Tripartite Motif Containing 21 | Protein Coding | 50 | GC11M004384 | 14.70735741 | https://www.genecards.org/cgi-bin/carddisp.pl?gene=TRIM21 |
| USP3 | Ubiquitin Specific Peptidase 3 | Protein Coding | 46 | GC15P063504 | 14.64448357 | https://www.genecards.org/cgi-bin/carddisp.pl?gene=USP3 |
| UBQLN1 | Ubiquilin 1 | Protein Coding | 49 | GC09M083659 | 14.62230301 | https://www.genecards.org/cgi-bin/carddisp.pl?gene=UBQLN1 |
| DTL | Denticleless E3 Ubiquitin Protein Ligase Homolog | Protein Coding | 44 | GC01P212035 | 14.59458923 | https://www.genecards.org/cgi-bin/carddisp.pl?gene=DTL |
| USP22 | Ubiquitin Specific Peptidase 22 | Protein Coding | 47 | GC17M020999 | 14.52153111 | https://www.genecards.org/cgi-bin/carddisp.pl?gene=USP22 |
| FBXW11 | F-Box And WD Repeat Domain Containing 11 | Protein Coding | 52 | GC05M171861 | 14.44142437 | https://www.genecards.org/cgi-bin/carddisp.pl?gene=FBXW11 |
| USP24 | Ubiquitin Specific Peptidase 24 | Protein Coding | 50 | GC01M055066 | 14.4074707 | https://www.genecards.org/cgi-bin/carddisp.pl?gene=USP24 |
| RNF111 | Ring Finger Protein 111 | Protein Coding | 46 | GC15P058866 | 14.40196419 | https://www.genecards.org/cgi-bin/carddisp.pl?gene=RNF111 |
| HECW1 | HECT, C2 And WW Domain Containing E3 Ubiquitin Protein Ligase 1 | Protein Coding | 44 | GC07P043112 | 14.32943249 | https://www.genecards.org/cgi-bin/carddisp.pl?gene=HECW1 |
| TRIM32 | Tripartite Motif Containing 32 | Protein Coding | 50 | GC09P116687 | 14.30493355 | https://www.genecards.org/cgi-bin/carddisp.pl?gene=TRIM32 |
| ELOB | Elongin B | Protein Coding | 45 | GC16M009866 | 14.29764938 | https://www.genecards.org/cgi-bin/carddisp.pl?gene=ELOB |
| RNF144A | Ring Finger Protein 144A | Protein Coding | 45 | GC02P006917 | 14.23851395 | https://www.genecards.org/cgi-bin/carddisp.pl?gene=RNF144A |
| FUS | FUS RNA Binding Protein | Protein Coding | 51 | GC16P031180 | 14.17036915 | https://www.genecards.org/cgi-bin/carddisp.pl?gene=FUS |
| CUL4B | Cullin 4B | Protein Coding | 50 | GC0XM120524 | 14.16279888 | https://www.genecards.org/cgi-bin/carddisp.pl?gene=CUL4B |
| USP9Y | Ubiquitin Specific Peptidase 9 Y-Linked | Protein Coding | 44 | GC0YP012569 | 14.13906097 | https://www.genecards.org/cgi-bin/carddisp.pl?gene=USP9Y |
| SUMO3 | Small Ubiquitin Like Modifier 3 | Protein Coding | 47 | GC21M044805 | 14.02994537 | https://www.genecards.org/cgi-bin/carddisp.pl?gene=SUMO3 |
| RNF2 | Ring Finger Protein 2 | Protein Coding | 51 | GC01P185045 | 14.019104 | https://www.genecards.org/cgi-bin/carddisp.pl?gene=RNF2 |
| RCHY1 | Ring Finger And CHY Zinc Finger Domain Containing 1 | Protein Coding | 44 | GC04M075479 | 14.0011816 | https://www.genecards.org/cgi-bin/carddisp.pl?gene=RCHY1 |
| UBR3 | Ubiquitin Protein Ligase E3 Component N-Recognin 3 | Protein Coding | 41 | GC02P169827 | 13.98936081 | https://www.genecards.org/cgi-bin/carddisp.pl?gene=UBR3 |
| TOPORS | TOP1 Binding Arginine/Serine Rich Protein, E3 Ubiquitin Ligase | Protein Coding | 51 | GC09M032540 | 13.90442753 | https://www.genecards.org/cgi-bin/carddisp.pl?gene=TOPORS |
| RNF4 | Ring Finger Protein 4 | Protein Coding | 46 | GC04P002462 | 13.8815937 | https://www.genecards.org/cgi-bin/carddisp.pl?gene=RNF4 |
| CHFR | Checkpoint With Forkhead And Ring Finger Domains | Protein Coding | 45 | GC12M132822 | 13.80503654 | https://www.genecards.org/cgi-bin/carddisp.pl?gene=CHFR |
| NHLRC1 | NHL Repeat Containing E3 Ubiquitin Protein Ligase 1 | Protein Coding | 47 | GC06M018120 | 13.79421043 | https://www.genecards.org/cgi-bin/carddisp.pl?gene=NHLRC1 |
| USP30 | Ubiquitin Specific Peptidase 30 | Protein Coding | 44 | GC12P109027 | 13.7471571 | https://www.genecards.org/cgi-bin/carddisp.pl?gene=USP30 |
| LTN1 | Listerin E3 Ubiquitin Protein Ligase 1 | Protein Coding | 44 | GC21M028928 | 13.74557209 | https://www.genecards.org/cgi-bin/carddisp.pl?gene=LTN1 |
| USP28 | Ubiquitin Specific Peptidase 28 | Protein Coding | 46 | GC11M113797 | 13.73915195 | https://www.genecards.org/cgi-bin/carddisp.pl?gene=USP28 |
| HERC4 | HECT And RLD Domain Containing E3 Ubiquitin Protein Ligase 4 | Protein Coding | 43 | GC10M067921 | 13.7386694 | https://www.genecards.org/cgi-bin/carddisp.pl?gene=HERC4 |
| MYCBP2 | MYC Binding Protein 2 | Protein Coding | 44 | GC13M077044 | 13.73286057 | https://www.genecards.org/cgi-bin/carddisp.pl?gene=MYCBP2 |
| CYLD | CYLD Lysine 63 Deubiquitinase | Protein Coding | 55 | GC16P050742 | 13.72035599 | https://www.genecards.org/cgi-bin/carddisp.pl?gene=CYLD |
| MIB2 | MIB E3 Ubiquitin Protein Ligase 2 | Protein Coding | 44 | GC01P004985 | 13.71077442 | https://www.genecards.org/cgi-bin/carddisp.pl?gene=MIB2 |
| UBL4A | Ubiquitin Like 4A | Protein Coding | 42 | GC0XM154483 | 13.69628239 | https://www.genecards.org/cgi-bin/carddisp.pl?gene=UBL4A |
| UBE3D | Ubiquitin Protein Ligase E3D | Protein Coding | 39 | GC06M082892 | 13.69541931 | https://www.genecards.org/cgi-bin/carddisp.pl?gene=UBE3D |
| LNX1 | Ligand Of Numb-Protein X 1 | Protein Coding | 49 | GC04M053459 | 13.64606285 | https://www.genecards.org/cgi-bin/carddisp.pl?gene=LNX1 |
| USP44 | Ubiquitin Specific Peptidase 44 | Protein Coding | 45 | GC12M095516 | 13.62387848 | https://www.genecards.org/cgi-bin/carddisp.pl?gene=USP44 |
| USP46 | Ubiquitin Specific Peptidase 46 | Protein Coding | 43 | GC04M052590 | 13.62306499 | https://www.genecards.org/cgi-bin/carddisp.pl?gene=USP46 |
| NPLOC4 | NPL4 Homolog, Ubiquitin Recognition Factor | Protein Coding | 47 | GC17M081556 | 13.61562157 | https://www.genecards.org/cgi-bin/carddisp.pl?gene=NPLOC4 |
| USP34 | Ubiquitin Specific Peptidase 34 | Protein Coding | 46 | GC02M061187 | 13.51800823 | https://www.genecards.org/cgi-bin/carddisp.pl?gene=USP34 |
| MGRN1 | Mahogunin Ring Finger 1 | Protein Coding | 46 | GC16P019961 | 13.50233364 | https://www.genecards.org/cgi-bin/carddisp.pl?gene=MGRN1 |
| ELOC | Elongin C | Protein Coding | 48 | GC08M073939 | 13.47767735 | https://www.genecards.org/cgi-bin/carddisp.pl?gene=ELOC |
| OTUB2 | OTU Deubiquitinase, Ubiquitin Aldehyde Binding 2 | Protein Coding | 42 | GC14P094023 | 13.43745232 | https://www.genecards.org/cgi-bin/carddisp.pl?gene=OTUB2 |
| PJA1 | Praja Ring Finger Ubiquitin Ligase 1 | Protein Coding | 47 | GC0XM069160 | 13.43328953 | https://www.genecards.org/cgi-bin/carddisp.pl?gene=PJA1 |
| HECTD3 | HECT Domain E3 Ubiquitin Protein Ligase 3 | Protein Coding | 42 | GC01M045002 | 13.42279243 | https://www.genecards.org/cgi-bin/carddisp.pl?gene=HECTD3 |
| HERC1 | HECT And RLD Domain Containing E3 Ubiquitin Protein Ligase Family Member 1 | Protein Coding | 47 | GC15M063608 | 13.40936852 | https://www.genecards.org/cgi-bin/carddisp.pl?gene=HERC1 |
| RNF213 | Ring Finger Protein 213 | Protein Coding | 45 | GC17P080260 | 13.36587334 | https://www.genecards.org/cgi-bin/carddisp.pl?gene=RNF213 |
| RNF125 | Ring Finger Protein 125 | Protein Coding | 47 | GC18P032253 | 13.30120754 | https://www.genecards.org/cgi-bin/carddisp.pl?gene=RNF125 |
| USP18 | Ubiquitin Specific Peptidase 18 | Protein Coding | 47 | GC22P049093 | 13.29953957 | https://www.genecards.org/cgi-bin/carddisp.pl?gene=USP18 |
| ANAPC11 | Anaphase Promoting Complex Subunit 11 | Protein Coding | 47 | GC17P081890 | 13.27404881 | https://www.genecards.org/cgi-bin/carddisp.pl?gene=ANAPC11 |
| CBLC | Cbl Proto-Oncogene C | Protein Coding | 44 | GC19P044777 | 13.26273251 | https://www.genecards.org/cgi-bin/carddisp.pl?gene=CBLC |
| BIRC6 | Baculoviral IAP Repeat Containing 6 | Protein Coding | 48 | GC02P032357 | 13.16705894 | https://www.genecards.org/cgi-bin/carddisp.pl?gene=BIRC6 |
| HERC3 | HECT And RLD Domain Containing E3 Ubiquitin Protein Ligase 3 | Protein Coding | 42 | GC04P088569 | 13.15673923 | https://www.genecards.org/cgi-bin/carddisp.pl?gene=HERC3 |
| UBQLN4 | Ubiquilin 4 | Protein Coding | 47 | GC01M156033 | 13.1126833 | https://www.genecards.org/cgi-bin/carddisp.pl?gene=UBQLN4 |
| DTX3L | Deltex E3 Ubiquitin Ligase 3L | Protein Coding | 44 | GC03P122564 | 13.06767559 | https://www.genecards.org/cgi-bin/carddisp.pl?gene=DTX3L |
| UIMC1 | Ubiquitin Interaction Motif Containing 1 | Protein Coding | 46 | GC05M176905 | 13.02734089 | https://www.genecards.org/cgi-bin/carddisp.pl?gene=UIMC1 |
| PJA2 | Praja Ring Finger Ubiquitin Ligase 2 | Protein Coding | 44 | GC05M109334 | 13.02537537 | https://www.genecards.org/cgi-bin/carddisp.pl?gene=PJA2 |
| FANCL | FA Complementation Group L | Protein Coding | 52 | GC02M058127 | 12.97396755 | https://www.genecards.org/cgi-bin/carddisp.pl?gene=FANCL |
| PELI1 | Pellino E3 Ubiquitin Protein Ligase 1 | Protein Coding | 48 | GC02M064092 | 12.95911026 | https://www.genecards.org/cgi-bin/carddisp.pl?gene=PELI1 |
| PCNA | Proliferating Cell Nuclear Antigen | Protein Coding | 57 | GC20M005114 | 12.91749096 | https://www.genecards.org/cgi-bin/carddisp.pl?gene=PCNA |
| ADRM1 | ADRM1 26S Proteasome Ubiquitin Receptor | Protein Coding | 46 | GC20P062302 | 12.89091492 | https://www.genecards.org/cgi-bin/carddisp.pl?gene=ADRM1 |
| USP6 | Ubiquitin Specific Peptidase 6 | Protein Coding | 46 | GC17P005116 | 12.84108639 | https://www.genecards.org/cgi-bin/carddisp.pl?gene=USP6 |
| MAPT | Microtubule Associated Protein Tau | Protein Coding | 57 | GC17P045894 | 12.83567429 | https://www.genecards.org/cgi-bin/carddisp.pl?gene=MAPT |
| HERC6 | HECT And RLD Domain Containing E3 Ubiquitin Protein Ligase Family Member 6 | Protein Coding | 42 | GC04P088378 | 12.78695965 | https://www.genecards.org/cgi-bin/carddisp.pl?gene=HERC6 |
| USP12 | Ubiquitin Specific Peptidase 12 | Protein Coding | 43 | GC13M027066 | 12.74953556 | https://www.genecards.org/cgi-bin/carddisp.pl?gene=USP12 |
| FZR1 | Fizzy And Cell Division Cycle 20 Related 1 | Protein Coding | 51 | GC19P003506 | 12.7469883 | https://www.genecards.org/cgi-bin/carddisp.pl?gene=FZR1 |
| USP37 | Ubiquitin Specific Peptidase 37 | Protein Coding | 46 | GC02M218450 | 12.73764896 | https://www.genecards.org/cgi-bin/carddisp.pl?gene=USP37 |
| BARD1 | BRCA1 Associated RING Domain 1 | Protein Coding | 54 | GC02M214725 | 12.71888065 | https://www.genecards.org/cgi-bin/carddisp.pl?gene=BARD1 |
| HERPUD1 | Homocysteine Inducible ER Protein With Ubiquitin Like Domain 1 | Protein Coding | 46 | GC16P056932 | 12.71513653 | https://www.genecards.org/cgi-bin/carddisp.pl?gene=HERPUD1 |
| RNF34 | Ring Finger Protein 34 | Protein Coding | 46 | GC12P121400 | 12.71052074 | https://www.genecards.org/cgi-bin/carddisp.pl?gene=RNF34 |
| TBK1 | TANK Binding Kinase 1 | Protein Coding | 56 | GC12P064451 | 12.69578362 | https://www.genecards.org/cgi-bin/carddisp.pl?gene=TBK1 |
| MARCHF2 | Membrane Associated Ring-CH-Type Finger 2 | Protein Coding | 36 | GC19P008479 | 12.67689705 | https://www.genecards.org/cgi-bin/carddisp.pl?gene=MARCHF2 |
| HECTD2 | HECT Domain E3 Ubiquitin Protein Ligase 2 | Protein Coding | 42 | GC10P091409 | 12.65810776 | https://www.genecards.org/cgi-bin/carddisp.pl?gene=HECTD2 |
| HSPA8 | Heat Shock Protein Family A (Hsp70) Member 8 | Protein Coding | 56 | GC11M123057 | 12.64606571 | https://www.genecards.org/cgi-bin/carddisp.pl?gene=HSPA8 |
| MARCHF8 | Membrane Associated Ring-CH-Type Finger 8 | Protein Coding | 42 | GC10M046842 | 12.64158249 | https://www.genecards.org/cgi-bin/carddisp.pl?gene=MARCHF8 |
| ATXN3 | Ataxin 3 | Protein Coding | 52 | GC14M114120 | 12.62506104 | https://www.genecards.org/cgi-bin/carddisp.pl?gene=ATXN3 |
| RFFL | Ring Finger And FYVE Like Domain Containing E3 Ubiquitin Protein Ligase | Protein Coding | 45 | GC17M056638 | 12.59281921 | https://www.genecards.org/cgi-bin/carddisp.pl?gene=RFFL |
| UBAP1 | Ubiquitin Associated Protein 1 | Protein Coding | 47 | GC09P034179 | 12.52760983 | https://www.genecards.org/cgi-bin/carddisp.pl?gene=UBAP1 |
| HGS | Hepatocyte Growth Factor-Regulated Tyrosine Kinase Substrate | Protein Coding | 50 | GC17P081683 | 12.46327019 | https://www.genecards.org/cgi-bin/carddisp.pl?gene=HGS |
| USP26 | Ubiquitin Specific Peptidase 26 | Protein Coding | 43 | GC0XM133024 | 12.46114635 | https://www.genecards.org/cgi-bin/carddisp.pl?gene=USP26 |
| OTULIN | OTU Deubiquitinase With Linear Linkage Specificity | Protein Coding | 48 | GC05P014667 | 12.42200661 | https://www.genecards.org/cgi-bin/carddisp.pl?gene=OTULIN |
| RNF144B | Ring Finger Protein 144B | Protein Coding | 44 | GC06P018690 | 12.41704559 | https://www.genecards.org/cgi-bin/carddisp.pl?gene=RNF144B |
| RAD23A | RAD23 Homolog A, Nucleotide Excision Repair Protein | Protein Coding | 50 | GC19P015311 | 12.38823223 | https://www.genecards.org/cgi-bin/carddisp.pl?gene=RAD23A |
| UBA2 | Ubiquitin Like Modifier Activating Enzyme 2 | Protein Coding | 52 | GC19P034428 | 12.38171387 | https://www.genecards.org/cgi-bin/carddisp.pl?gene=UBA2 |
| MARCHF7 | Membrane Associated Ring-CH-Type Finger 7 | Protein Coding | 39 | GC02P159713 | 12.37924385 | https://www.genecards.org/cgi-bin/carddisp.pl?gene=MARCHF7 |
| USP40 | Ubiquitin Specific Peptidase 40 | Protein Coding | 42 | GC02M233475 | 12.36583519 | https://www.genecards.org/cgi-bin/carddisp.pl?gene=USP40 |
| USP49 | Ubiquitin Specific Peptidase 49 | Protein Coding | 43 | GC06M078157 | 12.30690002 | https://www.genecards.org/cgi-bin/carddisp.pl?gene=USP49 |
| PRNP | Prion Protein | Protein Coding | 55 | GC20P004686 | 12.29712582 | https://www.genecards.org/cgi-bin/carddisp.pl?gene=PRNP |
| RNF14 | Ring Finger Protein 14 | Protein Coding | 48 | GC05P147643 | 12.29293823 | https://www.genecards.org/cgi-bin/carddisp.pl?gene=RNF14 |
| KEAP1 | Kelch Like ECH Associated Protein 1 | Protein Coding | 56 | GC19M010486 | 12.28538322 | https://www.genecards.org/cgi-bin/carddisp.pl?gene=KEAP1 |
| SHPRH | SNF2 Histone Linker PHD RING Helicase | Protein Coding | 45 | GC06M145863 | 12.27191448 | https://www.genecards.org/cgi-bin/carddisp.pl?gene=SHPRH |
| RNF41 | Ring Finger Protein 41 | Protein Coding | 45 | GC12M056202 | 12.25482941 | https://www.genecards.org/cgi-bin/carddisp.pl?gene=RNF41 |
| RLIM | Ring Finger Protein, LIM Domain Interacting | Protein Coding | 46 | GC0XM074737 | 12.23776436 | https://www.genecards.org/cgi-bin/carddisp.pl?gene=RLIM |
| USP38 | Ubiquitin Specific Peptidase 38 | Protein Coding | 41 | GC04P143184 | 12.23383522 | https://www.genecards.org/cgi-bin/carddisp.pl?gene=USP38 |
| USP32 | Ubiquitin Specific Peptidase 32 | Protein Coding | 46 | GC17M060330 | 12.19812584 | https://www.genecards.org/cgi-bin/carddisp.pl?gene=USP32 |
| PSMC2 | Proteasome 26S Subunit, ATPase 2 | Protein Coding | 47 | GC07P103328 | 12.17809582 | https://www.genecards.org/cgi-bin/carddisp.pl?gene=PSMC2 |
| USP17L2 | Ubiquitin Specific Peptidase 17 Like Family Member 2 | Protein Coding | 35 | GC08M013082 | 12.15877438 | https://www.genecards.org/cgi-bin/carddisp.pl?gene=USP17L2 |
| UBAC1 | UBA Domain Containing 1 | Protein Coding | 43 | GC09M135932 | 12.14672661 | https://www.genecards.org/cgi-bin/carddisp.pl?gene=UBAC1 |
| MARCHF1 | Membrane Associated Ring-CH-Type Finger 1 | Protein Coding | 39 | GC04M163525 | 12.06647301 | https://www.genecards.org/cgi-bin/carddisp.pl?gene=MARCHF1 |
| UBE2F | Ubiquitin Conjugating Enzyme E2 F (Putative) | Protein Coding | 46 | GC02P238014 | 12.05893612 | https://www.genecards.org/cgi-bin/carddisp.pl?gene=UBE2F |
| G2E3 | G2/M-Phase Specific E3 Ubiquitin Protein Ligase | Protein Coding | 45 | GC14P030559 | 12.02679729 | https://www.genecards.org/cgi-bin/carddisp.pl?gene=G2E3 |
| YOD1 | YOD1 Deubiquitinase | Protein Coding | 40 | GC01M207044 | 12.02029991 | https://www.genecards.org/cgi-bin/carddisp.pl?gene=YOD1 |
| CBLL1 | Cbl Proto-Oncogene Like 1 | Protein Coding | 46 | GC07P107743 | 12.01704121 | https://www.genecards.org/cgi-bin/carddisp.pl?gene=CBLL1 |
| TRAF7 | TNF Receptor Associated Factor 7 | Protein Coding | 48 | GC16P019881 | 12.00003433 | https://www.genecards.org/cgi-bin/carddisp.pl?gene=TRAF7 |
| PSMD2 | Proteasome 26S Subunit Ubiquitin Receptor, Non-ATPase 2 | Protein Coding | 50 | GC03P184298 | 11.96281528 | https://www.genecards.org/cgi-bin/carddisp.pl?gene=PSMD2 |
| PSMC5 | Proteasome 26S Subunit, ATPase 5 | Protein Coding | 48 | GC17P063827 | 11.96144867 | https://www.genecards.org/cgi-bin/carddisp.pl?gene=PSMC5 |
| HERC5 | HECT And RLD Domain Containing E3 Ubiquitin Protein Ligase 5 | Protein Coding | 46 | GC04P088457 | 11.95974064 | https://www.genecards.org/cgi-bin/carddisp.pl?gene=HERC5 |
| RNF139 | Ring Finger Protein 139 | Protein Coding | 46 | GC08P124474 | 11.94983578 | https://www.genecards.org/cgi-bin/carddisp.pl?gene=RNF139 |
| RNF7 | Ring Finger Protein 7 | Protein Coding | 46 | GC03P141738 | 11.78768826 | https://www.genecards.org/cgi-bin/carddisp.pl?gene=RNF7 |
| USP35 | Ubiquitin Specific Peptidase 35 | Protein Coding | 42 | GC11P078188 | 11.77636337 | https://www.genecards.org/cgi-bin/carddisp.pl?gene=USP35 |
| ZNRF1 | Zinc And Ring Finger 1 | Protein Coding | 46 | GC16P074999 | 11.76861572 | https://www.genecards.org/cgi-bin/carddisp.pl?gene=ZNRF1 |
| CDC20 | Cell Division Cycle 20 | Protein Coding | 51 | GC01P043358 | 11.75480938 | https://www.genecards.org/cgi-bin/carddisp.pl?gene=CDC20 |
| UBR7 | Ubiquitin Protein Ligase E3 Component N-Recognin 7 | Protein Coding | 45 | GC14P093207 | 11.72177029 | https://www.genecards.org/cgi-bin/carddisp.pl?gene=UBR7 |
| IKBKG | Inhibitor Of Nuclear Factor Kappa B Kinase Regulatory Subunit Gamma | Protein Coding | 55 | GC0XP154541 | 11.68265152 | https://www.genecards.org/cgi-bin/carddisp.pl?gene=IKBKG |
| RNF128 | Ring Finger Protein 128 | Protein Coding | 46 | GC0XP106693 | 11.67143726 | https://www.genecards.org/cgi-bin/carddisp.pl?gene=RNF128 |
| RNF146 | Ring Finger Protein 146 | Protein Coding | 44 | GC06P127266 | 11.67007256 | https://www.genecards.org/cgi-bin/carddisp.pl?gene=RNF146 |
| UBOX5 | U-Box Domain Containing 5 | Protein Coding | 40 | GC20M003107 | 11.66035271 | https://www.genecards.org/cgi-bin/carddisp.pl?gene=UBOX5 |
| HSP90AA1 | Heat Shock Protein 90 Alpha Family Class A Member 1 | Protein Coding | 58 | GC14M102080 | 11.62375641 | https://www.genecards.org/cgi-bin/carddisp.pl?gene=HSP90AA1 |
| BAG6 | BAG Cochaperone 6 | Protein Coding | 45 | GC06M031639 | 11.58633423 | https://www.genecards.org/cgi-bin/carddisp.pl?gene=BAG6 |
| USP31 | Ubiquitin Specific Peptidase 31 | Protein Coding | 43 | GC16M023061 | 11.57434559 | https://www.genecards.org/cgi-bin/carddisp.pl?gene=USP31 |
| HSPA5 | Heat Shock Protein Family A (Hsp70) Member 5 | Protein Coding | 55 | GC09M125234 | 11.5179739 | https://www.genecards.org/cgi-bin/carddisp.pl?gene=HSPA5 |
| CTNNB1 | Catenin Beta 1 | Protein Coding | 60 | GC03P041194 | 11.48426819 | https://www.genecards.org/cgi-bin/carddisp.pl?gene=CTNNB1 |
| HNRNPA2B1 | Heterogeneous Nuclear Ribonucleoprotein A2/B1 | Protein Coding | 52 | GC07M026174 | 11.47144222 | https://www.genecards.org/cgi-bin/carddisp.pl?gene=HNRNPA2B1 |
| MYLIP | Myosin Regulatory Light Chain Interacting Protein | Protein Coding | 47 | GC06P016129 | 11.43603802 | https://www.genecards.org/cgi-bin/carddisp.pl?gene=MYLIP |
| RNF123 | Ring Finger Protein 123 | Protein Coding | 43 | GC03P049689 | 11.42266369 | https://www.genecards.org/cgi-bin/carddisp.pl?gene=RNF123 |
| MARCHF5 | Membrane Associated Ring-CH-Type Finger 5 | Protein Coding | 43 | GC10P096725 | 11.40021706 | https://www.genecards.org/cgi-bin/carddisp.pl?gene=MARCHF5 |
| HSPA1A | Heat Shock Protein Family A (Hsp70) Member 1A | Protein Coding | 53 | GC06P100221 | 11.37464809 | https://www.genecards.org/cgi-bin/carddisp.pl?gene=HSPA1A |
| FBXO11 | F-Box Protein 11 | Protein Coding | 48 | GC02M047789 | 11.35251236 | https://www.genecards.org/cgi-bin/carddisp.pl?gene=FBXO11 |
| RNF114 | Ring Finger Protein 114 | Protein Coding | 44 | GC20P049936 | 11.34768677 | https://www.genecards.org/cgi-bin/carddisp.pl?gene=RNF114 |
| RNF25 | Ring Finger Protein 25 | Protein Coding | 44 | GC02M218663 | 11.3332119 | https://www.genecards.org/cgi-bin/carddisp.pl?gene=RNF25 |
| PSMC3 | Proteasome 26S Subunit, ATPase 3 | Protein Coding | 51 | GC11M105738 | 11.33169651 | https://www.genecards.org/cgi-bin/carddisp.pl?gene=PSMC3 |
| HSPA4 | Heat Shock Protein Family A (Hsp70) Member 4 | Protein Coding | 50 | GC05P133054 | 11.32316113 | https://www.genecards.org/cgi-bin/carddisp.pl?gene=HSPA4 |
| DZIP3 | DAZ Interacting Zinc Finger Protein 3 | Protein Coding | 44 | GC03P108589 | 11.31972122 | https://www.genecards.org/cgi-bin/carddisp.pl?gene=DZIP3 |
| RBBP6 | RB Binding Protein 6, Ubiquitin Ligase | Protein Coding | 46 | GC16P024537 | 11.30887985 | https://www.genecards.org/cgi-bin/carddisp.pl?gene=RBBP6 |
| BIRC3 | Baculoviral IAP Repeat Containing 3 | Protein Coding | 53 | GC11P102317 | 11.25270081 | https://www.genecards.org/cgi-bin/carddisp.pl?gene=BIRC3 |
| RNF138 | Ring Finger Protein 138 | Protein Coding | 44 | GC18P032091 | 11.2495079 | https://www.genecards.org/cgi-bin/carddisp.pl?gene=RNF138 |
| DCAF1 | DDB1 And CUL4 Associated Factor 1 | Protein Coding | 44 | GC03M051395 | 11.24554157 | https://www.genecards.org/cgi-bin/carddisp.pl?gene=DCAF1 |
| SOD1 | Superoxide Dismutase 1 | Protein Coding | 60 | GC21P031659 | 11.24325848 | https://www.genecards.org/cgi-bin/carddisp.pl?gene=SOD1 |
| PSMC4 | Proteasome 26S Subunit, ATPase 4 | Protein Coding | 47 | GC19P079659 | 11.23804092 | https://www.genecards.org/cgi-bin/carddisp.pl?gene=PSMC4 |
| TRAF3 | TNF Receptor Associated Factor 3 | Protein Coding | 53 | GC14P111591 | 11.20524311 | https://www.genecards.org/cgi-bin/carddisp.pl?gene=TRAF3 |
| USP29 | Ubiquitin Specific Peptidase 29 | Protein Coding | 42 | GC19P057119 | 11.18614674 | https://www.genecards.org/cgi-bin/carddisp.pl?gene=USP29 |
| DCTN1 | Dynactin Subunit 1 | Protein Coding | 53 | GC02M074361 | 11.16609192 | https://www.genecards.org/cgi-bin/carddisp.pl?gene=DCTN1 |
| TSG101 | Tumor Susceptibility 101 | Protein Coding | 50 | GC11M018468 | 11.16187096 | https://www.genecards.org/cgi-bin/carddisp.pl?gene=TSG101 |
| UBL7 | Ubiquitin Like 7 | Protein Coding | 42 | GC15M129689 | 11.12388039 | https://www.genecards.org/cgi-bin/carddisp.pl?gene=UBL7 |
| HECTD4 | HECT Domain E3 Ubiquitin Protein Ligase 4 | Protein Coding | 39 | GC12M112160 | 11.1238203 | https://www.genecards.org/cgi-bin/carddisp.pl?gene=HECTD4 |
| TMUB1 | Transmembrane And Ubiquitin Like Domain Containing 1 | Protein Coding | 40 | GC07M151081 | 11.11927319 | https://www.genecards.org/cgi-bin/carddisp.pl?gene=TMUB1 |
| TRIM27 | Tripartite Motif Containing 27 | Protein Coding | 48 | GC06M028903 | 11.06319237 | https://www.genecards.org/cgi-bin/carddisp.pl?gene=TRIM27 |
| MAEA | Macrophage Erythroblast Attacher, E3 Ubiquitin Ligase | Protein Coding | 44 | GC04P001289 | 11.06019306 | https://www.genecards.org/cgi-bin/carddisp.pl?gene=MAEA |
| ANAPC2 | Anaphase Promoting Complex Subunit 2 | Protein Coding | 46 | GC09M137174 | 11.0352335 | https://www.genecards.org/cgi-bin/carddisp.pl?gene=ANAPC2 |
| TNFAIP3 | TNF Alpha Induced Protein 3 | Protein Coding | 55 | GC06P137866 | 11.01523495 | https://www.genecards.org/cgi-bin/carddisp.pl?gene=TNFAIP3 |
| USP43 | Ubiquitin Specific Peptidase 43 | Protein Coding | 42 | GC17P009644 | 11.01257229 | https://www.genecards.org/cgi-bin/carddisp.pl?gene=USP43 |
| RNF152 | Ring Finger Protein 152 | Protein Coding | 42 | GC18M061808 | 10.99821758 | https://www.genecards.org/cgi-bin/carddisp.pl?gene=RNF152 |
| ATG7 | Autophagy Related 7 | Protein Coding | 50 | GC03P014331 | 10.99603844 | https://www.genecards.org/cgi-bin/carddisp.pl?gene=ATG7 |
| PINK1 | PTEN Induced Kinase 1 | Protein Coding | 53 | GC01P020634 | 10.99510384 | https://www.genecards.org/cgi-bin/carddisp.pl?gene=PINK1 |
| RING1 | Ring Finger Protein 1 | Protein Coding | 49 | GC06P033208 | 10.96930027 | https://www.genecards.org/cgi-bin/carddisp.pl?gene=RING1 |
| SETX | Senataxin | Protein Coding | 49 | GC09M132261 | 10.92051888 | https://www.genecards.org/cgi-bin/carddisp.pl?gene=SETX |
| RNF19B | Ring Finger Protein 19B | Protein Coding | 41 | GC01M033488 | 10.90735149 | https://www.genecards.org/cgi-bin/carddisp.pl?gene=RNF19B |
| UBE2L5 | Ubiquitin Conjugating Enzyme E2 L5 | Protein Coding | 19 | GC13P030660 | 10.90523624 | https://www.genecards.org/cgi-bin/carddisp.pl?gene=UBE2L5 |
| RNF115 | Ring Finger Protein 115 | Protein Coding | 43 | GC01M145738 | 10.90282154 | https://www.genecards.org/cgi-bin/carddisp.pl?gene=RNF115 |
| DDB2 | Damage Specific DNA Binding Protein 2 | Protein Coding | 53 | GC11P047237 | 10.87335777 | https://www.genecards.org/cgi-bin/carddisp.pl?gene=DDB2 |
| PSMC1 | Proteasome 26S Subunit, ATPase 1 | Protein Coding | 49 | GC14P090256 | 10.85992527 | https://www.genecards.org/cgi-bin/carddisp.pl?gene=PSMC1 |
| RNF185 | Ring Finger Protein 185 | Protein Coding | 43 | GC22P031160 | 10.85266399 | https://www.genecards.org/cgi-bin/carddisp.pl?gene=RNF185 |
| FAU | FAU Ubiquitin Like And Ribosomal Protein S30 Fusion | Protein Coding | 44 | GC11M065120 | 10.83218479 | https://www.genecards.org/cgi-bin/carddisp.pl?gene=FAU |
| CHMP2B | Charged Multivesicular Body Protein 2B | Protein Coding | 51 | GC03P087227 | 10.82691002 | https://www.genecards.org/cgi-bin/carddisp.pl?gene=CHMP2B |
| ZNRF2 | Zinc And Ring Finger 2 | Protein Coding | 40 | GC07P030284 | 10.81381607 | https://www.genecards.org/cgi-bin/carddisp.pl?gene=ZNRF2 |
| MARCHF4 | Membrane Associated Ring-CH-Type Finger 4 | Protein Coding | 38 | GC02M216260 | 10.80830097 | https://www.genecards.org/cgi-bin/carddisp.pl?gene=MARCHF4 |
| USP39 | Ubiquitin Specific Peptidase 39 | Protein Coding | 44 | GC02P085973 | 10.80592537 | https://www.genecards.org/cgi-bin/carddisp.pl?gene=USP39 |
| LRSAM1 | Leucine Rich Repeat And Sterile Alpha Motif Containing 1 | Protein Coding | 47 | GC09P127451 | 10.79171181 | https://www.genecards.org/cgi-bin/carddisp.pl?gene=LRSAM1 |
| PELI2 | Pellino E3 Ubiquitin Protein Ligase Family Member 2 | Protein Coding | 43 | GC14P056117 | 10.7841053 | https://www.genecards.org/cgi-bin/carddisp.pl?gene=PELI2 |
| TRIM11 | Tripartite Motif Containing 11 | Protein Coding | 45 | GC01M228393 | 10.75950241 | https://www.genecards.org/cgi-bin/carddisp.pl?gene=TRIM11 |
| CDC27 | Cell Division Cycle 27 | Protein Coding | 50 | GC17M047117 | 10.74221897 | https://www.genecards.org/cgi-bin/carddisp.pl?gene=CDC27 |
| FBXO7 | F-Box Protein 7 | Protein Coding | 50 | GC22P032474 | 10.73581982 | https://www.genecards.org/cgi-bin/carddisp.pl?gene=FBXO7 |
| RNF43 | Ring Finger Protein 43 | Protein Coding | 46 | GC17M058352 | 10.72966385 | https://www.genecards.org/cgi-bin/carddisp.pl?gene=RNF43 |
| UBE2QL1 | Ubiquitin Conjugating Enzyme E2 Q Family Like 1 | Protein Coding | 36 | GC05P006448 | 10.72357941 | https://www.genecards.org/cgi-bin/carddisp.pl?gene=UBE2QL1 |
| SPOP | Speckle Type BTB/POZ Protein | Protein Coding | 51 | GC17M049598 | 10.71509075 | https://www.genecards.org/cgi-bin/carddisp.pl?gene=SPOP |
| RNF126 | Ring Finger Protein 126 | Protein Coding | 42 | GC19M000647 | 10.70098591 | https://www.genecards.org/cgi-bin/carddisp.pl?gene=RNF126 |
| TMEM129 | Transmembrane Protein 129, E3 Ubiquitin Ligase | Protein Coding | 38 | GC04M001715 | 10.69683456 | https://www.genecards.org/cgi-bin/carddisp.pl?gene=TMEM129 |
| RNF217 | Ring Finger Protein 217 | Protein Coding | 40 | GC06P124962 | 10.69258118 | https://www.genecards.org/cgi-bin/carddisp.pl?gene=RNF217 |
| UBFD1 | Ubiquitin Family Domain Containing 1 | Protein Coding | 40 | GC16P023557 | 10.68615723 | https://www.genecards.org/cgi-bin/carddisp.pl?gene=UBFD1 |
| PRPF19 | Pre-MRNA Processing Factor 19 | Protein Coding | 44 | GC11M060890 | 10.68554497 | https://www.genecards.org/cgi-bin/carddisp.pl?gene=PRPF19 |
| GSK3B | Glycogen Synthase Kinase 3 Beta | Protein Coding | 57 | GC03M119821 | 10.65645313 | https://www.genecards.org/cgi-bin/carddisp.pl?gene=GSK3B |
| USP51 | Ubiquitin Specific Peptidase 51 | Protein Coding | 37 | GC0XM055484 | 10.61720371 | https://www.genecards.org/cgi-bin/carddisp.pl?gene=USP51 |
| RAD23B | RAD23 Homolog B, Nucleotide Excision Repair Protein | Protein Coding | 51 | GC09P107283 | 10.61023521 | https://www.genecards.org/cgi-bin/carddisp.pl?gene=RAD23B |
| DTX1 | Deltex E3 Ubiquitin Ligase 1 | Protein Coding | 45 | GC12P113056 | 10.59988594 | https://www.genecards.org/cgi-bin/carddisp.pl?gene=DTX1 |
| TRIM5 | Tripartite Motif Containing 5 | Protein Coding | 48 | GC11M007529 | 10.57747746 | https://www.genecards.org/cgi-bin/carddisp.pl?gene=TRIM5 |
| RMND5A | Required For Meiotic Nuclear Division 5 Homolog A | Protein Coding | 45 | GC02P086721 | 10.56783772 | https://www.genecards.org/cgi-bin/carddisp.pl?gene=RMND5A |
| RNF181 | Ring Finger Protein 181 | Protein Coding | 40 | GC02P085988 | 10.5671196 | https://www.genecards.org/cgi-bin/carddisp.pl?gene=RNF181 |
| C9orf72 | C9orf72-SMCR8 Complex Subunit | Protein Coding | 47 | GC09M028760 | 10.56157207 | https://www.genecards.org/cgi-bin/carddisp.pl?gene=C9orf72 |
| RFWD3 | Ring Finger And WD Repeat Domain 3 | Protein Coding | 46 | GC16M074621 | 10.55560303 | https://www.genecards.org/cgi-bin/carddisp.pl?gene=RFWD3 |
| ERBB4 | Erb-B2 Receptor Tyrosine Kinase 4 | Protein Coding | 61 | GC02M211375 | 10.54681587 | https://www.genecards.org/cgi-bin/carddisp.pl?gene=ERBB4 |
| LOC100532749 | Ubiquitination Factor E4A (UFD2 Homolog, Yeast) Pseudogene | Pseudogene | 2 | GC03M112245 | 10.54193211 | https://www.genecards.org/cgi-bin/carddisp.pl?gene=LOC100532749 |
| PSMA6 | Proteasome 20S Subunit Alpha 6 | Protein Coding | 52 | GC14P035278 | 10.5408287 | https://www.genecards.org/cgi-bin/carddisp.pl?gene=PSMA6 |
| RNF167 | Ring Finger Protein 167 | Protein Coding | 44 | GC17P005953 | 10.51654339 | https://www.genecards.org/cgi-bin/carddisp.pl?gene=RNF167 |
| UFM1 | Ubiquitin Fold Modifier 1 | Protein Coding | 48 | GC13P038349 | 10.50078011 | https://www.genecards.org/cgi-bin/carddisp.pl?gene=UFM1 |
| TRIM28 | Tripartite Motif Containing 28 | Protein Coding | 51 | GC19P058544 | 10.5001955 | https://www.genecards.org/cgi-bin/carddisp.pl?gene=TRIM28 |
| PELI3 | Pellino E3 Ubiquitin Protein Ligase Family Member 3 | Protein Coding | 42 | GC11P066466 | 10.48686218 | https://www.genecards.org/cgi-bin/carddisp.pl?gene=PELI3 |
| SNCA | Synuclein Alpha | Protein Coding | 59 | GC04M089724 | 10.47774506 | https://www.genecards.org/cgi-bin/carddisp.pl?gene=SNCA |
| DCUN1D1 | Defective In Cullin Neddylation 1 Domain Containing 1 | Protein Coding | 45 | GC03M182938 | 10.44395447 | https://www.genecards.org/cgi-bin/carddisp.pl?gene=DCUN1D1 |
| TRIM71 | Tripartite Motif Containing 71 | Protein Coding | 47 | GC03P033029 | 10.42352676 | https://www.genecards.org/cgi-bin/carddisp.pl?gene=TRIM71 |
| HIF1A | Hypoxia Inducible Factor 1 Subunit Alpha | Protein Coding | 55 | GC14P061695 | 10.40226936 | https://www.genecards.org/cgi-bin/carddisp.pl?gene=HIF1A |
| USP42 | Ubiquitin Specific Peptidase 42 | Protein Coding | 44 | GC07P006078 | 10.40221024 | https://www.genecards.org/cgi-bin/carddisp.pl?gene=USP42 |
| PSMA5 | Proteasome 20S Subunit Alpha 5 | Protein Coding | 49 | GC01M109399 | 10.39825344 | https://www.genecards.org/cgi-bin/carddisp.pl?gene=PSMA5 |
| DET1 | DET1 Partner Of COP1 E3 Ubiquitin Ligase | Protein Coding | 39 | GC15M130122 | 10.356143 | https://www.genecards.org/cgi-bin/carddisp.pl?gene=DET1 |
| SH3RF1 | SH3 Domain Containing Ring Finger 1 | Protein Coding | 47 | GC04M169094 | 10.35443783 | https://www.genecards.org/cgi-bin/carddisp.pl?gene=SH3RF1 |
| HLTF | Helicase Like Transcription Factor | Protein Coding | 47 | GC03M149030 | 10.32648563 | https://www.genecards.org/cgi-bin/carddisp.pl?gene=HLTF |
| PSMD1 | Proteasome 26S Subunit, Non-ATPase 1 | Protein Coding | 47 | GC02P231056 | 10.32025528 | https://www.genecards.org/cgi-bin/carddisp.pl?gene=PSMD1 |
| PSMC6 | Proteasome 26S Subunit, ATPase 6 | Protein Coding | 46 | GC14P052707 | 10.31492615 | https://www.genecards.org/cgi-bin/carddisp.pl?gene=PSMC6 |
| SHARPIN | SHANK Associated RH Domain Interactor | Protein Coding | 46 | GC08M144098 | 10.28182411 | https://www.genecards.org/cgi-bin/carddisp.pl?gene=SHARPIN |
| EGFR | Epidermal Growth Factor Receptor | Protein Coding | 62 | GC07P055019 | 10.26973915 | https://www.genecards.org/cgi-bin/carddisp.pl?gene=EGFR |
| MKRN1 | Makorin Ring Finger Protein 1 | Protein Coding | 47 | GC07M140453 | 10.26172256 | https://www.genecards.org/cgi-bin/carddisp.pl?gene=MKRN1 |
| CDC23 | Cell Division Cycle 23 | Protein Coding | 47 | GC05M138198 | 10.26115704 | https://www.genecards.org/cgi-bin/carddisp.pl?gene=CDC23 |
| MARCHF3 | Membrane Associated Ring-CH-Type Finger 3 | Protein Coding | 36 | GC05M126869 | 10.25261497 | https://www.genecards.org/cgi-bin/carddisp.pl?gene=MARCHF3 |
| CAND1 | Cullin Associated And Neddylation Dissociated 1 | Protein Coding | 46 | GC12P067270 | 10.23664188 | https://www.genecards.org/cgi-bin/carddisp.pl?gene=CAND1 |
| NUB1 | Negative Regulator Of Ubiquitin Like Proteins 1 | Protein Coding | 44 | GC07P151341 | 10.23326969 | https://www.genecards.org/cgi-bin/carddisp.pl?gene=NUB1 |
| PSMA3 | Proteasome 20S Subunit Alpha 3 | Protein Coding | 52 | GC14P058244 | 10.23298645 | https://www.genecards.org/cgi-bin/carddisp.pl?gene=PSMA3 |
| CUL7 | Cullin 7 | Protein Coding | 47 | GC06M043037 | 10.16039276 | https://www.genecards.org/cgi-bin/carddisp.pl?gene=CUL7 |
| MATR3 | Matrin 3 | Protein Coding | 49 | GC05P139274 | 10.14552402 | https://www.genecards.org/cgi-bin/carddisp.pl?gene=MATR3 |
| NFE2L2 | NFE2 Like BZIP Transcription Factor 2 | Protein Coding | 58 | GC02M177227 | 10.12966537 | https://www.genecards.org/cgi-bin/carddisp.pl?gene=NFE2L2 |
| MARCHF10 | Membrane Associated Ring-CH-Type Finger 10 | Protein Coding | 39 | GC17M062704 | 10.08117485 | https://www.genecards.org/cgi-bin/carddisp.pl?gene=MARCHF10 |
| TTC3 | Tetratricopeptide Repeat Domain 3 | Protein Coding | 46 | GC21P037073 | 10.05251694 | https://www.genecards.org/cgi-bin/carddisp.pl?gene=TTC3 |
| UBASH3B | Ubiquitin Associated And SH3 Domain Containing B | Protein Coding | 48 | GC11P122655 | 10.02021122 | https://www.genecards.org/cgi-bin/carddisp.pl?gene=UBASH3B |
| PFN1 | Profilin 1 | Protein Coding | 53 | GC17M004945 | 10.00924778 | https://www.genecards.org/cgi-bin/carddisp.pl?gene=PFN1 |
| HDAC6 | Histone Deacetylase 6 | Protein Coding | 61 | GC0XP048801 | 10.00913715 | https://www.genecards.org/cgi-bin/carddisp.pl?gene=HDAC6 |
