# Supplementary material for: Integration of ubiquitination-related genes in predictive signatures for prognosis and immunotherapy response in sarcoma
Source: Front Oncol. 2024 Oct 14;14:1446522. doi: 10.3389/fonc.2024.1446522 (PMC11513255; doi:10.3389/fonc.2024.1446522)
Supplement: Supplementary file 1 [file DataSheet1.zip › Supplementary Table 3.docx]

**Supplementary Table 3. Immune related gene**

| Symbol | ID | Name | Synonyms | Chromosome | Category |
| --- | --- | --- | --- | --- | --- |
| AZGP1 | 563 | alpha-2-glycoprotein 1, zinc-binding | ZA2G\|ZAG | 7 | Antigen_Processing_and_Presentation |
| B2M | 567 | beta-2-microglobulin | IMD43 | 15 | Antigen_Processing_and_Presentation |
| CALR | 811 | calreticulin | CRT\|HEL-S-99n\|RO\|SSA\|cC1qR | 19 | Antigen_Processing_and_Presentation |
| CANX | 821 | calnexin | CNX\|IP90\|P90 | 5 | Antigen_Processing_and_Presentation |
| CD1A | 909 | CD1a molecule | CD1\|FCB6\|HTA1\|R4\|T6 | 1 | Antigen_Processing_and_Presentation |
| CD1B | 910 | CD1b molecule | CD1\|CD1A\|R1 | 1 | Antigen_Processing_and_Presentation |
| CD1C | 911 | CD1c molecule | BDCA1\|CD1\|CD1A\|R7 | 1 | Antigen_Processing_and_Presentation |
| CD1D | 912 | CD1d molecule | CD1A\|R3\|R3G1 | 1 | Antigen_Processing_and_Presentation |
| CD1E | 913 | CD1e molecule | CD1A\|R2 | 1 | Antigen_Processing_and_Presentation |
| CD4 | 920 | CD4 molecule | CD4mut | 12 | Antigen_Processing_and_Presentation |
| CD8A | 925 | CD8a molecule | CD8\|Leu2\|p32 | 2 | Antigen_Processing_and_Presentation |
| CD8B | 926 | CD8b molecule | CD8B1\|LEU2\|LY3\|LYT3\|P37 | 2 | Antigen_Processing_and_Presentation |
| CD74 | 972 | CD74 molecule | DHLAG\|HLADG\|II\|Ia-GAMMA\|p33 | 5 | Antigen_Processing_and_Presentation |
| CREB1 | 1385 | cAMP responsive element binding protein 1 | CREB\|CREB-1 | 2 | Antigen_Processing_and_Presentation |
| CTSB | 1508 | cathepsin B | APPS\|CPSB\|RECEUP | 8 | Antigen_Processing_and_Presentation |
| CTSE | 1510 | cathepsin E | CATE | 1 | Antigen_Processing_and_Presentation |
| CTSL | 1514 | cathepsin L | CATL\|CTSL1\|MEP | 9 | Antigen_Processing_and_Presentation |
| CTSS | 1520 | cathepsin S | - | 1 | Antigen_Processing_and_Presentation |
| FCER1G | 2207 | Fc fragment of IgE receptor Ig | FCRG | 1 | Antigen_Processing_and_Presentation |
| FCGRT | 2217 | Fc fragment of IgG receptor and transporter | FCRN\|alpha-chain | 19 | Antigen_Processing_and_Presentation |
| PDIA3 | 2923 | protein disulfide isomerase family A member 3 | ER60\|ERp57\|ERp60\|ERp61\|GRP57\|GRP58\|HEL-S-269\|HEL-S-93n\|HsT17083\|P58\|PI-PLC | 15 | Antigen_Processing_and_Presentation |
| HFE | 3077 | homeostatic iron regulator | HFE1\|HH\|HLA-H\|MVCD7\|TFQTL2 | 6 | Antigen_Processing_and_Presentation |
| HLA-A | 3105 | major histocompatibility complex, class I, A | HLAA | 6 | Antigen_Processing_and_Presentation |
| HLA-B | 3106 | major histocompatibility complex, class I, B | AS\|B-4901\|HLAB | 6 | Antigen_Processing_and_Presentation |
| HLA-C | 3107 | major histocompatibility complex, class I, C | D6S204\|HLA-JY3\|HLAC\|HLC-C\|MHC\|PSORS1 | 6 | Antigen_Processing_and_Presentation |
| HLA-DMA | 3108 | major histocompatibility complex, class II, DM alpha | D6S222E\|DMA\|HLADM\|RING6 | 6 | Antigen_Processing_and_Presentation |
| HLA-DMB | 3109 | major histocompatibility complex, class II, DM beta | D6S221E\|RING7 | 6 | Antigen_Processing_and_Presentation |
| HLA-DOA | 3111 | major histocompatibility complex, class II, DO alpha | HLA-DNA\|HLA-DZA\|HLADZ | 6 | Antigen_Processing_and_Presentation |
| HLA-DOB | 3112 | major histocompatibility complex, class II, DO beta | DOB\|HLA_DOB | 6 | Antigen_Processing_and_Presentation |
| HLA-DPA1 | 3113 | major histocompatibility complex, class II, DP alpha 1 | DP(W3)\|DP(W4)\|DPA1\|HLA-DP1A\|HLA-DPB1\|HLADP\|HLASB\|PLT1 | 6 | Antigen_Processing_and_Presentation |
| HLA-DPB1 | 3115 | major histocompatibility complex, class II, DP beta 1 | DPB1\|HLA-DP\|HLA-DP1B\|HLA-DPB | 6 | Antigen_Processing_and_Presentation |
| HLA-DQA1 | 3117 | major histocompatibility complex, class II, DQ alpha 1 | CELIAC1\|DQ-A1\|DQA1\|HLA-DQA | 6 | Antigen_Processing_and_Presentation |
| HLA-DQA2 | 3118 | major histocompatibility complex, class II, DQ alpha 2 | DC-alpha\|DX-ALPHA\|HLA-DCA\|HLA-DXA\|HLADQA2 | 6 | Antigen_Processing_and_Presentation |
| HLA-DQB1 | 3119 | major histocompatibility complex, class II, DQ beta 1 | CELIAC1\|HLA-DQB\|IDDM1 | 6 | Antigen_Processing_and_Presentation |
| HLA-DRA | 3122 | major histocompatibility complex, class II, DR alpha | HLA-DRA1 | 6 | Antigen_Processing_and_Presentation |
| HLA-DRB1 | 3123 | major histocompatibility complex, class II, DR beta 1 | DRB1\|HLA-DR1B\|HLA-DRB\|SS1 | 6 | Antigen_Processing_and_Presentation |
| HLA-DRB3 | 3125 | major histocompatibility complex, class II, DR beta 3 | DRB3\|HLA-DPB1\|HLA-DR1B\|HLA-DR3B | 6 | Antigen_Processing_and_Presentation |
| HLA-DRB4 | 3126 | major histocompatibility complex, class II, DR beta 4 | DR4\|DRB4\|HLA-DR4B\|HLA-DRB4* | 6 | Antigen_Processing_and_Presentation |
| HLA-DRB5 | 3127 | major histocompatibility complex, class II, DR beta 5 | - | 6 | Antigen_Processing_and_Presentation |
| HLA-E | 3133 | major histocompatibility complex, class I, E | HLA-6.2\|QA1 | 6 | Antigen_Processing_and_Presentation |
| HLA-F | 3134 | major histocompatibility complex, class I, F | CDA12\|HLA-5.4\|HLA-CDA12\|HLAF | 6 | Antigen_Processing_and_Presentation |
| HLA-G | 3135 | major histocompatibility complex, class I, G | MHC-G | 6 | Antigen_Processing_and_Presentation |
| HLA-H | 3136 | major histocompatibility complex, class I, H (pseudogene) | HLAHP | 6 | Antigen_Processing_and_Presentation |
| MR1 | 3140 | major histocompatibility complex, class I-related | HLALS | 1 | Antigen_Processing_and_Presentation |
| HSPA1A | 3303 | heat shock protein family A (Hsp70) member 1A | HEL-S-103\|HSP70-1\|HSP70-1A\|HSP70-2\|HSP70.1\|HSP70.2\|HSP70I\|HSP72\|HSPA1 | 6 | Antigen_Processing_and_Presentation |
| HSPA1B | 3304 | heat shock protein family A (Hsp70) member 1B | HSP70-1\|HSP70-1B\|HSP70-2\|HSP70.1\|HSP70.2\|HSP72\|HSPA1\|HSX70 | 6 | Antigen_Processing_and_Presentation |
| HSPA1L | 3305 | heat shock protein family A (Hsp70) member 1 like | HSP70-1L\|HSP70-HOM\|HSP70T\|hum70t | 6 | Antigen_Processing_and_Presentation |
| HSPA2 | 3306 | heat shock protein family A (Hsp70) member 2 | HSP70-2\|HSP70-3 | 14 | Antigen_Processing_and_Presentation |
| HSPA4 | 3308 | heat shock protein family A (Hsp70) member 4 | APG-2\|HEL-S-5a\|HS24/P52\|HSPH2\|RY\|hsp70\|hsp70RY | 5 | Antigen_Processing_and_Presentation |
| HSPA5 | 3309 | heat shock protein family A (Hsp70) member 5 | BIP\|GRP78\|HEL-S-89n\|MIF2 | 9 | Antigen_Processing_and_Presentation |
| HSPA6 | 3310 | heat shock protein family A (Hsp70) member 6 | HSP70B' | 1 | Antigen_Processing_and_Presentation |
| HSPA8 | 3312 | heat shock protein family A (Hsp70) member 8 | HEL-33\|HEL-S-72p\|HSC54\|HSC70\|HSC71\|HSP71\|HSP73\|HSPA10\|LAP-1\|LAP1\|NIP71 | 11 | Antigen_Processing_and_Presentation |
| HSP90AA1 | 3320 | heat shock protein 90 alpha family class A member 1 | EL52\|HEL-S-65p\|HSP86\|HSP89A\|HSP90A\|HSP90N\|HSPC1\|HSPCA\|HSPCAL1\|HSPCAL4\|HSPN\|Hsp103\|Hsp89\|Hsp90\|LAP-2\|LAP2 | 14 | Antigen_Processing_and_Presentation |
| HSP90AB1 | 3326 | heat shock protein 90 alpha family class B member 1 | D6S182\|HSP84\|HSP90B\|HSPC2\|HSPCB | 6 | Antigen_Processing_and_Presentation |
| ICAM1 | 3383 | intercellular adhesion molecule 1 | BB2\|CD54\|P3.58 | 19 | Antigen_Processing_and_Presentation |
| IFNA1 | 3439 | interferon alpha 1 | IFL\|IFN\|IFN-ALPHA\|IFN-alphaD\|IFNA13\|IFNA@\|leIF D | 9 | Antigen_Processing_and_Presentation |
| IFNA2 | 3440 | interferon alpha 2 | IFN-alpha-2\|IFN-alphaA\|IFNA\|IFNA2B\|leIF A | 9 | Antigen_Processing_and_Presentation |
| IFNA4 | 3441 | interferon alpha 4 | IFN-alpha4a\|INFA4 | 9 | Antigen_Processing_and_Presentation |
| IFNA5 | 3442 | interferon alpha 5 | IFN-alpha-5\|IFN-alphaG\|INA5\|INFA5\|leIF G | 9 | Antigen_Processing_and_Presentation |
| IFNA6 | 3443 | interferon alpha 6 | IFN-alphaK | 9 | Antigen_Processing_and_Presentation |
| IFNA7 | 3444 | interferon alpha 7 | IFN-alphaJ\|IFNA-J | 9 | Antigen_Processing_and_Presentation |
| IFNA8 | 3445 | interferon alpha 8 | IFN-alphaB | 9 | Antigen_Processing_and_Presentation |
| IFNA10 | 3446 | interferon alpha 10 | IFN-alphaC | 9 | Antigen_Processing_and_Presentation |
| IFNA13 | 3447 | interferon alpha 13 | - | 9 | Antigen_Processing_and_Presentation |
| IFNA14 | 3448 | interferon alpha 14 | IFN-alphaH\|LEIF2H | 9 | Antigen_Processing_and_Presentation |
| IFNA16 | 3449 | interferon alpha 16 | IFN-alpha-16\|IFN-alphaO | 9 | Antigen_Processing_and_Presentation |
| IFNA17 | 3451 | interferon alpha 17 | IFN-alphaI\|IFNA\|INFA\|LEIF2C1 | 9 | Antigen_Processing_and_Presentation |
| IFNA21 | 3452 | interferon alpha 21 | IFN-alphaI\|LeIF F\|leIF-F | 9 | Antigen_Processing_and_Presentation |
| IFNG | 3458 | interferon gamma | IFG\|IFI | 12 | Antigen_Processing_and_Presentation |
| KIR2DL1 | 3802 | killer cell immunoglobulin like receptor, two Ig domains and long cytoplasmic tail 1 | CD158A\|KIR-K64\|KIR221\|KIR2DL3\|NKAT\|NKAT-1\|NKAT1\|p58.1 | 19 | Antigen_Processing_and_Presentation |
| KIR2DL2 | 3803 | killer cell immunoglobulin like receptor, two Ig domains and long cytoplasmic tail 2 | CD158B1\|CD158b\|NKAT-6\|NKAT6\|p58.2 | 19 | Antigen_Processing_and_Presentation |
| KIR2DL3 | 3804 | killer cell immunoglobulin like receptor, two Ig domains and long cytoplasmic tail 3 | CD158B2\|CD158b\|GL183\|KIR-023GB\|KIR-K7b\|KIR-K7c\|KIR2DL\|KIR2DS5\|KIRCL23\|NKAT\|NKAT2\|NKAT2A\|NKAT2B\|p58 | 19 | Antigen_Processing_and_Presentation |
| KIR2DL4 | 3805 | killer cell immunoglobulin like receptor, two Ig domains and long cytoplasmic tail 4 | CD158D\|G9P\|KIR-103AS\|KIR-2DL4\|KIR103\|KIR103AS | 19 | Antigen_Processing_and_Presentation |
| KIR2DS1 | 3806 | killer cell immunoglobulin like receptor, two Ig domains and short cytoplasmic tail 1 | CD158H\|CD158a\|p50.1 | 19 | Antigen_Processing_and_Presentation |
| KIR2DS3 | 3808 | killer cell immunoglobulin like receptor, two Ig domains and short cytoplasmic tail 3 | NKAT7 | 19 | Antigen_Processing_and_Presentation |
| KIR2DS4 | 3809 | killer cell immunoglobulin like receptor, two Ig domains and short cytoplasmic tail 4 | CD158I\|KIR-2DS4\|KIR1D\|KIR412\|KKA3\|NKAT-8\|NKAT8 | 19 | Antigen_Processing_and_Presentation |
| KIR2DS5 | 3810 | killer cell immunoglobulin like receptor, two Ig domains and short cytoplasmic tail 5 | CD158G\|NKAT9 | 19 | Antigen_Processing_and_Presentation |
| KIR3DL1 | 3811 | killer cell immunoglobulin like receptor, three Ig domains and long cytoplasmic tail 1 | CD158E1\|KIR\|KIR3DL1/S1\|NKAT-3\|NKAT3\|NKB1\|NKB1B | 19 | Antigen_Processing_and_Presentation |
| KIR3DL2 | 3812 | killer cell immunoglobulin like receptor, three Ig domains and long cytoplasmic tail 2 | 3DL2\|CD158K\|KIR-3DL2\|NKAT-4\|NKAT4\|NKAT4B\|p140 | 19 | Antigen_Processing_and_Presentation |
| KLRC1 | 3821 | killer cell lectin like receptor C1 | CD159A\|NKG2\|NKG2A | 12 | Antigen_Processing_and_Presentation |
| KLRC2 | 3822 | killer cell lectin like receptor C2 | CD159c\|NKG2-C\|NKG2C | 12 | Antigen_Processing_and_Presentation |
| KLRC3 | 3823 | killer cell lectin like receptor C3 | NKG2-E\|NKG2E | 12 | Antigen_Processing_and_Presentation |
| KLRD1 | 3824 | killer cell lectin like receptor D1 | CD94 | 12 | Antigen_Processing_and_Presentation |
| LTA | 4049 | lymphotoxin alpha | LT\|TNFB\|TNFSF1\|TNLG1E | 6 | Antigen_Processing_and_Presentation |
| CIITA | 4261 | class II major histocompatibility complex transactivator | C2TA\|CIITAIV\|MHC2TA\|NLRA | 16 | Antigen_Processing_and_Presentation |
| MICA | 100507436 | MHC class I polypeptide-related sequence A | MIC-A\|PERB11.1 | 6 | Antigen_Processing_and_Presentation |
| MICB | 4277 | MHC class I polypeptide-related sequence B | PERB11.2 | 6 | Antigen_Processing_and_Presentation |
| NFYA | 4800 | nuclear transcription factor Y subunit alpha | CBF-A\|CBF-B\|HAP2\|NF-YA | 6 | Antigen_Processing_and_Presentation |
| NFYB | 4801 | nuclear transcription factor Y subunit beta | CBF-A\|CBF-B\|HAP3\|NF-YB | 12 | Antigen_Processing_and_Presentation |
| NFYC | 4802 | nuclear transcription factor Y subunit gamma | CBF-C\|CBFC\|H1TF2A\|HAP5\|HSM\|NF-YC | 1 | Antigen_Processing_and_Presentation |
| LGMN | 5641 | legumain | AEP\|LGMN1\|PRSC1 | 14 | Antigen_Processing_and_Presentation |
| PSMB8 | 5696 | proteasome 20S subunit beta 8 | ALDD\|D6S216\|D6S216E\|JMP\|LMP7\|NKJO\|PRAAS1\|PSMB5i\|RING10 | 6 | Antigen_Processing_and_Presentation |
| PSMC1 | 5700 | proteasome 26S subunit, ATPase 1 | P26S4\|S4\|p56 | 14 | Antigen_Processing_and_Presentation |
| PSMC2 | 5701 | proteasome 26S subunit, ATPase 2 | MSS1\|Nbla10058\|S7 | 7 | Antigen_Processing_and_Presentation |
| PSMC3 | 5702 | proteasome 26S subunit, ATPase 3 | TBP1 | 11 | Antigen_Processing_and_Presentation |
| PSMC4 | 5704 | proteasome 26S subunit, ATPase 4 | MIP224\|RPT3\|S6\|TBP-7\|TBP7 | 19 | Antigen_Processing_and_Presentation |
| PSMC5 | 5705 | proteasome 26S subunit, ATPase 5 | S8\|SUG-1\|SUG1\|TBP10\|TRIP1\|p45\|p45/SUG | 17 | Antigen_Processing_and_Presentation |
| PSMC6 | 5706 | proteasome 26S subunit, ATPase 6 | SUG2\|p42 | 14 | Antigen_Processing_and_Presentation |
| PSMD1 | 5707 | proteasome 26S subunit, non-ATPase 1 | P112\|Rpn2\|S1 | 2 | Antigen_Processing_and_Presentation |
| PSMD2 | 5708 | proteasome 26S subunit, non-ATPase 2 | P97\|RPN1\|S2\|TRAP2 | 3 | Antigen_Processing_and_Presentation |
| PSMD3 | 5709 | proteasome 26S subunit, non-ATPase 3 | P58\|RPN3\|S3\|TSTA2 | 17 | Antigen_Processing_and_Presentation |
| PSMD4 | 5710 | proteasome 26S subunit, non-ATPase 4 | AF\|AF-1\|ASF\|MCB1\|Rpn10\|S5A\|pUB-R5 | 1 | Antigen_Processing_and_Presentation |
| PSMD5 | 5711 | proteasome 26S subunit, non-ATPase 5 | S5B | 9 | Antigen_Processing_and_Presentation |
| PSMD7 | 5713 | proteasome 26S subunit, non-ATPase 7 | MOV34\|P40\|Rpn8\|S12 | 16 | Antigen_Processing_and_Presentation |
| PSMD8 | 5714 | proteasome 26S subunit, non-ATPase 8 | HEL-S-91n\|HIP6\|HYPF\|Nin1p\|Rpn12\|S14\|p31 | 19 | Antigen_Processing_and_Presentation |
| PSMD10 | 5716 | proteasome 26S subunit, non-ATPase 10 | dJ889N15.2\|p28\|p28(GANK) | X | Antigen_Processing_and_Presentation |
| PSMD11 | 5717 | proteasome 26S subunit, non-ATPase 11 | Rpn6\|S9\|p44.5 | 17 | Antigen_Processing_and_Presentation |
| PSMD13 | 5719 | proteasome 26S subunit, non-ATPase 13 | HSPC027\|Rpn9\|S11\|p40.5 | 11 | Antigen_Processing_and_Presentation |
| PSME1 | 5720 | proteasome activator subunit 1 | HEL-S-129m\|IFI5111\|PA28A\|PA28alpha\|REGalpha | 14 | Antigen_Processing_and_Presentation |
| PSME2 | 5721 | proteasome activator subunit 2 | PA28B\|PA28beta\|REGbeta | 14 | Antigen_Processing_and_Presentation |
| RELB | 5971 | RELB proto-oncogene, NF-kB subunit | I-REL\|IMD53\|IREL\|REL-B | 19 | Antigen_Processing_and_Presentation |
| RFX5 | 5993 | regulatory factor X5 | - | 1 | Antigen_Processing_and_Presentation |
| RFXAP | 5994 | regulatory factor X associated protein | - | 13 | Antigen_Processing_and_Presentation |
| SLC10A2 | 6555 | solute carrier family 10 member 2 | ASBT\|IBAT\|ISBT\|NTCP2\|PBAM | 13 | Antigen_Processing_and_Presentation |
| TAP1 | 6890 | transporter 1, ATP binding cassette subfamily B member | ABC17\|ABCB2\|APT1\|D6S114E\|PSF-1\|PSF1\|RING4\|TAP1*0102N\|TAP1N | 6 | Antigen_Processing_and_Presentation |
| TAP2 | 6891 | transporter 2, ATP binding cassette subfamily B member | ABC18\|ABCB3\|APT2\|D6S217E\|PSF-2\|PSF2\|RING11 | 6 | Antigen_Processing_and_Presentation |
| TAPBP | 6892 | TAP binding protein | NGS17\|TAPA\|TPN\|TPSN | 6 | Antigen_Processing_and_Presentation |
| THBS1 | 7057 | thrombospondin 1 | THBS\|THBS-1\|TSP\|TSP-1\|TSP1 | 15 | Antigen_Processing_and_Presentation |
| SEM1 | 7979 | SEM1 26S proteasome complex subunit | C7orf76\|DSS1\|ECD\|SHFD1\|SHFM1\|SHSF1\|Shfdg1 | 7 | Antigen_Processing_and_Presentation |
| KLRC4 | 8302 | killer cell lectin like receptor C4 | NKG2-F\|NKG2F | 12 | Antigen_Processing_and_Presentation |
| AP3B1 | 8546 | adaptor related protein complex 3 subunit beta 1 | ADTB3\|ADTB3A\|HPS\|HPS2\|PE | 5 | Antigen_Processing_and_Presentation |
| RFXANK | 8625 | regulatory factor X associated ankyrin containing protein | ANKRA1\|BLS\|F14150_1\|RFX-B | 19 | Antigen_Processing_and_Presentation |
| PSMD6 | 9861 | proteasome 26S subunit, non-ATPase 6 | Rpn7\|S10\|SGA-113M\|p42A\|p44S10 | 3 | Antigen_Processing_and_Presentation |
| PSME3 | 10197 | proteasome activator subunit 3 | HEL-S-283\|Ki\|PA28-gamma\|PA28G\|PA28gamma\|REG-GAMMA | 17 | Antigen_Processing_and_Presentation |
| PSMD14 | 10213 | proteasome 26S subunit, non-ATPase 14 | PAD1\|POH1\|RPN11 | 2 | Antigen_Processing_and_Presentation |
| CLEC4M | 10332 | C-type lectin domain family 4 member M | CD209L\|CD299\|DC-SIGN2\|DC-SIGNR\|DCSIGNR\|HP10347\|L-SIGN\|LSIGN | 19 | Antigen_Processing_and_Presentation |
| IFI30 | 10437 | IFI30 lysosomal thiol reductase | GILT\|IFI-30\|IP-30\|IP30 | 19 | Antigen_Processing_and_Presentation |
| PROCR | 10544 | protein C receptor | CCCA\|CCD41\|EPCR | 20 | Antigen_Processing_and_Presentation |
| ADRM1 | 11047 | adhesion regulating molecule 1 | ARM-1\|ARM1\|GP110 | 20 | Antigen_Processing_and_Presentation |
| ECPAS | 23392 | Ecm29 proteasome adaptor and scaffold | ECM29\|KIAA0368 | 9 | Antigen_Processing_and_Presentation |
| TRPC4AP | 26133 | transient receptor potential cation channel subfamily C member 4 associated protein | C20orf188\|PPP1R158\|TRRP4AP\|TRUSS | 20 | Antigen_Processing_and_Presentation |
| CD209 | 30835 | CD209 molecule | CDSIGN\|CLEC4L\|DC-SIGN\|DC-SIGN1 | 19 | Antigen_Processing_and_Presentation |
| UBXN1 | 51035 | UBX domain protein 1 | 2B28\|SAKS1\|UBXD10 | 11 | Antigen_Processing_and_Presentation |
| ERAP1 | 51752 | endoplasmic reticulum aminopeptidase 1 | A-LAP\|ALAP\|APPILS\|ARTS-1\|ARTS1\|ERAAP\|ERAAP1\|PILS-AP\|PILSAP | 5 | Antigen_Processing_and_Presentation |
| TAPBPL | 55080 | TAP binding protein like | TAPBP-R\|TAPBPR | 12 | Antigen_Processing_and_Presentation |
| KIR2DL5A | 57292 | killer cell immunoglobulin like receptor, two Ig domains and long cytoplasmic tail 5A | CD158F\|KIR2DL5\|KIR2DL5.1\|KIR2DL5.3 | 19 | Antigen_Processing_and_Presentation |
| ERAP2 | 64167 | endoplasmic reticulum aminopeptidase 2 | L-RAP\|LRAP | 5 | Antigen_Processing_and_Presentation |
| ULBP3 | 79465 | UL16 binding protein 3 | N2DL-3\|NKG2DL3\|RAET1N | 6 | Antigen_Processing_and_Presentation |
| ULBP2 | 80328 | UL16 binding protein 2 | ALCAN-alpha\|N2DL2\|NKG2DL2\|RAET1H\|RAET1L | 6 | Antigen_Processing_and_Presentation |
| ULBP1 | 80329 | UL16 binding protein 1 | N2DL-1\|NKG2DL1\|RAET1I | 6 | Antigen_Processing_and_Presentation |
| KIR3DL3 | 115653 | killer cell immunoglobulin like receptor, three Ig domains and long cytoplasmic tail 3 | CD158Z\|KIR3DL7\|KIR44\|KIRC1 | 19 | Antigen_Processing_and_Presentation |
| RAET1E | 135250 | retinoic acid early transcript 1E | LETAL\|N2DL-4\|NKG2DL4\|RAET1E2\|RL-4\|ULBP4\|bA350J20.7 | 6 | Antigen_Processing_and_Presentation |
| RAET1L | 154064 | retinoic acid early transcript 1L | ULBP6 | 6 | Antigen_Processing_and_Presentation |
| UBR1 | 197131 | ubiquitin protein ligase E3 component n-recognin 1 | JBS | 15 | Antigen_Processing_and_Presentation |
| RAET1G | 353091 | retinoic acid early transcript 1G | ULBP5 | 6 | Antigen_Processing_and_Presentation |
| PDIA2 | 64714 | protein disulfide isomerase family A member 2 | PDA2\|PDI\|PDIP\|PDIR | 16 | Antigen_Processing_and_Presentation |
| HAMP | 57817 | hepcidin antimicrobial peptide | HEPC\|HFE2B\|LEAP1\|PLTR | 19 | Antimicrobials |
| PI3 | 5266 | peptidase inhibitor 3 | ESI\|SKALP\|WAP3\|WFDC14\|cementoin | 20 | Antimicrobials |
| CAMP | 820 | cathelicidin antimicrobial peptide | CAP-18\|CAP18\|CRAMP\|FALL-39\|FALL39\|HSD26\|LL37 | 3 | Antimicrobials |
| DEFB4A | 1673 | defensin beta 4A | BD-2\|DEFB-2\|DEFB102\|DEFB2\|DEFB4\|HBD-2\|SAP1 | 8 | Antimicrobials |
| PPBP | 5473 | pro-platelet basic protein | B-TG1\|Beta-TG\|CTAP-III\|CTAP3\|CTAPIII\|CXCL7\|LA-PF4\|LDGF\|MDGF\|NAP-2\|PBP\|SCYB7\|TC1\|TC2\|TGB\|TGB1\|THBGB\|THBGB1 | 4 | Antimicrobials |
| REG3G | 130120 | regenerating family member 3 gamma | LPPM429\|PAP IB\|PAP-1B\|PAP1B\|PAPIB\|REG III\|REG-III\|UNQ429 | 2 | Antimicrobials |
| CXCL14 | 9547 | C-X-C motif chemokine ligand 14 | BMAC\|BRAK\|KEC\|KS1\|MIP-2g\|MIP2G\|NJAC\|SCYB14 | 5 | Antimicrobials |
| CXCL16 | 58191 | C-X-C motif chemokine ligand 16 | CXCLG16\|SR-PSOX\|SRPSOX | 17 | Antimicrobials |
| SLPI | 6590 | secretory leukocyte peptidase inhibitor | ALK1\|ALP\|BLPI\|HUSI\|HUSI-I\|MPI\|WAP4\|WFDC4 | 20 | Antimicrobials |
| CXCL8 | 3576 | C-X-C motif chemokine ligand 8 | GCP-1\|GCP1\|IL8\|LECT\|LUCT\|LYNAP\|MDNCF\|MONAP\|NAF\|NAP-1\|NAP1\|SCYB8 | 4 | Antimicrobials |
| CXCL10 | 3627 | C-X-C motif chemokine ligand 10 | C7\|IFI10\|INP10\|IP-10\|SCYB10\|crg-2\|gIP-10\|mob-1 | 4 | Antimicrobials |
| CXCL9 | 4283 | C-X-C motif chemokine ligand 9 | CMK\|Humig\|MIG\|SCYB9\|crg-10 | 4 | Antimicrobials |
| CXCL5 | 6374 | C-X-C motif chemokine ligand 5 | ENA-78\|SCYB5 | 4 | Antimicrobials |
| CXCL11 | 6373 | C-X-C motif chemokine ligand 11 | H174\|I-TAC\|IP-9\|IP9\|SCYB11\|SCYB9B\|b-R1 | 4 | Antimicrobials |
| CXCL6 | 6372 | C-X-C motif chemokine ligand 6 | CKA-3\|GCP-2\|GCP2\|SCYB6 | 4 | Antimicrobials |
| CXCL1 | 2919 | C-X-C motif chemokine ligand 1 | FSP\|GRO1\|GROa\|MGSA\|MGSA-a\|NAP-3\|SCYB1 | 4 | Antimicrobials |
| CXCL12 | 6387 | C-X-C motif chemokine ligand 12 | IRH\|PBSF\|SCYB12\|SDF1\|TLSF\|TPAR1 | 10 | Antimicrobials |
| CXCL13 | 10563 | C-X-C motif chemokine ligand 13 | ANGIE\|ANGIE2\|BCA-1\|BCA1\|BLC\|BLR1L\|SCYB13 | 4 | Antimicrobials |
| CXCL2 | 2920 | C-X-C motif chemokine ligand 2 | CINC-2a\|GRO2\|GROb\|MGSA-b\|MIP-2a\|MIP2\|MIP2A\|SCYB2 | 4 | Antimicrobials |
| PF4 | 5196 | platelet factor 4 | CXCL4\|PF-4\|SCYB4 | 4 | Antimicrobials |
| XCL1 | 6375 | X-C motif chemokine ligand 1 | ATAC\|LPTN\|LTN\|SCM-1\|SCM-1a\|SCM1\|SCM1A\|SCYC1 | 1 | Antimicrobials |
| CXCL3 | 2921 | C-X-C motif chemokine ligand 3 | CINC-2b\|GRO3\|GROg\|MIP-2b\|MIP2B\|SCYB3 | 4 | Antimicrobials |
| DEFB103B | 55894 | defensin beta 103B | BD-3\|DEFB-3\|DEFB103\|DEFB3\|HBD-3\|HBD3\|HBP-3\|HBP3 | 8 | Antimicrobials |
| CCL13 | 6357 | C-C motif chemokine ligand 13 | CKb10\|MCP-4\|NCC-1\|NCC1\|SCYA13\|SCYL1 | 17 | Antimicrobials |
| CCL1 | 6346 | C-C motif chemokine ligand 1 | I-309\|P500\|SCYA1\|SISe\|TCA3 | 17 | Antimicrobials |
| DEFB1 | 1672 | defensin beta 1 | BD1\|DEFB-1\|DEFB101\|HBD1 | 8 | Antimicrobials |
| CCL8 | 6355 | C-C motif chemokine ligand 8 | HC14\|MCP-2\|MCP2\|SCYA10\|SCYA8 | 17 | Antimicrobials |
| ELANE | 1991 | elastase, neutrophil expressed | ELA2\|GE\|HLE\|HNE\|NE\|PMN-E\|SCN1 | 19 | Antimicrobials |
| DEFB103A | 414325 | defensin beta 103A | BD-3\|DEFB-3\|DEFB103\|DEFB3\|HBD3\|HBP-3\|HBP3 | 8 | Antimicrobials |
| DEFA3 | 1668 | defensin alpha 3 | DEF3\|HNP-3\|HNP3\|HP-3\|HP3 | 8 | Antimicrobials |
| DEFA1 | 1667 | defensin alpha 1 | DEF1\|DEFA2\|HNP-1\|HP-1\|HP1\|MRS | 8 | Antimicrobials |
| TMSB10 | 9168 | thymosin beta 10 | MIG12\|TB10 | 2 | Antimicrobials |
| DEFA6 | 1671 | defensin alpha 6 | DEF6\|HD-6 | 8 | Antimicrobials |
| DEFA5 | 1670 | defensin alpha 5 | DEF5\|HD-5 | 8 | Antimicrobials |
| DEFA4 | 1669 | defensin alpha 4 | DEF4\|HNP-4\|HP-4\|HP4 | 8 | Antimicrobials |
| LCN2 | 3934 | lipocalin 2 | 24p3\|MSFI\|NGAL\|p25 | 9 | Antimicrobials |
| LCN1 | 3933 | lipocalin 1 | PMFA\|TLC\|TP\|VEGP | 9 | Antimicrobials |
| COLEC10 | 10584 | collectin subfamily member 10 | 3MC3\|CL-34\|CLL1 | 8 | Antimicrobials |
| BPI | 671 | bactericidal permeability increasing protein | BPIFD1\|rBPI | 20 | Antimicrobials |
| S100A9 | 6280 | S100 calcium binding protein A9 | 60B8AG\|CAGB\|CFAG\|CGLB\|L1AG\|LIAG\|MAC387\|MIF\|MRP14\|NIF\|P14 | 1 | Antimicrobials |
| S100A8 | 6279 | S100 calcium binding protein A8 | 60B8AG\|CAGA\|CFAG\|CGLA\|CP-10\|L1Ag\|MA387\|MIF\|MRP8\|NIF\|P8 | 1 | Antimicrobials |
| DCD | 117159 | dermcidin | AIDD\|DCD-1\|DSEP\|HCAP\|PIF | 12 | Antimicrobials |
| LCN6 | 158062 | lipocalin 6 | LCN5\|UNQ643\|hLcn5 | 9 | Antimicrobials |
| S100A12 | 6283 | S100 calcium binding protein A12 | CAAF1\|CAGC\|CGRP\|ENRAGE\|MRP-6\|MRP6\|p6 | 1 | Antimicrobials |
| HTN3 | 3347 | histatin 3 | HIS2\|HTN2\|HTN5\|PB | 4 | Antimicrobials |
| LCN8 | 138307 | lipocalin 8 | EP17\|LCN5 | 9 | Antimicrobials |
| DEFA1B | 728358 | defensin alpha 1B | HNP-1\|HP-1\|HP1 | 8 | Antimicrobials |
| CCR10 | 2826 | C-C motif chemokine receptor 10 | GPR2 | 17 | Antimicrobials |
| CELA1 | 1990 | chymotrypsin like elastase 1 | ELA1 | 12 | Antimicrobials |
| DEFB106A | 245909 | defensin beta 106A | BD-6\|DEFB-6\|DEFB106 | 8 | Antimicrobials |
| PENK | 5179 | proenkephalin | PE\|PENK-A | 8 | Antimicrobials |
| BPIFC | 254240 | BPI fold containing family C | BPIL2 | 22 | Antimicrobials |
| MMP12 | 4321 | matrix metallopeptidase 12 | HME\|ME\|MME\|MMP-12 | 11 | Antimicrobials |
| BPIFB6 | 128859 | BPI fold containing family B member 6 | BPIL3\|LPLUNC6 | 20 | Antimicrobials |
| LEAP2 | 116842 | liver enriched antimicrobial peptide 2 | LEAP-2 | 5 | Antimicrobials |
| SFTPD | 6441 | surfactant protein D | COLEC7\|PSP-D\|SFTP4\|SP-D | 10 | Antimicrobials |
| LCN9 | 392399 | lipocalin 9 | HEL129 | 9 | Antimicrobials |
| BPIFB2 | 80341 | BPI fold containing family B member 2 | BPIL1\|C20orf184\|LPLUNC2\|RYSR\|dJ726C3.2 | 20 | Antimicrobials |
| PTGDS | 5730 | prostaglandin D2 synthase | L-PGDS\|LPGDS\|PDS\|PGD2\|PGDS\|PGDS2 | 9 | Antimicrobials |
| TMSB4X | 7114 | thymosin beta 4 X-linked | FX\|PTMB4\|TB4X\|TMSB4 | X | Antimicrobials |
| PGLYRP1 | 8993 | peptidoglycan recognition protein 1 | PGLYRP\|PGRP\|PGRP-S\|PGRPS\|TAG7\|TNFSF3L | 19 | Antimicrobials |
| ZC3HAV1 | 56829 | zinc finger CCCH-type containing, antiviral 1 | ARTD13\|FLB6421\|PARP13\|ZAP\|ZC3H2\|ZC3HDC2 | 7 | Antimicrobials |
| TMSB15A | 11013 | thymosin beta 15a | TMSB15\|TMSB15B\|TMSL8\|TMSNB\|Tb15\|TbNB | X | Antimicrobials |
| S100B | 6285 | S100 calcium binding protein B | NEF\|S100\|S100-B\|S100beta | 21 | Antimicrobials |
| S100A13 | 6284 | S100 calcium binding protein A13 | - | 1 | Antimicrobials |
| S100A6 | 6277 | S100 calcium binding protein A6 | 2A9\|5B10\|CABP\|CACY\|PRA\|S10A6 | 1 | Antimicrobials |
| DEFB119 | 245932 | defensin beta 119 | DEFB-19\|DEFB-20\|DEFB120\|DEFB20\|ESC42-RELA\|ESC42-RELB | 20 | Antimicrobials |
| DEFB107A | 245910 | defensin beta 107A | BD-7\|DEFB-7\|DEFB107 | 8 | Antimicrobials |
| DEFB105A | 245908 | defensin beta 105A | BD-5\|DEFB-5\|DEFB105 | 8 | Antimicrobials |
| SERPIND1 | 3053 | serpin family D member 1 | D22S673\|HC2\|HCF2\|HCII\|HLS2\|LS2\|THPH10 | 22 | Antimicrobials |
| DEFB129 | 140881 | defensin beta 129 | C20orf87\|DEFB-29\|DEFB29\|bA530N10.3\|hBD-29 | 20 | Antimicrobials |
| DEFB127 | 140850 | defensin beta 127 | C20orf73\|DEF-27\|DEFB-27\|DEFB27\|bA530N10.2\|hBD-27 | 20 | Antimicrobials |
| S100P | 6286 | S100 calcium binding protein P | MIG9 | 4 | Antimicrobials |
| S100A7 | 6278 | S100 calcium binding protein A7 | PSOR1\|S100A7c | 1 | Antimicrobials |
| DEFB104A | 140596 | defensin beta 104A | BD-4\|DEFB-4\|DEFB104\|DEFB4\|hBD-4 | 8 | Antimicrobials |
| DEFB126 | 81623 | defensin beta 126 | C20orf8\|DEFB-26\|DEFB26\|HBD26\|bA530N10.1\|hBD-26 | 20 | Antimicrobials |
| DEFB106B | 503841 | defensin beta 106B | BD-6\|DEFB-6 | 8 | Antimicrobials |
| DEFB104B | 503618 | defensin beta 104B | BD-4\|DEFB-4\|hBD-4 | 8 | Antimicrobials |
| DEFB107B | 503614 | defensin beta 107B | HsT21816 | 8 | Antimicrobials |
| PGLYRP3 | 114771 | peptidoglycan recognition protein 3 | PGLYRPIalpha\|PGRP-Ialpha\|PGRPIA | 1 | Antimicrobials |
| PGLYRP2 | 114770 | peptidoglycan recognition protein 2 | HMFT0141\|PGLYRPL\|PGRP-L\|PGRPL\|TAGL-like\|tagL\|tagL-alpha\|tagl-beta | 19 | Antimicrobials |
| S100A10 | 6281 | S100 calcium binding protein A10 | 42C\|ANX2L\|ANX2LG\|CAL1L\|CLP11\|Ca[1]\|GP11\|P11\|p10 | 1 | Antimicrobials |
| S100A2 | 6273 | S100 calcium binding protein A2 | CAN19\|S100L | 1 | Antimicrobials |
| DEFB125 | 245938 | defensin beta 125 | DEFB-25 | 20 | Antimicrobials |
| DEFB123 | 245936 | defensin beta 123 | DEFB-23\|DEFB23\|ESC42-RELD | 20 | Antimicrobials |
| DEFB105B | 504180 | defensin beta 105B | BD-5\|DEFB-5 | 8 | Antimicrobials |
| DEFB132 | 400830 | defensin beta 132 | BD-32\|DEFB-32\|DEFB32\|HEL-75\|KFLL827\|UNQ827 | 20 | Antimicrobials |
| BPIFB3 | 359710 | BPI fold containing family B member 3 | C20orf185\|LPLUNC3\|RYA3 | 20 | Antimicrobials |
| LCN12 | 286256 | lipocalin 12 | - | 9 | Antimicrobials |
| PGLYRP4 | 57115 | peptidoglycan recognition protein 4 | PGLYRPIbeta\|PGRP-Ibeta\|PGRPIB\|SBBI67 | 1 | Antimicrobials |
| S100A11 | 6282 | S100 calcium binding protein A11 | HEL-S-43\|MLN70\|S100C | 1 | Antimicrobials |
| S100A5 | 6276 | S100 calcium binding protein A5 | S100D | 1 | Antimicrobials |
| S100A3 | 6274 | S100 calcium binding protein A3 | S100E | 1 | Antimicrobials |
| S100A1 | 6271 | S100 calcium binding protein A1 | S100\|S100-alpha\|S100A | 1 | Antimicrobials |
| DEFB128 | 245939 | defensin beta 128 | DEFB-28\|DEFB28\|hBD-28 | 20 | Antimicrobials |
| DEFB108B | 245911 | defensin beta 108B | DEFB-8\|hBD-8 | 11 | Antimicrobials |
| HTN1 | 3346 | histatin 1 | HIS1 | 4 | Antimicrobials |
| LMBR1L | 55716 | limb development membrane protein 1 like | LIMR | 12 | Antimicrobials |
| S100A7A | 338324 | S100 calcium binding protein A7A | NICE-2\|NICE2\|S100A15\|S100A7L1\|S100A7f | 1 | Antimicrobials |
| DEFB118 | 117285 | defensin beta 118 | C20orf63\|DEFB-18\|ESC42\|ESP13.6 | 20 | Antimicrobials |
| COLEC12 | 81035 | collectin subfamily member 12 | CLP1\|NSR2\|SCARA4\|SRCL | 18 | Antimicrobials |
| TMSB4Y | 9087 | thymosin beta 4 Y-linked | TB4Y | Y | Antimicrobials |
| DEFB131A | 644414 | defensin beta 131A | DEFB-31\|DEFB131 | 4 | Antimicrobials |
| DEFB134 | 613211 | defensin beta 134 | - | 8 | Antimicrobials |
| DEFB130A | 245940 | defensin beta 130A | DEFB-30\|DEFB130\|DEFB130L\|DEFB30 | 8 | Antimicrobials |
| DEFB124 | 245937 | defensin beta 124 | DEFB-24 | 20 | Antimicrobials |
| DEFB121 | 245934 | defensin beta 121 | DEFB21\|ESC42RELC | 20 | Antimicrobials |
| DEFB116 | 245930 | defensin beta 116 | DEFB-16 | 20 | Antimicrobials |
| DEFB115 | 245929 | defensin beta 115 | DEFB-15 | 20 | Antimicrobials |
| DEFB114 | 245928 | defensin beta 114 | DEFB-14\|DEFB14 | 6 | Antimicrobials |
| DEFB113 | 245927 | defensin beta 113 | DEFB-13 | 6 | Antimicrobials |
| DEFB112 | 245915 | defensin beta 112 | DEFB-12 | 6 | Antimicrobials |
| DEFB110 | 245913 | defensin beta 110 | DEFB-10\|DEFB-11\|DEFB111 | 6 | Antimicrobials |
| TMSB15B | 286527 | thymosin beta 15B | TMSB15A\|TMSL8\|TMSNB\|Tbeta15b | X | Antimicrobials |
| DEFB133 | 403339 | defensin beta 133 | - | 6 | Antimicrobials |
| S100Z | 170591 | S100 calcium binding protein Z | Gm625\|S100-zeta | 5 | Antimicrobials |
| MAVS | 57506 | mitochondrial antiviral signaling protein | CARDIF\|IPS-1\|IPS1\|VISA | 20 | Antimicrobials |
| TMSB4XP8 | 7117 | TMSB4X pseudogene 8 | TMSL3 | 4 | Antimicrobials |
| S100A14 | 57402 | S100 calcium binding protein A14 | BCMP84\|S100A15 | 1 | Antimicrobials |
| LCN10 | 414332 | lipocalin 10 | - | 9 | Antimicrobials |
| S100A16 | 140576 | S100 calcium binding protein A16 | AAG13\|DT1P1A7\|S100F | 1 | Antimicrobials |
| DEFB136 | 613210 | defensin beta 136 | DEFB137 | 8 | Antimicrobials |
| DEFB135 | 613209 | defensin beta 135 | DEFB136 | 8 | Antimicrobials |
| DEFB117 | 245931 | defensin beta 117 (pseudogene) | DEFB-17 | 20 | Antimicrobials |
| ZC3HAV1L | 92092 | zinc finger CCCH-type containing, antiviral 1 like | C7orf39 | 7 | Antimicrobials |
| S100A7L2 | 645922 | S100 calcium binding protein A7 like 2 | S100a7b | 1 | Antimicrobials |
| MBL3P | 50639 | mannose-binding lectin family member 3, pseudogene | COLEC2\|MBL | 10 | Antimicrobials |
| DEFB4B | 100289462 | defensin beta 4B | DEFB4P | 8 | Antimicrobials |
| BPIFB4 | 149954 | BPI fold containing family B member 4 | C20orf186\|LPLUNC4\|RY2G5\|dJ726C3.5 | 20 | Antimicrobials |
| IFNAR1 | 3454 | interferon alpha and beta receptor subunit 1 | AVP\|IFN-alpha-REC\|IFNAR\|IFNBR\|IFRC | 21 | Antimicrobials |
| AZU1 | 566 | azurocidin 1 | AZAMP\|AZU\|CAP37\|HBP\|HUMAZUR\|NAZC\|hHBP | 19 | Antimicrobials |
| DEFB131B | 100129216 | defensin beta 131B | - | 11 | Antimicrobials |
| DEFA1A3 | 613253 | defensin alpha 1 and alpha 3, variable copy number locus | DEFA1\|DEFA3\|DEFT1P | 8 | Antimicrobials |
| LCN1P1 | 286310 | lipocalin 1 pseudogene 1 | LCN1L1\|bA430N14.2 | 9 | Antimicrobials |
| S100G | 795 | S100 calcium binding protein G | CABP\|CABP1\|CABP9K\|CALB3 | X | Antimicrobials |
| DEFA7P | 724067 | defensin alpha 7, pseudogene | DEFA7 | 8 | Antimicrobials |
| DEFB130B | 100133267 | defensin beta 130B | - | 8 | Antimicrobials |
| DEFB108F | 100133128 | defensin beta 108F (pseudogene) | DEFB108P5 | 4 | Antimicrobials |
| DEFB131C | 100128174 | defensin beta 131C (pseudogene) | - | 8 | Antimicrobials |
| TCHHL1 | 126637 | trichohyalin like 1 | S100A17\|THHL1 | 1 | Antimicrobials |
| TINAGL1 | 64129 | tubulointerstitial nephritis antigen like 1 | ARG1\|LCN7\|LIECG3\|TINAGRP | 1 | Antimicrobials |
| IFNGR1 | 3459 | interferon gamma receptor 1 | CD119\|IFNGR\|IMD27A\|IMD27B | 6 | Antimicrobials |
| SLC22A17 | 51310 | solute carrier family 22 member 17 | 24p3R\|BOCT\|BOIT\|NGALR\|NGALR2\|NGALR3\|hBOIT | 14 | Antimicrobials |
| WFIKKN1 | 117166 | WAP, follistatin/kazal, immunoglobulin, kunitz and netrin domain containing 1 | C16orf12\|RJD2\|WFDC20A\|WFIKKN | 16 | Antimicrobials |
| WFDC2 | 10406 | WAP four-disulfide core domain 2 | EDDM4\|HE4\|WAP5\|dJ461P17.6 | 20 | Antimicrobials |
| IL6 | 3569 | interleukin 6 | BSF-2\|BSF2\|CDF\|HGF\|HSF\|IFN-beta-2\|IFNB2\|IL-6 | 7 | Antimicrobials |
| UMODL1 | 89766 | uromodulin like 1 | - | 21 | Antimicrobials |
| TGFB1 | 7040 | transforming growth factor beta 1 | CED\|DPD1\|IBDIMDE\|LAP\|TGF-beta1\|TGFB\|TGFbeta | 19 | Antimicrobials |
| PF4V1 | 5197 | platelet factor 4 variant 1 | CXCL4L1\|CXCL4V1\|PF4-ALT\|PF4A\|SCYB4V1 | 4 | Antimicrobials |
| MMP9 | 4318 | matrix metallopeptidase 9 | CLG4B\|GELB\|MANDP2\|MMP-9 | 20 | Antimicrobials |
| ANOS1 | 3730 | anosmin 1 | ADMLX\|HH1\|HHA\|KAL\|KAL1\|KALIG-1\|KMS\|WFDC19 | X | Antimicrobials |
| TLR4 | 7099 | toll like receptor 4 | ARMD10\|CD284\|TLR-4\|TOLL | 9 | Antimicrobials |
| SPAG11B | 10407 | sperm associated antigen 11B | EDDM2B\|EP2\|EP2C\|EP2D\|HE2\|HE2C\|SPAG11\|SPAG11A | 8 | Antimicrobials |
| A2M | 2 | alpha-2-macroglobulin | A2MD\|CPAMD5\|FWP007\|S863-7 | 12 | Antimicrobials |
| NFKB1 | 4790 | nuclear factor kappa B subunit 1 | CVID12\|EBP-1\|KBF1\|NF-kB\|NF-kB1\|NF-kappa-B1\|NF-kappaB\|NF-kappabeta\|NFKB-p105\|NFKB-p50\|NFkappaB | 4 | Antimicrobials |
| APOBEC3G | 60489 | apolipoprotein B mRNA editing enzyme catalytic subunit 3G | A3G\|ARCD\|ARP-9\|ARP9\|CEM-15\|CEM15\|MDS019\|bK150C2.7\|dJ494G10.1 | 22 | Antimicrobials |
| FABP6 | 2172 | fatty acid binding protein 6 | I-15P\|I-BABP\|I-BALB\|I-BAP\|ILBP\|ILBP3\|ILLBP | 5 | Antimicrobials |
| NOD2 | 64127 | nucleotide binding oligomerization domain containing 2 | ACUG\|BLAU\|BLAUS\|CARD15\|CD\|CLR16.3\|IBD1\|NLRC2\|NOD2B\|PSORAS1\|YAOS | 16 | Antimicrobials |
| MBL2 | 4153 | mannose binding lectin 2 | COLEC1\|HSMBPC\|MBL\|MBL2D\|MBP\|MBP-C\|MBP1\|MBPD | 10 | Antimicrobials |
| SFTPA1 | 653509 | surfactant protein A1 | COLEC4\|PSAP\|PSP-A\|PSPA\|SFTP1\|SFTPA1B\|SP-A\|SP-A1\|SP-A1 beta\|SP-A1 delta\|SP-A1 epsilon\|SP-A1 gamma\|SPA\|SPA1 | 10 | Antimicrobials |
| RBP1 | 5947 | retinol binding protein 1 | CRABP-I\|CRBP\|CRBP1\|CRBPI\|RBPC | 3 | Antimicrobials |
| TLR2 | 7097 | toll like receptor 2 | CD282\|TIL4 | 4 | Antimicrobials |
| SLC40A1 | 30061 | solute carrier family 40 member 1 | FPN1\|HFE4\|IREG1\|MST079\|MSTP079\|MTP1\|SLC11A3 | 2 | Antimicrobials |
| PLAU | 5328 | plasminogen activator, urokinase | ATF\|BDPLT5\|QPD\|UPA\|URK\|u-PA | 10 | Antimicrobials |
| IL1B | 3553 | interleukin 1 beta | IL-1\|IL1-BETA\|IL1F2\|IL1beta | 2 | Antimicrobials |
| PAEP | 5047 | progestagen associated endometrial protein | GD\|GdA\|GdF\|GdS\|PAEG\|PEP\|PP14\|ZIF-1 | 9 | Antimicrobials |
| HJV | 148738 | hemojuvelin BMP co-receptor | HFE2\|HFE2A\|JH\|RGMC | 1 | Antimicrobials |
| MUC5AC | 4586 | mucin 5AC, oligomeric mucus/gel-forming | MUC5\|TBM\|leB\|mucin | 11 | Antimicrobials |
| OBP2A | 29991 | odorant binding protein 2A | LCN13\|OBP\|OBP2C\|OBPIIa\|hOBPIIa | 9 | Antimicrobials |
| PLTP | 5360 | phospholipid transfer protein | BPIFE\|HDLCQ9 | 20 | Antimicrobials |
| MX1 | 4599 | MX dynamin like GTPase 1 | IFI-78K\|IFI78\|MX\|MxA\|lncMX1-215 | 21 | Antimicrobials |
| DDX58 | 23586 | DExD/H-box helicase 58 | RIG-I\|RIG1\|RIGI\|RLR-1\|SGMRT2 | 9 | Antimicrobials |
| IFNL1 | 282618 | interferon lambda 1 | IL-29\|IL29 | 19 | Antimicrobials |
| IRF3 | 3661 | interferon regulatory factor 3 | IIAE7 | 19 | Antimicrobials |
| SFTPA2 | 729238 | surfactant protein A2 | COLEC5\|PSAP\|PSP-A\|PSPA\|SFTP1\|SFTPA2B\|SP-2A\|SP-A\|SPA2\|SPAII | 10 | Antimicrobials |
| LPA | 4018 | lipoprotein(a) | AK38\|APOA\|LP | 6 | Antimicrobials |
| LBP | 3929 | lipopolysaccharide binding protein | BPIFD2 | 20 | Antimicrobials |
| RBP4 | 5950 | retinol binding protein 4 | MCOPCB10\|RDCCAS | 10 | Antimicrobials |
| NOX4 | 50507 | NADPH oxidase 4 | KOX\|KOX-1\|RENOX | 11 | Antimicrobials |
| LTF | 4057 | lactotransferrin | GIG12\|HEL110\|HLF2\|LF | 3 | Antimicrobials |
| IFNB1 | 3456 | interferon beta 1 | IFB\|IFF\|IFN-beta\|IFNB | 9 | Antimicrobials |
| RBP5 | 83758 | retinol binding protein 5 | CRBP-III\|CRBP3\|CRBPIII\|HRBPiso | 12 | Antimicrobials |
| FABP7 | 2173 | fatty acid binding protein 7 | B-FABP\|BLBP\|FABPB\|MRG | 6 | Antimicrobials |
| FABP5 | 2171 | fatty acid binding protein 5 | E-FABP\|EFABP\|KFABP\|PA-FABP\|PAFABP | 8 | Antimicrobials |
| FABP3 | 2170 | fatty acid binding protein 3 | FABP11\|H-FABP\|M-FABP\|MDGI\|O-FABP | 1 | Antimicrobials |
| FABP2 | 2169 | fatty acid binding protein 2 | FABPI\|I-FABP | 4 | Antimicrobials |
| FABP4 | 2167 | fatty acid binding protein 4 | A-FABP\|AFABP\|ALBP\|HEL-S-104\|aP2 | 8 | Antimicrobials |
| R3HDML | 140902 | R3H domain containing like | dJ881L22.3 | 20 | Antimicrobials |
| BPIFA3 | 128861 | BPI fold containing family A member 3 | C20orf71\|SPLUNC3 | 20 | Antimicrobials |
| BPIFB1 | 92747 | BPI fold containing family B member 1 | C20orf114\|LPLUNC1 | 20 | Antimicrobials |
| OASL | 8638 | 2'-5'-oligoadenylate synthetase like | OASL1\|OASLd\|TRIP-14\|TRIP14\|p59 OASL\|p59-OASL\|p59OASL | 12 | Antimicrobials |
| CRABP2 | 1382 | cellular retinoic acid binding protein 2 | CRABP-II\|RBP6 | 1 | Antimicrobials |
| CRABP1 | 1381 | cellular retinoic acid binding protein 1 | CRABP\|CRABP-I\|CRABPI\|RBP5 | 15 | Antimicrobials |
| RBP7 | 116362 | retinol binding protein 7 | CRABP4\|CRBP4\|CRBPIV | 1 | Antimicrobials |
| DUOX1 | 53905 | dual oxidase 1 | LNOX1\|NOXEF1\|THOX1 | 15 | Antimicrobials |
| OBP2B | 29989 | odorant binding protein 2B | LCN14\|OBPIIb | 9 | Antimicrobials |
| RBP2 | 5948 | retinol binding protein 2 | CRABP-II\|CRBP2\|CRBPII\|RBPC2 | 3 | Antimicrobials |
| LCN15 | 389812 | lipocalin 15 | PRO6093\|UNQ2541 | 9 | Antimicrobials |
| CETP | 1071 | cholesteryl ester transfer protein | BPIFF\|HDLCQ10 | 16 | Antimicrobials |
| FABP12 | 646486 | fatty acid binding protein 12 | - | 8 | Antimicrobials |
| FABP9 | 646480 | fatty acid binding protein 9 | PERF\|PERF15\|T-FABP\|TLBP | 8 | Antimicrobials |
| BPIFA1 | 51297 | BPI fold containing family A member 1 | LUNX\|NASG\|PLUNC\|SPLUNC1\|SPURT\|bA49G10.5 | 20 | Antimicrobials |
| LCNL1 | 401562 | lipocalin like 1 | - | 9 | Antimicrobials |
| C8G | 733 | complement C8 gamma chain | C8C | 9 | Antimicrobials |
| SPAG11A | 653423 | sperm associated antigen 11A | EDDM2A\|HE2 | 8 | Antimicrobials |
| PI15 | 51050 | peptidase inhibitor 15 | CRISP8\|P24TI\|P25TI | 8 | Antimicrobials |
| NOX1 | 27035 | NADPH oxidase 1 | GP91-2\|MOX1\|NOH-1\|NOH1 | X | Antimicrobials |
| PMP2 | 5375 | peripheral myelin protein 2 | CMT1G\|FABP8\|M-FABP\|MP2\|P2 | 8 | Antimicrobials |
| APOD | 347 | apolipoprotein D | - | 3 | Antimicrobials |
| ORM2 | 5005 | orosomucoid 2 | AGP-B\|AGP-B'\|AGP2 | 9 | Antimicrobials |
| ORM1 | 5004 | orosomucoid 1 | AGP-A\|AGP1\|HEL-S-153w\|ORM | 9 | Antimicrobials |
| TNF | 7124 | tumor necrosis factor | DIF\|TNF-alpha\|TNFA\|TNFSF2\|TNLG1F | 6 | Antimicrobials |
| CTSG | 1511 | cathepsin G | CATG\|CG | 14 | Antimicrobials |
| PRTN3 | 5657 | proteinase 3 | ACPA\|AGP7\|C-ANCA\|CANCA\|MBN\|MBT\|NP-4\|NP4\|P29\|PR-3\|PR3 | 19 | Antimicrobials |
| MAPK1 | 5594 | mitogen-activated protein kinase 1 | ERK\|ERK-2\|ERK2\|ERT1\|MAPK2\|P42MAPK\|PRKM1\|PRKM2\|p38\|p40\|p41\|p41mapk\|p42-MAPK | 22 | Antimicrobials |
| PML | 5371 | PML nuclear body scaffold | MYL\|PP8675\|RNF71\|TRIM19 | 15 | Antimicrobials |
| AEN | 64782 | apoptosis enhancing nuclease | ISG20L1\|pp12744 | 15 | Antimicrobials |
| CYBB | 1536 | cytochrome b-245 beta chain | AMCBX2\|CGD\|GP91-1\|GP91-PHOX\|GP91PHOX\|IMD34\|NOX2\|p91-PHOX | X | Antimicrobials |
| BPIFA2 | 140683 | BPI fold containing family A member 2 | C20orf70\|PSP\|SPLUNC2\|bA49G10.1 | 20 | Antimicrobials |
| ISG20 | 3669 | interferon stimulated exonuclease gene 20 | CD25\|HEM45 | 15 | Antimicrobials |
| BCL3 | 602 | BCL3 transcription coactivator | BCL4\|D19S37 | 19 | Antimicrobials |
| ISG20L2 | 81875 | interferon stimulated exonuclease gene 20 like 2 | HSD38 | 1 | Antimicrobials |
| NOX5 | 79400 | NADPH oxidase 5 | - | 15 | Antimicrobials |
| NOX3 | 50508 | NADPH oxidase 3 | GP91-3\|MOX-2 | 6 | Antimicrobials |
| DUOX2 | 50506 | dual oxidase 2 | LNOX2\|NOXEF2\|P138-TOX\|TDH6\|THOX2 | 15 | Antimicrobials |
| TLR3 | 7098 | toll like receptor 3 | CD283\|IIAE2 | 4 | Antimicrobials |
| TFRC | 7037 | transferrin receptor | CD71\|IMD46\|T9\|TFR\|TFR1\|TR\|TRFR\|p90 | 3 | Antimicrobials |
| IFIH1 | 64135 | interferon induced with helicase C domain 1 | AGS7\|Hlcd\|IDDM19\|MDA-5\|MDA5\|RLR-2\|SGMRT1 | 2 | Antimicrobials |
| LRP1 | 4035 | LDL receptor related protein 1 | A2MR\|APOER\|APR\|CD91\|IGFBP-3R\|IGFBP3R\|IGFBP3R1\|KPA\|LRP\|LRP1A\|TGFBR5 | 12 | Antimicrobials |
| TRIM5 | 85363 | tripartite motif containing 5 | RNF88\|TRIM5alpha | 11 | Antimicrobials |
| IDO1 | 3620 | indoleamine 2,3-dioxygenase 1 | IDO\|IDO-1\|INDO | 8 | Antimicrobials |
| GDF15 | 9518 | growth differentiation factor 15 | GDF-15\|MIC-1\|MIC1\|NAG-1\|PDF\|PLAB\|PTGFB | 19 | Antimicrobials |
| NEDD4 | 4734 | NEDD4 E3 ubiquitin protein ligase | NEDD4-1\|RPF1 | 15 | Antimicrobials |
| ADIPOQ | 9370 | adiponectin, C1Q and collagen domain containing | ACDC\|ACRP30\|ADIPQTL1\|ADPN\|APM-1\|APM1\|GBP28 | 3 | Antimicrobials |
| STAT3 | 6774 | signal transducer and activator of transcription 3 | ADMIO\|ADMIO1\|APRF\|HIES | 17 | Antimicrobials |
| STAT1 | 6772 | signal transducer and activator of transcription 1 | CANDF7\|IMD31A\|IMD31B\|IMD31C\|ISGF-3\|STAT91 | 2 | Antimicrobials |
| IFNL2 | 282616 | interferon lambda 2 | IL-28A\|IL28A | 19 | Antimicrobials |
| SOCS3 | 9021 | suppressor of cytokine signaling 3 | ATOD4\|CIS3\|Cish3\|SOCS-3\|SSI-3\|SSI3 | 17 | Antimicrobials |
| SEMG1 | 6406 | semenogelin 1 | CT103\|SEMG\|SGI\|dJ172H20.2 | 20 | Antimicrobials |
| TNFSF10 | 8743 | TNF superfamily member 10 | APO2L\|Apo-2L\|CD253\|TL2\|TNLG6A\|TRAIL | 3 | Antimicrobials |
| CCL20 | 6364 | C-C motif chemokine ligand 20 | CKb4\|Exodus\|LARC\|MIP-3-alpha\|MIP-3a\|MIP3A\|SCYA20\|ST38 | 2 | Antimicrobials |
| SOCS1 | 8651 | suppressor of cytokine signaling 1 | CIS1\|CISH1\|JAB\|SOCS-1\|SSI-1\|SSI1\|TIP-3\|TIP3 | 16 | Antimicrobials |
| RNASEL | 6041 | ribonuclease L | PRCA1\|RNS4 | 1 | Antimicrobials |
| IRF1 | 3659 | interferon regulatory factor 1 | IRF-1\|MAR | 5 | Antimicrobials |
| IL15 | 3600 | interleukin 15 | IL-15 | 4 | Antimicrobials |
| APOBEC3F | 200316 | apolipoprotein B mRNA editing enzyme catalytic subunit 3F | A3F\|ARP8\|BK150C2.4.MRNA\|KA6 | 22 | Antimicrobials |
| PLAAT4 | 5920 | phospholipase A and acyltransferase 4 | HRASLS4\|HRSL4\|PLA1/2-3\|PLAAT-4\|RARRES3\|RIG1\|TIG3 | 11 | Antimicrobials |
| CHIT1 | 1118 | chitinase 1 | CHI3\|CHIT\|CHITD | 1 | Antimicrobials |
| CD40 | 958 | CD40 molecule | Bp50\|CDW40\|TNFRSF5\|p50 | 20 | Antimicrobials |
| TLR7 | 51284 | toll like receptor 7 | TLR7-like | X | Antimicrobials |
| PPIA | 5478 | peptidylprolyl isomerase A | CYPA\|CYPH\|HEL-S-69p | 7 | Antimicrobials |
| ZYX | 7791 | zyxin | ESP-2\|HED-2 | 7 | Antimicrobials |
| NLRX1 | 79671 | NLR family member X1 | CLR11.3\|DLNB26\|NOD26\|NOD5\|NOD9 | 11 | Antimicrobials |
| PGC | 5225 | progastricsin | PEPC\|PGII | 6 | Antimicrobials |
| VEGFA | 7422 | vascular endothelial growth factor A | MVCD1\|VEGF\|VPF | 6 | Antimicrobials |
| IKBKE | 9641 | inhibitor of nuclear factor kappa B kinase subunit epsilon | IKK-E\|IKK-i\|IKKE\|IKKI | 1 | Antimicrobials |
| ISG15 | 9636 | ISG15 ubiquitin like modifier | G1P2\|IFI15\|IMD38\|IP17\|UCRP\|hUCRP | 1 | Antimicrobials |
| DHX58 | 79132 | DExH-box helicase 58 | D11LGP2\|D11lgp2e\|LGP2\|RLR-3 | 17 | Antimicrobials |
| TNFAIP3 | 7128 | TNF alpha induced protein 3 | A20\|AISBL\|OTUD7C\|TNFA1P2 | 6 | Antimicrobials |
| TFR2 | 7036 | transferrin receptor 2 | HFE3\|TFRC2 | 7 | Antimicrobials |
| FCN2 | 2220 | ficolin 2 | EBP-37\|FCNL\|P35\|ficolin-2 | 9 | Antimicrobials |
| MUC4 | 4585 | mucin 4, cell surface associated | ASGP\|HSA276359\|MUC-4 | 3 | Antimicrobials |
| F2R | 2149 | coagulation factor II thrombin receptor | CF2R\|HTR\|PAR-1\|PAR1\|TR | 5 | Antimicrobials |
| ELN | 2006 | elastin | ADCL1\|SVAS\|WBS\|WS | 7 | Antimicrobials |
| IL27 | 246778 | interleukin 27 | IL-27\|IL-27A\|IL27A\|IL27p28\|IL30\|p28 | 16 | Antimicrobials |
| MAPT | 4137 | microtubule associated protein tau | DDPAC\|FTDP-17\|MAPTL\|MSTD\|MTBT1\|MTBT2\|PPND\|PPP1R103\|TAU | 17 | Antimicrobials |
| LYZ | 4069 | lysozyme | LYZF1\|LZM | 12 | Antimicrobials |
| CCL5 | 6352 | C-C motif chemokine ligand 5 | D17S136E\|RANTES\|SCYA5\|SIS-delta\|SISd\|TCP228\|eoCP | 17 | Antimicrobials |
| LEP | 3952 | leptin | LEPD\|OB\|OBS | 7 | Antimicrobials |
| CYLD | 1540 | CYLD lysine 63 deubiquitinase | BRSS\|CDMT\|CYLD1\|CYLDI\|EAC\|MFT\|MFT1\|SBS\|TEM\|USPL2 | 16 | Antimicrobials |
| KLKB1 | 3818 | kallikrein B1 | KLK3\|PKK\|PKKD\|PPK | 4 | Antimicrobials |
| CST4 | 1472 | cystatin S | - | 20 | Antimicrobials |
| CSRP1 | 1465 | cysteine and glycine rich protein 1 | CRP\|CRP1\|CSRP\|CYRP\|D1S181E\|HEL-141\|HEL-S-286 | 1 | Antimicrobials |
| MAPK14 | 1432 | mitogen-activated protein kinase 14 | CSBP\|CSBP1\|CSBP2\|CSPB1\|EXIP\|Mxi2\|PRKM14\|PRKM15\|RK\|SAPK2A\|p38\|p38ALPHA | 6 | Antimicrobials |
| JUN | 3725 | Jun proto-oncogene, AP-1 transcription factor subunit | AP-1\|AP1\|c-Jun\|cJUN\|p39 | 1 | Antimicrobials |
| ITGAV | 3685 | integrin subunit alpha V | CD51\|MSK8\|VNRA\|VTNR | 2 | Antimicrobials |
| IRF5 | 3663 | interferon regulatory factor 5 | SLEB10 | 7 | Antimicrobials |
| CCR6 | 1235 | C-C motif chemokine receptor 6 | BN-1\|C-C CKR-6\|CC-CKR-6\|CCR-6\|CD196\|CKR-L3\|CKRL3\|CMKBR6\|DCR2\|DRY6\|GPR29\|GPRCY4\|STRL22 | 6 | Antimicrobials |
| IL12B | 3593 | interleukin 12B | CLMF\|CLMF2\|IL-12B\|IMD28\|IMD29\|NKSF\|NKSF2 | 5 | Antimicrobials |
| TLR8 | 51311 | toll like receptor 8 | CD288 | X | Antimicrobials |
| GNLY | 10578 | granulysin | D2S69E\|LAG-2\|LAG2\|NKG5\|TLA519 | 2 | Antimicrobials |
| CD81 | 975 | CD81 molecule | CVID6\|S5.7\|TAPA1\|TSPAN28 | 11 | Antimicrobials |
| EIF2AK2 | 5610 | eukaryotic translation initiation factor 2 alpha kinase 2 | EIF2AK1\|LEUDEN\|PKR\|PPP1R83\|PRKR | 2 | Antimicrobials |
| APOM | 55937 | apolipoprotein M | G3a\|HSPC336\|NG20\|apo-M | 6 | Antimicrobials |
| CACYBP | 27101 | calcyclin binding protein | GIG5\|PNAS-107\|S100A6BP\|SIP | 1 | Antimicrobials |
| NOD1 | 10392 | nucleotide binding oligomerization domain containing 1 | CARD4\|CLR7.1\|NLRC1 | 7 | Antimicrobials |
| MAPK8 | 5599 | mitogen-activated protein kinase 8 | JNK\|JNK-46\|JNK1\|JNK1A2\|JNK21B1/2\|PRKM8\|SAPK1\|SAPK1c | 10 | Antimicrobials |
| MAPK3 | 5595 | mitogen-activated protein kinase 3 | ERK-1\|ERK1\|ERT2\|HS44KDAP\|HUMKER1A\|P44ERK1\|P44MAPK\|PRKM3\|p44-ERK1\|p44-MAPK | 16 | Antimicrobials |
| BST2 | 684 | bone marrow stromal cell antigen 2 | CD317\|TETHERIN | 19 | Antimicrobials |
| BPHL | 670 | biphenyl hydrolase like | BPH-RP\|MCNAA\|VACVASE | 6 | Antimicrobials |
| PLA2G2A | 5320 | phospholipase A2 group IIA | MOM1\|PLA2\|PLA2B\|PLA2L\|PLA2S\|PLAS1\|sPLA2 | 1 | Antimicrobials |
| GRN | 2896 | granulin precursor | CLN11\|GEP\|GP88\|PCDGF\|PEPI\|PGRN | 17 | Antimicrobials |
| NEWENTRY | 192343 | - | - | - | Antimicrobials |
| PDGFRA | 5156 | platelet derived growth factor receptor alpha | CD140A\|PDGFR-2\|PDGFR2 | 4 | Antimicrobials |
| GNAI1 | 2770 | G protein subunit alpha i1 | Gi | 7 | Antimicrobials |
| WNT5A | 7474 | Wnt family member 5A | hWNT5A | 3 | Antimicrobials |
| FURIN | 5045 | furin, paired basic amino acid cleaving enzyme | FUR\|PACE\|PCSK3\|SPC1 | 15 | Antimicrobials |
| ADAR | 103 | adenosine deaminase RNA specific | ADAR1\|AGS6\|DRADA\|DSH\|DSRAD\|G1P1\|IFI-4\|IFI4\|K88DSRBP\|P136 | 1 | Antimicrobials |
| TYK2 | 7297 | tyrosine kinase 2 | IMD35\|JTK1 | 19 | Antimicrobials |
| NOS2 | 4843 | nitric oxide synthase 2 | HEP-NOS\|INOS\|NOS\|NOS2A | 17 | Antimicrobials |
| TRAF3 | 7187 | TNF receptor associated factor 3 | CAP-1\|CAP1\|CD40bp\|CRAF1\|IIAE5\|LAP1\|RNF118 | 14 | Antimicrobials |
| TPT1 | 7178 | tumor protein, translationally-controlled 1 | HRF\|TCTP\|p02\|p23 | 13 | Antimicrobials |
| TPM2 | 7169 | tropomyosin 2 | AMCD1\|DA1\|DA2B\|DA2B4\|HEL-S-273\|NEM4\|TMSB | 9 | Antimicrobials |
| NEO1 | 4756 | neogenin 1 | IGDCC2\|NGN\|NTN1R2 | 15 | Antimicrobials |
| AHNAK | 79026 | AHNAK nucleoprotein | AHNAKRS\|PM227 | 11 | Antimicrobials |
| TLR1 | 7096 | toll like receptor 1 | CD281\|TIL\|TIL. LPRS5\|rsc786 | 4 | Antimicrobials |
| TK2 | 7084 | thymidine kinase 2 | MTDPS2\|MTTK\|PEOB3\|SCA31 | 16 | Antimicrobials |
| PRDX2 | 7001 | peroxiredoxin 2 | HEL-S-2a\|NKEF-B\|NKEFB\|PRP\|PRX2\|PRXII\|PTX1\|TDPX1\|TPX1\|TSA | 19 | Antimicrobials |
| MX2 | 4600 | MX dynamin like GTPase 2 | MXB | 21 | Antimicrobials |
| FGF2 | 2247 | fibroblast growth factor 2 | BFGF\|FGF-2\|FGFB\|HBGF-2 | 4 | Antimicrobials |
| FGA | 2243 | fibrinogen alpha chain | Fib2 | 4 | Antimicrobials |
| TCF7L2 | 6934 | transcription factor 7 like 2 | TCF-4\|TCF4 | 10 | Antimicrobials |
| F2RL1 | 2150 | F2R like trypsin receptor 1 | GPR11\|PAR2 | 5 | Antimicrobials |
| TKFC | 26007 | triokinase and FMN cyclase | DAK\|NET45\|TKFCD | 11 | Antimicrobials |
| MSR1 | 4481 | macrophage scavenger receptor 1 | CD204\|SCARA1\|SR-A\|SR-AI\|SR-AII\|SR-AIII\|SRA\|phSR1\|phSR2 | 8 | Antimicrobials |
| NFKBIZ | 64332 | NFKB inhibitor zeta | IKBZ\|INAP\|MAIL | 3 | Antimicrobials |
| LMBR1 | 64327 | limb development membrane protein 1 | ACHP\|C7orf2\|DIF14\|LSS\|PPD2\|THYP\|TPT\|ZRS | 7 | Antimicrobials |
| EPPIN | 57119 | epididymal peptidase inhibitor | CT71\|CT72\|SPINLW1\|WAP7\|WFDC7\|dJ461P17.2 | 20 | Antimicrobials |
| SRC | 6714 | SRC proto-oncogene, non-receptor tyrosine kinase | ASV\|SRC1\|THC6\|c-SRC\|p60-Src | 20 | Antimicrobials |
| MPO | 4353 | myeloperoxidase | - | 17 | Antimicrobials |
| ELAVL1 | 1994 | ELAV like RNA binding protein 1 | ELAV1\|HUR\|Hua\|MelG | 19 | Antimicrobials |
| ROBO3 | 64221 | roundabout guidance receptor 3 | HGPPS\|HGPPS1\|HGPS\|RBIG1\|RIG1 | 11 | Antimicrobials |
| SP1 | 6667 | Sp1 transcription factor | - | 12 | Antimicrobials |
| SOD1 | 6647 | superoxide dismutase 1 | ALS\|ALS1\|HEL-S-44\|IPOA\|SOD\|STAHP\|hSod1\|homodimer | 21 | Antimicrobials |
| PDF | 64146 | peptide deformylase, mitochondrial | - | 16 | Antimicrobials |
| DLL4 | 54567 | delta like canonical Notch ligand 4 | AOS6\|delta4\|hdelta2 | 15 | Antimicrobials |
| ECD | 11319 | ecdysoneless cell cycle regulator | GCR2\|HSGT1\|SGT1 | 10 | Antimicrobials |
| SLC11A1 | 6556 | solute carrier family 11 member 1 | LSH\|NRAMP\|NRAMP1 | 2 | Antimicrobials |
| DMBT1 | 1755 | deleted in malignant brain tumors 1 | GP340\|SAG\|SALSA\|muclin | 10 | Antimicrobials |
| STING1 | 340061 | stimulator of interferon response cGAMP interactor 1 | ERIS\|MITA\|MPYS\|NET23\|SAVI\|STING\|STING-beta\|TMEM173\|hMITA\|hSTING | 5 | Antimicrobials |
| SKIV2L | 6499 | Ski2 like RNA helicase | 170A\|DDX13\|HLP\|SKI2\|SKI2W\|SKIV2\|SKIV2L1\|THES2 | 6 | Antimicrobials |
| SEMG2 | 6407 | semenogelin 2 | SGII | 20 | Antimicrobials |
| DES | 1674 | desmin | CDCD3\|CSM1\|CSM2\|LGMD1D\|LGMD1E\|LGMD2R | 2 | Antimicrobials |
| DCK | 1633 | deoxycytidine kinase | - | 4 | Antimicrobials |
| DAXX | 1616 | death domain associated protein | BING2\|DAP6\|EAP1\|SMIM40 | 6 | Antimicrobials |
| TNFRSF10A | 8797 | TNF receptor superfamily member 10a | APO2\|CD261\|DR4\|TRAILR-1\|TRAILR1 | 8 | Antimicrobials |
| TNFRSF10B | 8795 | TNF receptor superfamily member 10b | CD262\|DR5\|KILLER\|KILLER/DR5\|TRAIL-R2\|TRAILR2\|TRICK2\|TRICK2A\|TRICK2B\|TRICKB\|ZTNFR9 | 8 | Antimicrobials |
| EED | 8726 | embryonic ectoderm development | COGIS\|HEED\|WAIT1 | 11 | Antimicrobials |
| CCL4 | 6351 | C-C motif chemokine ligand 4 | ACT2\|AT744.1\|G-26\|HC21\|LAG-1\|LAG1\|MIP-1-beta\|MIP1B\|MIP1B1\|SCYA2\|SCYA4 | 17 | Antimicrobials |
| LIMS1 | 3987 | LIM zinc finger domain containing 1 | PINCH\|PINCH-1\|PINCH1 | 2 | Antimicrobials |
| LALBA | 3906 | lactalbumin alpha | LYZG | 12 | Antimicrobials |
| APOBEC3H | 164668 | apolipoprotein B mRNA editing enzyme catalytic subunit 3H | A3H\|ARP-10\|ARP10 | 22 | Antimicrobials |
| TMPRSS6 | 164656 | transmembrane serine protease 6 | IRIDA\|MT2 | 22 | Antimicrobials |
| SPINK5 | 11005 | serine peptidase inhibitor Kazal type 5 | LEKTI\|LETKI\|NETS\|NS\|VAKTI | 5 | Antimicrobials |
| MARCO | 8685 | macrophage receptor with collagenous structure | SCARA2\|SR-A6 | 2 | Antimicrobials |
| BECN1 | 8678 | beclin 1 | ATG6\|VPS30\|beclin1 | 17 | Antimicrobials |
| TNFSF11 | 8600 | TNF superfamily member 11 | CD254\|ODF\|OPGL\|OPTB2\|RANKL\|TNLG6B\|TRANCE\|hRANKL2\|sOdf | 13 | Antimicrobials |
| KNG1 | 3827 | kininogen 1 | BDK\|BK\|HMWK\|KNG | 3 | Antimicrobials |
| CSK | 1445 | C-terminal Src kinase | - | 15 | Antimicrobials |
| KLRK1 | 22914 | killer cell lectin like receptor K1 | CD314\|D12S2489E\|KLR\|NKG2-D\|NKG2D | 12 | Antimicrobials |
| KCNH2 | 3757 | potassium voltage-gated channel subfamily H member 2 | ERG-1\|ERG1\|H-ERG\|HERG\|HERG1\|Kv11.1\|LQT2\|SQT1 | 7 | Antimicrobials |
| JUND | 3727 | JunD proto-oncogene, AP-1 transcription factor subunit | AP-1 | 19 | Antimicrobials |
| JAK1 | 3716 | Janus kinase 1 | JAK1A\|JAK1B\|JTK3 | 1 | Antimicrobials |
| CLDN4 | 1364 | claudin 4 | CPE-R\|CPER\|CPETR\|CPETR1\|WBSCR8\|hCPE-R | 7 | Antimicrobials |
| CCL28 | 56477 | C-C motif chemokine ligand 28 | CCK1\|MEC\|SCYA28 | 5 | Antimicrobials |
| RNASE3 | 6037 | ribonuclease A family member 3 | ECP\|RAF1\|RNS3 | 14 | Antimicrobials |
| RN7SL1 | 6029 | RNA component of signal recognition particle 7SL1 | 7L1a\|7SL\|RN7SL\|RNSRP1 | 14 | Antimicrobials |
| IRF7 | 3665 | interferon regulatory factor 7 | IMD39\|IRF-7\|IRF-7H\|IRF7A\|IRF7B\|IRF7C\|IRF7H | 11 | Antimicrobials |
| IREB2 | 3658 | iron responsive element binding protein 2 | ACO3\|IRE-BP 2\|IRE-BP2\|IRP2\|IRP2AD\|NDCAMA | 15 | Antimicrobials |
| ILK | 3611 | integrin linked kinase | HEL-S-28\|ILK-1\|ILK-2\|P59\|p59ILK | 11 | Antimicrobials |
| IL18 | 3606 | interleukin 18 | IGIF\|IL-18\|IL-1g\|IL1F4 | 11 | Antimicrobials |
| IL17A | 3605 | interleukin 17A | CTLA-8\|CTLA8\|IL-17\|IL-17A\|IL17 | 6 | Antimicrobials |
| LTB4R | 1241 | leukotriene B4 receptor | BLT1\|BLTR\|CMKRL1\|GPR16\|LTB4R1\|LTBR1\|P2RY7\|P2Y7 | 14 | Antimicrobials |
| APOBEC3A | 200315 | apolipoprotein B mRNA editing enzyme catalytic subunit 3A | A3A\|ARP3\|PHRBN\|bK150C2.1 | 22 | Antimicrobials |
| MASP2 | 10747 | mannan binding lectin serine peptidase 2 | MAP19\|MASP-2\|MASP1P1\|sMAP | 1 | Antimicrobials |
| TRIM27 | 5987 | tripartite motif containing 27 | RFP\|RNF76 | 6 | Antimicrobials |
| RELA | 5970 | RELA proto-oncogene, NF-kB subunit | CMCU\|NFKB3\|p65 | 11 | Antimicrobials |
| IL7R | 3575 | interleukin 7 receptor | CD127\|CDW127\|IL-7R-alpha\|IL7RA\|ILRA | 5 | Antimicrobials |
| IL1A | 3552 | interleukin 1 alpha | IL-1 alpha\|IL-1A\|IL1\|IL1-ALPHA\|IL1F1 | 2 | Antimicrobials |
| PTX3 | 5806 | pentraxin 3 | TNFAIP5\|TSG-14 | 3 | Antimicrobials |
| IFNAR2 | 3455 | interferon alpha and beta receptor subunit 2 | IFN-R\|IFN-alpha-REC\|IFNABR\|IFNARB\|IMD45 | 21 | Antimicrobials |
| IFN1@ | 3438 | - | IFNA | 9 | Antimicrobials |
| SYTL1 | 84958 | synaptotagmin like 1 | JFC1\|SLP1 | 1 | Antimicrobials |
| APOBEC3C | 27350 | apolipoprotein B mRNA editing enzyme catalytic subunit 3C | A3C\|APOBEC1L\|ARDC2\|ARDC4\|ARP5\|PBI\|bK150C2.3 | 22 | Antimicrobials |
| DDX17 | 10521 | DEAD-box helicase 17 | P72\|RH70 | 22 | Antimicrobials |
| PTGS2 | 5743 | prostaglandin-endoperoxide synthase 2 | COX-2\|COX2\|GRIPGHS\|PGG/HS\|PGHS-2\|PHS-2\|hCox-2 | 1 | Antimicrobials |
| HTR1A | 3350 | 5-hydroxytryptamine receptor 1A | 5-HT-1A\|5-HT1A\|5HT1a\|ADRB2RL1\|ADRBRL1\|G-21\|PFMCD | 5 | Antimicrobials |
| SEPTIN7 | 989 | septin 7 | CDC10\|CDC3\|NBLA02942\|SEPT7\|SEPT7A | 7 | Antimicrobials |
| CD40LG | 959 | CD40 ligand | CD154\|CD40L\|HIGM1\|IGM\|IMD3\|T-BAM\|TNFSF5\|TRAP\|gp39\|hCD40L | X | Antimicrobials |
| CD14 | 929 | CD14 molecule | - | 5 | Antimicrobials |
| MASP1 | 5648 | mannan binding lectin serine peptidase 1 | 3MC1\|CRARF\|CRARF1\|MAP1\|MASP\|MASP3\|MAp44\|PRSS5\|RaRF | 3 | Antimicrobials |
| PROC | 5624 | protein C, inactivator of coagulation factors Va and VIIIa | APC\|PC\|PROC1\|THPH3\|THPH4 | 2 | Antimicrobials |
| MAP2K2 | 5605 | mitogen-activated protein kinase kinase 2 | CFC4\|MAPKK2\|MEK2\|MKK2\|PRKMK2 | 19 | Antimicrobials |
| MAP2K1 | 5604 | mitogen-activated protein kinase kinase 1 | CFC3\|MAPKK1\|MEK1\|MKK1\|PRKMK1 | 15 | Antimicrobials |
| HRG | 3273 | histidine rich glycoprotein | HPRG\|HRGP\|THPH11 | 3 | Antimicrobials |
| NDRG1 | 10397 | N-myc downstream regulated 1 | CAP43\|CMT4D\|DRG-1\|DRG1\|GC4\|HMSNL\|NDR1\|NMSL\|PROXY1\|RIT42\|RTP\|TARG1\|TDD5 | 8 | Antimicrobials |
| IRF9 | 10379 | interferon regulatory factor 9 | IRF-9\|ISGF3\|ISGF3G\|p48 | 14 | Antimicrobials |
| TRIM22 | 10346 | tripartite motif containing 22 | GPSTAF50\|RNF94\|STAF50 | 11 | Antimicrobials |
| LANCL1 | 10314 | LanC like 1 | GPR69A\|p40 | 2 | Antimicrobials |
| PPP4C | 5531 | protein phosphatase 4 catalytic subunit | PP-X\|PP4\|PP4C\|PPH3\|PPP4\|PPX | 16 | Antimicrobials |
| HMOX1 | 3162 | heme oxygenase 1 | HMOX1D\|HO-1\|HSP32\|bK286B10 | 22 | Antimicrobials |
| HMGB1 | 3146 | high mobility group box 1 | HMG-1\|HMG1\|HMG3\|SBP-1 | 13 | Antimicrobials |
| RNASE7 | 84659 | ribonuclease A family member 7 | RAE1 | 14 | Antimicrobials |
| ABCC4 | 10257 | ATP binding cassette subfamily C member 4 | MOAT-B\|MOATB\|MRP4 | 13 | Antimicrobials |
| HGF | 3082 | hepatocyte growth factor | DFNB39\|F-TCF\|HGFB\|HPTA\|SF | 7 | Antimicrobials |
| HDAC1 | 3065 | histone deacetylase 1 | GON-10\|HD1\|KDAC1\|RPD3\|RPD3L1 | 1 | Antimicrobials |
| IFNLR1 | 163702 | interferon lambda receptor 1 | CRF2/12\|IFNLR\|IL-28R1\|IL28RA\|LICR2 | 1 | Antimicrobials |
| PLSCR1 | 5359 | phospholipid scramblase 1 | MMTRA1B | 3 | Antimicrobials |
| BACH2 | 60468 | BTB domain and CNC homolog 2 | BTBD25\|IMD60 | 6 | Antimicrobials |
| TANK | 10010 | TRAF family member associated NFKB activator | I-TRAF\|ITRAF\|TRAF2 | 2 | Antimicrobials |
| PIK3CG | 5294 | phosphatidylinositol-4,5-bisphosphate 3-kinase catalytic subunit gamma | PI3CG\|PI3K\|PI3Kgamma\|PIK3\|p110gamma\|p120-PI3K | 7 | Antimicrobials |
| ARRB1 | 408 | arrestin beta 1 | ARB1\|ARR1 | 11 | Antimicrobials |
| RSAD2 | 91543 | radical S-adenosyl methionine domain containing 2 | 2510004L01Rik\|cig33\|cig5\|vig1 | 2 | Antimicrobials |
| STAB2 | 55576 | stabilin 2 | FEEL2\|FELE-2\|FELL2\|FEX2\|HARE\|SCARH1 | 12 | Antimicrobials |
| TBK1 | 29110 | TANK binding kinase 1 | FTDALS4\|IIAE8\|NAK\|T2K | 12 | Antimicrobials |
| PDYN | 5173 | prodynorphin | ADCA\|PENKB\|SCA23 | 20 | Antimicrobials |
| PDGFRB | 5159 | platelet derived growth factor receptor beta | CD140B\|IBGC4\|IMF1\|JTK12\|KOGS\|PDGFR\|PDGFR-1\|PDGFR1\|PENTT | 5 | Antimicrobials |
| PDCD1 | 5133 | programmed cell death 1 | CD279\|PD-1\|PD1\|SLEB2\|hPD-1\|hPD-l\|hSLE1 | 2 | Antimicrobials |
| PCSK2 | 5126 | proprotein convertase subtilisin/kexin type 2 | NEC 2\|NEC-2\|NEC2\|PC2\|SPC2 | 20 | Antimicrobials |
| PCSK1 | 5122 | proprotein convertase subtilisin/kexin type 1 | BMIQ12\|NEC1\|PC1\|PC3\|SPC3 | 5 | Antimicrobials |
| ARG2 | 384 | arginase 2 | - | 14 | Antimicrobials |
| AQP9 | 366 | aquaporin 9 | AQP-9\|HsT17287\|SSC1\|T17287 | 15 | Antimicrobials |
| FASLG | 356 | Fas ligand | ALPS1B\|APT1LG1\|APTL\|CD178\|CD95-L\|CD95L\|FASL\|TNFSF6\|TNLG1A | 1 | Antimicrobials |
| APOH | 350 | apolipoprotein H | B2G1\|B2GP1\|BG | 17 | Antimicrobials |
| BIRC5 | 332 | baculoviral IAP repeat containing 5 | API4\|EPR-1 | 17 | Antimicrobials |
| ANXA6 | 309 | annexin A6 | ANX6\|CBP68\|CPB-II\|p68\|p70 | 5 | Antimicrobials |
| IL22 | 50616 | interleukin 22 | IL-21\|IL-22\|IL-D110\|IL-TIF\|ILTIF\|TIFIL-23\|TIFa\|zcyto18 | 12 | Antimicrobials |
| VTN | 7448 | vitronectin | V75\|VN\|VNT | 17 | Antimicrobials |
| VIM | 7431 | vimentin | - | 10 | Antimicrobials |
| VCAM1 | 7412 | vascular cell adhesion molecule 1 | CD106\|INCAM-100 | 1 | Antimicrobials |
| PRDX1 | 5052 | peroxiredoxin 1 | MSP23\|NKEF-A\|NKEFA\|PAG\|PAGA\|PAGB\|PRX1\|PRXI\|TDPX2 | 1 | Antimicrobials |
| GFAP | 2670 | glial fibrillary acidic protein | ALXDRD | 17 | Antimicrobials |
| GBP2 | 2634 | guanylate binding protein 2 | - | 1 | Antimicrobials |
| ALB | 213 | albumin | HSA\|PRO0883\|PRO0903\|PRO1341 | 4 | Antimicrobials |
| SLC29A3 | 55315 | solute carrier family 29 member 3 | ENT3\|HCLAP\|HJCD\|PHID | 10 | Antimicrobials |
| OAS1 | 4938 | 2'-5'-oligoadenylate synthetase 1 | E18/E16\|IFI-4\|OIAS\|OIASI | 12 | Antimicrobials |
| AGER | 177 | advanced glycosylation end-product specific receptor | RAGE\|SCARJ1 | 6 | Antimicrobials |
| UNC93B1 | 81622 | unc-93 homolog B1, TLR signaling regulator | IIAE1\|UNC93\|UNC93B\|Unc-93B1 | 11 | Antimicrobials |
| TNFSF4 | 7292 | TNF superfamily member 4 | CD134L\|CD252\|GP34\|OX-40L\|OX4OL\|TNLG2B\|TXGP1 | 1 | Antimicrobials |
| NOS1 | 4842 | nitric oxide synthase 1 | IHPS1\|N-NOS\|NC-NOS\|NOS\|bNOS\|nNOS | 12 | Antimicrobials |
| ACTG1 | 71 | actin gamma 1 | ACT\|ACTG\|DFNA20\|DFNA26\|HEL-176 | 17 | Antimicrobials |
| ACTA1 | 58 | actin alpha 1, skeletal muscle | ACTA\|ASMA\|CFTD\|CFTD1\|CFTDM\|MPFD\|NEM1\|NEM2\|NEM3\|SHPM | 1 | Antimicrobials |
| ACO1 | 48 | aconitase 1 | ACONS\|HEL60\|IREB1\|IREBP\|IREBP1\|IRP1 | 9 | Antimicrobials |
| SERPINA3 | 12 | serpin family A member 3 | AACT\|ACT\|GIG24\|GIG25 | 14 | Antimicrobials |
| CXCR1 | 3577 | C-X-C motif chemokine receptor 1 | C-C\|C-C-CKR-1\|CD128\|CD181\|CDw128a\|CKR-1\|CMKAR1\|IL8R1\|IL8RA\|IL8RBA | 2 | Antimicrobials |
| CCL15 | 6359 | C-C motif chemokine ligand 15 | HCC-2\|HMRP-2B\|LKN-1\|LKN1\|MIP-1 delta\|MIP-1D\|MIP-5\|MRP-2B\|NCC-3\|NCC3\|SCYA15\|SCYL3\|SY15 | 17 | Antimicrobials |
| CCL14 | 6358 | C-C motif chemokine ligand 14 | CC-1\|CC-3\|CKB1\|HCC-1\|HCC-1(1-74)\|HCC-1/HCC-3\|HCC-3\|MCIF\|NCC-2\|NCC2\|SCYA14\|SCYL2\|SY14 | 17 | Antimicrobials |
| CCL16 | 6360 | C-C motif chemokine ligand 16 | CKb12\|HCC-4\|ILINCK\|LCC-1\|LEC\|LMC\|Mtn-1\|NCC-4\|NCC4\|SCYA16\|SCYL4 | 17 | Antimicrobials |
| CCL19 | 6363 | C-C motif chemokine ligand 19 | CKb11\|ELC\|MIP-3b\|MIP3B\|SCYA19 | 9 | Antimicrobials |
| CCL18 | 6362 | C-C motif chemokine ligand 18 | AMAC-1\|AMAC1\|CKb7\|DC-CK1\|DCCK1\|MIP-4\|PARC\|SCYA18 | 17 | Antimicrobials |
| CCL17 | 6361 | C-C motif chemokine ligand 17 | A-152E5.3\|ABCD-2\|SCYA17\|TARC | 16 | Antimicrobials |
| CCL26 | 10344 | C-C motif chemokine ligand 26 | IMAC\|MIP-4a\|MIP-4alpha\|SCYA26\|TSC-1 | 7 | Antimicrobials |
| CCL22 | 6367 | C-C motif chemokine ligand 22 | A-152E5.1\|ABCD-1\|DC/B-CK\|MDC\|SCYA22\|STCP-1 | 16 | Antimicrobials |
| CCR3 | 1232 | C-C motif chemokine receptor 3 | C C CKR3\|CC-CKR-3\|CD193\|CKR 3\|CKR3\|CMKBR3 | 3 | Antimicrobials |
| CCL4L1 | 388372 | C-C motif chemokine ligand 4 like 1 | AT744.2\|CCL4L\|LAG-1\|LAG1\|MIP-1-beta\|SCYA4L\|SCYA4L1\|SCYA4L2 | 17 | Antimicrobials |
| ACKR2 | 1238 | atypical chemokine receptor 2 | CCBP2\|CCR10\|CCR9\|CMKBR9\|D6\|hD6 | 3 | Antimicrobials |
| CCR7 | 1236 | C-C motif chemokine receptor 7 | BLR2\|CC-CKR-7\|CCR-7\|CD197\|CDw197\|CMKBR7\|EBI1 | 17 | Antimicrobials |
| CCL27 | 10850 | C-C motif chemokine ligand 27 | ALP\|CTACK\|CTAK\|ESKINE\|ILC\|PESKY\|SCYA27 | 9 | Antimicrobials |
| CCR8 | 1237 | C-C motif chemokine receptor 8 | CC-CKR-8\|CCR-8\|CDw198\|CKRL1\|CMKBR8\|CMKBRL2\|CY6\|GPRCY6\|TER1 | 3 | Antimicrobials |
| ACKR4 | 51554 | atypical chemokine receptor 4 | CC-CKR-11\|CCBP2\|CCR-11\|CCR10\|CCR11\|CCRL1\|CCX CKR\|CCX-CKR\|CKR-11\|PPR1\|VSHK1 | 3 | Antimicrobials |
| CCL2 | 6347 | C-C motif chemokine ligand 2 | GDCF-2\|HC11\|HSMCR30\|MCAF\|MCP-1\|MCP1\|SCYA2\|SMC-CF | 17 | Antimicrobials |
| CCL21 | 6366 | C-C motif chemokine ligand 21 | 6Ckine\|CKb9\|ECL\|SCYA21\|SLC\|TCA4 | 9 | Antimicrobials |
| CCL7 | 6354 | C-C motif chemokine ligand 7 | FIC\|MARC\|MCP-3\|MCP3\|NC28\|SCYA6\|SCYA7 | 17 | Antimicrobials |
| CCL3 | 6348 | C-C motif chemokine ligand 3 | G0S19-1\|LD78ALPHA\|MIP-1-alpha\|MIP1A\|SCYA3 | 17 | Antimicrobials |
| CCL11 | 6356 | C-C motif chemokine ligand 11 | SCYA11 | 17 | Antimicrobials |
| CCR5 | 1234 | C-C motif chemokine receptor 5 | CC-CKR-5\|CCCKR5\|CCR-5\|CD195\|CKR-5\|CKR5\|CMKBR5\|IDDM22 | 3 | Antimicrobials |
| CCL23 | 6368 | C-C motif chemokine ligand 23 | CK-BETA-8\|CKb8\|Ckb-8\|Ckb-8-1\|MIP-3\|MIP3\|MPIF-1\|SCYA23\|hmrp-2a | 17 | Antimicrobials |
| CCL25 | 6370 | C-C motif chemokine ligand 25 | Ckb15\|SCYA25\|TECK | 19 | Antimicrobials |
| CCL3L3 | 414062 | C-C motif chemokine ligand 3 like 3 | 464.2\|D17S1718\|G0S19-2\|LD78\|LD78BETA\|SCYA3L\|SCYA3L1 | 17 | Antimicrobials |
| CCL4L2 | 9560 | C-C motif chemokine ligand 4 like 2 | AT744.2\|CCL4L\|SCYA4L\|SCYQ4L2 | 17 | Antimicrobials |
| CCL3L1 | 6349 | C-C motif chemokine ligand 3 like 1 | 464.2\|D17S1718\|G0S19-2\|LD78\|LD78-beta(1-70)\|LD78BETA\|MIP1AP\|SCYA3L\|SCYA3L1 | 17 | Antimicrobials |
| CCR1 | 1230 | C-C motif chemokine receptor 1 | CD191\|CKR-1\|CKR1\|CMKBR1\|HM145\|MIP1aR\|SCYAR1 | 3 | Antimicrobials |
| CCL24 | 6369 | C-C motif chemokine ligand 24 | Ckb-6\|MPIF-2\|MPIF2\|SCYA24 | 7 | Antimicrobials |
| XCL2 | 6846 | X-C motif chemokine ligand 2 | SCM-1b\|SCM1B\|SCYC2 | 1 | Antimicrobials |
| CXCR4 | 7852 | C-X-C motif chemokine receptor 4 | CD184\|D2S201E\|FB22\|HM89\|HSY3RR\|LAP-3\|LAP3\|LCR1\|LESTR\|NPY3R\|NPYR\|NPYRL\|NPYY3R\|WHIM\|WHIMS | 2 | Antimicrobials |
| CXCR6 | 10663 | C-X-C motif chemokine receptor 6 | BONZO\|CD186\|STRL33\|TYMSTR | 3 | Antimicrobials |
| CCR4 | 1233 | C-C motif chemokine receptor 4 | CC-CKR-4\|CD194\|CKR4\|CMKBR4\|ChemR13\|HGCN:14099\|K5-5 | 3 | Antimicrobials |
| TAFA5 | 25817 | TAFA chemokine like family member 5 | FAM19A5\|QLLK5208\|TAFA-5\|UNQ5208 | 22 | Antimicrobials |
| TAFA3 | 284467 | TAFA chemokine like family member 3 | FAM19A3\|TAFA-3 | 1 | Antimicrobials |
| TAFA4 | 151647 | TAFA chemokine like family member 4 | FAM19A4\|TAFA-4 | 3 | Antimicrobials |
| TAFA1 | 407738 | TAFA chemokine like family member 1 | FAM19A1\|TAFA-1 | 3 | Antimicrobials |
| TAFA2 | 338811 | TAFA chemokine like family member 2 | FAM19A2\|TAFA-2 | 12 | Antimicrobials |
| CCL15-CCL14 | 348249 | CCL15-CCL14 readthrough (NMD candidate) | CCL15\|HCC-2\|LKN-1\|MIP-5\|MIP5\|Mrp-2b\|NCC-3\|NCC3\|SCYA15 | 17 | Antimicrobials |
| PTK2B | 2185 | protein tyrosine kinase 2 beta | CADTK\|CAKB\|FADK2\|FAK2\|PKB\|PTK\|PYK2\|RAFTK | 8 | Antimicrobials |
| IL4 | 3565 | interleukin 4 | BCGF-1\|BCGF1\|BSF-1\|BSF1\|IL-4 | 5 | Antimicrobials |
| CDH1 | 999 | cadherin 1 | Arc-1\|BCDS1\|CD324\|CDHE\|ECAD\|LCAM\|UVO | 16 | Antimicrobials |
| LTBP1 | 4052 | latent transforming growth factor beta binding protein 1 | - | 2 | Antimicrobials |
| IL13 | 3596 | interleukin 13 | IL-13\|P600 | 5 | Antimicrobials |
| IL10 | 3586 | interleukin 10 | CSIF\|GVHDS\|IL-10\|IL10A\|TGIF | 1 | Antimicrobials |
| IL2 | 3558 | interleukin 2 | IL-2\|TCGF\|lymphokine | 4 | Antimicrobials |
| PPARG | 5468 | peroxisome proliferator activated receptor gamma | CIMT1\|GLM1\|NR1C3\|PPARG1\|PPARG2\|PPARG5\|PPARgamma | 3 | Antimicrobials |
| FGR | 2268 | FGR proto-oncogene, Src family tyrosine kinase | SRC2\|c-fgr\|c-src2\|p55-Fgr\|p55c-fgr\|p58-Fgr\|p58c-fgr | 1 | Antimicrobials |
| MIF | 4282 | macrophage migration inhibitory factor | GIF\|GLIF\|MMIF | 22 | Antimicrobials |
| CRP | 1401 | C-reactive protein | PTX1 | 1 | Antimicrobials |
| JAK2 | 3717 | Janus kinase 2 | JTK10\|THCYT3 | 9 | Antimicrobials |
| PTK2 | 5747 | protein tyrosine kinase 2 | FADK\|FAK\|FAK1\|FRNK\|PPP1R71\|p125FAK\|pp125FAK | 8 | Antimicrobials |
| PTGDR | 5729 | prostaglandin D2 receptor | AS1\|ASRT1\|DP\|DP1\|PTGDR1 | 14 | Antimicrobials |
| CD86 | 942 | CD86 molecule | B7-2\|B7.2\|B70\|CD28LG2\|LAB72 | 3 | Antimicrobials |
| HCK | 3055 | HCK proto-oncogene, Src family tyrosine kinase | JTK9\|p59Hck\|p61Hck | 20 | Antimicrobials |
| VDR | 7421 | vitamin D receptor | NR1I1\|PPP1R163 | 12 | Antimicrobials |
| OLR1 | 4973 | oxidized low density lipoprotein receptor 1 | CLEC8A\|LOX1\|LOXIN\|SCARE1\|SLOX1 | 12 | Antimicrobials |
| GRK2 | 156 | G protein-coupled receptor kinase 2 | ADRBK1\|BARK1\|BETA-ARK1 | 11 | Antimicrobials |
| TXK | 7294 | TXK tyrosine kinase | BTKL\|PSCTK5\|PTK4\|RLK\|TKL | 4 | Antimicrobials |
| RNASE2 | 6036 | ribonuclease A family member 2 | EDN\|RAF3\|RNS2 | 14 | Antimicrobials |
| CD79A | 973 | CD79a molecule | IGA\|MB-1 | 19 | BCRSignalingPathway |
| CD79B | 974 | CD79b molecule | AGM6\|B29\|IGB | 17 | BCRSignalingPathway |
| LYN | 4067 | LYN proto-oncogene, Src family tyrosine kinase | JTK8\|p53Lyn\|p56Lyn | 8 | BCRSignalingPathway |
| SYK | 6850 | spleen associated tyrosine kinase | p72-Syk | 9 | BCRSignalingPathway |
| BTK | 695 | Bruton tyrosine kinase | AGMX1\|AT\|ATK\|BPK\|IGHD3\|IMD1\|PSCTK1\|XLA | X | BCRSignalingPathway |
| BLNK | 29760 | B cell linker | AGM4\|BASH\|BLNK-S\|LY57\|SLP-65\|SLP65\|bca | 10 | BCRSignalingPathway |
| VAV3 | 10451 | vav guanine nucleotide exchange factor 3 | - | 1 | BCRSignalingPathway |
| VAV1 | 7409 | vav guanine nucleotide exchange factor 1 | VAV | 19 | BCRSignalingPathway |
| VAV2 | 7410 | vav guanine nucleotide exchange factor 2 | VAV-2 | 9 | BCRSignalingPathway |
| RAC1 | 5879 | Rac family small GTPase 1 | MIG5\|MRD48\|Rac-1\|TC-25\|p21-Rac1 | 7 | BCRSignalingPathway |
| RAC2 | 5880 | Rac family small GTPase 2 | EN-7\|Gx\|HSPC022\|p21-Rac2 | 22 | BCRSignalingPathway |
| RAC3 | 5881 | Rac family small GTPase 3 | - | 17 | BCRSignalingPathway |
| PPP3CA | 5530 | protein phosphatase 3 catalytic subunit alpha | ACCIID\|CALN\|CALNA\|CALNA1\|CCN1\|CNA1\|IECEE\|IECEE1\|PPP2B | 4 | BCRSignalingPathway |
| PPP3CB | 5532 | protein phosphatase 3 catalytic subunit beta | CALNA2\|CALNB\|CNA2\|PP2Bbeta | 10 | BCRSignalingPathway |
| PPP3CC | 5533 | protein phosphatase 3 catalytic subunit gamma | CALNA3\|CNA3\|PP2Bgamma | 8 | BCRSignalingPathway |
| CHP1 | 11261 | calcineurin like EF-hand protein 1 | CHP\|SLC9A1BP\|SPAX9\|Sid470p\|p22\|p24 | 15 | BCRSignalingPathway |
| PPP3R1 | 5534 | protein phosphatase 3 regulatory subunit B, alpha | CALNB1\|CNB\|CNB1 | 2 | BCRSignalingPathway |
| PPP3R2 | 5535 | protein phosphatase 3 regulatory subunit B, beta | PPP3RL | 9 | BCRSignalingPathway |
| CHP2 | 63928 | calcineurin like EF-hand protein 2 | - | 16 | BCRSignalingPathway |
| NFAT5 | 10725 | nuclear factor of activated T cells 5 | NF-AT5\|NFATL1\|NFATZ\|OREBP\|TONEBP | 16 | BCRSignalingPathway |
| NFATC1 | 4772 | nuclear factor of activated T cells 1 | NF-ATC\|NF-ATc1.2\|NFAT2\|NFATc | 18 | BCRSignalingPathway |
| NFATC2 | 4773 | nuclear factor of activated T cells 2 | NFAT1\|NFATP | 20 | BCRSignalingPathway |
| NFATC3 | 4775 | nuclear factor of activated T cells 3 | NF-AT4c\|NFAT4\|NFATX | 16 | BCRSignalingPathway |
| NFATC4 | 4776 | nuclear factor of activated T cells 4 | NF-AT3\|NF-ATC4\|NFAT3 | 14 | BCRSignalingPathway |
| HRAS | 3265 | HRas proto-oncogene, GTPase | C-BAS/HAS\|C-H-RAS\|C-HA-RAS1\|CTLO\|H-RASIDX\|HAMSV\|HRAS1\|RASH1\|p21ras | 11 | BCRSignalingPathway |
| KRAS | 3845 | KRAS proto-oncogene, GTPase | C-K-RAS\|C-K-RAS\|CFC2\|K-RAS2A\|K-RAS2B\|K-RAS4A\|K-RAS4B\|K-Ras\|K-Ras 2\|KI-RAS\|KRAS1\|KRAS2\|NS\|NS3\|OES\|RALD\|RASK2\|c-Ki-ras\|c-Ki-ras2 | 12 | BCRSignalingPathway |
| NRAS | 4893 | NRAS proto-oncogene, GTPase | ALPS4\|CMNS\|N-ras\|NCMS\|NRAS1\|NS6 | 1 | BCRSignalingPathway |
| FOS | 2353 | Fos proto-oncogene, AP-1 transcription factor subunit | AP-1\|C-FOS\|p55 | 14 | BCRSignalingPathway |
| CARD11 | 84433 | caspase recruitment domain family member 11 | BENTA\|BIMP3\|CARMA1\|IMD11\|IMD11A\|PPBL | 7 | BCRSignalingPathway |
| BCL10 | 8915 | BCL10 immune signaling adaptor | CARMEN\|CIPER\|CLAP\|IMD37\|c-E10\|mE10 | 1 | BCRSignalingPathway |
| MALT1 | 10892 | MALT1 paracaspase | IMD12\|MLT\|MLT1\|PCASP1 | 18 | BCRSignalingPathway |
| CHUK | 1147 | component of inhibitor of nuclear factor kappa B kinase complex | IKBKA\|IKK-alpha\|IKK1\|IKKA\|NFKBIKA\|TCF16 | 10 | BCRSignalingPathway |
| IKBKB | 3551 | inhibitor of nuclear factor kappa B kinase subunit beta | IKK-beta\|IKK2\|IKKB\|IMD15\|IMD15A\|IMD15B\|NFKBIKB | 8 | BCRSignalingPathway |
| IKBKG | 8517 | inhibitor of nuclear factor kappa B kinase regulatory subunit gamma | AMCBX1\|EDAID1\|FIP-3\|FIP3\|Fip3p\|IKK-gamma\|IKKAP1\|IKKG\|IMD33\|IP\|IP1\|IP2\|IPD2\|NEMO\|ZC2HC9 | X | BCRSignalingPathway |
| NFKBIA | 4792 | NFKB inhibitor alpha | EDAID2\|IKBA\|MAD-3\|NFKBI | 14 | BCRSignalingPathway |
| NFKBIB | 4793 | NFKB inhibitor beta | IKBB\|TRIP9 | 19 | BCRSignalingPathway |
| NFKBIE | 4794 | NFKB inhibitor epsilon | IKBE | 6 | BCRSignalingPathway |
| CD19 | 930 | CD19 molecule | B4\|CVID3 | 16 | BCRSignalingPathway |
| CR2 | 1380 | complement C3d receptor 2 | C3DR\|CD21\|CR\|CVID7\|SLEB9 | 1 | BCRSignalingPathway |
| PIK3R5 | 23533 | phosphoinositide-3-kinase regulatory subunit 5 | F730038I15Rik\|FOAP-2\|P101-PI3K\|p101 | 17 | BCRSignalingPathway |
| PIK3R1 | 5295 | phosphoinositide-3-kinase regulatory subunit 1 | AGM7\|GRB1\|IMD36\|p85\|p85-ALPHA | 5 | BCRSignalingPathway |
| PIK3R2 | 5296 | phosphoinositide-3-kinase regulatory subunit 2 | MPPH\|MPPH1\|P85B\|p85\|p85-BETA | 19 | BCRSignalingPathway |
| PIK3R3 | 8503 | phosphoinositide-3-kinase regulatory subunit 3 | p55\|p55-GAMMA\|p55PIK | 1 | BCRSignalingPathway |
| PIK3CA | 5290 | phosphatidylinositol-4,5-bisphosphate 3-kinase catalytic subunit alpha | CLAPO\|CLOVE\|CWS5\|MCAP\|MCM\|MCMTC\|PI3K\|PI3K-alpha\|p110-alpha | 3 | BCRSignalingPathway |
| PIK3CB | 5291 | phosphatidylinositol-4,5-bisphosphate 3-kinase catalytic subunit beta | P110BETA\|PI3K\|PI3KBETA\|PIK3C1 | 3 | BCRSignalingPathway |
| PIK3CD | 5293 | phosphatidylinositol-4,5-bisphosphate 3-kinase catalytic subunit delta | APDS\|IMD14\|P110DELTA\|PI3K\|p110D | 1 | BCRSignalingPathway |
| AKT3 | 10000 | AKT serine/threonine kinase 3 | MPPH\|MPPH2\|PKB-GAMMA\|PKBG\|PRKBG\|RAC-PK-gamma\|RAC-gamma\|STK-2 | 1 | BCRSignalingPathway |
| AKT1 | 207 | AKT serine/threonine kinase 1 | AKT\|CWS6\|PKB\|PKB-ALPHA\|PRKBA\|RAC\|RAC-ALPHA | 14 | BCRSignalingPathway |
| AKT2 | 208 | AKT serine/threonine kinase 2 | HIHGHH\|PKBB\|PKBBETA\|PRKBB\|RAC-BETA | 19 | BCRSignalingPathway |
| GSK3B | 2932 | glycogen synthase kinase 3 beta | - | 3 | BCRSignalingPathway |
| INPP5D | 3635 | inositol polyphosphate-5-phosphatase D | SHIP\|SHIP-1\|SHIP1\|SIP-145\|hp51CN\|p150Ship | 2 | BCRSignalingPathway |
| CD22 | 933 | CD22 molecule | SIGLEC-2\|SIGLEC2 | 19 | BCRSignalingPathway |
| CD72 | 971 | CD72 molecule | CD72b\|LYB2 | 9 | BCRSignalingPathway |
| PTPN6 | 5777 | protein tyrosine phosphatase non-receptor type 6 | HCP\|HCPH\|HPTP1C\|PTP-1C\|SH-PTP1\|SHP-1\|SHP-1L\|SHP1 | 12 | BCRSignalingPathway |
| LILRB3 | 11025 | leukocyte immunoglobulin like receptor B3 | CD85A\|HL9\|ILT-5\|ILT5\|LILRA6\|LIR-3\|LIR3\|PIR-B\|PIRB | 19 | BCRSignalingPathway |
| FCGR2B | 2213 | Fc fragment of IgG receptor IIb | CD32\|CD32B\|FCG2\|FCGR2\|FCGR2C\|FcRII-c\|IGFR2 | 1 | BCRSignalingPathway |
| RASGRP3 | 25780 | RAS guanyl releasing protein 3 | GRP3 | 2 | BCRSignalingPathway |
| PLCG2 | 5336 | phospholipase C gamma 2 | APLAID\|FCAS3\|PLC-IV\|PLC-gamma-2 | 16 | BCRSignalingPathway |
| PRKCB | 5579 | protein kinase C beta | PKC-beta\|PKCB\|PKCI(2)\|PKCbeta\|PRKCB1\|PRKCB2 | 16 | BCRSignalingPathway |
| IFITM1 | 8519 | interferon induced transmembrane protein 1 | 9-27\|CD225\|DSPA2a\|IFI17\|LEU13 | 11 | BCRSignalingPathway |
| IGH | 3492 | immunoglobulin heavy locus | IGD1\|IGH.1@\|IGH@\|IGHD@\|IGHDY1\|IGHJ\|IGHJ@\|IGHV\|IGHV@ | 14 | BCRSignalingPathway |
| IGHA1 | 3493 | immunoglobulin heavy constant alpha 1 | IgA1 | 14 | BCRSignalingPathway |
| IGHA2 | 3494 | immunoglobulin heavy constant alpha 2 (A2m marker) | - | 14 | BCRSignalingPathway |
| IGHD | 3495 | immunoglobulin heavy constant delta | - | 14 | BCRSignalingPathway |
| IGHD1-1 | 28510 | immunoglobulin heavy diversity 1-1 | IGHD11 | 14 | BCRSignalingPathway |
| IGHD1-14 | 28508 | immunoglobulin heavy diversity 1-14 (non-functional) | DM2\|IGHD114 | 14 | BCRSignalingPathway |
| IGHD1-20 | 28507 | immunoglobulin heavy diversity 1-20 | IGHD120 | 14 | BCRSignalingPathway |
| IGHD1-26 | 28506 | immunoglobulin heavy diversity 1-26 | IGHD126 | 14 | BCRSignalingPathway |
| IGHD1-7 | 28509 | immunoglobulin heavy diversity 1-7 | DM1\|IGHD17 | 14 | BCRSignalingPathway |
| IGHD2-15 | 28503 | immunoglobulin heavy diversity 2-15 | D2\|IGHD215 | 14 | BCRSignalingPathway |
| IGHD2-2 | 28505 | immunoglobulin heavy diversity 2-2 | IGHD22 | 14 | BCRSignalingPathway |
| IGHD2-21 | 28502 | immunoglobulin heavy diversity 2-21 | IGHD221 | 14 | BCRSignalingPathway |
| IGHD2-8 | 28504 | immunoglobulin heavy diversity 2-8 | DLR1\|IGHD28 | 14 | BCRSignalingPathway |
| IGHD3-10 | 28499 | immunoglobulin heavy diversity 3-10 | DXP'1\|IGHD310 | 14 | BCRSignalingPathway |
| IGHD3-16 | 28498 | immunoglobulin heavy diversity 3-16 | IGHD316 | 14 | BCRSignalingPathway |
| IGHD3-22 | 28497 | immunoglobulin heavy diversity 3-22 | IGHD322 | 14 | BCRSignalingPathway |
| IGHD3-3 | 28501 | immunoglobulin heavy diversity 3-3 | DXP4\|IGHD33 | 14 | BCRSignalingPathway |
| IGHD3-9 | 28500 | immunoglobulin heavy diversity 3-9 | DXP1\|IGHD39 | 14 | BCRSignalingPathway |
| IGHD4-11 | 28495 | immunoglobulin heavy diversity 4-11 (non-functional) | DA1\|IGHD411 | 14 | BCRSignalingPathway |
| IGHD4-17 | 28494 | immunoglobulin heavy diversity 4-17 | IGHD417 | 14 | BCRSignalingPathway |
| IGHD4-23 | 28493 | immunoglobulin heavy diversity 4-23 (non-functional) | IGHD423 | 14 | BCRSignalingPathway |
| IGHD4-4 | 28496 | immunoglobulin heavy diversity 4-4 | DA4\|IGHD44 | 14 | BCRSignalingPathway |
| IGHD5-12 | 28491 | immunoglobulin heavy diversity 5-12 | DK1\|IGHD512 | 14 | BCRSignalingPathway |
| IGHD5-18 | 28490 | immunoglobulin heavy diversity 5-18 | IGHD518 | 14 | BCRSignalingPathway |
| IGHD5-24 | 28489 | immunoglobulin heavy diversity 5-24 (non-functional) | IGHD524 | 14 | BCRSignalingPathway |
| IGHD5-5 | 28492 | immunoglobulin heavy diversity 5-5 | DK4\|IGHD55 | 14 | BCRSignalingPathway |
| IGHD6-13 | 28487 | immunoglobulin heavy diversity 6-13 | DN1\|IGHD613 | 14 | BCRSignalingPathway |
| IGHD6-19 | 28486 | immunoglobulin heavy diversity 6-19 | IGHD619 | 14 | BCRSignalingPathway |
| IGHD6-25 | 28485 | immunoglobulin heavy diversity 6-25 | IGHD625 | 14 | BCRSignalingPathway |
| IGHD6-6 | 28488 | immunoglobulin heavy diversity 6-6 | D(N4)\|IGHD66 | 14 | BCRSignalingPathway |
| IGHD7-27 | 28484 | immunoglobulin heavy diversity 7-27 | DHQ52\|IGHD727 | 14 | BCRSignalingPathway |
| IGHE | 3497 | immunoglobulin heavy constant epsilon | IgE | 14 | BCRSignalingPathway |
| IGHG1 | 3500 | immunoglobulin heavy constant gamma 1 (G1m marker) | - | 14 | BCRSignalingPathway |
| IGHG2 | 3501 | immunoglobulin heavy constant gamma 2 (G2m marker) | - | 14 | BCRSignalingPathway |
| IGHG3 | 3502 | immunoglobulin heavy constant gamma 3 (G3m marker) | IgG3 | 14 | BCRSignalingPathway |
| IGHG4 | 3503 | immunoglobulin heavy constant gamma 4 (G4m marker) | - | 14 | BCRSignalingPathway |
| IGHJ1 | 28483 | immunoglobulin heavy joining 1 | JH1 | 14 | BCRSignalingPathway |
| IGHJ2 | 28481 | immunoglobulin heavy joining 2 | JH2 | 14 | BCRSignalingPathway |
| IGHJ3 | 28479 | immunoglobulin heavy joining 3 | JH3b | 14 | BCRSignalingPathway |
| IGHJ4 | 28477 | immunoglobulin heavy joining 4 | JH4b | 14 | BCRSignalingPathway |
| IGHJ5 | 28476 | immunoglobulin heavy joining 5 | JH5b | 14 | BCRSignalingPathway |
| IGHJ6 | 28475 | immunoglobulin heavy joining 6 | JH6b | 14 | BCRSignalingPathway |
| IGHM | 3507 | immunoglobulin heavy constant mu | AGM1\|MU\|VH | 14 | BCRSignalingPathway |
| IGHV1-18 | 28468 | immunoglobulin heavy variable 1-18 | IGHV118 | 14 | BCRSignalingPathway |
| IGHV1-2 | 28474 | immunoglobulin heavy variable 1-2 | IGHV12\|V35 | 14 | BCRSignalingPathway |
| IGHV1-24 | 28467 | immunoglobulin heavy variable 1-24 | IGHV124\|VH | 14 | BCRSignalingPathway |
| IGHV1-3 | 28473 | immunoglobulin heavy variable 1-3 | IGHV13\|VI-3B | 14 | BCRSignalingPathway |
| IGHV1-45 | 28466 | immunoglobulin heavy variable 1-45 | IGHV145\|VH | 14 | BCRSignalingPathway |
| IGHV1-46 | 28465 | immunoglobulin heavy variable 1-46 | IGHV146 | 14 | BCRSignalingPathway |
| IGHV1-58 | 28464 | immunoglobulin heavy variable 1-58 | IGHV158\|VH | 14 | BCRSignalingPathway |
| IGHV1-69 | 28461 | immunoglobulin heavy variable 1-69 | IGHV1-E\|IGHV169\|IGHV1E | 14 | BCRSignalingPathway |
| IGHV1-8 | 28472 | immunoglobulin heavy variable 1-8 | IGHV18 | 14 | BCRSignalingPathway |
| IGHV1-38-4 | 28460 | immunoglobulin heavy variable 1-38-4 (non-functional) | IGHV1-C\|IGHV1C | 14 | BCRSignalingPathway |
| IGHV1-69-2 | 28458 | immunoglobulin heavy variable 1-69-2 | IGHV1-F\|IGHV1F | 14 | BCRSignalingPathway |
| IGHV2-26 | 28455 | immunoglobulin heavy variable 2-26 | IGHV226\|VH | 14 | BCRSignalingPathway |
| IGHV2-5 | 28457 | immunoglobulin heavy variable 2-5 | IGHV25\|VH | 14 | BCRSignalingPathway |
| IGHV2-70 | 28454 | immunoglobulin heavy variable 2-70 | IGHV270\|VH | 14 | BCRSignalingPathway |
| IGHV3-11 | 28450 | immunoglobulin heavy variable 3-11 | IGHV311\|VH | 14 | BCRSignalingPathway |
| IGHV3-13 | 28449 | immunoglobulin heavy variable 3-13 | IGHV313 | 14 | BCRSignalingPathway |
| IGHV3-15 | 28448 | immunoglobulin heavy variable 3-15 | IGHV315\|VH | 14 | BCRSignalingPathway |
| IGHV3-16 | 28447 | immunoglobulin heavy variable 3-16 (non-functional) | IGHV316\|VH | 14 | BCRSignalingPathway |
| IGHV3-20 | 28445 | immunoglobulin heavy variable 3-20 | IGHV320\|VH | 14 | BCRSignalingPathway |
| IGHV3-21 | 28444 | immunoglobulin heavy variable 3-21 | IGHV321\|VH | 14 | BCRSignalingPathway |
| IGHV3-23 | 28442 | immunoglobulin heavy variable 3-23 | DP47\|IGHV323\|V3-23\|VH26 | 14 | BCRSignalingPathway |
| IGHV3-30 | 28439 | immunoglobulin heavy variable 3-30 | IGHV330\|VH | 14 | BCRSignalingPathway |
| IGHV3-30-3 | 57290 | immunoglobulin heavy variable 3-30-3 | IGHV3-3\|IGHV3303 | 14 | BCRSignalingPathway |
| IGHV3-30-5 | 89770 | immunoglobulin heavy variable 3-30-5 | IGHV3-3\|IGHV3305 | 14 | BCRSignalingPathway |
| IGHV3-33 | 28434 | immunoglobulin heavy variable 3-33 | IGHV333\|VH | 14 | BCRSignalingPathway |
| IGHV3-35 | 28432 | immunoglobulin heavy variable 3-35 (non-functional) | IGHV335\|VH | 14 | BCRSignalingPathway |
| IGHV3-38 | 28429 | immunoglobulin heavy variable 3-38 (non-functional) | IGHV338\|VH | 14 | BCRSignalingPathway |
| IGHV3-43 | 28426 | immunoglobulin heavy variable 3-43 | IGHV343\|VH | 14 | BCRSignalingPathway |
| IGHV3-48 | 28424 | immunoglobulin heavy variable 3-48 | IGHV348\|VH | 14 | BCRSignalingPathway |
| IGHV3-49 | 28423 | immunoglobulin heavy variable 3-49 | IGHV349\|VH | 14 | BCRSignalingPathway |
| IGHV3-53 | 28420 | immunoglobulin heavy variable 3-53 | IGHV353\|VH | 14 | BCRSignalingPathway |
| IGHV3-64 | 28414 | immunoglobulin heavy variable 3-64 | IGHV364\|VH | 14 | BCRSignalingPathway |
| IGHV3-66 | 28412 | immunoglobulin heavy variable 3-66 | IGHV366\|VH | 14 | BCRSignalingPathway |
| IGHV3-7 | 28452 | immunoglobulin heavy variable 3-7 | IGHV37\|VH | 14 | BCRSignalingPathway |
| IGHV3-72 | 28410 | immunoglobulin heavy variable 3-72 | IGHV372\|VH | 14 | BCRSignalingPathway |
| IGHV3-73 | 28409 | immunoglobulin heavy variable 3-73 | IGHV373\|VH | 14 | BCRSignalingPathway |
| IGHV3-74 | 28408 | immunoglobulin heavy variable 3-74 | IGHV374\|VH | 14 | BCRSignalingPathway |
| IGHV3-9 | 28451 | immunoglobulin heavy variable 3-9 | IGHV39\|VH | 14 | BCRSignalingPathway |
| IGHV3-38-3 | 28404 | immunoglobulin heavy variable 3-38-3 (non-functional) | IGHV3-D\|IGHV3D | 14 | BCRSignalingPathway |
| IGHV3-69-1 | 28402 | immunoglobulin heavy variable 3-69-1 (pseudogene) | IGH\|IGHM\|IGHV\|IGHV3-11\|IGHV3-H\|IGHV3H\|IgVH | 14 | BCRSignalingPathway |
| IGHV4-28 | 28400 | immunoglobulin heavy variable 4-28 | IGHV428\|VH | 14 | BCRSignalingPathway |
| IGHV4-30-1 | 28399 | immunoglobulin heavy variable 4-30-1 | IGHV4-3 | 14 | BCRSignalingPathway |
| IGHV4-30-2 | 28398 | immunoglobulin heavy variable 4-30-2 | IGHV4-3\|IGHV4302 | 14 | BCRSignalingPathway |
| IGHV4-30-4 | 28397 | immunoglobulin heavy variable 4-30-4 | IGHV4-3\|IGHV4304 | 14 | BCRSignalingPathway |
| IGHV4-31 | 28396 | immunoglobulin heavy variable 4-31 | IGHV431 | 14 | BCRSignalingPathway |
| IGHV4-34 | 28395 | immunoglobulin heavy variable 4-34 | IGHV434\|VH | 14 | BCRSignalingPathway |
| IGHV4-39 | 28394 | immunoglobulin heavy variable 4-39 | IGHV439\|VH | 14 | BCRSignalingPathway |
| IGHV4-4 | 28401 | immunoglobulin heavy variable 4-4 | IGHV44\|VH | 14 | BCRSignalingPathway |
| IGHV4-59 | 28392 | immunoglobulin heavy variable 4-59 | IGHV459\|VH | 14 | BCRSignalingPathway |
| IGHV4-61 | 28391 | immunoglobulin heavy variable 4-61 | IGHV461\|VH | 14 | BCRSignalingPathway |
| IGHV4-38-2 | 28389 | immunoglobulin heavy variable 4-38-2 | IGHV4-B\|IGHV4B | 14 | BCRSignalingPathway |
| IGHV5-51 | 28388 | immunoglobulin heavy variable 5-51 | IGHV551\|VH | 14 | BCRSignalingPathway |
| IGHV5-10-1 | 28386 | immunoglobulin heavy variable 5-10-1 | IGHV5-A\|IGHV5A | 14 | BCRSignalingPathway |
| IGHV6-1 | 28385 | immunoglobulin heavy variable 6-1 | IGHV61\|VH | 14 | BCRSignalingPathway |
| IGHV7-4-1 | 57289 | immunoglobulin heavy variable 7-4-1 | IGHV7-41\|IGHV741 | 14 | BCRSignalingPathway |
| IGHV7-81 | 28378 | immunoglobulin heavy variable 7-81 (non-functional) | IGHV781 | 14 | BCRSignalingPathway |
| IGK | 50802 | immunoglobulin kappa locus | IGK@ | 2 | BCRSignalingPathway |
| IGKC | 3514 | immunoglobulin kappa constant | HCAK1\|IGKCD\|Km | 2 | BCRSignalingPathway |
| IGKDEL | 3515 | immunoglobulin kappa deleting element or like | IGKDE | 2 | BCRSignalingPathway |
| IGKJ | 7842 | - | IGKJ@ | 2 | BCRSignalingPathway |
| IGKJ1 | 28950 | immunoglobulin kappa joining 1 | J1 | 2 | BCRSignalingPathway |
| IGKJ2 | 28949 | immunoglobulin kappa joining 2 | J2 | 2 | BCRSignalingPathway |
| IGKJ3 | 28948 | immunoglobulin kappa joining 3 | J3 | 2 | BCRSignalingPathway |
| IGKJ4 | 28947 | immunoglobulin kappa joining 4 | J4 | 2 | BCRSignalingPathway |
| IGKJ5 | 28946 | immunoglobulin kappa joining 5 | J5 | 2 | BCRSignalingPathway |
| IGKV@ | 3519 | - | IGKV\|IGKV1\|IGKV1@\|IGKV2\|IGKV2@\|IGKV3\|IGKV3@ | 2 | BCRSignalingPathway |
| IGKV1-12 | 28940 | immunoglobulin kappa variable 1-12 | IGKV112\|L19 | 2 | BCRSignalingPathway |
| IGKV1-13 | 28939 | immunoglobulin kappa variable 1-13 | IGKV113\|L18 | 2 | BCRSignalingPathway |
| IGKV1-16 | 28938 | immunoglobulin kappa variable 1-16 | IGKV116\|L1 | 2 | BCRSignalingPathway |
| IGKV1-17 | 28937 | immunoglobulin kappa variable 1-17 | A30\|IGKV117 | 2 | BCRSignalingPathway |
| IGKV1-27 | 28935 | immunoglobulin kappa variable 1-27 | A20\|IGKV127 | 2 | BCRSignalingPathway |
| IGKV1-33 | 28933 | immunoglobulin kappa variable 1-33 | IGKV133\|O18 | 2 | BCRSignalingPathway |
| IGKV1-37 | 28931 | immunoglobulin kappa variable 1-37 (non-functional) | IGKV137\|O14 | 2 | BCRSignalingPathway |
| IGKV1-39 | 28930 | immunoglobulin kappa variable 1-39 | IGKV139\|O12\|O12a | 2 | BCRSignalingPathway |
| IGKV1-5 | 28299 | immunoglobulin kappa variable 1-5 | IGKV\|IGKV15\|L12\|L12a\|V1 | 2 | BCRSignalingPathway |
| IGKV1-6 | 28943 | immunoglobulin kappa variable 1-6 | IGKV16\|L11 | 2 | BCRSignalingPathway |
| IGKV1-8 | 28942 | immunoglobulin kappa variable 1-8 | IGKV18\|L9 | 2 | BCRSignalingPathway |
| IGKV1-9 | 28941 | immunoglobulin kappa variable 1-9 | IGKV19\|L8 | 2 | BCRSignalingPathway |
| IGKV1D-12 | 28903 | immunoglobulin kappa variable 1D-12 | IGKV1D12\|L19 | 2 | BCRSignalingPathway |
| IGKV1D-13 | 28902 | immunoglobulin kappa variable 1D-13 | IGKV1D13\|L18 | 2 | BCRSignalingPathway |
| IGKV1D-16 | 28901 | immunoglobulin kappa variable 1D-16 | IGKV1D16\|L15\|L15a | 2 | BCRSignalingPathway |
| IGKV1D-17 | 28900 | immunoglobulin kappa variable 1D-17 | IGKV1D17\|L14 | 2 | BCRSignalingPathway |
| IGKV1D-33 | 28896 | immunoglobulin kappa variable 1D-33 | IGKV1D33\|O8 | 2 | BCRSignalingPathway |
| IGKV1D-37 | 28894 | immunoglobulin kappa variable 1D-37 (non-functional) | IGKV1D37\|O4 | 2 | BCRSignalingPathway |
| IGKV1D-39 | 28893 | immunoglobulin kappa variable 1D-39 | IGKV1D39\|O2 | 2 | BCRSignalingPathway |
| IGKV1D-42 | 28892 | immunoglobulin kappa variable 1D-42 (non-functional) | IGKV1D42\|L22 | 2 | BCRSignalingPathway |
| IGKV1D-43 | 28891 | immunoglobulin kappa variable 1D-43 | IGKV1D43\|L23\|L23a | 2 | BCRSignalingPathway |
| IGKV1D-8 | 28904 | immunoglobulin kappa variable 1D-8 | IGKV1D8\|L24\|L24a | 2 | BCRSignalingPathway |
| IGKV2-24 | 28923 | immunoglobulin kappa variable 2-24 | A23\|IGKV224 | 2 | BCRSignalingPathway |
| IGKV2-28 | 28921 | immunoglobulin kappa variable 2-28 | A19\|IGKV228 | 2 | BCRSignalingPathway |
| IGKV2-30 | 28919 | immunoglobulin kappa variable 2-30 | A17\|IGKV230 | 2 | BCRSignalingPathway |
| IGKV2-40 | 28916 | immunoglobulin kappa variable 2-40 | IGKV240\|O11\|O11a | 2 | BCRSignalingPathway |
| IGKV2D-24 | 28885 | immunoglobulin kappa variable 2D-24 (non-functional) | A7\|IGKV2D24 | 2 | BCRSignalingPathway |
| IGKV2D-28 | 28883 | immunoglobulin kappa variable 2D-28 | A3\|IGKV2D28 | 2 | BCRSignalingPathway |
| IGKV2D-29 | 28882 | immunoglobulin kappa variable 2D-29 | A2a\|A2c\|IGKV2D29 | 2 | BCRSignalingPathway |
| IGKV2D-30 | 28881 | immunoglobulin kappa variable 2D-30 | A1\|IGKV2D30 | 2 | BCRSignalingPathway |
| IGKV2D-40 | 28878 | immunoglobulin kappa variable 2D-40 | IGKV2D40\|O1 | 2 | BCRSignalingPathway |
| IGKV3-11 | 28914 | immunoglobulin kappa variable 3-11 | IGKV311\|L6 | 2 | BCRSignalingPathway |
| IGKV3-15 | 28913 | immunoglobulin kappa variable 3-15 | IGKV315\|L2 | 2 | BCRSignalingPathway |
| IGKV3-20 | 28912 | immunoglobulin kappa variable 3-20 | 13K18\|A27\|IGKV320 | 2 | BCRSignalingPathway |
| IGKV3-7 | 28915 | immunoglobulin kappa variable 3-7 (non-functional) | IGKV37\|L10\|L10a\|Vh | 2 | BCRSignalingPathway |
| IGKV3D-11 | 28876 | immunoglobulin kappa variable 3D-11 | IGKV3D11\|L20 | 2 | BCRSignalingPathway |
| IGKV3D-15 | 28875 | immunoglobulin kappa variable 3D-15 | IGKV3D15\|L16\|L16a\|L16b\|L16c | 2 | BCRSignalingPathway |
| IGKV3D-20 | 28874 | immunoglobulin kappa variable 3D-20 | A11\|A11a\|IGKV3D20 | 2 | BCRSignalingPathway |
| IGKV3D-7 | 28877 | immunoglobulin kappa variable 3D-7 | IGKV3D7\|L25 | 2 | BCRSignalingPathway |
| IGKV4-1 | 28908 | immunoglobulin kappa variable 4-1 | B3\|IGKV41 | 2 | BCRSignalingPathway |
| IGKV5-2 | 28907 | immunoglobulin kappa variable 5-2 | B2\|IGKV52 | 2 | BCRSignalingPathway |
| IGKV6-21 | 28906 | immunoglobulin kappa variable 6-21 (non-functional) | A26\|IGKV621 | 2 | BCRSignalingPathway |
| IGKV6D-21 | 28870 | immunoglobulin kappa variable 6D-21 (non-functional) | A10\|IGKV6D21 | 2 | BCRSignalingPathway |
| IGKV6D-41 | 28869 | immunoglobulin kappa variable 6D-41 (non-functional) | A14 | 2 | BCRSignalingPathway |
| IGL | 3535 | immunoglobulin lambda locus | IGL@\|IGLC6 | 22 | BCRSignalingPathway |
| IGLC1 | 3537 | immunoglobulin lambda constant 1 | IGLC | 22 | BCRSignalingPathway |
| IGLC2 | 3538 | immunoglobulin lambda constant 2 | IGLC | 22 | BCRSignalingPathway |
| IGLC3 | 3539 | immunoglobulin lambda constant 3 (Kern-Oz+ marker) | IGLC | 22 | BCRSignalingPathway |
| IGLC6 | 3542 | immunoglobulin lambda constant 6 | IGLC | 22 | BCRSignalingPathway |
| IGLC7 | 28834 | immunoglobulin lambda constant 7 | C7 | 22 | BCRSignalingPathway |
| IGLJ | 8217 | - | IGLJ@ | 22 | BCRSignalingPathway |
| IGLJ1 | 28833 | immunoglobulin lambda joining 1 | J1 | 22 | BCRSignalingPathway |
| IGLJ2 | 28832 | immunoglobulin lambda joining 2 | J2 | 22 | BCRSignalingPathway |
| IGLJ3 | 28831 | immunoglobulin lambda joining 3 | J3 | 22 | BCRSignalingPathway |
| IGLJ4 | 28830 | immunoglobulin lambda joining 4 (non-functional) | - | 22 | BCRSignalingPathway |
| IGLJ5 | 28829 | immunoglobulin lambda joining 5 (non-functional) | - | 22 | BCRSignalingPathway |
| IGLJ6 | 28828 | immunoglobulin lambda joining 6 | - | 22 | BCRSignalingPathway |
| IGLJ7 | 28827 | immunoglobulin lambda joining 7 | J7 | 22 | BCRSignalingPathway |
| IGLV@ | 3546 | - | IGLV | 22 | BCRSignalingPathway |
| IGLV1-36 | 28826 | immunoglobulin lambda variable 1-36 | IGLV136\|V1-11 | 22 | BCRSignalingPathway |
| IGLV1-40 | 28825 | immunoglobulin lambda variable 1-40 | IGLV140\|V1-13 | 22 | BCRSignalingPathway |
| IGLV1-44 | 28823 | immunoglobulin lambda variable 1-44 | IGLV144\|V1-16 | 22 | BCRSignalingPathway |
| IGLV1-47 | 28822 | immunoglobulin lambda variable 1-47 | IGLV147\|V1-17 | 22 | BCRSignalingPathway |
| IGLV1-50 | 28821 | immunoglobulin lambda variable 1-50 (non-functional) | IGLV150\|V1-18 | 22 | BCRSignalingPathway |
| IGLV1-51 | 28820 | immunoglobulin lambda variable 1-51 | IGLV151\|V1-19 | 22 | BCRSignalingPathway |
| IGLV10-54 | 28772 | immunoglobulin lambda variable 10-54 | IGLV1054\|V1-20 | 22 | BCRSignalingPathway |
| IGLV11-55 | 28770 | immunoglobulin lambda variable 11-55 (non-functional) | IGLV1155\|V4-6 | 22 | BCRSignalingPathway |
| IGLV2-11 | 28816 | immunoglobulin lambda variable 2-11 | IGLV211\|V1-3 | 22 | BCRSignalingPathway |
| IGLV2-14 | 28815 | immunoglobulin lambda variable 2-14 | IGLV214\|V1-4 | 22 | BCRSignalingPathway |
| IGLV2-18 | 28814 | immunoglobulin lambda variable 2-18 | IGLV218\|V1-5 | 22 | BCRSignalingPathway |
| IGLV2-23 | 28813 | immunoglobulin lambda variable 2-23 | IGLV223\|V1-7 | 22 | BCRSignalingPathway |
| IGLV2-33 | 28811 | immunoglobulin lambda variable 2-33 (non-functional) | IGLV233\|V1-9 | 22 | BCRSignalingPathway |
| IGLV2-8 | 28817 | immunoglobulin lambda variable 2-8 | IGLV28\|V1-2 | 22 | BCRSignalingPathway |
| IGLV3-1 | 28809 | immunoglobulin lambda variable 3-1 | IGLV31\|V2-1 | 22 | BCRSignalingPathway |
| IGLV3-10 | 28803 | immunoglobulin lambda variable 3-10 | IGLV310\|V2-7 | 22 | BCRSignalingPathway |
| IGLV3-12 | 28802 | immunoglobulin lambda variable 3-12 | IGLV312\|V2-8 | 22 | BCRSignalingPathway |
| IGLV3-16 | 28799 | immunoglobulin lambda variable 3-16 | IGLV316\|V2-11 | 22 | BCRSignalingPathway |
| IGLV3-19 | 28797 | immunoglobulin lambda variable 3-19 | IGLV319\|V2-13\|VL3L | 22 | BCRSignalingPathway |
| IGLV3-21 | 28796 | immunoglobulin lambda variable 3-21 | IGLV321\|V2-14 | 22 | BCRSignalingPathway |
| IGLV3-22 | 28795 | immunoglobulin lambda variable 3-22 | IGLV322\|V2-15 | 22 | BCRSignalingPathway |
| IGLV3-25 | 28793 | immunoglobulin lambda variable 3-25 | IGLV325\|V2-17 | 22 | BCRSignalingPathway |
| IGLV3-27 | 28791 | immunoglobulin lambda variable 3-27 | IGLV327\|V2-19 | 22 | BCRSignalingPathway |
| IGLV3-32 | 28787 | immunoglobulin lambda variable 3-32 (non-functional) | IGLV332\|V2-23P | 22 | BCRSignalingPathway |
| IGLV3-9 | 28804 | immunoglobulin lambda variable 3-9 | IGLV39\|V2-6 | 22 | BCRSignalingPathway |
| IGLV4-3 | 28786 | immunoglobulin lambda variable 4-3 | IGLV43\|V5-1 | 22 | BCRSignalingPathway |
| IGLV4-60 | 28785 | immunoglobulin lambda variable 4-60 | IGLV460\|V5-4 | 22 | BCRSignalingPathway |
| IGLV4-69 | 28784 | immunoglobulin lambda variable 4-69 | IGLV469\|V5-6 | 22 | BCRSignalingPathway |
| IGLV5-37 | 28783 | immunoglobulin lambda variable 5-37 | IGLV537\|V4-1 | 22 | BCRSignalingPathway |
| IGLV5-39 | 28782 | immunoglobulin lambda variable 5-39 | IGLV539 | 22 | BCRSignalingPathway |
| IGLV5-45 | 28781 | immunoglobulin lambda variable 5-45 | IGLV545\|V4-2 | 22 | BCRSignalingPathway |
| IGLV5-48 | 28780 | immunoglobulin lambda variable 5-48 (non-functional) | IGLV548\|V4-3 | 22 | BCRSignalingPathway |
| IGLV5-52 | 28779 | immunoglobulin lambda variable 5-52 | IGLV552\|V4-4 | 22 | BCRSignalingPathway |
| IGLV6-57 | 28778 | immunoglobulin lambda variable 6-57 | IGLV657\|V1-22 | 22 | BCRSignalingPathway |
| IGLV7-43 | 28776 | immunoglobulin lambda variable 7-43 | IGLV743\|V3-2 | 22 | BCRSignalingPathway |
| IGLV7-46 | 28775 | immunoglobulin lambda variable 7-46 | IGLV746\|V3-3 | 22 | BCRSignalingPathway |
| IGLV8-61 | 28774 | immunoglobulin lambda variable 8-61 | IGLV861\|V3-4 | 22 | BCRSignalingPathway |
| IGLV9-49 | 28773 | immunoglobulin lambda variable 9-49 | IGLV949\|V5-2 | 22 | BCRSignalingPathway |
| C3 | 718 | complement C3 | AHUS5\|ARMD9\|ASP\|C3a\|C3b\|CPAMD1\|HEL-S-62p | 19 | Chemokines |
| C5 | 727 | complement C5 | C5D\|C5a\|C5b\|CPAMD4\|ECLZB | 9 | Chemokines |
| CCL3P1 | 390788 | C-C motif chemokine ligand 3 pseudogene 1 | CCL3L2\|G0S19-3\|LD78gamma\|SCYA3L2 | 17 | Chemokines |
| CKLF | 51192 | chemokine like factor | C32\|CKLF1\|CKLF2\|CKLF3\|CKLF4\|HSPC224\|UCK-1 | 16 | Chemokines |
| CMA1 | 1215 | chymase 1 | CYH\|MCT1\|chymase | 14 | Chemokines |
| CX3CL1 | 6376 | C-X3-C motif chemokine ligand 1 | ABCD-3\|C3Xkine\|CXC3\|CXC3C\|NTN\|NTT\|SCYD1\|fractalkine\|neurotactin | 16 | Chemokines |
| CXCL17 | 284340 | C-X-C motif chemokine ligand 17 | DMC\|Dcip1\|UNQ473\|VCC-1\|VCC1 | 19 | Chemokines |
| CCN1 | 3491 | cellular communication network factor 1 | CYR61\|GIG1\|IGFBP10 | 1 | Chemokines |
| EDN1 | 1906 | endothelin 1 | ARCND3\|ET1\|HDLCQ7\|PPET1\|QME | 6 | Chemokines |
| EDN2 | 1907 | endothelin 2 | ET-2\|ET2\|PPET2 | 1 | Chemokines |
| EDN3 | 1908 | endothelin 3 | ET-3\|ET3\|HSCR4\|PPET3\|WS4B | 20 | Chemokines |
| FGF10 | 2255 | fibroblast growth factor 10 | - | 5 | Chemokines |
| LECT2 | 3950 | leukocyte cell derived chemotaxin 2 | chm-II\|chm2 | 5 | Chemokines |
| PPBPP1 | 728045 | pro-platelet basic protein pseudogene 1 | PPBPL1\|TGB2 | 4 | Chemokines |
| PROK2 | 60675 | prokineticin 2 | BV8\|HH4\|KAL4\|MIT1\|PK2 | 3 | Chemokines |
| SAA1 | 6288 | serum amyloid A1 | PIG4\|SAA\|SAA2\|TP53I4 | 11 | Chemokines |
| SAA2 | 6289 | serum amyloid A2 | SAA\|SAA1 | 11 | Chemokines |
| SBDS | 51119 | SBDS ribosome maturation factor | CGI-97\|SDS\|SWDS | 7 | Chemokines |
| SEMA3A | 10371 | semaphorin 3A | COLL1\|HH16\|Hsema-I\|Hsema-III\|SEMA1\|SEMAD\|SEMAIII\|SEMAL\|SemD\|coll-1 | 7 | Chemokines |
| SEMA3B | 7869 | semaphorin 3B | LUCA-1\|SEMA5\|SEMAA\|SemA\|semaV | 3 | Chemokines |
| SEMA3C | 10512 | semaphorin 3C | SEMAE\|SemE | 7 | Chemokines |
| SEMA3D | 223117 | semaphorin 3D | Sema-Z2\|coll-2 | 7 | Chemokines |
| SEMA3E | 9723 | semaphorin 3E | M-SEMAH\|M-SemaK\|SEMAH\|coll-5 | 7 | Chemokines |
| SEMA3F | 6405 | semaphorin 3F | SEMA-IV\|SEMA4\|SEMAK | 3 | Chemokines |
| SEMA3G | 56920 | semaphorin 3G | sem2 | 3 | Chemokines |
| SEMA4A | 64218 | semaphorin 4A | CORD10\|RP35\|SEMAB\|SEMB | 1 | Chemokines |
| SEMA4B | 10509 | semaphorin 4B | SEMAC\|SemC | 15 | Chemokines |
| SEMA4C | 54910 | semaphorin 4C | M-SEMA-F\|SEMACL1\|SEMAF\|SEMAI | 2 | Chemokines |
| SEMA4D | 10507 | semaphorin 4D | A8\|BB18\|C9orf164\|CD100\|COLL4\|GR3\|M-sema-G\|SEMAJ\|coll-4 | 9 | Chemokines |
| SEMA4F | 10505 | ssemaphorin 4F | M-SEMA\|PRO2353\|S4F\|SEMAM\|SEMAW\|m-Sema-M | 2 | Chemokines |
| SEMA4G | 57715 | semaphorin 4G | - | 10 | Chemokines |
| SEMA5A | 9037 | semaphorin 5A | SEMAF\|semF | 5 | Chemokines |
| SEMA5B | 54437 | semaphorin 5B | SEMAG\|SemG | 3 | Chemokines |
| SEMA6A | 57556 | semaphorin 6A | HT018\|SEMA\|SEMA6A1\|SEMAQ\|VIA | 5 | Chemokines |
| SEMA6B | 10501 | semaphorin 6B | EPM11\|SEM-SEMA-Y\|SEMA-VIB\|SEMAN\|semaZ | 19 | Chemokines |
| SEMA6C | 10500 | semaphorin 6C | SEMAY\|m-SemaY\|m-SemaY2 | 1 | Chemokines |
| SEMA6D | 80031 | semaphorin 6D | - | 15 | Chemokines |
| SEMA7A | 8482 | semaphorin 7A (John Milton Hagen blood group) | CD108\|CDw108\|H-SEMA-K1\|H-Sema-L\|JMH\|SEMAK1\|SEMAL | 15 | Chemokines |
| SLIT1 | 6585 | slit guidance ligand 1 | MEGF4\|SLIL1\|SLIT-1\|SLIT3 | 10 | Chemokines |
| SLIT2 | 9353 | slit guidance ligand 2 | SLIL3\|Slit-2 | 4 | Chemokines |
| TNC | 3371 | tenascin C | 150-225\|DFNA56\|GMEM\|GP\|HXB\|JI\|TN\|TN-C | 9 | Chemokines |
| TYMP | 1890 | thymidine phosphorylase | ECGF\|ECGF1\|MEDPS1\|MNGIE\|MTDPS1\|PDECGF\|TP\|hPD-ECGF | 22 | Chemokines |
| C5AR1 | 728 | complement C5a receptor 1 | C5A\|C5AR\|C5R1\|CD88 | 19 | Chemokine_Receptors |
| CCR9 | 10803 | C-C motif chemokine receptor 9 | CC-CKR-9\|CDw199\|GPR-9-6\|GPR28 | 3 | Chemokine_Receptors |
| CCRL2 | 9034 | C-C motif chemokine receptor like 2 | ACKR5\|CKRX\|CRAM\|CRAM-A\|CRAM-B\|HCR | 3 | Chemokine_Receptors |
| CMKLR1 | 1240 | chemerin chemokine-like receptor 1 | CHEMERINR\|ChemR23\|DEZ\|RVER1 | 12 | Chemokine_Receptors |
| CX3CR1 | 1524 | C-X3-C motif chemokine receptor 1 | CCRL1\|CMKBRL1\|CMKDR1\|GPR13\|GPRV28\|V28 | 3 | Chemokine_Receptors |
| CXCR3 | 2833 | C-X-C motif chemokine receptor 3 | CD182\|CD183\|CKR-L2\|CMKAR3\|GPR9\|IP10-R\|Mig-R\|MigR | X | Chemokine_Receptors |
| CXCR5 | 643 | C-X-C motif chemokine receptor 5 | BLR1\|CD185\|MDR15 | 11 | Chemokine_Receptors |
| ACKR3 | 57007 | atypical chemokine receptor 3 | CMKOR1\|CXC-R7\|CXCR-7\|CXCR7\|GPR159\|RDC-1\|RDC1 | 2 | Chemokine_Receptors |
| CYSLTR1 | 10800 | cysteinyl leukotriene receptor 1 | CYSLT1\|CYSLT1R\|CYSLTR\|HMTMF81 | X | Chemokine_Receptors |
| CYSLTR2 | 57105 | cysteinyl leukotriene receptor 2 | CYSLT2\|CYSLT2R\|GPCR21\|HG57\|HPN321\|KPG_011\|PSEC0146\|hGPCR21 | 13 | Chemokine_Receptors |
| ACKR1 | 2532 | atypical chemokine receptor 1 (Duffy blood group) | CCBP1\|CD234\|DARC\|DARC/ACKR1\|Dfy\|FY\|GPD\|GpFy\|WBCQ1 | 1 | Chemokine_Receptors |
| EDNRA | 1909 | endothelin receptor type A | ET-A\|ETA\|ETA-R\|ETAR\|ETRA\|MFDA\|hET-AR | 4 | Chemokine_Receptors |
| EDNRB | 1910 | endothelin receptor type B | ABCDS\|ET-B\|ET-BR\|ETB\|ETB1\|ETBR\|ETRB\|HSCR\|HSCR2\|WS4A | 13 | Chemokine_Receptors |
| FPR1 | 2357 | formyl peptide receptor 1 | FMLP\|FPR | 19 | Chemokine_Receptors |
| FPR2 | 2358 | formyl peptide receptor 2 | ALXR\|FMLP-R-II\|FMLPX\|FPR2A\|FPRH1\|FPRH2\|FPRL1\|HM63\|LXA4R | 19 | Chemokine_Receptors |
| GPR17 | 2840 | G protein-coupled receptor 17 | - | 2 | Chemokine_Receptors |
| GPR32 | 2854 | G protein-coupled receptor 32 | RVDR1 | 19 | Chemokine_Receptors |
| GPR33 | 2856 | G protein-coupled receptor 33 | - | 14 | Chemokine_Receptors |
| PTGDR2 | 11251 | prostaglandin D2 receptor 2 | CD294\|CRTH2\|DL1R\|DP2\|GPR44 | 11 | Chemokine_Receptors |
| C5AR2 | 27202 | complement component 5a receptor 2 | C5L2\|GPF77\|GPR77 | 19 | Chemokine_Receptors |
| CXCR2 | 3579 | C-X-C motif chemokine receptor 2 | CD182\|CDw128b\|CMKAR2\|IL8R2\|IL8RA\|IL8RB | 2 | Chemokine_Receptors |
| LTB4R2 | 56413 | leukotriene B4 receptor 2 | BLT2\|BLTR2\|JULF2\|KPG_004\|LTB4-R 2\|LTB4-R2\|NOP9 | 14 | Chemokine_Receptors |
| PLAUR | 5329 | plasminogen activator, urokinase receptor | CD87\|U-PAR\|UPAR\|URKR | 19 | Chemokine_Receptors |
| PLXNA1 | 5361 | plexin A1 | NOV\|NOVP\|PLEXIN-A1\|PLXN1 | 3 | Chemokine_Receptors |
| PLXNA2 | 5362 | plexin A2 | OCT\|PLXN2 | 1 | Chemokine_Receptors |
| PLXNA3 | 55558 | plexin A3 | 6.3\|HSSEXGENE\|PLXN3\|PLXN4\|XAP-6 | X | Chemokine_Receptors |
| PLXNA4 | 91584 | plexin A4 | FAYV2820\|PLEXA4\|PLXNA4A\|PLXNA4B\|PRO34003 | 7 | Chemokine_Receptors |
| PLXNB1 | 5364 | plexin B1 | PLEXIN-B1\|PLXN5\|SEP | 3 | Chemokine_Receptors |
| PLXNB2 | 23654 | plexin B2 | MM1\|Nbla00445\|PLEXB2\|dJ402G11.3 | 22 | Chemokine_Receptors |
| PLXNB3 | 5365 | plexin B3 | PLEXB3\|PLEXR\|PLXN6 | X | Chemokine_Receptors |
| PLXNC1 | 10154 | plexin C1 | CD232\|PLXN-C1\|VESPR | 12 | Chemokine_Receptors |
| PLXND1 | 23129 | plexin D1 | PLEXD1 | 3 | Chemokine_Receptors |
| PTAFR | 5724 | platelet activating factor receptor | PAFR | 1 | Chemokine_Receptors |
| ROBO1 | 6091 | roundabout guidance receptor 1 | DUTT1\|SAX3 | 3 | Chemokine_Receptors |
| ROBO2 | 6092 | roundabout guidance receptor 2 | SAX3 | 3 | Chemokine_Receptors |
| RXFP3 | 51289 | relaxin family peptide receptor 3 | GPCR135\|RLN3R1\|RXFPR3\|SALPR | 5 | Chemokine_Receptors |
| XCR1 | 2829 | X-C motif chemokine receptor 1 | CCXCR1\|GPR5 | 3 | Chemokine_Receptors |
| ADM | 133 | adrenomedullin | AM\|PAMP | 11 | Cytokines |
| ADM2 | 79924 | adrenomedullin 2 | AM2\|dJ579N16.4 | 22 | Cytokines |
| AGRP | 181 | agouti related neuropeptide | AGRT\|ART\|ASIP2 | 16 | Cytokines |
| AGT | 183 | angiotensinogen | ANHU\|SERPINA8\|hFLT1 | 1 | Cytokines |
| AMBN | 258 | ameloblastin | AI1F | 4 | Cytokines |
| AMELX | 265 | amelogenin X-linked | AI1E\|AIH1\|ALGN\|AMG\|AMGL\|AMGX | X | Cytokines |
| AMH | 268 | anti-Mullerian hormone | MIF\|MIS | 19 | Cytokines |
| ANGPTL5 | 253935 | angiopoietin like 5 | - | 11 | Cytokines |
| ANGPTL7 | 10218 | angiopoietin like 7 | AngX\|CDT6\|dJ647M16.1 | 1 | Cytokines |
| APLN | 8862 | apelin | APEL\|XNPEP2 | X | Cytokines |
| AREG | 374 | amphiregulin | AR\|AREGB\|CRDGF\|SDGF | 4 | Cytokines |
| MANF | 7873 | mesencephalic astrocyte derived neurotrophic factor | ARMET\|ARP | 3 | Cytokines |
| CDNF | 441549 | cerebral dopamine neurotrophic factor | ARMETL1 | 10 | Cytokines |
| ARTN | 9048 | artemin | ART\|ENOVIN\|EVN\|NBN | 1 | Cytokines |
| AVP | 551 | arginine vasopressin | ADH\|ARVP\|AVP-NPII\|AVRP\|VP | 20 | Cytokines |
| BDNF | 627 | brain derived neurotrophic factor | ANON2\|BULN2 | 11 | Cytokines |
| BMP1 | 649 | bone morphogenetic protein 1 | OI13\|PCOLC\|PCP\|PCP2\|TLD | 8 | Cytokines |
| BMP10 | 27302 | bone morphogenetic protein 10 | - | 2 | Cytokines |
| BMP15 | 9210 | bone morphogenetic protein 15 | GDF9B\|ODG2\|POF4 | X | Cytokines |
| BMP2 | 650 | bone morphogenetic protein 2 | BDA2\|BMP2A\|SSFSC | 20 | Cytokines |
| BMP3 | 651 | bone morphogenetic protein 3 | BMP-3A | 4 | Cytokines |
| BMP4 | 652 | bone morphogenetic protein 4 | BMP2B\|BMP2B1\|MCOPS6\|OFC11\|ZYME | 14 | Cytokines |
| BMP5 | 653 | bone morphogenetic protein 5 | - | 6 | Cytokines |
| BMP6 | 654 | bone morphogenetic protein 6 | VGR\|VGR1 | 6 | Cytokines |
| BMP7 | 655 | bone morphogenetic protein 7 | OP-1 | 20 | Cytokines |
| BMP8A | 353500 | bone morphogenetic protein 8a | OP-2 | 1 | Cytokines |
| BMP8B | 656 | bone morphogenetic protein 8b | BMP8\|OP2 | 1 | Cytokines |
| BTC | 685 | betacellulin | - | 4 | Cytokines |
| MYDGF | 56005 | myeloid derived growth factor | C19orf10\|EUROIMAGE1875335\|IL25\|IL27\|IL27w\|R33729_1\|SF20 | 19 | Cytokines |
| CALCA | 796 | calcitonin related polypeptide alpha | CALC1\|CGRP\|CGRP-I\|CGRP-alpha\|CGRP1\|CT\|KC\|PCT | 11 | Cytokines |
| CALCB | 797 | calcitonin related polypeptide beta | CALC2\|CGRP-II\|CGRP2 | 11 | Cytokines |
| CAT | 847 | catalase | - | 11 | Cytokines |
| CCK | 885 | cholecystokinin | - | 3 | Cytokines |
| CD320 | 51293 | CD320 molecule | 8D6\|8D6A\|TCBLR\|TCN2R | 19 | Cytokines |
| CD70 | 970 | CD70 molecule | CD27-L\|CD27L\|CD27LG\|LPFS3\|TNFSF7\|TNLG8A | 19 | Cytokines |
| ADA2 | 51816 | adenosine deaminase 2 | ADGF\|CECR1\|IDGFL\|PAN\|SNEDS\|VAIHS | 22 | Cytokines |
| CER1 | 9350 | cerberus 1, DAN family BMP antagonist | DAND4 | 9 | Cytokines |
| CGA | 1081 | glycoprotein hormones, alpha polypeptide | CG-ALPHA\|FSHA\|GPA1\|GPHA1\|GPHa\|HCG\|LHA\|TSHA | 6 | Cytokines |
| CGB3 | 1082 | chorionic gonadotropin subunit beta 3 | CGB\|CGB5\|CGB7\|CGB8\|hCGB | 19 | Cytokines |
| CGB1 | 114335 | chorionic gonadotropin subunit beta 1 | - | 19 | Cytokines |
| CGB2 | 114336 | chorionic gonadotropin subunit beta 2 | - | 19 | Cytokines |
| CGB5 | 93659 | chorionic gonadotropin subunit beta 5 | CGB\|HCG\|hCGB | 19 | Cytokines |
| CGB7 | 94027 | chorionic gonadotropin subunit beta 7 | CG-beta-a\|CGB6 | 19 | Cytokines |
| CGB8 | 94115 | chorionic gonadotropin subunit beta 8 | - | 19 | Cytokines |
| CHGA | 1113 | chromogranin A | CGA | 14 | Cytokines |
| CHGB | 1114 | chromogranin B | SCG1 | 20 | Cytokines |
| CLCF1 | 23529 | cardiotrophin like cytokine factor 1 | BSF-3\|BSF3\|CISS2\|CLC\|NNT-1\|NNT1\|NR6 | 11 | Cytokines |
| CLEC11A | 6320 | C-type lectin domain containing 11A | CLECSF3\|LSLCL\|P47\|SCGF | 19 | Cytokines |
| CMTM1 | 113540 | CKLF like MARVEL transmembrane domain containing 1 | CKLFH\|CKLFH1\|CKLFSF1 | 16 | Cytokines |
| CMTM2 | 146225 | CKLF like MARVEL transmembrane domain containing 2 | CKLFSF2 | 16 | Cytokines |
| CMTM3 | 123920 | CKLF like MARVEL transmembrane domain containing 3 | BNAS2\|CKLFSF3 | 16 | Cytokines |
| CMTM4 | 146223 | CKLF like MARVEL transmembrane domain containing 4 | CKLFSF4 | 16 | Cytokines |
| CMTM5 | 116173 | CKLF like MARVEL transmembrane domain containing 5 | CKLFSF5 | 14 | Cytokines |
| CMTM6 | 54918 | CKLF like MARVEL transmembrane domain containing 6 | CKLFSF6\|PRO2219 | 3 | Cytokines |
| CMTM7 | 112616 | CKLF like MARVEL transmembrane domain containing 7 | CKLFSF7 | 3 | Cytokines |
| CMTM8 | 152189 | CKLF like MARVEL transmembrane domain containing 8 | CKLFSF8\|CKLFSF8-V2 | 3 | Cytokines |
| CNTF | 1270 | ciliary neurotrophic factor | HCNTF | 11 | Cytokines |
| CORT | 1325 | cortistatin | CST-14\|CST-17\|CST-29 | 1 | Cytokines |
| CRH | 1392 | corticotropin releasing hormone | CRF\|CRH1 | 8 | Cytokines |
| CSF1 | 1435 | colony stimulating factor 1 | CSF-1\|MCSF | 1 | Cytokines |
| CSF2 | 1437 | colony stimulating factor 2 | CSF\|GMCSF | 5 | Cytokines |
| CSF3 | 1440 | colony stimulating factor 3 | C17orf33\|CSF3OS\|GCSF | 17 | Cytokines |
| CSH1 | 1442 | chorionic somatomammotropin hormone 1 | CS-1\|CSA\|CSMT\|GHB3\|PL\|hCS-1\|hCS-A | 17 | Cytokines |
| CSH2 | 1443 | chorionic somatomammotropin hormone 2 | CS-2\|CSB\|GHB1\|PL\|hCS-B | 17 | Cytokines |
| CSHL1 | 1444 | chorionic somatomammotropin hormone like 1 | CS-5\|CSHP1\|CSL\|GHB4\|hCS-L | 17 | Cytokines |
| CSPG5 | 10675 | chondroitin sulfate proteoglycan 5 | NGC | 3 | Cytokines |
| CTF1 | 1489 | cardiotrophin 1 | CT-1\|CT1 | 16 | Cytokines |
| CCN2 | 1490 | cellular communication network factor 2 | CTGF\|HCS24\|IGFBP8\|NOV2 | 6 | Cytokines |
| DKK1 | 22943 | dickkopf WNT signaling pathway inhibitor 1 | DKK-1\|SK | 10 | Cytokines |
| EBI3 | 10148 | Epstein-Barr virus induced 3 | IL-27B\|IL27B\|IL35B | 19 | Cytokines |
| EGF | 1950 | epidermal growth factor | HOMG4\|URG | 4 | Cytokines |
| EPGN | 255324 | epithelial mitogen | ALGV3072\|EPG\|PRO9904 | 4 | Cytokines |
| EPO | 2056 | erythropoietin | DBAL\|ECYT5\|EP\|MVCD2 | 7 | Cytokines |
| EREG | 2069 | epiregulin | EPR\|ER\|Ep | 4 | Cytokines |
| ESM1 | 11082 | endothelial cell specific molecule 1 | endocan | 5 | Cytokines |
| FAM3B | 54097 | FAM3 metabolism regulating signaling molecule B | 2-21\|C21orf11\|C21orf76\|ORF9\|PANDER\|PRED44 | 21 | Cytokines |
| FAM3C | 10447 | FAM3 metabolism regulating signaling molecule C | GS3786\|ILEI | 7 | Cytokines |
| FAM3D | 131177 | FAM3 metabolism regulating signaling molecule D | EF7\|OIT1 | 3 | Cytokines |
| FGF1 | 2246 | fibroblast growth factor 1 | AFGF\|ECGF\|ECGF-beta\|ECGFA\|ECGFB\|FGF-1\|FGF-alpha\|FGFA\|GLIO703\|HBGF-1\|HBGF1 | 5 | Cytokines |
| FGF11 | 2256 | fibroblast growth factor 11 | FGF-11\|FHF-3\|FHF3 | 17 | Cytokines |
| FGF12 | 2257 | fibroblast growth factor 12 | EIEE47\|FGF12B\|FHF1 | 3 | Cytokines |
| FGF13 | 2258 | fibroblast growth factor 13 | FGF-13\|FGF2\|FHF-2\|FHF2\|LINC00889 | X | Cytokines |
| FGF14 | 2259 | fibroblast growth factor 14 | FGF-14\|FHF-4\|FHF4\|SCA27 | 13 | Cytokines |
| FGF16 | 8823 | fibroblast growth factor 16 | FGF-16\|MF4 | X | Cytokines |
| FGF17 | 8822 | fibroblast growth factor 17 | FGF-13\|FGF-17\|HH20 | 8 | Cytokines |
| FGF18 | 8817 | fibroblast growth factor 18 | FGF-18\|ZFGF5 | 5 | Cytokines |
| FGF19 | 9965 | fibroblast growth factor 19 | - | 11 | Cytokines |
| FGF20 | 26281 | fibroblast growth factor 20 | FGF-20\|RHDA2 | 8 | Cytokines |
| FGF21 | 26291 | fibroblast growth factor 21 | - | 19 | Cytokines |
| FGF22 | 27006 | fibroblast growth factor 22 | - | 19 | Cytokines |
| FGF23 | 8074 | fibroblast growth factor 23 | ADHR\|FGFN\|HFTC2\|HPDR2\|HYPF\|PHPTC | 12 | Cytokines |
| FGF3 | 2248 | fibroblast growth factor 3 | HBGF-3\|INT2 | 11 | Cytokines |
| FGF4 | 2249 | fibroblast growth factor 4 | FGF-4\|HBGF-4\|HST\|HST-1\|HSTF-1\|HSTF1\|K-FGF\|KFGF | 11 | Cytokines |
| FGF5 | 2250 | fibroblast growth factor 5 | HBGF-5\|Smag-82\|TCMGLY | 4 | Cytokines |
| FGF6 | 2251 | fibroblast growth factor 6 | HBGF-6\|HST2 | 12 | Cytokines |
| FGF7 | 2252 | fibroblast growth factor 7 | HBGF-7\|KGF | 15 | Cytokines |
| FGF8 | 2253 | fibroblast growth factor 8 | AIGF\|FGF-8\|HBGF-8\|HH6\|KAL6 | 10 | Cytokines |
| FGF9 | 2254 | fibroblast growth factor 9 | FGF-9\|GAF\|HBFG-9\|HBGF-9\|SYNS3 | 13 | Cytokines |
| VEGFD | 2277 | vascular endothelial growth factor D | FIGF\|VEGF-D | X | Cytokines |
| FIGNL2 | 401720 | fidgetin like 2 | - | 12 | Cytokines |
| FLT3LG | 2323 | fms related receptor tyrosine kinase 3 ligand | FL\|FLG3L\|FLT3L | 19 | Cytokines |
| FSHB | 2488 | follicle stimulating hormone subunit beta | HH24 | 11 | Cytokines |
| GAL | 51083 | galanin and GMAP prepropeptide | ETL8\|GAL-GMAP\|GALN\|GLNN\|GMAP | 11 | Cytokines |
| GALP | 85569 | galanin like peptide | - | 19 | Cytokines |
| GAST | 2520 | gastrin | GAS | 17 | Cytokines |
| GCG | 2641 | glucagon | GLP-1\|GLP1\|GLP2\|GRPP | 2 | Cytokines |
| GDF1 | 2657 | growth differentiation factor 1 | CERS1\|CHTD6\|DORV\|DTGA3\|LAG1\|LASS1\|RAI\|UOG1 | 19 | Cytokines |
| GDF10 | 2662 | growth differentiation factor 10 | BIP\|BMP-3b\|BMP3B | 10 | Cytokines |
| GDF11 | 10220 | growth differentiation factor 11 | BMP-11\|BMP11 | 12 | Cytokines |
| GDF2 | 2658 | growth differentiation factor 2 | BMP-9\|BMP9\|HHT5 | 10 | Cytokines |
| GDF3 | 9573 | growth differentiation factor 3 | KFS3\|MCOP7\|MCOPCB6 | 12 | Cytokines |
| GDF5 | 8200 | growth differentiation factor 5 | BDA1C\|BMP-14\|BMP14\|CDMP1\|DUPANS\|LAP-4\|LAP4\|OS5\|SYM1B\|SYNS2 | 20 | Cytokines |
| GDF6 | 392255 | growth differentiation factor 6 | BMP-13\|BMP13\|CDMP2\|KFM\|KFS\|KFS1\|KFSL\|SGM1\|SYNS4 | 8 | Cytokines |
| GDF7 | 151449 | growth differentiation factor 7 | BMP12 | 2 | Cytokines |
| GDF9 | 2661 | growth differentiation factor 9 | POF14 | 5 | Cytokines |
| GDNF | 2668 | glial cell derived neurotrophic factor | ATF\|ATF1\|ATF2\|HFB1-GDNF\|HSCR3 | 5 | Cytokines |
| GH1 | 2688 | growth hormone 1 | GH\|GH-N\|GHB5\|GHN\|IGHD1A\|IGHD1B\|IGHD2\|hGH-N | 17 | Cytokines |
| GH2 | 2689 | growth hormone 2 | GH-V\|GHB2\|GHL\|GHV\|hGH-V | 17 | Cytokines |
| GHRH | 2691 | growth hormone releasing hormone | GHRF\|GRF\|INN | 20 | Cytokines |
| GHRL | 51738 | ghrelin and obestatin prepropeptide | MTLRP | 3 | Cytokines |
| GIP | 2695 | gastric inhibitory polypeptide | - | 17 | Cytokines |
| GKN1 | 56287 | gastrokine 1 | AMP18\|BRICD1\|CA11\|FOV\|foveolin | 2 | Cytokines |
| GMFB | 2764 | glia maturation factor beta | GMF | 14 | Cytokines |
| GMFG | 9535 | glia maturation factor gamma | GMF-GAMMA | 19 | Cytokines |
| GNRH1 | 2796 | gonadotropin releasing hormone 1 | GNRH\|GRH\|LHRH\|LNRH | 8 | Cytokines |
| GNRH2 | 2797 | gonadotropin releasing hormone 2 | GnRH-II\|LH-RHII | 20 | Cytokines |
| GPHA2 | 170589 | glycoprotein hormone subunit alpha 2 | A2\|GPA2\|ZSIG51 | 11 | Cytokines |
| GPHB5 | 122876 | glycoprotein hormone subunit beta 5 | B5\|GPB5\|ZLUT1 | 14 | Cytokines |
| GPI | 2821 | glucose-6-phosphate isomerase | AMF\|GNPI\|NLK\|PGI\|PHI\|SA-36\|SA36 | 19 | Cytokines |
| GREM1 | 26585 | gremlin 1, DAN family BMP antagonist | C15DUPq\|CKTSF1B1\|CRAC1\|CRCS4\|DAND2\|DRM\|DUP15q\|GREMLIN\|HMPS\|HMPS1\|IHG-2\|MPSH\|PIG2 | 15 | Cytokines |
| GREM2 | 64388 | gremlin 2, DAN family BMP antagonist | CKTSF1B2\|DAND3\|PRDC\|STHAG9 | 1 | Cytokines |
| GRP | 2922 | gastrin releasing peptide | BN\|GRP-10\|preproGRP\|proGRP | 18 | Cytokines |
| GUCA2A | 2980 | guanylate cyclase activator 2A | GCAP-I\|GUCA2\|STARA | 1 | Cytokines |
| HBEGF | 1839 | heparin binding EGF like growth factor | DTR\|DTS\|DTSF\|HEGFL | 5 | Cytokines |
| HDGF | 3068 | heparin binding growth factor | HMG1L2 | 1 | Cytokines |
| HDGFL3 | 50810 | HDGF like 3 | CGI-142\|HDGF-2\|HDGF2\|HDGFRP3\|HRP-3 | 15 | Cytokines |
| IAPP | 3375 | islet amyloid polypeptide | DAP\|IAP | 12 | Cytokines |
| IFNE | 338376 | interferon epsilon | IFN-E\|IFNE1\|IFNT1\|INFE1\|PRO655 | 9 | Cytokines |
| IFNK | 56832 | interferon kappa | IFNT1\|INFE1 | 9 | Cytokines |
| IFNW1 | 3467 | interferon omega 1 | - | 9 | Cytokines |
| IGF1 | 3479 | insulin like growth factor 1 | IGF\|IGF-I\|IGFI\|MGF | 12 | Cytokines |
| IGF2 | 3481 | insulin like growth factor 2 | C11orf43\|GRDF\|IGF-II\|PP9974 | 11 | Cytokines |
| IL11 | 3589 | interleukin 11 | AGIF\|IL-11 | 19 | Cytokines |
| IL12A | 3592 | interleukin 12A | CLMF\|IL-12A\|NFSK\|NKSF1\|P35 | 3 | Cytokines |
| IL16 | 3603 | interleukin 16 | LCF\|NIL16\|PRIL16\|prIL-16 | 15 | Cytokines |
| IL17B | 27190 | interleukin 17B | IL-17B\|IL-20\|NIRF\|ZCYTO7 | 5 | Cytokines |
| IL17C | 27189 | interleukin 17C | CX2\|IL-17C | 16 | Cytokines |
| IL17D | 53342 | interleukin 17D | IL-17D | 13 | Cytokines |
| IL17F | 112744 | interleukin 17F | CANDF6\|IL-17F\|ML-1\|ML1 | 6 | Cytokines |
| IL19 | 29949 | interleukin 19 | IL-10C\|MDA1\|NG.1\|ZMDA1 | 1 | Cytokines |
| IL1F10 | 84639 | interleukin 1 family member 10 | FIL1-theta\|FKSG75\|IL-1HY2\|IL-38\|IL1-theta\|IL1HY2 | 2 | Cytokines |
| IL36RN | 26525 | interleukin 36 receptor antagonist | FIL1\|FIL1(DELTA)\|FIL1D\|IL-36Ra\|IL1F5\|IL1HY1\|IL1L1\|IL1RP3\|IL36RA\|PSORP\|PSORS14 | 2 | Cytokines |
| IL36A | 27179 | interleukin 36 alpha | FIL1\|FIL1(EPSILON)\|FIL1E\|IL-1F6\|IL1(EPSILON)\|IL1F6 | 2 | Cytokines |
| IL37 | 27178 | interleukin 37 | FIL1\|FIL1(ZETA)\|FIL1Z\|IL-1F7\|IL-1H\|IL-1H4\|IL-1RP1\|IL-37\|IL1F7\|IL1H4\|IL1RP1 | 2 | Cytokines |
| IL36B | 27177 | interleukin 36 beta | FIL1\|FIL1-(ETA)\|FIL1H\|FILI-(ETA)\|IL-1F8\|IL-1H2\|IL1-ETA\|IL1F8\|IL1H2 | 2 | Cytokines |
| IL36G | 56300 | interleukin 36 gamma | IL-1F9\|IL-1H1\|IL-1RP2\|IL1E\|IL1F9\|IL1H1\|IL1RP2 | 2 | Cytokines |
| IL1RN | 3557 | interleukin 1 receptor antagonist | DIRA\|ICIL-1RA\|IL-1RN\|IL-1ra\|IL-1ra3\|IL1F3\|IL1RA\|IRAP\|MVCD4 | 2 | Cytokines |
| IL20 | 50604 | interleukin 20 | IL-20\|IL10D\|ZCYTO10 | 1 | Cytokines |
| IL21 | 59067 | interleukin 21 | CVID11\|IL-21\|Za11 | 4 | Cytokines |
| IL23A | 51561 | interleukin 23 subunit alpha | IL-23\|IL-23A\|IL23P19\|P19\|SGRF | 12 | Cytokines |
| IL24 | 11009 | interleukin 24 | C49A\|FISP\|IL10B\|MDA7\|MOB5\|ST16 | 1 | Cytokines |
| IL25 | 64806 | interleukin 25 | IL17E | 14 | Cytokines |
| IL26 | 55801 | interleukin 26 | AK155\|IL-26 | 12 | Cytokines |
| IFNL3 | 282617 | interferon lambda 3 | IFN-lambda-3\|IFN-lambda-4\|IL-28B\|IL-28C\|IL28B\|IL28C | 19 | Cytokines |
| IL3 | 3562 | interleukin 3 | IL-3\|MCGF\|MULTI-CSF | 5 | Cytokines |
| IL31 | 386653 | interleukin 31 | IL-31 | 12 | Cytokines |
| IL32 | 9235 | interleukin 32 | IL-32alpha\|IL-32beta\|IL-32delta\|IL-32gamma\|NK4\|TAIF\|TAIFa\|TAIFb\|TAIFc\|TAIFd | 16 | Cytokines |
| IL33 | 90865 | interleukin 33 | C9orf26\|DVS27\|IL1F11\|NF-HEV\|NFEHEV | 9 | Cytokines |
| IL34 | 146433 | interleukin 34 | C16orf77\|IL-34 | 16 | Cytokines |
| IL5 | 3567 | interleukin 5 | EDF\|IL-5\|TRF | 5 | Cytokines |
| IL6ST | 3572 | interleukin 6 signal transducer | CD130\|CDW130\|GP130\|HIES4\|IL-6RB\|sGP130 | 5 | Cytokines |
| IL7 | 3574 | interleukin 7 | IL-7 | 8 | Cytokines |
| IL9 | 3578 | interleukin 9 | HP40\|IL-9\|P40 | 5 | Cytokines |
| INHA | 3623 | inhibin subunit alpha | - | 2 | Cytokines |
| INHBA | 3624 | inhibin subunit beta A | EDF\|FRP | 7 | Cytokines |
| INHBB | 3625 | inhibin subunit beta B | - | 2 | Cytokines |
| INHBC | 3626 | inhibin subunit beta C | IHBC | 12 | Cytokines |
| INHBE | 83729 | inhibin subunit beta E | - | 12 | Cytokines |
| INS | 3630 | insulin | IDDM\|IDDM1\|IDDM2\|ILPR\|IRDN\|MODY10\|PNDM4 | 11 | Cytokines |
| INS-IGF2 | 723961 | INS-IGF2 readthrough | INSIGF | 11 | Cytokines |
| INSL3 | 3640 | insulin like 3 | RLF\|RLNL\|ley-I-L | 19 | Cytokines |
| INSL4 | 3641 | insulin like 4 | EPIL\|PLACENTIN | 9 | Cytokines |
| INSL5 | 10022 | insulin like 5 | PRO182\|UNQ156 | 1 | Cytokines |
| INSL6 | 11172 | insulin like 6 | RIF1 | 9 | Cytokines |
| JAG1 | 182 | jagged canonical Notch ligand 1 | AGS\|AGS1\|AHD\|AWS\|CD339\|DCHE\|HJ1\|JAGL1 | 20 | Cytokines |
| JAG2 | 3714 | jagged canonical Notch ligand 2 | HJ2\|SER2 | 14 | Cytokines |
| FGF7P6 | 387628 | fibroblast growth factor 7 pseudogene 6 | KGFLP1 | 9 | Cytokines |
| FGF7P3 | 654466 | fibroblast growth factor 7 pseudogene 3 | KGFLP2 | 9 | Cytokines |
| KITLG | 4254 | KIT ligand | DCUA\|DFNA69\|FPH2\|FPHH\|KL-1\|Kitl\|MGF\|SCF\|SF\|SHEP7\|SLF | 12 | Cytokines |
| KL | 9365 | klotho | HFTC3 | 13 | Cytokines |
| LACRT | 90070 | lacritin | - | 12 | Cytokines |
| LEFTY1 | 10637 | left-right determination factor 1 | LEFTB\|LEFTYB | 1 | Cytokines |
| LEFTY2 | 7044 | left-right determination factor 2 | EBAF\|LEFTA\|LEFTYA\|TGFB4 | 1 | Cytokines |
| LHB | 3972 | luteinizing hormone subunit beta | CGB4\|HH23\|LSH-B\|LSH-beta | 19 | Cytokines |
| LIF | 3976 | LIF interleukin 6 family cytokine | CDF\|DIA\|HILDA\|MLPLI | 22 | Cytokines |
| LRSAM1 | 90678 | leucine rich repeat and sterile alpha motif containing 1 | CMT2P\|RIFLE\|TAL | 9 | Cytokines |
| LTB | 4050 | lymphotoxin beta | TNFC\|TNFSF3\|TNLG1C\|p33 | 6 | Cytokines |
| LTBP2 | 4053 | latent transforming growth factor beta binding protein 2 | C14orf141\|GLC3D\|LTBP3\|MSPKA\|MSTP031\|WMS3 | 14 | Cytokines |
| LTBP3 | 4054 | latent transforming growth factor beta binding protein 3 | DASS\|GPHYSD3\|LTBP-3\|LTBP2\|STHAG6\|pp6425 | 11 | Cytokines |
| LTBP4 | 8425 | latent transforming growth factor beta binding protein 4 | ARCL1C\|LTBP-4\|LTBP4L\|LTBP4S | 19 | Cytokines |
| MDK | 4192 | midkine | ARAP\|MK\|NEGF2 | 11 | Cytokines |
| MIA | 8190 | MIA SH3 domain containing | CD-RAP | 19 | Cytokines |
| MLN | 4295 | motilin | - | 6 | Cytokines |
| MSTN | 2660 | myostatin | GDF8\|MSLHP | 2 | Cytokines |
| NAMPT | 10135 | nicotinamide phosphoribosyltransferase | 1110035O14Rik\|PBEF\|PBEF1\|VF\|VISFATIN | 7 | Cytokines |
| NDP | 4693 | norrin cystine knot growth factor NDP | EVR2\|FEVR\|ND | X | Cytokines |
| NENF | 29937 | neudesin neurotrophic factor | CIR2\|SCIRP10\|SPUF | 1 | Cytokines |
| NGF | 4803 | nerve growth factor | Beta-NGF\|HSAN5\|NGFB | 1 | Cytokines |
| NMB | 4828 | neuromedin B | - | 15 | Cytokines |
| NODAL | 4838 | nodal growth differentiation factor | HTX5 | 10 | Cytokines |
| CCN3 | 4856 | cellular communication network factor 3 | IBP-9\|IGFBP-9\|IGFBP9\|NOV\|NOVh | 8 | Cytokines |
| NPFF | 8620 | neuropeptide FF-amide peptide precursor | FMRFAL | 12 | Cytokines |
| NPPA | 4878 | natriuretic peptide A | ANF\|ANP\|ATFB6\|ATRST2\|CDD\|CDD-ANF\|CDP\|PND | 1 | Cytokines |
| NPPB | 4879 | natriuretic peptide B | BNP | 1 | Cytokines |
| NPPC | 4880 | natriuretic peptide C | CNP\|CNP2 | 2 | Cytokines |
| NPY | 4852 | neuropeptide Y | PYY4 | 7 | Cytokines |
| NRG1 | 3084 | neuregulin 1 | ARIA\|GGF\|GGF2\|HGL\|HRG\|HRG1\|HRGA\|MST131\|MSTP131\|NDF\|NRG1-IT2\|SMDF | 8 | Cytokines |
| NRG2 | 9542 | neuregulin 2 | DON1\|HRG2\|NTAK | 5 | Cytokines |
| NRG3 | 10718 | neuregulin 3 | HRG3\|pro-NRG3 | 10 | Cytokines |
| NRG4 | 145957 | neuregulin 4 | HRG4 | 15 | Cytokines |
| NRTN | 4902 | neurturin | NTN | 19 | Cytokines |
| NTF3 | 4908 | neurotrophin 3 | HDNF\|NGF-2\|NGF2\|NT-3\|NT3 | 12 | Cytokines |
| NTF4 | 4909 | neurotrophin 4 | GLC10\|GLC1O\|NT-4\|NT-4/5\|NT-5\|NT4\|NT5\|NTF5 | 19 | Cytokines |
| NTS | 4922 | neurotensin | NMN-125\|NN\|NT\|NT/N\|NTS1 | 12 | Cytokines |
| NUDT6 | 11162 | nudix hydrolase 6 | ASFGF2\|FGF-AS\|FGF2AS\|GFG-1\|GFG1 | 4 | Cytokines |
| OGN | 4969 | osteoglycin | OG\|OIF\|SLRR3A | 9 | Cytokines |
| OSGIN1 | 29948 | oxidative stress induced growth inhibitor 1 | BDGI\|OKL38 | 16 | Cytokines |
| OSM | 5008 | oncostatin M | - | 22 | Cytokines |
| OSTN | 344901 | osteocrin | MUSCLIN | 3 | Cytokines |
| OXT | 5020 | oxytocin/neurophysin I prepropeptide | OT\|OT-NPI\|OXT-NPI | 20 | Cytokines |
| ENDOU | 8909 | endonuclease, poly(U) specific | P11\|PP11\|PRSS26 | 12 | Cytokines |
| PDGFA | 5154 | platelet derived growth factor subunit A | PDGF-A\|PDGF1 | 7 | Cytokines |
| PDGFB | 5155 | platelet derived growth factor subunit B | IBGC5\|PDGF-2\|PDGF2\|SIS\|SSV\|c-sis | 22 | Cytokines |
| PDGFC | 56034 | platelet derived growth factor C | FALLOTEIN\|SCDGF | 4 | Cytokines |
| PDGFD | 80310 | platelet derived growth factor D | IEGF\|MSTP036\|SCDGF-B\|SCDGFB | 11 | Cytokines |
| PDGFRL | 5157 | platelet derived growth factor receptor like | PDGRL\|PRLTS | 8 | Cytokines |
| PGF | 5228 | placental growth factor | D12S1900\|PGFL\|PIGF\|PLGF\|PlGF-2\|SHGC-10760 | 14 | Cytokines |
| PMCH | 5367 | pro-melanin concentrating hormone | MCH\|ppMCH | 12 | Cytokines |
| PNOC | 5368 | prepronociceptin | N/OFQ\|NOP\|OFQ\|PPNOC\|ppN/OFQ | 8 | Cytokines |
| POMC | 5443 | proopiomelanocortin | ACTH\|CLIP\|LPH\|MSH\|NPP\|OBAIRH\|POC | 2 | Cytokines |
| PPBPP2 | 10895 | pro-platelet basic protein pseudogene 2 | PPBPL2\|SPBPBP | 4 | Cytokines |
| PPY | 5539 | pancreatic polypeptide | PNP\|PP | 17 | Cytokines |
| PRL | 5617 | prolactin | GHA1 | 6 | Cytokines |
| PRLH | 51052 | prolactin releasing hormone | PRH\|PRRP | 2 | Cytokines |
| PROK1 | 84432 | prokineticin 1 | EGVEGF\|PK1\|PRK1 | 1 | Cytokines |
| PSPN | 5623 | persephin | PSP | 19 | Cytokines |
| PTH | 5741 | parathyroid hormone | FIH1\|PTH1 | 11 | Cytokines |
| PTH2 | 113091 | parathyroid hormone 2 | TIP39 | 19 | Cytokines |
| PTHLH | 5744 | parathyroid hormone like hormone | BDE2\|HHM\|PLP\|PTHR\|PTHRP | 12 | Cytokines |
| PTN | 5764 | pleiotrophin | HARP\|HB-GAM\|HBBM\|HBGF-8\|HBGF8\|HBNF\|HBNF-1\|NEGF1\|OSF-1 | 7 | Cytokines |
| PYY | 5697 | peptide YY | PYY-I\|PYY1 | 17 | Cytokines |
| QRFP | 347148 | pyroglutamylated RFamide peptide | 26RFa\|P518 | 9 | Cytokines |
| RABEP1 | 9135 | rabaptin, RAB GTPase binding effector protein 1 | RAB5EP\|RABPT5 | 17 | Cytokines |
| RABEP2 | 79874 | rabaptin, RAB GTPase binding effector protein 2 | FRA | 16 | Cytokines |
| REG1A | 5967 | regenerating family member 1 alpha | ICRF\|P19\|PSP\|PSPS\|PSPS1\|PTP\|REG | 2 | Cytokines |
| RETN | 56729 | resistin | ADSF\|FIZZ3\|RETN1\|RSTN\|XCP1 | 19 | Cytokines |
| RETNLB | 84666 | resistin like beta | FIZZ1\|FIZZ2\|HXCP2\|RELM-beta\|RELMb\|RELMbeta\|XCP2 | 3 | Cytokines |
| RLN1 | 6013 | relaxin 1 | H1\|H1RLX\|RLXH1\|bA12D24.3.1\|bA12D24.3.2 | 9 | Cytokines |
| RLN2 | 6019 | relaxin 2 | H2\|H2-RLX\|RLXH2\|bA12D24.1.1\|bA12D24.1.2 | 9 | Cytokines |
| RLN3 | 117579 | relaxin 3 | H3\|RXN3\|ZINS4\|insl7 | 19 | Cytokines |
| SCG2 | 7857 | secretogranin II | CHGC\|EM66\|SN\|SgII | 2 | Cytokines |
| SCGB3A1 | 92304 | secretoglobin family 3A member 1 | HIN-1\|HIN1\|LU105\|PnSP-2\|UGRP2 | 5 | Cytokines |
| SCT | 6343 | secretin | - | 11 | Cytokines |
| AIMP1 | 9255 | aminoacyl tRNA synthetase complex interacting multifunctional protein 1 | EMAP2\|EMAPII\|HLD3\|SCYE1\|p43 | 4 | Cytokines |
| SECTM1 | 6398 | secreted and transmembrane 1 | K12\|SECTM | 17 | Cytokines |
| SLURP1 | 57152 | secreted LY6/PLAUR domain containing 1 | ANUP\|ARS\|ArsB\|LY6-MT\|LY6LS\|MDM | 8 | Cytokines |
| SPP1 | 6696 | secreted phosphoprotein 1 | BNSP\|BSPI\|ETA-1\|OPN | 4 | Cytokines |
| SST | 6750 | somatostatin | SMST | 3 | Cytokines |
| STC1 | 6781 | stanniocalcin 1 | STC | 8 | Cytokines |
| STC2 | 8614 | stanniocalcin 2 | STC-2\|STCRP | 5 | Cytokines |
| TAC1 | 6863 | tachykinin precursor 1 | Hs.2563\|NK2\|NKNA\|NPK\|TAC2 | 7 | Cytokines |
| TDGF1 | 6997 | teratocarcinoma-derived growth factor 1 | CR\|CR-1\|CRGF\|CRIPTO | 3 | Cytokines |
| TDGF1P3 | 6998 | teratocarcinoma-derived growth factor 1 pseudogene 3 | CR-3\|CRIPTO\|CRIPTO-3\|CRIPTO3\|TDGF1\|TDGF2\|TDGF3 | X | Cytokines |
| TG | 7038 | thyroglobulin | AITD3\|TGN | 8 | Cytokines |
| TGFA | 7039 | transforming growth factor alpha | TFGA | 2 | Cytokines |
| TGFB2 | 7042 | transforming growth factor beta 2 | G-TSF\|LDS4\|TGF-beta2 | 1 | Cytokines |
| TGFB3 | 7043 | transforming growth factor beta 3 | ARVD\|ARVD1\|LDS5\|RNHF\|TGF-beta3 | 14 | Cytokines |
| THPO | 7066 | thrombopoietin | MGDF\|MKCSF\|ML\|MPLLG\|THCYT1\|TPO | 3 | Cytokines |
| TNFRSF11B | 4982 | TNF receptor superfamily member 11b | OCIF\|OPG\|PDB5\|TR1 | 8 | Cytokines |
| TNFSF12 | 8742 | TNF superfamily member 12 | APO3L\|DR3LG\|TNLG4A\|TWEAK | 17 | Cytokines |
| TNFSF13 | 8741 | TNF superfamily member 13 | APRIL\|CD256\|TALL-2\|TALL2\|TNLG7B\|TRDL-1\|UNQ383/PRO715\|ZTNF2 | 17 | Cytokines |
| TNFSF13B | 10673 | TNF superfamily member 13b | BAFF\|BLYS\|CD257\|DTL\|TALL-1\|TALL1\|THANK\|TNFSF20\|TNLG7A\|ZTNF4 | 13 | Cytokines |
| TNFSF14 | 8740 | TNF superfamily member 14 | CD258\|HVEML\|LIGHT\|LTg | 19 | Cytokines |
| TNFSF15 | 9966 | TNF superfamily member 15 | TL1\|TL1A\|TNLG1B\|VEGI\|VEGI192A | 9 | Cytokines |
| TNFSF18 | 8995 | TNF superfamily member 18 | AITRL\|GITRL\|TL6\|TNLG2A\|hGITRL | 1 | Cytokines |
| TNFSF8 | 944 | TNF superfamily member 8 | CD153\|CD30L\|CD30LG\|TNLG3A | 9 | Cytokines |
| TNFSF9 | 8744 | TNF superfamily member 9 | 4-1BB-L\|CD137L\|TNLG5A | 19 | Cytokines |
| TOR2A | 27433 | torsin family 2 member A | TORP1 | 9 | Cytokines |
| TRH | 7200 | thyrotropin releasing hormone | Pro-TRH\|TRF | 3 | Cytokines |
| TSHB | 7252 | thyroid stimulating hormone subunit beta | TSH-B\|TSH-BETA | 1 | Cytokines |
| TSLP | 85480 | thymic stromal lymphopoietin | - | 5 | Cytokines |
| TXLNA | 200081 | taxilin alpha | IL14\|TXLN | 1 | Cytokines |
| UCN | 7349 | urocortin | UI\|UROC | 2 | Cytokines |
| UCN2 | 90226 | urocortin 2 | SRP\|UCN-II\|UCNI\|UR\|URP | 3 | Cytokines |
| UCN3 | 114131 | urocortin 3 | SCP\|SPC\|UCNIII | 10 | Cytokines |
| UTS2 | 10911 | urotensin 2 | PRO1068\|U-II\|UCN2\|UII | 1 | Cytokines |
| UTS2B | 257313 | urotensin 2B | U2B\|URP\|UTS2D | 3 | Cytokines |
| VEGFB | 7423 | vascular endothelial growth factor B | VEGFL\|VRF | 11 | Cytokines |
| VEGFC | 7424 | vascular endothelial growth factor C | Flt4-L\|LMPH1D\|LMPHM4\|VRP | 4 | Cytokines |
| VGF | 7425 | VGF nerve growth factor inducible | SCG7\|SgVII | 7 | Cytokines |
| VIP | 7432 | vasoactive intestinal peptide | PHM27 | 6 | Cytokines |
| ACVR1B | 91 | activin A receptor type 1B | ACTRIB\|ACVRLK4\|ALK4\|SKR2 | 12 | Cytokine_Receptors |
| ACVR1C | 130399 | activin A receptor type 1C | ACVRLK7\|ALK7 | 2 | Cytokine_Receptors |
| ACVR2A | 92 | activin A receptor type 2A | ACTRII\|ACVR2 | 2 | Cytokine_Receptors |
| ACVR2B | 93 | activin A receptor type 2B | ACTRIIB\|ActR-IIB\|HTX4 | 3 | Cytokine_Receptors |
| ACVRL1 | 94 | activin A receptor like type 1 | ACVRLK1\|ALK-1\|ALK1\|HHT\|HHT2\|ORW2\|SKR3\|TSR-I | 12 | Cytokine_Receptors |
| ADCYAP1R1 | 117 | ADCYAP receptor type I | PAC1\|PAC1R\|PACAPR\|PACAPRI | 7 | Cytokine_Receptors |
| ADIPOR1 | 51094 | adiponectin receptor 1 | ACDCR1\|CGI-45\|CGI45\|PAQR1\|TESBP1A | 1 | Cytokine_Receptors |
| ADIPOR2 | 79602 | adiponectin receptor 2 | ACDCR2\|PAQR2 | 12 | Cytokine_Receptors |
| ADRB1 | 153 | adrenoceptor beta 1 | ADRB1R\|B1AR\|BETA1AR\|FNSS2\|RHR | 10 | Cytokine_Receptors |
| ADRB2 | 154 | adrenoceptor beta 2 | ADRB2R\|ADRBR\|B2AR\|BAR\|BETA2AR | 5 | Cytokine_Receptors |
| AGTR1 | 185 | angiotensin II receptor type 1 | AG2S\|AGTR1B\|AT1\|AT1AR\|AT1B\|AT1BR\|AT1R\|AT2R1\|HAT1R | 3 | Cytokine_Receptors |
| AGTR2 | 186 | angiotensin II receptor type 2 | AT2\|ATGR2\|MRX88 | X | Cytokine_Receptors |
| AMHR2 | 269 | anti-Mullerian hormone receptor type 2 | AMHR\|MISR2\|MISRII\|MRII | 12 | Cytokine_Receptors |
| ANGPT1 | 284 | angiopoietin 1 | AGP1\|AGPT\|ANG1 | 8 | Cytokine_Receptors |
| ANGPT4 | 51378 | angiopoietin 4 | ANG3\|ANG4 | 20 | Cytokine_Receptors |
| ANGPTL1 | 9068 | angiopoietin like 1 | ANG3\|ANGPT3\|ARP1\|AngY\|UNQ162\|dJ595C2.2 | 1 | Cytokine_Receptors |
| ANGPTL2 | 23452 | angiopoietin like 2 | ARP2\|HARP | 9 | Cytokine_Receptors |
| ANGPTL3 | 27329 | angiopoietin like 3 | ANG-5\|ANGPT5\|ANL3\|FHBL2 | 1 | Cytokine_Receptors |
| ANGPTL4 | 51129 | angiopoietin like 4 | ARP4\|FIAF\|HARP\|HFARP\|NL2\|PGAR\|TGQTL\|UNQ171\|pp1158 | 19 | Cytokine_Receptors |
| ANGPTL6 | 83854 | angiopoietin like 6 | AGF\|ARP5 | 19 | Cytokine_Receptors |
| APLNR | 187 | apelin receptor | AGTRL1\|APJ\|APJR\|HG11 | 11 | Cytokine_Receptors |
| AR | 367 | androgen receptor | AIS\|AR8\|DHTR\|HUMARA\|HYSP1\|KD\|NR3C4\|SBMA\|SMAX1\|TFM | X | Cytokine_Receptors |
| AVPR1A | 552 | arginine vasopressin receptor 1A | AVPR V1a\|AVPR1\|V1aR | 12 | Cytokine_Receptors |
| AVPR1B | 553 | arginine vasopressin receptor 1B | AVPR3\|V1bR | 1 | Cytokine_Receptors |
| AVPR2 | 554 | arginine vasopressin receptor 2 | ADHR\|DI1\|DIR\|DIR3\|NDI\|V2R | X | Cytokine_Receptors |
| BMPR1A | 657 | bone morphogenetic protein receptor type 1A | 10q23del\|ACVRLK3\|ALK3\|CD292\|SKR5 | 10 | Cytokine_Receptors |
| BMPR1B | 658 | bone morphogenetic protein receptor type 1B | ALK-6\|ALK6\|AMDD\|BDA1D\|BDA2\|CDw293 | 4 | Cytokine_Receptors |
| BMPR2 | 659 | bone morphogenetic protein receptor type 2 | BMPR-II\|BMPR3\|BMR2\|BRK-3\|POVD1\|PPH1\|T-ALK | 2 | Cytokine_Receptors |
| BRD8 | 10902 | bromodomain containing 8 | SMAP\|SMAP2\|p120 | 5 | Cytokine_Receptors |
| C3AR1 | 719 | complement C3a receptor 1 | AZ3B\|C3AR\|HNFAG09 | 12 | Cytokine_Receptors |
| CALCR | 799 | calcitonin receptor | CRT\|CT-R\|CTR\|CTR1 | 7 | Cytokine_Receptors |
| CALCRL | 10203 | calcitonin receptor like receptor | CGRPR\|CRLR\|LMPHM8 | 2 | Cytokine_Receptors |
| CNTFR | 1271 | ciliary neurotrophic factor receptor | - | 9 | Cytokine_Receptors |
| CRHR1 | 1394 | corticotropin releasing hormone receptor 1 | CRF-R\|CRF-R-1\|CRF-R1\|CRF1\|CRFR-1\|CRFR1\|CRH-R-1\|CRH-R1\|CRHR\|CRHR1L | 17 | Cytokine_Receptors |
| CRHR2 | 1395 | corticotropin releasing hormone receptor 2 | CRF-RB\|CRF2\|CRFR2\|HM-CRF | 7 | Cytokine_Receptors |
| CRIM1 | 51232 | cysteine rich transmembrane BMP regulator 1 | CRIM-1\|S52 | 2 | Cytokine_Receptors |
| CRLF1 | 9244 | cytokine receptor like factor 1 | CISS\|CISS1\|CLF\|CLF-1\|NR6\|zcytor5 | 19 | Cytokine_Receptors |
| CRLF2 | 64109 | cytokine receptor like factor 2 | CRL2\|CRLF2Y\|TSLPR | X\|Y | Cytokine_Receptors |
| CRLF3 | 51379 | cytokine receptor like factor 3 | CREME-9\|CREME9\|CRLM9\|CYTOR4\|FRWS\|p48.2 | 17 | Cytokine_Receptors |
| CSF1R | 1436 | colony stimulating factor 1 receptor | BANDDOS\|C-FMS\|CD115\|CSF-1R\|CSFR\|FIM2\|FMS\|HDLS\|M-CSF-R | 5 | Cytokine_Receptors |
| CSF2RA | 1438 | colony stimulating factor 2 receptor subunit alpha | CD116\|CDw116\|CSF2R\|CSF2RAX\|CSF2RAY\|CSF2RX\|CSF2RY\|GM-CSF-R-alpha\|GMCSFR\|GMCSFR-alpha\|GMR\|GMR-alpha\|SMDP4\|alphaGMR | X\|Y | Cytokine_Receptors |
| CSF2RB | 1439 | colony stimulating factor 2 receptor subunit beta | CD131\|CDw131\|IL3RB\|IL5RB\|SMDP5\|betaGMR | 22 | Cytokine_Receptors |
| CSF3R | 1441 | colony stimulating factor 3 receptor | CD114\|GCSFR\|SCN7 | 1 | Cytokine_Receptors |
| EGFR | 1956 | epidermal growth factor receptor | ERBB\|ERBB1\|HER1\|NISBD2\|PIG61\|mENA | 7 | Cytokine_Receptors |
| ENG | 2022 | endoglin | END\|HHT1\|ORW1 | 9 | Cytokine_Receptors |
| EPOR | 2057 | erythropoietin receptor | EPO-R | 19 | Cytokine_Receptors |
| ESR1 | 2099 | estrogen receptor 1 | ER\|ESR\|ESRA\|ESTRR\|Era\|NR3A1 | 6 | Cytokine_Receptors |
| ESR2 | 2100 | estrogen receptor 2 | ER-BETA\|ESR-BETA\|ESRB\|ESTRB\|Erb\|NR3A2\|ODG8 | 14 | Cytokine_Receptors |
| ESRRA | 2101 | estrogen related receptor alpha | ERR1\|ERRa\|ERRalpha\|ESRL1\|NR3B1 | 11 | Cytokine_Receptors |
| ESRRB | 2103 | estrogen related receptor beta | DFNB35\|ERR beta-2\|ERR2\|ERRb\|ERRbeta2\|ESRL2\|NR3B2 | 14 | Cytokine_Receptors |
| ESRRG | 2104 | estrogen related receptor gamma | ERR-gamma\|ERR3\|ERRg\|ERRgamma\|NR3B3 | 1 | Cytokine_Receptors |
| FGFR1 | 2260 | fibroblast growth factor receptor 1 | BFGFR\|CD331\|CEK\|ECCL\|FGFBR\|FGFR-1\|FLG\|FLT-2\|FLT2\|HBGFR\|HH2\|HRTFDS\|KAL2\|N-SAM\|OGD\|bFGF-R-1 | 8 | Cytokine_Receptors |
| FGFR2 | 2263 | fibroblast growth factor receptor 2 | BBDS\|BEK\|BFR-1\|CD332\|CEK3\|CFD1\|ECT1\|JWS\|K-SAM\|KGFR\|TK14\|TK25 | 10 | Cytokine_Receptors |
| FGFR3 | 2261 | fibroblast growth factor receptor 3 | ACH\|CD333\|CEK2\|HSFGFR3EX\|JTK4 | 4 | Cytokine_Receptors |
| FGFR4 | 2264 | fibroblast growth factor receptor 4 | CD334\|JTK2\|TKF | 5 | Cytokine_Receptors |
| FGFRL1 | 53834 | fibroblast growth factor receptor like 1 | FGFR-5\|FGFR5\|FHFR | 4 | Cytokine_Receptors |
| FLT1 | 2321 | fms related receptor tyrosine kinase 1 | FLT\|FLT-1\|VEGFR-1\|VEGFR1 | 13 | Cytokine_Receptors |
| FLT3 | 2322 | fms related receptor tyrosine kinase 3 | CD135\|FLK-2\|FLK2\|STK1 | 13 | Cytokine_Receptors |
| FLT4 | 2324 | fms related receptor tyrosine kinase 4 | CHTD7\|FLT-4\|FLT41\|LMPH1A\|LMPHM1\|PCL\|VEGFR-3\|VEGFR3 | 5 | Cytokine_Receptors |
| FSHR | 2492 | follicle stimulating hormone receptor | FSHR1\|FSHRO\|LGR1\|ODG1 | 2 | Cytokine_Receptors |
| GALR2 | 8811 | galanin receptor 2 | GAL2-R\|GALNR2\|GALR-2 | 17 | Cytokine_Receptors |
| GALR3 | 8484 | galanin receptor 3 | - | 22 | Cytokine_Receptors |
| GCGR | 2642 | glucagon receptor | GGR\|GL-R | 17 | Cytokine_Receptors |
| GHR | 2690 | growth hormone receptor | GHBP\|GHIP | 5 | Cytokine_Receptors |
| GHRHR | 2692 | growth hormone releasing hormone receptor | GHRFR\|GRFR\|IGHD1B\|IGHD4 | 7 | Cytokine_Receptors |
| GHSR | 2693 | growth hormone secretagogue receptor | GHDP | 3 | Cytokine_Receptors |
| GIPR | 2696 | gastric inhibitory polypeptide receptor | PGQTL2 | 19 | Cytokine_Receptors |
| GLP1R | 2740 | glucagon like peptide 1 receptor | GLP-1\|GLP-1-R\|GLP-1R | 6 | Cytokine_Receptors |
| GLP2R | 9340 | glucagon like peptide 2 receptor | - | 17 | Cytokine_Receptors |
| GNRHR | 2798 | gonadotropin releasing hormone receptor | GNRHR1\|GRHR\|HH7\|LHRHR\|LRHR | 4 | Cytokine_Receptors |
| GPER1 | 2852 | G protein-coupled estrogen receptor 1 | CEPR\|CMKRL2\|DRY12\|FEG-1\|GPCR-Br\|GPER\|GPR30\|LERGU\|LERGU2\|LyGPR\|mER | 7 | Cytokine_Receptors |
| HNF4A | 3172 | hepatocyte nuclear factor 4 alpha | FRTS4\|HNF4\|HNF4a7\|HNF4a8\|HNF4a9\|HNF4alpha\|MODY\|MODY1\|NR2A1\|NR2A21\|TCF\|TCF14 | 20 | Cytokine_Receptors |
| HNF4G | 3174 | hepatocyte nuclear factor 4 gamma | NR2A2\|NR2A3 | 8 | Cytokine_Receptors |
| HTR3A | 3359 | 5-hydroxytryptamine receptor 3A | 5-HT-3\|5-HT3A\|5-HT3R\|5HT3R\|HTR3 | 11 | Cytokine_Receptors |
| HTR3B | 9177 | 5-hydroxytryptamine receptor 3B | 5-HT3B | 11 | Cytokine_Receptors |
| HTR3C | 170572 | 5-hydroxytryptamine receptor 3C | - | 3 | Cytokine_Receptors |
| HTR3D | 200909 | 5-hydroxytryptamine receptor 3D | 5HT3D | 3 | Cytokine_Receptors |
| HTR3E | 285242 | 5-hydroxytryptamine receptor 3E | 5-HT3-E\|5-HT3E\|5-HT3c1 | 3 | Cytokine_Receptors |
| IFNGR2 | 3460 | interferon gamma receptor 2 | AF-1\|IFGR2\|IFNGT1\|IMD28 | 21 | Cytokine_Receptors |
| IGF1R | 3480 | insulin like growth factor 1 receptor | CD221\|IGFIR\|IGFR\|JTK13 | 15 | Cytokine_Receptors |
| IGF2R | 3482 | insulin like growth factor 2 receptor | CD222\|CI-M6PR\|CIMPR\|M6P-R\|M6P/IGF2R\|MPR 300\|MPR1\|MPR300\|MPRI | 6 | Cytokine_Receptors |
| IL10RA | 3587 | interleukin 10 receptor subunit alpha | CD210\|CD210a\|CDW210A\|HIL-10R\|IL-10R1\|IL10R | 11 | Cytokine_Receptors |
| IL10RB | 3588 | interleukin 10 receptor subunit beta | CDW210B\|CRF2-4\|CRFB4\|D21S58\|D21S66\|IL-10R2 | 21 | Cytokine_Receptors |
| IL11RA | 3590 | interleukin 11 receptor subunit alpha | CRSDA | 9 | Cytokine_Receptors |
| IL12RB1 | 3594 | interleukin 12 receptor subunit beta 1 | CD212\|IL-12R-BETA1\|IL12RB\|IMD30 | 19 | Cytokine_Receptors |
| IL12RB2 | 3595 | interleukin 12 receptor subunit beta 2 | - | 1 | Cytokine_Receptors |
| IL13RA1 | 3597 | interleukin 13 receptor subunit alpha 1 | CD213A1\|CT19\|IL-13Ra\|NR4 | X | Cytokine_Receptors |
| IL13RA2 | 3598 | interleukin 13 receptor subunit alpha 2 | CD213A2\|CT19\|IL-13R\|IL13BP | X | Cytokine_Receptors |
| IL15RA | 3601 | interleukin 15 receptor subunit alpha | CD215 | 10 | Cytokine_Receptors |
| IL2RB | 3560 | interleukin 2 receptor subunit beta | CD122\|IL15RB\|IMD63\|P70-75 | 22 | Cytokine_Receptors |
| IL17RA | 23765 | interleukin 17 receptor A | CANDF5\|CD217\|CDw217\|IL-17RA\|IL17R\|IMD51\|hIL-17R | 22 | Cytokine_Receptors |
| IL17RB | 55540 | interleukin 17 receptor B | CRL4\|EVI27\|IL17BR\|IL17RH1 | 3 | Cytokine_Receptors |
| IL17RC | 84818 | interleukin 17 receptor C | CANDF9\|IL17-RL\|IL17RL | 3 | Cytokine_Receptors |
| IL17RD | 54756 | interleukin 17 receptor D | HH18\|IL-17RD\|IL17RLM\|SEF | 3 | Cytokine_Receptors |
| IL17RE | 132014 | interleukin 17 receptor E | - | 3 | Cytokine_Receptors |
| IL18R1 | 8809 | interleukin 18 receptor 1 | CD218a\|CDw218a\|IL-18R-alpha\|IL-18Ralpha\|IL-1Rrp\|IL18RA\|IL18Ralpha2\|IL1RRP | 2 | Cytokine_Receptors |
| IL18RAP | 8807 | interleukin 18 receptor accessory protein | ACPL\|CD218b\|CDw218b\|IL-18R-beta\|IL-18RAcP\|IL-18Rbeta\|IL-1R-7\|IL-1R7\|IL-1RAcPL\|IL18RB | 2 | Cytokine_Receptors |
| IL1R1 | 3554 | interleukin 1 receptor type 1 | CD121A\|D2S1473\|IL-1R-alpha\|IL1R\|IL1RA\|P80 | 2 | Cytokine_Receptors |
| IL1R2 | 7850 | interleukin 1 receptor type 2 | CD121b\|CDw121b\|IL-1R-2\|IL-1RT-2\|IL-1RT2\|IL1R2c\|IL1RB | 2 | Cytokine_Receptors |
| IL1RAP | 3556 | interleukin 1 receptor accessory protein | C3orf13\|IL-1RAcP\|IL1R3 | 3 | Cytokine_Receptors |
| IL1RL1 | 9173 | interleukin 1 receptor like 1 | DER4\|FIT-1\|IL33R\|ST2\|ST2L\|ST2V\|T1 | 2 | Cytokine_Receptors |
| IL1RL2 | 8808 | interleukin 1 receptor like 2 | IL-1Rrp2\|IL-36R\|IL1R-rp2\|IL1RRP2 | 2 | Cytokine_Receptors |
| IL20RA | 53832 | interleukin 20 receptor subunit alpha | CRF2-8\|IL-20R-alpha\|IL-20R1\|IL-20RA | 6 | Cytokine_Receptors |
| IL20RB | 53833 | interleukin 20 receptor subunit beta | DIRS1\|FNDC6\|IL-20R2 | 3 | Cytokine_Receptors |
| IL21R | 50615 | interleukin 21 receptor | CD360\|IMD56\|NILR | 16 | Cytokine_Receptors |
| IL22RA1 | 58985 | interleukin 22 receptor subunit alpha 1 | CRF2-9\|IL22R\|IL22R1 | 1 | Cytokine_Receptors |
| IL22RA2 | 116379 | interleukin 22 receptor subunit alpha 2 | CRF2-10\|CRF2-S1\|CRF2X\|IL-22BP\|IL-22R-alpha-2\|IL-22RA2\|ZCYTOR16 | 6 | Cytokine_Receptors |
| IL23R | 149233 | interleukin 23 receptor | - | 1 | Cytokine_Receptors |
| IL27RA | 9466 | interleukin 27 receptor subunit alpha | CRL1\|IL-27RA\|IL27R\|TCCR\|WSX1\|zcytor1 | 19 | Cytokine_Receptors |
| IL2RA | 3559 | interleukin 2 receptor subunit alpha | CD25\|IDDM10\|IL2R\|IMD41\|TCGFR\|p55 | 10 | Cytokine_Receptors |
| IL2RG | 3561 | interleukin 2 receptor subunit gamma | CD132\|CIDX\|IL-2RG\|IMD4\|P64\|SCIDX\|SCIDX1 | X | Cytokine_Receptors |
| IL31RA | 133396 | interleukin 31 receptor A | CRL\|CRL3\|GLM-R\|GLMR\|GPL\|IL-31RA\|PLCA2\|PRO21384\|hGLM-R | 5 | Cytokine_Receptors |
| IL3RA | 3563 | interleukin 3 receptor subunit alpha | CD123\|IL3R\|IL3RAY\|IL3RX\|IL3RY\|hIL-3Ra | X\|Y | Cytokine_Receptors |
| IL4R | 3566 | interleukin 4 receptor | CD124\|IL-4RA\|IL4RA | 16 | Cytokine_Receptors |
| IL5RA | 3568 | interleukin 5 receptor subunit alpha | CD125\|CDw125\|HSIL5R3\|IL5R | 3 | Cytokine_Receptors |
| IL6R | 3570 | interleukin 6 receptor | CD126\|IL-6R-1\|IL-6RA\|IL6Q\|IL6RA\|IL6RQ\|gp80 | 1 | Cytokine_Receptors |
| IL9R | 3581 | interleukin 9 receptor | CD129\|IL-9R | X\|Y | Cytokine_Receptors |
| INSR | 3643 | insulin receptor | CD220\|HHF5 | 19 | Cytokine_Receptors |
| KDR | 3791 | kinase insert domain receptor | CD309\|FLK1\|VEGFR\|VEGFR2 | 4 | Cytokine_Receptors |
| LEPR | 3953 | leptin receptor | CD295\|LEP-R\|LEPRD\|OB-R\|OBR | 1 | Cytokine_Receptors |
| LGR4 | 55366 | leucine rich repeat containing G protein-coupled receptor 4 | BNMD17\|GPR48 | 11 | Cytokine_Receptors |
| LGR5 | 8549 | leucine rich repeat containing G protein-coupled receptor 5 | FEX\|GPR49\|GPR67\|GRP49\|HG38 | 12 | Cytokine_Receptors |
| LGR6 | 59352 | leucine rich repeat containing G protein-coupled receptor 6 | GPCR\|VTS20631 | 1 | Cytokine_Receptors |
| LHCGR | 3973 | luteinizing hormone/choriogonadotropin receptor | HHG\|LCGR\|LGR2\|LH/CG-R\|LH/CGR\|LHR\|LHRHR\|LSH-R\|ULG5 | 2 | Cytokine_Receptors |
| LIFR | 3977 | LIF receptor subunit alpha | CD118\|LIF-R\|SJS2\|STWS\|SWS | 5 | Cytokine_Receptors |
| LTBR | 4055 | lymphotoxin beta receptor | D12S370\|LT-BETA-R\|TNF-R-III\|TNFCR\|TNFR-RP\|TNFR2-RP\|TNFR3\|TNFRSF3 | 12 | Cytokine_Receptors |
| MC1R | 4157 | melanocortin 1 receptor | CMM5\|MSH-R\|SHEP2 | 16 | Cytokine_Receptors |
| MC2R | 4158 | melanocortin 2 receptor | ACTHR | 18 | Cytokine_Receptors |
| MC3R | 4159 | melanocortin 3 receptor | BMIQ9\|MC3\|MC3-R\|OB20\|OQTL | 20 | Cytokine_Receptors |
| MC4R | 4160 | melanocortin 4 receptor | BMIQ20 | 18 | Cytokine_Receptors |
| MCHR1 | 2847 | melanin concentrating hormone receptor 1 | GPR24\|MCH-1R\|MCH1R\|SLC-1\|SLC1 | 22 | Cytokine_Receptors |
| MCHR2 | 84539 | melanin concentrating hormone receptor 2 | GPR145\|GPRv17\|MCH-2R\|MCH-R2\|MCH2\|MCH2R\|MCHR-2\|SLT | 6 | Cytokine_Receptors |
| MET | 4233 | MET proto-oncogene, receptor tyrosine kinase | AUTS9\|DFNB97\|HGFR\|RCCP2\|c-Met | 7 | Cytokine_Receptors |
| MLNR | 2862 | motilin receptor | GPR38\|MTLR1 | 13 | Cytokine_Receptors |
| MPL | 4352 | MPL proto-oncogene, thrombopoietin receptor | C-MPL\|CD110\|MPLV\|THCYT2\|THPOR\|TPOR | 1 | Cytokine_Receptors |
| MTNR1A | 4543 | melatonin receptor 1A | MEL-1A-R\|MT1 | 4 | Cytokine_Receptors |
| MTNR1B | 4544 | melatonin receptor 1B | FGQTL2\|MEL-1B-R\|MT2 | 11 | Cytokine_Receptors |
| NGFR | 4804 | nerve growth factor receptor | CD271\|Gp80-LNGFR\|TNFRSF16\|p75(NTR)\|p75NTR | 17 | Cytokine_Receptors |
| NMBR | 4829 | neuromedin B receptor | BB1\|BB1R\|NMB-R | 6 | Cytokine_Receptors |
| NPR1 | 4881 | natriuretic peptide receptor 1 | ANPRA\|ANPa\|GUC2A\|GUCY2A\|NPRA | 1 | Cytokine_Receptors |
| NPR3 | 4883 | natriuretic peptide receptor 3 | ANP-C\|ANPR-C\|ANPRC\|C5orf23\|GUCY2B\|NPR-C\|NPRC | 5 | Cytokine_Receptors |
| NR0B1 | 190 | nuclear receptor subfamily 0 group B member 1 | AHC\|AHCH\|AHX\|DAX-1\|DAX1\|DSS\|GTD\|HHG\|NROB1\|SRXY2 | X | Cytokine_Receptors |
| NR0B2 | 8431 | nuclear receptor subfamily 0 group B member 2 | SHP\|SHP1 | 1 | Cytokine_Receptors |
| NR1D1 | 9572 | nuclear receptor subfamily 1 group D member 1 | EAR1\|REVERBA\|REVERBalpha\|THRA1\|THRAL\|ear-1\|hRev | 17 | Cytokine_Receptors |
| NR1D2 | 9975 | nuclear receptor subfamily 1 group D member 2 | BD73\|EAR-1R\|REVERBB\|REVERBbeta\|RVR | 3 | Cytokine_Receptors |
| NR1H2 | 7376 | nuclear receptor subfamily 1 group H member 2 | LXR-b\|LXRB\|NER\|NER-I\|RIP15\|UNR | 19 | Cytokine_Receptors |
| NR1H3 | 10062 | nuclear receptor subfamily 1 group H member 3 | LXR-a\|LXRA\|RLD-1 | 11 | Cytokine_Receptors |
| NR1H4 | 9971 | nuclear receptor subfamily 1 group H member 4 | BAR\|FXR\|HRR-1\|HRR1\|PFIC5\|RIP14 | 12 | Cytokine_Receptors |
| NR1I2 | 8856 | nuclear receptor subfamily 1 group I member 2 | BXR\|ONR1\|PAR\|PAR1\|PAR2\|PARq\|PRR\|PXR\|SAR\|SXR | 3 | Cytokine_Receptors |
| NR1I3 | 9970 | nuclear receptor subfamily 1 group I member 3 | CAR\|CAR1\|MB67 | 1 | Cytokine_Receptors |
| NR2C1 | 7181 | nuclear receptor subfamily 2 group C member 1 | TR2 | 12 | Cytokine_Receptors |
| NR2C2 | 7182 | nuclear receptor subfamily 2 group C member 2 | TAK1\|TR4 | 3 | Cytokine_Receptors |
| NR2E1 | 7101 | nuclear receptor subfamily 2 group E member 1 | TLL\|TLX\|XTLL | 6 | Cytokine_Receptors |
| NR2E3 | 10002 | nuclear receptor subfamily 2 group E member 3 | ESCS\|PNR\|RNR\|RP37\|rd7 | 15 | Cytokine_Receptors |
| NR2F1 | 7025 | nuclear receptor subfamily 2 group F member 1 | BBOAS\|BBSOAS\|COUP-TFI\|COUPTF1\|EAR-3\|EAR3\|ERBAL3\|SVP44\|TCFCOUP1\|TFCOUP1 | 5 | Cytokine_Receptors |
| NR2F2 | 7026 | nuclear receptor subfamily 2 group F member 2 | ARP-1\|ARP1\|CHTD4\|COUPTF2\|COUPTFB\|COUPTFII\|NF-E3\|SVP40\|TFCOUP2 | 15 | Cytokine_Receptors |
| NR2F6 | 2063 | nuclear receptor subfamily 2 group F member 6 | EAR-2\|EAR2\|ERBAL2 | 19 | Cytokine_Receptors |
| NR3C1 | 2908 | nuclear receptor subfamily 3 group C member 1 | GCCR\|GCR\|GCRST\|GR\|GRL | 5 | Cytokine_Receptors |
| NR3C2 | 4306 | nuclear receptor subfamily 3 group C member 2 | MCR\|MLR\|MR\|NR3C2VIT | 4 | Cytokine_Receptors |
| NR4A1 | 3164 | nuclear receptor subfamily 4 group A member 1 | GFRP1\|HMR\|N10\|NAK-1\|NGFIB\|NP10\|NUR77\|TR3 | 12 | Cytokine_Receptors |
| NR4A2 | 4929 | nuclear receptor subfamily 4 group A member 2 | HZF-3\|NOT\|NURR1\|RNR1\|TINUR | 2 | Cytokine_Receptors |
| NR4A3 | 8013 | nuclear receptor subfamily 4 group A member 3 | CHN\|CSMF\|MINOR\|NOR1\|TEC | 9 | Cytokine_Receptors |
| NR5A1 | 2516 | nuclear receptor subfamily 5 group A member 1 | AD4BP\|ELP\|FTZ1\|FTZF1\|POF7\|SF-1\|SF1\|SPGF8\|SRXX4\|SRXY3\|hSF-1 | 9 | Cytokine_Receptors |
| NR5A2 | 2494 | nuclear receptor subfamily 5 group A member 2 | B1F\|B1F2\|CPF\|FTF\|FTZ-F1\|FTZ-F1beta\|LRH-1\|LRH1\|hB1F-2 | 1 | Cytokine_Receptors |
| NR6A1 | 2649 | nuclear receptor subfamily 6 group A member 1 | CT150\|GCNF\|GCNF1\|NR61\|RTR\|hGCNF\|hRTR | 9 | Cytokine_Receptors |
| NRP1 | 8829 | neuropilin 1 | BDCA4\|CD304\|NP1\|NRP\|VEGF165R | 10 | Cytokine_Receptors |
| NRP2 | 8828 | neuropilin 2 | NP2\|NPN2\|PRO2714\|VEGF165R2 | 2 | Cytokine_Receptors |
| OGFR | 11054 | opioid growth factor receptor | - | 20 | Cytokine_Receptors |
| OPRD1 | 4985 | opioid receptor delta 1 | DOP\|DOR\|DOR1\|OPRD | 1 | Cytokine_Receptors |
| OPRK1 | 4986 | opioid receptor kappa 1 | K-OR-1\|KOP\|KOR\|KOR-1\|KOR1\|OPRK | 8 | Cytokine_Receptors |
| OPRL1 | 4987 | opioid related nociceptin receptor 1 | KOR-3\|KOR3\|NOCIR\|NOP\|NOPr\|OOR\|OPRL\|ORL1 | 20 | Cytokine_Receptors |
| OPRM1 | 4988 | opioid receptor mu 1 | LMOR\|M-OR-1\|MOP\|MOR\|MOR1\|OPRM | 6 | Cytokine_Receptors |
| OSMR | 9180 | oncostatin M receptor | IL-31R-beta\|IL-31RB\|OSMRB\|OSMRbeta\|PLCA1 | 5 | Cytokine_Receptors |
| OXTR | 5021 | oxytocin receptor | OT-R | 3 | Cytokine_Receptors |
| PGR | 5241 | progesterone receptor | NR3C3\|PR | 11 | Cytokine_Receptors |
| PGRMC2 | 10424 | progesterone receptor membrane component 2 | DG6\|PMBP | 4 | Cytokine_Receptors |
| PPARA | 5465 | peroxisome proliferator activated receptor alpha | NR1C1\|PPAR\|PPARalpha\|hPPAR | 22 | Cytokine_Receptors |
| PPARD | 5467 | peroxisome proliferator activated receptor delta | FAAR\|NR1C2\|NUC1\|NUCI\|NUCII\|PPARB | 6 | Cytokine_Receptors |
| PRLHR | 2834 | prolactin releasing hormone receptor | GPR10\|GR3\|PrRPR | 10 | Cytokine_Receptors |
| PRLR | 5618 | prolactin receptor | HPRL\|MFAB\|RI-PRLR\|hPRLrI | 5 | Cytokine_Receptors |
| PTGER1 | 5731 | prostaglandin E receptor 1 | EP1 | 19 | Cytokine_Receptors |
| PTGER2 | 5732 | prostaglandin E receptor 2 | EP2 | 14 | Cytokine_Receptors |
| PTGER3 | 5733 | prostaglandin E receptor 3 | EP3\|EP3-I\|EP3-II\|EP3-III\|EP3-IV\|EP3-VI\|EP3e\|PGE2-R\|lnc003875 | 1 | Cytokine_Receptors |
| PTGER4 | 5734 | prostaglandin E receptor 4 | EP4\|EP4R | 5 | Cytokine_Receptors |
| PTGFR | 5737 | prostaglandin F receptor | FP | 1 | Cytokine_Receptors |
| PTH1R | 5745 | parathyroid hormone 1 receptor | EKNS\|PFE\|PTHR\|PTHR1 | 3 | Cytokine_Receptors |
| PTH2R | 5746 | parathyroid hormone 2 receptor | PTHR2 | 2 | Cytokine_Receptors |
| RARA | 5914 | retinoic acid receptor alpha | NR1B1\|RAR | 17 | Cytokine_Receptors |
| RARB | 5915 | retinoic acid receptor beta | HAP\|MCOPS12\|NR1B2\|RARbeta1\|RRB2 | 3 | Cytokine_Receptors |
| RARG | 5916 | retinoic acid receptor gamma | NR1B3\|RARC | 12 | Cytokine_Receptors |
| RORA | 6095 | RAR related orphan receptor A | IDDECA\|NR1F1\|ROR1\|ROR2\|ROR3\|RZR-ALPHA\|RZRA | 15 | Cytokine_Receptors |
| RORB | 6096 | RAR related orphan receptor B | EIG15\|NR1F2\|ROR-BETA\|RZR-BETA\|RZRB\|bA133M9.1 | 9 | Cytokine_Receptors |
| RORC | 6097 | RAR related orphan receptor C | IMD42\|NR1F3\|RORG\|RZR-GAMMA\|RZRG\|TOR | 1 | Cytokine_Receptors |
| RXFP1 | 59350 | relaxin family peptide receptor 1 | LGR7\|RXFPR1 | 4 | Cytokine_Receptors |
| RXFP2 | 122042 | relaxin family peptide receptor 2 | GPR106\|GREAT\|INSL3R\|LGR8\|LGR8.1\|RXFPR2 | 13 | Cytokine_Receptors |
| RXRA | 6256 | retinoid X receptor alpha | NR2B1 | 9 | Cytokine_Receptors |
| RXRB | 6257 | retinoid X receptor beta | DAUDI6\|H-2RIIBP\|NR2B2\|RCoR-1 | 6 | Cytokine_Receptors |
| RXRG | 6258 | retinoid X receptor gamma | NR2B3\|RXRC | 1 | Cytokine_Receptors |
| S1PR1 | 1901 | sphingosine-1-phosphate receptor 1 | CD363\|CHEDG1\|D1S3362\|ECGF1\|EDG-1\|EDG1\|S1P1 | 1 | Cytokine_Receptors |
| S1PR2 | 9294 | sphingosine-1-phosphate receptor 2 | AGR16\|DFNB68\|EDG-5\|EDG5\|Gpcr13\|H218\|LPB2\|S1P2 | 19 | Cytokine_Receptors |
| SCTR | 6344 | secretin receptor | SR | 2 | Cytokine_Receptors |
| SDC1 | 6382 | syndecan 1 | CD138\|SDC\|SYND1\|syndecan | 2 | Cytokine_Receptors |
| SDC2 | 6383 | syndecan 2 | CD362\|HSPG\|HSPG1\|SYND2 | 8 | Cytokine_Receptors |
| SDC3 | 9672 | syndecan 3 | SDCN\|SYND3 | 1 | Cytokine_Receptors |
| SDC4 | 6385 | syndecan 4 | SYND4 | 20 | Cytokine_Receptors |
| SORT1 | 6272 | sortilin 1 | Gp95\|LDLCQ6\|NT3\|NTR3 | 1 | Cytokine_Receptors |
| SSTR1 | 6751 | somatostatin receptor 1 | SRIF-2\|SS-1-R\|SS1-R\|SS1R | 14 | Cytokine_Receptors |
| SSTR2 | 6752 | somatostatin receptor 2 | - | 17 | Cytokine_Receptors |
| SSTR5 | 6755 | somatostatin receptor 5 | SS-5-R | 16 | Cytokine_Receptors |
| ST2 | 6761 | - | - | 11 | Cytokine_Receptors |
| TACR1 | 6869 | tachykinin receptor 1 | NK1R\|NKIR\|SPR\|TAC1R | 2 | Cytokine_Receptors |
| TEK | 7010 | TEK receptor tyrosine kinase | CD202B\|GLC3E\|TIE-2\|TIE2\|VMCM\|VMCM1 | 9 | Cytokine_Receptors |
| TGFBR1 | 7046 | transforming growth factor beta receptor 1 | AAT5\|ACVRLK4\|ALK-5\|ALK5\|ESS1\|LDS1\|LDS1A\|LDS2A\|MSSE\|SKR4\|TBR-i\|TBRI\|TGFR-1\|tbetaR-I | 9 | Cytokine_Receptors |
| TGFBR2 | 7048 | transforming growth factor beta receptor 2 | AAT3\|FAA3\|LDS1B\|LDS2\|LDS2B\|MFS2\|RIIC\|TAAD2\|TBR-ii\|TBRII\|TGFR-2\|TGFbeta-RII | 3 | Cytokine_Receptors |
| TGFBR3 | 7049 | transforming growth factor beta receptor 3 | BGCAN\|betaglycan | 1 | Cytokine_Receptors |
| THRA | 7067 | thyroid hormone receptor alpha | AR7\|CHNG6\|EAR7\|ERB-T-1\|ERBA\|ERBA1\|NR1A1\|THRA1\|THRA2\|c-ERBA-1 | 17 | Cytokine_Receptors |
| THRB | 7068 | thyroid hormone receptor beta | C-ERBA-2\|C-ERBA-BETA\|ERBA2\|GRTH\|NR1A2\|PRTH\|THR1\|THRB1\|THRB2 | 3 | Cytokine_Receptors |
| TIE1 | 7075 | tyrosine kinase with immunoglobulin like and EGF like domains 1 | JTK14\|TIE | 1 | Cytokine_Receptors |
| TNFRSF10C | 8794 | TNF receptor superfamily member 10c | CD263\|DCR1\|DCR1-TNFR\|LIT\|TRAIL-R3\|TRAILR3\|TRID | 8 | Cytokine_Receptors |
| TNFRSF10D | 8793 | TNF receptor superfamily member 10d | CD264\|DCR2\|TRAIL-R4\|TRAILR4\|TRUNDD | 8 | Cytokine_Receptors |
| TNFRSF11A | 8792 | TNF receptor superfamily member 11a | CD265\|FEO\|LOH18CR1\|ODFR\|OFE\|OPTB7\|OSTS\|PDB2\|RANK\|TRANCER | 18 | Cytokine_Receptors |
| TNFRSF12A | 51330 | TNF receptor superfamily member 12A | CD266\|FN14\|TWEAKR | 16 | Cytokine_Receptors |
| TNFRSF13B | 23495 | TNF receptor superfamily member 13B | CD267\|CVID\|CVID2\|IGAD2\|RYZN\|TACI\|TNFRSF14B | 17 | Cytokine_Receptors |
| TNFRSF13C | 115650 | TNF receptor superfamily member 13C | BAFF-R\|BAFFR\|BROMIX\|CD268\|CVID4\|prolixin | 22 | Cytokine_Receptors |
| TNFRSF14 | 8764 | TNF receptor superfamily member 14 | ATAR\|CD270\|HVEA\|HVEM\|LIGHTR\|TR2 | 1 | Cytokine_Receptors |
| TNFRSF17 | 608 | TNF receptor superfamily member 17 | BCM\|BCMA\|CD269\|TNFRSF13A | 16 | Cytokine_Receptors |
| TNFRSF18 | 8784 | TNF receptor superfamily member 18 | AITR\|CD357\|ENERGEN\|GITR\|GITR-D | 1 | Cytokine_Receptors |
| TNFRSF19 | 55504 | TNF receptor superfamily member 19 | TAJ\|TAJ-alpha\|TRADE\|TROY | 13 | Cytokine_Receptors |
| TNFRSF1A | 7132 | TNF receptor superfamily member 1A | CD120a\|FPF\|TBP1\|TNF-R\|TNF-R-I\|TNF-R55\|TNFAR\|TNFR1\|TNFR55\|TNFR60\|p55\|p55-R\|p60 | 12 | Cytokine_Receptors |
| TNFRSF1B | 7133 | TNF receptor superfamily member 1B | CD120b\|TBPII\|TNF-R-II\|TNF-R75\|TNFBR\|TNFR1B\|TNFR2\|TNFR80\|p75\|p75TNFR | 1 | Cytokine_Receptors |
| TNFRSF21 | 27242 | TNF receptor superfamily member 21 | BM-018\|CD358\|DR6 | 6 | Cytokine_Receptors |
| TNFRSF25 | 8718 | TNF receptor superfamily member 25 | APO-3\|DDR3\|DR3\|GEF720\|LARD\|PLEKHG5\|TNFRSF12\|TR3\|TRAMP\|WSL-1\|WSL-LR | 1 | Cytokine_Receptors |
| TNFRSF4 | 7293 | TNF receptor superfamily member 4 | ACT35\|CD134\|IMD16\|OX40\|TXGP1L | 1 | Cytokine_Receptors |
| TNFRSF6B | 8771 | TNF receptor superfamily member 6b | DCR3\|DJ583P15.1.1\|M68\|M68E\|TR6 | 20 | Cytokine_Receptors |
| TNFRSF8 | 943 | TNF receptor superfamily member 8 | CD30\|D1S166E\|Ki-1 | 1 | Cytokine_Receptors |
| TNFRSF9 | 3604 | TNF receptor superfamily member 9 | 4-1BB\|CD137\|CDw137\|ILA | 1 | Cytokine_Receptors |
| TRHR | 7201 | thyrotropin releasing hormone receptor | CHNG7\|TRH-R | 8 | Cytokine_Receptors |
| TSHR | 7253 | thyroid stimulating hormone receptor | CHNG1\|LGR3\|hTSHR-I | 14 | Cytokine_Receptors |
| TUBB3 | 10381 | tubulin beta 3 class III | CDCBM\|CDCBM1\|CFEOM3\|CFEOM3A\|FEOM3\|TUBB4\|beta-4 | 16 | Cytokine_Receptors |
| VIPR1 | 7433 | vasoactive intestinal peptide receptor 1 | HVR1\|II\|PACAP-R-2\|PACAP-R2\|RDC1\|V1RG\|VAPC1\|VIP-R-1\|VIPR\|VIRG\|VPAC1\|VPAC1R\|VPCAP1R | 3 | Cytokine_Receptors |
| VIPR2 | 7434 | vasoactive intestinal peptide receptor 2 | C16DUPq36.3\|DUP7q36.3\|PACAP-R-3\|PACAP-R3\|VIP-R-2\|VPAC2\|VPAC2R\|VPCAP2R | 7 | Cytokine_Receptors |
| PTPN11 | 5781 | protein tyrosine phosphatase non-receptor type 11 | BPTP3\|CFC\|JMML\|METCDS\|NS1\|PTP-1D\|PTP2C\|SH-PTP2\|SH-PTP3\|SHP2 | 12 | NaturalKiller_Cell_Cytotoxicity |
| ICAM2 | 3384 | intercellular adhesion molecule 2 | CD102 | 17 | NaturalKiller_Cell_Cytotoxicity |
| ITGAL | 3683 | integrin subunit alpha L | CD11A\|LFA-1\|LFA1A | 16 | NaturalKiller_Cell_Cytotoxicity |
| ITGB2 | 3689 | integrin subunit beta 2 | CD18\|LAD\|LCAMB\|LFA-1\|MAC-1\|MF17\|MFI7 | 21 | NaturalKiller_Cell_Cytotoxicity |
| PAK1 | 5058 | p21 (RAC1) activated kinase 1 | IDDMSSD\|PAKalpha\|alpha-PAK\|p65-PAK | 11 | NaturalKiller_Cell_Cytotoxicity |
| NCR2 | 9436 | natural cytotoxicity triggering receptor 2 | CD336\|LY95\|NK-p44\|NKP44\|dJ149M18.1 | 6 | NaturalKiller_Cell_Cytotoxicity |
| TYROBP | 7305 | transmembrane immune signaling adaptor TYROBP | DAP12\|KARAP\|PLOSL\|PLOSL1 | 19 | NaturalKiller_Cell_Cytotoxicity |
| LCK | 3932 | LCK proto-oncogene, Src family tyrosine kinase | IMD22\|LSK\|YT16\|p56lck\|pp58lck | 1 | NaturalKiller_Cell_Cytotoxicity |
| FCGR3A | 2214 | Fc fragment of IgG receptor IIIa | CD16\|CD16A\|FCG3\|FCGR3\|FCGRIII\|FCR-10\|FCRIII\|FCRIIIA\|IGFR3\|IMD20 | 1 | NaturalKiller_Cell_Cytotoxicity |
| FCGR3B | 2215 | Fc fragment of IgG receptor IIIb | CD16\|CD16A\|CD16b\|FCG3\|FCGR3\|FCGR3A\|FCR-10\|FCRIII\|FCRIIIb | 1 | NaturalKiller_Cell_Cytotoxicity |
| NCR1 | 9437 | natural cytotoxicity triggering receptor 1 | CD335\|LY94\|NK-p46\|NKP46 | 19 | NaturalKiller_Cell_Cytotoxicity |
| NCR3 | 259197 | natural cytotoxicity triggering receptor 3 | 1C7\|CD337\|LY117\|MALS\|NKp30 | 6 | NaturalKiller_Cell_Cytotoxicity |
| CD247 | 919 | CD247 molecule | CD3-ZETA\|CD3H\|CD3Q\|CD3Z\|IMD25\|T3Z\|TCRZ | 1 | NaturalKiller_Cell_Cytotoxicity |
| ZAP70 | 7535 | zeta chain of T cell receptor associated protein kinase 70 | ADMIO2\|IMD48\|SRK\|STCD\|STD\|TZK\|ZAP-70 | 2 | NaturalKiller_Cell_Cytotoxicity |
| LCP2 | 3937 | lymphocyte cytosolic protein 2 | SLP-76\|SLP76 | 5 | NaturalKiller_Cell_Cytotoxicity |
| LAT | 27040 | linker for activation of T cells | IMD52\|LAT1\|pp36 | 16 | NaturalKiller_Cell_Cytotoxicity |
| PLCG1 | 5335 | phospholipase C gamma 1 | NCKAP3\|PLC-II\|PLC1\|PLC148\|PLCgamma1 | 20 | NaturalKiller_Cell_Cytotoxicity |
| SH3BP2 | 6452 | SH3 domain binding protein 2 | 3BP-2\|3BP2\|CRBM\|CRPM\|RES4-23 | 4 | NaturalKiller_Cell_Cytotoxicity |
| FYN | 2534 | FYN proto-oncogene, Src family tyrosine kinase | SLK\|SYN\|p59-FYN | 6 | NaturalKiller_Cell_Cytotoxicity |
| SHC2 | 25759 | SHC adaptor protein 2 | SCK\|SHCB\|SLI | 19 | NaturalKiller_Cell_Cytotoxicity |
| SHC4 | 399694 | SHC adaptor protein 4 | RaLP\|SHCD | 15 | NaturalKiller_Cell_Cytotoxicity |
| SHC3 | 53358 | SHC adaptor protein 3 | N-Shc\|NSHC\|RAI\|SHCC | 9 | NaturalKiller_Cell_Cytotoxicity |
| SHC1 | 6464 | SHC adaptor protein 1 | SHC\|SHCA | 1 | NaturalKiller_Cell_Cytotoxicity |
| GRB2 | 2885 | growth factor receptor bound protein 2 | ASH\|EGFRBP-GRB2\|Grb3-3\|MST084\|MSTP084\|NCKAP2 | 17 | NaturalKiller_Cell_Cytotoxicity |
| SOS1 | 6654 | SOS Ras/Rac guanine nucleotide exchange factor 1 | GF1\|GGF1\|GINGF\|HGF\|NS4\|SOS-1 | 2 | NaturalKiller_Cell_Cytotoxicity |
| SOS2 | 6655 | SOS Ras/Rho guanine nucleotide exchange factor 2 | NS9\|SOS-2 | 14 | NaturalKiller_Cell_Cytotoxicity |
| ARAF | 369 | A-Raf proto-oncogene, serine/threonine kinase | A-RAF\|ARAF1\|PKS2\|RAFA1 | X | NaturalKiller_Cell_Cytotoxicity |
| BRAF | 673 | B-Raf proto-oncogene, serine/threonine kinase | B-RAF1\|B-raf\|BRAF1\|NS7\|RAFB1 | 7 | NaturalKiller_Cell_Cytotoxicity |
| RAF1 | 5894 | Raf-1 proto-oncogene, serine/threonine kinase | CMD1NN\|CRAF\|NS5\|Raf-1\|c-Raf | 3 | NaturalKiller_Cell_Cytotoxicity |
| HCST | 10870 | hematopoietic cell signal transducer | DAP10\|KAP10\|PIK3AP | 19 | NaturalKiller_Cell_Cytotoxicity |
| CD48 | 962 | CD48 molecule | BCM1\|BLAST\|BLAST1\|MEM-102\|SLAMF2\|hCD48\|mCD48 | 1 | NaturalKiller_Cell_Cytotoxicity |
| CD244 | 51744 | CD244 molecule | 2B4\|NAIL\|NKR2B4\|Nmrk\|SLAMF4 | 1 | NaturalKiller_Cell_Cytotoxicity |
| PRKCA | 5578 | protein kinase C alpha | AAG6\|PKC-alpha\|PKCA\|PKCI+/-\|PKCalpha\|PRKACA | 17 | NaturalKiller_Cell_Cytotoxicity |
| PRKCG | 5582 | protein kinase C gamma | PKC-gamma\|PKCC\|PKCG\|PKCI(3)\|PKCgamma\|SCA14 | 19 | NaturalKiller_Cell_Cytotoxicity |
| SH2D1B | 117157 | SH2 domain containing 1B | EAT2 | 1 | NaturalKiller_Cell_Cytotoxicity |
| SH2D1A | 4068 | SH2 domain containing 1A | DSHP\|EBVS\|IMD5\|LYP\|MTCP1\|SAP\|SAP/SH2D1A\|XLP\|XLPD\|XLPD1 | X | NaturalKiller_Cell_Cytotoxicity |
| FAS | 355 | Fas cell surface death receptor | ALPS1A\|APO-1\|APT1\|CD95\|FAS1\|FASTM\|TNFRSF6 | 10 | NaturalKiller_Cell_Cytotoxicity |
| GZMB | 3002 | granzyme B | C11\|CCPI\|CGL-1\|CGL1\|CSP-B\|CSPB\|CTLA1\|CTSGL1\|HLP\|SECT | 14 | NaturalKiller_Cell_Cytotoxicity |
| PRF1 | 5551 | perforin 1 | HPLH2\|P1\|PFP | 10 | NaturalKiller_Cell_Cytotoxicity |
| CASP3 | 836 | caspase 3 | CPP32\|CPP32B\|SCA-1 | 4 | NaturalKiller_Cell_Cytotoxicity |
| BID | 637 | BH3 interacting domain death agonist | FP497 | 22 | NaturalKiller_Cell_Cytotoxicity |
| CD3D | 915 | CD3d molecule | CD3-DELTA\|IMD19\|T3D | 11 | TCRsignalingPathway |
| CD3E | 916 | CD3e molecule | IMD18\|T3E\|TCRE | 11 | TCRsignalingPathway |
| CD3G | 917 | CD3g molecule | CD3-GAMMA\|IMD17\|T3G | 11 | TCRsignalingPathway |
| PTPRC | 5788 | protein tyrosine phosphatase receptor type C | B220\|CD45\|CD45R\|GP180\|L-CA\|LCA\|LY5\|T200 | 1 | TCRsignalingPathway |
| ITK | 3702 | IL2 inducible T cell kinase | EMT\|LPFS1\|LYK\|PSCTK2 | 5 | TCRsignalingPathway |
| TEC | 7006 | tec protein tyrosine kinase | PSCTK4 | 4 | TCRsignalingPathway |
| NCK1 | 4690 | NCK adaptor protein 1 | NCK\|NCKalpha\|nck-1 | 3 | TCRsignalingPathway |
| NCK2 | 8440 | NCK adaptor protein 2 | GRB4\|NCKbeta | 2 | TCRsignalingPathway |
| GRAP2 | 9402 | GRB2 related adaptor protein 2 | GADS\|GRAP-2\|GRB2L\|GRBLG\|GRID\|GRPL\|GrbX\|Grf40\|Mona\|P38 | 22 | TCRsignalingPathway |
| PAK2 | 5062 | p21 (RAC1) activated kinase 2 | PAK65\|PAKgamma | 3 | TCRsignalingPathway |
| PAK3 | 5063 | p21 (RAC1) activated kinase 3 | ARA\|MRX30\|MRX47\|OPHN3\|PAK-3\|PAK3beta\|bPAK\|beta-PAK | X | TCRsignalingPathway |
| PAK4 | 10298 | p21 (RAC1) activated kinase 4 | - | 19 | TCRsignalingPathway |
| PAK6 | 56924 | p21 (RAC1) activated kinase 6 | PAK5 | 15 | TCRsignalingPathway |
| PAK5 | 57144 | p21 (RAC1) activated kinase 5 | PAK7 | 20 | TCRsignalingPathway |
| RHOA | 387 | ras homolog family member A | ARH12\|ARHA\|EDFAOB\|RHO12\|RHOH12 | 3 | TCRsignalingPathway |
| CDC42 | 998 | cell division cycle 42 | CDC42Hs\|G25K\|TKS | 1 | TCRsignalingPathway |
| CD28 | 940 | CD28 molecule | Tp44 | 2 | TCRsignalingPathway |
| ICOS | 29851 | inducible T cell costimulator | AILIM\|CD278\|CVID1 | 2 | TCRsignalingPathway |
| MAP3K8 | 1326 | mitogen-activated protein kinase kinase kinase 8 | AURA2\|COT\|EST\|ESTF\|MEKK8\|TPL2\|Tpl-2\|c-COT | 10 | TCRsignalingPathway |
| MAP3K14 | 9020 | mitogen-activated protein kinase kinase kinase 14 | FTDCR1B\|HS\|HSNIK\|NIK | 17 | TCRsignalingPathway |
| CTLA4 | 1493 | cytotoxic T-lymphocyte associated protein 4 | ALPS5\|CD\|CD152\|CELIAC3\|CTLA-4\|GRD4\|GSE\|IDDM12 | 2 | TCRsignalingPathway |
| CBLC | 23624 | Cbl proto-oncogene C | CBL-3\|CBL-SL\|RNF57 | 19 | TCRsignalingPathway |
| CBL | 867 | Cbl proto-oncogene | C-CBL\|CBL2\|FRA11B\|NSLL\|RNF55 | 11 | TCRsignalingPathway |
| CBLB | 868 | Cbl proto-oncogene B | Cbl-b\|Nbla00127\|RNF56 | 3 | TCRsignalingPathway |
| CDK4 | 1019 | cyclin dependent kinase 4 | CMM3\|PSK-J3 | 12 | TCRsignalingPathway |
| RASGRP1 | 10125 | RAS guanyl releasing protein 1 | CALDAG-GEFI\|CALDAG-GEFII\|IMD64\|RASGRP | 15 | TCRsignalingPathway |
| PDK1 | 5163 | pyruvate dehydrogenase kinase 1 | - | 2 | TCRsignalingPathway |
| PRKCQ | 5588 | protein kinase C theta | PRKCT\|nPKC-theta | 10 | TCRsignalingPathway |
| TRAC | 28755 | T cell receptor alpha constant | IMD7\|TCRA\|TRA\|TRCA | 14 | TCRsignalingPathway |
| TRAJ1 | 28754 | T cell receptor alpha joining 1 (non-functional) | - | 14 | TCRsignalingPathway |
| TRAJ2 | 28753 | T cell receptor alpha joining 2 (non-functional) | - | 14 | TCRsignalingPathway |
| TRAJ3 | 28752 | T cell receptor alpha joining 3 | - | 14 | TCRsignalingPathway |
| TRAJ4 | 28751 | T cell receptor alpha joining 4 | - | 14 | TCRsignalingPathway |
| TRAJ5 | 28750 | T cell receptor alpha joining 5 | - | 14 | TCRsignalingPathway |
| TRAJ6 | 28749 | T cell receptor alpha joining 6 | - | 14 | TCRsignalingPathway |
| TRAJ7 | 28748 | T cell receptor alpha joining 7 | - | 14 | TCRsignalingPathway |
| TRAJ8 | 28747 | T cell receptor alpha joining 8 | - | 14 | TCRsignalingPathway |
| TRAJ9 | 28746 | T cell receptor alpha joining 9 | - | 14 | TCRsignalingPathway |
| TRAJ10 | 28745 | T cell receptor alpha joining 10 | - | 14 | TCRsignalingPathway |
| TRAJ11 | 28744 | T cell receptor alpha joining 11 | - | 14 | TCRsignalingPathway |
| TRAJ12 | 28743 | T cell receptor alpha joining 12 | - | 14 | TCRsignalingPathway |
| TRAJ13 | 28742 | T cell receptor alpha joining 13 | - | 14 | TCRsignalingPathway |
| TRAJ14 | 28741 | T cell receptor alpha joining 14 | - | 14 | TCRsignalingPathway |
| TRAJ15 | 28740 | T cell receptor alpha joining 15 | - | 14 | TCRsignalingPathway |
| TRAJ16 | 28739 | T cell receptor alpha joining 16 | - | 14 | TCRsignalingPathway |
| TRAJ17 | 28738 | T cell receptor alpha joining 17 | - | 14 | TCRsignalingPathway |
| TRAJ18 | 28737 | T cell receptor alpha joining 18 | - | 14 | TCRsignalingPathway |
| TRAJ19 | 28736 | T cell receptor alpha joining 19 (non-functional) | - | 14 | TCRsignalingPathway |
| TRAJ20 | 28735 | T cell receptor alpha joining 20 | - | 14 | TCRsignalingPathway |
| TRAJ21 | 28734 | T cell receptor alpha joining 21 | - | 14 | TCRsignalingPathway |
| TRAJ22 | 28733 | T cell receptor alpha joining 22 | - | 14 | TCRsignalingPathway |
| TRAJ23 | 28732 | T cell receptor alpha joining 23 | - | 14 | TCRsignalingPathway |
| TRAJ24 | 28731 | T cell receptor alpha joining 24 | - | 14 | TCRsignalingPathway |
| TRAJ25 | 28730 | T cell receptor alpha joining 25 (non-functional) | - | 14 | TCRsignalingPathway |
| TRAJ26 | 28729 | T cell receptor alpha joining 26 | - | 14 | TCRsignalingPathway |
| TRAJ27 | 28728 | T cell receptor alpha joining 27 | - | 14 | TCRsignalingPathway |
| TRAJ28 | 28727 | T cell receptor alpha joining 28 | - | 14 | TCRsignalingPathway |
| TRAJ29 | 28726 | T cell receptor alpha joining 29 | - | 14 | TCRsignalingPathway |
| TRAJ30 | 28725 | T cell receptor alpha joining 30 | - | 14 | TCRsignalingPathway |
| TRAJ31 | 28724 | T cell receptor alpha joining 31 | - | 14 | TCRsignalingPathway |
| TRAJ32 | 28723 | T cell receptor alpha joining 32 | - | 14 | TCRsignalingPathway |
| TRAJ33 | 28722 | T cell receptor alpha joining 33 | - | 14 | TCRsignalingPathway |
| TRAJ34 | 28721 | T cell receptor alpha joining 34 | - | 14 | TCRsignalingPathway |
| TRAJ35 | 28720 | T cell receptor alpha joining 35 (non-functional) | - | 14 | TCRsignalingPathway |
| TRAJ36 | 28719 | T cell receptor alpha joining 36 | - | 14 | TCRsignalingPathway |
| TRAJ37 | 28718 | T cell receptor alpha joining 37 | - | 14 | TCRsignalingPathway |
| TRAJ38 | 28717 | T cell receptor alpha joining 38 | - | 14 | TCRsignalingPathway |
| TRAJ39 | 28716 | T cell receptor alpha joining 39 | - | 14 | TCRsignalingPathway |
| TRAJ40 | 28715 | T cell receptor alpha joining 40 | - | 14 | TCRsignalingPathway |
| TRAJ41 | 28714 | T cell receptor alpha joining 41 | - | 14 | TCRsignalingPathway |
| TRAJ42 | 28713 | T cell receptor alpha joining 42 | - | 14 | TCRsignalingPathway |
| TRAJ43 | 28712 | T cell receptor alpha joining 43 | - | 14 | TCRsignalingPathway |
| TRAJ44 | 28711 | T cell receptor alpha joining 44 | - | 14 | TCRsignalingPathway |
| TRAJ45 | 28710 | T cell receptor alpha joining 45 | - | 14 | TCRsignalingPathway |
| TRAJ46 | 28709 | T cell receptor alpha joining 46 | - | 14 | TCRsignalingPathway |
| TRAJ47 | 28708 | T cell receptor alpha joining 47 | - | 14 | TCRsignalingPathway |
| TRAJ48 | 28707 | T cell receptor alpha joining 48 | - | 14 | TCRsignalingPathway |
| TRAJ49 | 28706 | T cell receptor alpha joining 49 | - | 14 | TCRsignalingPathway |
| TRAJ50 | 28705 | T cell receptor alpha joining 50 | - | 14 | TCRsignalingPathway |
| TRAJ52 | 28703 | T cell receptor alpha joining 52 | - | 14 | TCRsignalingPathway |
| TRAJ53 | 28702 | T cell receptor alpha joining 53 | - | 14 | TCRsignalingPathway |
| TRAJ54 | 28701 | T cell receptor alpha joining 54 | - | 14 | TCRsignalingPathway |
| TRAJ56 | 28699 | T cell receptor alpha joining 56 | - | 14 | TCRsignalingPathway |
| TRAJ57 | 28698 | T cell receptor alpha joining 57 | - | 14 | TCRsignalingPathway |
| TRAJ58 | 28697 | T cell receptor alpha joining 58 (non-functional) | - | 14 | TCRsignalingPathway |
| TRAJ59 | 28696 | T cell receptor alpha joining 59 (non-functional) | - | 14 | TCRsignalingPathway |
| TRAJ61 | 28694 | T cell receptor alpha joining 61 (non-functional) | - | 14 | TCRsignalingPathway |
| TRAV1-1 | 28693 | T cell receptor alpha variable 1-1 | TCRAV1S1\|TCRAV7S1\|TRAV11 | 14 | TCRsignalingPathway |
| TRAV1-2 | 28692 | T cell receptor alpha variable 1-2 | TCRAV1S2\|TCRAV7S2\|TRAV12 | 14 | TCRsignalingPathway |
| TRAV2 | 28691 | T cell receptor alpha variable 2 | TCRAV11S1\|TCRAV2S1 | 14 | TCRsignalingPathway |
| TRAV3 | 28690 | T cell receptor alpha variable 3 | TCRAV16S1\|TCRAV3S1 | 14 | TCRsignalingPathway |
| TRAV4 | 28689 | T cell receptor alpha variable 4 | TCRAV20S1\|TCRAV4S1 | 14 | TCRsignalingPathway |
| TRAV5 | 28688 | T cell receptor alpha variable 5 | TCRAV15S1\|TCRAV5S1 | 14 | TCRsignalingPathway |
| TRAV7 | 28686 | T cell receptor alpha variable 7 | TCRAV7S1 | 14 | TCRsignalingPathway |
| TRAV8-1 | 28685 | T cell receptor alpha variable 8-1 | TCRAV1S1\|TCRAV8S1\|TRAV81 | 14 | TCRsignalingPathway |
| TRAV8-2 | 28684 | T cell receptor alpha variable 8-2 | TCRAV1S5\|TCRAV8S2\|TRAV82 | 14 | TCRsignalingPathway |
| TRAV8-3 | 28683 | T cell receptor alpha variable 8-3 | TCRAV1S4\|TCRAV8S3\|TRAV83 | 14 | TCRsignalingPathway |
| TRAV8-4 | 28682 | T cell receptor alpha variable 8-4 | TCRAV1S2\|TCRAV8S4\|TRAV84 | 14 | TCRsignalingPathway |
| TRAV8-6 | 28680 | T cell receptor alpha variable 8-6 | TCRAV1S3\|TCRAV8S6\|TRAV86 | 14 | TCRsignalingPathway |
| TRAV8-7 | 28679 | T cell receptor alpha variable 8-7 (pseudogene) | TCRAV8S7\|TRAV87 | 14 | TCRsignalingPathway |
| TRAV9-1 | 28678 | T cell receptor alpha variable 9-1 | TCRAV9S1\|TRAV91 | 14 | TCRsignalingPathway |
| TRAV9-2 | 28677 | T cell receptor alpha variable 9-2 | TCRAV22S1\|TCRAV9S2\|TRAV92 | 14 | TCRsignalingPathway |
| TRAV10 | 28676 | T cell receptor alpha variable 10 | TCRAV10S1\|TCRAV24S1 | 14 | TCRsignalingPathway |
| TRAV12-1 | 28674 | T cell receptor alpha variable 12-1 | TCRAV12S1\|TCRAV2S3\|TRAV121 | 14 | TCRsignalingPathway |
| TRAV12-2 | 28673 | T cell receptor alpha variable 12-2 | TCRAV12S2\|TCRAV2S1\|TRAV122 | 14 | TCRsignalingPathway |
| TRAV12-3 | 28672 | T cell receptor alpha variable 12-3 | TCRAV12S3\|TCRAV2S2\|TRAV123 | 14 | TCRsignalingPathway |
| TRAV13-1 | 28671 | T cell receptor alpha variable 13-1 | TCRAV13S1\|TCRAV8S1\|TRAV131 | 14 | TCRsignalingPathway |
| TRAV13-2 | 28670 | T cell receptor alpha variable 13-2 | TCRAV13S2\|TCRAV8S2\|TRAV132 | 14 | TCRsignalingPathway |
| TRAV14DV4 | 28669 | T cell receptor alpha variable 14/delta variable 4 | TCRAV6S1-hDV104S1\|TRAV14/DV4\|hADV14S1 | 14 | TCRsignalingPathway |
| TRAV16 | 28667 | T cell receptor alpha variable 16 | TCRAV16S1\|TCRAV9S1 | 14 | TCRsignalingPathway |
| TRAV17 | 28666 | T cell receptor alpha variable 17 | TCRAV17S1\|TCRAV3S1 | 14 | TCRsignalingPathway |
| TRAV18 | 28665 | T cell receptor alpha variable 18 | TCRAV18S1 | 14 | TCRsignalingPathway |
| TRAV19 | 28664 | T cell receptor alpha variable 19 | TCRAV12S1\|TCRAV19S1 | 14 | TCRsignalingPathway |
| TRAV20 | 28663 | T cell receptor alpha variable 20 | TCRAV20S1\|TCRAV30S1 | 14 | TCRsignalingPathway |
| TRAV21 | 28662 | T cell receptor alpha variable 21 | TCRAV21S1\|TCRAV23S1 | 14 | TCRsignalingPathway |
| TRAV22 | 28661 | T cell receptor alpha variable 22 | TCRAV13S1\|TCRAV22S1 | 14 | TCRsignalingPathway |
| TRAV23DV6 | 28660 | T cell receptor alpha variable 23/delta variable 6 | TCRAV17S1\|TRAV23/DV6\|hADV23S1 | 14 | TCRsignalingPathway |
| TRAV24 | 28659 | T cell receptor alpha variable 24 | TCRAV18S1\|TCRAV24S1 | 14 | TCRsignalingPathway |
| TRAV25 | 28658 | T cell receptor alpha variable 25 | TCRAV25S1\|TCRAV32S1 | 14 | TCRsignalingPathway |
| TRAV26-1 | 28657 | T cell receptor alpha variable 26-1 | TCRAV26S1\|TCRAV4S2\|TRAV261 | 14 | TCRsignalingPathway |
| TRAV26-2 | 28656 | T cell receptor alpha variable 26-2 | TCRAV26S2\|TCRAV4S1\|TRAV262 | 14 | TCRsignalingPathway |
| TRAV27 | 28655 | T cell receptor alpha variable 27 | TCRAV10S1\|TCRAV27S1 | 14 | TCRsignalingPathway |
| TRAV29DV5 | 28653 | T cell receptor alpha variable 29/delta variable 5 | TCRA\|TCRAV21S1\|TRAV29/DV5\|hADV29S1 | 14 | TCRsignalingPathway |
| TRAV30 | 28652 | T cell receptor alpha variable 30 | TCRAV29S1\|TCRAV30S1 | 14 | TCRsignalingPathway |
| TRAV34 | 28648 | T cell receptor alpha variable 34 | TCRAV26S1\|TCRAV34S1 | 14 | TCRsignalingPathway |
| TRAV35 | 28647 | T cell receptor alpha variable 35 | TCRAV25S1\|TCRAV35S1 | 14 | TCRsignalingPathway |
| TRAV36DV7 | 28646 | T cell receptor alpha variable 36/delta variable 7 | TCRAV28S1\|TRAV36/DV7\|hADV36S1 | 14 | TCRsignalingPathway |
| TRAV38-1 | 28644 | T cell receptor alpha variable 38-1 | TCRAV14S2\|TCRAV38S1\|TRAV381 | 14 | TCRsignalingPathway |
| TRAV38-2DV8 | 28643 | T cell receptor alpha variable 38-2/delta variable 8 | TCRAV14S1\|TRAV382DV8\|hADV38S2 | 14 | TCRsignalingPathway |
| TRAV39 | 28642 | T cell receptor alpha variable 39 | TCRAV27S1\|TCRAV39S1 | 14 | TCRsignalingPathway |
| TRAV40 | 28641 | T cell receptor alpha variable 40 | TCRAV31S1\|TCRAV40S1 | 14 | TCRsignalingPathway |
| TRAV41 | 28640 | T cell receptor alpha variable 41 | TCRAV19S1\|TCRAV41S1 | 14 | TCRsignalingPathway |
| TRBC1 | 28639 | T cell receptor beta constant 1 | BV05S1J2.2\|TCRB\|TCRBC1 | 7 | TCRsignalingPathway |
| TRBC2 | 28638 | T cell receptor beta constant 2 | TCRBC2 | 7 | TCRsignalingPathway |
| TRBD1 | 28637 | T cell receptor beta diversity 1 | TCRBD1 | 7 | TCRsignalingPathway |
| TRBD2 | 28636 | T cell receptor beta diversity 2 | TCRBD2 | 7 | TCRsignalingPathway |
| TRBJ1-1 | 28635 | T cell receptor beta joining 1-1 | TCRBJ1S1\|TRBJ11 | 7 | TCRsignalingPathway |
| TRBJ1-2 | 28634 | T cell receptor beta joining 1-2 | TCRBJ1S2\|TRBJ12 | 7 | TCRsignalingPathway |
| TRBJ1-3 | 28633 | T cell receptor beta joining 1-3 | TCRBJ1S3\|TRBJ13 | 7 | TCRsignalingPathway |
| TRBJ1-4 | 28632 | T cell receptor beta joining 1-4 | TCRBJ1S4\|TRBJ14 | 7 | TCRsignalingPathway |
| TRBJ1-5 | 28631 | T cell receptor beta joining 1-5 | TCRBJ1S5\|TRBJ15 | 7 | TCRsignalingPathway |
| TRBJ1-6 | 28630 | T cell receptor beta joining 1-6 | TCRBJ1S6\|TRBJ16 | 7 | TCRsignalingPathway |
| TRBJ2-1 | 28629 | T cell receptor beta joining 2-1 | TCRBJ2S1\|TRBJ21 | 7 | TCRsignalingPathway |
| TRBJ2-2 | 28628 | T cell receptor beta joining 2-2 | TCRBJ2S2\|TRBJ22 | 7 | TCRsignalingPathway |
| TRBJ2-3 | 28626 | T cell receptor beta joining 2-3 | TCRBJ2S3\|TRBJ23 | 7 | TCRsignalingPathway |
| TRBJ2-4 | 28625 | T cell receptor beta joining 2-4 | TCRBJ2S4\|TRBJ24 | 7 | TCRsignalingPathway |
| TRBJ2-5 | 28624 | T cell receptor beta joining 2-5 | TCRBJ2S5\|TRBJ25 | 7 | TCRsignalingPathway |
| TRBJ2-6 | 28623 | T cell receptor beta joining 2-6 | TCRBJ2S6\|TRBJ26 | 7 | TCRsignalingPathway |
| TRBJ2-7 | 28622 | T cell receptor beta joining 2-7 | TCRBJ2S7\|TRBJ27 | 7 | TCRsignalingPathway |
| TRBV2 | 28620 | T cell receptor beta variable 2 | TCRBV22S1A2N1T\|TCRBV2S1 | 7 | TCRsignalingPathway |
| TRBV3-1 | 28619 | T cell receptor beta variable 3-1 | TCRBV3S1\|TCRBV9S1A1T\|TRBV31 | 7 | TCRsignalingPathway |
| TRBV4-1 | 28617 | T cell receptor beta variable 4-1 | BV07S1J2.7\|TCRBV4S1\|TCRBV7S1A1N2T\|TRBV41 | 7 | TCRsignalingPathway |
| TRBV4-2 | 28616 | T cell receptor beta variable 4-2 | TCRBV4S2\|TCRBV7S3A2\|TCRBV7S3A2T\|TRBV42 | 7 | TCRsignalingPathway |
| TRBV4-3 | 28615 | T cell receptor beta variable 4-3 | TCRBV4S3\|TCRBV7S2A1N4T\|TRBV43 | 7 | TCRsignalingPathway |
| TRBV5-1 | 28614 | T cell receptor beta variable 5-1 | TCRBV5S1\|TCRBV5S1A1T\|TRBV51 | 7 | TCRsignalingPathway |
| TRBV5-4 | 28611 | T cell receptor beta variable 5-4 | TCRBV5S4\|TCRBV5S6A3N2T\|TRBV54 | 7 | TCRsignalingPathway |
| TRBV5-5 | 28610 | T cell receptor beta variable 5-5 | TCRBV5S3A2T\|TCRBV5S5\|TRBV55 | 7 | TCRsignalingPathway |
| TRBV5-6 | 28609 | T cell receptor beta variable 5-6 | TCRBV5S2\|TCRBV5S6\|TRBV56 | 7 | TCRsignalingPathway |
| TRBV5-7 | 28608 | T cell receptor beta variable 5-7 (non-functional) | TCRBV5S7\|TCRBV5S7P\|TRBV57 | 7 | TCRsignalingPathway |
| TRBV5-8 | 28607 | T cell receptor beta variable 5-8 | TCRBV5S4A2T\|TCRBV5S8\|TRBV58 | 7 | TCRsignalingPathway |
| TRBV6-1 | 28606 | T cell receptor beta variable 6-1 | TCRBV13S3\|TCRBV6S1\|TRBV61 | 7 | TCRsignalingPathway |
| TRBV6-2 | 28605 | T cell receptor beta variable 6-2 | TCRBV13S2\|TCRBV13S2A1T\|TCRBV6S2\|TRBV62 | 7 | TCRsignalingPathway |
| TRBV6-3 | 28604 | T cell receptor beta variable 6-3 | TCRBV13S9/13S2A1T\|TCRBV6S3\|TRBV63 | 7 | TCRsignalingPathway |
| TRBV6-4 | 28603 | T cell receptor beta variable 6-4 | TCRBV13S5\|TCRBV6S4\|TRBV64 | 7 | TCRsignalingPathway |
| TRBV6-5 | 28602 | T cell receptor beta variable 6-5 | TCRBV13S1\|TCRBV6S5\|TRBV65 | 7 | TCRsignalingPathway |
| TRBV6-6 | 28601 | T cell receptor beta variable 6-6 | TCRBV13S6A2T\|TCRBV6S6\|TRBV66 | 7 | TCRsignalingPathway |
| TRBV6-7 | 28600 | T cell receptor beta variable 6-7 (non-functional) | TCRBV13S8P\|TCRBV6S7\|TRBV67 | 7 | TCRsignalingPathway |
| TRBV6-8 | 28599 | T cell receptor beta variable 6-8 | TCRBV13S7P\|TCRBV6S8\|TRBV68 | 7 | TCRsignalingPathway |
| TRBV6-9 | 28598 | T cell receptor beta variable 6-9 | TCRBV13S4\|TCRBV6S9\|TRBV69 | 7 | TCRsignalingPathway |
| TRBV7-2 | 28596 | T cell receptor beta variable 7-2 | TCRBV6S5A1N1\|TCRBV6S5A2\|TCRBV7S2\|TRBV72 | 7 | TCRsignalingPathway |
| TRBV7-3 | 28595 | T cell receptor beta variable 7-3 | TCRBV6S1A1N1\|TCRBV7S3\|TRBV73 | 7 | TCRsignalingPathway |
| TRBV7-4 | 28594 | T cell receptor beta variable 7-4 | TCRBV6S8A2T\|TCRBV7S4\|TRBV74 | 7 | TCRsignalingPathway |
| TRBV7-6 | 28592 | T cell receptor beta variable 7-6 | TCRBV6S3A1N1T\|TCRBV7S6\|TRBV76 | 7 | TCRsignalingPathway |
| TRBV7-7 | 28591 | T cell receptor beta variable 7-7 | TCRBV6S6A2T\|TCRBV7S7\|TRBV77 | 7 | TCRsignalingPathway |
| TRBV7-8 | 28590 | T cell receptor beta variable 7-8 | TCRBV6S2A1N1T\|TCRBV7S8\|TRBV78 | 7 | TCRsignalingPathway |
| TRBV7-9 | 28589 | T cell receptor beta variable 7-9 | TCRB\|TCRBV6S4A1\|TCRBV7S9\|TRBV79 | 7 | TCRsignalingPathway |
| TRBV9 | 28586 | T cell receptor beta variable 9 | TCRBV1S1A1N1\|TCRBV9S1 | 7 | TCRsignalingPathway |
| TRBV10-1 | 28585 | T cell receptor beta variable 10-1 | TCRBV10S1\|TCRBV12S2\|TCRBV12S2A1T\|TRBV101 | 7 | TCRsignalingPathway |
| TRBV10-2 | 28584 | T cell receptor beta variable 10-2 | TCRBV10S2\|TCRBV12S3\|TRBV102 | 7 | TCRsignalingPathway |
| TRBV10-3 | 28583 | T cell receptor beta variable 10-3 | TCRBV10S3\|TCRBV12S1A1N2\|TRBV103 | 7 | TCRsignalingPathway |
| TRBV11-1 | 28582 | T cell receptor beta variable 11-1 | TCRBV11S1\|TCRBV21S1\|TRBV111 | 7 | TCRsignalingPathway |
| TRBV11-2 | 28581 | T cell receptor beta variable 11-2 | TCRBV11S2\|TCRBV21S3A2N2T\|TRBV112 | 7 | TCRsignalingPathway |
| TRBV11-3 | 28580 | T cell receptor beta variable 11-3 | TCRBV11S3\|TCRBV21S2A2\|TRBV113 | 7 | TCRsignalingPathway |
| TRBV12-3 | 28577 | T cell receptor beta variable 12-3 | TCRBV12S3\|TCRBV8S1\|TRBV123 | 7 | TCRsignalingPathway |
| TRBV12-4 | 28576 | T cell receptor beta variable 12-4 | TCRBV12S4\|TCRBV8S2A1T\|TRBV124 | 7 | TCRsignalingPathway |
| TRBV12-5 | 28575 | T cell receptor beta variable 12-5 | TCRBV12S5\|TCRBV8S3\|TRBV125 | 7 | TCRsignalingPathway |
| TRBV13 | 28574 | T cell receptor beta variable 13 | TCRBV13S1\|TCRBV23S1A2T | 7 | TCRsignalingPathway |
| TRBV14 | 28573 | T cell receptor beta variable 14 | TCRBV14S1\|TCRBV16S1A1N1 | 7 | TCRsignalingPathway |
| TRBV15 | 28572 | T cell receptor beta variable 15 | TCRBV15S1\|TCRBV24S1A3T | 7 | TCRsignalingPathway |
| TRBV16 | 28571 | T cell receptor beta variable 16 | BV25S1J1.2\|TCRB\|TCRBV16S1\|TCRBV25S1\|TCRBV25S1A2PT | 7 | TCRsignalingPathway |
| TRBV17 | 28570 | T cell receptor beta variable 17 (non-functional) | TCRBV17S1\|TCRBV26S1P | 7 | TCRsignalingPathway |
| TRBV18 | 28569 | T cell receptor beta variable 18 | TCRBV18S1 | 7 | TCRsignalingPathway |
| TRBV19 | 28568 | T cell receptor beta variable 19 | TCRBV17S1A1T\|TCRBV19S1 | 7 | TCRsignalingPathway |
| TRBV20-1 | 28567 | T cell receptor beta variable 20-1 | TCRBV20S1\|TCRBV2S1\|TRBV201 | 7 | TCRsignalingPathway |
| TRBV24-1 | 28563 | T cell receptor beta variable 24-1 | TCRBV15S1\|TCRBV24S1\|TRBV241 | 7 | TCRsignalingPathway |
| TRBV25-1 | 28562 | T cell receptor beta variable 25-1 | TCRBV11S1A1T\|TCRBV25S1\|TRBV251 | 7 | TCRsignalingPathway |
| TRBV27 | 28560 | T cell receptor beta variable 27 | TCRBV14S1\|TCRBV27S1 | 7 | TCRsignalingPathway |
| TRBV28 | 28559 | T cell receptor beta variable 28 | TCRBV28S1\|TCRBV3S1 | 7 | TCRsignalingPathway |
| TRBV29-1 | 28558 | T cell receptor beta variable 29-1 | TCRBV29S1\|TCRBV4S1A1T\|TRBV291 | 7 | TCRsignalingPathway |
| TRBV30 | 28557 | T cell receptor beta variable 30 | TCRBV20S1A1N2\|TCRBV30S1 | 7 | TCRsignalingPathway |
| TRDC | 28526 | T cell receptor delta constant | TCRD | 14 | TCRsignalingPathway |
| TRDD1 | 28525 | T cell receptor delta diversity 1 | - | 14 | TCRsignalingPathway |
| TRDD2 | 28524 | T cell receptor delta diversity 2 | - | 14 | TCRsignalingPathway |
| TRDD3 | 28523 | T cell receptor delta diversity 3 | TCRD | 14 | TCRsignalingPathway |
| TRDJ1 | 28522 | T cell receptor delta joining 1 | TCRD | 14 | TCRsignalingPathway |
| TRDJ2 | 28521 | T cell receptor delta joining 2 | - | 14 | TCRsignalingPathway |
| TRDJ3 | 28520 | T cell receptor delta joining 3 | - | 14 | TCRsignalingPathway |
| TRDJ4 | 28519 | T cell receptor delta joining 4 | - | 14 | TCRsignalingPathway |
| TRDV1 | 28518 | T cell receptor delta variable 1 | hDV101S1 | 14 | TCRsignalingPathway |
| TRDV2 | 28517 | T cell receptor delta variable 2 | hDV102S1 | 14 | TCRsignalingPathway |
| TRDV3 | 28516 | T cell receptor delta variable 3 | hDV103S1 | 14 | TCRsignalingPathway |
| TRGV9 | 6983 | T cell receptor gamma variable 9 | TCRGV9\|TRGC1\|V2 | 7 | TCRsignalingPathway |
| TRGV8 | 6982 | T cell receptor gamma variable 8 | TCRGV8\|V1S8 | 7 | TCRsignalingPathway |
| TRGV5 | 6978 | T cell receptor gamma variable 5 | TCRGV5\|V1S5 | 7 | TCRsignalingPathway |
| TRGV4 | 6977 | T cell receptor gamma variable 4 | TCRGV4\|V1S4 | 7 | TCRsignalingPathway |
| TRGV3 | 6976 | T cell receptor gamma variable 3 | TCRGV3\|V1S3 | 7 | TCRsignalingPathway |
| TRGV2 | 6974 | T cell receptor gamma variable 2 | TCRGV2\|VIS2 | 7 | TCRsignalingPathway |
| TRGJP2 | 6972 | T cell receptor gamma joining P2 | JP2\|TCRGJP2 | 7 | TCRsignalingPathway |
| TRGJP1 | 6971 | T cell receptor gamma joining P1 | JP1\|TCRGJP1 | 7 | TCRsignalingPathway |
| TRGJP | 6970 | T cell receptor gamma joining P | JP\|TCRGJP | 7 | TCRsignalingPathway |
| TRGJ2 | 6969 | T cell receptor gamma joining 2 | J2\|TCRGJ2 | 7 | TCRsignalingPathway |
| TRGJ1 | 6968 | T cell receptor gamma joining 1 | J1\|TCRGJ1 | 7 | TCRsignalingPathway |
| TRGC2 | 6967 | T cell receptor gamma constant 2 | TCRGC2\|TRGC2(2X)\|TRGC2(3X) | 7 | TCRsignalingPathway |
| TRGC1 | 6966 | T cell receptor gamma constant 1 | C1\|TCRG\|TCRGC1 | 7 | TCRsignalingPathway |
| TRAV6 | 6956 | T cell receptor alpha variable 6 | TCRAV5S1\|TCRAV6S1 | 14 | TCRsignalingPathway |
