# Supplementary material for: Integration of ubiquitination-related genes in predictive signatures for prognosis and immunotherapy response in sarcoma
Source: Front Oncol. 2024 Oct 14;14:1446522. doi: 10.3389/fonc.2024.1446522 (PMC11513255; doi:10.3389/fonc.2024.1446522)
Supplement: Supplementary file 2 [file DataSheet2.zip › Supplementary Figures.docx]

# Supplementary Figures


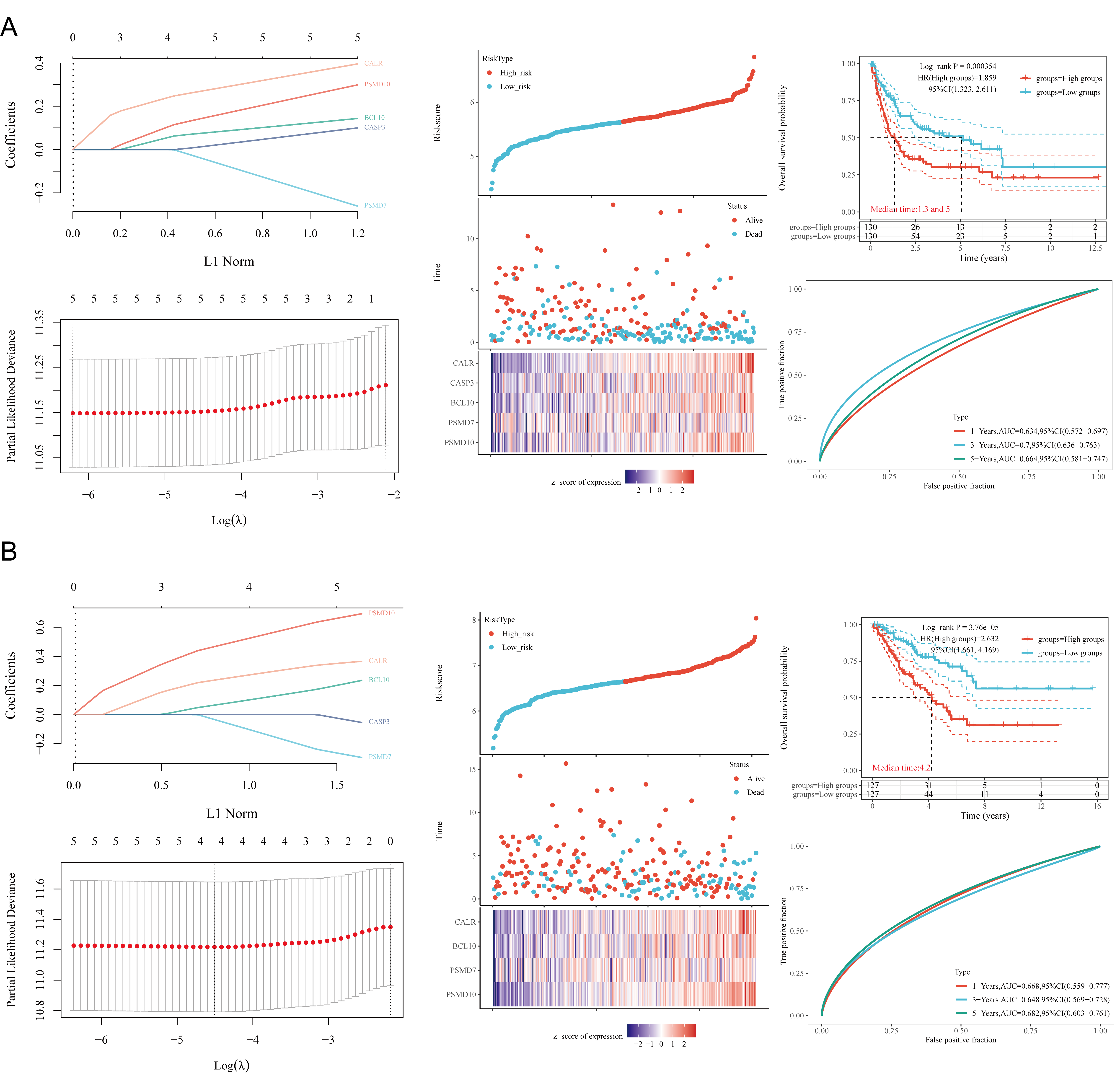


**Supplementary Figure 1.** Construction of prognostic models for URGs in SARC tissues based on PFS and DSS. (A) PFS; (B) DSS.


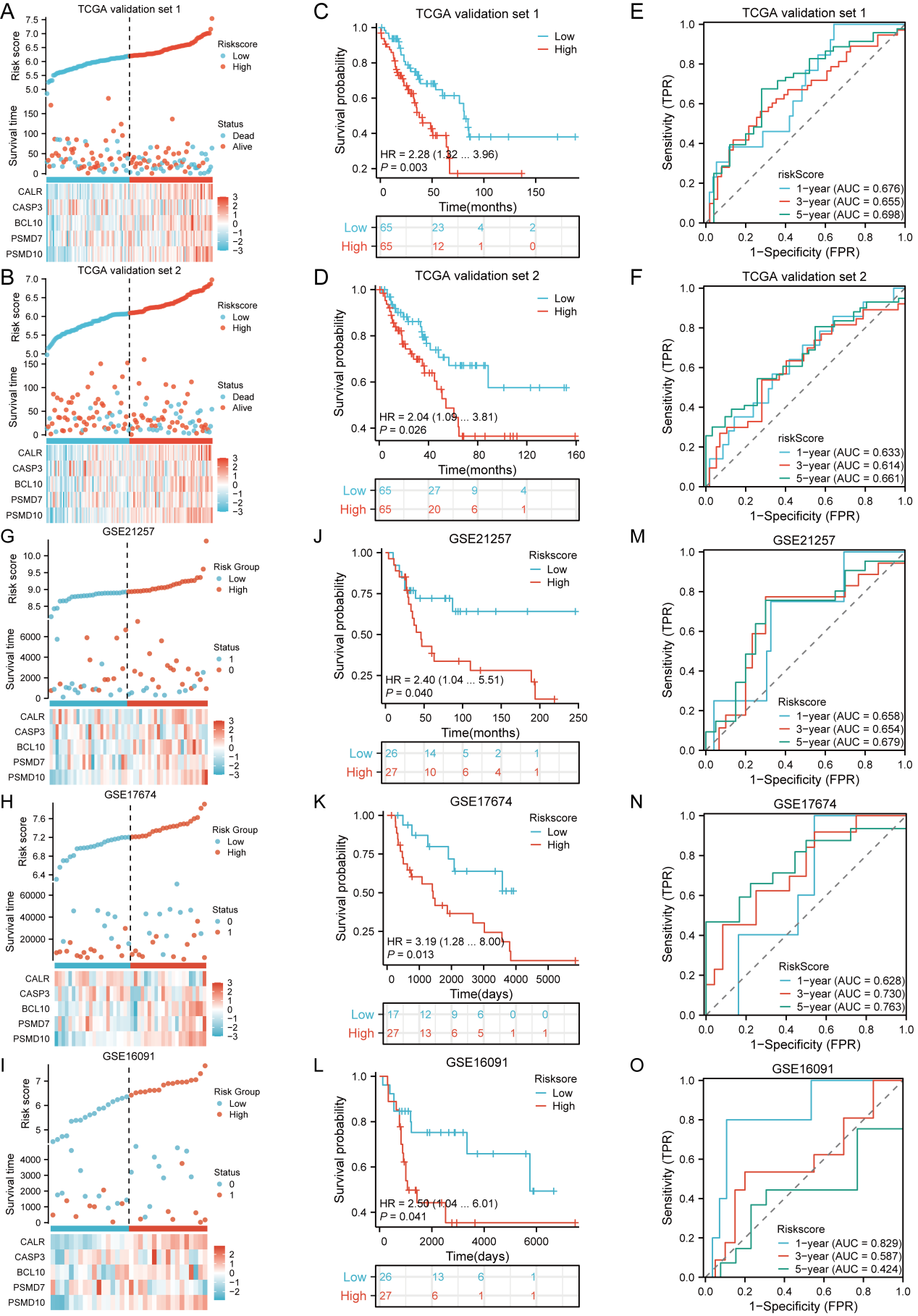


**Supplementary Figure 2.** Prognostic value of URGs signature and risk score validation in SARC patients. (A) Distribution of risk score, survival status, and expression of prognostic URGs for patients in low- and high-risk groups in TCGA validation set 1. (B) Distribution of risk score, survival status, and expression of prognostic URGs for patients in low- and high-risk groups in TCGA validation set 2. (C)Risk score and survival probabilities in TCGA validation set 1. (D) Risk score and survival probabilities in TCGA validation set 2. (E) Time-dependent ROC curve analyses of risk score in TCGA validation set 1.(F) Time-dependent ROC curve analyses of risk score in TCGA validation set 2. (G-I) Distribution of risk score, survival status, and expression of prognostic URGs for patients in low- and high-risk groups in GSE21257, GSE17674, GSE16091 dataset. (J-L) Risk score and survival probabilities in GSE21257, GSE17674, GSE16091 dataset. (M-O) Time-dependent ROC curve analyses of risk score GSE21257, GSE17674, GSE16091 dataset.


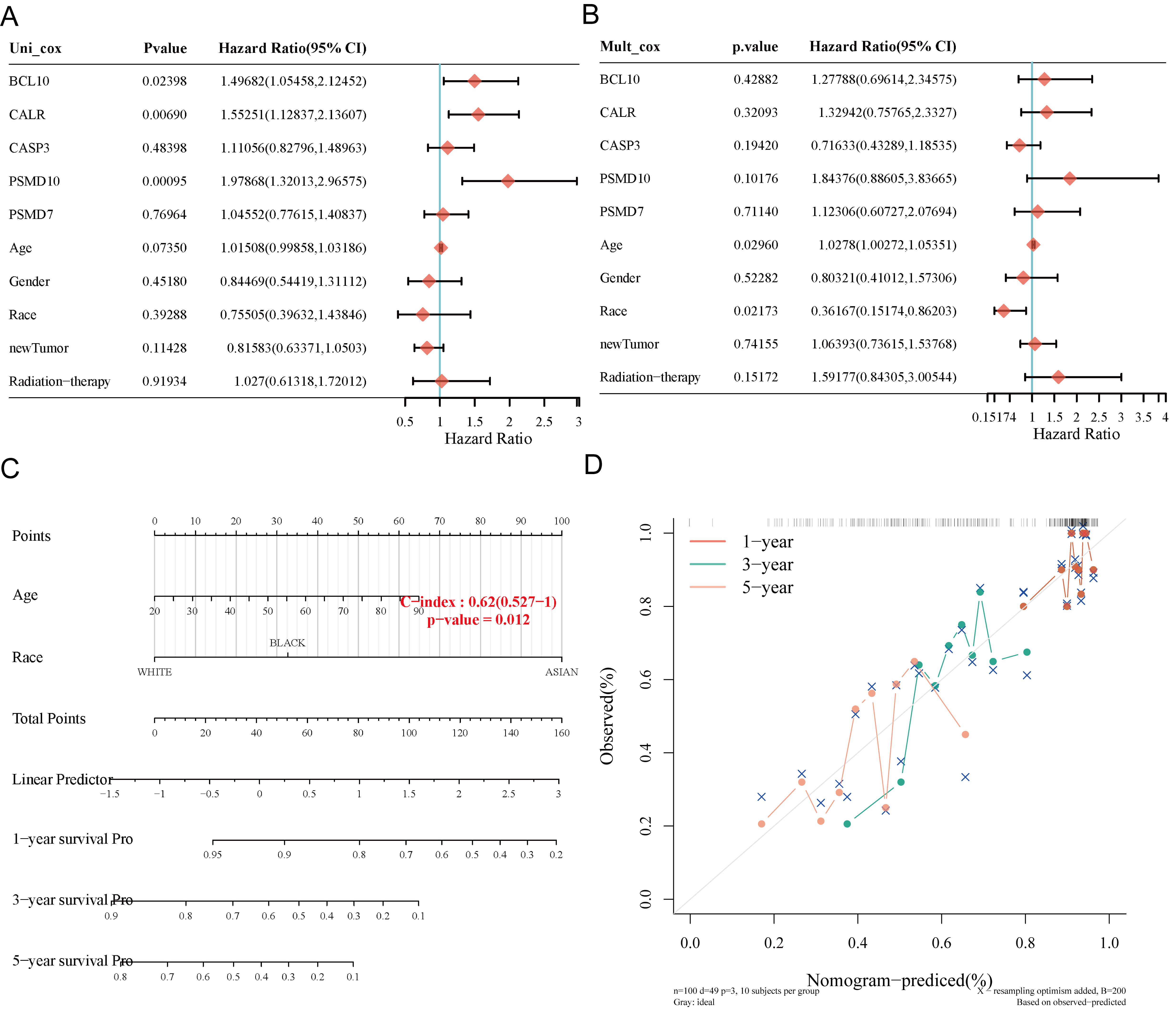


**Supplementary Figure 3.** Construction of the prediction nomogram (DSS) (A, B) Univariate and multivariate Cox regression analysis of clinicopathological features and URGs in SARC patients for DSS; (C) Nomogram for predicting 1-, 3-, and 5-year DSS of SARC patients; (D) Calibration curve of the DSS nomogram model in the discovery group. The diagonal dotted line represents the ideal nomogram. **Supplementary Figure 4** The relationship between URGs expression levels and immune infiltration in the tumor microenvironment. (A) TIMER, (B) quantTIseq, (C) MCP-counter, (D) xCell, (E) EPIC.
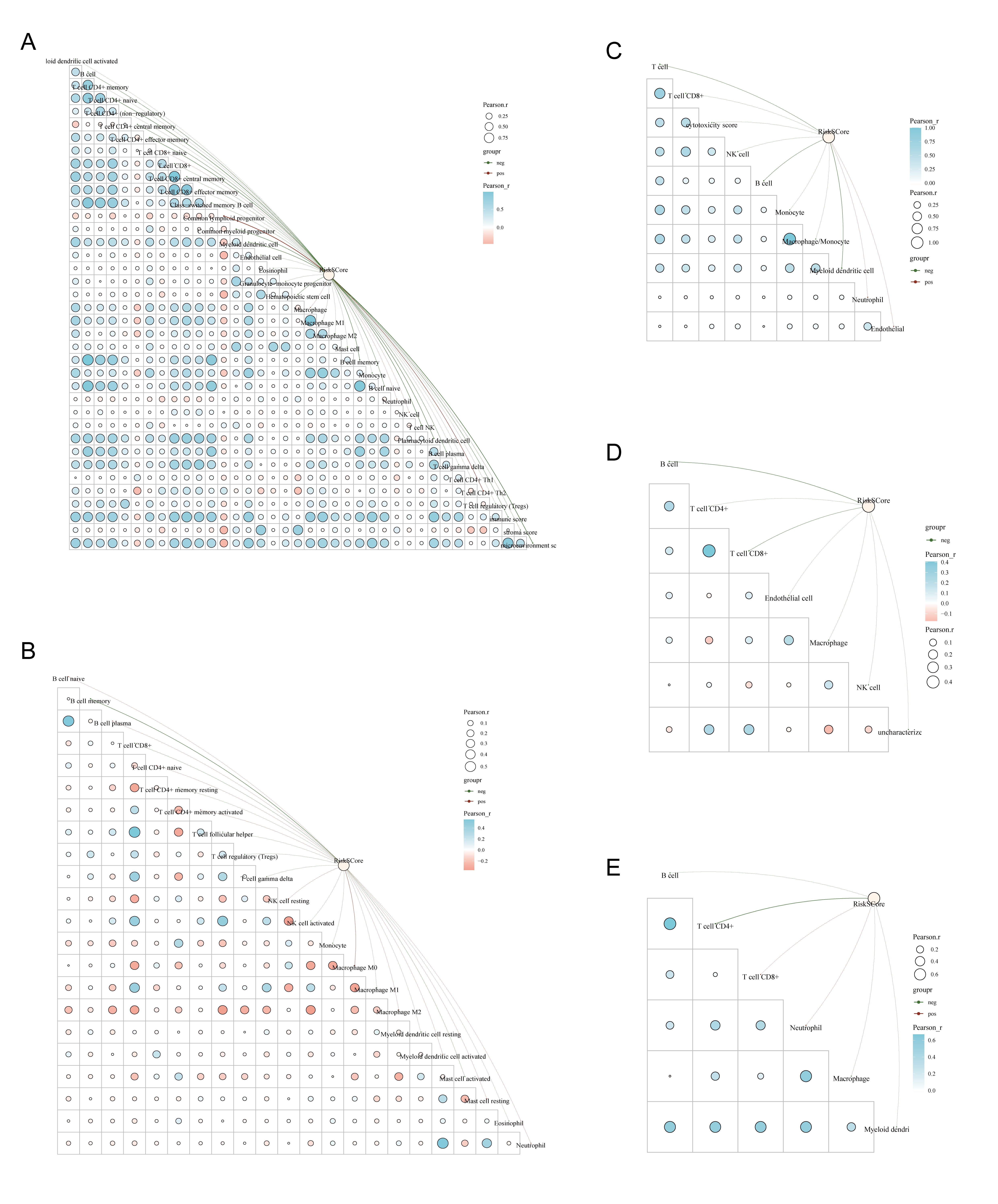


**Supplementary Figure 5** Relationship between Riskscore and immune scores through different algorithms. (A) xCell, (B) CIBERSORT, (C) MCPcounter, (D) EPIC, (E) TIMER.


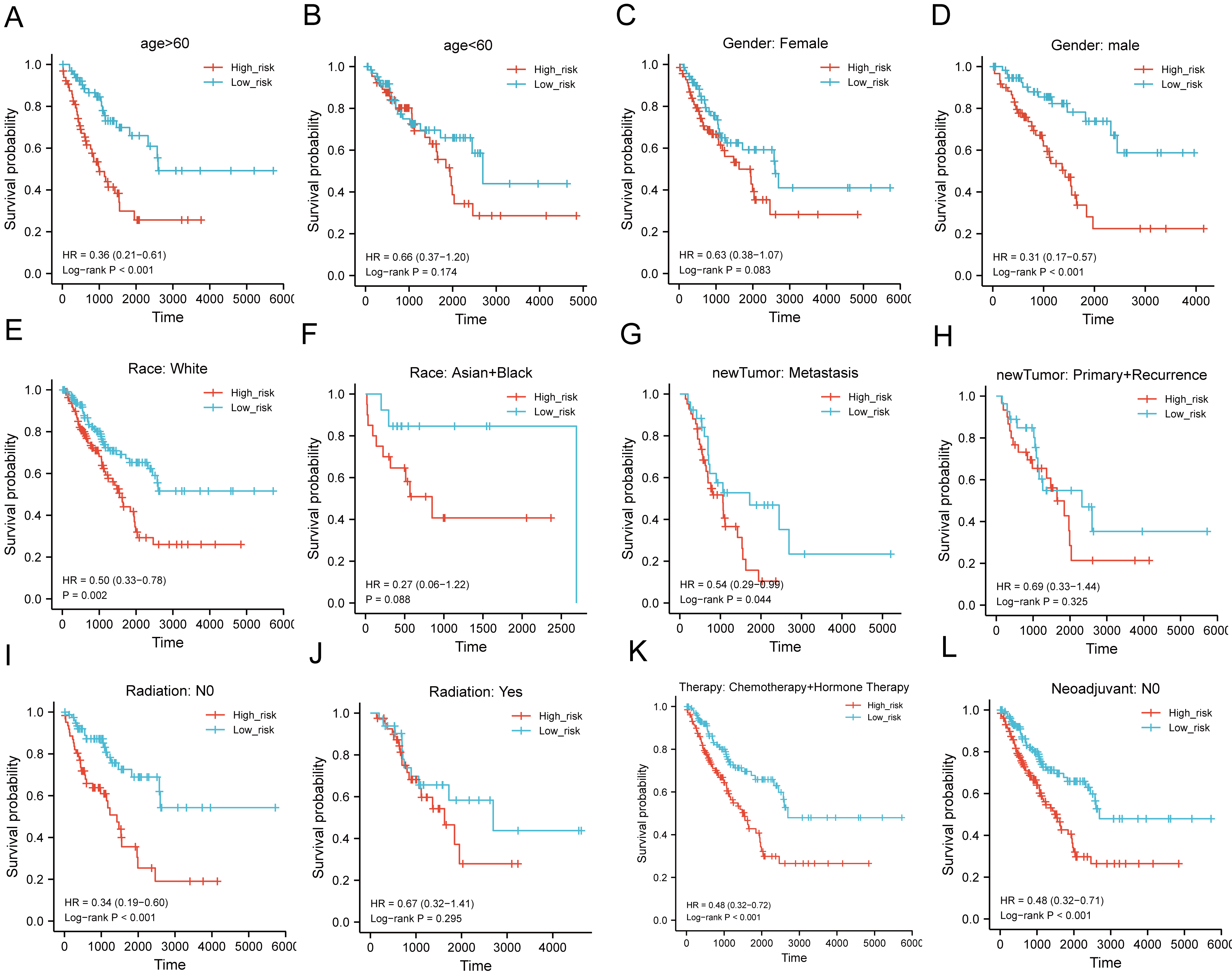


**Supplementary Figure 6.** Survival analysis of risk scores and subgroups in SARC. Survival curves for high-risk and low-risk patients in different subgroups of the TCGA cohort. (A) age > 60, (B) age ≤ 60, (C) Female, (D) Male, (E) White, (F) Asian + Black, (G) Metastasis, (H) Primary + Recurrence, (I) No Radiation, (J) Yes Radiation, (K) Chemotherapy + Hormone Therapy, (L) No Neoadjuvant.
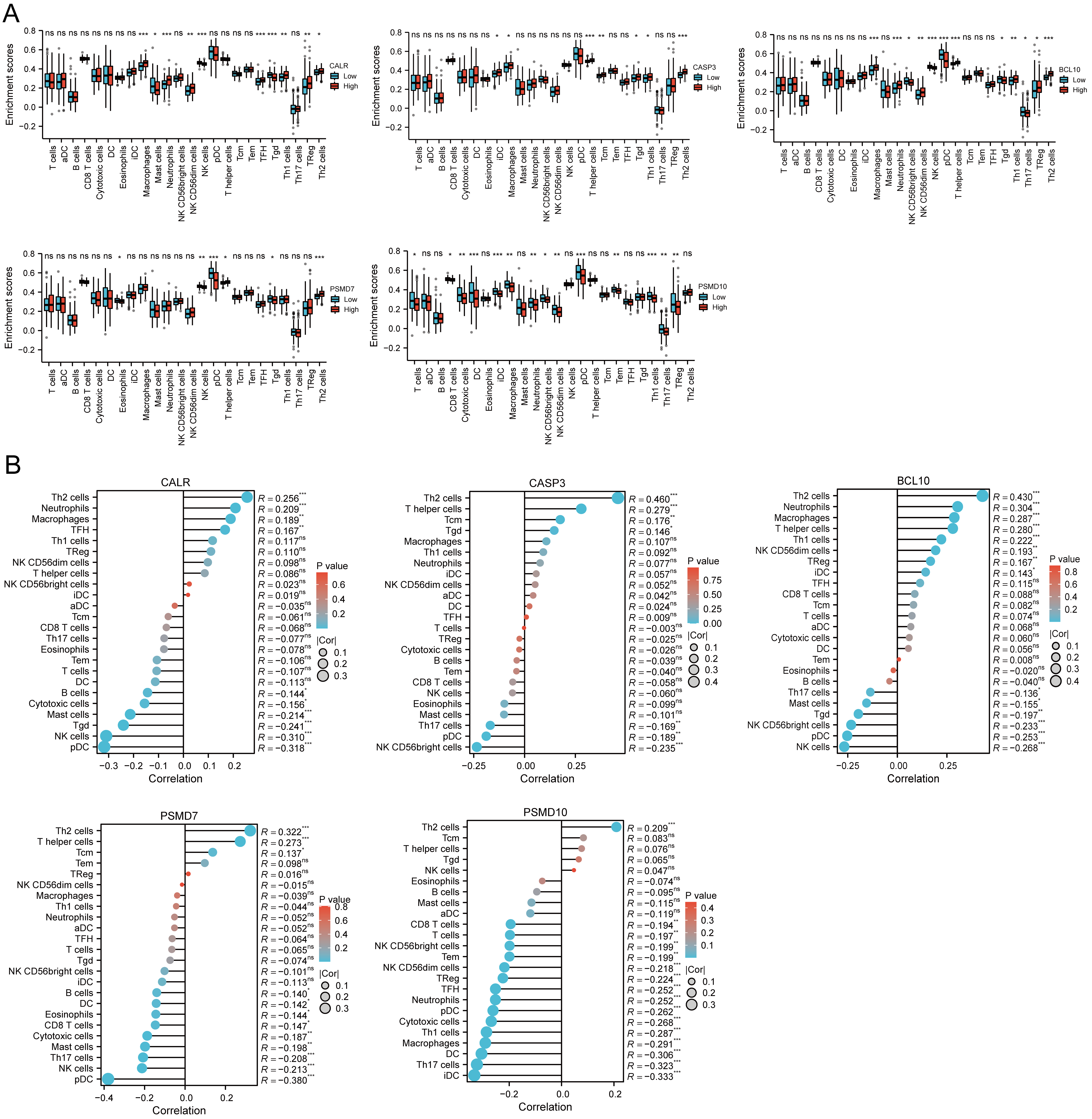


**Supplementary Figure 7.** The relationship between 5 URGs and tumor immune cell infiltration in SARC. (A) Differences in immune cell infiltration between high and low expression groups of URGs. (B) Correlation analysis between URGs and tumor immune cell infiltration.


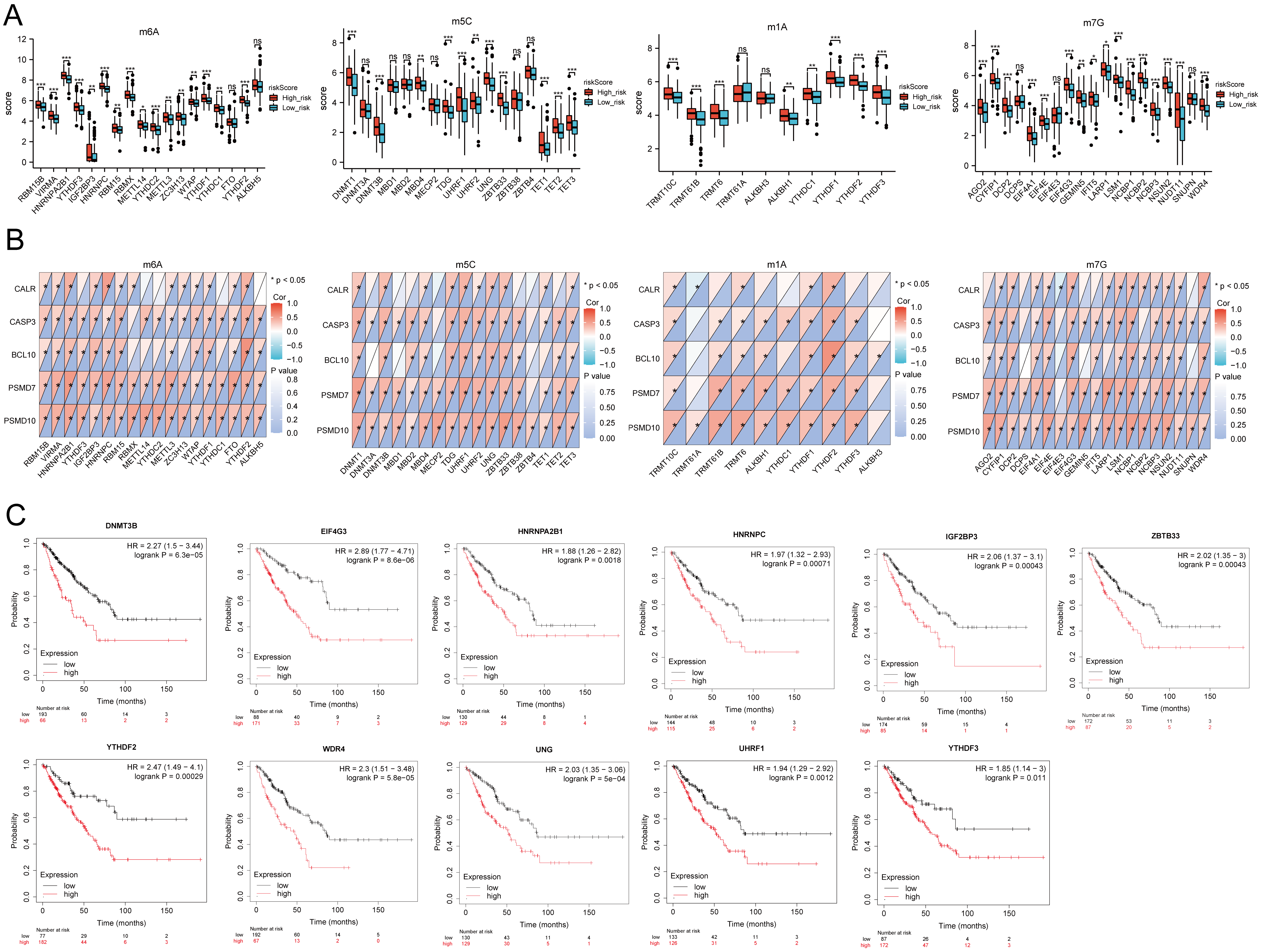


**Supplementary Figure 8.** The association between prognostic URGs and genes related to pan-RNA epigenetic modifications. (A) Differential expression of m6A, m5C, m1A, and m7G between high-risk and low-risk groups. (B) Correlation analysis between 5 prognostic URGs and genes related to m6A, m5C, m1A, and m7G in the TCGA-SARC cohort. (C) Survival curves of DNMT3B, EIF4G3, HNRNPA2B1, HNRNPC, IGF2BP3, ZBTB33, YTHDF2, WDR4, UNG, UHRF1, and YTHDF3.


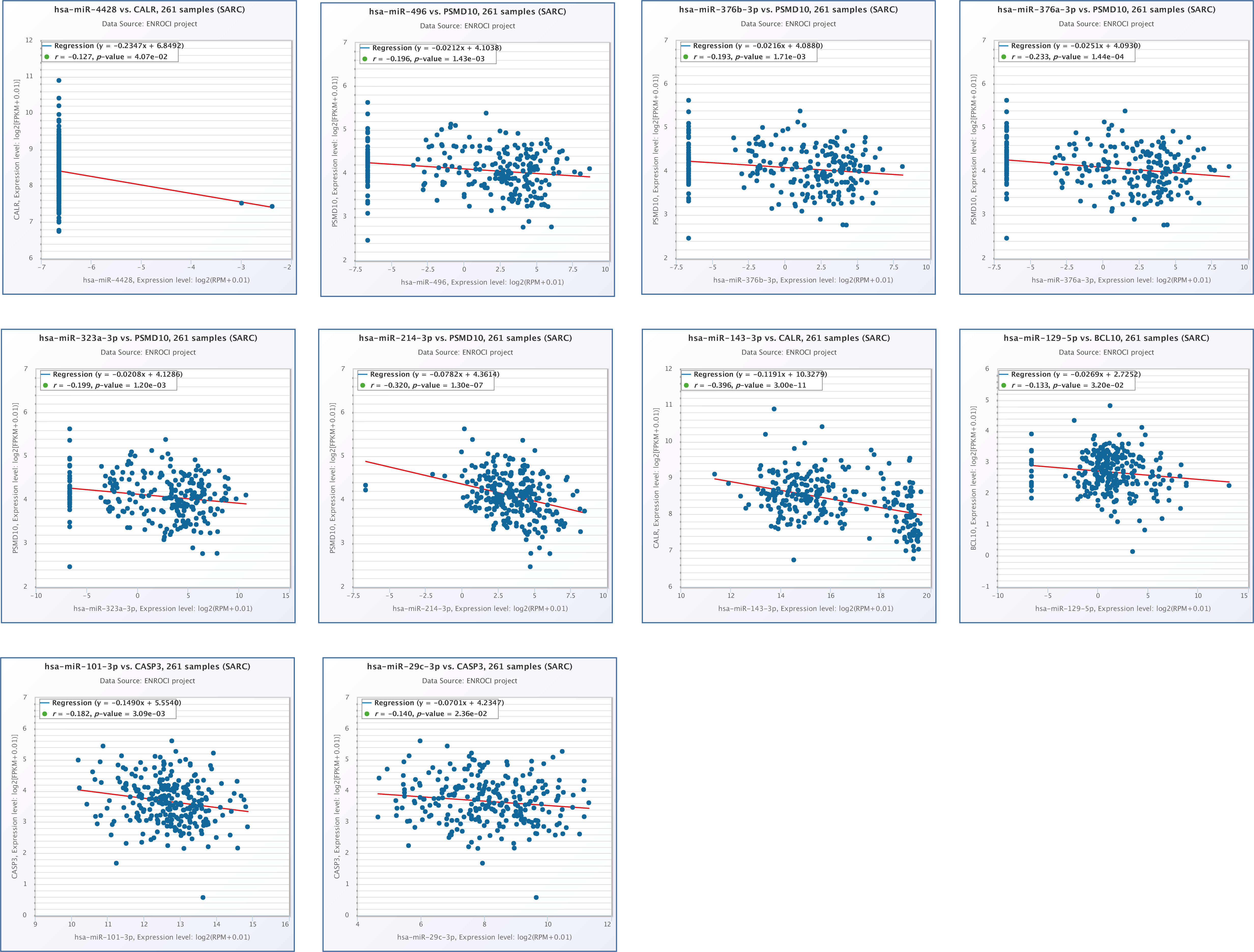


**Supplementary Figure 9.** Correlation between potential miRNAs and URGs mRNA in SARC.
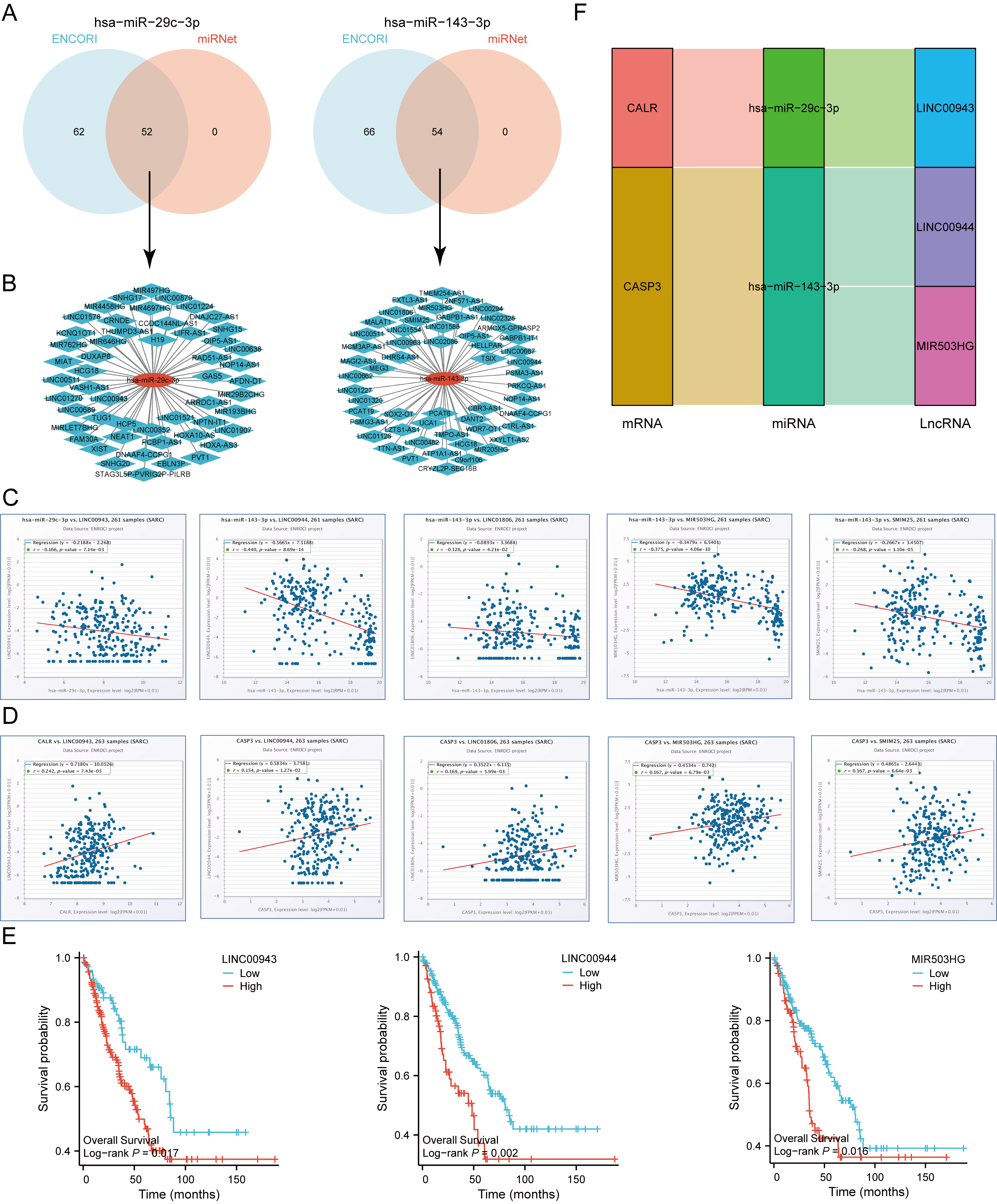


**Supplementary Figure 10.** Screening of the LncRNA-miRNA-URGs regulatory axis in SARC. (A) Prediction of potential lncRNAs for hsa-miR-29c-3p and hsa-miR-143-3p using ENCORI and miRNet databases; (B) Construction of the potential miRNA-lncRNA network using Cytoscape software; (C) Correlation of potential lncRNAs with hsa-miR-29c-3p and hsa-miR-143-3p in SARC; (D) Correlation of potential lncRNAs with prognostic URGs (CALR, CASP3) in SARC; (E) Prognostic value of potential lncRNAs in SARC; (F) mRNA-miRNA-lncRNA triple regulatory network affecting the prognosis of SARC.
